# Palladium-catalysed C–F alumination of fluorobenzenes: mechanistic diversity and origin of selectivity†

**DOI:** 10.1039/d0sc01915a

**Published:** 2020-07-21

**Authors:** Feriel Rekhroukh, Wenyi Chen, Ryan K. Brown, Andrew J. P. White, Mark R. Crimmin

**Affiliations:** Department of Chemistry, Molecular Sciences Research Hub, Imperial College London 80 Wood Lane, Shepherds Bush London W12 0BZ UK m.crimmin@imperial.ac.uk

## Abstract

A palladium pre-catalyst, [Pd(PCy_3_)_2_] is reported for the efficient and selective C–F alumination of fluorobenzenes with the aluminium(i) reagent [{(ArNCMe)_2_CH}Al] (**1**, Ar = 2,6-di-iso-propylphenyl). The catalytic protocol results in the transformation of sp^2^ C–F bonds to sp^2^ C–Al bonds and provides a route to reactive organoaluminium complexes (**2a–h**) from fluorocarbons. The catalyst is highly active. Reactions proceed within 5 minutes at 25 °C (and at appreciable rates at even −50 °C) and the scope includes low-fluorine-content substrates such as fluorobenzene, difluorobenzenes and trifluorobenzenes. The reaction proceeds with complete chemoselectivity (C–F *vs.* C–H) and high regioselectivities (>90% for C–F bonds adjacent to the most acidic C–H sites). The heterometallic complex [Pd(PCy_3_)(**1**)_2_] was shown to be catalytically competent. Catalytic C–F alumination proceeds with a KIE of 1.1–1.3. DFT calculations have been used to model potential mechanisms for C–F bond activation. These calculations suggest that two competing mechanisms may be in operation. Pathway 1 involves a ligand-assisted oxidative addition to [Pd(**1**)_2_] and leads directly to the product. Pathway 2 involves a stepwise C–H → C–F functionalisation mechanism in which the C–H bond is broken and reformed along the reaction coordinate, guiding the catalyst to an adjacent C–F site. This second mechanism explains the experimentally observed regioselectivity. Experimental support for this C–H activation playing a key role in C–F alumination was obtained by employing [{(MesNCMe)_2_CH}AlH_2_] (**3**, Mes = 2,4,6-tri-methylphenyl) as a reagent in place of **1**. In this instance, the kinetic C–H alumination intermediate could be isolated. Under catalytic conditions this intermediate converts to the thermodynamic C–F alumination product.

## Abstract 2

A palladium pre-catalyst, [Pd(PCy_3_)_2_] is reported for the efficient and selective C–F alumination of fluorobenzenes with the aluminium(i) reagent [{(ArNCMe)_2_CH}Al] (Ar = 2,6-di-iso-propylphenyl).

## Graphical abstract

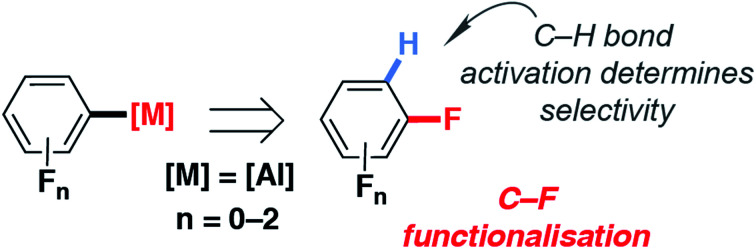

## Introduction

**Fig. 1:**
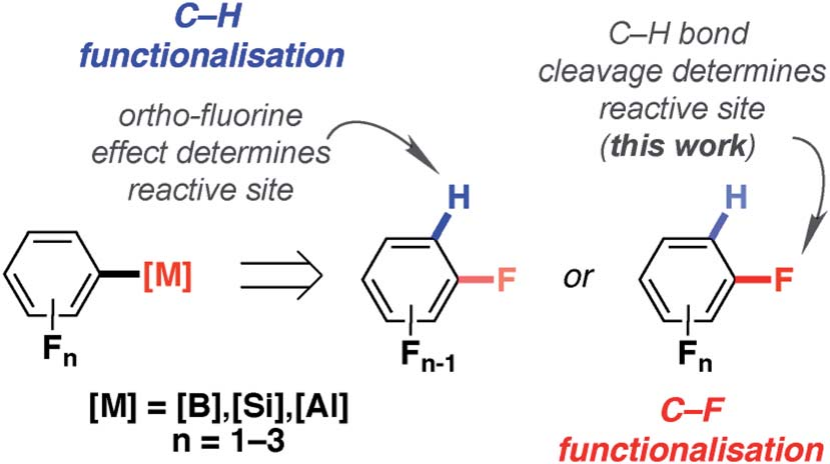
C–H and C–F functionalisation of fluorobenzenes.

The importance of fluorine in synthetic chemistry has inspired methods to generate reactive building blocks from commercial and inexpensive fluorobenzenes such as fluorobenzene (FB), difluorobenzenes (diFBs) and trifluorobenzenes (triFBs).^1–4^ These studies are motivated by the importance of fluorine as both a hydrogen isostere and a radiolabel in drug discovery.^5–7^ Two approaches have emerged: C–H and C–F bond functionalisation (Fig. 1).

For C–H bond functionalisation, the selectivity is often dictated by the *ortho*-fluorine effect, with C–H bonds being flanked by one or more fluorine atoms being the most reactive sites.^8,9^ 1,2-diFB and FB are particularly challenging substrates in these reactions, leading some to explore novel strategies for substrate activation. These include the reversible generation of π-coordinated arene complexes as a means to decrease the p*K*_a_ of the C–H bonds,^10,11^ along with stepwise carboxylation/decarboxylation to obtain *meta*-substitution products from FB.^12^

An alternative method to prepare fluorinated building blocks from fluorobenzenes is C–F bond functionalisation. A number of non-catalysed, photo-catalysed and transition metal catalysed methods for the borylation,^13–20^ magnesiation,^21–24^ alumination^25–28^ and silylation^29–33^ of fluorobenzenes have emerged in recent years. The advantage of these approaches is that they form new C–B, C–Mg, C–Al and C–Si bonds respectively, allowing access to main group intermediates that can be used in onwards reactions. Due to established trends in C–F bond strengths,^34^ the generation of reactive building blocks from low-fluorine-content substrates (C_6_H_6−*n*_F_*n*_, *n* ≤ 3) is considerably more challenging than for high-fluorine-content analogues (C_6_H_6−*n*_F_*n*_, *n* > 3). When catalysts or reagents have been found to react with these substrates, chemoselectivity and regioselectivity become a critical issue.

For example, the copper-catalysed *ipso*-borylation of (poly)fluorinated benzenes with low-fluorine-content substrates proceeds with poor chemoselectivity leading to (poly)borylated products with complete exchange of C–F for C–B bonds.^35^ Similarly, the borylation of triFBs with nucleophilic [B(CN)_3_]^2−^ is facile, but when multiple isomers can form, low regioselectivity is observed.^36^ A rare example of a catalytic system that operates with high selectivity has been reported by Marder, Radius and co-workers.^37,38^ The nickel-catalysed C–F borylation of FBs, diFBs and triFBs proceeds with excellent chemoselectivity. In cases where regioselectivity is an issue, bond functionalisation was found to occur with high selectivity (>10 : 1) for C–F positions that are adjacent to the most acidic C–H bonds. The precise origin of this selectivity remains unclear.

**Fig. 2:**
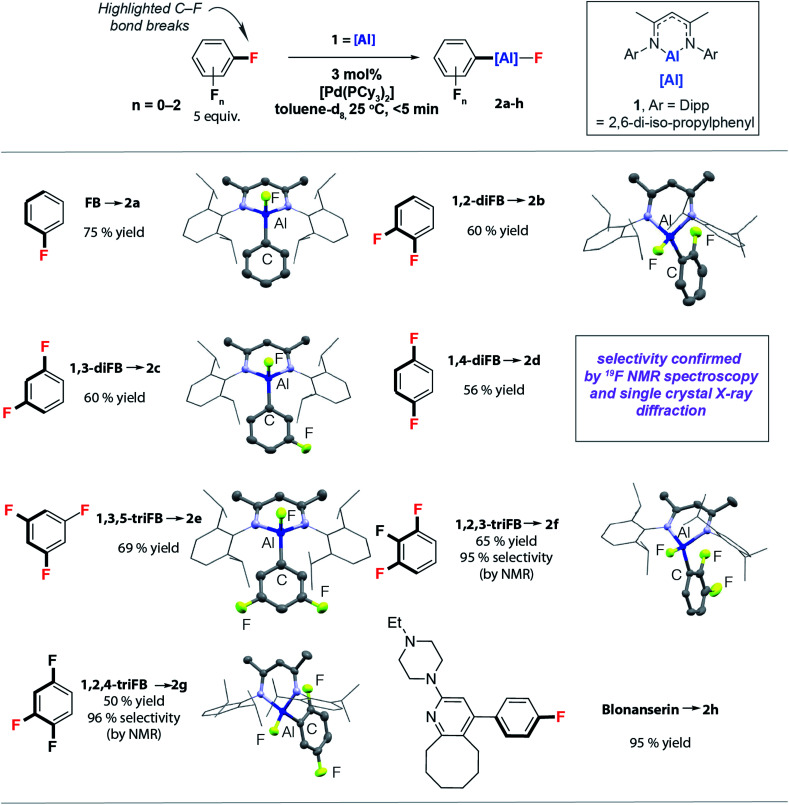
Palladium-catalysed C–F bond alumination of FB, diFBs and triFBs.

In this paper, we report an exceptionally mild (25 °C, <5 min) catalytic approach to the conversion of C–F bonds of FB, diFBs and triFBs to C–Al bonds. We show that the aluminium reagent **1** (Fig. 2), which is known to react with high-fluorine-content substrates in the absence of a catalyst,^25,26,39^ can effect C–F alumination of low-fluorine-content substrates on addition of catalytic [Pd(PCy_3_)_2_]. High selectivity (20 : 1) is observed for reaction sites adjacent to acidic C–H bonds and this selectivity parallels that observed in the hydrodefluorination of polyfluorinated substrates with related aluminium reagents and precious metal catalysts.^27,28^ We provide experimental and computational evidence that supports two competing mechanisms. One of these new pathways explains the regioselectivity and provides the rare mechanistic insight into how the reversible breaking of a C–H bond can determine the regioselectivity of catalytic C–F bond functionalisation.

## Results and discussion

### Reaction scope

The reactions of the monomeric aluminium(i) complex **1**^40,41^ with FB, diFBs and triFBs catalysed by 3 mol% [Pd(PCy_3_)_2_] in benzene or toluene solutions proceed extremely rapidly at 25 °C. Facile C–F bond alumination to form **2a–h** was observed. In all cases the reactions were complete within the acquisition of the first time point (<5 min) as evidenced by ^19^F NMR spectroscopy (Fig. 2). Even more surprisingly, low temperature experiments indicate that **2a** and **2c** were formed in high yield at −30 °C and −50 °C, respectively. There is no appreciable reaction of these substrates with **1** in the absence of a catalyst at 25 °C or below. At higher temperatures slow and non-selective C–F alumination of triFBs could be observed. For example, 1,2,3-triFB yields a 1 : 3 mixture of regioisomers from C–F alumination with **1** in 63% yield after 96 h at 80 °C with the major product resulting from reaction of the central C–F bond of the three. Complementary regioselectivity is observed during catalysis. When more than one regioisomer is possible, high selectivity is recorded for the functionalisation of C–F bonds adjacent to acidic C–H bonds. Hence, 1,2,3-triFB and 1,2,4-triFB lead to products in which the aluminium fragment is installed next to an existing C–H bond (Fig. 2). The high activity and selectivity of the catalytic protocol means it can be applied to the late-stage functionalisation of complex molecules. For example, the palladium catalysed reaction of **1** with Blonanserin, an active pharmaceutical ingredient with a fluorobenzene motif, proceeds rapidly at 25 °C to form **2h**.^31^

### An off-cycle intermediate and KIEs

**Fig. 3:**
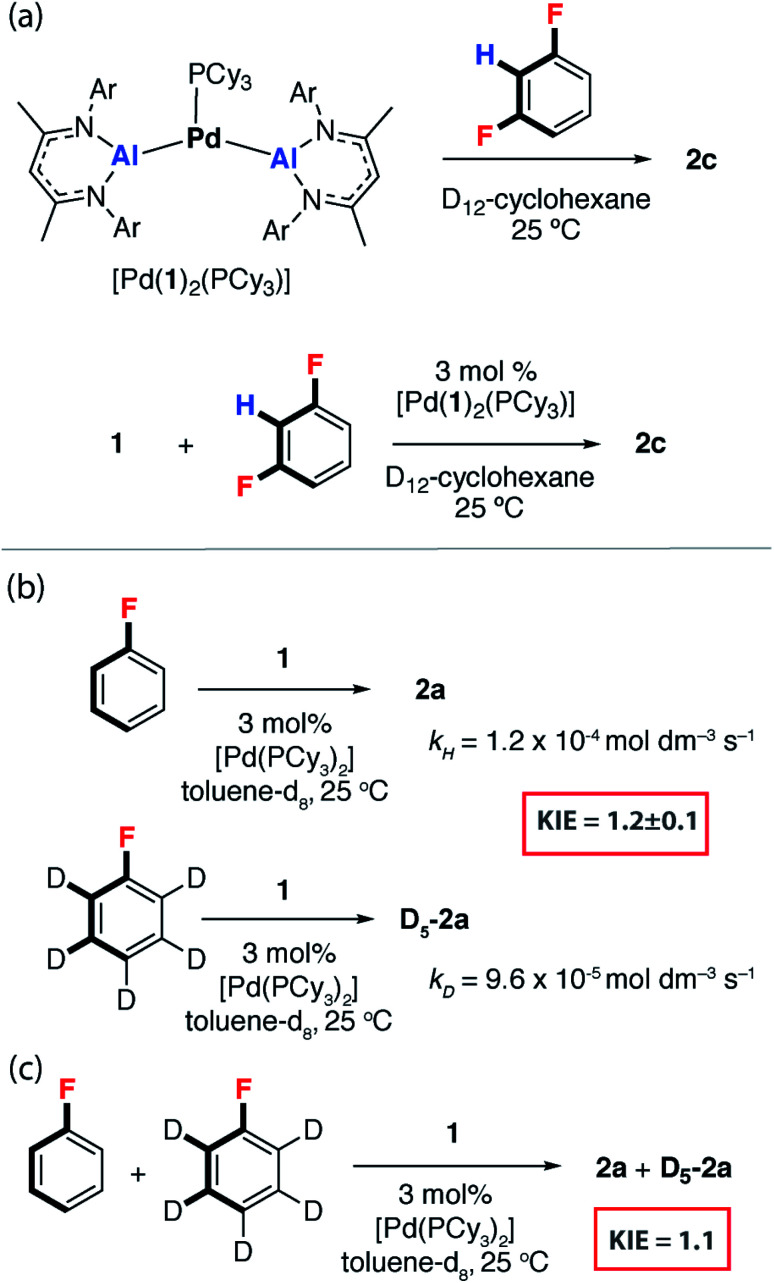
(a) [Pd(**1**)_2_(PCy_3_)] as a catalyst for C–F alumination of 1,3-diFB. (b) KIE in the reaction of FB with **1** through measurement of *k*_obs_ and (c) an intermolecular competition experiment.

We have previously reported [Pd(PCy_3_)_2_] mixtures as highly active catalysts for the C–H alumination of benzene, toluene and xylenes with **1**, [Pd(**1**)_2_(PCy_3_)] was identified as an off-cycle resting state in catalysis.^42^ This complex reacts stoichiometrically with 1,3-diFB to form **2c** (Fig. 3a). Similarly, [Pd(**1**)_2_(PCy_3_)] was catalytically competent for the C–F bond alumination of 1,3-diFB with **1** in cyclohexane solution at 25 °C (Fig. 3a). Curious as to whether C–H activation plays a role in reactions described herein, the KIE for the reaction of **1** with fluorobenzene and fluorobenzene-d_5_ was measured by two different approaches. A KIE of 1.2 ± 0.1 was measured by relative rates (Fig. 3b) and a KIE of 1.1 (Fig. 3c) was determined by intermolecular competition. These experiments reveal a small isotope effect that is most conservatively interpreted as a secondary KIE. Related palladium-catalysed reactions of **1** with benzene and furan involve turnover limiting C–H and activation have been recorded with KIEs ranging between 4–6. These data suggest that in the case of FB, breaking of the C–H bond is unlikely to be involved in the turnover-limiting step.^43^

### DFT calculated mechanism

To gain a deeper understanding of the KIE and the origin of selectivity, a series of plausible mechanisms were investigated by DFT calculations using the M06L functional. Although 1,3-diFB was the initial focus of these calculations key transition states have been located for a number of substrates (*vide infra*). Two distinct mechanisms were found to be viable: the first involves a ligand-assisted oxidative addition step to break the C–F bond and form the product in a concerted step (pathway 1), the second, more complicated pathway, is based on a stepwise C–H → C–F functionalisation process (pathway 2).

**Fig. 4:**
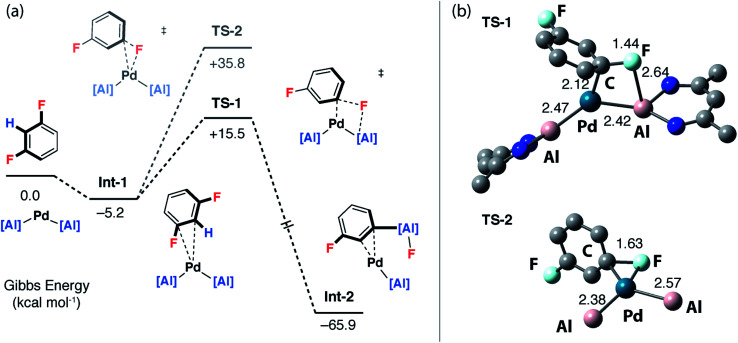
Pathway 1: (a) DFT calculated ligand-assisted oxidative addition pathway. (b) Geometries of ligand-assisted oxidative addition transition state (**TS-1**, di-iso-propylphenyl groups omitted for clarity) and direct oxidative addition to Pd transition state (**TS-2**, β-diketiminate ligands omitted for clarity) with bond lengths (Å).

Calculations were initiated from [Pd(**1**)_2_] a proposed on-cycle intermediate formed from ligand dissociation from [Pd(**1**)_2_(PCy_3_)].^42^ 1,3-diFB can associate with this 14-electron bent Pd(0) fragment leading to the formation of an encounter complex **Int-1**. **TS-1** was identified as a low-energy transition state (Δ*G*^‡^ = 20.7 kcal mol^−1^) that connects directly to **Int-2**, a palladium complex of the product **2c** (Fig. 4a – pathway 1). **TS-1** involves the ligand-assisted oxidative addition of the C–F bond of 1,3-diFB to [Pd(**1**)_2_]. This pathway relies on the participation of the vacant 3p-orbital on the aluminylene ligand in a four-membered transition state for C–F bond-breaking (Fig. 4b). Macgregor, Braun and coworkers have proposed a related ligand-assisted oxidative addition pathway involving the addition of a C–F bond of pentafluoropyridine to a Rh–boryl complex.^13^ In contrast, the direct C–F oxidative addition to Pd in the absence of ligand-assistance (**TS-2**, Δ*G*^‡^ = 41.0 kcal mol^−1^) is not expected to be competitive with the other pathways (Fig. 4).

**Fig. 5:**
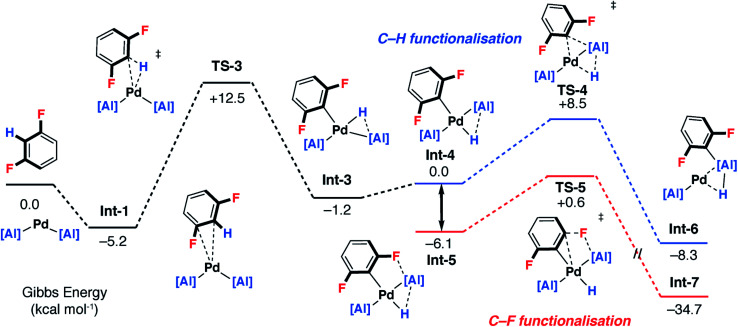
Pathway 2: DFT calculated stepwise pathway for C–H → C–F functionalisation (part 1).

**Fig. 6:**
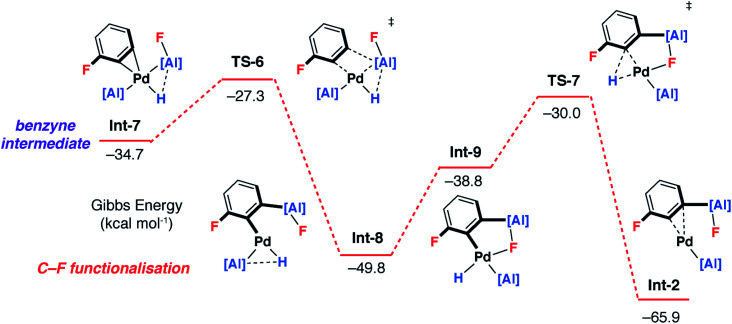
Pathway 2: DFT calculated stepwise pathway for C–H → C–F functionalisation (part 2).

A plausible mechanism involving C–H activation was also identified from **Int-1** (Fig. 5 and 6 – pathway 2). Breaking of the C–H bond is predicted to be slightly endergonic and forms **Int-3** by a classical three-centred oxidative addition transition state, **TS-3**, with an activation barrier of Δ*G*^‡^ = 17.7 kcal mol^−1^. **Int-3** can undergo a *cis*–*trans* isomerisation process affording **Int-4** and ultimately the more stable isomer, **Int-5**. From **Int-5** there are two plausible pathways, a low-energy and non-reversible pathway to form the C–F alumination product and a higher energy and potentially reversible pathway to form the C–H alumination product.

Hence, isomerisation of **Int-5** back to **Int-4** followed by a concerted double-migration of both the phenyl and hydride ligands on Pd to Al may occur with a modest energy activation barrier (Δ*G*^‡^ = 14.6 kcal mol^−1^) leading to the formation of Al–C and Al–H bonds in **Int-6**. We have previously identified this step by DFT in a closely related mechanism.^42^ Both are substantiated by experimental data including benchmarking of the DFT methods by experimentally determined activation parameters. Aldridge and coworkers have identified a related double-migration pathway through the analysis of a series of crystallographic snapshots of gallium–rhodium hydride complexes.^44^ Dissociation of the σ-alane ligand from **Int-6** would liberate the C–H alumination product and regenerate a catalytically active Pd fragment.

Alternatively, C–F bond breaking may occur directly from **Int-5**. **Int-5** possesses a periplanar arrangement between the Al and the F centres. NBO calculations identified a stabilizing donor–acceptor interaction between the lone pair of the F atom and the vacant p-orbital on Al. This intermediate is perfectly organised for C–F bond activation. Fluoride abstraction occurs with a readily accessible local Gibbs activation barrier (Δ*G*^‡^ = 6.7 kcal mol^−1^). **TS-5** is a late transition state in line with the Al⋯F bond length (1.79 Å), C⋯F bond length (1.97 Å) and the C≡C bond length (1.31 Å), that results in the formation of a benzyne intermediate **Int-7**. Insertion of the benzyne ligand into the Al–Pd bond proceeds *via***TS-6** (Δ*G*^‡^ = 7.4 kcal mol^−1^). The Al⋯C (2.18 Å) and the Pd⋯C (2.30 Å) bond lengths in **TS-6** are consistent with a late transition state. The resulting complex **Int-8**, can undergo an isomerisation and subsequent reductive elimination by **TS-7**, to form the C–F alumination product **Int-2**. This step has the highest activation barrier and could be considered to be turnover limiting (Δ*G*^‡^ = 19.8 kcal mol^−1^). **Int-2**, is formed as the most thermodynamically stable product of the reaction 
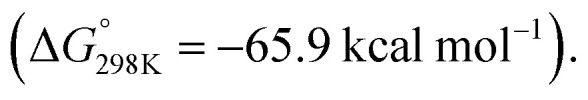
 Dissociation of **2c** from **Int-2** would liberate the C–F alumination product and regenerate a catalytically active Pd fragment.

While uncommon, the generation of benzyne intermediates during C–F bond activation has been observed experimentally. For example, Jones and Hughes prepared tetrafluorobenzyne compounds *via ortho*-fluoride abstraction from pentafluorophenyl ligands on zirconium.^45–49^ The structure and bonding situation of **Int-7** were further investigated by DFT: the optimized geometry shows an η^2^ coordination of the benzyne triple bond. The palladium centre has a trigonal planar arrangement characteristic of [M(η^2^-alkyne)L_2_] complexes. The length of the coordinated triple bond is similar to a related Pd η^2^-benzyne complexes.^50^ The bonding situation in **Int-7** was further examined by NBO analysis. At the second-order perturbation level, donation from the triple bond of the benzyne ligand to Pd-based orbitals is apparent (ESI†).

A number of mechanistic studies have concluded on the potential for C–H activation as a prerequisite for C–F bond functionalisation. For example, Goldman and co-workers proposed a mechanism for the net oxidative addition of the sp^3^ C–F bond of fluoroethane to an iridium pincer complex involving stepwise C–H activation followed by β-fluoride elimination.^51^ Similarly, Braun and coworkers very recently described the reaction of 2,3,3,3-tetrafluoropropene with a Rh(i) complex that, in the presence of a fluorosilane, proceeds by an initial C–H activation step followed by a 1,2-fluorine atom shift.^52^ While mechanistic data for these stepwise process operating for fluoroarenes is less well described, Johnson and coworkers have proposed that reversible C–H activation occurs *en route* to non-reversible C–F activation during reactions with *in situ* generated [Ni(PEt_3_)_2_].^53,54^

### Origin of selectivity

For pathway 2, calculations clearly predict the C–F functionalisation pathway to be kinetically favoured and lead to the thermodynamic product. In contrast, C–H activation proceeds by a higher energy barrier and leads to a kinetic product. For 1,3-diFB, C–H bond functionalisation is not competitive with C–F bond functionalisation in the forward direction (**TS-4***vs.***TS-5**, ΔΔ*G*^‡^ = 7.9 kcal mol^−1^). For pathway 1, there is no issue of chemoselectivity. Experimentally, **1** and 1,3-diFB leads exclusively to **2c** with no evidence for C–H functionalisation.

Which of the two pathways dominates is expected to differ based on the well-understood trends in C–H and C–F bond strengths of fluoroarenes. Ligand-assisted oxidative addition (pathway 1) is likely to be favoured for substrates with the strongest and least acidic C–H bonds and should proceed to break C–F bonds flanked by additional fluorine atom(s). These are the weakest C–F bonds^34^ and lead to the formation of the strongest C–M bonds.^8,55^ The stepwise C–H → C–F functionalisation process (pathway 2) should be favoured for substrates with more acidic C–H bonds.^8,55^ The C–H bond is broken and reformed in the mechanism, guiding the catalyst to an adjacent C–F bond. There is a strict *ortho* relationship between the two reactive sites which determines the regioselectivity of the reaction. The DFT studies predict that neither pathway involves the breaking of the C–H bond in the turnover-limiting step. Therefore, neither mechanism predicts a strong primary KIE.

In order to better understand the interplay of the two possible mechanisms additional substrates were considered. Key transition states for FB and 1,2,3-triFB were calculated and compared to those of 1,3-diFB (Table 1). It appears that while both mechanisms may be in operation, variation of the substrate influences the barriers of these transition states and may led to switches in the favoured mechanism as the fluorine content of the substrate changes. For FB, ligand-assisted oxidative addition is expected to be the dominant pathway, while for 1,3-diFB the stepwise C–H → C–F functionalisation process is expected to operate exclusively. Based on our current understanding, the stepwise C–H → C–F functionalisation mechanism is likely the dominant mechanism at play for diFBs and triFBs as it provides the clearest rationale for the origin of the regioselectivity for these substrates (Fig. 2).

**Table 1:** Comparison of DFT calculated activation barriers for key steps in pathway 1 and 2

	FB	1,3-DiFB	1,2,3-TriFB
**TS-1** pathway 1 (Δ*G*^‡^ kcal mol^−1^)	**21.4**	20.7	22.7^a^
**TS-3** pathway 2 (Δ*G*^‡^ kcal mol^−1^)	23.0	**17.7**	22.8^a^

a For reaction at the 1/3-position.

### C–H bond activation

**Fig. 7:**
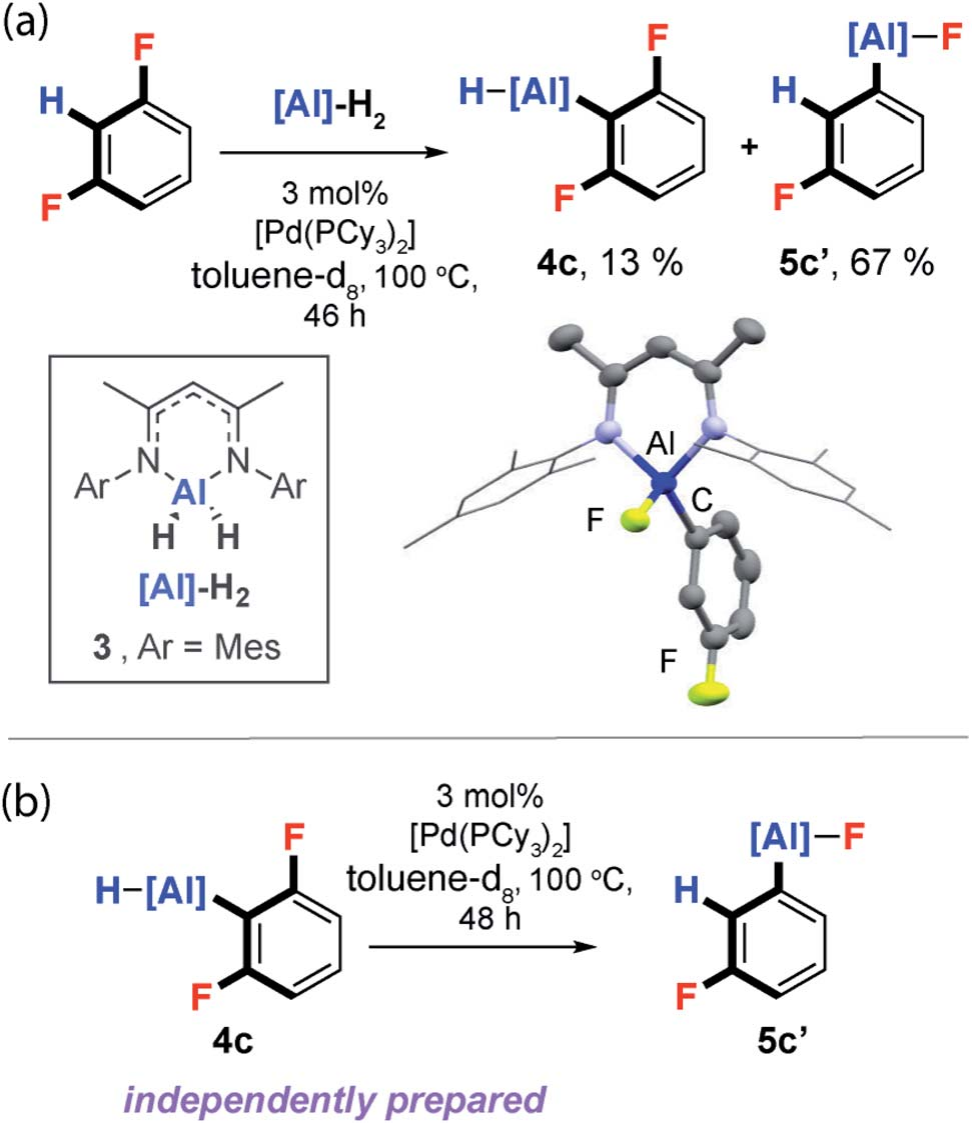
(a) Reaction of **3** with 1,3-diFB. (b) Isomerisation of **4c** to **5c′**.

Unambiguous support for C–H activation playing a role in C–F bond functionalisation was obtained during reactions of the aluminium(iii) dihydride **3**, an analogue of **1**, with 1,3-difluorobenzene. Reaction of **3**, 3 mol% [Pd(PCy_3_)_2_] and 1,3-diFB at 100 °C in toluene-d_8_ yielded a mixture of C–H and C–F functionalised products **4c** and **5c′** respectively (Fig. 7a). Monitoring this reaction as a function of time by ^19^F NMR spectroscopy, revealed slow consumption of **4c** at longer time points, suggesting this species may be a kinetic product which can equilibrate to the thermodynamic product **5c′**. While independently prepared samples of **4c** did not convert to **5c′** under thermal conditions, addition of catalytic quantities of [Pd(PCy_3_)_2_] exposed an unprecedented and 100% atom efficient isomerisation reaction which interconverts the C–H functionalisation aluminium hydride to a C–F functionalised aluminium fluoride (Fig. 7b).^56^ From the perspective of the DFT calculations, the data represent the verification of an entry point into the mechanistic manifold from **Int-6** and conversion of the kinetic to thermodynamic product.

The isomerisation pathway represents a rare class of transition metal catalysed process that leads to the molecular rearrangement of a reactive organometallic compound. Further reactions of **3** with 1,2-diFB, 1,2,3-triFB and 1,3,5-triFB also led to isomeric mixtures of C–H and C–F bond activation products (ESI†).^57^

## Conclusions

In summary, we report a highly active catalytic system for the C–F alumination of fluorobenzene. Reactions proceed rapidly at 25 °C and below. The substrate scope includes highly challenging low-fluorine-content substrates (C_6_H_6−*n*_F_*n*_, *n* ≤ 3) and even an active pharmaceutical ingredient. Heterometallic Al–Pd–Al complexes have been proposed as on-cycle intermediates during catalysis. Catalytic C–F bond functionalisation occurs without a strong KIE and DFT calculations suggest that two plausible mechanisms may be in operation. The simplest mechanism (pathway 1) involves the ligand-assisted oxidative addition of the C–F bond of the substrate to a Al–Pd–Al heterometallic complex and proceeds directly to the palladium-bound product. The second more complex mechanism (pathway 2) involves a stepwise C–H → C–F functionalisation process in which the C–H bond breaks and reforms, directing the catalyst to an adjacent C–F site. This latter mechanism provides a rationale for the regioselectivity of the reaction of diFB and triFB under catalytic conditions. Clear experimental support for C–H functionalisation playing a role in catalysis was obtained by the identification of a 100% atom efficient palladium catalysed isomerisation of the kinetic C–H alumination product to the thermodynamic C–F alumination product. The new organoaluminium compounds derived from C–F functionalisation have potential in synthesis. Preliminary experiments show that these are viable partners in a nickel-catalysed cross-coupling with aryl bromides (ESI†). Future studies will focus on generating synthetic value from organoaluminium compounds including those derived from active pharmaceutical ingredients.

## Author contributions

FR and WY conducted the experimental work. RKB conducted reactions with Blonanserin. FR conducted the DFT calculations. AJPW collected and analysed single crystal X-ray diffraction data. MRC managed the project. The manuscript was written through contributions of all authors.

## Conflicts of interest

There are no conflicts to declare.

## Supplementary Material

SC-011-D0SC01915A-s001

SC-011-D0SC01915A-s002
