# Supplementary material for: Palladium-catalysed C–F alumination of fluorobenzenes: mechanistic diversity and origin of selectivity
Source: Chem Sci. 2020 Jul 21;11(30):7842–9. doi: 10.1039/d0sc01915a (PMC8163258; doi:10.1039/d0sc01915a)
Supplement: SC-011-D0SC01915A-s001 [file SC-011-D0SC01915A-s001.pdf]

Electronic Supplementary Material (ESI) for Chemical Science.
This journal is © The Royal Society of Chemistry 2020

Electronic Supplementary Information for:

Palladium-Catalysed C–F Alumination of Fluorobenzenes: Mechanistic Diversity and Origin of Selectivity

*Feriel Rekhroukh, Wenyi Chen, Ryan K. Brown, Andrew J. P. White and Mark R. Crimmin**

Department of Chemistry, Molecular Sciences Research Hub, Imperial College London, 80 Wood Lane, Shepherds Bush, London, W12 0BZ, UK.

**Corresponding author. E-mail: m.crimmin@imperial.ac.uk*

Table of contents

1- Materials and methods S3

2- Experimental procedures and analytical data S4

3- NMR spectra S29

4- X-ray Crystallographic Data S63

5- Computational details S72

1- Materials and methods

All reactions and manipulations were carried out under an atmosphere of dry argon or dinitrogen using standard Schlenk techniques or in a glovebox under inert atmosphere.

Solvents for air sensitive procedures (toluene, n-hexane) were dried using a solvent purification system (SPS) and stored over activated 3 Å molecular sieves under an inert atmosphere of N_2 or argon before use. C_6H_6 (Sigma-Aldrich anhydrous grade), C_6D_6 and toluene-d_8 were degassed by the freeze-pump-thaw method (x 3) and stored under inert atmosphere over activated 3 Å molecular sieves. Fluoroarenes, aryl bromides and heteroarenes were purchased as anhydrous reagents (Sigma-Aldrich, Acros or Fluorochem) and further dried over 3 Å molecular sieves, degassed by the freeze-pump-thaw method (x 3) and stored under inert atmosphere with activated 3 Å molecular sieves. Dipp-*BDI*Al(I) (**1**), Mes-*BDI*AlH_2 (**3**) and Mes-*BDI*AlHCl (**S1**) were synthesized according to the literature procedure (Ar-*BDI* = {$(ArNCMe)_2CH$}, Ar = 2,4,6-$Me_3C_6H_2$ (Mes) or 2,6-$iPr_2C_6H_3$ (Dipp)).[1] [Pd(PCy_3)$_2$] was synthesised as an analytically pure pale brown crystalline solid from $PdCl_2$ in a 3 step procedure (*via* intermediates [Pd(η^3-C_3H_4Ph)(μ-Cl)]$_2$ and [CpPd(η^3-C_3H_4Ph)]) based on literature reactions.[2] The intermetallic complex [Pd(PCy_3)(Dipp-BDIAl)$_2$] was prepared according to the previously described procedure.[3]

Solution ^1H, ^{13}C, ^{19}F and ^{31}P NMR spectra were recorded on Bruker Avance 400 or 500 spectrometers at 298 K. Chemical shifts (δ) are expressed with a positive sign, in parts per million. ^1H and ^{13}C chemical shifts reported are referenced internally to residual proteo- (^1H) or deutero- (^{13}C) solvent, while ^{19}F chemical shifts are relative to $CFCl_3$ as an external reference. The following abbreviations and their combinations are used: br, broad; s, singlet; d, doublet; t, triplet; q, quartet, m, multiplet.

1 (a) S. Yow, S. J. Gates, A. J. P. White, M. R. Crimmin, *Angew. Chem. Int. Ed.*, 2012, **51**, 12559. (b) C. Cui, H.W. Roesky, H.-G. Schmidt, M. Noltemeyer, H. Hao and F. Cimpoesu, *Angew. Chem. Int. Ed.*, 2000, **39**, 4274. (c) M. Stender, B.E. Eichler, N.J. Hardman, P.P. Power, J. Prust, M. Noltemeyer and H.W. Roesky, *Inorg. Chem.*, 2001, **40**, 2794.

2 (a) P. R. Auburn, P. B. Mackenzie, B. Bosnich, *J. Am. Chem. Soc.*, 1985, **107**, 2033. (b) S. D. Robinson, B. L. Shaw, *J. Chem. Soc.*, 1963, 4806. (c) T. Yoshida, S. Otsuka, *Inorg. Synth.*, 1990, **28**, 114.

3 T. N. Hooper, M. Garçon, A. J. P. White, M. R. Crimmin *Chem. Sci.*, 2018, **9**, 5435.

2- Experimental procedures and analytical data

2-1- Al(I) reactivity with fluoroaromatics

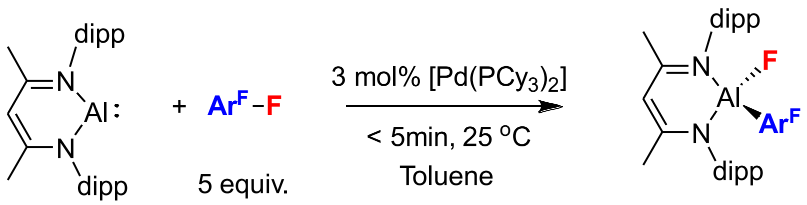

Procedure for the addition of fluoroarenes to **1** and [Pd(PCy_3)$_2$]: In a glovebox, **1** (20 mg, 0.045 mmol, 1 equiv.) and [Pd(PCy_3)$_2$] (134 µL of 0.01 M solution in toluene, 0.00134 mmol, 3 mol%) were weighed into a vial, dissolved in toluene (2 mL) and mixed thoroughly to form a red/orange solution. The fluoroarene was added by micropipette (0.225 mmol, 5 equiv.) and the solution was stirred at room temperature for 5 min. An immediate color change was observed from orange to yellow. The product was isolated by removal of the solvent under vacuum and recrystallisation of the crude mixture from toluene (1 mL) / *n*-hexane (0.5 mL).

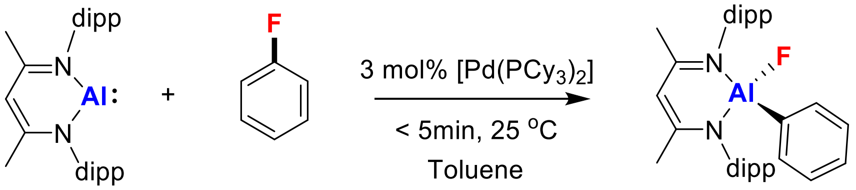

COMPLEX 2a:

Yield: 18 mg, 0.033 mmol (75 %).

^{1}H NMR (400 MHz, C_6D_6): δ 0.84 (d, $^3J_{HH}$ = 6.8 Hz, 6H, CH(C*H*$_3$)$_2$), 1.06 (d, $^3J_{HH}$ = 6.8 Hz, 6H, CH(C*H*$_3$)$_2$), 1.21 (d, $^3J_{HH}$ = 6.8 Hz, 6H, CH(C*H*$_3$)$_2$), 1.45 (d, $^3J_{HH}$ = 6.8 Hz, 6H, CH(C*H*$_3$)$_2$), 1.62 (s, 6H, C*H*$_3$), 3.08 (sept, $^3J_{HH}$ = 6.8 Hz, 2H, C*H*(CH$_3$)$_2$), 3.74 (sept, $^3J_{HH}$ = 6.8 Hz, 2H, C*H*(CH$_3$)$_2$), 5.01 (s, 1H, C(CH$_3$)C*H*C(CH$_3$)), 6.85-6.89 (m, 2H, ArH_{ortho}), 6.89-6.93 (m, 2H, ArH_{metha}), 6.95-6.98 (m, 1H, ArH_{para}), 7.04-7.08 (m, 2H, Ar$_{dipp}$*H*), 7.17-7.20 (m, 4H, Ar$_{dipp}$*H*). **^{19}F NMR** (376.5 MHz, C_6D_6): δ -159.62 (s, 1F, Al–F). **^{13}C{^{1}H} NMR** (100 MHz, C_6D_6): δ 23.22 (s, *C*H$_3$), 23.69 (s, CH(*C*H$_3$)$_2$), 24.52 (s, CH(*C*H$_3$)$_2$), 24.70 (s, CH(*C*H$_3$)$_2$), 25.71 (s, CH(*C*H$_3$)$_2$), 28.24 (s, *C*H(CH$_3$)$_2$), 29.03 (s, *C*H(CH$_3$)$_2$), 98.31 (s, C(CH$_3$)*C*HC(CH$_3$)), 124.44 (s, *C*H$_{dipp}$), 125.17 (s, *C*H$_{dipp}$), 127.03 (s, Ar*C*H$_{metha}$), 127.70 (s, Ar*C*H$_{para}$), 127.90 (s, *C*H$_{dipp}$) 138.04 (s, Ar*C*H$_{ortho}$), 140.25 (s, C^{IV}), 143.78 (s, C^{IV}), 145.37 (s, C^{IV}), 170.94 (s, *C*(CH$_3$)CH*C*(CH$_3$)). Al–C not observed; **HRMS** (EI, +ve) calc. for $[M]^+$ $C_{35}H_{46}N_2FAl$: 540.3460. Found: 540.3477; **Elemental Analysis**: calc. for $C_{35}H_{46}AlFN_2$: C, 77.74; H, 8.57; N, 5.18. Found: C, 77.91; H, 8.51; N, 5.16.

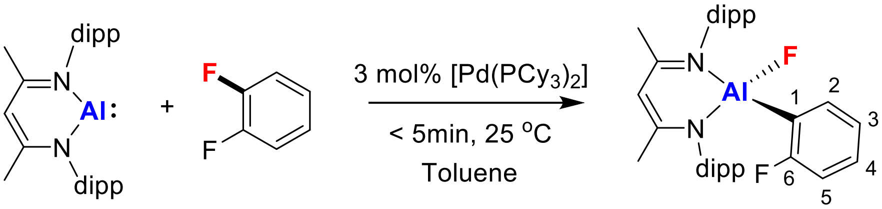

COMPLEX 2b:

Yield: 15 mg, 0.027 mmol (60 %).

^{1}H NMR (400 MHz, C_6D_6): δ 0.55 (d, $^3J_{HH}$ = 6.7 Hz, 6H, CH(CH_3)$_2$), 1.15 (d, $^3J_{HH}$ = 6.8 Hz, 6H, CH(CH_3)$_2$), 1.21 (d, $^3J_{HH}$ = 6.8 Hz, 6H, CH(CH_3)$_2$), 1.58 (d, $^3J_{HH}$ = 6.7 Hz, 6H, CH(CH_3)$_2$), 1.63 (s, 6H, CH_3), 3.24 (sept, $^3J_{HH}$ = 6.8 Hz, 2H, CH(CH$_3$)$_2$), 3.61 (sept, $^3J_{HH}$ = 6.8 Hz, 2H, CH(CH$_3$)$_2$), 5.07 (s, 1H, C(CH$_3$)CHC(CH$_3$)), 6.66 (t, $^3J_{HH}$ = 7.5 Hz, 1H, H$_3$), 6.82 (dd, $^3J_{HH}$ = 7.5 Hz; $^3J_{HF}$ = 7.5 Hz, 1H, H$_5$), 6.95-6.99 (m, 1H, H$_4$), 6.99-7.04 (m, 2H, Ar$_{dipp}$$H$), 7.13-7.21 (m, 4H, Ar$_{dipp}$$H$), 7.31-7.36 (m, 1H, H$_2$). **^{19}F{^{1}H} NMR** (376.5 MHz, C_6D_6): δ -91.81 (s, 1F, F$_6$), -172.01 (bs, 1F, Al–F). **^{13}C{^{1}H} NMR** (100 MHz, C_6D_6): δ 23.31 (s, CH$_3$), 24.05 (s, CH(CH$_3$)$_2$), 24.88 (s, CH(CH$_3$)$_2$), 24.96 (s, CH(CH$_3$)$_2$), 24.98 (s, CH(CH$_3$)$_2$), 28.05 (s, CH(CH$_3$)$_2$), 28.90 (s, CH(CH$_3$)$_2$), 98.29 (s, C(CH$_3$)CHC(CH$_3$)), 113.86 (d, $^2J_{CF}$ = 28.4 Hz, C$_5$), 123.70 (bs, C$_3$), 124.66 (s, CH$_{dipp}$), 124.79 (s, CH$_{dipp}$), 127.81 (s, CH$_{dipp}$), 130.85 (d, $^2J_{CF}$ = 7.8 Hz, C$_4$), 139.18 (C^{IV}), 140.24 (d, $^2J_{CF}$ = 18.1 Hz, C$_2$), 144.68 (s, C^{IV}), 145.09 (s, C^{IV}), 171.15 (s, C(CH$_3$)CHC(CH$_3$)), 171.21 (d, $^1J_{CF}$ = 233.8 Hz, C$_6$). Al–C not observed; **HRMS** (EI, +ve) calc. for [M]$^+$ $C_{35}H_{45}N_2F_2Al$: 558.3366. Found: 558.3354.

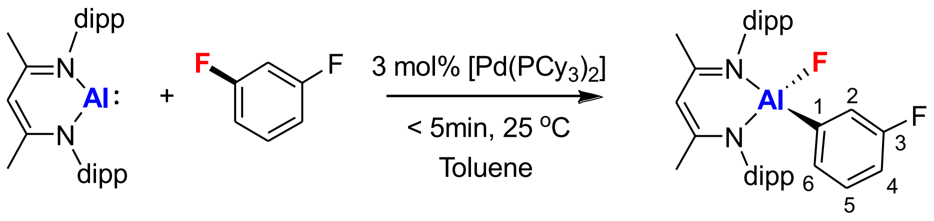

COMPLEX 2c:

Yield: 15 mg, 0.027 mmol (60 %).

^{1}H NMR (400 MHz, C_6D_6): δ 0.84 (d, $^3J_{HH}$ = 6.8 Hz, 6H, CH(C*H*$_3$)$_2$), 1.02 (d, $^3J_{HH}$ = 6.8 Hz, 6H, CH(C*H*$_3$)$_2$), 1.18 (d, $^3J_{HH}$ = 6.8 Hz, 6H, CH(C*H*$_3$)$_2$), 1.42 (d, $^3J_{HH}$ = 6.8 Hz, 6H, CH(C*H*$_3$)$_2$), 1.59 (s, 6H, C*H*$_3$), 3.01 (sept, $^3J_{HH}$ = 6.8 Hz, 2H, C*H*(CH$_3$)$_2$), 3.68 (sept, $^3J_{HH}$ = 6.8 Hz, 2H, C*H*(CH$_3$)$_2$), 5.01 (s, 1H, C(CH$_3$)C*H*C(CH$_3$)), 6.53-6.59 (m, 2H, H_2&H_6), 6.60-6.66 (m, 1H, H_4), 6.68-6.75 (m, 1H, H_5), 7.04-7.08 (m, 2H, Ar$_{dipp}$*H*), 7.14-7.20 (m, 4H, Ar$_{dipp}$*H*); **^{19}F{^{1}H} NMR** (376.5 MHz, C_6D_6): δ -114.84 (s, 1F, C–F), -159.22 (bs, 1F, Al–F); **^{13}C{^{1}H} NMR** (100 MHz, C_6D_6): δ 23.18 (s, *C*H$_3$), 23.62 (s, CH(*C*H$_3$)$_2$), 24.53 (s, CH(*C*H$_3$)$_2$), 24.67 (s, CH(*C*H$_3$)$_2$), 25.65 (s, CH(*C*H$_3$)$_2$), 28.24 (s, *C*H(CH$_3$)$_2$), 29.04 (s, *C*H(CH$_3$)$_2$), 98.25 (s, C(CH$_3$)*C*HC(CH$_3$)), 114.86 (d, $^2J_{CF}$ = 20.7 Hz, C_4), 123.88 (d, $^2J_{CF}$ = 16.3 Hz, C_2), 124.53 (s, *C*H$_{dipp}$), 125.28 (s, *C*H$_{dipp}$), 128.16 (s, *C*H$_{dipp}$), 128.60 (bs, C_5), 133.45 (d, $^2J_{CF}$ = 2.8 Hz, C_6), 139.89 (s, C^{IV}), 143.66 (s, C^{IV}), 145.32 (s, C^{IV}), 162.61 (d, $^1J_{CF}$ = 247 Hz, C_3), 171.16 (s, *C*(CH$_3$)CH*C*(CH$_3$)). Al–C not observed; **HRMS** (EI, +ve) calc. for $[M]^+$ $C_{35}H_{45}N_2F_2Al$: 558.3366. Found: 558.3353; **Elemental Analysis**: calc. for $C_{35}H_{45}AlF_2N_2$: C, 75.24; H, 8.12; N, 5.01. Found: C, 75.19; H, 8.20; N, 4.96.

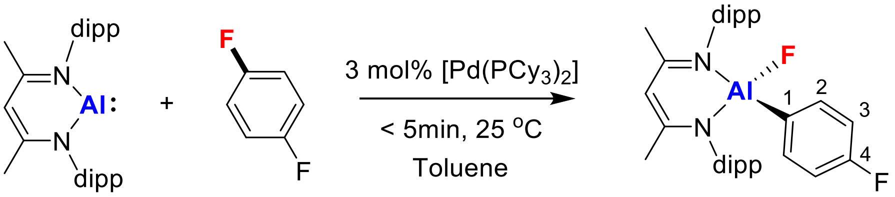

COMPLEX 2d:

Yield: 14 mg, 0.025 mmol (56 %).

^{1}H NMR (400 MHz, C_6D_6): δ 0.77 (d, $^3J_{HH}$ = 6.8 Hz, 6H, CH(C*H*$_3$)$_2$), 1.03 (d, $^3J_{HH}$ = 6.8 Hz, 6H, CH(C*H*$_3$)$_2$), 1.19 (d, $^3J_{HH}$ = 6.8 Hz, 6H, CH(C*H*$_3$)$_2$), 1.43 (d, $^3J_{HH}$ = 6.8 Hz, 6H, CH(C*H*$_3$)$_2$), 1.59 (s, 6H, C*H*$_3$), 2.99 (sept, $^3J_{HH}$ = 6.8 Hz, 2H, C*H*(CH$_3$)$_2$), 3.69 (sept, $^3J_{HH}$ = 6.8 Hz, 2H, C*H*(CH$_3$)$_2$), 5.01 (s, 1H, C(CH$_3$)C*H*C(CH$_3$)), 6.57-6.64 (m, 2H, H_3&$H_{3'}$), 6.71-6.79 (m, 2H, H_2&$H_{2'}$), 7.04-7.08 (m, 2H, Ar$_{dipp}$*H*), 7.14-7.20 (m, 4H, Ar$_{dipp}$*H*). **^{19}F NMR** (376.5 MHz, C_6D_6): -113.03 (s, 1F, F_3), -159.51 (bs, 1F, Al–F). **^{13}C{^{1}H} NMR** (100 MHz, C_6D_6): δ 23.13 (s, CH$_3$), 23.61 (s, CH(*C*H$_3$)$_2$), 24.47 (s, CH(*C*H$_3$)$_2$), 24.59 (s, CH(*C*H$_3$)$_2$), 25.57 (s, CH(*C*H$_3$)$_2$), 28.19 (s, *C*H(CH$_3$)$_2$), 28.90 (s, *C*H(CH$_3$)$_2$), 98.25 (s, C(CH$_3$)*C*HC(CH$_3$)), 114.03 (d, $^2J_{CF}$ = 17.8 Hz, C_3), 124.43 (s, *C*H$_{dipp}$), 125.15 (s, *C*H$_{dipp}$), 128.16 (s, *C*H$_{dipp}$), 139.72 (d, $^3J_{CF}$ = 6.7 Hz, C_2), 139.95 (s, C^{IV}), 143.66 (s, C^{IV}), 145.30 (s, C^{IV}), 163.71 (d, $^1J_{CF}$ = 246 Hz, C_4), 171.16 (s, *C*(CH$_3$)CH*C*(CH$_3$)). Al–C not observed; **HRMS** (EI, +ve) calc. for $[M]^+$ $C_{35}H_{45}N_2F_2Al$: 558.3366. Found: 558.3354; **Elemental Analysis**: calc. for $C_{35}H_{45}AlF_2N_2$: C, 75.24; H, 8.12; N, 5.01. Found: C, 75.16; H, 8.30; N, 4.85.

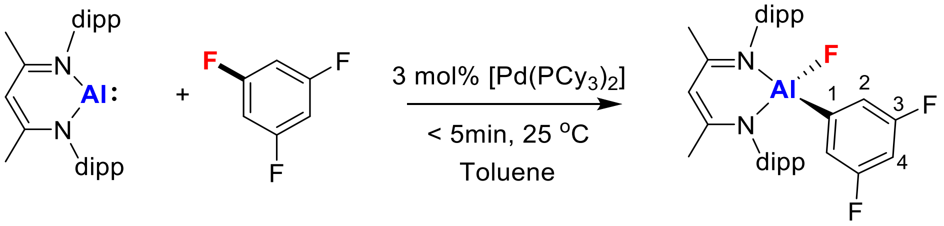

COMPLEX 2e:

Yield: 18 mg, 0.031 mmol (69 %).

^{1}H NMR (400 MHz, C_6D_6): δ 0.87 (d, $^3J_{HH}$ = 6.8 Hz, 6H, CH(CH_3)$_2$), 0.99 (d, $^3J_{HH}$ = 6.8 Hz, 6H, CH(CH_3)$_2$), 1.16 (d, $^3J_{HH}$ = 6.8 Hz, 6H, CH(CH_3)$_2$), 1.41 (d, $^3J_{HH}$ = 6.8 Hz, 6H, CH(CH_3)$_2$), 1.57 (s, 6H, CH_3), 2.97 (sept, $^3J_{HH}$ = 6.8 Hz, 2H, C*H*(CH_3)$_2$), 3.63 (sept, $^3J_{HH}$ = 6.8 Hz, 2H, C*H*(CH_3)$_2$), 4.99 (s, 1H, C(CH_3)C*H*C(CH_3)), 6.30-6.34 (m, 2H, H_2&$H_{2'}$), 6.37 (tt, $^3J_{HF}$ = 9.3 Hz; $^4J_{HH}$ = 2.4 Hz, 1H, H_4), 7.03-7.06 (m, 2H, $Ar_{dipp}H$), 7.13-7.21 (m, 4H, $Ar_{dipp}H$); **^{19}F{^{1}H} NMR** (376.5 MHz, C_6D_6): δ -112.32 (s, 1F, C–F), -159.26 (bs, 1F, Al–F); **^{13}C{^{1}H} NMR** (100 MHz, C_6D_6): δ 23.12 (s, CH_3), 23.50 (s, CH(*C*H_3)$_2$), 24.53 (s, CH(*C*H_3)$_2$), 24.63 (s, CH(*C*H_3)$_2$), 25.60 (d, CH(*C*H_3)$_2$), 28.22 (s, *C*H(CH_3)$_2$), 29.03 (s, *C*H(CH_3)$_2$), 98.41 (s, C(CH_3)*C*HC(CH_3)), 103.41 (t, $^2J_{CF}$ = 25 Hz, C_4), 119.44 (dd, $^2J_{CF}$ = 15 Hz; $^4J_{CF}$ = 4.2 Hz, C_2), 124.60 (s, *C*H_{dipp}), 125.28 (s, *C*H_{dipp}), 128.16 (s, *C*H_{dipp}), 139.55 (s, C^{IV}), 143.52 (s, C^{IV}), 145.29 (s, C^{IV}), 172.37 (s, *C*(CH_3)CH*C*(CH_3)). C_3 is observed *via* HMBC at 162ppm. Al–C not observed; **HRMS** (EI, +ve) calc. for $[M]^+$ $C_{35}H_{44}N_2F_3Al$: 576.3272. Found: 576.3264; **Elemental Analysis**: calc. for $C_{35}H_{44}AlF_3N_2$: C, 72.89; H, 7.69; N, 4.86. Found: C, 72.72; H, 7.76; N, 4.87.

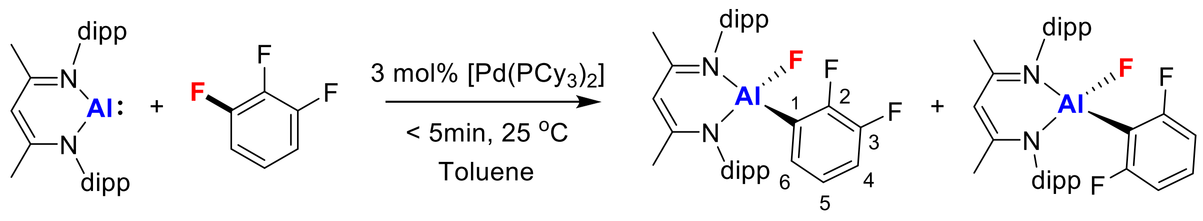

major
2f

minor
2f'

The product was isolated as a mixture of isomers (**2f** 95 % / **2f'** 5 %) based on ^{19}F NMR spectroscopy.

Yield: 17 mg, 0.029 mmol (65 %).

COMPLEX 2f (95 %):

^{1}H NMR (400 MHz, C_6D_6): δ 0.51 (d, $^3J_{HH}$ = 6.8 Hz, 6H, CH(CH_3)$_2$), 1.07 (d, $^3J_{HH}$ = 6.8 Hz, 6H, CH(CH_3)$_2$), 1.15 (d, $^3J_{HH}$ = 6.8 Hz, 6H, CH(CH_3)$_2$), 1.54 (d, $^3J_{HH}$ = 6.8 Hz, 6H, CH(CH_3)$_2$), 1.57 (s, 6H, CH_3), 3.17 (sept, $^3J_{HH}$ = 6.8 Hz, 2H, CH(CH_3)$_2$), 3.52 (sept, $^3J_{HH}$ = 6.8 Hz, 2H, CH(CH_3)$_2$), 5.03 (s, 1H, C(CH_3)CHC(CH_3)), 6.37-6.44 (m, 1H, H_5), 6.79-6.89 (m, 1H, H_4), 6.90-6.97 (m, 2H, Ar$_{dipp}$$H$), 6.98-7.04 (m, 1H, H_6), 7.13-7.21 (m, 4H, Ar$_{dipp}$$H$). **^{19}F{^{1}H} NMR** (376.5 MHz, C_6D_6): δ -119.15 (d, $^3J_{FF}$ = 28.5 Hz, 1F, F_2), -140.83 ($^3J_{FF}$ = 28.5 Hz, 1F, F_3), -172.7 (bs, 1F, Al–F). **^{13}C{^{1}H} NMR** (100 MHz, C_6D_6): δ 23.25 (s, CH_3), 24.04 (s, CH(CH$_3$)$_2$), 24.72 (s, CH(CH$_3$)$_2$), 24.90 (s, CH(CH$_3$)$_2$), 25.06 (s, CH(CH$_3$)$_2$), 27.99 (s, CH(CH_3)$_2$), 28.98 (s, CH(CH_3)$_2$), 98.24 (s, C(CH_3)CHC(CH_3)), 117.76 (d, $^2J_{CF}$ = 17 Hz, C_4), 124.73 (s, CH$_{dipp}$), 124.80 (s, CH$_{dipp}$), 124.84 (bs, C_5), 128.35 (s, CH$_{dipp}$), 134.42 (dd, $^3J_{CF}$ = 16.7 Hz; $^4J_{CF}$ = 4.4 Hz , C_6), 138.73 (s, C^{IV}), 144.71 (s, C^{IV}), 145.01 (s, C^{IV}), 150.50 (dd, $^1J_{CF}$ = 230 Hz; $^2J_{CF}$ = 10 Hz, C_3), 157.40 (dd, $^1J_{CF}$ = 240 Hz; $^2J_{CF}$ = 19 Hz, C_2), 171.43 (s, C(CH_3)CHC(CH_3)). Al–C not observed. **HRMS** (EI, +ve) calc. for $[M]^+$ $C_{35}H_{44}N_2F_3Al$: 576.3272. Found: 576.3263; **Elemental Analysis**: calcd for $C_{35}H_{44}AlF_3N_2$: C, 72.89; H, 7.69; N, 4.86. Found: C, 72.79; H, 7.72; N, 4.83.

COMPLEX 2f' (5 %): ^{19}F{^{1}H} NMR (376.5 MHz, C_6D_6): δ – 89.24 (d, $^4J_{FF}$ = 10.1 Hz, 1F), -168.4 (bs, 1F, Al–F). Partial data only due to low concentration of this minor product.

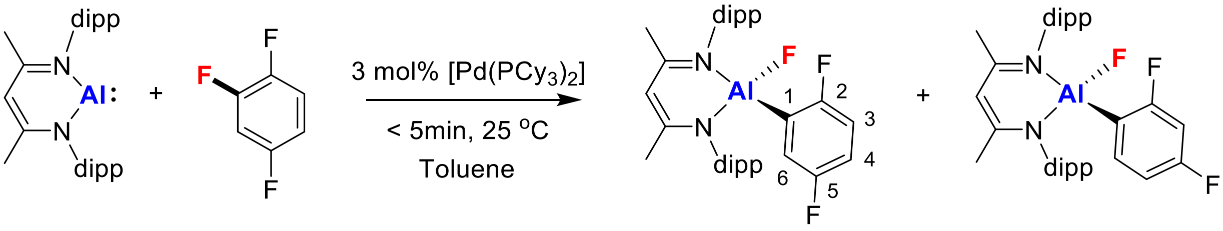

major **2g** minor **2g'**

The product was isolated as a mixture of isomers (**2g** 96 % / **2g'** 4 %) based on ^{19}F NMR spectroscopy.

Yield: 13 mg, 0.022 mmol (50 %).

COMPLEX 2g (96 %):

^{1}H NMR (400 MHz, C_6D_6): δ 0.60 (d, $^3J_{HH}$ = 6.7 Hz, 6H, CH(CH_3)$_2$), 1.11 (d, $^3J_{HH}$ = 6.8 Hz, 6H, CH(CH_3)$_2$), 1.18 (d, $^3J_{HH}$ = 6.8 Hz, 6H, CH(CH_3)$_2$), 1.54 (d, $^3J_{HH}$ = 6.7 Hz, 6H, CH(CH_3)$_2$), 1.59 (s, 6H, CH_3), 3.16 (sept, $^3J_{HH}$ = 6.8 Hz, 2H, CH(CH$_3$)$_2$), 3.56 (sept, $^3J_{HH}$ = 6.8 Hz, 2H, CH(CH$_3$)$_2$), 5.03 (s, 1H, C(CH$_3$)CHC(CH$_3$)), 6.52-6.58 (m, 2H, H$_3$&H$_4$), 6.92-6.97 (m, 1H, H$_6$), 6.98-7.03 (m, 2H, Ar$_{dipp}$$H$), 7.11-7.18 (m, 4H, Ar$_{dipp}$$H$); **^{19}F{^{1}H} NMR** (376.5 MHz, C_6D_6): δ -99.32 (d, $^5J_{FF}$ = 21.6 Hz, 1F, F$_2$), -121.57 (d, $^5J_{FF}$ = 21.6 Hz, 1F, F$_5$), -170.44 (bs, 1F, Al–F); **^{13}C{^{1}H} NMR** (100 MHz, C_6D_6): δ 23.28 (s, CH$_3$), 24.01 (s, CH(CH$_3$)$_2$), 24.88 (s, CH(CH$_3$)$_2$), 24.91 (s, CH(CH$_3$)$_2$), 24.94 (s, CH(CH$_3$)$_2$), 28.10 (s, CH(CH$_3$)$_2$), 28.84 (s, CH(CH$_3$)$_2$), 98.41 (s, C(CH$_3$)CHC(CH$_3$)), 114.88 (dd, $^2J_{CF}$ = 32.7 Hz; 7.1 Hz, C$_3$), 117.01 (dd, $^2J_{CF}$ = 32.7 Hz; 7.1 Hz, C$_4$), 124.69 (s, CH$_{dipp}$), 124.93 (s, CH$_{dipp}$), 125.40 (t, J_{CF} = 19.6 Hz, C$_6$), 127.81 (s, CH$_{dipp}$), 139.02 (s, C^{IV}), 144.37 (s, C^{IV}), 145.10 (s, C^{IV}), 159.38 (d, $^{l}J_{CF}$ = 243.9 Hz, C$_2$), 166.56 (d, $^1J_{CF}$ = 228 Hz, C$_5$), 171.40 (s, C(CH$_3$)CHC(CH$_3$)), Al–C not observed; **HRMS** (EI, +ve) calc. for [M]$^+$ $C_{35}H_{44}N_2F_3Al$: 576.3272. Found: 576.3266; **Elemental Analysis**: calc. for $C_{35}H_{44}AlF_3N_2$: C, 72.89; H, 7.69; N, 4.86. Found: C, 72.66; H, 7.80; N, 4.80.

COMPLEX 2g' (4 %): ^{19}F{^{1}H} NMR (376.5 MHz, C_6D_6): δ -89.62 (d, $^4J_{FF}$ = 9.8 Hz, 1F), -110.37 (d, $^4J_{FF}$ = 9.8 Hz, 1F), -172.06 (bs, 1F, Al–F). Partial data only due to low concentration of this minor product.

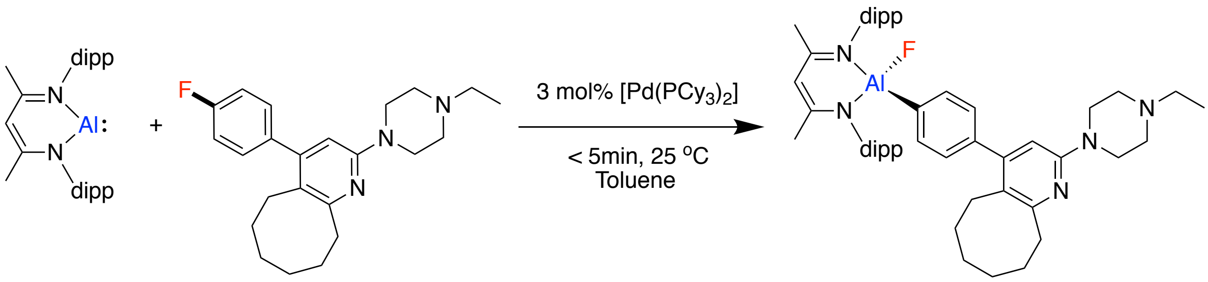

Procedure: In a glovebox, **1** (30.3 mg, 0.068 mmol, 1 equiv.) and $[Pd(PCy_3)_2]$ (204 µL of 0.01 M solution in toluene, 0.00204 mmol, 3 mol%) were weighed into a vial, dissolved in toluene (2 mL) and mixed thoroughly to form a red/orange solution. Blonanserin (25 mg, 0.068 mmol, 1 equiv.) was added and the solution was stirred at room temperature. An immediate colour change was observed from orange to dark green (attributed to the active catalytic species $[Pd(\mathbf{1})_2(PCy_3)]$). After 5 mins ^{1}H NMR indicated the reaction was complete. Over the course of an hour the color faded from green to pale-yellow. The solvent was removed *in-vacuo* and the crude material dissolved in n-hexane (1 mL). A beige solid was isolated by slow evaporation of n-hexane at -35°C. The mother-liquor was decanted from the vial and the product was dried *in-vacuo*.

COMPLEX 2h: The product was isolated as a beige solid. **Yield:** 52.4 mg (95 %)

^{1}H NMR (500 MHz, C_6D_6): δ 7.22 – 7.18 (m, 4H, $Ar_{dipp}H$), 7.11–7.09 (dd, $^3J_{HH}$ = 6.6 Hz, $^3J_{HH}$ = 2.7 Hz, 2H, Ar_{dipp}H), 6.94 – 6.86 (m, 4H), 6.24 (s, 1H, C^{pyr}–H), 5.07 (s, 1H, C–H), 3.71 (sept, $^3J_{HH}$ = 6.7 Hz, 2H, CH(CH$_3$)$_2$), 3.48 (m, 2H, $N^{cyclo}CH_2$), 3.07 (sept, $^3J_{HH}$ = 6.8 Hz, 2H, CH(CH$_3$)$_2$), 2.98 (m, 2H, $CC^{cyclo}H_2$), 2.53 (m, 2H, $CC^{cyclo}H_2$), 2.30 (m, 2H, $N^{cyclo}CH_2$), 2.16 (q, $^3J_{HH}$ = 7.2 Hz, 2H, N-CH_2CH$_3$), 1.88 – 1.82 (m, 2H, $C^{cyclo}H_2$), 1.64 (s, 6H, CCH$_3$), 1.47 (d, $^3J_{HH}$ = 6.6 Hz, 6H, CH(CH_3)$_2$), 1.40 – 1.28 (m, 6H, $C^{cyclo}H_2$), 1.21 (d, $^3J_{HH}$ = 6.9 Hz, 6H, CH(CH_3)$_2$), 1.09 (d, $^3J_{HH}$ = 6.9 Hz, 6H, CH(CH_3)$_2$), 0.95 (overlapping t, $^3J_{HH}$ = 7.2 Hz, 3H, N-CH$_2$CH_3), 0.91 (overlapping d, 6H, CH(CH_3)$_2$) ppm; **^{19}F{^{1}H} NMR** (376.5 MHz, C_6D_6): δ -159.04 (s, Al–F) ppm; **^{13}C{^{1}H} NMR** (100 MHz, C_6D_6): 170.96, 159.43, 157.68, 152.28, 145.39, 143.70, 141.43, 140.18, 137.62, 127.89, 127.19, 125.20, 124.38, 122.39, 106.09 (s, C^{pyr}–H), 98.32 (s, C–H), 53.13, 52.55 (s, NCH$_2$CH$_3$), 45.64, 35.77, 32.06, 31.05, 28.97, 28.20, 26.84, 26.81, 26.32, 25.74, 24.69, 24.46, 23.68, 23.17, 12.44 (s, NCH$_2$CH$_3$). The C–Al resonance could not be observed due to line-broadening associated with coupling to the quadrapolar I = 5/2 ^{27}Al nucleus.

2-2- Al(I) reactivity with fluoroaromatics: Low temperature experiments

In a glovebox **1** (12 mg, 0.017 mmol, 1 equiv.) and [Pd(PCy_3)$_2$] (81.0 µL of 0.01 M solution in toluene, 0.00081 mmol, 3 mol%) were weighed into a vial, dissolved in toluene-d_8 (0.8 mL), mixed thoroughly to form a red/orange solution and transferred into a screw-cap NMR tube. The fluoroarene (either monofluorobenzene or 1,3-difluorobenzene) was loaded into a micro-syringe (5 equiv.), the syringe was closed by blocking the needle with a septum. Outside of the glovebox, the NMR tube was put under positive argon pressure and cooled down to -80 °C (acetone/N_2 coldbath). At this temperature the fluoroarene was added. The tube was kept at -80 °C and immediately introduced into the NMR machine for analysis. The reaction was monitored by ^{1}H NMR spectroscopy from -80 °C to -20 °C. Complex **2a** was formed at -30 °C and complex **2c** at -50 °C (**S.3.29** and **S.3.30**).

2-3- Reaction of [Pd(1)$_2$(PCy_3)]

In a glovebox [Pd(**1**)$_2$(PCy_3)] was introduced into a J Young's tap NMR tube (9 mg, 0.0071 mmol), dissolved in cyclohexane (0.6 mL) and mixed thoroughly to form a black solution. 1,3-difluorobenzene was added by micropipette (1.4 µL, 0.0141 mmol, 2 equiv.) resulting in an immediate color change from black to orange. The acquisition of the first time point (< 5min) by ^{19}F NMR spectroscopy indicates the exclusive C–F bond alumination to form **2c** (**S.3.31**).

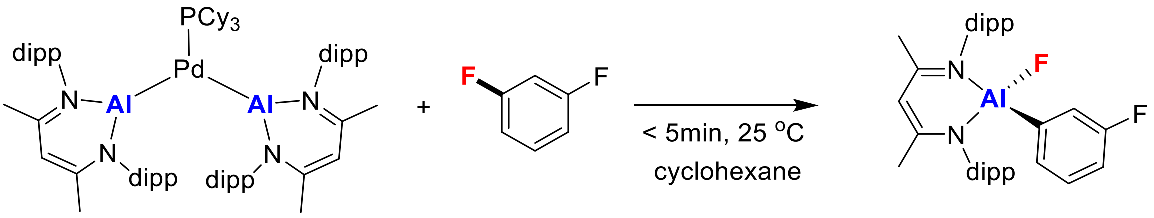

2-4- Kinetics Studies by NMR Spectroscopy

2-4-1- Kinetic Isotope Effect of Palladium Catalyzed C−F Alumination of Benzene *via* the two parallel reactions method

In a glovebox **1** (5 mg, 0.011 mmol, 1 equiv.) and $[Pd(PCy_3)_2]$ (67.4 μL of 0.01 M solution in toluene, 0.00033 mmol, 3 mol%) were weighed into a vial, dissolved in toluene-d_8 (0.8 mL), mixed thoroughly to form a red/orange solution and transferred into a screw-cap NMR tube containing a capillary insert standard (ferrocene in toluene-d_8). The fluoroarene (either C_6H_5F or C_6D_5F) was loaded into a micro-syringe (10.6 μL, 5 equiv.), the syringe was closed by blocking the needle with a septum. Outside of the glovebox, the NMR tube was put under positive argon pressure and cooled down to -80 °C (Acetone/N_2 coldbath). At this temperature the fluoroarene was added. The tube was kept at -80 °C and immediately introduced into the NMR machine for analysis. The sample was warmed to -40 °C at which temperature the first data acquisition was made. While no reaction occurred at -40 °C upon further warming to -20 °C the rate is appreciable, reaction kinetics were measured at this temperature.

A plot of [**1**] (determined from initial concentration and integration against internal standard) *vs* time for both reactions using C_6H_5F or C_6D_5F showed a straight line indicating the reaction is zero order in [**1**] (**Figure S.2.1**). Standard errors were calculated by use of the regression analysis calculation in Microsoft Excel software. The rate constant for the C_6H_5F reaction was found to be $k_{C\text{-}H} = 1.21 \times 10^{-4}$ ($\pm 2.38 \times 10^{-6}$) mol dm^{-3} s^{-1} and $k_{C\text{-}D} = 9.61 \times 10^{-5}$ ($\pm 1.86 \times 10^{-6}$) mol dm^{-3} s^{-1} for C_6D_5F. This gave a **KIE of 1.2** (± 0.1) for the reaction.

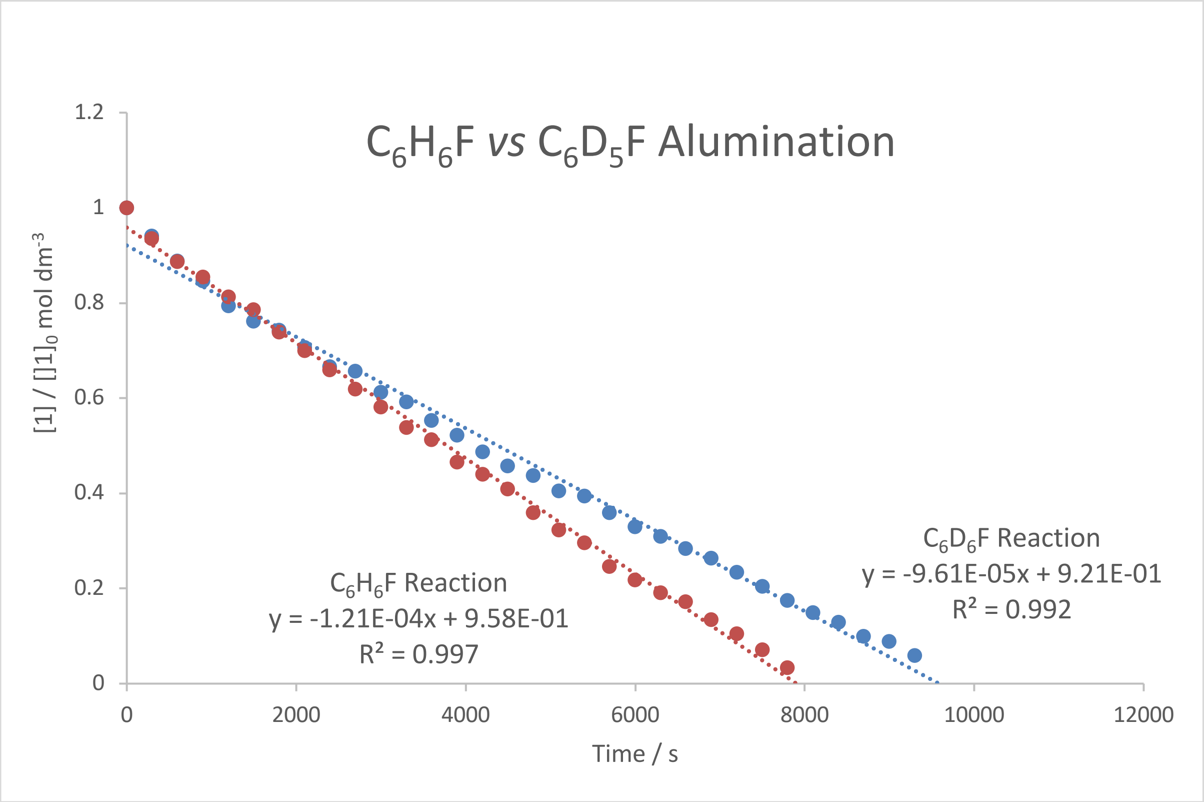

Figure S.2.1: Kinetic study of the C–H vs C–D alumination reaction.

2-4-2- Kinetic Isotope Effect of Palladium Catalyzed C−F Alumination of Benzene *via* the two parallel reactions method

In a glovebox **1** (5 mg, 0.011 mmol, 1 equiv.) and [Pd(PCy$_3$)$_2$] (67.4 µL of 0.01 M solution in substrate, 0.00033 mmol, 3 mol%) were weighed into a vial, dissolved in toluene (0.6 mL), mixed thoroughly to form a red/orange solution and transferred into a vial containing a mixture of C_6H_5F (10.6 µL, 5 equiv.) and C_6D_5F (10.6 µL, 5 equiv.) in solution in toluene (0.2 mL). The solution was stirred at room temperature for 5 min (an immediate color change was observed from orange to yellow) and the solvent was removed under vacuum. The mixture was dissolved in 0.6 mL of C_6D_6 and the ratio of P_H/P_D was measured according to the relative integration by ^{1}H NMR. **KIE = 1.1.**

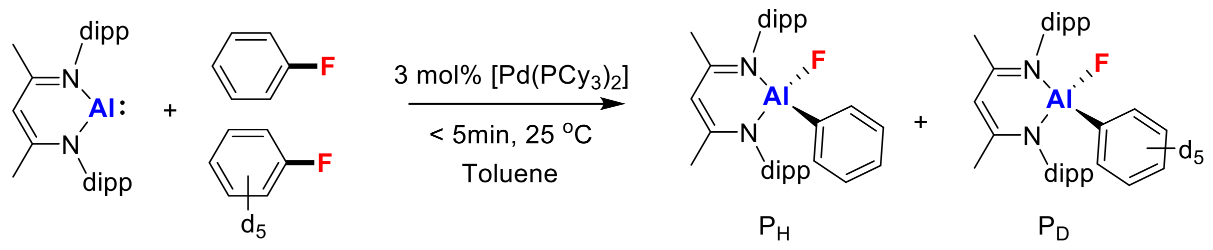

2-5- Catalytic Alumination reaction of 1,3-difluorobenzene

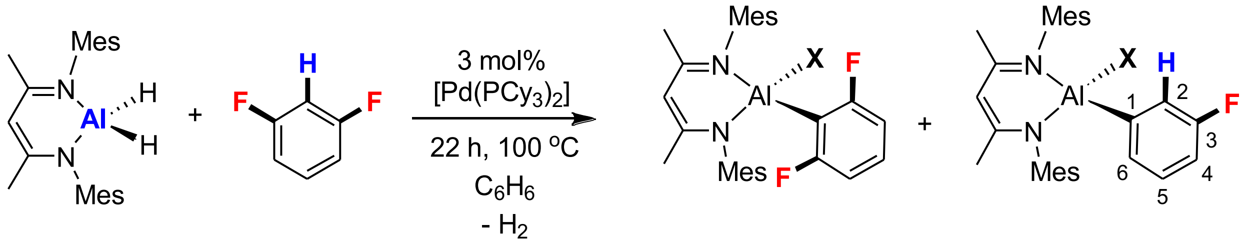

4c (X = H), **4c'** (X = F)
minor
(16%)

5c (X = H), **5c'** (X = F)
major
(67%)

In a glove box, to a 20 mL scintillation vial containing a solution of [Pd(PCy_3)$_2$] (2.01 mg, 0.003 mmol, 0.03 equiv.) in 600 µl C_6H_6, **3** (54.3 mg, 0.15 mmol, 1.5 equiv.) was added, followed by the fluoroarene (0.1 mmol, 1 equiv.). The mixture was transferred to a J Young's tube and removed from the glove box. A ^{19}F NMR spectrum was measured using α,α,α-trifluorotoluene in a capillary as the internal standard. The reaction mixture was then heated at 100 °C for 22 hours. Two new resonances appeared in the ^{19}F NMR spectrum with chemical shifts δ = -90.2 and -115.6 ppm corresponding to complexes **4c'** and **5c'**. All solvent and unconsumed 1,3-difluorobenzene were removed *in vacuo* and the resulting orange residue was dissolved in toluene (2 mL). Several drops of *n*-hexane were added to effect a fractional crystallisation of **5c/5c'** (mixture of aluminium hydride and aluminium fluoride species) with a ratio of 37 % and 63 %, respectively, based on ^{19}F and ^{1}H spectroscopic yields. Similar mixtures of fluoride and hydride compounds have been observed by our group before and we have shown that in this compound series H/F ligand exchange is fast.[1a] Unambiguous support for the formulation of the products was provided by the independent synthesis of **4c** and **5c** (see **section 2-8**). **Yield**: 53 mg, 0.056 mmol (57 %).

COMPLEX 5c' X = F (63 %): ^{1}H NMR (400 MHz, 298K, C_6D_6) δ 1.50 (s, 6H, CH_3), 1.96 (s, 6H, CH_{3Mes}), 2.03 (s, 6H, CH_{3Mes}), 2.47 (s, 6H, CH_{3Mes}), 5.01 (s, 1H, C(CH$_3$)C*H*C(CH$_3$)), 6.63 (bs, 2H, Ar$_{Mes}$*H*), 6.74 (bs, 2H, Ar$_{Mes}$*H*), 6.84 (t, $^{3}J_{HH}$ = 8.2 Hz, 1H, H$_5$), 6.93–7.03 (m, 1H, H$_4$), 7.12 (d, J = 7.1 Hz, 1H, H$_6$), 7.14-7.16 (m, 1H, H$_2$); **^{19}F NMR** (376.5 MHz, 298K, C_6D_6): δ -115.23 – -115.12 (m, 1F), -158.27 (bs, 1H, Al–F).

COMPLEX 5c X = H (37 %): ^{1}H NMR (400 MHz, 298K, C_6D_6) δ 1.50 (s, 6H, CH_3), 1.89 (s, 6H, CH_{3Mes}), 2.03 (s, 6H, CH_{3Mes}), 2.37 (s, 6H, CH_{3Mes}), 4.92 (s, 1H, C(CH$_3$)C*H*C(CH$_3$)), 6.58 (bs, 2H, Ar$_{Mes}$*H*), 6.74 (bs, 2H, Ar$_{Mes}$*H*), 6.84 (t, $^{3}J_{HH}$ = 8.2 Hz, 1H, H$_5$), 6.93–7.03 (m, 1H, H$_4$), 7.39 (d, $^{3}J_{HH}$ = 6.8 Hz, 1H, H$_6$), 7.53 (dd, $^{3}J_{HF}$ = 8.8 Hz; $^{4}J_{HH}$ = 2.8 Hz, 1H, H$_2$); **^{19}F{^{1}H} NMR** (376.5 MHz, 298K, C_6D_6): δ -115.38 – -115.27 (m, 1F).

2-6- Attempted Optimization of Catalytic C–F Alumination

Different catalyst loadings and catalysts were screened to try to increase the yield of desired alumination product **5c/5c'** relative to **4c/4c'**. It can be seen from **Table S.2.1** that both [Pd(OAc)$_2$] and [CpPd(η^3-cinnamyl)] (cinnamyl = 3-phenylprop-2-enyl) can catalyse the alumination reaction, yielding both C–F and C–H alumination products **5c/5c'** and **4c/4c'**; however, the yields of products were lower than the reaction catalysed by [Pd(PCy$_3$)$_2$]. Increasing the catalyst loading of [Pd(PCy$_3$)$_2$] from 1 mol% to 3 mol% led to a higher yield of **5c/5c'** (67%), while further increase of the catalyst loading did not the selectivity (entry 4 and 5). Based on these results, we determined the most efficient catalyst system for C–F alumination of 1,3-difluorobenzene, 3 mol% of [Pd(PCy$_3$)$_2$].

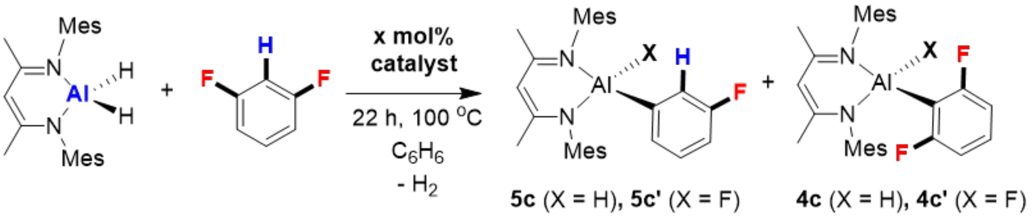

5c (X = H), **5c'** (X = F) **4c** (X = H), **4c'** (X = F)

Entry	catalyst	(mol%)	5c/5c' [a]	4c/4c' [a]
1	[Pd(PCy$_3$)$_2$]	1	43%	10%
2	[Pd(OAc)$_2$]	1	28%	7%
3	[CpPd(cinnamyl)]	1	24%	7%
4	[Pd(PCy$_3$)$_2$]	3	67%	16%
5	[Pd(PCy$_3$)$_2$]	10	68%	14%

[a]Yield measured by ^{19}F NMR spectroscopy using α,α,α-trifluorotoluene as the internal standard.

Table S.2.1: Catalyst Screening of the alumination reaction of 1,3-difluorobenzene.

While reducing the amount of **3** to 1 equiv. led to slightly lower yields, reaction with 2 equiv. of **3** did not show higher yields than the reaction with 1.5 equiv. (**Table S.2.2** entry 1 and 3).

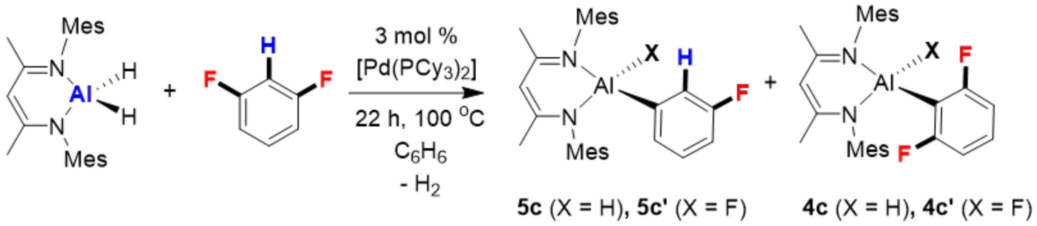

Entry	equiv.	5c/5c' [a]	4c/4c' [a]
1	1.5	67%	13%
2	1	50%	11%
3	2	68%	12%

[a]Yield measured by ^{19}F NMR spectroscopy using α,α,α-trifluorotoluene as the internal standard.

Table S.2.2: Alumination reaction with different amount of **3**.

Solvent effects were also investigated and hydrocarbon solvents provided the highest yields (**Table S.2.3**).

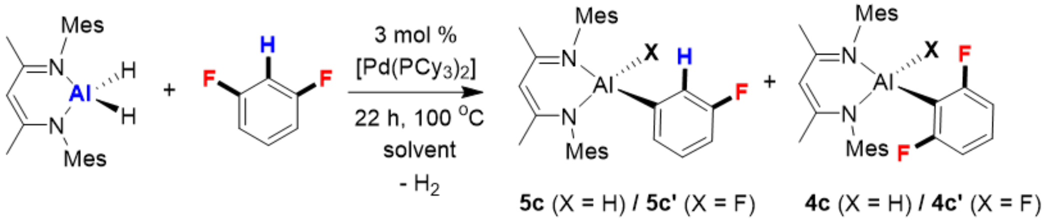

Entry	Solvent	Substrate concentration M	5c/5c' [a]	4c/4c' [a]
1	Toluene	0.167	69%	16%
2	THF	0.167	51%	12%
3	1,4-dioxane	0.167	54%	8%
4	C_6H_6	0.167	70%	16%

[a]Yield measured by ^{19}F NMR spectroscopy using α,α,α-trifluorotoluene as the internal standard.

Table S.2.3: Solvent effect on alumination reaction of 1,3-difluorobenzene.

2-7- Catalytic Alumination of Other Fluoroaromatics

In a glove box, to a 20 mL scintillation vial containing a solution of $[Pd(PCy_3)_2]$ (0.67 mg, 0.001 mmol, 0.01 equiv.) in 600 µl C_6H_6, was added **3** (54.3 mg, 0.15 mmol, 1.5 equiv.) followed by the fluoroarene (0.1 mmol, 1 equiv.). The reactivity of three fluoroarenes was investigated: 1,2-difluorobenzene; 1,2,3-trifluorobenzene and 1,3,5-trifluorobenzene. The mixture was transferred to a J Young's tube and removed from the glove box. The reaction mixture was then heated at 100 °C overnight. A ^{19}F NMR spectrum was measured using α,α,α-trifluorotoluene in a capillary as the internal standard. Both C–H and C–F alumination products are observed for each fluoroarenes. Fractional crystallisation to give pure samples of either product failed due to their similar solubility. Therefore, ^{19}F NMR spectra of the reactions (before protic work-up) are given below with peaks assigned to different alumination products (**S.3.36**, **S.3.37** and **S.3.38**).

The products were assigned by independent synthesis (see **Section 2-8** for complexes **4f** and **4e**) or by comparison with data in **Section 2-1** for the C–F alumination compounds (complexes **2b**, **2e** and **2f**).

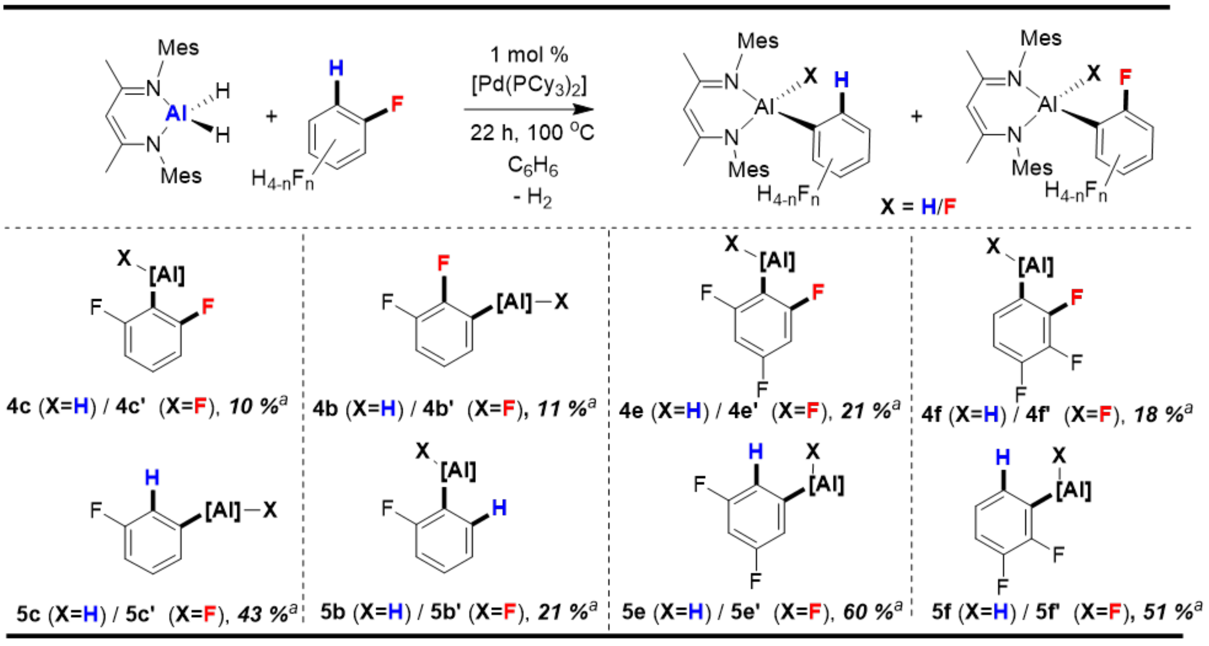

[a]Spectroscopic yield measured by ^{19}F NMR spectroscopy using α,α,α-trifluorotoluene as the internal standard.

Figure S.2.2: Catalytic alumination of 1,3-difluorobenzene 1,2-difluorobenzene; 1,3,5-trifluorobenzene and 1,2,3-trifluorobenzene.

2-8- General Procedure for Non-Catalytic Synthesis of [{(MesNCMe)$_2$CH}AlHAr^F]

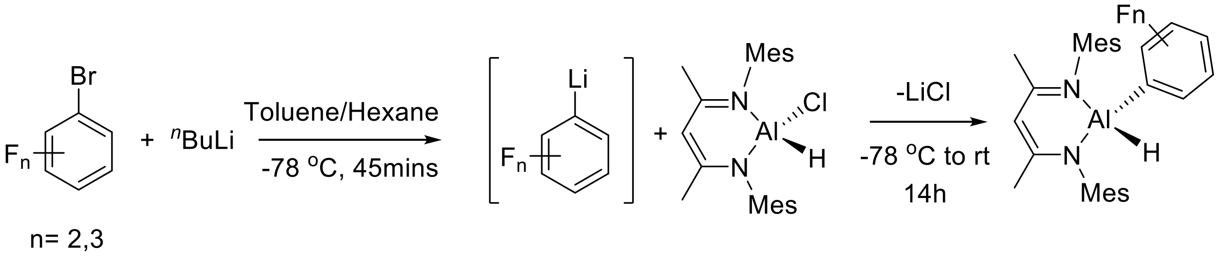

A solution of ^{n}BuLi (0.31 mL, 1.6M in hexanes, 1.0 equiv.) was added dropwise to a solution of bromofluorobenzene (0.5 mmol, 1.0 equiv.) in n-hexane (5 mL) at -78 °C. The resulting mixture was stirred at this temperature for 45min. To this mixture a solution of **S1** (198 mg, 0.5 mmol) in toluene (8 mL) was added dropwise at -78 °C. The reaction mixture was stirred for 2 h at -78 °C and then allowed to warm slowly to 25 °C and stirred for a further 18 h. The solvent was removed *in vacuo* and the crude product was extracted into toluene. After filtration *via* a cannula, the filtrate was dried *in vacuo* to give a yellowish oily product. In a glovebox, the yellowish oily product was washed with n-hexane resulting in the formation of colourless solid which was dried *in vacuo*. The products were crystallised from toluene/*n*-hexane (2 mL/1 mL) at –35 °C in the glovebox freezer.

COMPLEX 5c:

Yield: 21 mg, 0.048 mmol, (<10 %).

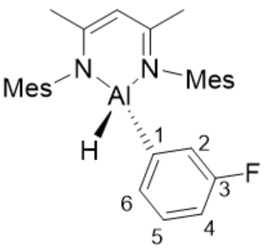

^{1}H NMR (400 MHz, 298K, C_6D_6) δ 1.50 (s, 6H, C*H$_3$*), 1.89 (s, 6H, C*H$_3$*$_{Mes}$), 2.03 (s, 6H, C*H$_3$*$_{Mes}$), 2.37 (s, 6H, C*H$_3$*$_{Mes}$), 4.92 (s, 1H, C(CH$_3$)C*H*C(CH$_3$)), 6.58 (bs, 2H, Ar$_{Mes}$*H*), 6.74 (bs, 2H, Ar$_{Mes}$*H*), 6.84 (t, $^3J_{HH}$ = 8.2 Hz, 1H, H$_5$), 6.93–7.03 (m, 1H, H$_4$), 7.39 (d, $^3J_{HH}$ = 6.8 Hz, 1H, H$_6$), 7.53 (dd, $^3J_{HF}$ = 8.8 Hz; $^4J_{HH}$ = 2.8 Hz, 1H, H$_2$); **^{19}F{^{1}H} NMR** (376.5 MHz, 298K, C_6D_6): δ -115.38 – -115.27 (m, 1F).

COMPLEX 4c:

Yield: 58 mg, 0.12 mmol (25 %).

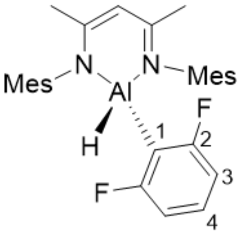

^{1}H NMR (400 MHz, C_6D_6): δ 1.54 (s, 6H, C*H_3*), 1.97 (s, 6H, C*H_{3}*$_{Mes}$), 2.03 (s, 6H, C*H_{3}*$_{Mes}$), 2.37 (s, 6H, C*H_{3}*$_{Mes}$), 5.09 (s, 1H, C(CH_3)C*H*C(CH_3)), 6.57 (s, m, 2H, Ar_{Mes}*H*), 6.60 (dd, *J* = 8.1 Hz; 6.2 Hz, 2H, H_3&$H_{3'}$), 6.74 (s, m, 2H, Ar_{Mes}*H*), 6.79-6.86 (m, 1H, H_4); **^{19}F{^{1}H} NMR** (376.5 MHz, C_6D_6): δ -89.80 (s); **^{13}C{^{1}H} NMR** (100 MHz, C_6D_6): 17.77 (s, *C*H_3), 19.11 (s, *C*H_{3Mes}), 20.84 (s, *C*H_{3Mes}), 22.64 (s, *C*H_{3Mes}), 97.99 (s, C(CH_3)*C*HC(CH_3)), 110.35 (d, $^2J_{CF}$ = 32.0 Hz, C_3), 130.0 (s, *C*H_{Mes}), 130.13 (s, *C*H_{Mes}), 131.62 (t, $^3J_{CF}$ = 9.4 Hz, C_4), 133.26 (s, C^{IV}), 133.88 (s, C^{IV}), 135.71 (s, C^{IV}), 140.08 (s, C^{IV}), 170.19 (s, *C*(CH_3)CH*C*(CH_3)), 170.58 (dd, $^1J_{CF}$ = 238.0 Hz; $^3J_{CF}$ = 21.0 Hz, C_2).

COMPLEX 4e:

Yield: 40 mg, 0.081 mmol (16 %).

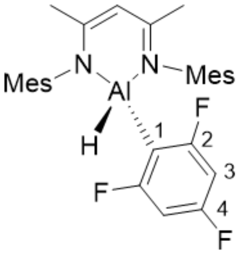

^{1}H NMR (400 MHz, C_6D_6): δ 1.52 (s, 6H, C*H_3*), 1.94 (s, 6H, C*H_{3}*$_{Mes}$), 2.04 (s, 6H, C*H_{3}*$_{Mes}$), 2.34 (s, 6H, C*H_{3}*$_{Mes}$), 5.06 (s, 1H, C(CH_3)C*H*C(CH_3)), 6.32 (dd, *J* = 9.3 Hz; 5.6 Hz, 2H, H_3&$H_{3'}$), 6.60 (s, m, 2H, Ar_{Mes}*H*), 6.74 (s, m, 2H, Ar_{Mes}*H*); **^{19}F NMR** (376.5 MHz, C_6D_6): δ -87.37 – -87.57 (m, 2F, F_2), -108.92 – -109.17 (m, 1F, F_4); **^{13}C{^{1}H} NMR** (100 MHz, C_6D_6): 17.60 (s, *C*H_3), 19.00 (s, *C*H_{3Mes}), 20.80 (s, *C*H_{3Mes}), 22.50 (s, *C*H_{3Mes}), 97.90 (s, C(CH_3)*C*HC(CH_3)), 130.0 (s, *C*H_{Mes}), 130.20 (s, *C*H_{Mes}), 133.70 (s, C^{IV}), 135.80 (s, C^{IV}), 149.90 (s, C^{IV}), 164.9 (dt, $^1J_{CF}$ = 246.3 Hz; $^3J_{CF}$ = 15.2 Hz, C_4), 170.30 (s, *C*(CH_3)CH*C*(CH_3)), 170.60 (ddd, $^1J_{CF}$ = 238.2 Hz; $^3J_{CF}$ = 23.6 Hz; $^3J_{CF}$ = 14.2 Hz, C_2); **Elemental analysis**: calc. for $C_{29}H_{32}AlF_3N_2$ – C 70.72%, H 6.55%, N 5.69%; found – C 70.78%, H 6.63%, N 5.70%; **Infrared** (solid, cm^{-1}): 2956, 2918, 2855, 1867, 1610, 1528, 1454, 1383.

COMPLEX 4f:

Yield: 19 mg, 0.038 mmol (<10 %).

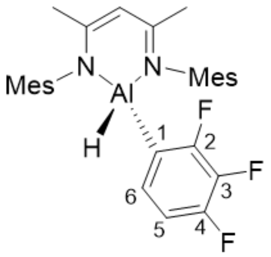

^{1}H NMR (400 MHz, C_6D_6): δ 1.50 (s, 6H, CH_3), 1.88 (s, 6H, CH_{3Mes}), 2.02 (s, 6H, CH_{3Mes}), 2.34 (s, 6H, CH_{3Mes}), 5.04 (s, 1H, C(CH$_3$)CHC(CH$_3$)), 6.44-6.33 (m, H_5 or H_6), 6.57 (s, 2H, Ar$_{Mes}$$H$), 6.74 (s, 2H, Ar$_{Mes}$$H$), 6.94-6.85 (m, H_5 or H_6); **^{19}F NMR** (377 MHz, C_6D_6): δ -114.54 – -114.77 (m, 1F), -136.31 – -136.52 (m, 1F), -164.73 – -165.00 (m, 1F). **^{13}C{^{1}H} NMR** (100 MHz, C_6D_6): 17.51 (s, CH$_{3Mes}$), 18.94 (s, CH$_{3Mes}$), 20.72 (s, CH$_{3Mes}$), 22.46 (s, CH$_3$), 97.78 (s, C(CH$_3$)CHC(CH$_3$)), 112.60 (d, $^2J_{CF}$ = 14.88 Hz, C_5), 129.82 (s, CH$_{Mes}$), 130.03 (s, CH$_{Mes}$), 132.64 (dt, $^3J_{CF}$ = 6.9 Hz, $^3J_{CF}$ = 6.9 Hz, $^3J_{CF}$ = 20.48 Hz, C_6), 133.03 (s, C^{IV}), 133.94 (s, C^{IV}), 135.86 (s, C^{IV}), 139.75 (s, C^{IV}), 139.86 (m, C_3), 152.43 (m, C_4), 157.15 (m, C_2), 170.32 (s, C(CH$_3$)CHC(CH$_3$)).

2-9- Isomerisation process with 4c

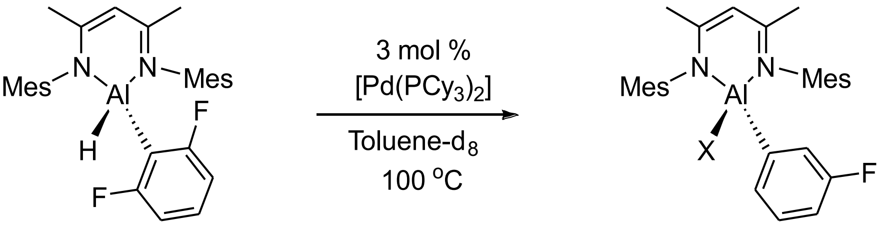

5c (X = H) / **5c'** (X = F)

In a glovebox, **4c** (11 mg, 0.0232 mmol, 1 equiv.) was weighed and transferred to a scintillation vial, dissolved in toluene-d_8 and transferred to a J Young's tube containing a capillary insert standard (α,α,α-trifluorotoluene in toluene-d_8). A solution of $[Pd(PCy_3)_2]$ (68.7 μL of 0.01 M solution in substrate, 0.00069 mmol, 3 mol%) in toluene was added to the NMR tube and removed from the glovebox. The mixture was heated at 100 °C and monitored overtime. After 48h, conversion to **5c/5c'** (66 % as a mixture of Al–H and Al–F complexes) was observed. The remaining 34 % is a mixture of **4c/4c' (**hydride and fluoride analogues) (**S.3.50** and **S3.51**).

2-10- Trapping experiments with 4c

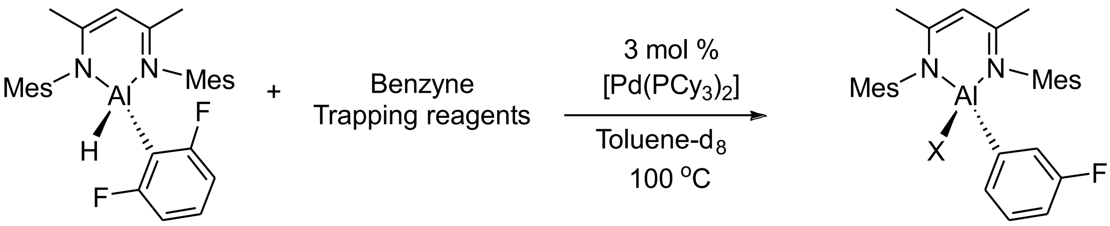

5c (X = H) / **5c'** (X = F)

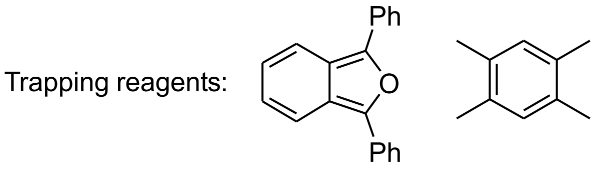

In a glovebox, **4c** (10 mg, 0.0210 mmol, 1 equiv.) was weighed into a scintillation vial and dissolved in toluene-d_8 (0.4 mL). To this solution was added the trapping reagent (either 1,3-diphenylisobenzofuran or durene) (0.210 mmol, 10 equiv) in toluene-d_8 (0.4 mL). The solution was transferred to a J Young's tube and a solution of $[Pd(PCy_3)_2]$ (62.4 μL of 0.01 M solution in substrate, 0.00069 mmol, 3 mol%) in toluene was added to the NMR tube and removed from the glovebox. The mixture was heated at 100 °C and monitored overtime. After 48h of reaction conversion of **4c** to **5c/5c'** was observed. No organic fragment resulting from the trapping of fluorobenzyne intermediates was detected.

2-11- Cross over experiment with 4c

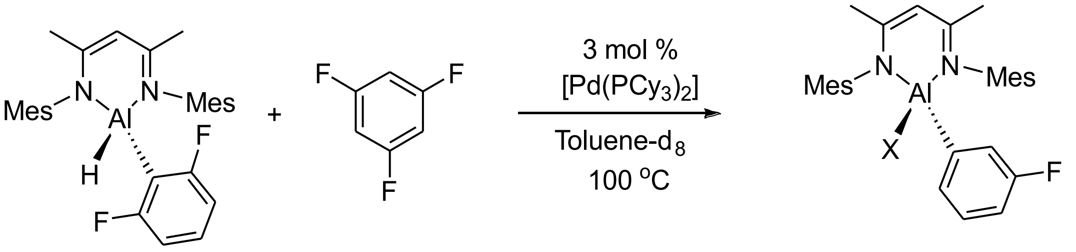

5c (X = H) / **5c'** (X = F)

In a glovebox, **4c** (10 mg, 0.0210 mmol, 1 equiv.) was weighed into a scintillation vial and dissolved in toluene-d_8 (0.8 mL) followed by 1,3,5-trifluorobenzene (10.8 mL, 0.105 mmol, 5 equiv.). The solution was transferred to a J Young's tube and a solution of [Pd(PCy_3)$_2$] (62.4 µL of 0.01 M solution in substrate, 0.00069 mmol, 3 mol%) in toluene was added to the NMR tube which was then removed from the glovebox. The mixture was heated at 100 °C and monitored over time. After 48h of reaction conversion of **4c** to **5c/5c'** was observed. No cross-over products were observed.

2-12- Cross Coupling reactions

2-12-1- Pd-catalysed cross-coupling reaction

General procedure: In a glovebox, **3** (54.3 mg, 0.15 mmol, 1.5 equiv.) was added to a scintillation vial containing a solution of [Pd(PCy_3)$_2$] (2.0 mg, 0.003 mmol, 3 mol%) in 600 µl C_6H_6, followed by fluoroarenes (0.1 mmol, 1 equiv.). The mixture was transferred to a J Young's tube and removed from the glovebox. The reaction mixture was heated at 100 °C overnight. All solvent and unconsumed fluoroarene were removed *in vacuo*. A solution of [Pd(PCy_3)$_2$] (2.0 mg, 0.003 mmol, 0.03 equiv.) in 1000 µl of toluene was added to the orange residue, which was then transferred to a microwave reaction vial. After the addition of salt (0-4 equiv.) and bromobenzene (47.1 mg, 0.3 mmol, 3 equiv.), the vial was sealed and removed from glovebox. The mixture was heated at 120 °C for 5 days before quenching with methanol. 15 mL distilled water and 15 mL ethyl acetate were added. The organic layer was separated and the aqueous layer was extracted with ethyl acetate (3×15 mL). The combined organic layers were dried over $MgSO_4$ and concentrated *in vacuo*. The residue was analysed by HPLC using following method. Three products were detected by HPLC analysis.

Solvent A: Water Solvent B: Acetonitrile *λ = 254 nm, T = 40°C, Stop time = 12 min*

Time / min	A %	B %	Flow mL/min	Pressure bar
**11.00**	56.0	44.0	1.500	400.00

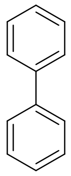

1,1'-Biphenyl, with retention time = 7.47 min. The detected solution was spiked with pure biphenyl and 2,6-difluorobiphenyl (rt = 7.375 min) to confirm. **GCMS**: m/z (EI, +ve) 154.

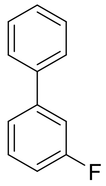

3-fluoro-1,1'-biphenyl, with retention time = 8.65 min. The detected solution was spiked with pure 3-fluoro-1,1'-biphenyl and 2-fluoro-1,1'-biphenyl (rt = 7.70 min) to confirm. **GCMS**: m/z (EI, +ve) 172. **^{19}F NMR** (377 MHz, 298K, C_6D_6) δ -112.98 ppm.[24] NMR data do not match the known isomer 2-fluoro-1,1'-biphenyl.[25]

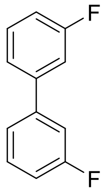

3,3'-difluoro-biphenyl, with retention time = 9.96 min. The detected solution was spiked with pure 3,3'-difluoro-biphenyl to confirm. **GCMS**: m/z (EI, +ve) 190. **^{19}F NMR** (377 MHz, 298K, C_6D_6) δ -112.7 ppm.[24] NMR data do not match the known isomer 2,6-difluorobiphenyl.[26]

In order to calculate the yields of these three products, we used calibration curves to quantify the relationship between the integration values of peaks on the HPLC chromatogram and concentrations. For example, a calibration curve of 3,3'-difluoro-biphenyl was obtained from six known concentrations solutions of 3,3'-difluoro-biphenyl. From the concentration values we can calculate the amount of product and therefore the yield.

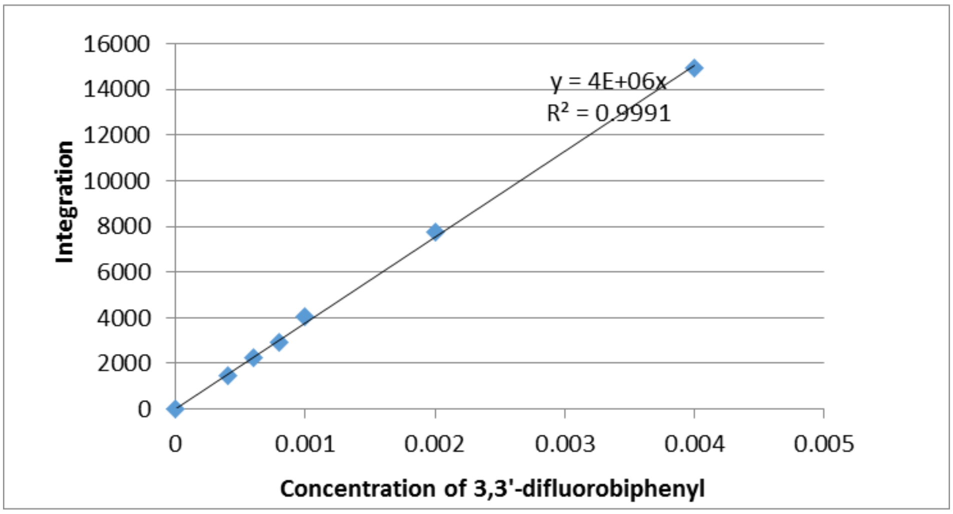

Figure S.2.3: The calibration curve of 3,3'-difluoro-biphenyl on HPLC chromatogram.

The result of cross-coupling reactions using different salts and solvents is presented.

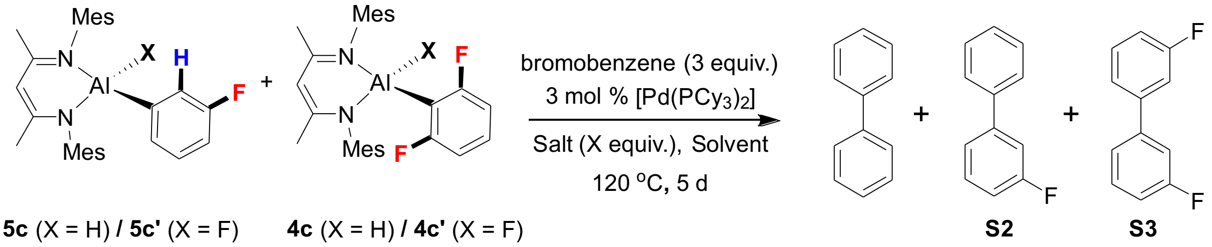

Entry	Salt	equiv.	solvent	biphenyl[a]	S2[a]	S3[a]
**1**	LiCl	0	toluene	3%	13%	3%
**2**	LiCl	2	toluene	3%	17%	9%
**3**	LiCl	4	toluene	2%	15%	8%
**4**	Bu_4NCl	2	toluene	8%	14%	trace
**5**	LiF	2	toluene	1%	16%	4%
**6**	LiBr	2	toluene	2%	13%	5%
**7**	LiI	2	toluene	--	--	--
**8**[b]	LiCl	2	THF	2%	8%	4%
**9**	LiCl	2	NMP	7%	9%	--
**10**	LiCl	2	DMF	1%	1%	--
**11**[c]	LiCl	2	THF/NMP	8%	14%	--

[a]Overall yield for 2-steps reaction (alumination and cross-coupling reaction), based on 0.1 mmol of 1,3-difluorobenzene. Reactions conducted in 1mL solvent in microwave reaction vials with stirring. Yields were determined by HPLC analysis. [b]Reaction conducted in 1mL THF in an ampoule with stirring.

Table S.2.4: Cross coupling reaction varying the nature and the amount of salts and using different solvents.

2-12-2- Ni-catalysed cross-coupling reaction

General procedure: In a glovebox, **3** (54.3 mg, 0.15 mmol, 1.5 equiv.) was added to a scintillation vial containing a solution of [Pd(PCy$_3$)$_2$] (2.0 mg, 0.003 mmol, 3 mol%) in 600 µl C_6H_6, followed by fluoroarenes (0.1 mmol, 1 equiv.). The mixture was transferred to a J Young's tube and removed from the glovebox. The reaction mixture was heated at 100 °C overnight. All solvent and unconsumed fluoroarene were removed *in vacuo*. A solution of [Ni(acac)$_2$] (0.7 mg, 0.003 mmol, 3 mol%) and L2 (1.6 mg, 0.003 mmol, 3 mol%) in 1000 µl

of toluene was added to the orange residue which was then transferred to a microwave reaction vial. After the addition of salt (0-4 equiv.) and bromobenzene (47.1 mg, 0.3 mmol, 3 equiv.), the vial was sealed and removed from glovebox. The mixture was heated at 120 °C for 5 days before quenching with methanol. 15 mL distilled water and 15 mL ethyl acetate were added. The organic layer was separated and the aqueous layer was extracted with ethyl acetate (3×15 mL). The combined organic layers were dried over $MgSO_4$ and concentrated *in vacuo*. The residue was analysed by HPLC using same method as previously. The same three products as previously were detected by HPLC analysis.

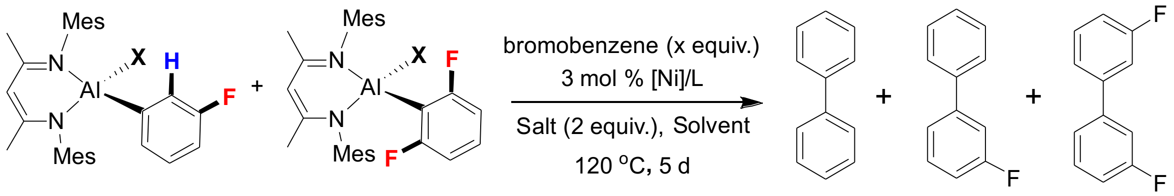

5c (X = H) / **5c'** (X = F) **4c** (X = H) / **4c'** (X = F) **S2** **S3**

Entry	Catalyst	Ligand	PhBr (equiv.)	Salt	solvent	biphenyl[a]	S2[a]	S3[a]
1	$Ni(cod)_2$	PCy_3	3	LiCl	toluene	7%	14%	5%
2	$Ni(acac)_2$	L1	3	LiCl	toluene	14%	34%	2%
3	$Ni(acac)_2$	L2	3	LiCl	toluene	4%	34%	4%
4	$Ni(acac)_2$	L2	3	--	toluene	4%	33%	4%
5	$Ni(acac)_2$	L2	6	--	toluene	4%	36%	4%
6	$Ni(acac)_2$	L2	6	--	THF	7%	18%	4%
7	$Ni(acac)_2$	L2	6	--	*o*-xylene	5%	32%	3%
8	$Ni(acac)_2$	--	6	--	toluene	4%	6%	traces
9	--	L2	6	--	toluene	3%	15%	4%
10[b]	$Ni(acac)_2$	L2	6	--	toluene	6%	39%	3%
11[c]	$Ni(acac)_2$	L2	6	--	toluene	8%	28%	2%

[a]Overall yield for 2-steps reaction (alumination and cross-coupling reaction), based on 0.1 mmol of 1,3-difluorobenzene. Reactions conducted in 1mL solvent in microwave reaction vials with stirring. Yields were determined by HPLC analysis. [b]Reaction using 5 mol% of catalyst/L2. [c]Reaction using 10 mol% of catalyst/L2.

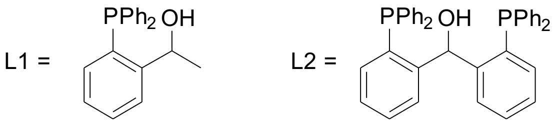

Table S.2.5: Cross-coupling reaction using different Ni catalysts, solvents and varying the stoichiometry of electrophiles.

3- NMR spectra of complexes

Figure S.3.1: ^{1}H NMR spectrum of complex **2a** (* residual toluene and hexane)

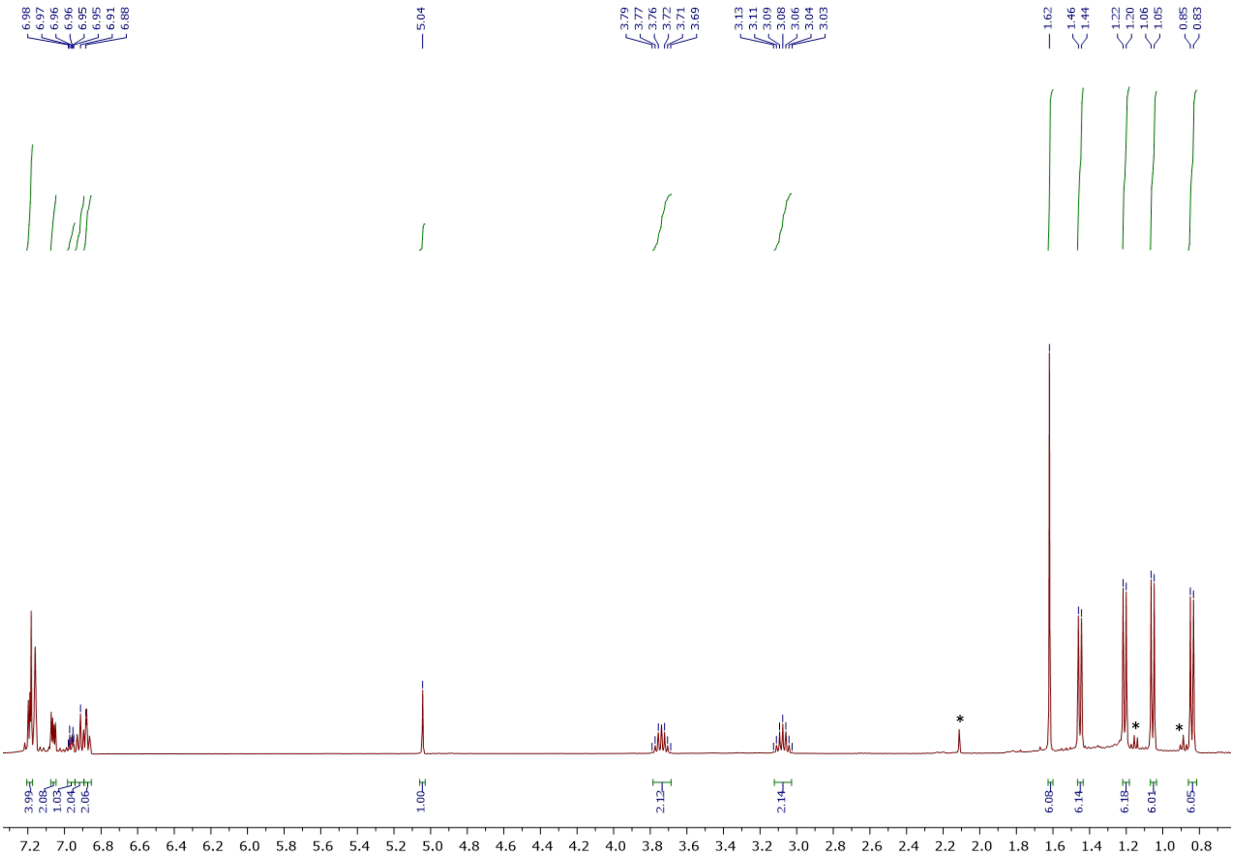

Figure S.3.2: $^{19}F\{^{1}H\}$ NMR spectrum of complex **2a**

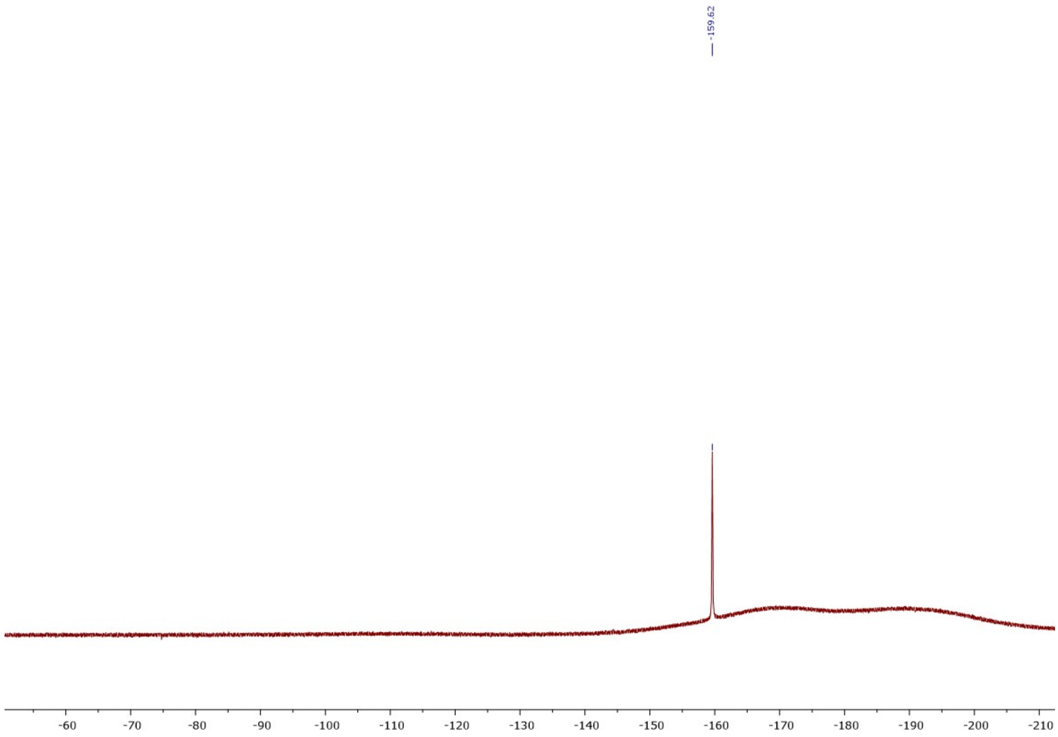

Figure S.3.3: $^{13}C\{^{1}H\}$ NMR spectrum of complex **2a**

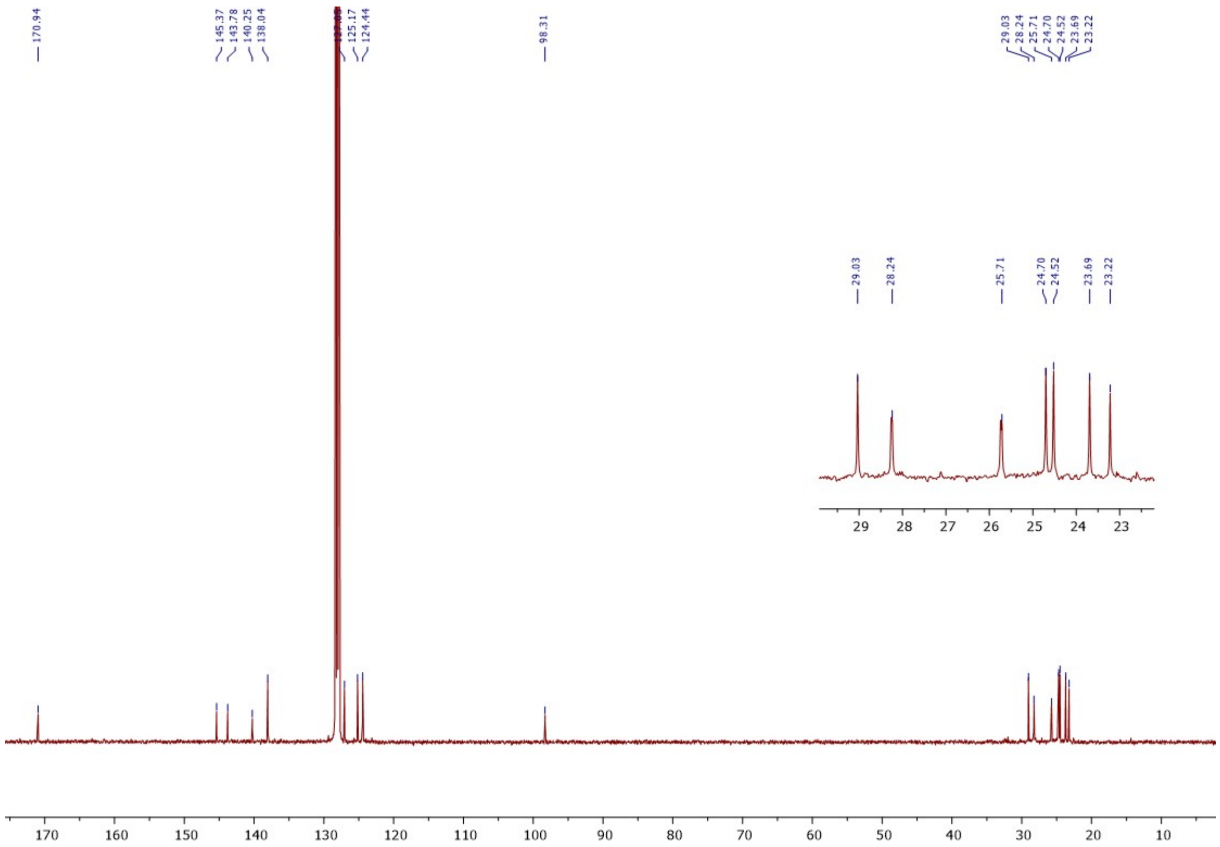

Figure S.3.4: ^{1}H NMR spectrum of complex **2b** (* residual toluene and hexane)

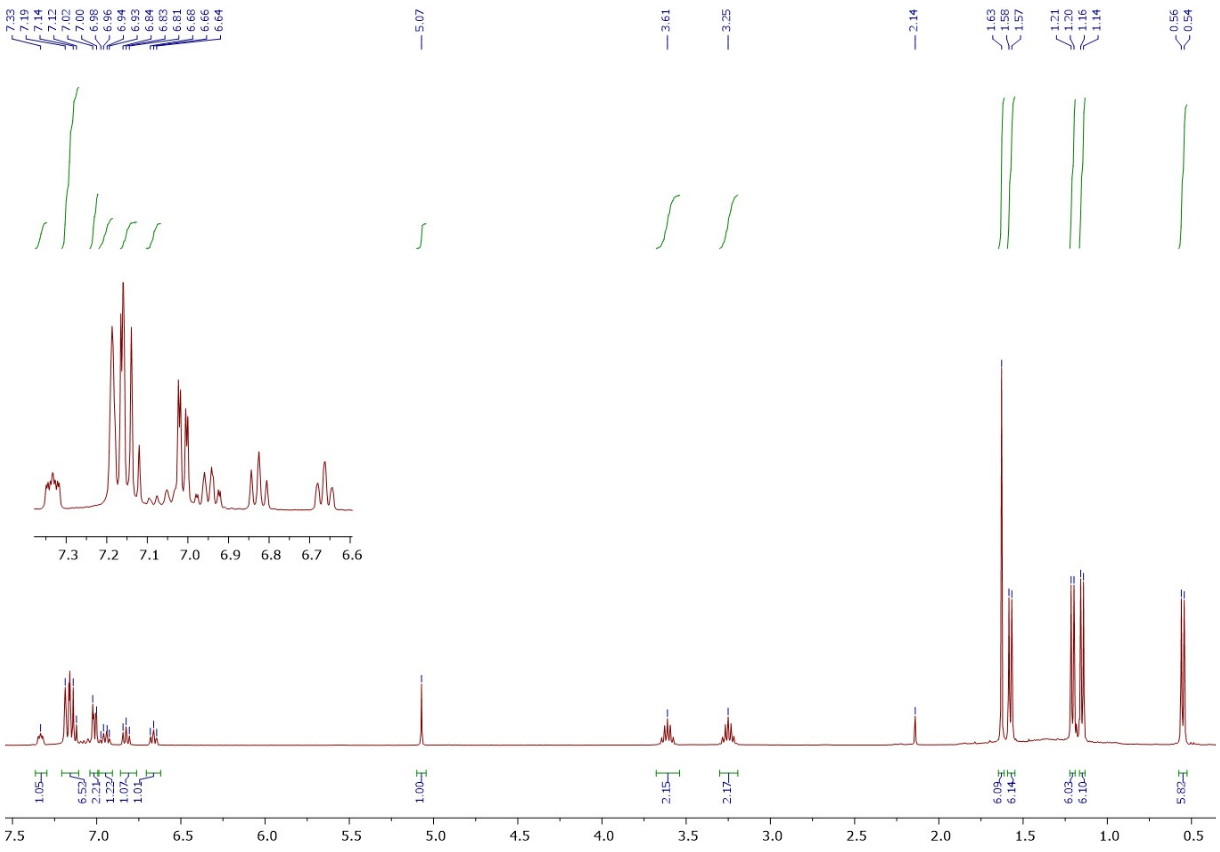

Figure S.3.5: ^{19}F{^{1}H} NMR spectrum of complex **2b**

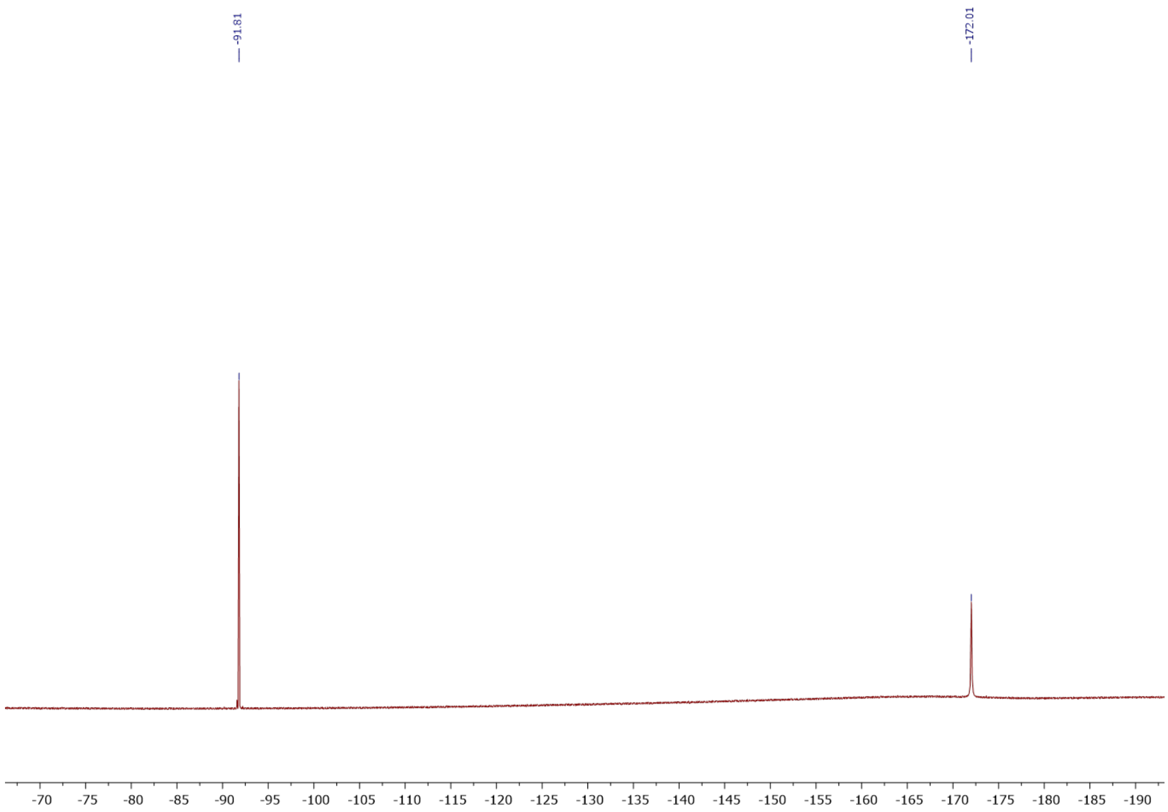

Figure S.3.6: $^{13}C\{^1H\}$ NMR spectrum of complex **2b**

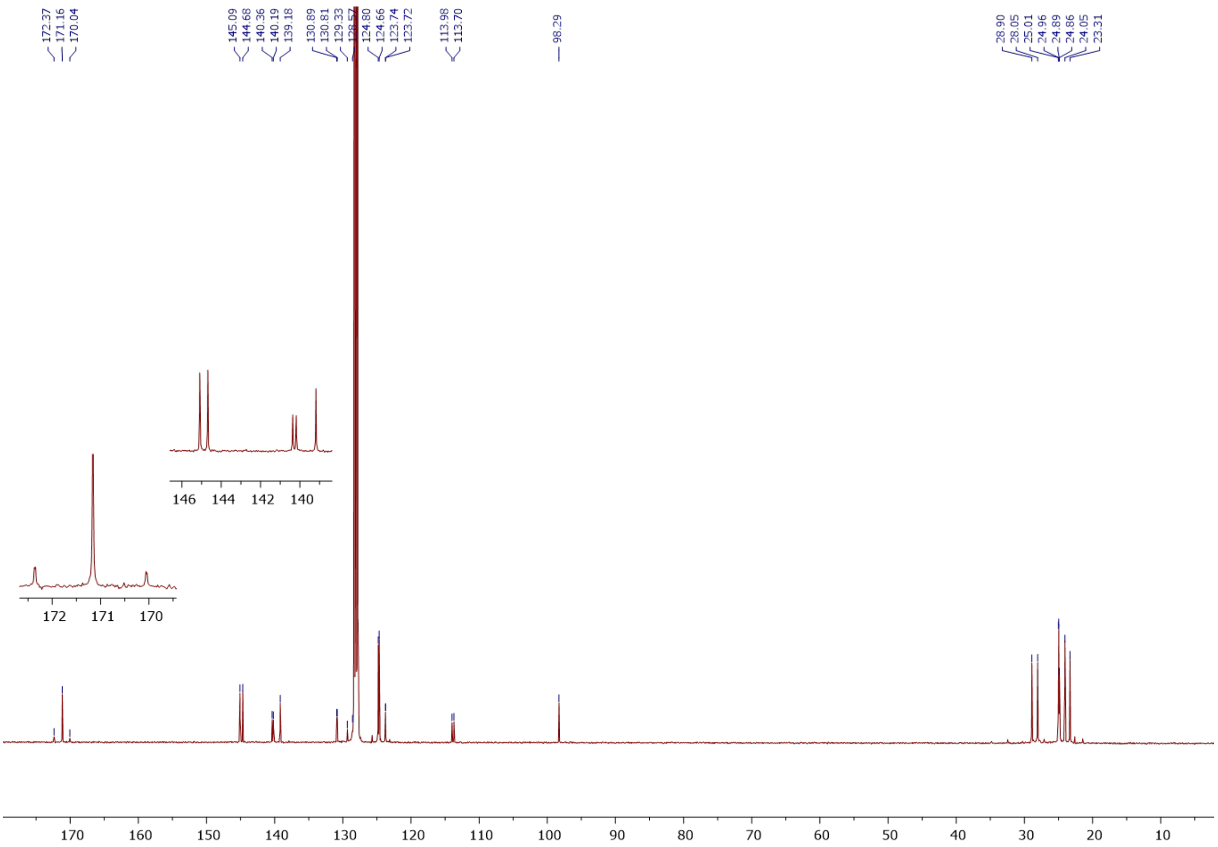

Figure S.3.7: ^{1}H NMR spectrum of complex **2c** (* residual toluene)

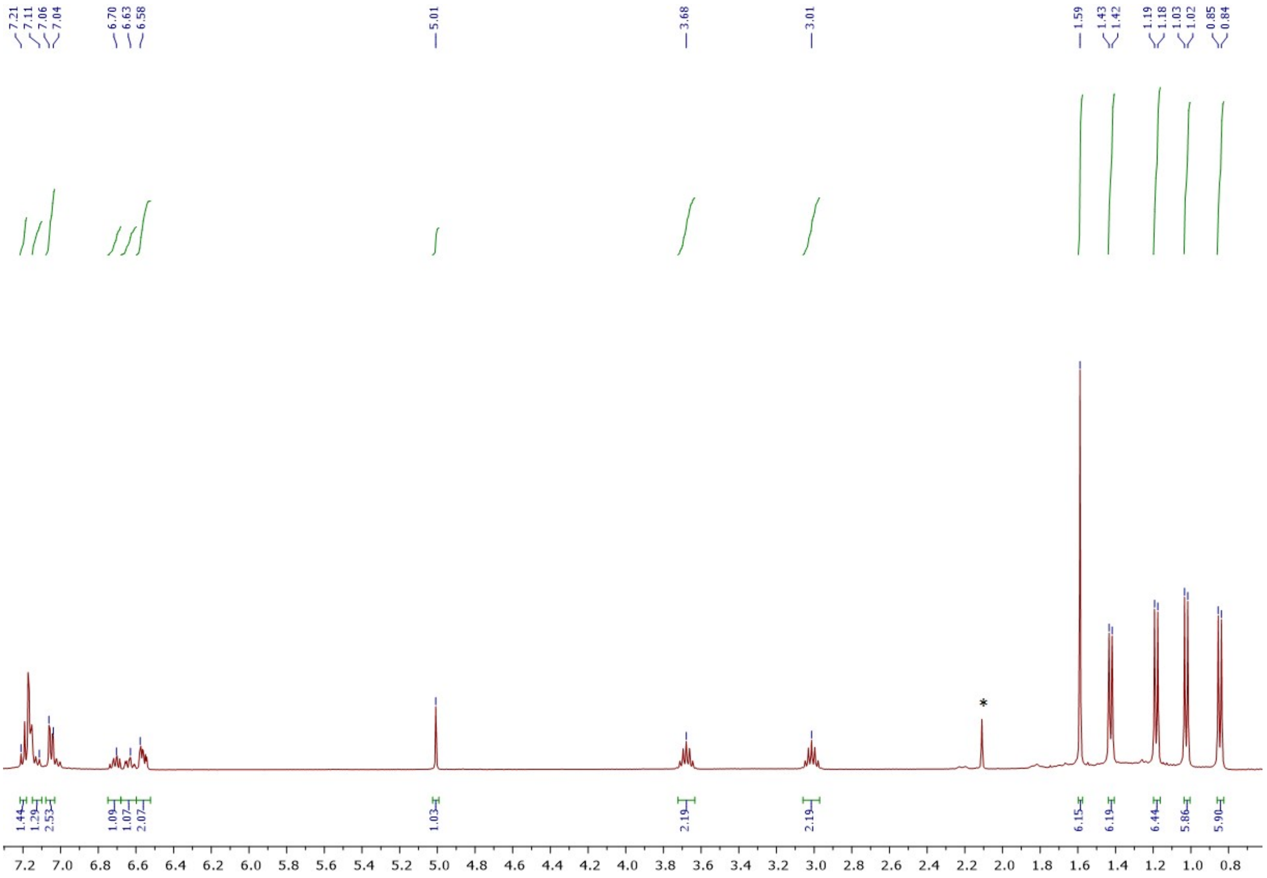

Figure S.3.8: $^{19}F\{^{1}H\}$ NMR spectrum of complex **2c** (* residual toluene)

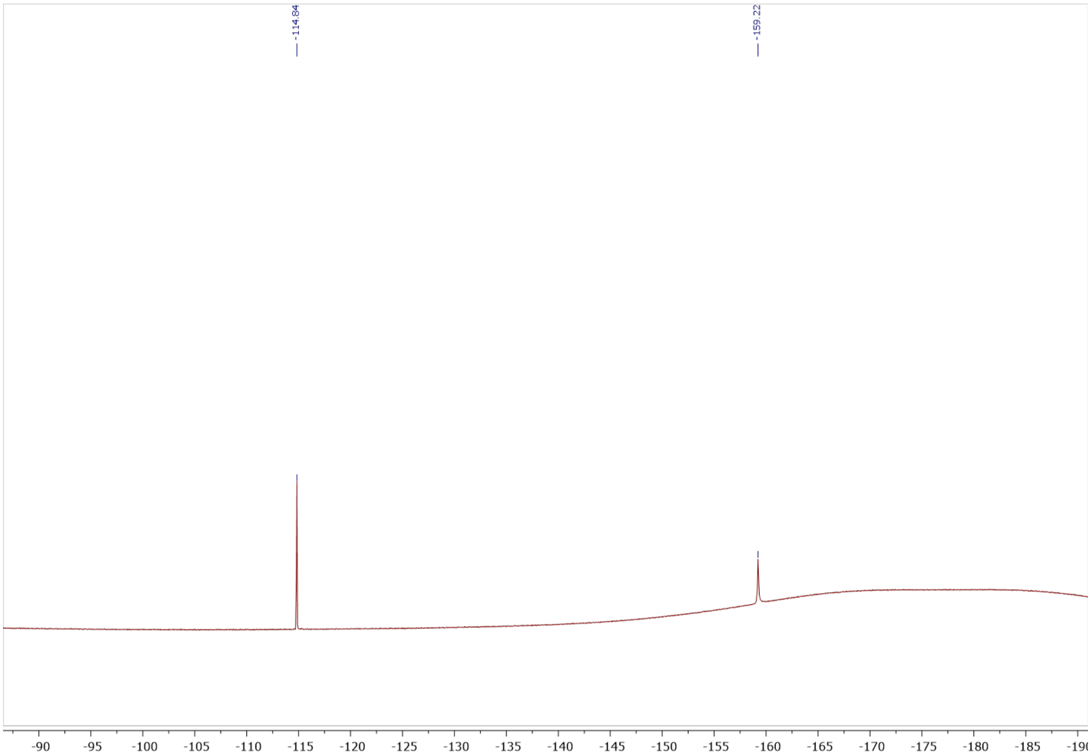

Figure S.3.9: $^{13}C\{^{1}H\}$ NMR spectrum of complex **2c**

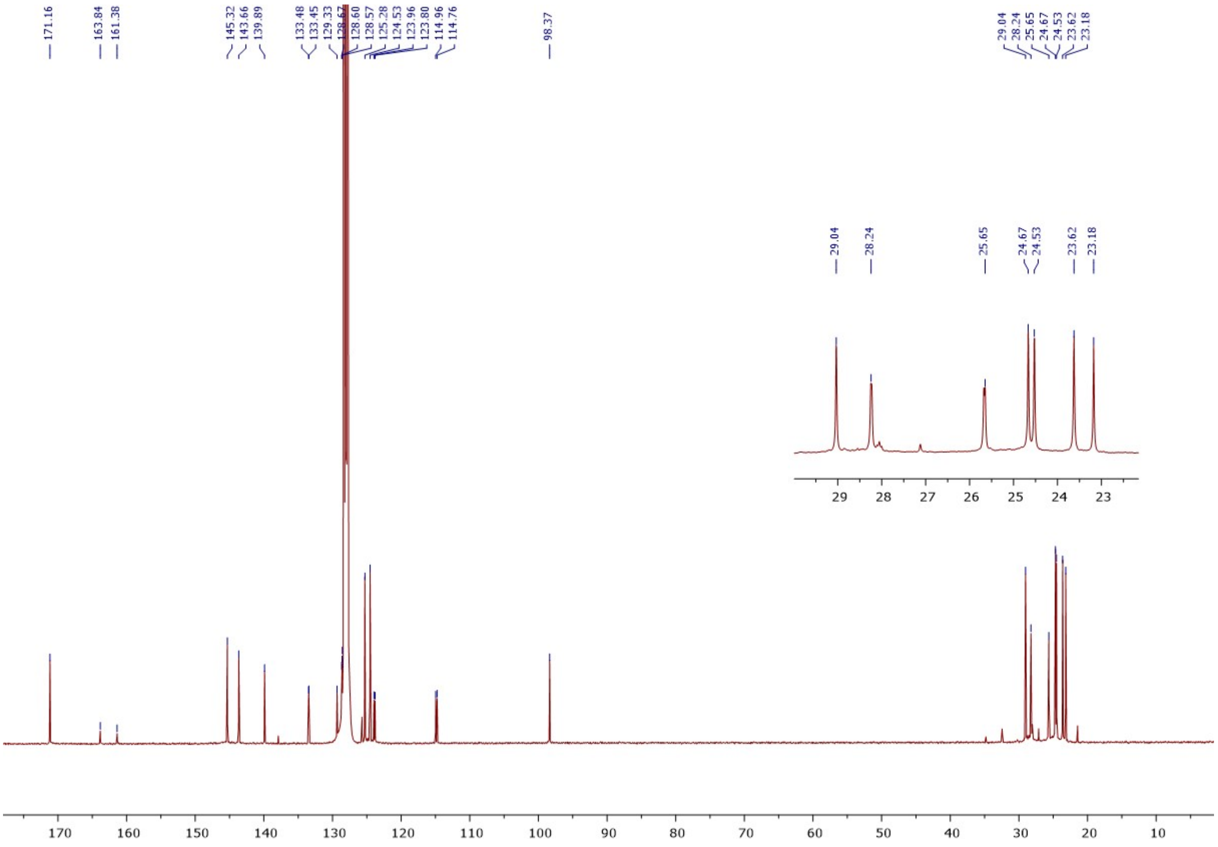

Figure S.3.10: ^{1}H NMR spectrum of complex **2d** (* residual hexane)

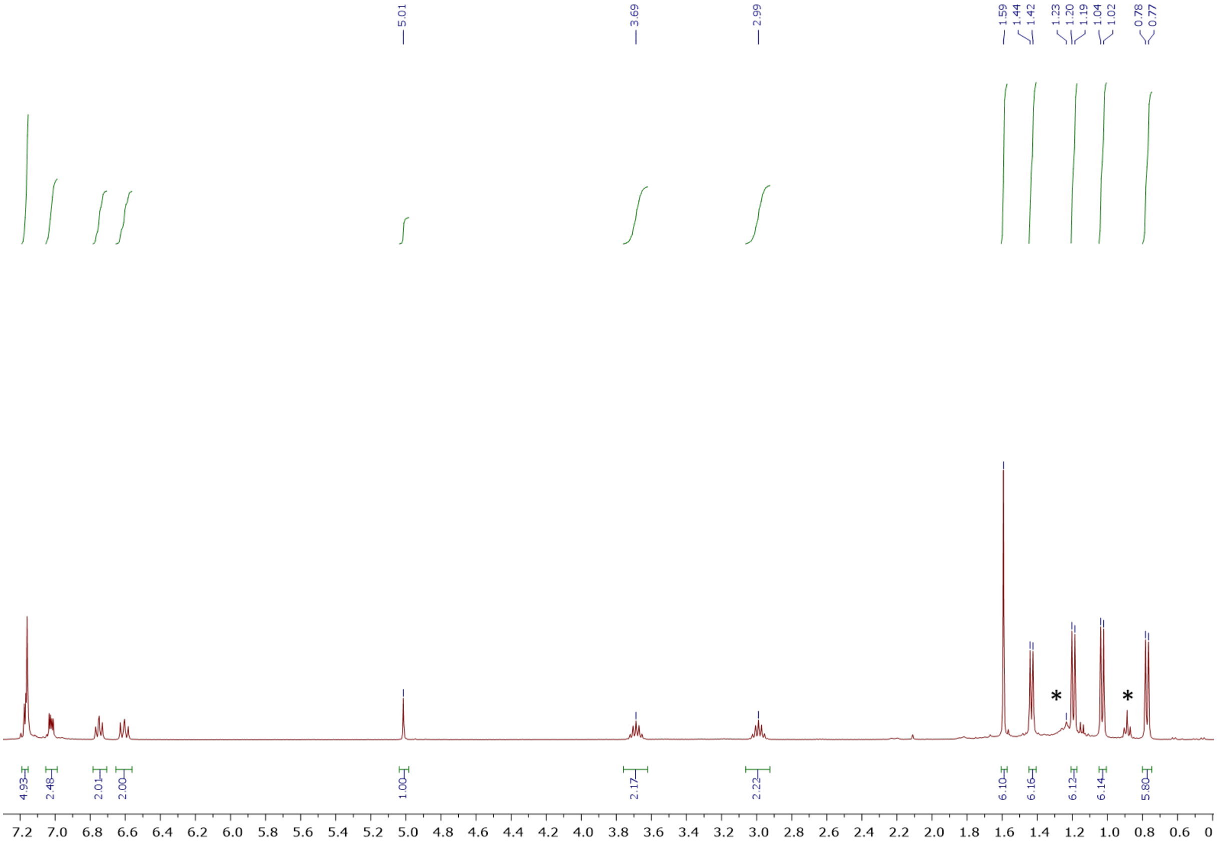

Figure S.3.11: ^{19}F{^{1}H} NMR spectrum of complex **2d**

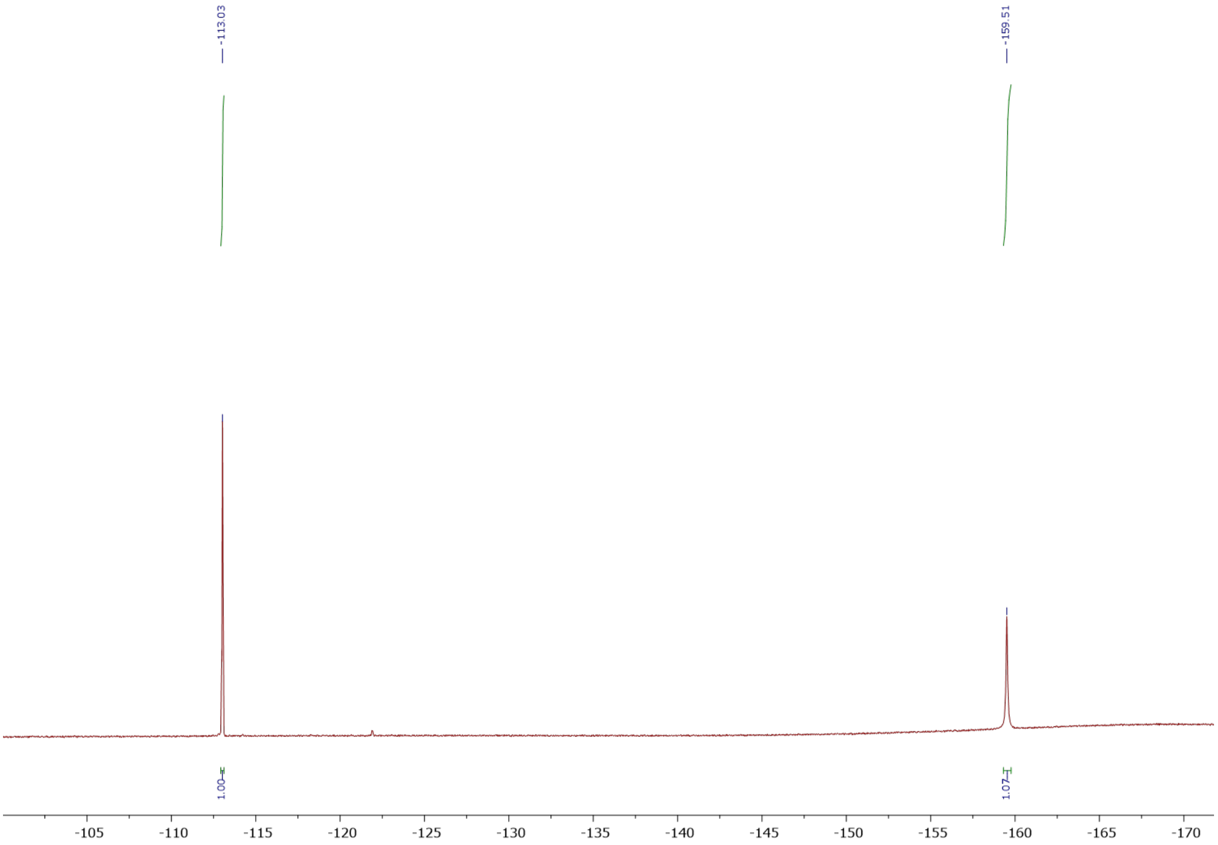

Figure S.3.12: $^{13}C\{^1H\}$ NMR spectrum of complex **2d** (* residual hexane)

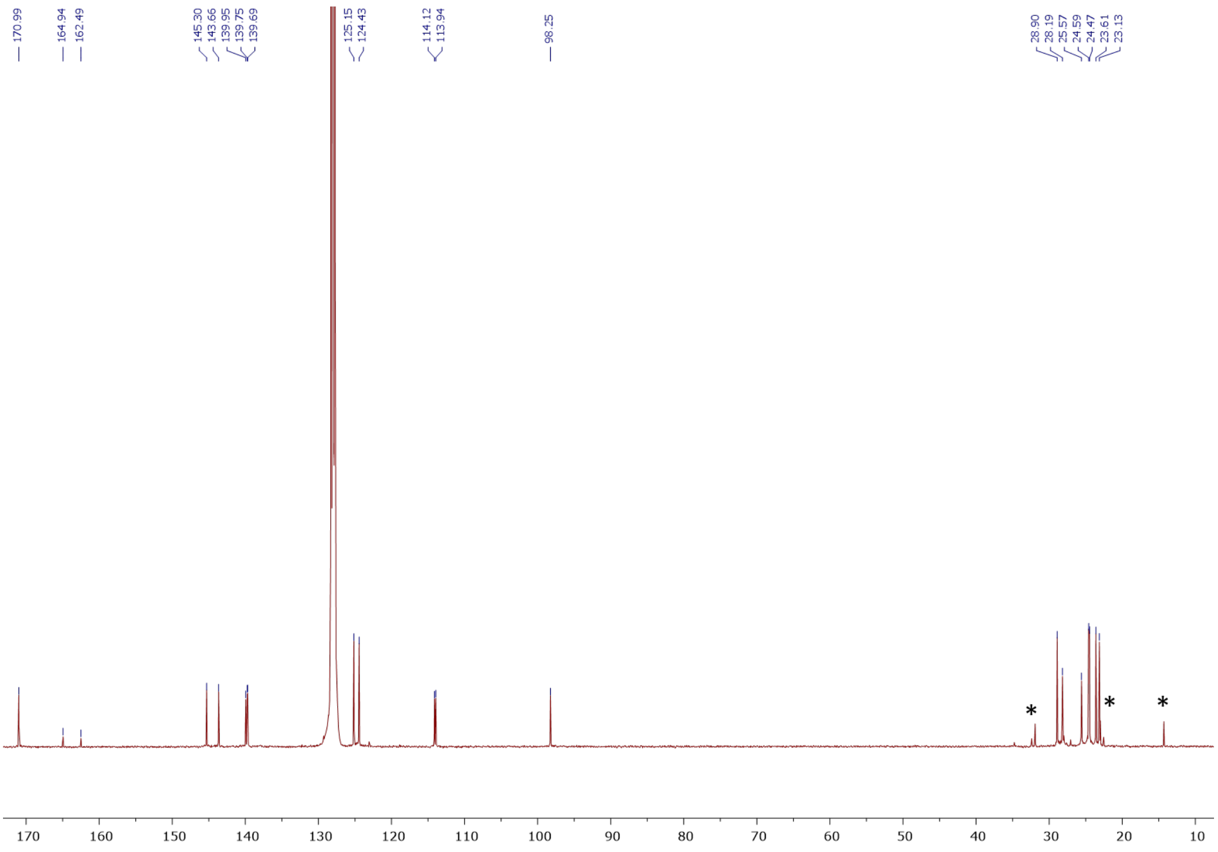

Figure S.3.13: ^{1}H NMR spectrum of complex **2e** (* residual toluene)

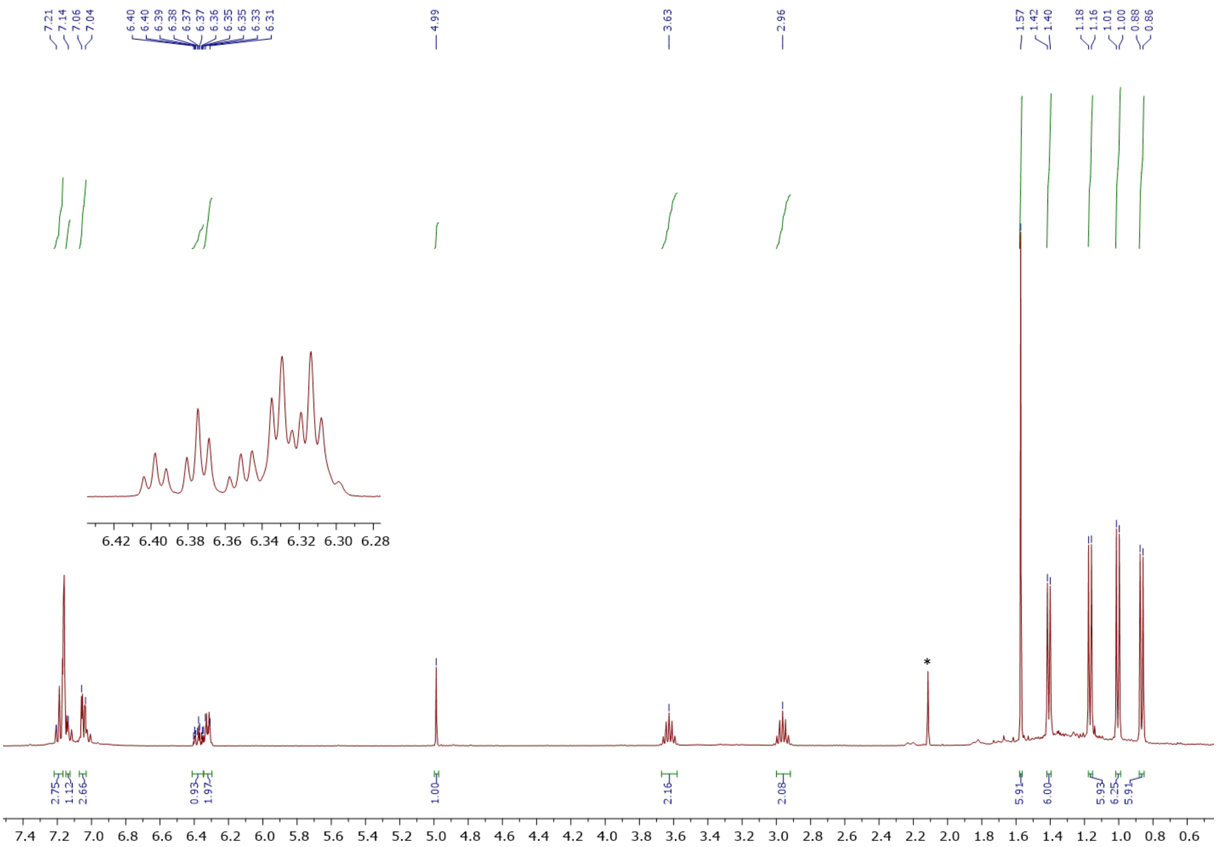

Figure S.3.14: ^{19}F NMR spectrum of complex **2e** (* residual toluene)

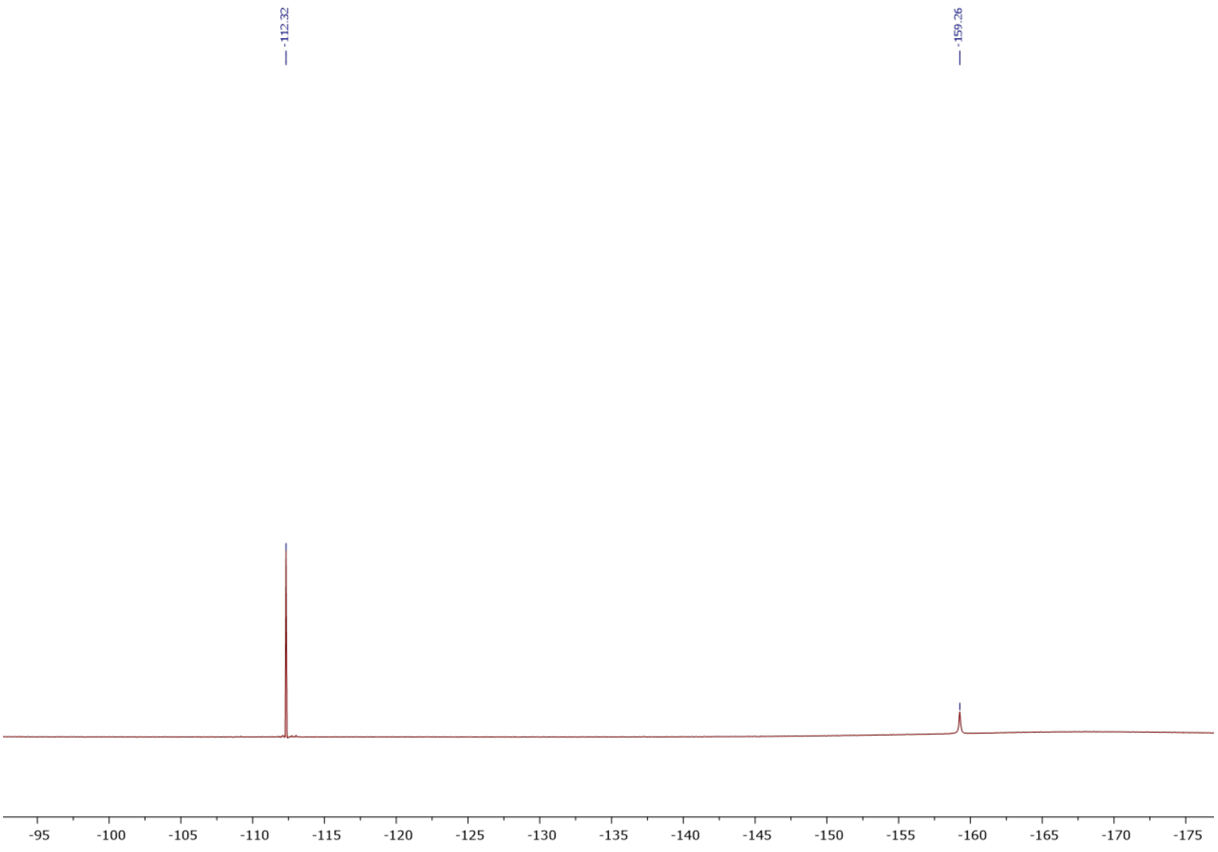

Figure S.3.15: $^{13}C\{^{1}H\}$ NMR spectrum of complex **2e**

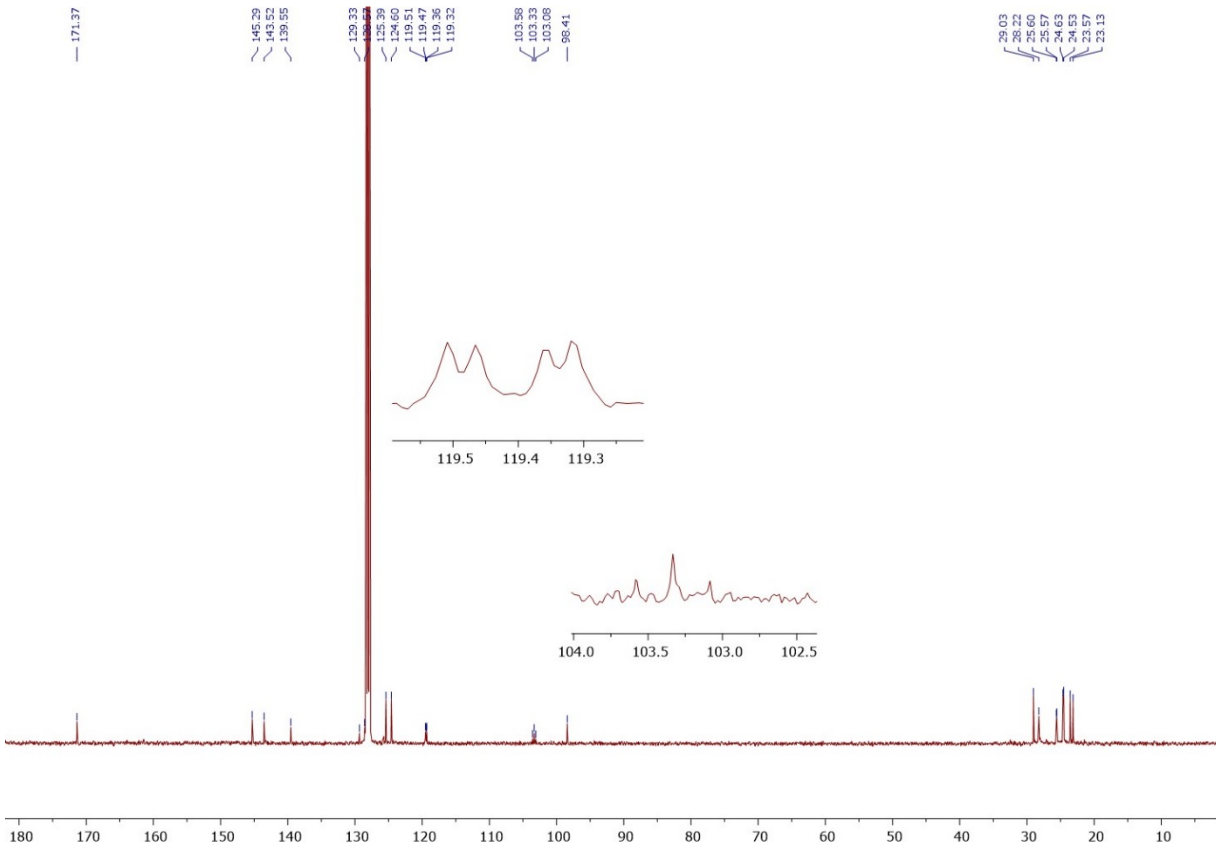

Figure S.3.16: ^{1}H NMR spectrum of complex **2f**

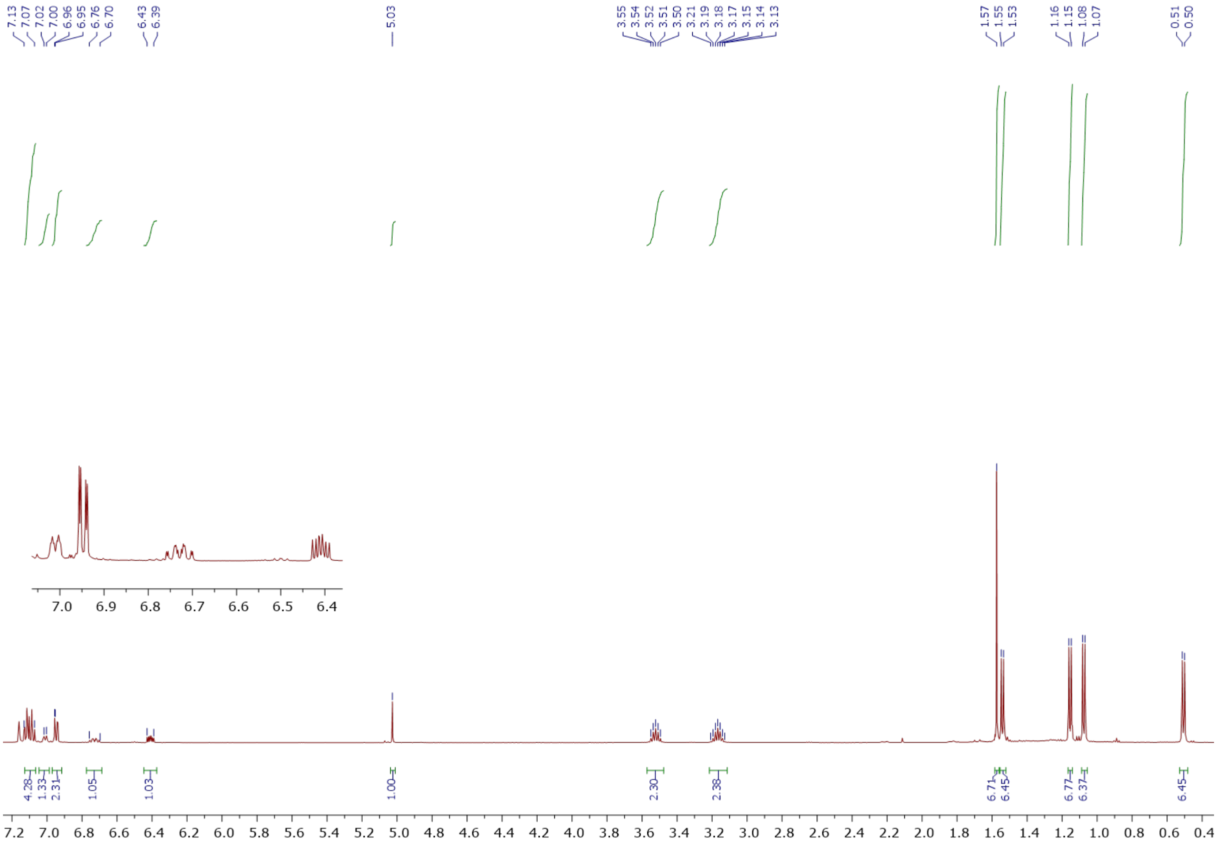

Figure S.3.17: ^{19}F NMR spectrum of complex **2f**

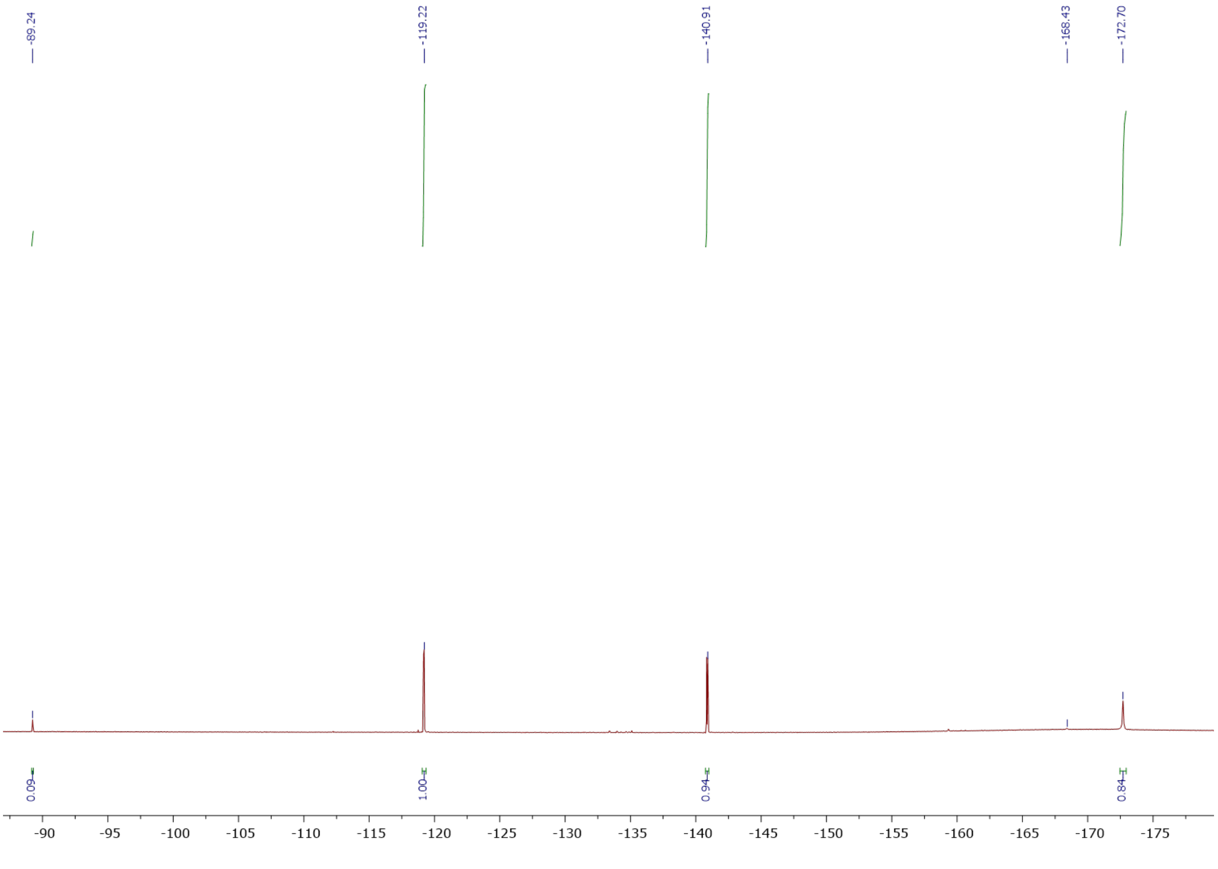

Figure S.3.18: $^{13}C\{^1H\}$ NMR spectrum of complex **2f**

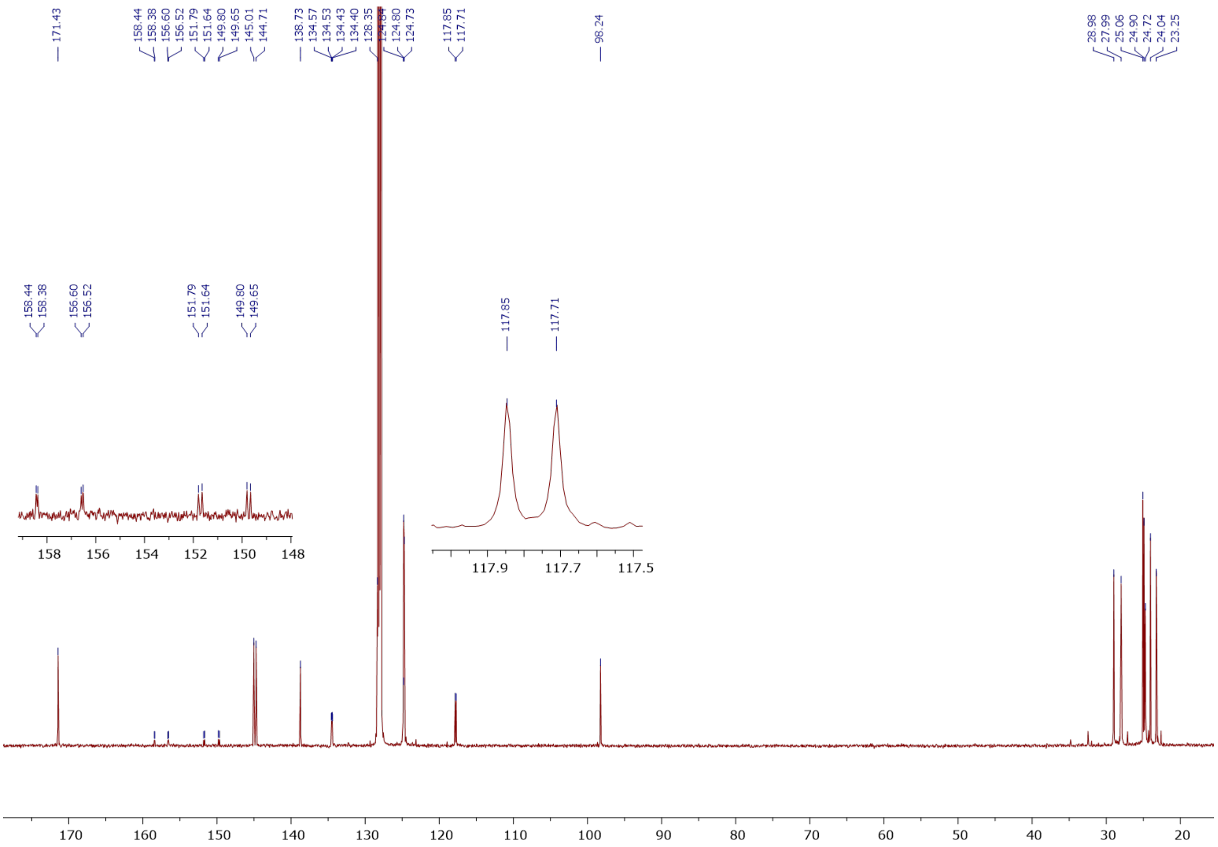

Figure S.3.19: ^{1}H NMR spectrum of complex **2g**

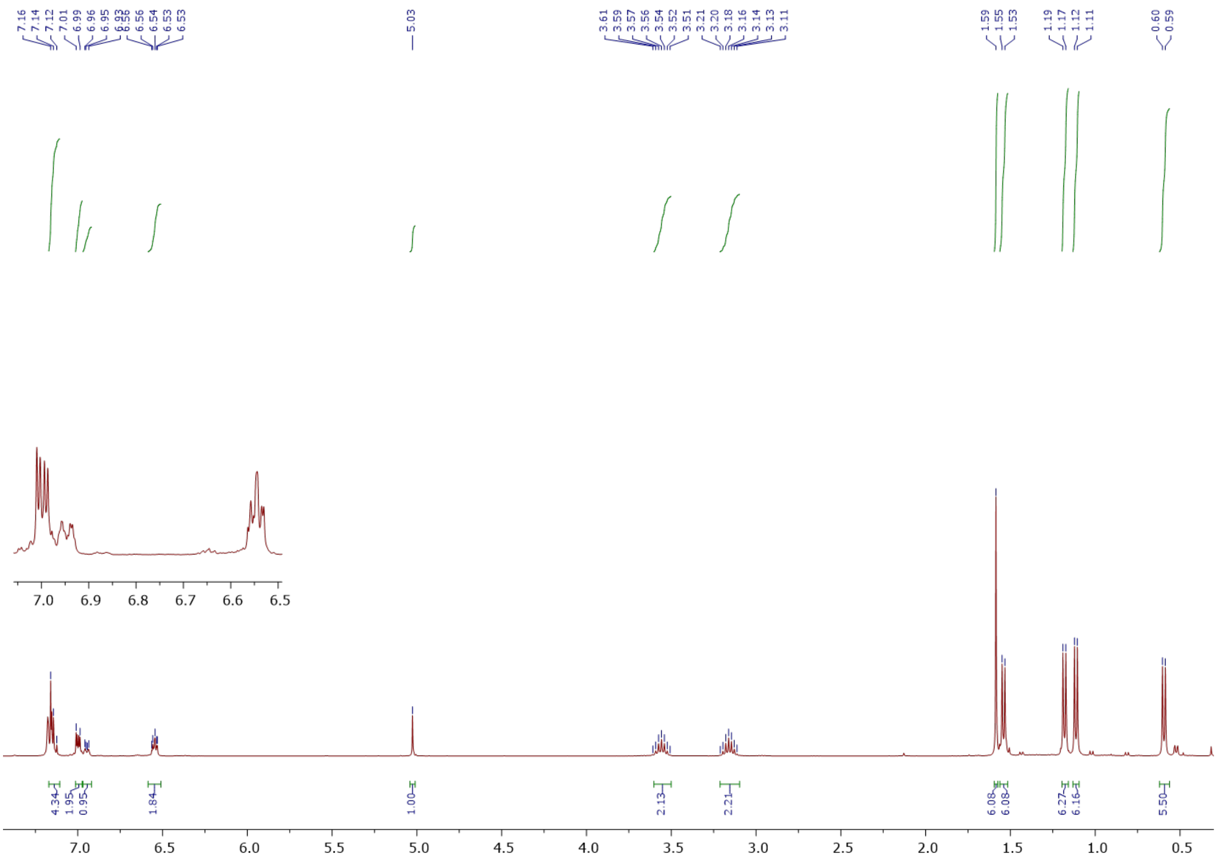

Figure S.3.20: ^{19}F{^{1}H} NMR spectrum of complex **2g**

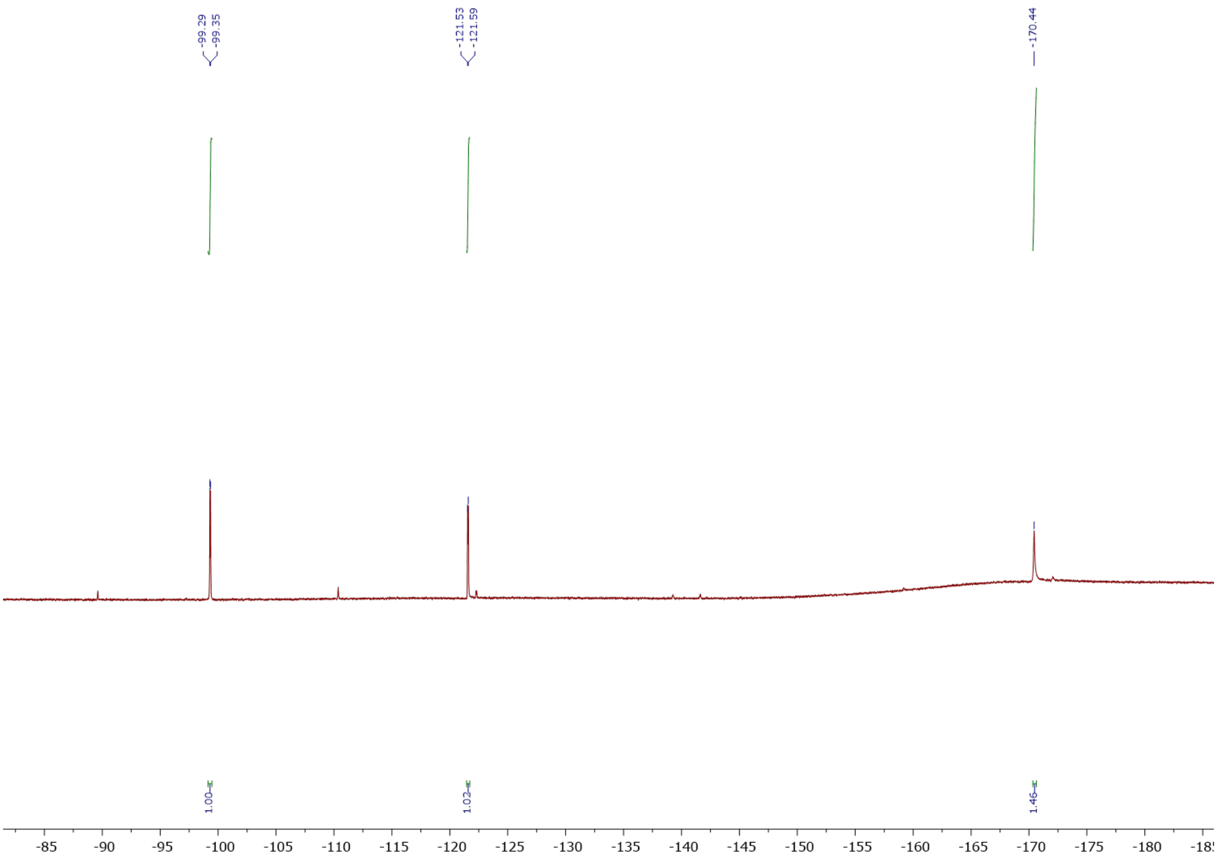

Figure S.3.21: $^{13}C\{^{1}H\}$ NMR spectrum of complex **2g**

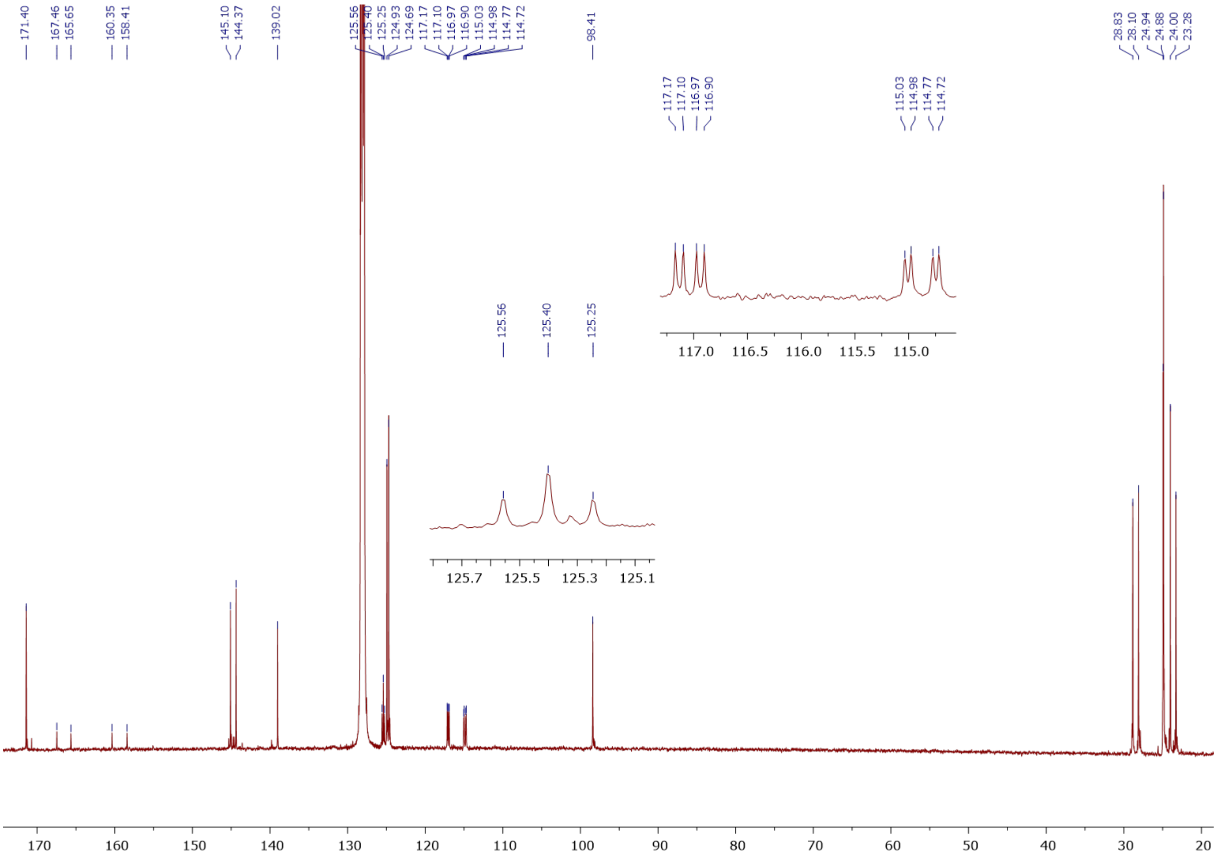

Figure S3.22: ^{1}H NMR spectrum of complex **2h**

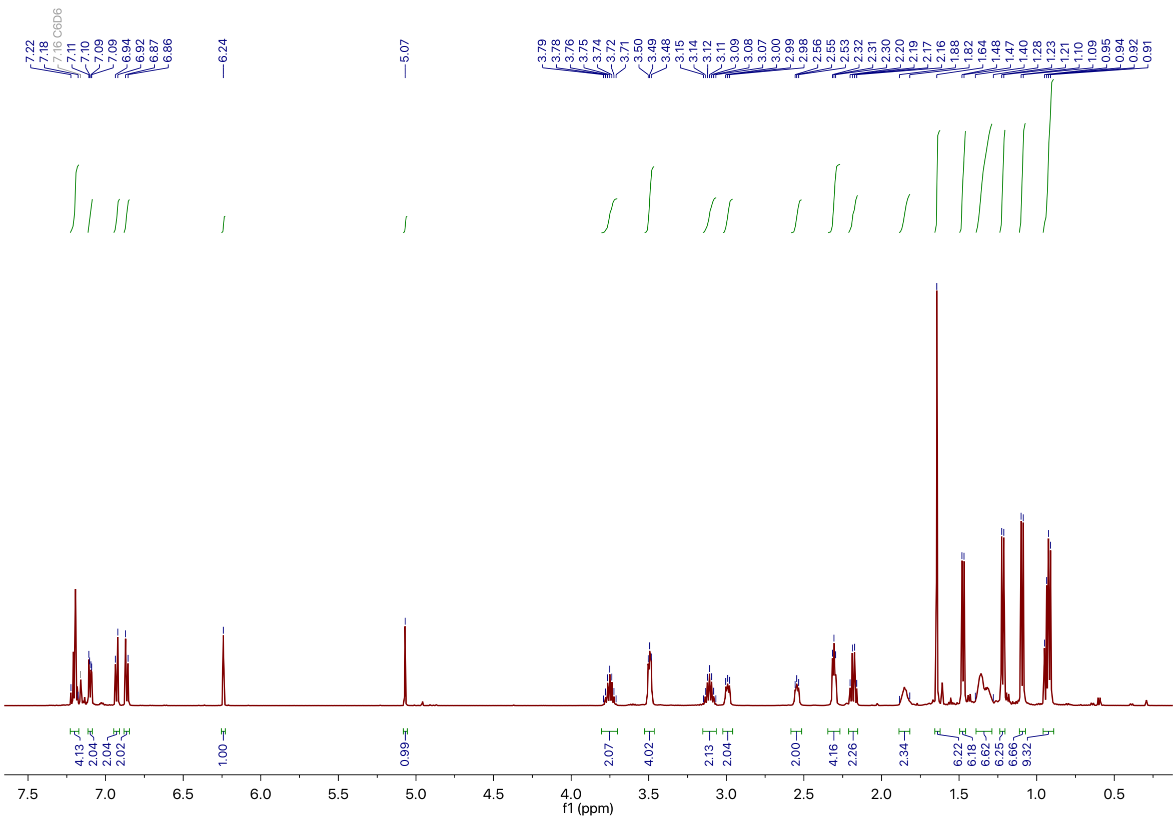

Figure S3.23: ^{19}F{^{1}H} NMR spectrum of complex **2h**

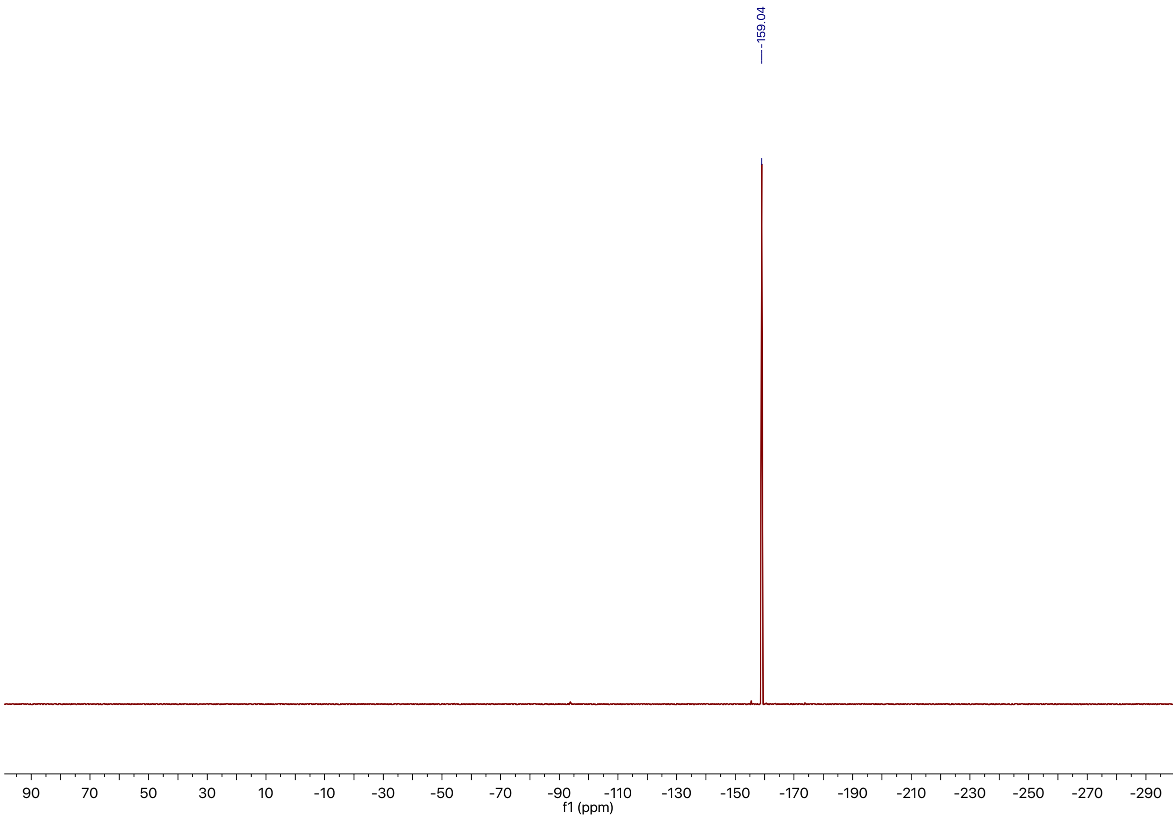

Figure S3.24: $^{13}C\{^{1}H\}$ spectrum of complex **2h**

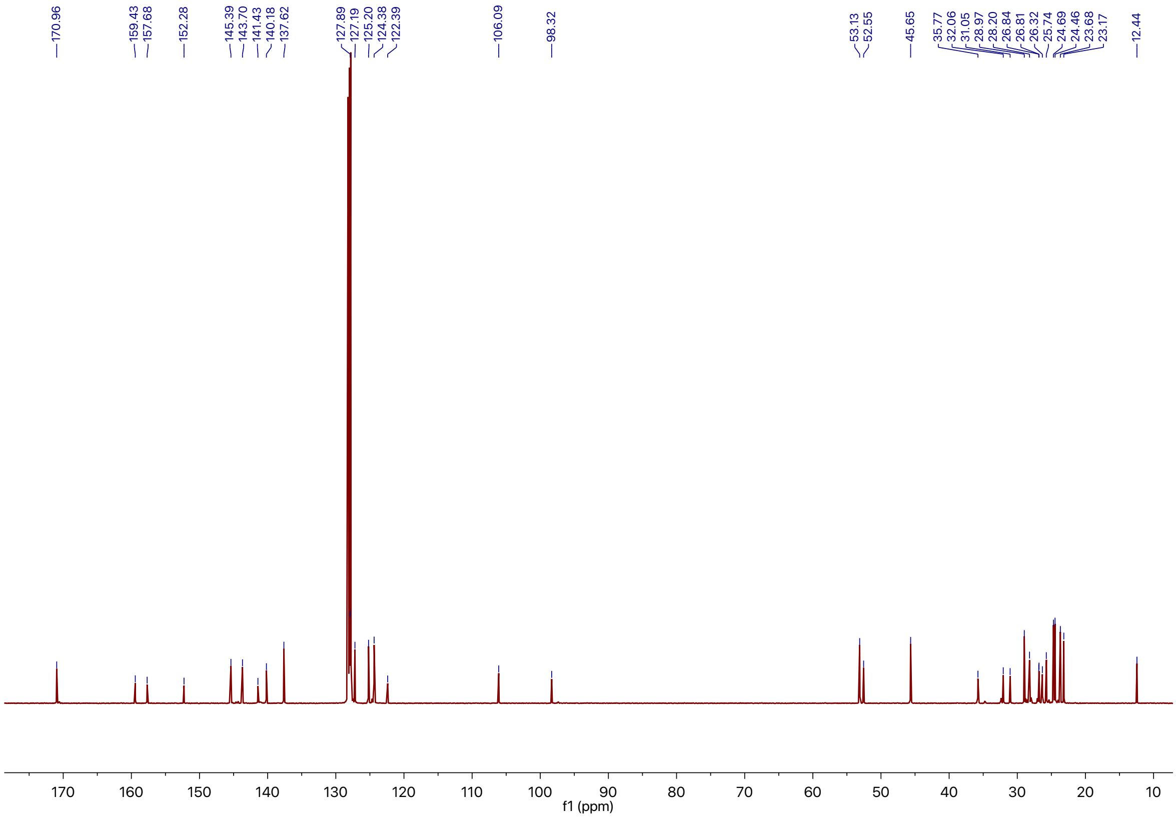

Figure S3.25: COSY (^{1}H-^{1}H) spectrum of complex **2h**

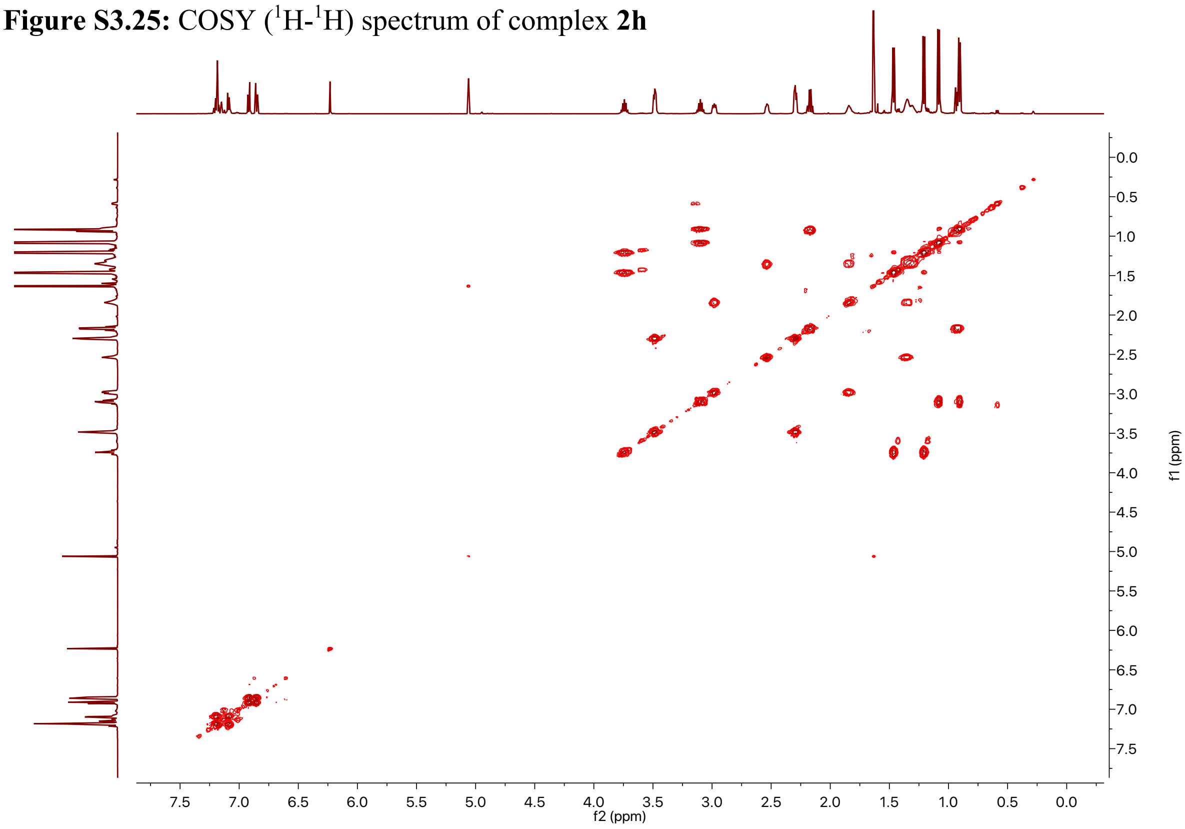

Figure S3.26: HSQC (^{1}H-^{13}C) spectrum of complex **2h**

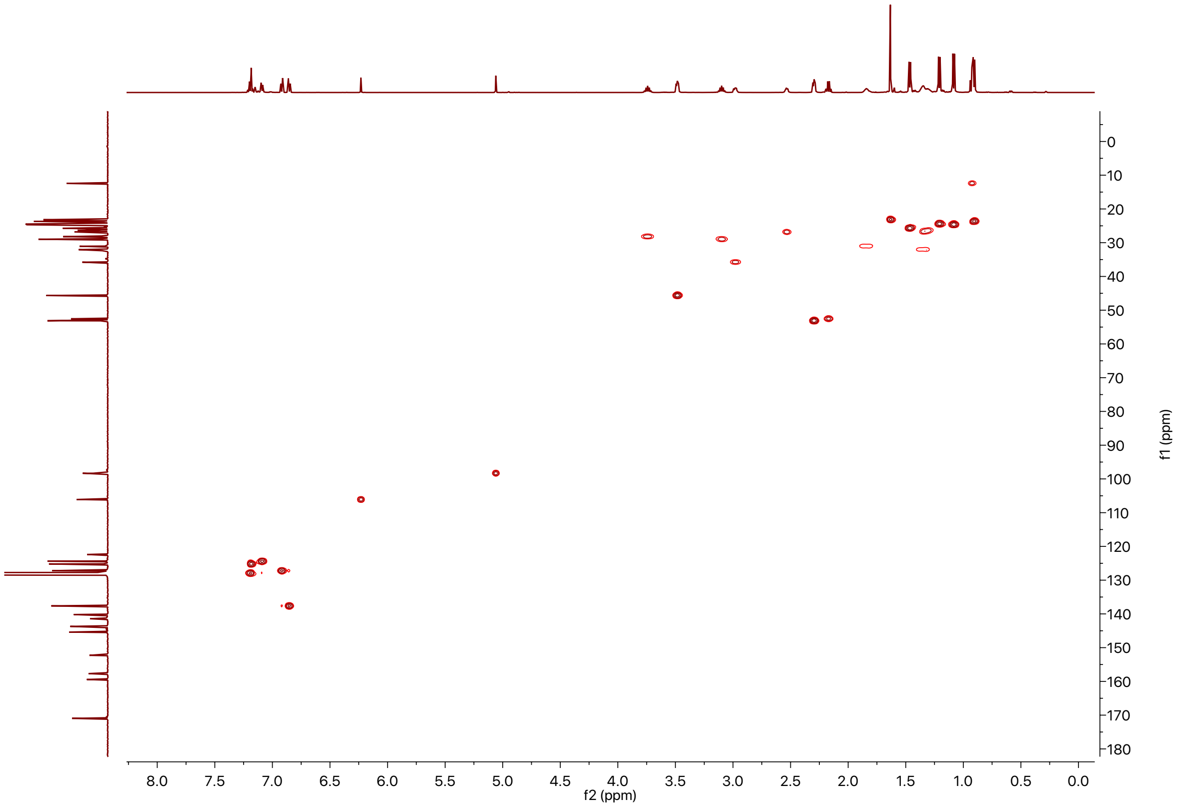

Figure S3.27: HMBC (^{1}H-^{13}C) spectrum of complex **2h**

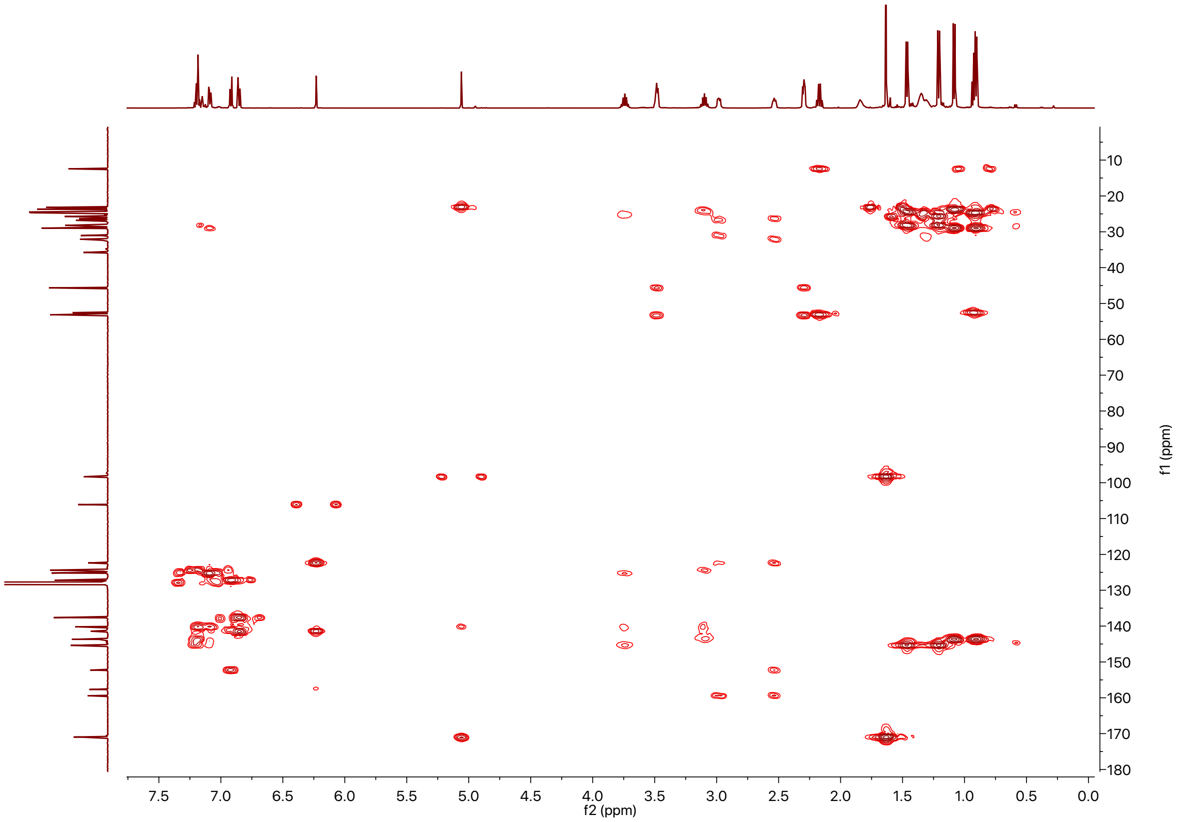

Figure S3.28: NOESY spectrum of complex **2h**

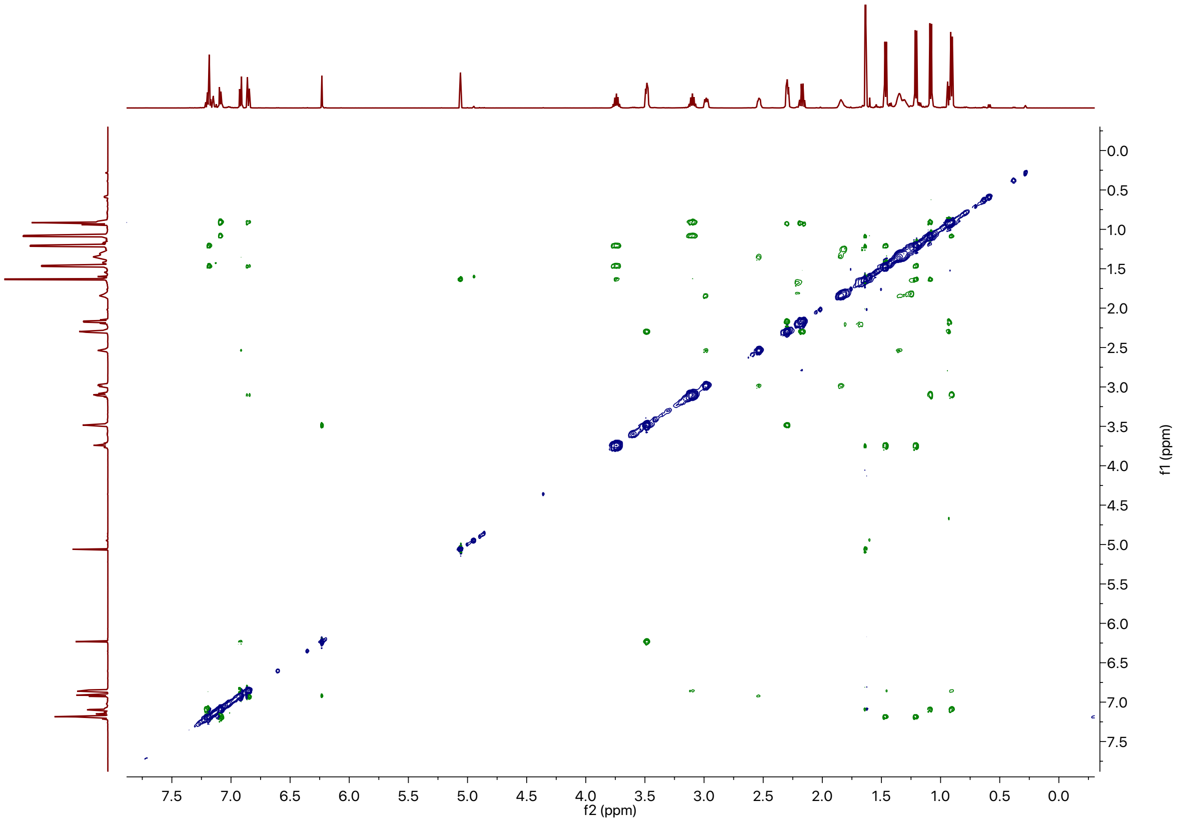

Figure S.3.29: ^{1}H NMR spectrum of the low temperature monitoring of the reaction of **1** with monofluorobenzene to give **2a** (* indicates the formation of product **2a**).

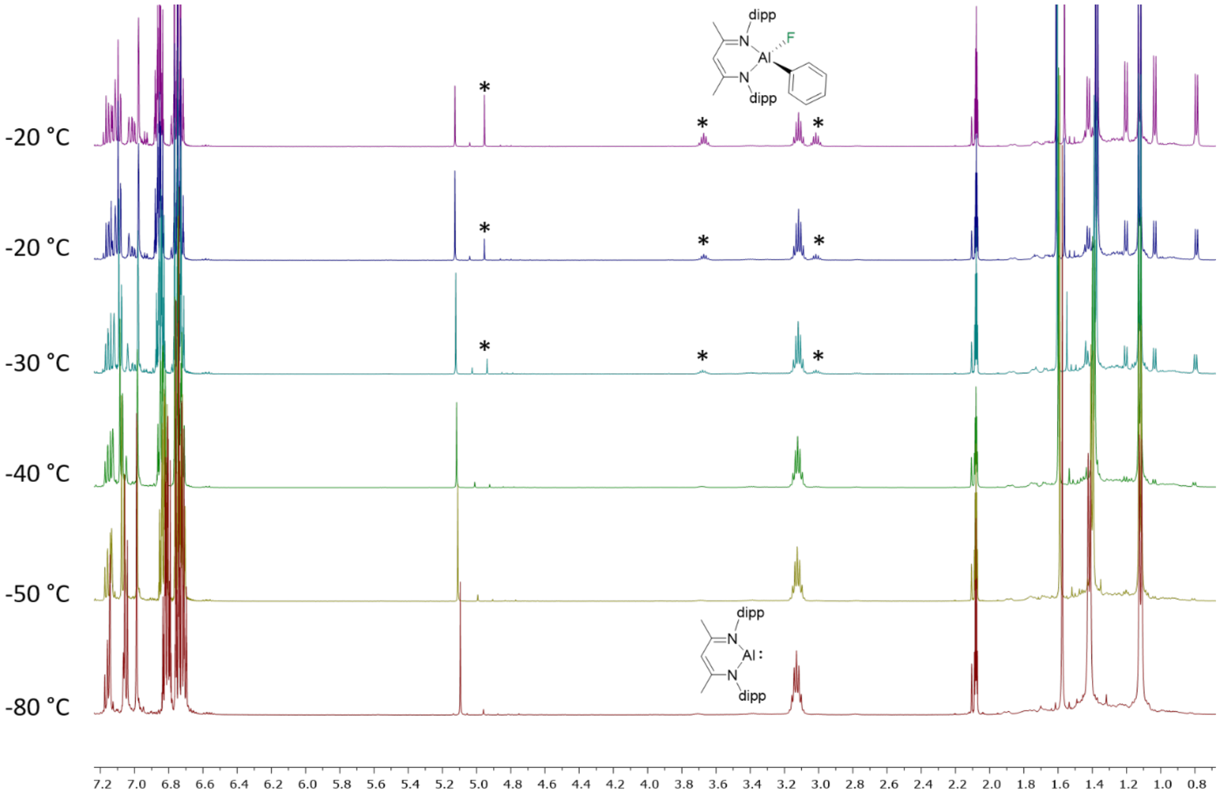

Figure S.3.30: ^{1}H NMR spectrum of the low temperature monitoring of the reaction of **1** with 1,3-difluorobenzene to give **2c** (* indicates the formation of product **2c**).

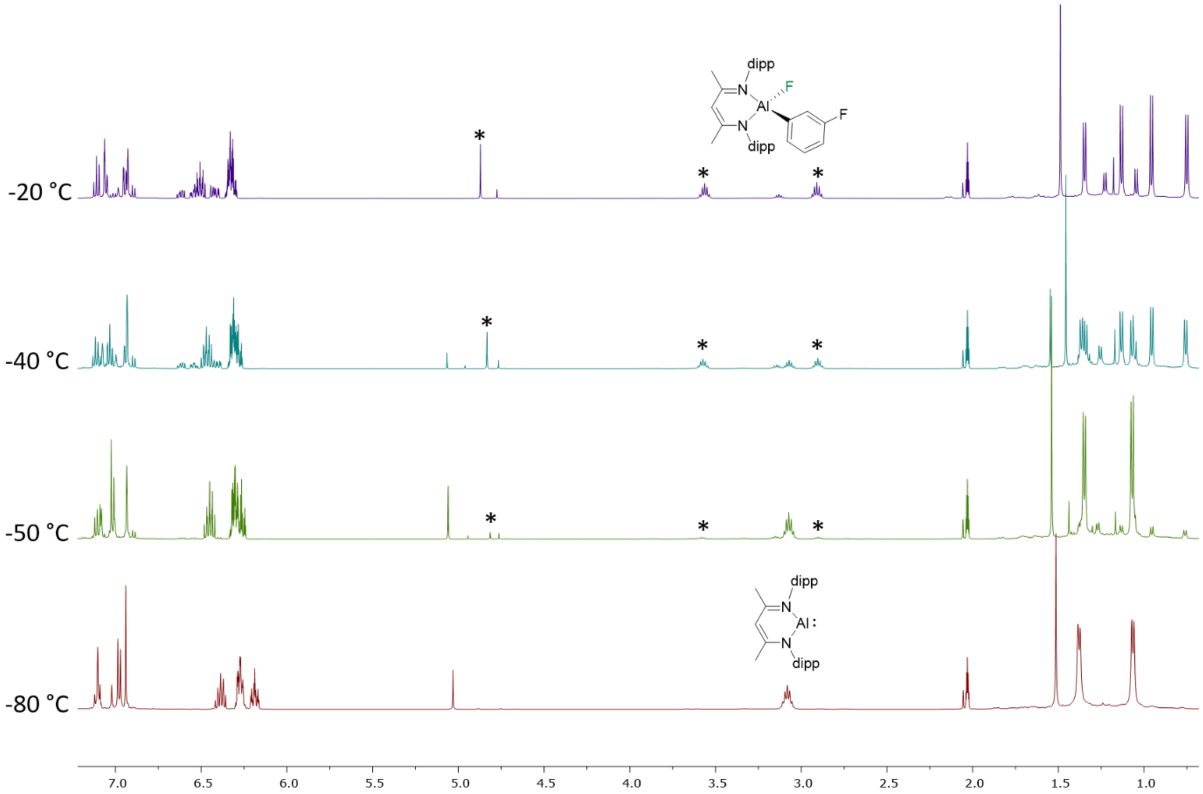

Figure S.3.31: $^{19}F\{^1H\}$ NMR spectrum of the reaction of [Pd(**1**)$_2$(PCy$_3$)] with 1,3-difluorobenzene (*excess of difluorobenzene).

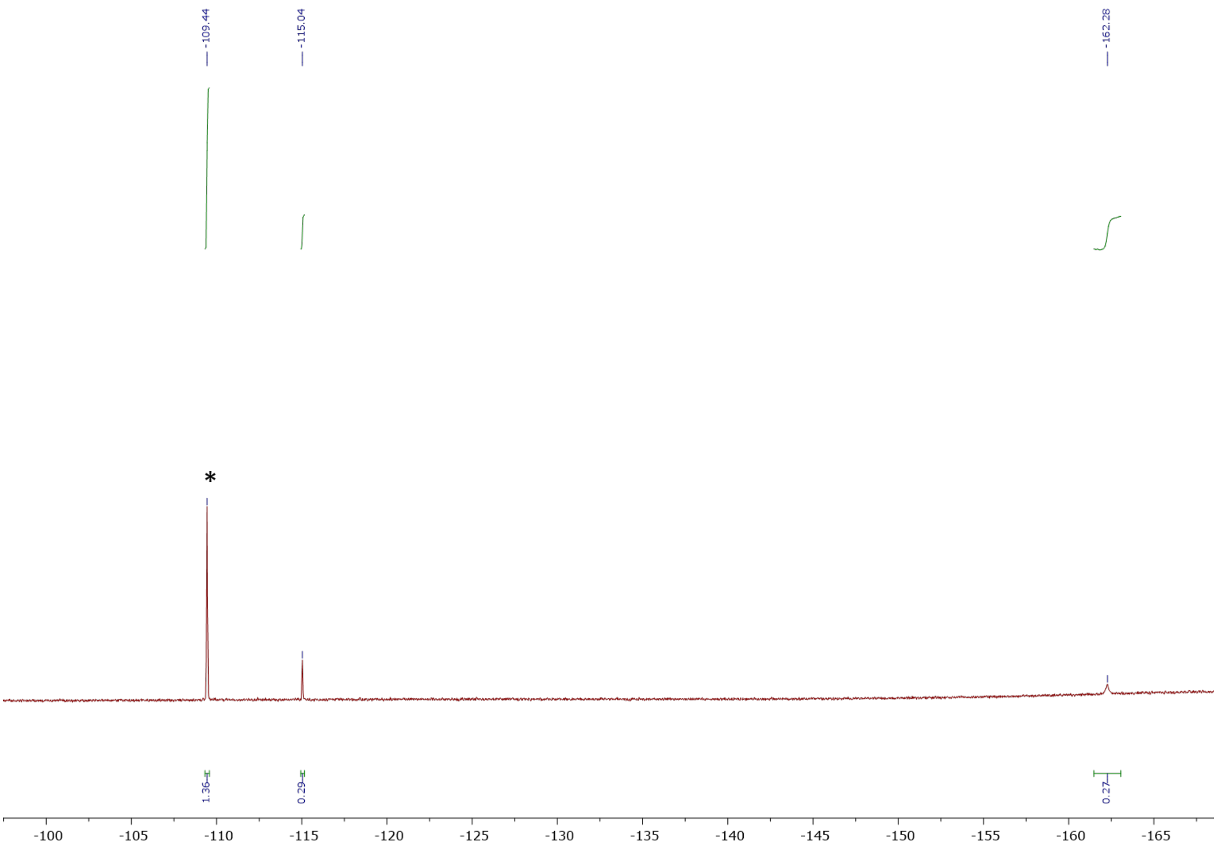

Figure S.3.32: ^{19}F NMR spectrum of the [Pd(PCy$_3$)$_2$] catalysed reaction of 1,3-difluorobenzene with **3**.

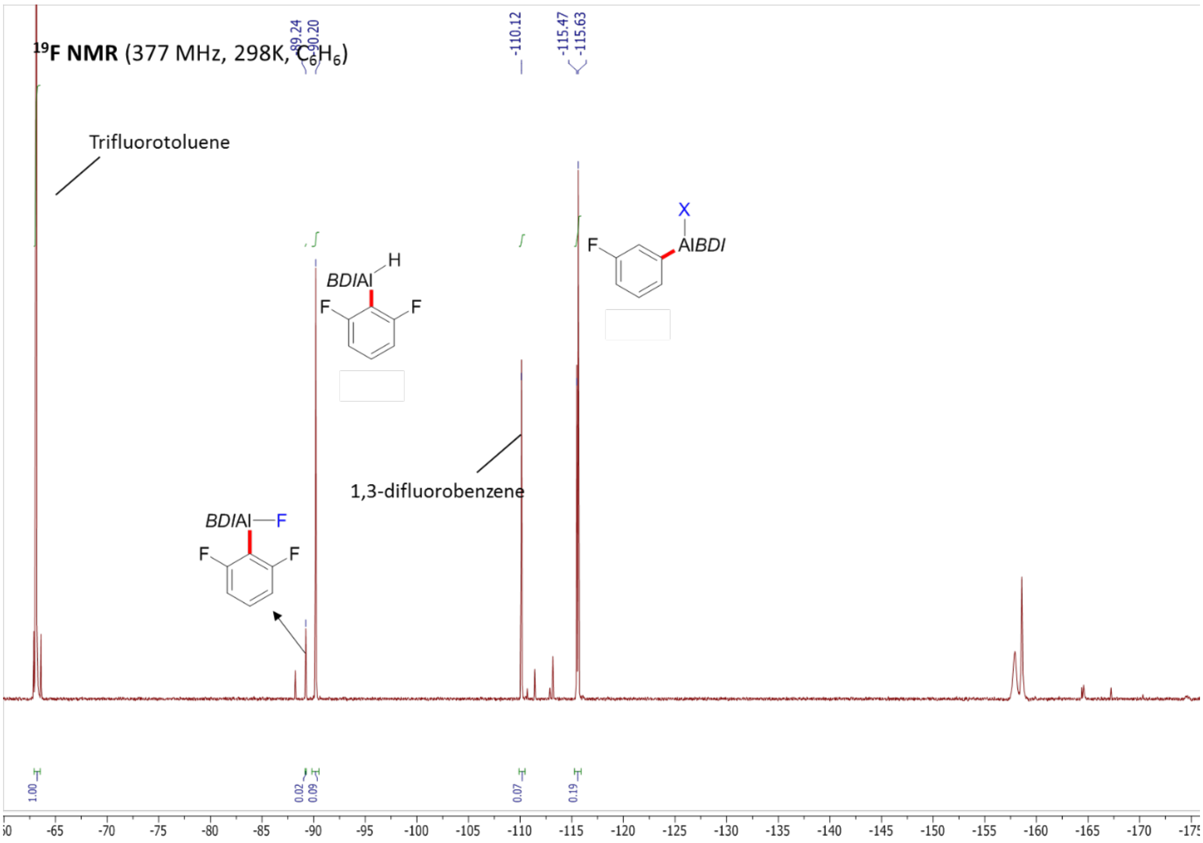

Figure S.3.33: ^{1}H NMR spectrum of the isolated **5c/5c'** from the catalytic reaction with 1,3-difluorobenzene.

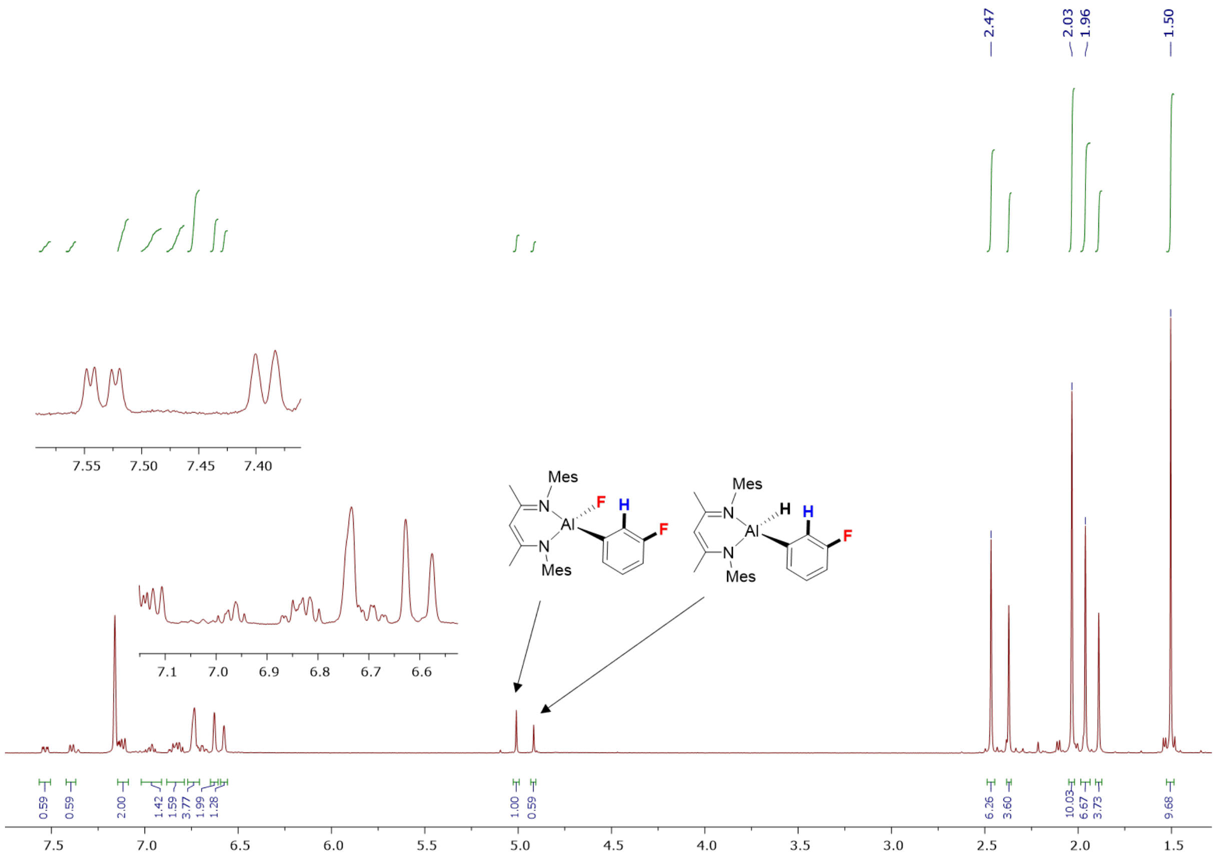

Figure S.3.34: ^{19}F NMR spectrum of the isolated **5c/5c'** from the catalytic reaction with 1,3-difluorobenzene.

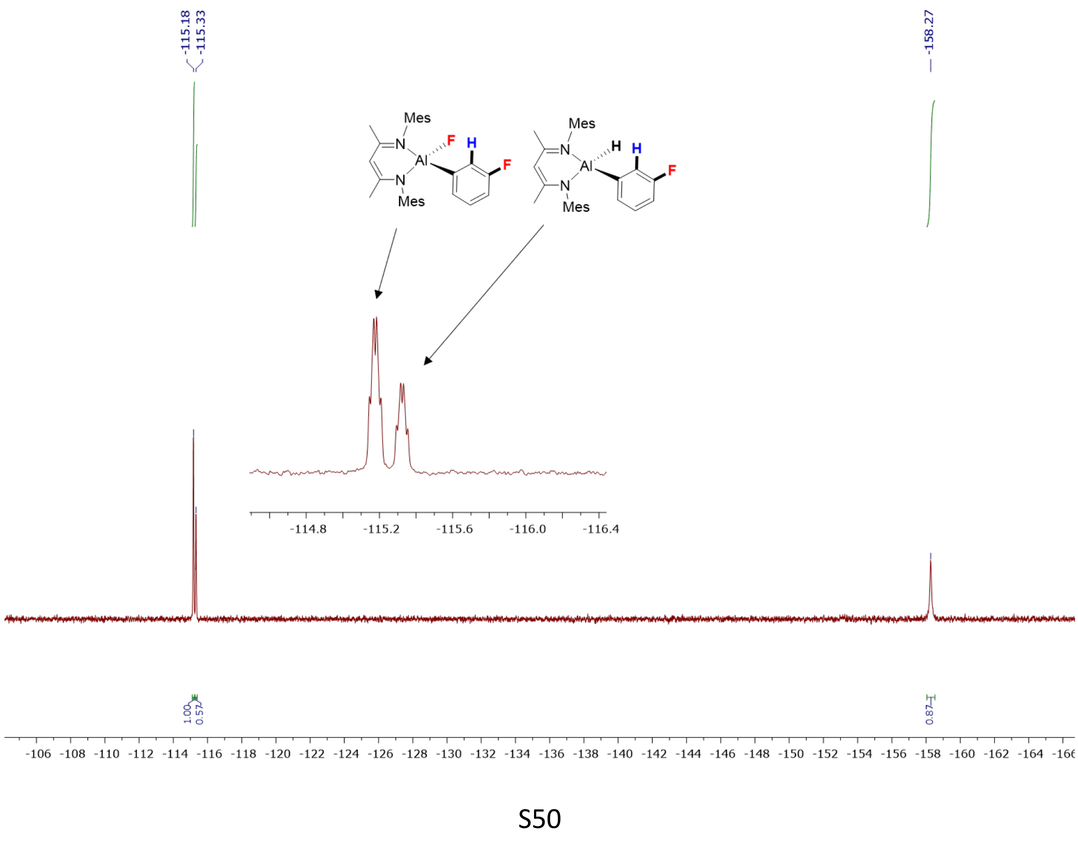

Figure S.3.35: COSY NMR spectrum of complex **5c/5c'.**

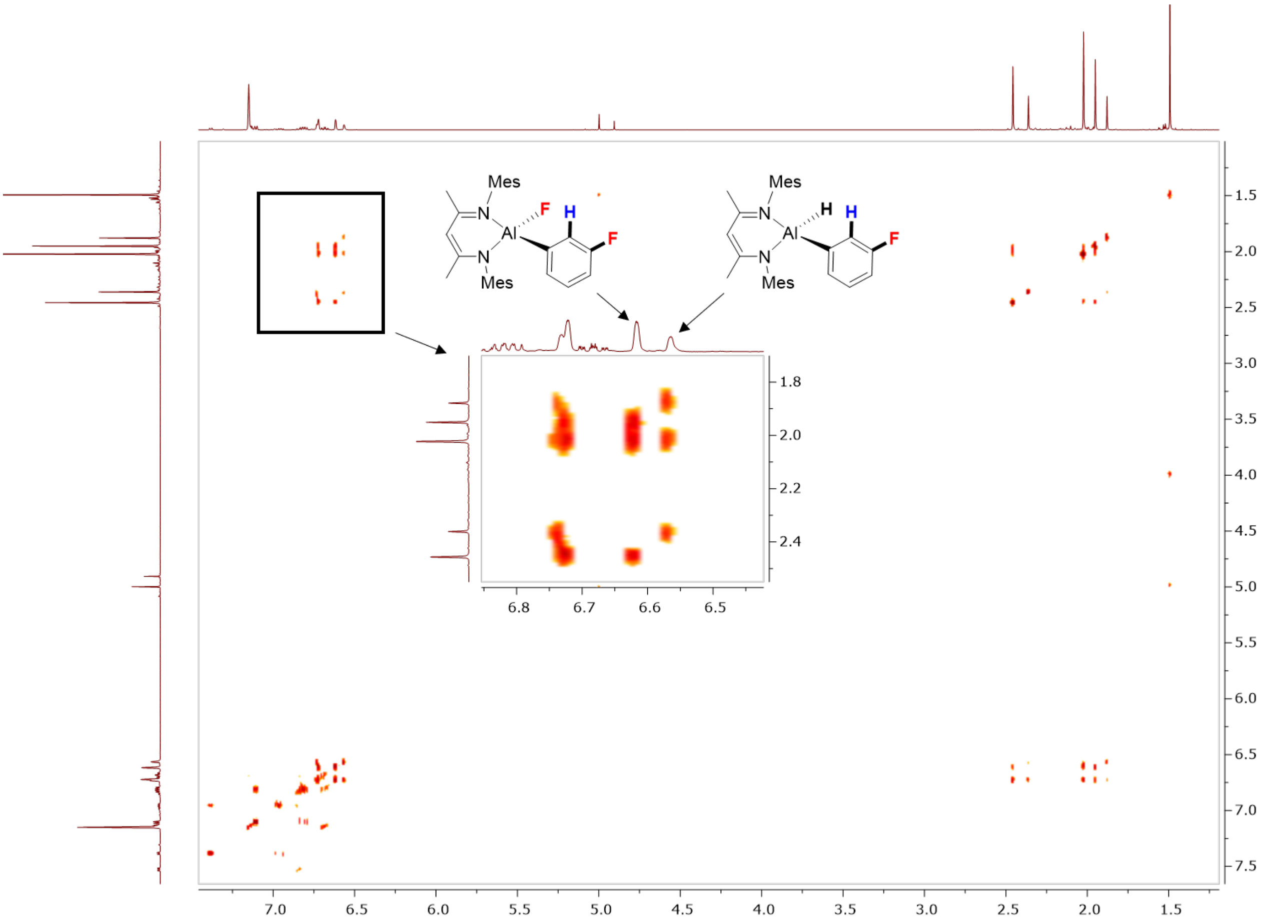

Figure S.3.36: ^{19}F NMR spectrum of the $[Pd(PCy_3)_2]$ catalysed reaction of **3** with 1,2,3-trifluorobenzene.

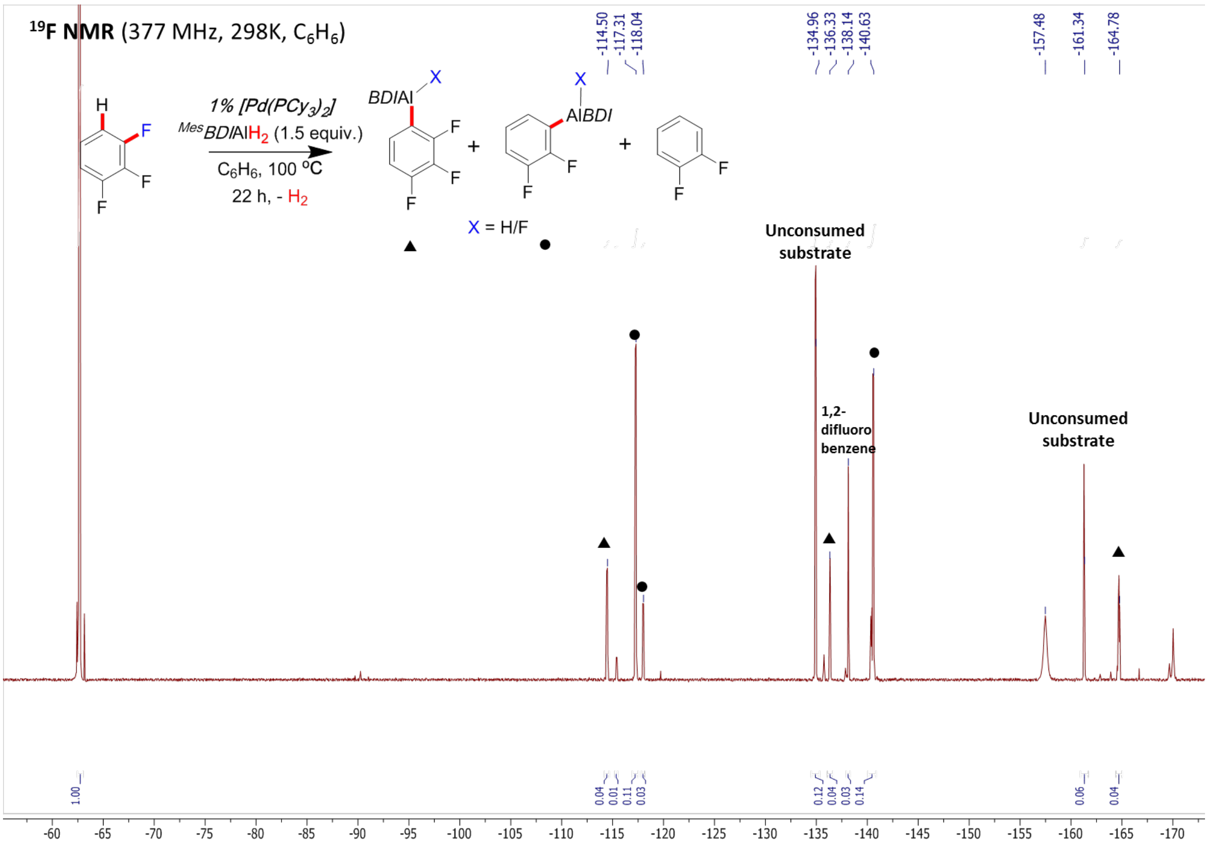

Figure S.3.37: ^{19}F NMR spectrum of the $[Pd(PCy_3)_2]$ catalysed reaction of **3** with 1,2-difluorobenzene.

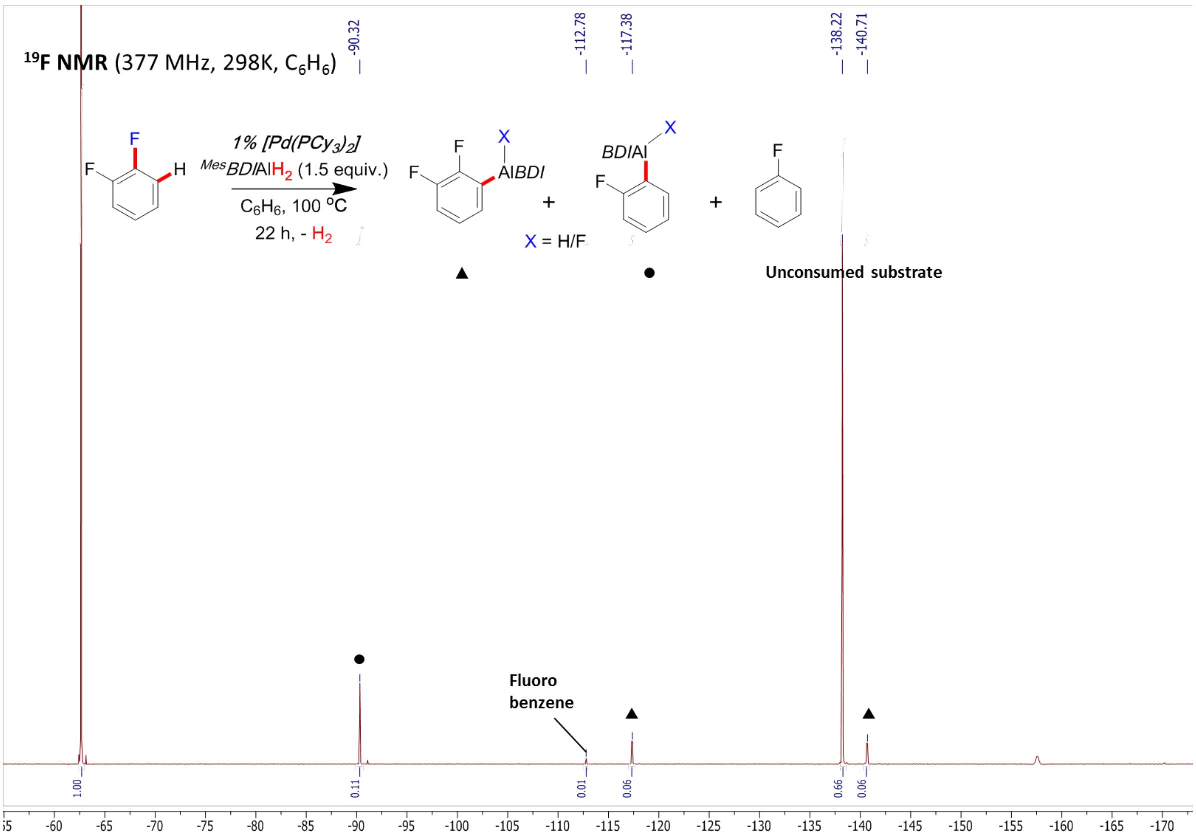

Figure S.3.38: ^{19}F NMR spectrum of the [Pd(PCy$_3$)$_2$] catalyzed reaction of **3** with 1,3,5-trifluorobenzene.

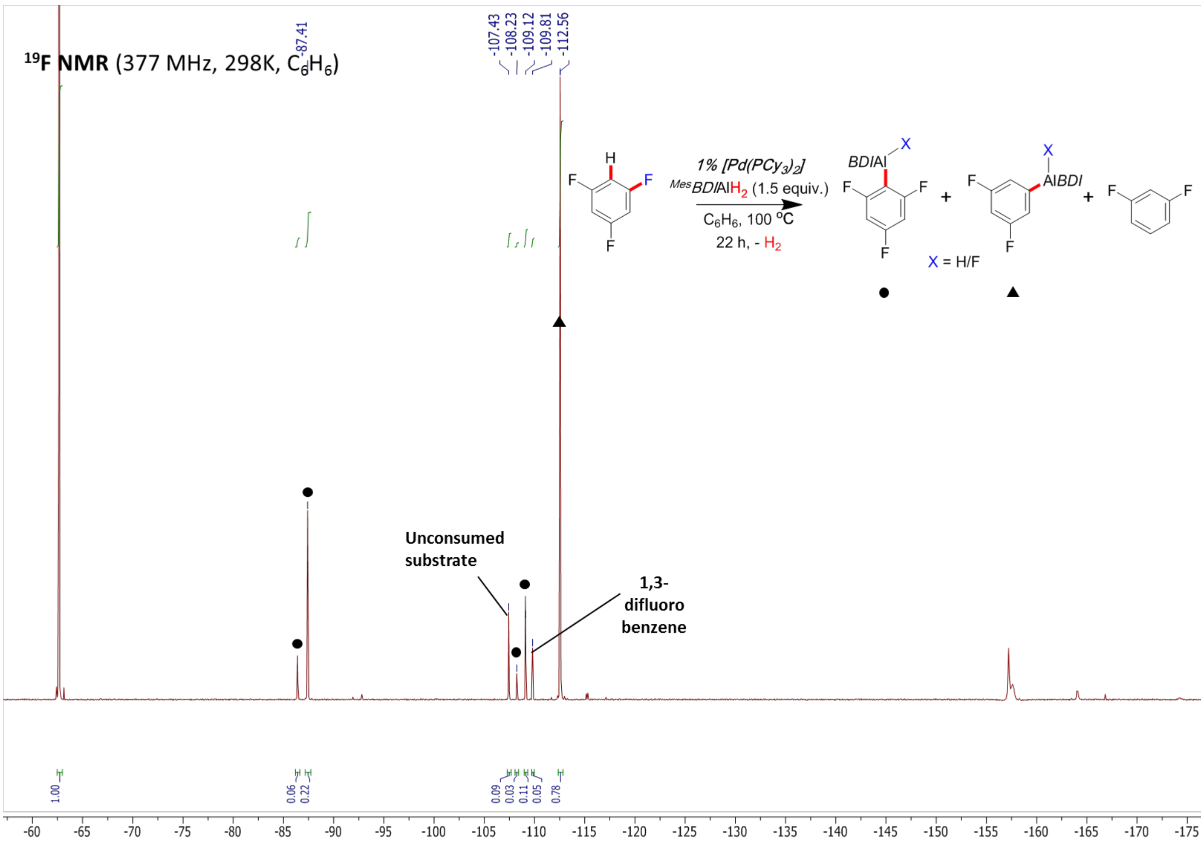

Figure S.3.39: ^{1}H NMR spectrum of complex **5c**

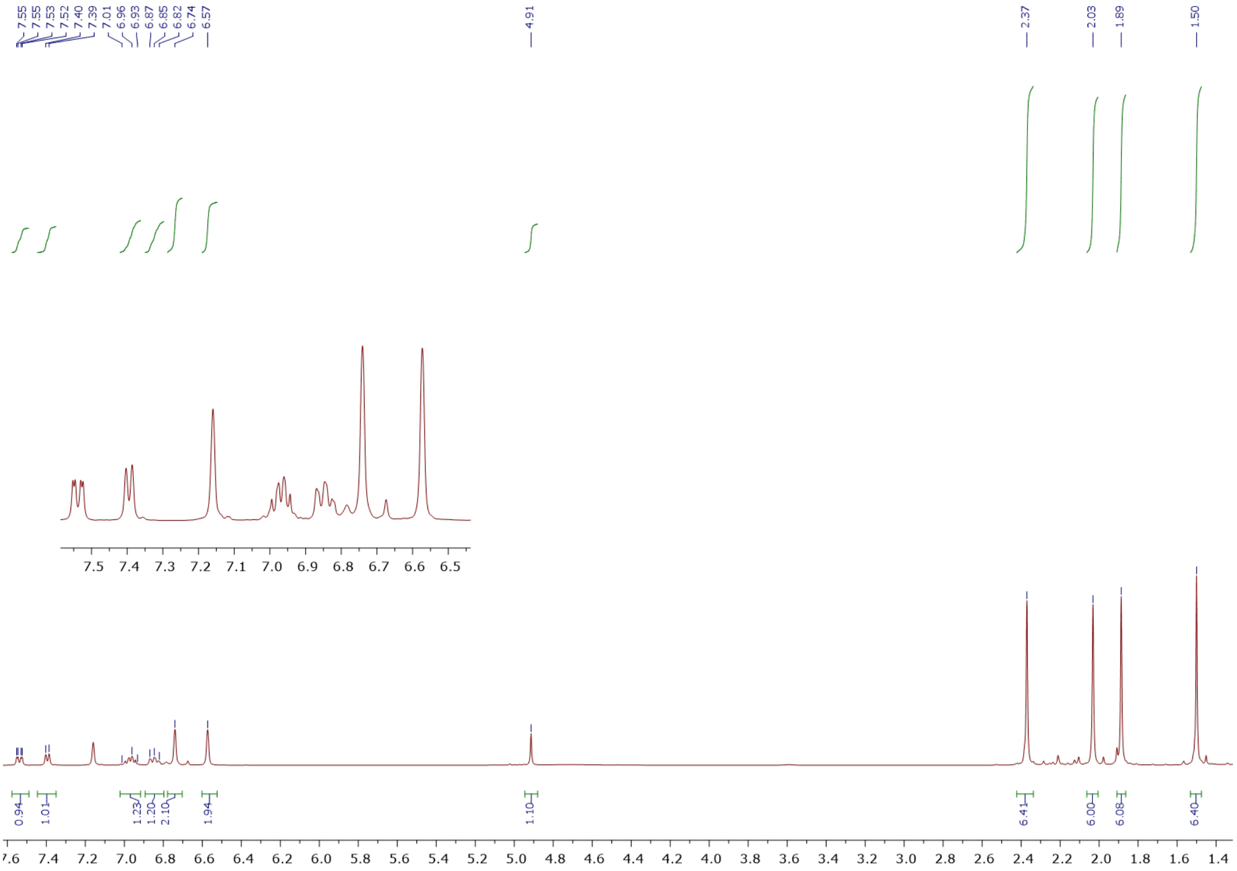

Figure S.3.40: ^{19}F NMR spectrum of complex **5c**

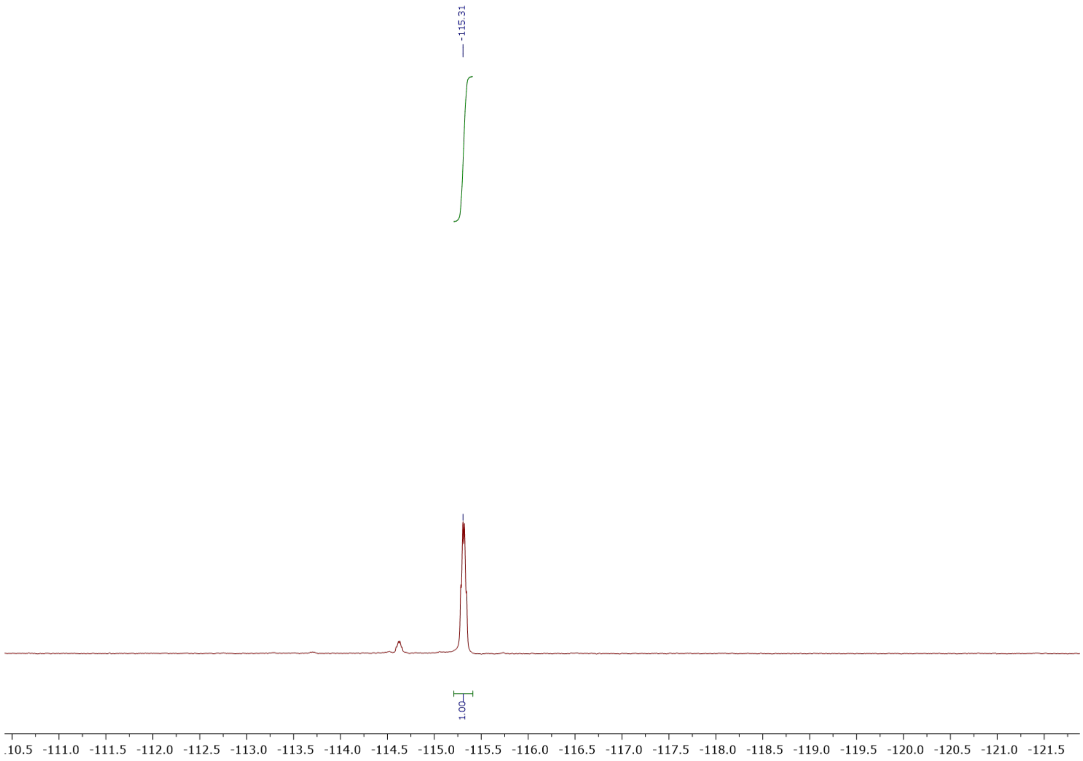

Figure S.3.41: ^{1}H NMR spectrum of complex **4c**

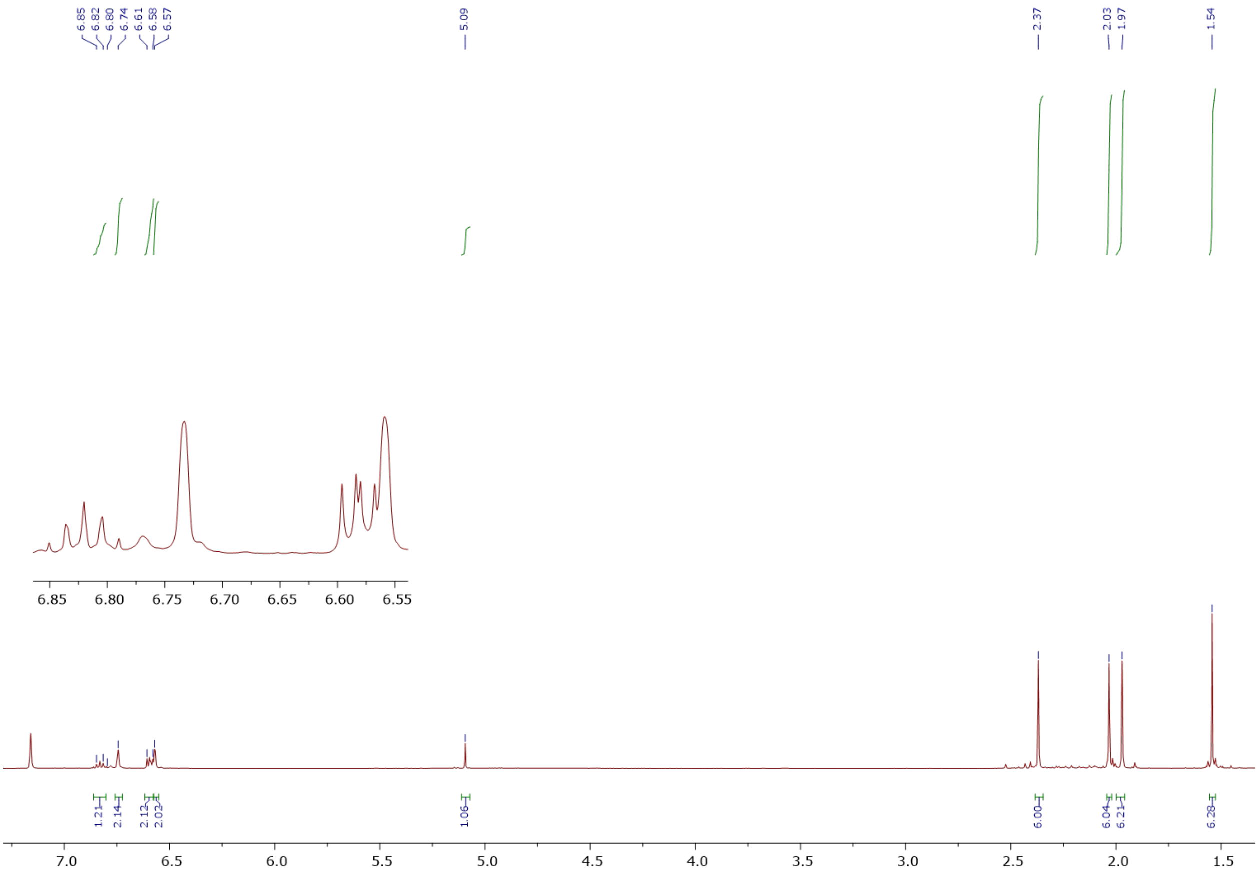

Figure S.3.42: ^{19}F{^{1}H} NMR spectrum of complex **4c**

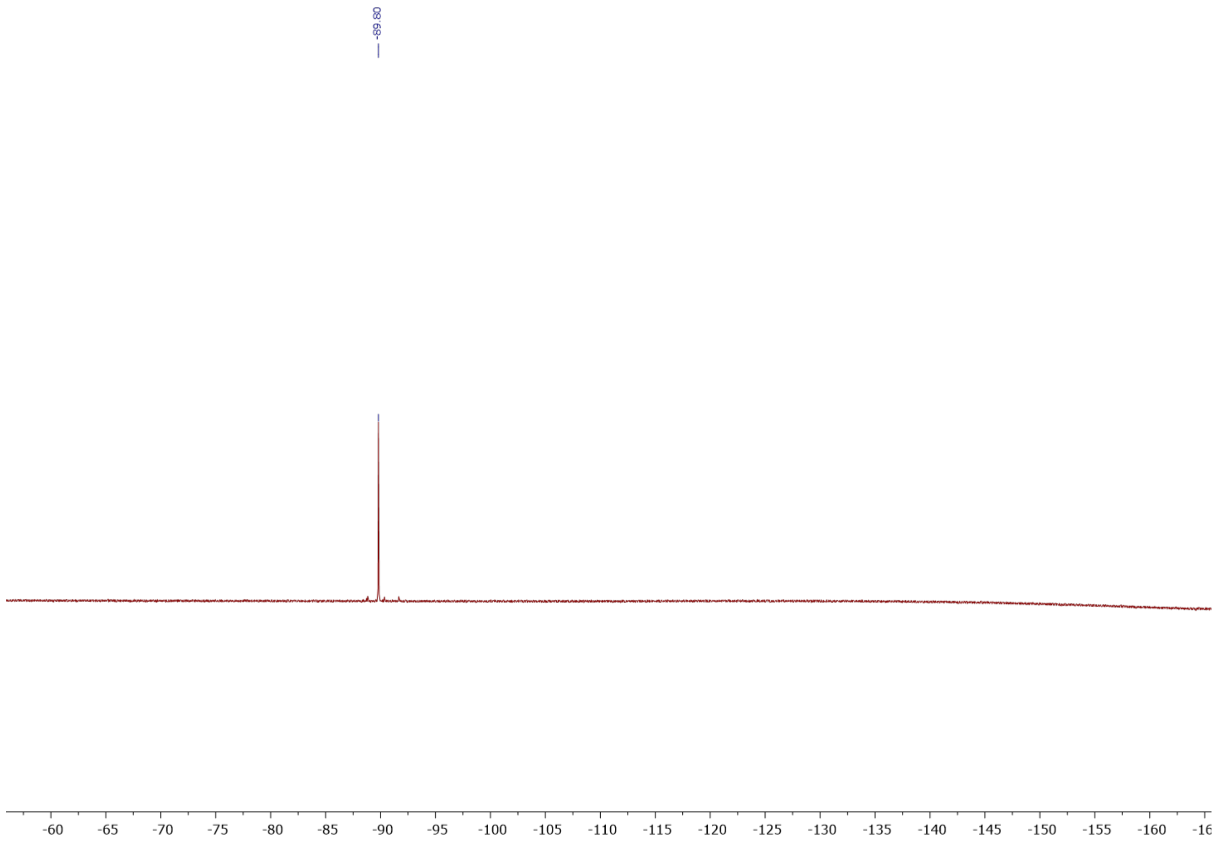

Figure S.3.43: $^{13}C\{^{1}H\}$ NMR spectrum of complex **4c** (* residual hexane)

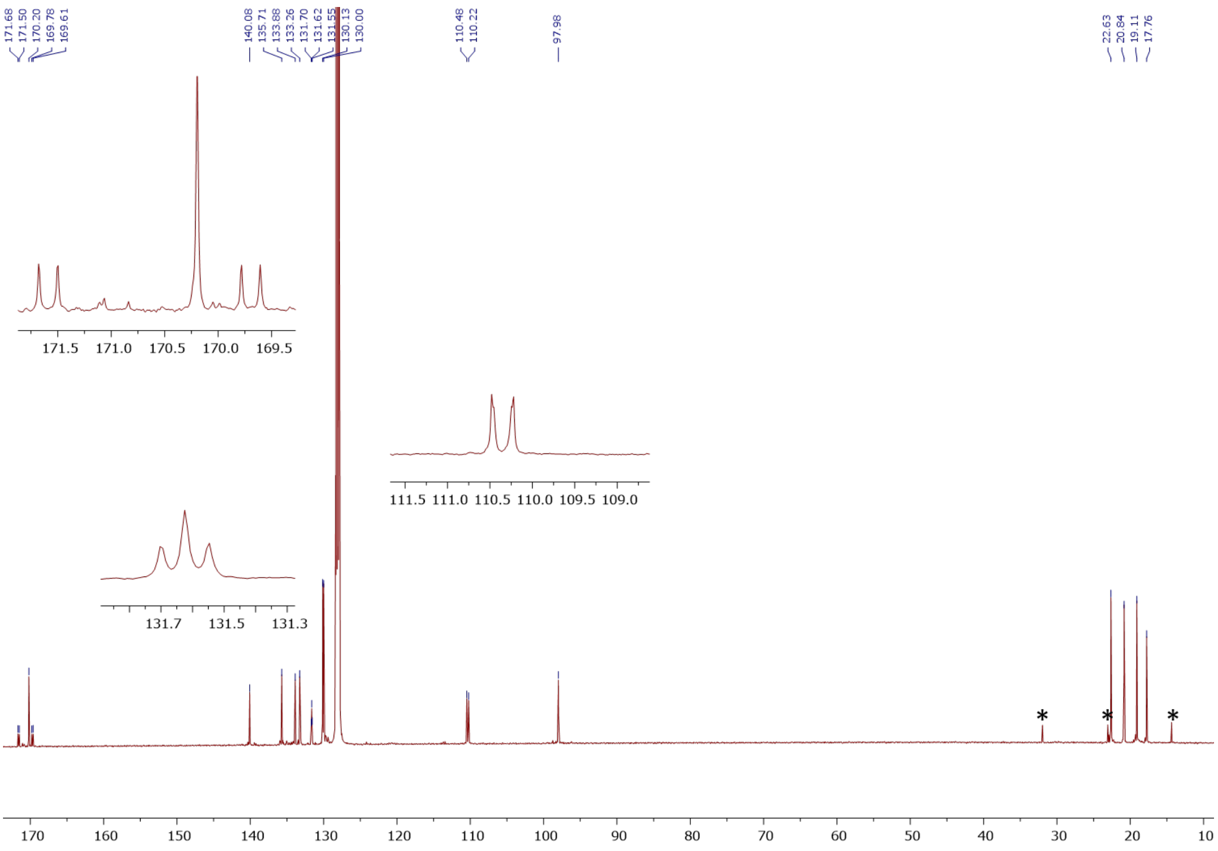

Figure S.3.44: ^{1}H NMR spectrum of complex **4e.**

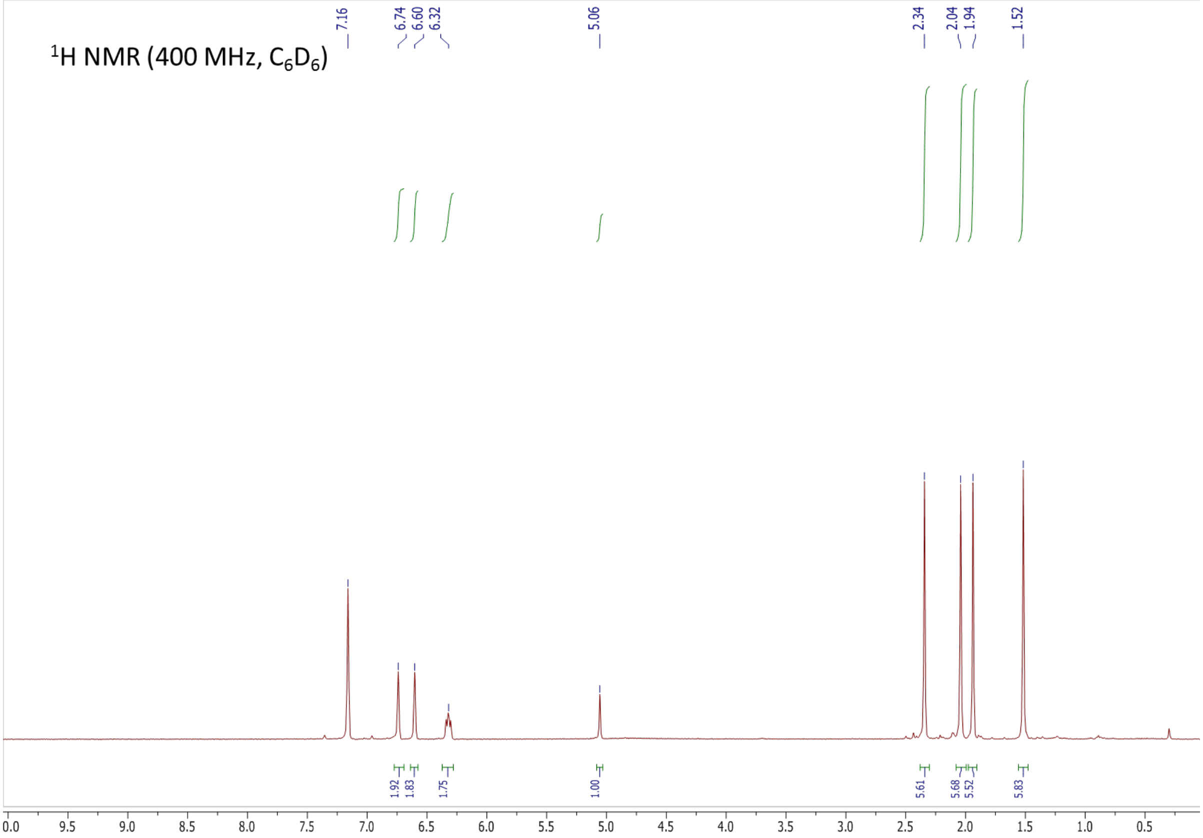

Figure S.3.45: ^{19}F NMR spectrum of complex **4e.**

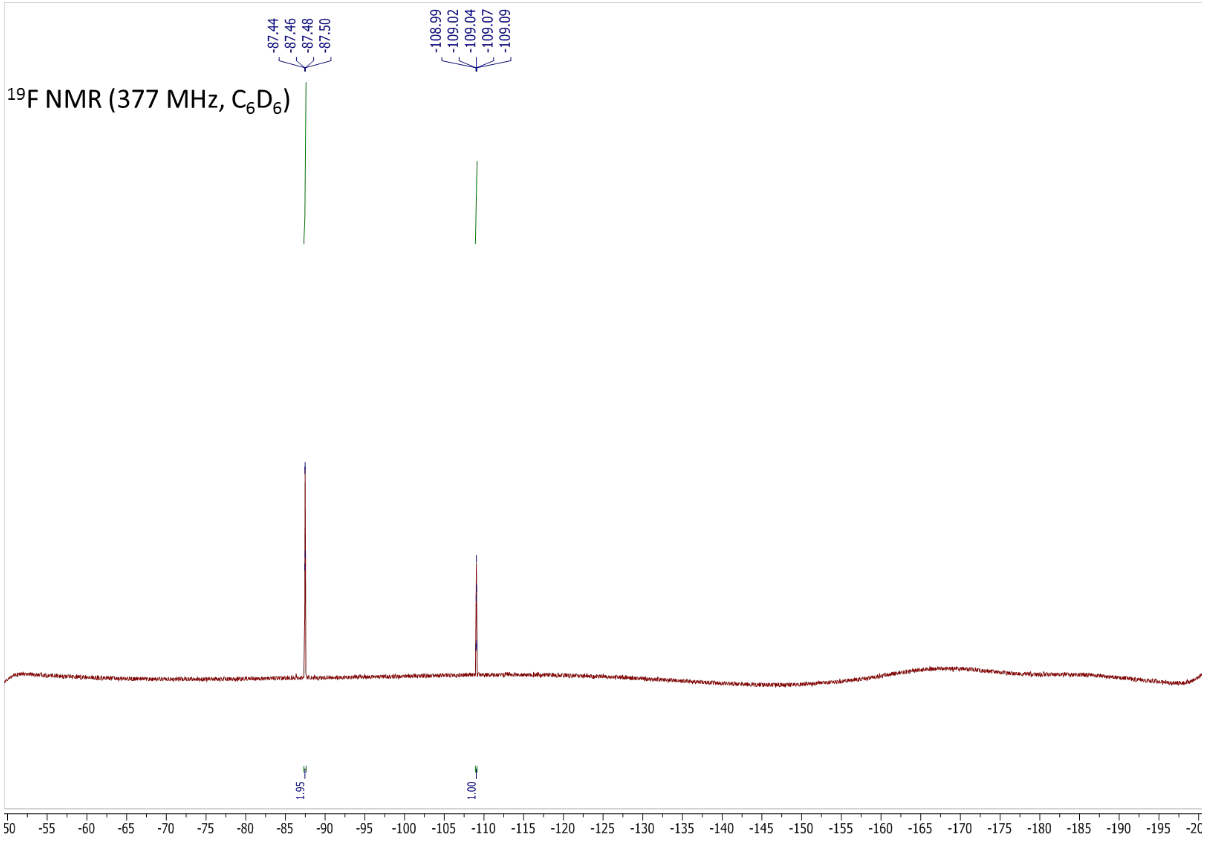

Figure S.3.46: ^{13}C NMR spectrum of complex **4e.**

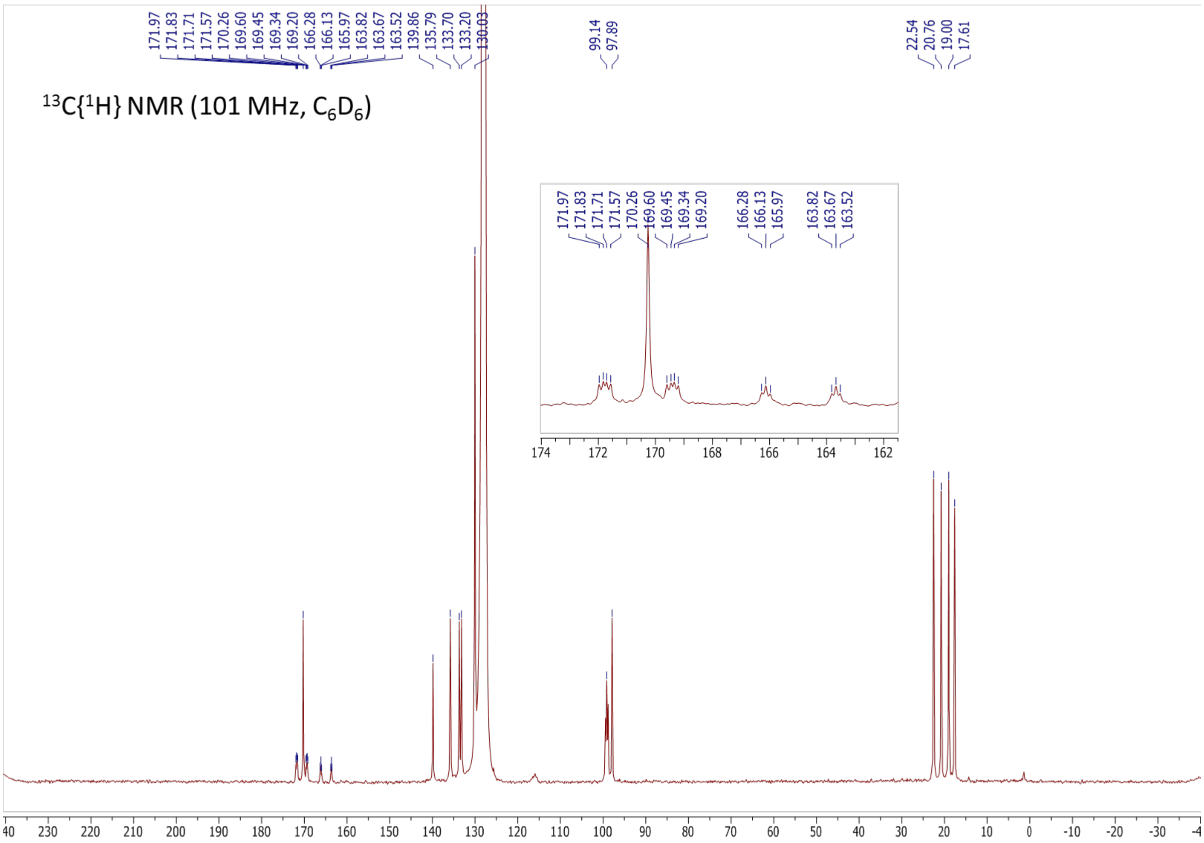

Figure S.3.47: ^{1}H NMR spectrum of complex **4f.**

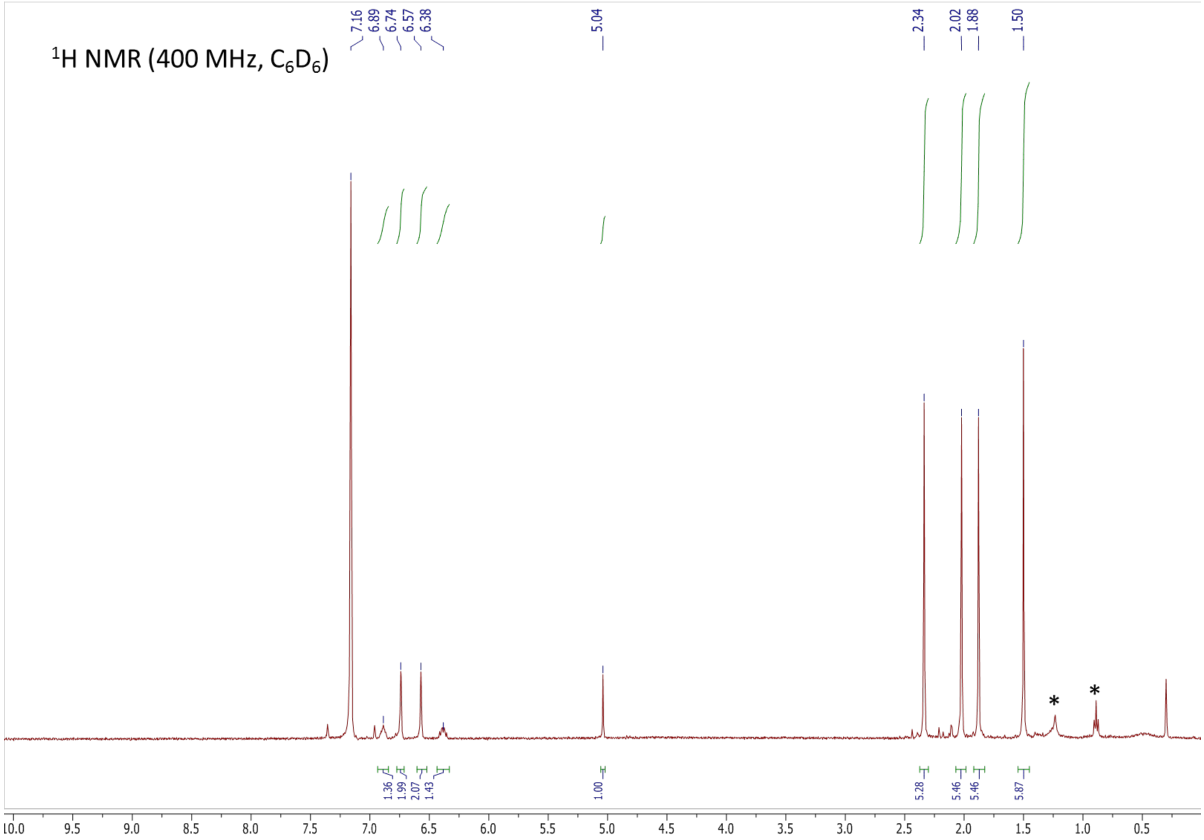

Figure S.3.48: ^{19}F NMR spectrum of complex **4f.**

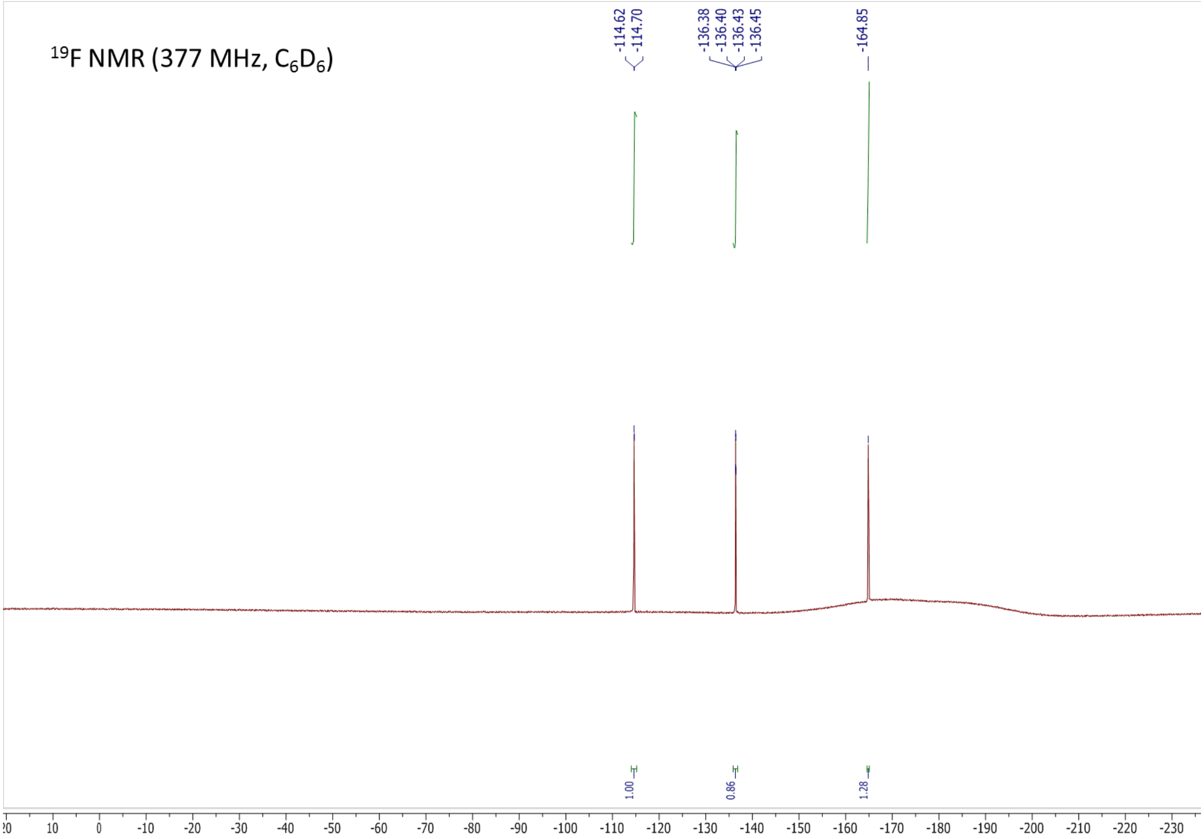

Figure S.3.49: ^{13}C NMR spectrum of complex **4f.**

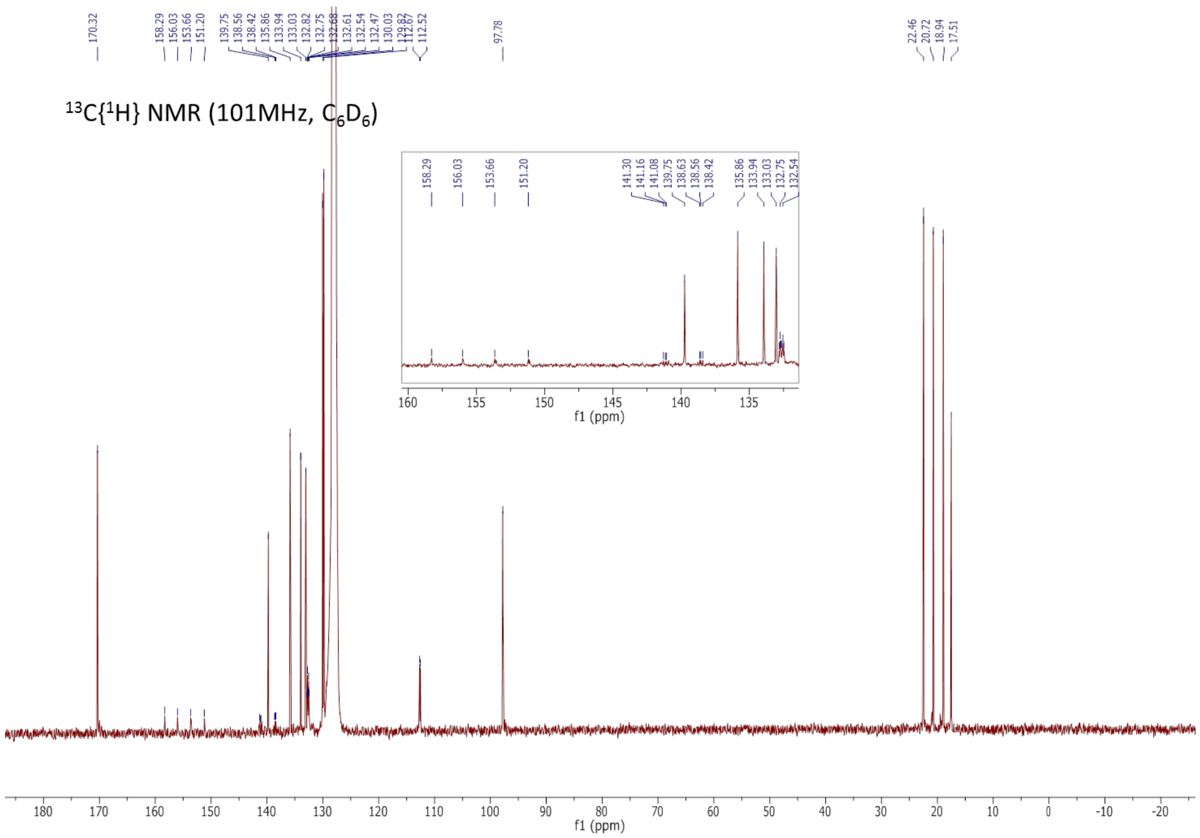

Figure S.3.50: ^{19}F NMR spectra following the isomerisation process of **4c** to **5c'** (* is α,α,α-trifluorotoluene internal standard)

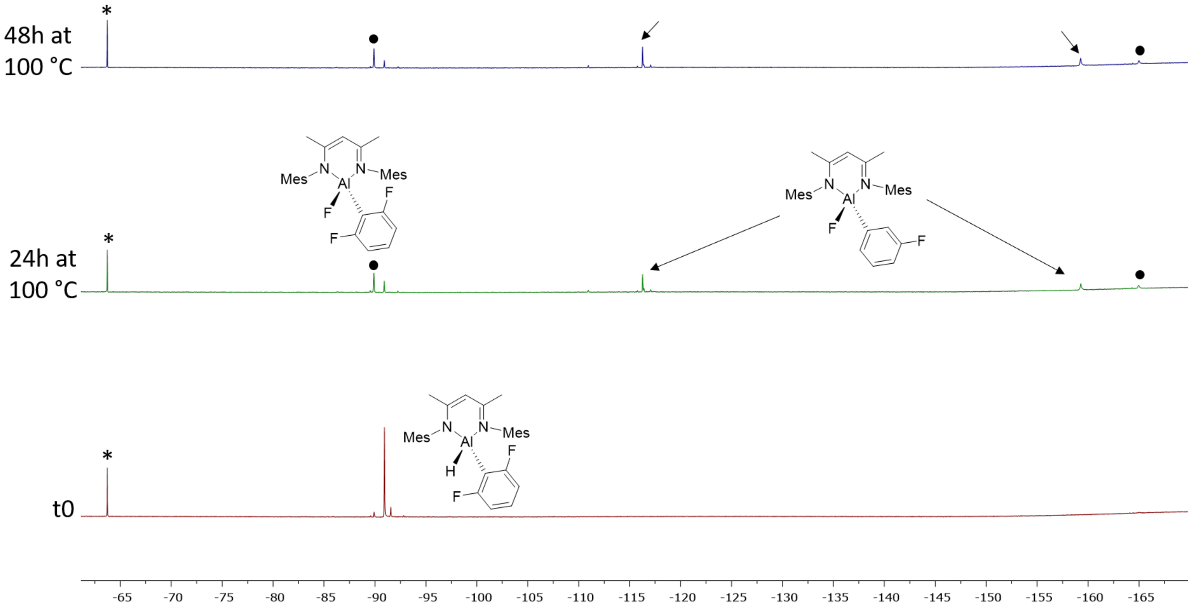

Figure S.3.51: ^{19}F NMR of the crude reaction mixture of isomerisation process with **4c** after 48h at 100 ºC (* is α,α,α-trifluorotoluene internal standard)

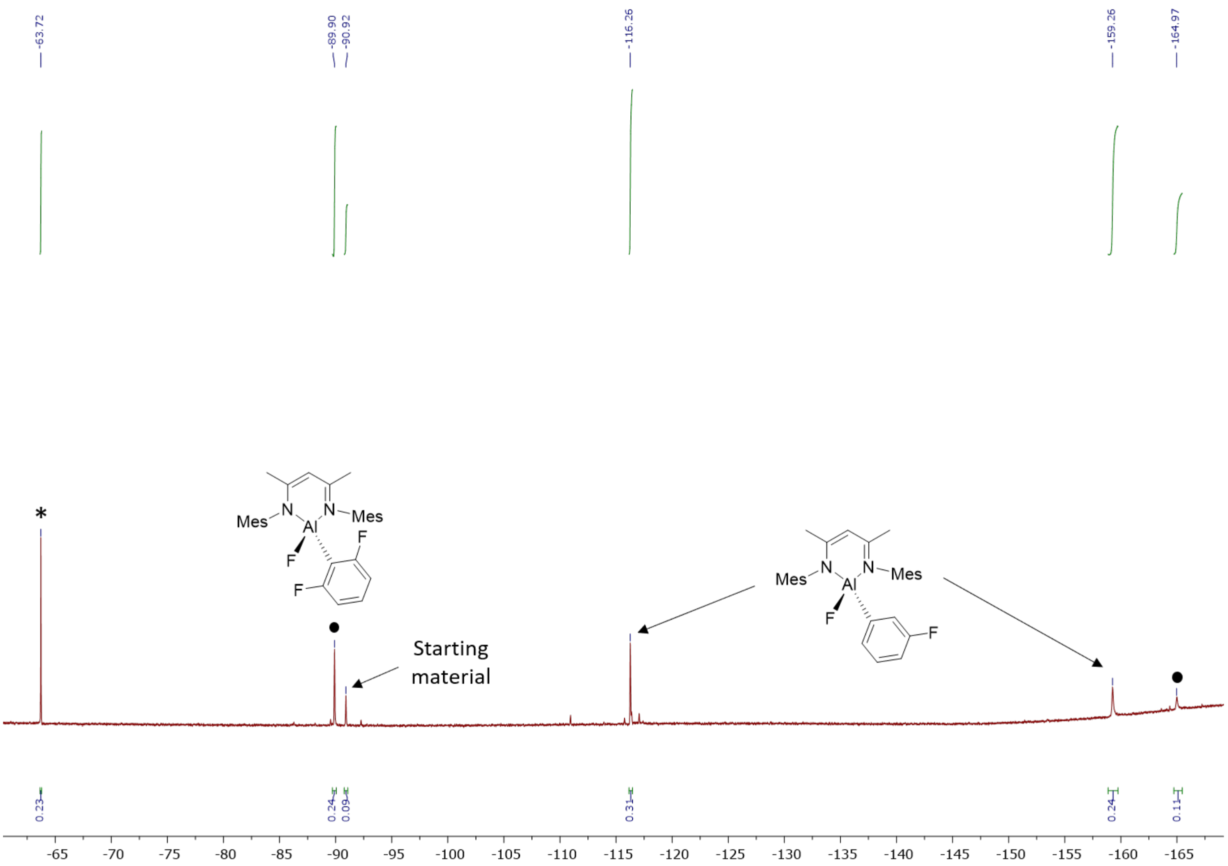

4- X-ray Crystallographic Data

4-1- Tabulated X-ray Data

data	2a	2b	2c	2e
formula	$C_{35}H_{46}AlFN_2$	$C_{35}H_{45}AlF_2N_2$	$C_{35}H_{45}AlF_2N_2$	$C_{35}H_{44}AlF_3N_2$
solvent	—	—	—	—
formula weight	540.72	558.71	558.71	576.70
colour, habit	colourless needles	colourless needles	colourless platy needles	colourless needles
temperature / K	173	173	173	173
crystal system	orthorhombic	orthorhombic	orthorhombic	monoclinic
space group	*Pnma* (no. 62)	*Ama*2 (no. 40)	*Pnma* (no. 62)	$P2_1/c$ (no. 14)
a / Å	16.6384(4)	22.3034(5)	16.6410(8)	21.4275(4)
b / Å	21.0131(6)	19.6726(5)	21.2084(7)	8.93859(19)
c / Å	9.0227(2)	7.40067(18)	8.9762(4)	16.6923(3)
α / deg	90	90	90	90
β / deg	90	90	90	92.9246(17)
γ / deg	90	90	90	90
V / Å^3	3154.55(14)	3247.16(13)	3167.9(2)	3192.93(11)
Z	4 [b]	4 [b]	4 [b]	4[b]
D_c / g cm^{-3}	1.139	1.143	1.171	1.200
radiation used	Cu-Kα	Cu-Kα	Mo-Kα	Cu-Kα
μ / mm^{-1}	0.791	0.830	0.101	0.906
2θ max / deg	147	147	56	147
no. of unique reflns measured (R_{int})	3135 (0.0427)	2260 (0.0224)	3254 (0.0328)	6088 (0.0282)
obs, $\|F_o\| > 4\sigma(\|F_o\|)$	2310	2038	2577	4568
no. of variables	200	201	209	402
R_1(obs), wR_2(all) [a]	0.0458, 0.1248	0.0355, 0.0936	0.0482, 0.1179	0.0464, 0.1260

Table S.4.1: Crystal Data, Data Collection and Refinement Parameters for the structures of **2a**, **2b**, **2c**, **2e**.

[a] $R_1 = \Sigma||F_o| - |F_c||/\Sigma|F_o|$; $wR_2 = \{\Sigma[w(F_o^2 - F_c^2)^2] / \Sigma[w(F_o^2)^2]\}^{1/2}$; $w^{-1} = \sigma^2(F_o^2) + (aP)^2 + bP$. [c] The molecule has crystallographic C_S symmetry.

data	2f	2g	5c/5c'
formula	$C_{35}H_{44}AlF_3N_2$	$C_{35}H_{44}AlF_3N_2$	$0.75(C_{29}H_{33}AlF_2N_2)$, $0.25(C_{29}H_{34}AlFN_2)$
solvent	$1.5(C_7H_8)$	—	—
formula weight	714.90	576.70	470.05
colour, habit	colourless tablets	colourless blocks	colourless blocks
temperature / K	173	173	173
crystal system	orthorhombic	monoclinic	orthorhombic
space group	*Aba*2 (no. 41)	$P2_1/c$ (no. 14)	*Pnma* (no. 62)
a / Å	25.1040(8)	12.2421(3)	14.5388(5)
b / Å	18.4374(8)	22.2792(4)	20.1047(6)
c / Å	17.7897(5)	12.5805(3)	8.9393(3)
α / deg	90	90	90
β / deg	90	109.551(3)	90
γ / deg	90	90	90
V / Å^3	8234.0(5)	3233.41(13)	2612.94(15)
Z	8 [b]	4 [b]	4 [b]
D_c / g cm^{-3}	1.153	1.185	1.195
radiation used	Mo-Kα	Cu-Kα	Cu-Kα
μ / mm^{-1}	0.095	0.895	0.929
2θ max / deg	57	147	148
no. of unique reflns measured (R_{int})	5630 (0.0205)	6180 (0.0269)	2615 (0.0381)
obs, $\|F_o\| > 4\sigma(\|F_o\|)$	4906	4810	2027
no. of variables	496	386	173
R_1(obs), wR_2(all) [a]	0.0353, 0.0850	0.0417, 0.1145	0.0549, 0.1609

Table S.4.1: Crystal Data, Data Collection and Refinement Parameters for the structures of **2f**, **2g** and **5c/5c'**.

[a] $R_1 = \Sigma||F_o| - |F_c||/\Sigma|F_o|$; $wR_2 = \{\Sigma[w(F_o^2 - F_c^2)^2] / \Sigma[w(F_o^2)^2]\}^{1/2}$; $w^{-1} = \sigma^2(F_o^2) + (aP)^2 + bP$. [b] The molecule has crystallographic C_S symmetry.

Table S.4.1 provides a summary of the crystallographic data for the structures of **2a**, **2b**, **2c**, **2e**, **2f**, **2g** and **5c/5c'**. Data were collected using Agilent Xcalibur 3 E (**2e** and **2f**) and Xcalibur PX Ultra A (**2a**, **2b**, **2c**, **2g** and **5c/5c'**) diffractometers, and the structures were refined using the SHELXTL and SHELX-2013 program systems.[4,5] The absolute structures of **2b** and **2f** were determined by use of the Flack parameter [**2b**: $x = +0.03(5)$, **2f**: $x = -0.01(10)$]. CCDC 1917502 to 1917508.

[4] SHELXTL v5.1, Bruker AXS, Madison, WI, 1998.
[5] SHELX-2013, G.M. Sheldrick, *Acta Cryst.*, 2015, **C71**, 3-8.

4-2- X-ray crystal structures

4-2-1- The X-ray crystal structure of 2a

The structure of **2a** was found to sit across a mirror plane that passes through C2, Al1, F1, C21 and C24. The C10-based isopropyl group was found to be disordered. Two orientations were identified of *ca*. 80 and 20% occupancy, their geometries were optimised, the thermal parameters of adjacent atoms were restrained to be similar, and only the non-hydrogen atoms of the major occupancy orientation were refined anisotropically (those of the minor occupancy orientation were refined isotropically).

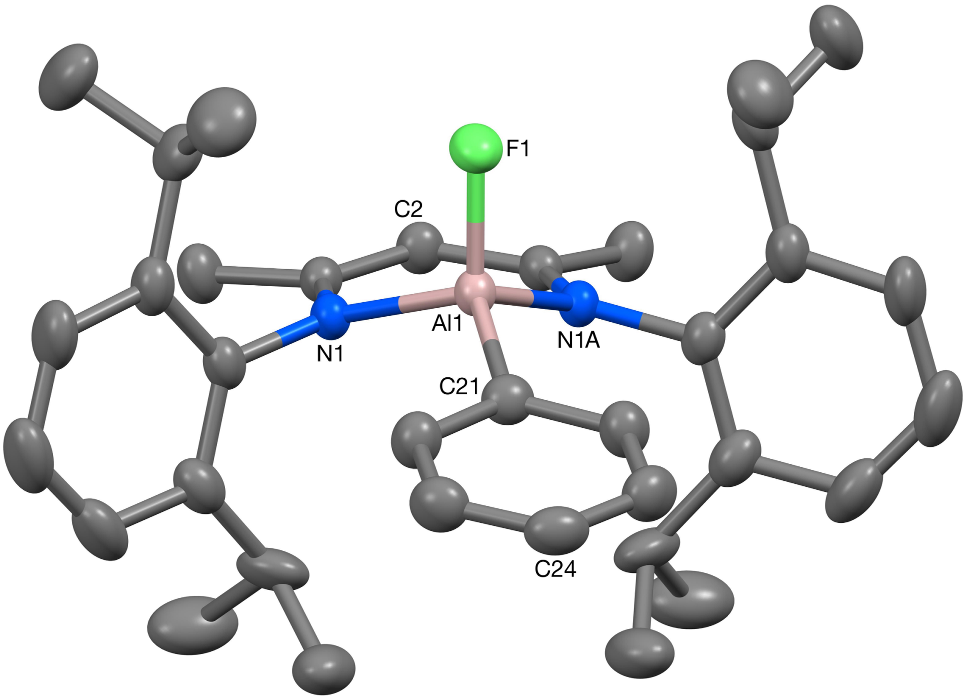

Fig. S.4.1: The crystal structure of the C_S symmetric complex **2a** (50% probability ellipsoids). Hydrogen atoms omitted for clarity.

4-2-2- The X-ray crystal structure of 2b

The structure of **2b** was found to sit across a mirror plane that passes through C2, Al1, F1, and the whole of the C21-based C_6H_4F group.

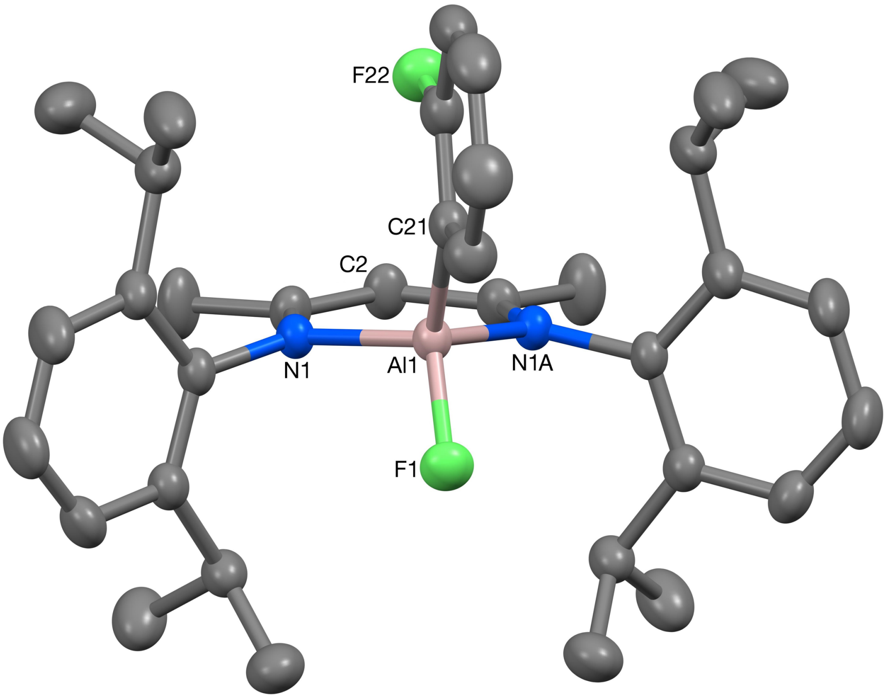

Fig. S.4.2: The crystal structure of the C_S symmetric complex **2b** (50% probability ellipsoids). Hydrogen atoms omitted for clarity.

4-2-3- The X-ray crystal structure of 2c

The structure of **2c** was found to sit across a mirror plane that passes through C2, Al1, F1, C21 and C24. When refined at full occupancy, the thermal parameter of F23 was clearly substantially too large compared to the rest of the structure. Allowed to refine freely, the occupancy settled at *ca*. 49%, and this was then fixed at 50% for simplicity and the atom was refined anisotropically. Consequently the structure can be interpreted as the expected 3-monofluoro species disordered across the mirror plane (though a 50:50 mixture of the phenyl and 3,5-difluoro species would give the same average, as would appropriate combinations of all three molecules).

The C10-based isopropyl group was found to be disordered. Two orientations were identified of *ca*. 71 and 29% occupancy, their geometries were optimised, the thermal parameters of adjacent atoms were restrained to be similar, and only the non-hydrogen atoms of the major occupancy orientation were refined anisotropically (those of the minor occupancy orientation were refined isotropically).

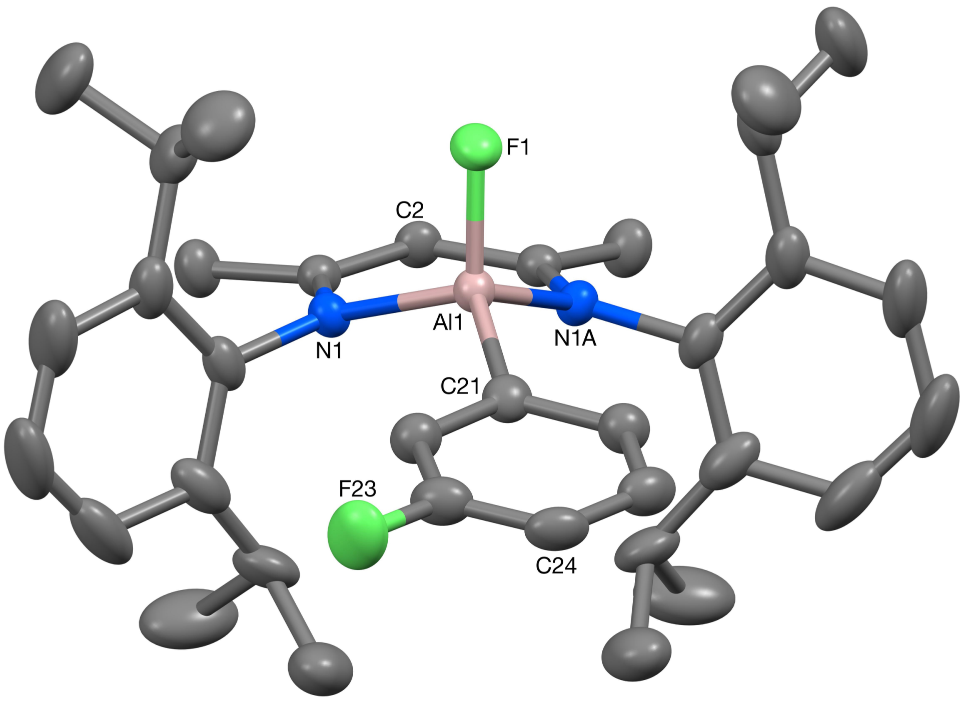

Fig. S.4.3: The crystal structure of the C_S symmetric complex **2c** (50% probability ellipsoids). Hydrogen atoms omitted for clarity.

4-2-4- The X-ray crystal structure of 2e

The C12- and C27-based isopropyl groups in the structure of 2e were both found to be disordered, and two orientations were identified in each case, of ca. 73:27 and 65:35% occupancy respectively. The geometries of each pair of orientations were optimised, the thermal parameters of adjacent atoms were restrained to be similar, and only the non-hydrogen atoms of the major occupancy orientations were refined anisotropically (those of the minor occupancy orientations were refined isotropically).

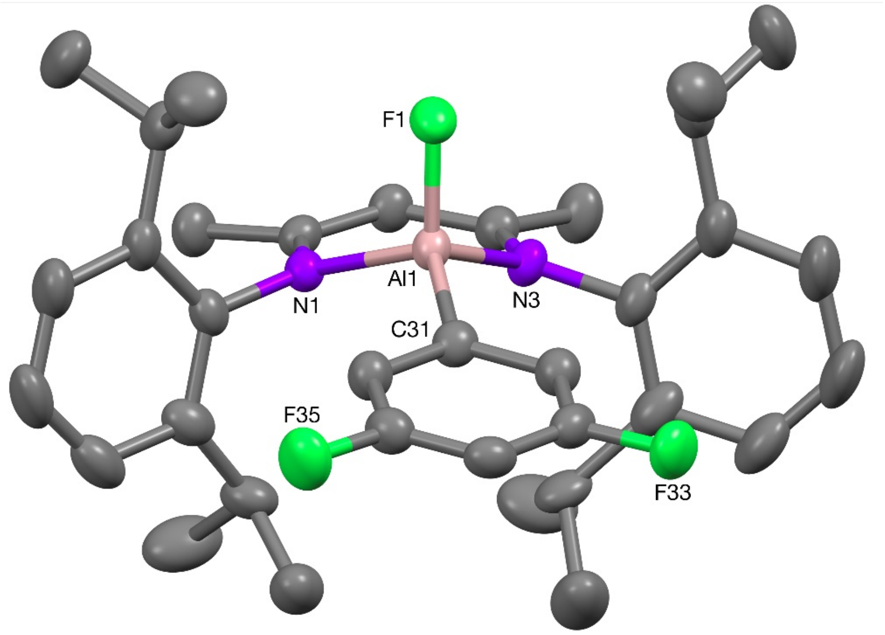

Fig. S.4.4: The crystal structure of **2e** (50% probability ellipsoids). Hydrogen atoms omitted for clarity.

4-2-5- The X-ray crystal structure of 2f

The C50-based included toluene solvent molecule in the structure of **2f** was found to be disordered across a C_2 axis. This was modelled using one complete, 50% occupancy, orientation (with a second 50% occupancy orientation being generated by operation of the C_2 axis). The geometry of the unique orientation was optimised, the thermal parameters of adjacent atoms were restrained to be similar, and all of the non-hydrogen atoms were refined anisotropically.

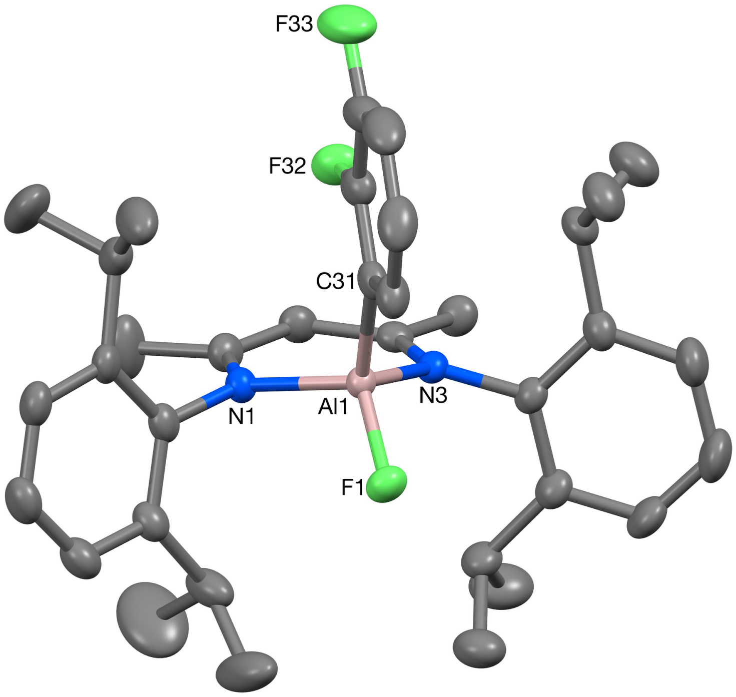

Fig. S.4.5: The crystal structure of **2f** (50% probability ellipsoids). Hydrogen atoms omitted for clarity.

4-2-6- The X-ray crystal structure of 2g

When refined at full occupancy, the thermal parameter for the F35 fluorine atom in the structure of **2g** was found to be too large compared to the rest of the structure, and when allowed to refine freely the occupancy of this atom settled at *ca*. 91%. Additionally, a small but noticeable electron density peak (*ca*. 1.35 eÅ^{-3} *cf*. 0.36 eÅ^{-3} for the next largest peak) was found near C34, in approximately the right place for a fluorine atom, suggesting the co-crystallisation of two different isomers. The crystal was thus modelled as a mixture of the 2,5– and 2,4-difluoro species (in a *ca*. 91:9 ratio) with the thermal parameters of F35 and F34' restrained to be similar, and with only the major occupancy atom (F35) refined anisotropically (F34' was refined isotropically).

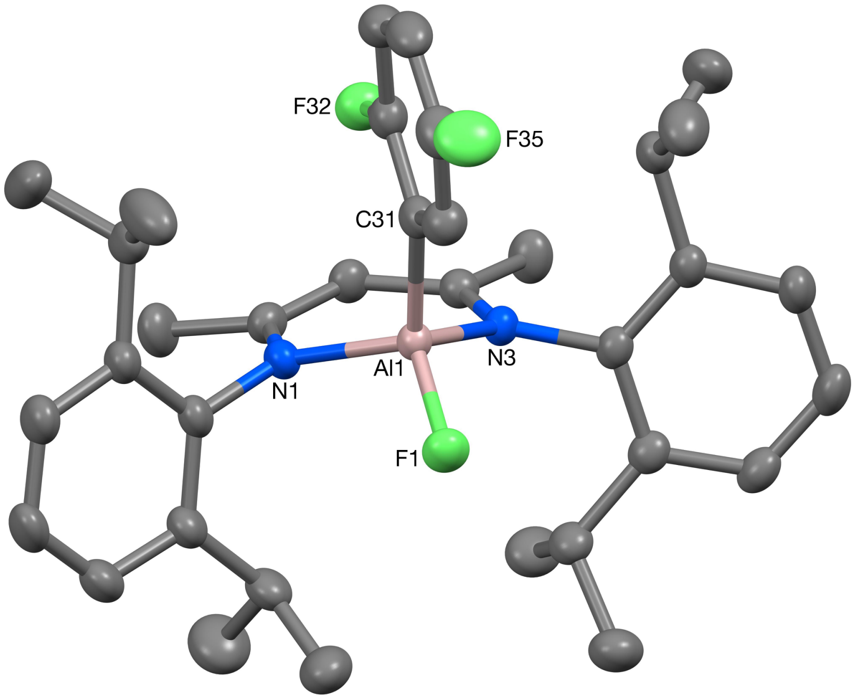

Fig. S.4.6: The crystal structure of **2g** (50% probability ellipsoids). Hydrogen atoms omitted for clarity.

4-2-7- The X-ray crystal structure of 5c/c'

The structure of **5c/5c'** was found to sit across a mirror plane that passes through C2, Al1, F1, and the whole of the C21-based C_6H_4F group. When refined at full occupancy, the thermal parameter of F1 was clearly too large compared to the rest of the structure. Allowed to refine freely, the occupancy settled at *ca*. 76%, and this was then fixed at 75% for simplicity. As such, the structure has been interpreted as a *ca*. 75:25 mixture of the Al–F and Al–H species, with the presumed 25% occupancy hydride not located. As a result, the atom list for the asymmetric unit is low by 0.125H, and that for the unit cell low by 1H.

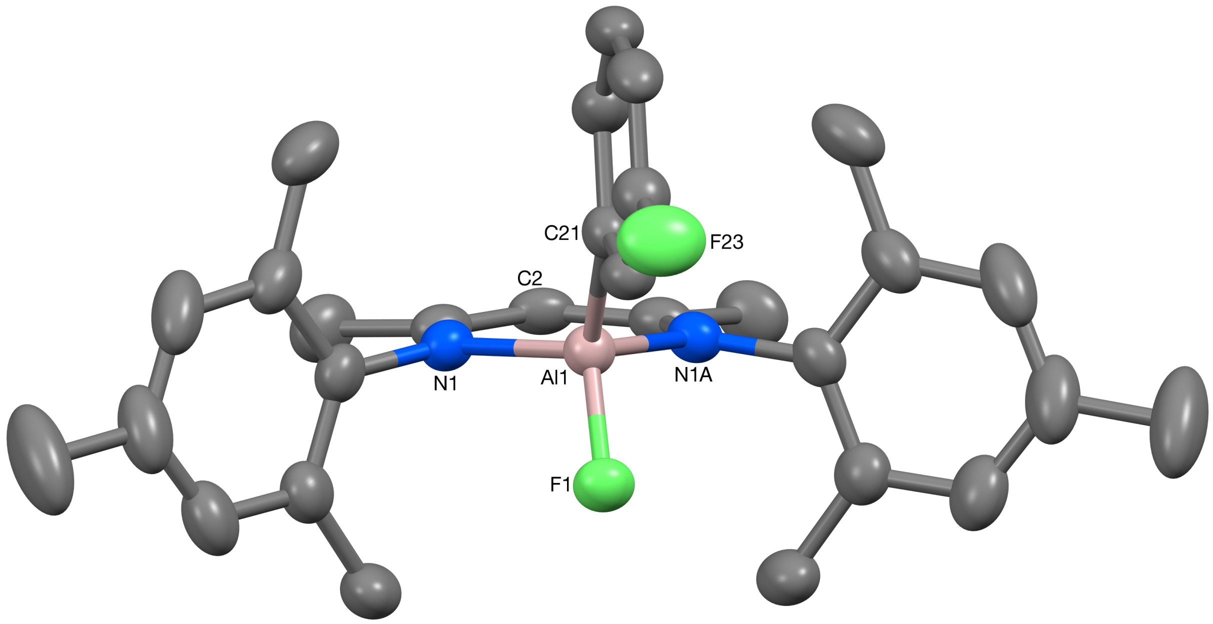

Fig. S.4.8: The crystal structure of the C_S symmetric complex **5c/5c'** (50% probability ellipsoids). Hydrogen atoms omitted for clarity.

5- Computational details

4-3- Methods

The geometries of products were optimised with the M06L Minnesota DFT functional using the Gaussian09 program package.[6] Stationary points were characterised depending on their imaginary frequencies (0 for minima and 1 for TSs). NBO analysis was performed using the NBO 6.0 version program.[7] The ωB97x hybrid exchange-correlation DFT functional and the B3PW91 functional were also employed to assess differences in performance arising from the level of theory.

The SDD effective core potential was used for all metals (SDDAll). The split-valence 6-31G** basis set was used for C, H, N, P and O atoms. The default numerical integration grid was also improved using a pruned grid with 99 radial shells and 590 angular points per shell (int=ultrafine).

Dispersion effects were included *via* single point energy corrections and were modelled using ωB97xD functional for ωB97x, using Grimme's D3 correction for M06L (EmpiricalDispersion=GD3)[8]

Solvent effects were included *via* single point energy corrections (benzene, $\varepsilon = 2.2706$) and were modelled using the polarizable continuum model (PCM) to free energies for M06L.

Intrinsic Reaction Coordinate (IRC) calculations were used to connect transition states and minima located on the potential energy surface to give a full potential energy profile.

---

[6] Frisch, M. J.; Trucks, G. W.; Schlegel, H. B.; Scuseria, G. E.; Robb, M. A.; Cheeseman, J. R.; Scalmani, G.; Barone, V.; Mennucci, B.; Petersson, G. A.; Nakatsuji, H.; Caricato, M.; Li, X.; Hratchian, H. P.; Izmaylov, A. F.; Bloino, J.; Zheng, G.; Sonnenberg, J. L.; Hada, M.; Ehara, M.; Toyota, K.; Fukuda, R.; Hasegawa, J.; Ishida, M.; Nakajima, T.; Honda, Y.; Kitao, O.; Nakai, H.; Vreven, T.; Montgomery, J. A., Jr.; Peralta, J. E.; Ogliaro, F.; Bearpark, M.; Heyd, J. J.; Brothers, E.; Kudin, K. N.; Staroverov, V. N.; Kobayashi, R.; Normand, J.; Raghavachari, K.; Rendell, A.; Burant, J. C.; Iyengar, S. S.; Tomasi, J.; Cossi, M.; Rega, N.; Millam, J. M.; Klene, M.; Knox, J. E.; Cross, J. B.; Bakken, V.; Adamo, C.; Jaramillo, J.; Gomperts, R.; Stratmann, R. E.; Yazyev, O.; Austin, A. J.; Cammi, R.; Pomelli, C.; Ochterski, J. W.; Martin, R. L.; Morokuma, K.; Zakrzewski, V. G.; Voth, G. A.; Salvador, P.; Dannenberg, J. J.; Dapprich, S.; Daniels, A. D.; Farkas, Ö.; Foresman, J. B.; Ortiz, J. V.; Cioslowski, J.; Fox, D. J. *Gaussian 09, Revision D.01*; Gaussian, Inc., Wallingford, CT, 2009.

[7] *NBO 6.0*. Glendening, E. D.; Badenhoop, J. K.; Reed, A. E.; Carpenter, J. E.; Bohmann, J. A.; Morales, C. M.; Landis, C. R.; Weinhold, F. Theoretical Chemistry Institute, University of Wisconsin, Madison (2013).

[8] Grimme, S.; Antony, J.; Ehrlich, S.; Krieg, H. *J Chem. Phys.* 2010, **132**, 154104.

5-2- <u>C–H and C–F alumination of Difluorobenzene: Mechanim</u>

5-2-1- Key geometrical parameters of intermediates, Int-1 – Int-9

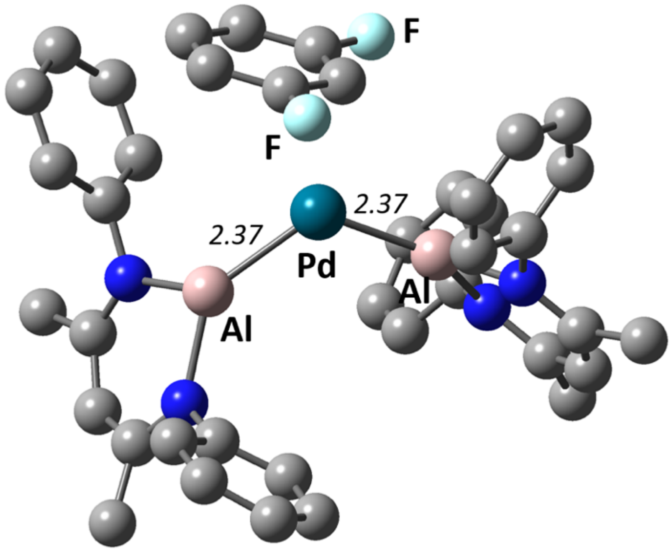

Fig. S.5.1: Selected bond lengths (in Å) for **Int-1**. *i*-Pr groups and some hydrogens have been omitted for clarity.

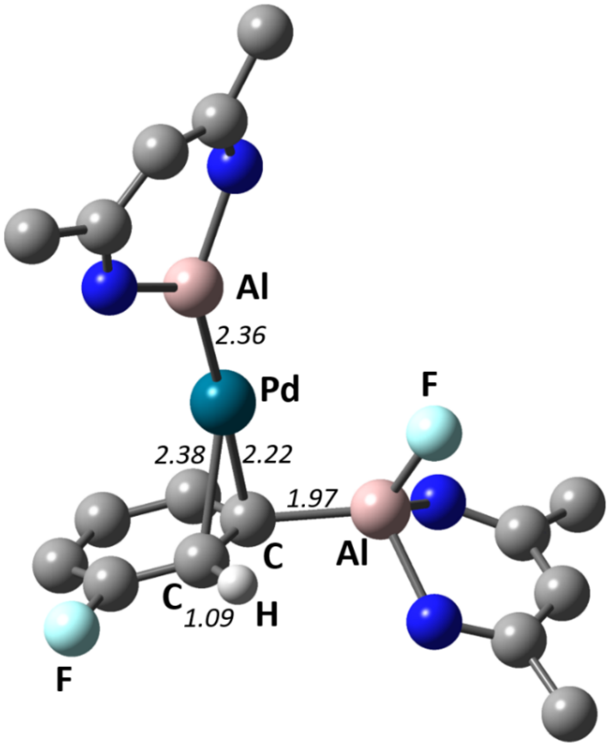

Fig. S.5.2: Selected bond lengths (in Å) for **Int-2**. 2,6-Diisopropylphenyl groups and some hydrogens have been omitted for clarity.

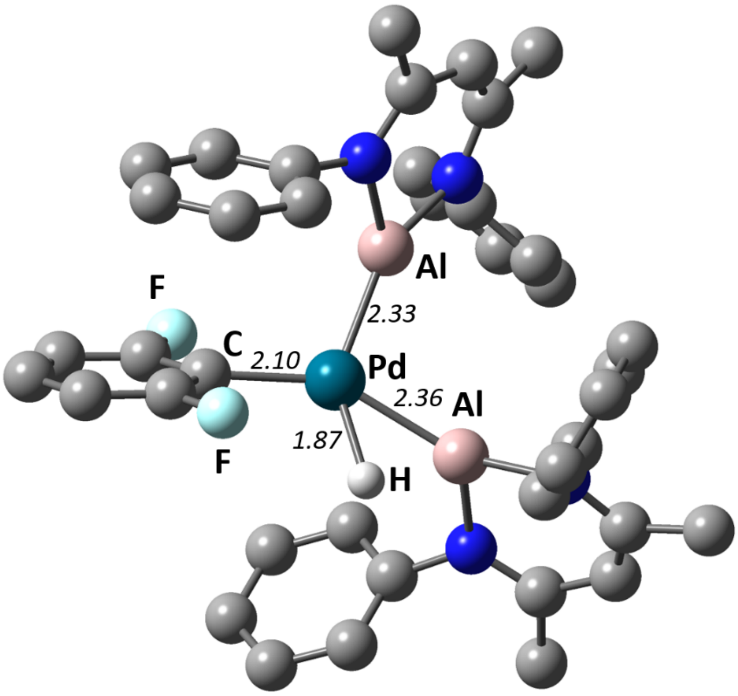

Fig. S.5.3: Selected bond lengths (in Å) for **Int-3**. *i*-Pr groups and some hydrogens have been omitted for clarity.

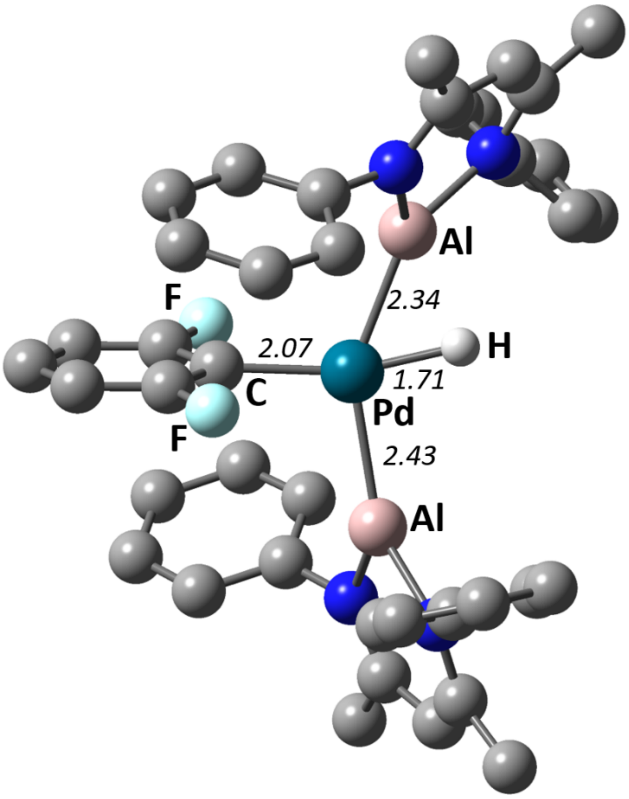

Fig. S.5.4: Selected bond lengths (in Å) for **Int-4**. *i*-Pr groups and some hydrogens have been omitted for clarity.

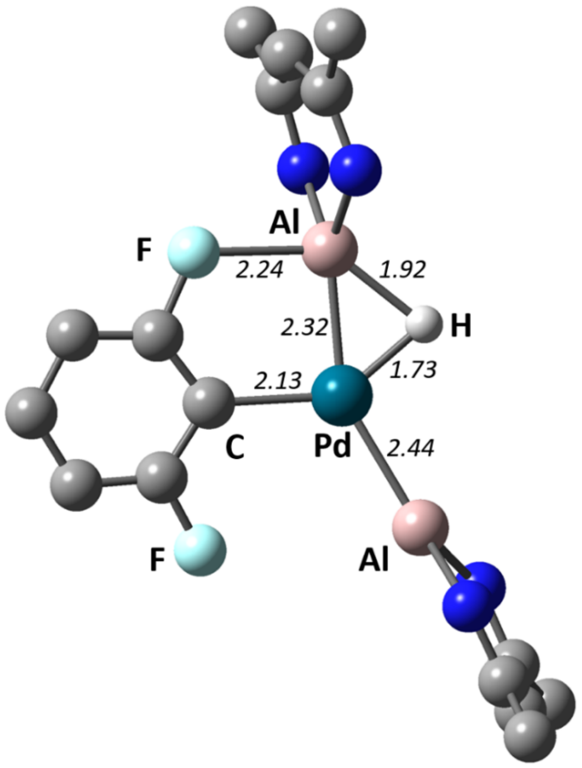

Fig. S.5.5: Selected bond lengths (in Å) for **Int-5**. 2,6-Diisopropylphenyl groups and some hydrogens have been omitted for clarity.

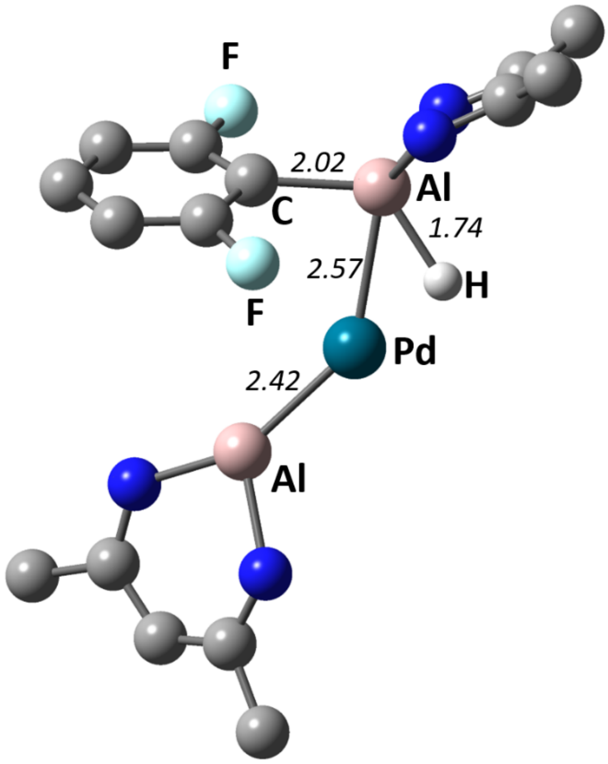

Fig. S.5.6: Selected bond lengths (in Å) for **Int-6**. 2,6-Diisopropylphenyl groups and some hydrogens have been omitted for clarity.

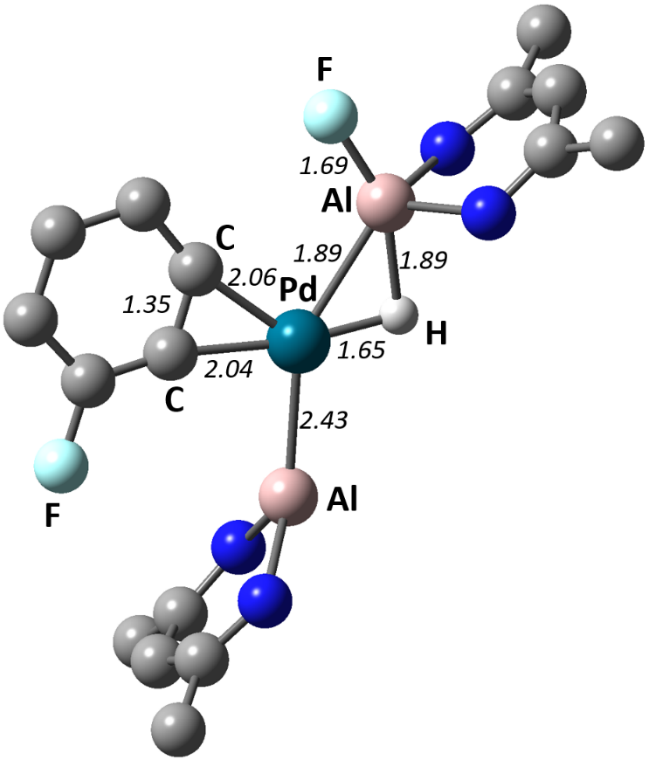

Fig. S.5.7: Selected bond lengths (in Å) for **Int-7**. 2,6-diisopropylphenyl groups and some hydrogens have been omitted for clarity.

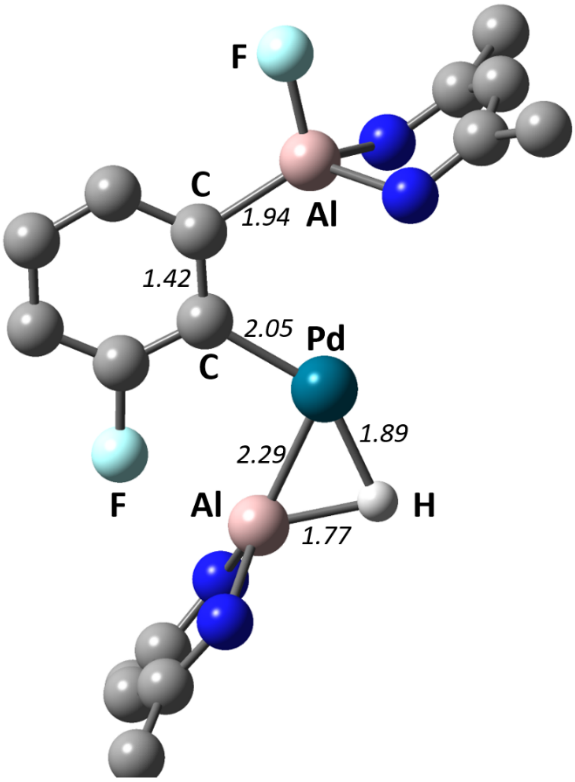

Fig. S.5.8: Selected bond lengths (in Å) for **Int-8**. 2,6-diisopropylphenyl groups and some hydrogens have been omitted for clarity.

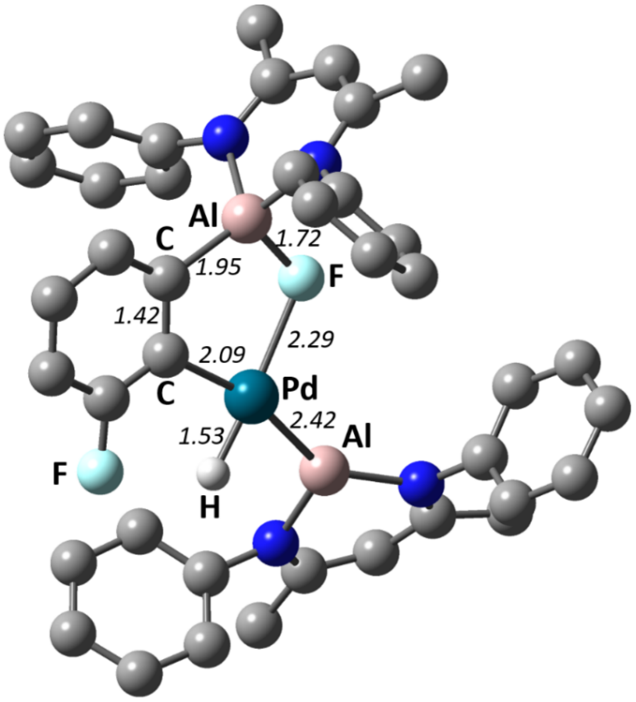

Fig. S.5.9: Selected bond lengths (in Å) for **Int-9**. *i*-Pr groups groups and some hydrogens have been omitted for clarity.

Int-S2 (given below) is a conformer of **Int-2** which is slightly higher in energy compared to **Int-2** ($\Delta\Delta G^{\ddagger} = +6.8$ kcal mol^{-1} less stable for **Int-S2**). **Int-S2** was obtained from the optimization of the minimum located on the potential energy surface of the IRC of **TS-1** whereas **Int-2** results from the optimization of the minimum located on the potential energy surface of the IRC of **TS-7**.

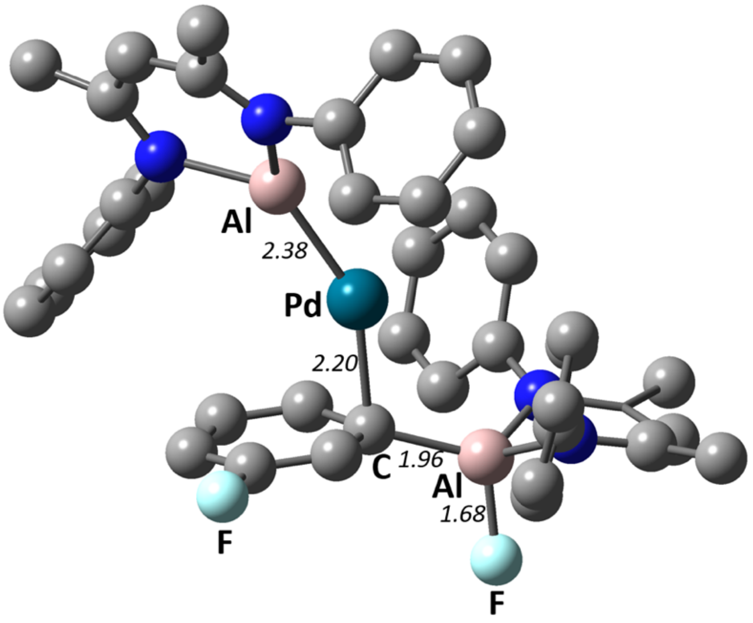

Fig. S.5.2: Selected bond lengths (in Å) for **Int-S2**. *i*-Pr groups and some hydrogens have been omitted

5-2-2- Key geometrical parameters of transition states, TS-1 – TS-7

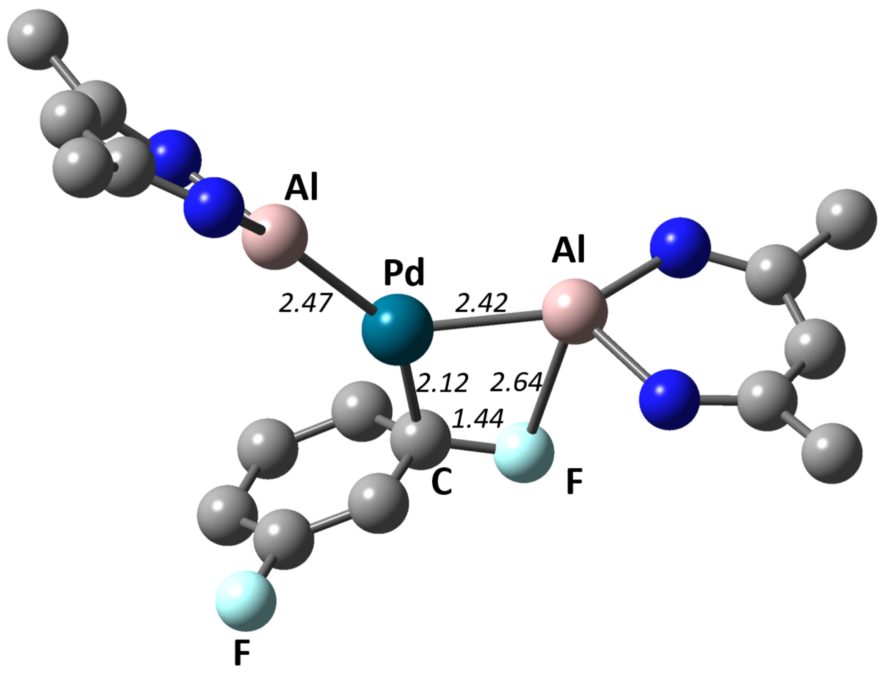

Fig. S.5.11: Selected bond lengths (in Å) for **TS-1**. 2,6-diisopropylphenyl groups and some hydrogens have been omitted for clarity.

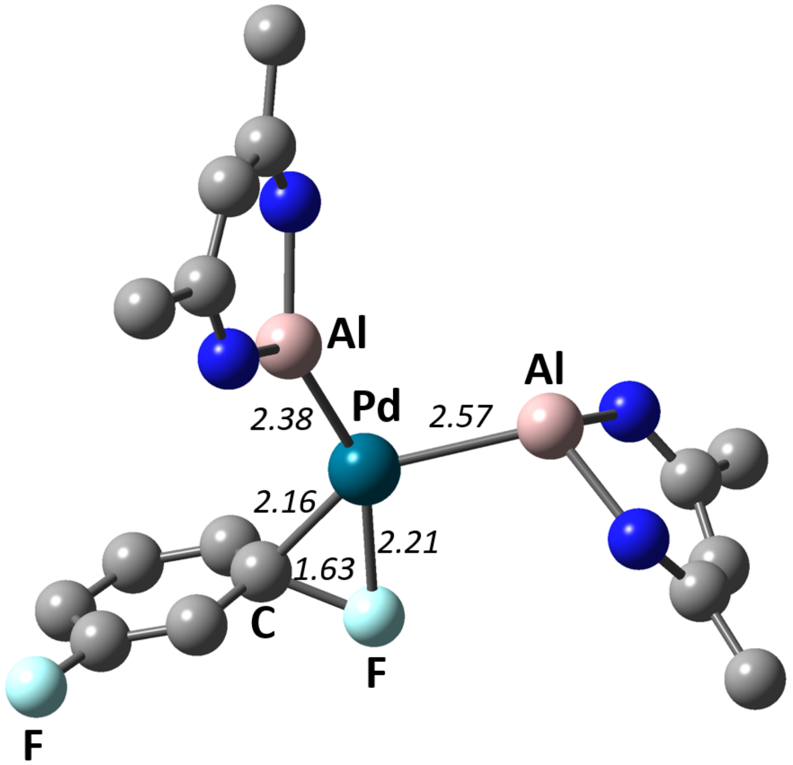

Fig. S.5.12: Selected bond lengths (in Å) for **TS-2**. 2,6-diisopropylphenyl groups and some hydrogens have been omitted for clarity.

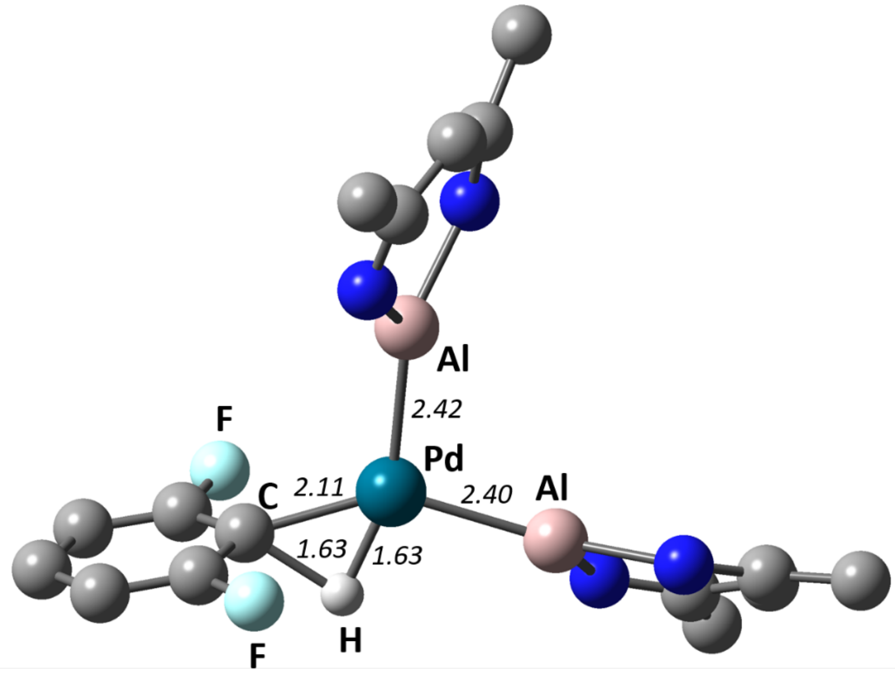

Fig. S.5.13: Selected bond lengths (in Å) for **TS-3**. 2,6-diisopropylphenyl groups and some hydrogens have been omitted for clarity.

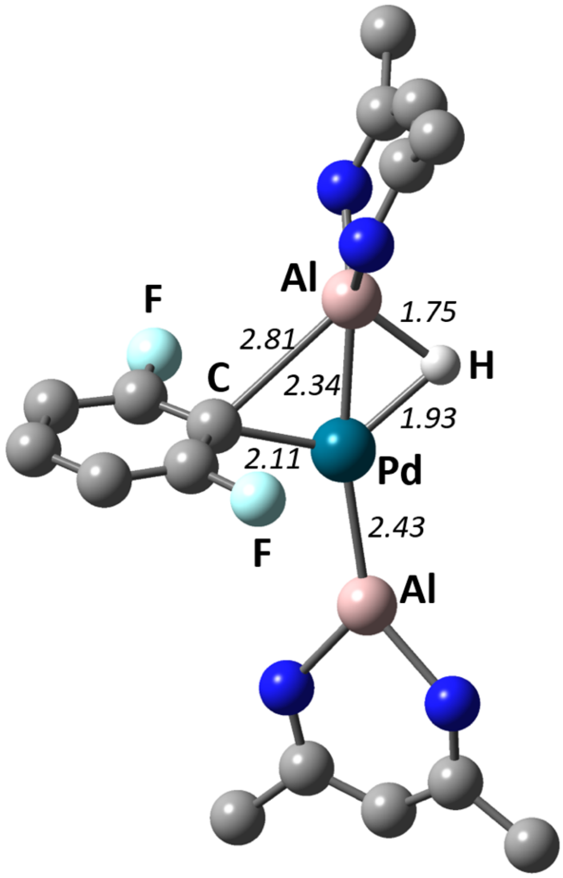

Fig. S.5.14: Selected bond lengths (in Å) for **TS-4**. 2,6-diisopropylphenyl groups and some hydrogens have been omitted for clarity.

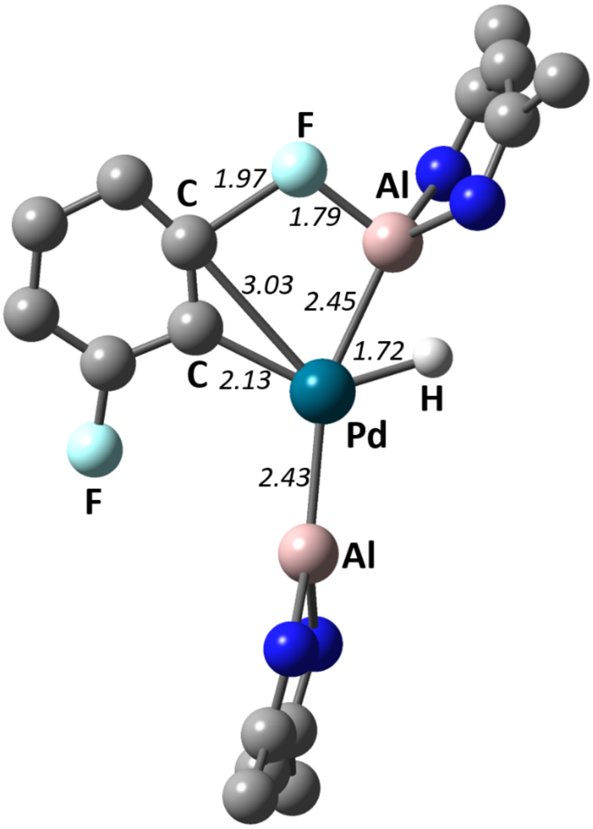

Fig. S.5.15: Selected bond lengths (in Å) for **TS-5**. 2,6-diisopropylphenyl groups and some hydrogens have been omitted for clarity.

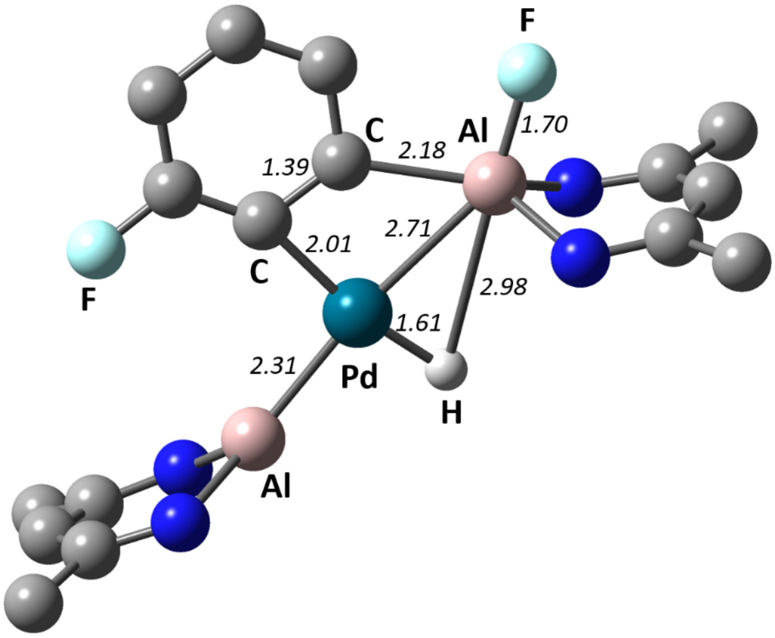

Fig. S.5.16: Selected bond lengths (in Å) for **TS-6**. 2,6-diisopropylphenyl groups and some hydrogens have been omitted for clarity.

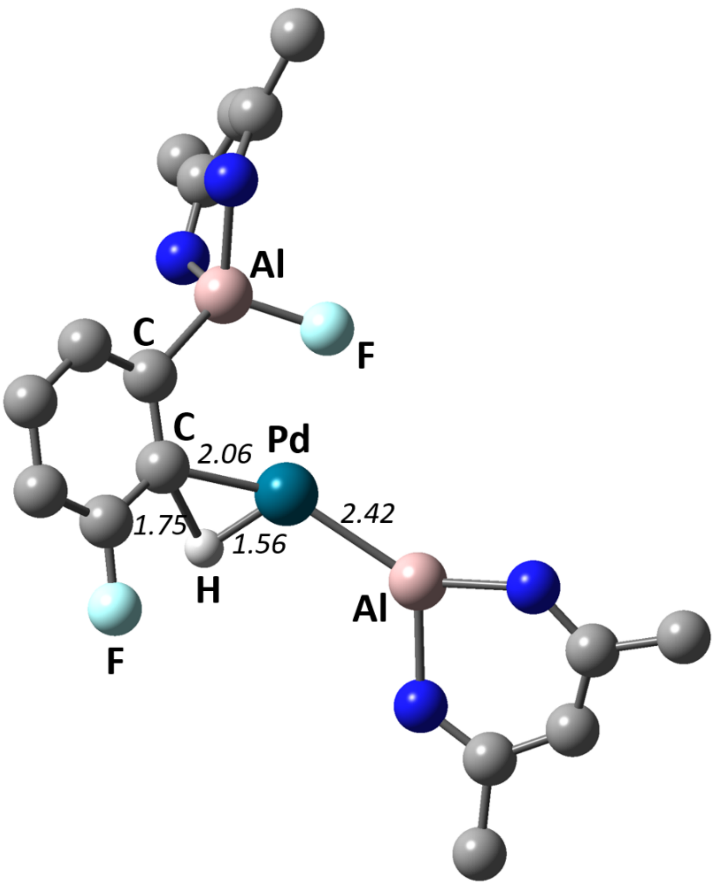

Fig. S.5.17: Selected bond lengths (in Å) for **TS-7**. 2,6-diisopropylphenyl groups and some hydrogens have been omitted for clarity.

5-2-3 C−H and C−F alumination of 1,3-difluorobenzene from [Pd(1)$_2$]: General Pathway

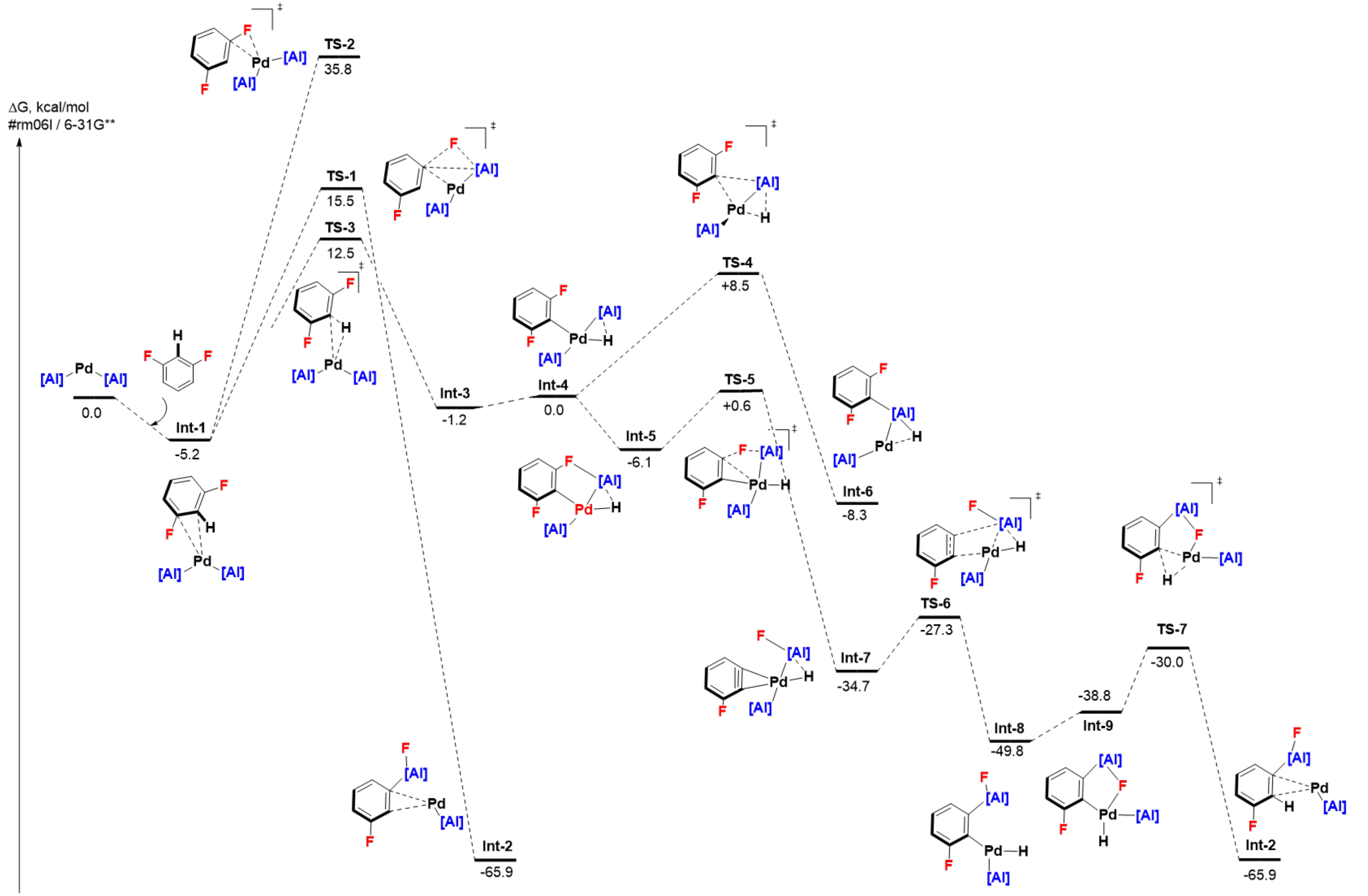

Figure. S.5.18: DFT calculated pathway for the palladium-catalyzed C–H and C–F alumination reactions of [Pd(**1**)$_2$] with 1,3-diFluorobenzene.

When dispersion and solvent corrections were included into the calculations with M06L for the general pathway, no impact on the local barriers energies was observed (**Table S.5.1**).

Functional	ΔTS-1	ΔTS-2	ΔTS-3	ΔTS-4	ΔTS-5	ΔTS-6	ΔTS-7
**M06L**	20.7	41.0	17.7	14.6	6.7	7.4	19.8
**M06L (GD3)**	23.5	41.1	17.9	15.4	6.7	7.3	21.8
**M06L (pcm)**	20.9	40.6	16.7	14.2	6.8	7.0	19.6

Table S.5.1: Comparison of the calculated free-energy local barriers using M06L and including dispersion (GD3) and solvent (pcm) corrections. All energies provided in kcal mol^{-1}.

The performance of the three functionals was then inspected for both **TS-1**, **TS-3**, **TS-4** and **TS-5**. M06L appeared to be more robust.

Functional	ΔTS-1	ΔTS-3	ΔTS-4	ΔTS-5
**M06L**	20.7	17.7	14.6	6.7
**ωB97X**	20.5	16.6	11.1	14.4
**B3PW91**	23.2	17.0	12.5	12.6

Table S.5.2: Comparison of the calculated free-energy local barriers of M06L with ωB97X and B3PW91. All energies provided in kcal mol^{-1}.

5-2-4 Key transition states for Fluorobenzene and 1,2,3-trifluorobenzene

TS-1 and **TS-2** were calculated for FB and 1,2,3-triFB and compared to those of 1,3-diFB.

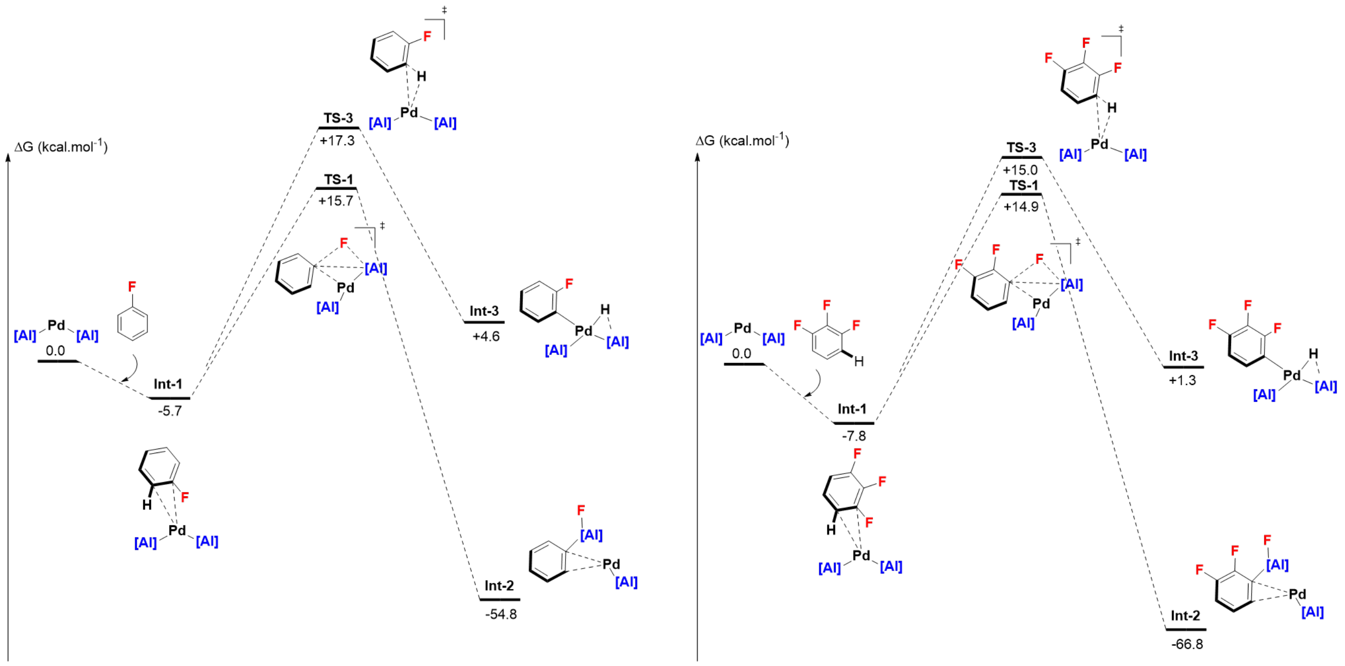

Fig. S.5.19: DFT calculated pathway for the palladium-catalyzed C–H and C–F alumination reactions of [Pd(**1**)$_2$] with Fluorobenzene (left) and 1,2,3-diFluorobenzene (right).

	Fluorobenzene	1,3-difluorobenzene	1,2,3-trifluorobenzene
**Int-1**	-5.7	-5.2	-7.8
**TS-1**	**15.7**	**15.5**	**14.9**
**Int-2**	-54.8	-59.1	-66.8
**TS-3**	**17.3**	**12.5**	**15.0**
**Int-3**	4.6	-1.2	1.3

Table S.5.3: Comparison of the ligand Assisted pathway TS-1 and the C–H oxidative addition pathway TS-3 for FB, 1,2-diFB and 1,2,3-triFB.

5-2-5 C–H and C–F alumination of 1,3-difluorobenzene from [Pd(1)(PCy_3)]

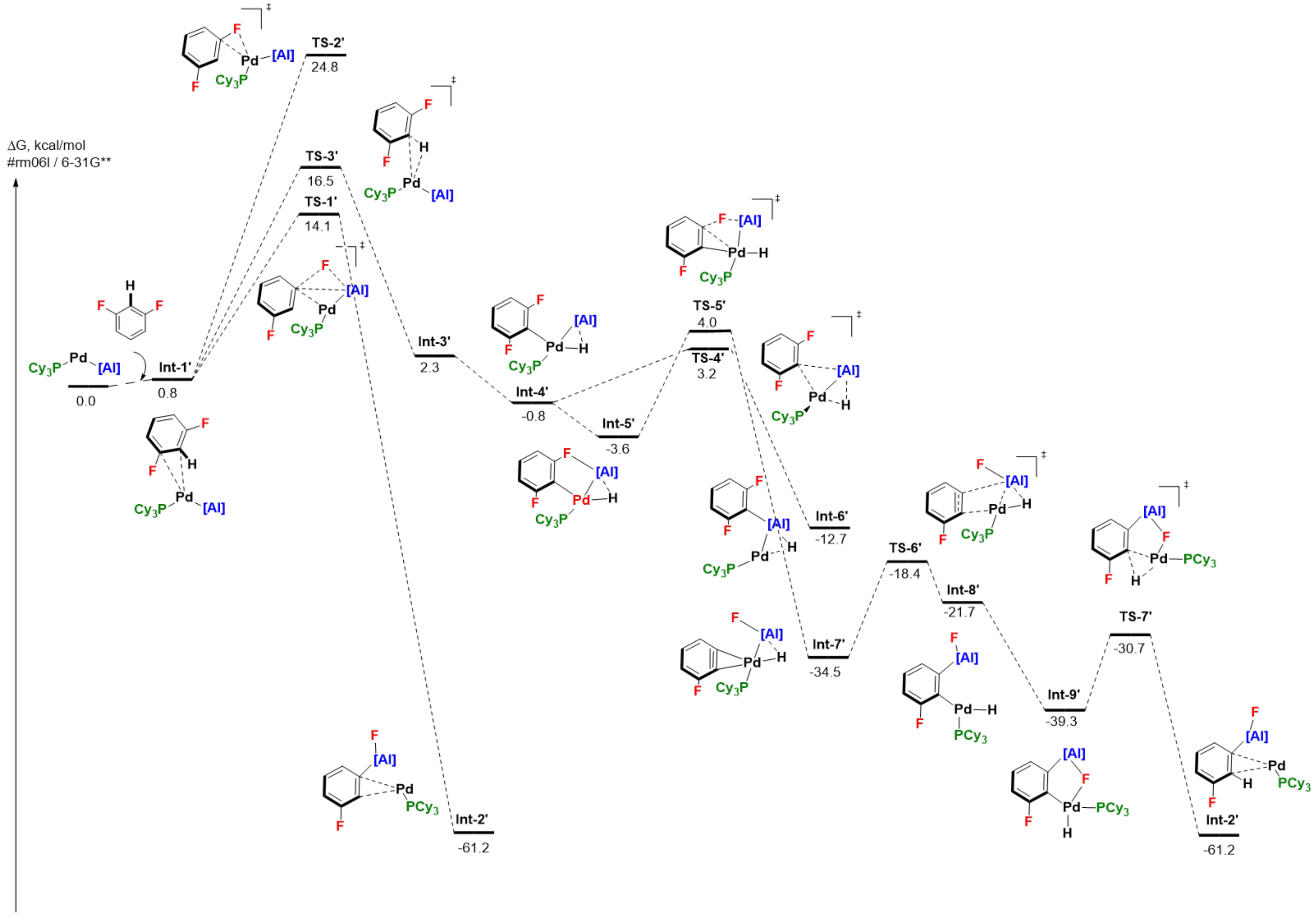

Fig. S.5.20: DFT calculated pathway for the palladium-catalyzed C–H and C–F alumination reactions of [Pd(**1**)(PCy_3)] with 1,3-diFluorobenzene

Depending on the PdL_2 fragment that is formed under catalytic conditions, two pathways can be considered. The pathway from [Pd(**1**)$_2$] is provided in the main text, while the pathway from [Pd(**1**)$_2$(PCy$_3$)] is given above.

	**[Pd(1)$_2$]**	**[Pd(1)(PCy$_3$)]**
**INT-1 & INT-1’**	-5.2	0.8
**TS-1 & TS-1’**	**15.5**	**14.1**
**INT-2 & INT-2’**	-65.9	-61.2
**TS-2 & TS-2’**	**35.8**	**24.8**
**TS-3 & TS-3’**	**12.5**	**16.5**
**INT-3 & INT-3’**	-1.2	2.3
**INT-4 & INT-4’**	0.0	-0.8
**TS-4 & TS-4’**	**8.5**	**3.2**
**INT-6 & INT-6’**	-8.3	-12.7
**INT-5 & INT-5’**	-6.1	-3.6
**TS-5 & TS-5’**	**0.6**	**4.0**
**INT-7 & INT-7’**	-34.7	-34.5
**TS-6 & TS-6’**	**-27.3**	**-18.4**
**INT-8 & INT-8’**	-49.8	-21.7
**INT-9 & INT-9’**	-38.8	-39.3
**TS-7 & TS-7’**	**-30.0**	**-30.7**
**INT-2 & INT-2’**	-65.9	-61.2
**Δ(TS-1 – Int-1) & Δ(TS-1’ – [Pd(1)(PCy$_3$)])**	**20.7**	**14.1**
**Δ(TS-2 – Int-1) & Δ(TS-2’ – [Pd(1)(PCy$_3$)])**	**41.0**	**24.8**
**Δ(TS-3 – Int-1) & Δ(TS-3’ – [Pd(1)(PCy$_3$)])**	**17.7**	**16.5**
**Δ(TS-4 – Int-5) or Δ(TS-4’ – Int-5’)**	**14.6**	**6.8**
**Δ(TS-5 – Int-5) & Δ(TS-5’ – Int-5’)**	**6.7**	**7.6**
**Δ(TS-6 – Int-7) & Δ(TS-6’ – Int-7’)**	**7.4**	**16.1**
**Δ(TS-7 – Int-8) & Δ(TS-7’ – Int-9’)**	**19.8**	**8.6**

Table S.5.3: Comparison of the calculated free energy profile for C–H and C–F alumination reactions of [Pd(**1**)$_2$] and [Pd(**1**)(PCy$_3$)] with 1,3-difluorobenzene.

For both pathways, initiated from either [Pd(**1**)$_2$] or [Pd(**1**)(PCy$_3$)], direct C–F oxidative addition on Pd was found to be high in energy and **TS-2** and **TS-2’** are not expected to be competitive with the other pathways. However, both key transition states for ligand-assisted oxidative addition pathway (**TS-1** and **TS-1’**) and the stepwise C–H → C–F functionalisation pathway (**TS-3 to TS-7** and **TS-3’ to TS-7’**) are within a reasonable energy range of one another. It is plausible that both these mechanisms may be in operation. Comparing both pathways, initiated from either [Pd(**1**)$_2$] or [Pd(**1**)(PCy$_3$)], lead to subtle different trends due to the presence of a second metalloligand on the former complex. For example, the presence of the Al atom considering [Pd(**1**)$_2$] catalyst allows a better stabilization of the Pd centre. The

absence of a stabilizing σ donation from the Al–H bond to the Pd centre in **Int-8'** leads to a higher energy intermediate compared to **Int-8**.

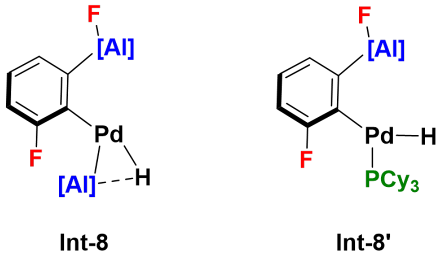

Fig. S.5.21: Comparison of **Int-8** and **Int-8'**.

4-4- NBO analysis

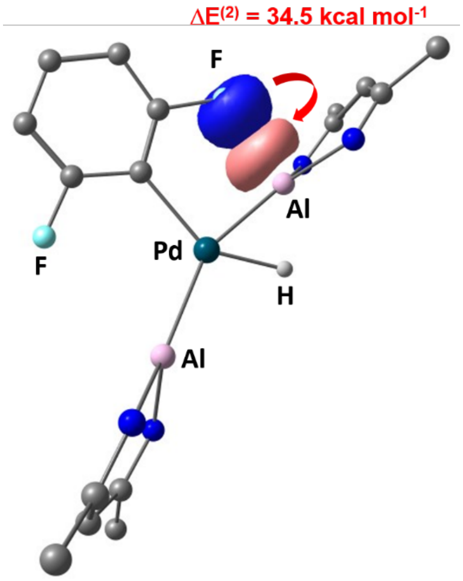

Fig. S.5.22: NBO Analysis of **Int-5**: Donor Acceptor interaction with the associated energy.

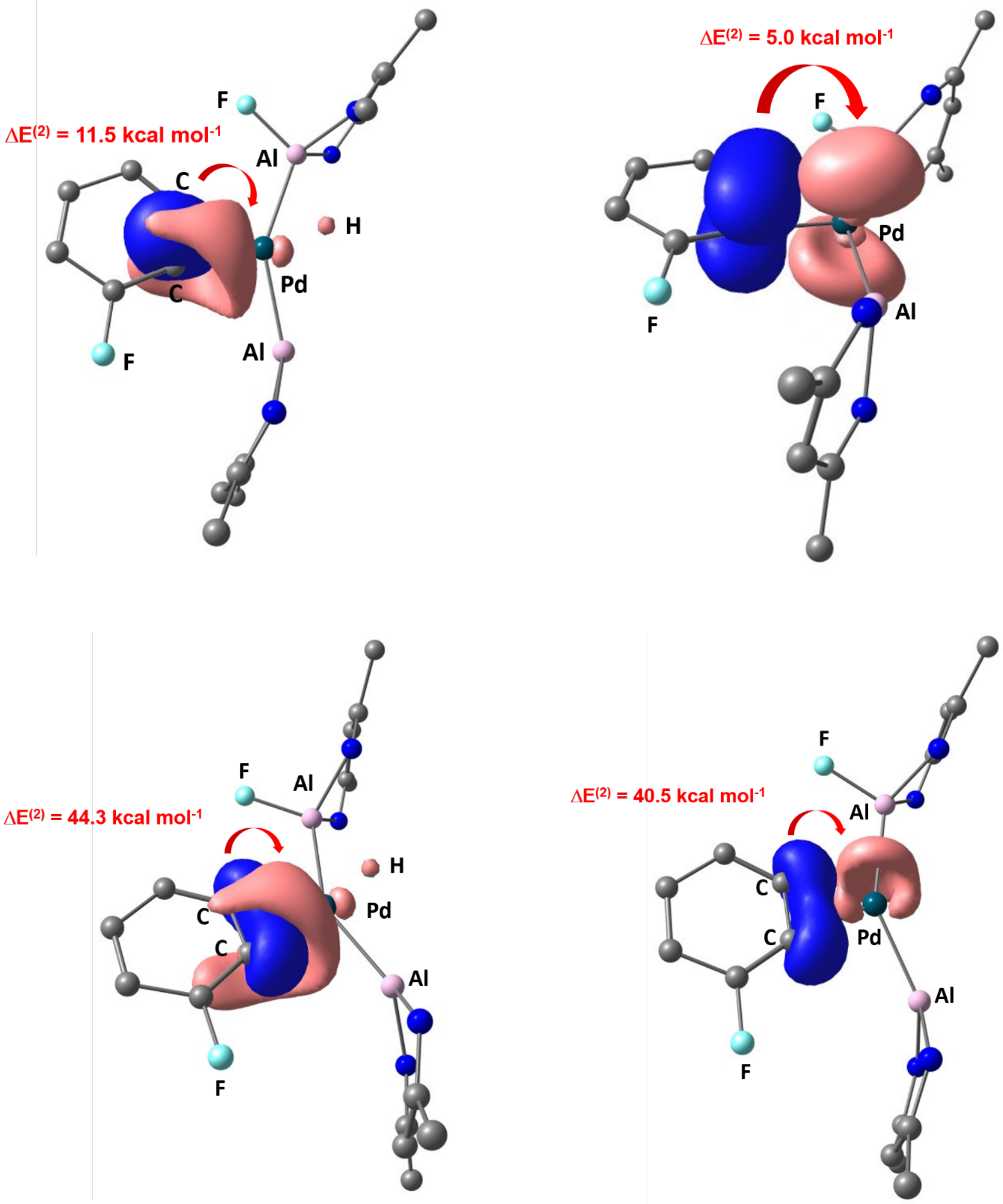

Fig. S.5.23: NBO Analysis of **Int-7**: Donor Acceptor interactions with the associated energies.

Wiberg Bond Indices (WBI) and NPA charges were inspected (M06L) for pathway from [Pd(**1**)$_2$].

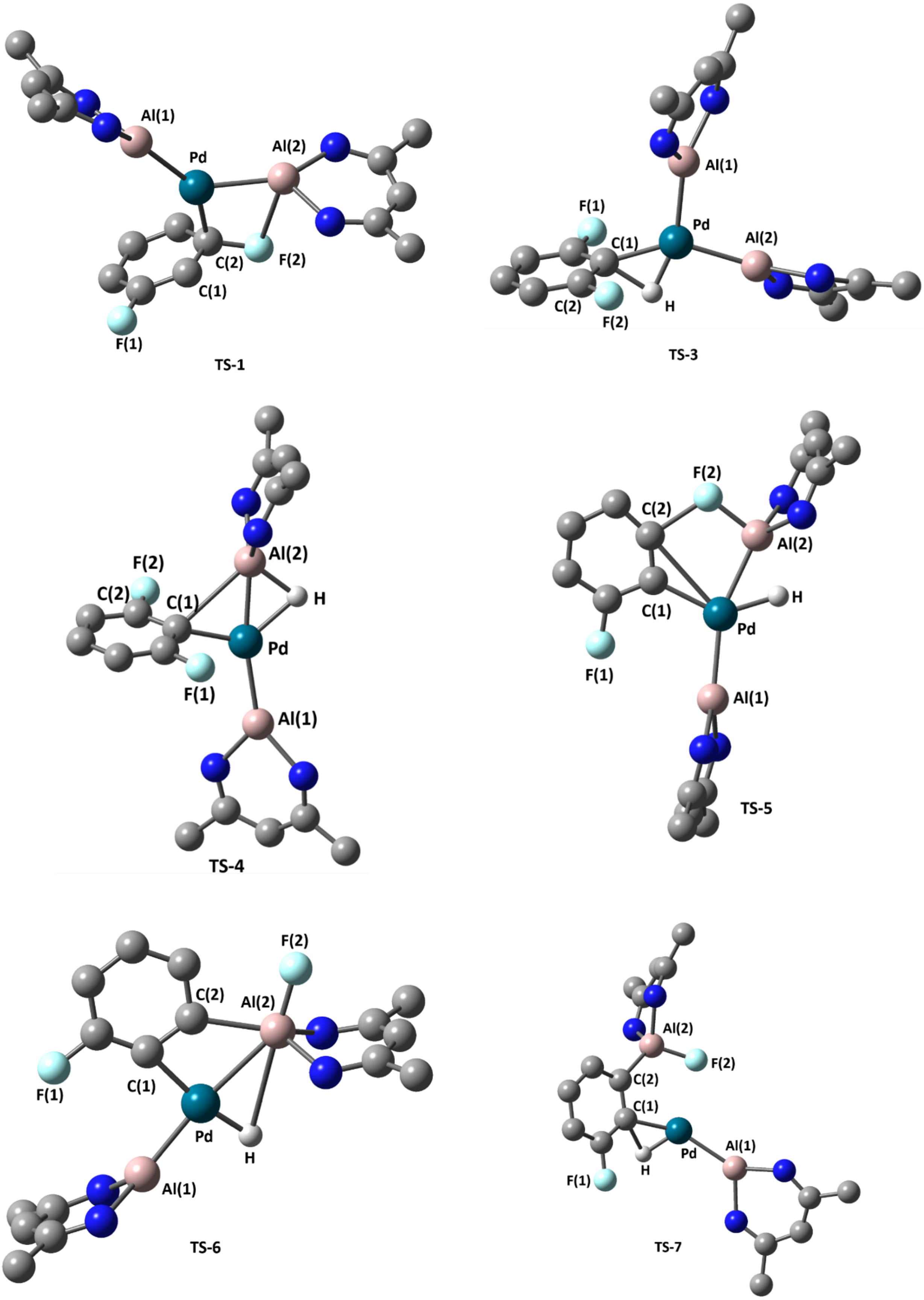

Fig. S.5.25: Models for **TS-1, TS-3, TS-4, TS-5, TS-6** and **TS-7**, showcasing the atoms and bonds relevant to the NBO analysis.

	Int-1	TS-1	Int-2	TS-3	Int-3	Int-4	TS-4	Int-6
Pd-Al(1)	0.77	0.78	0.85	0.84	0.79	0.83	0.89	0.78
Pd-Al(2)	0.78	0.70	0.07	0.86	0.62	0.71	0.51	0.26
Pd-H	0.01	0.02	0.01	0.41	0.28	0.46	0.27	0.17
Pd-C1	0.08	0.16	0.17	0.42	0.45	0.50	0.42	0.05
Pd-C2	0.04	0.34	0.21	0.04	0.03	0.03	0.04	0.02
Al(1)-H	0.00	0.00	0.00	0.07	0.09	0.06	0.03	0.07
Al(2)-H	0.00	0.01	0.00	0.10	0.44	0.28	0.48	0.54
Al(2)-C2	0.01	0.10	0.34	0.01	0.00	0.01	0.01	0.01
Al(2)-F2	0.01	0.09	0.35	0.00	0.00	0.00	0.01	0.02
C1-H	0.87	0.88	0.88	0.36	0.01	0.11	0.04	0.02
C2-F2	0.88	0.73	0.02	0.86	0.84	0.84	0.85	0.85
C1-C2	1.34	1.24	1.33	1.35	1.39	1.38	1.37	1.39

Table S.5.4: Wiberg Bond Indices on stationary points on the pathway from [Pd(**1**)$_2$] of **Int-1**, **TS-1**, **Int-2**, **Int-3**, **TS-3**, **Int-4**, **TS-4** and **Int-6**.

	Int-5	TS-5	Int-7	TS-6	Int-8	Int-9	TS-7	Int-2	Int-S2
Pd-Al(1)	0.87	0.82	0.77	0.74	0.54	0.76	0.78	0.85	0.81
Pd-Al(2)	0.54	0.38	0.32	0.17	0.04	0.05	0.05	0.07	0.05
Pd-H	0.39	0.32	0.48	0.52	0.20	0.67	0.48	0.01	0.01
Pd-C1	0.42	0.35	0.49	0.41	0.43	0.45	0.38	0.17	0.13
Pd-C2	0.04	0.11	0.50	0.23	0.06	0.05	0.07	0.21	0.20
Al(1)-H	0.05	0.04	0.054	0.13	0.49	0.13	0.043	0.00	0.00
Al(2)-H	0.38	0.45	0.28	0.09	0.00	0.00	0.01	0.00	0.00
Al(2)-C2	0.01	0.04	0.14	0.38	0.42	0.44	0.39	0.34	0.40
Al(2)-F2	0.12	0.25	0.37	0.32	0.36	0.28	0.34	0.35	0.39
C1-H	0.06	0.03	0.09	0.17	0.08	0.11	0.40	0.88	0.89
C2-F2	0.72	0.26	0.01	0.02	0.02	0.02	0.02	0.02	0.02
C1-C2	1.44	1.73	1.56	1.43	1.37	1.40	1.39	1.33	1.33

Table S.5.5: Wiberg Bond Indices on stationary points on the pathway from [Pd(**1**)$_2$] of **Int-5**, **TS-5**, **Int-7**, **TS-6**, **Int-8**, **Int-9**, **TS-7**, **Int-2** and **Int-S2**.

	Pd	H	C1	C2	Al(1)	Al(2)	F1	F2
**Int-1**	-0.89	0.28	-0.42	-0.46	1.11	1.18	-0.33	-0.32
**TS-1**	-0.41	0.11	-0.22	0.21	0.75	0.87	-0.37	-0.35
**Int-2**	-0.35	0.25	-0.34	-0.79	1.13	2.19	-0.34	-0.74
**TS-3**	-0.59	0.01	-0.39	0.51	0.91	0.95	-0.36	-0.35
**Int-3**	-0.79	-0.33	-0.39	0.42	1.46	1.44	-0.35	-0.35
**Int-4**	-1.05	-0.21	-0.35	0.41	1.52	1.55	-0.34	-0.35
**TS-4**	-0.84	-0.34	-0.42	0.41	1.36	1.61	-0.34	-0.34
**Int-6**	-0.59	-0.37	-0.72	0.44	1.23	1.69	-0.33	-0.34
**Int-5**	-0.88	-0.23	-0.39	0.36	1.33	1.64	-0.34	-0.38
**TS-5**	-0.68	-0.28	-0.33	0.27	1.24	1.74	-0.33	-0.61
**Int-7**	-0.77	-0.19	-0.28	-0.13	1.55	1.94	-0.34	-0.75
**TS-6**	-0.53	-0.16	-0.28	-0.57	1.54	2.04	-0.34	-0.76
**Int-8**	-0.17	-0.40	-0.33	-0.70	1.54	2.13	-0.38	-0.75
**Int-9**	-0.33	0.02	-0.33	-0.73	1.36	2.16	-0.34	-0.70
**TS-7**	-0.30	0.09	-0.29	-0.71	1.16	2.17	-0.34	-0.73
**Int-2**	-0.35	0.25	-0.34	-0.79	1.13	2.19	-0.34	-0.74
**Int-S2**	-0.37	0.24	-0.31	-0.76	1.07	2.12	-0.34	-0.73

Table S.5.6: NPA charges on stationary points on the pathway from [Pd(**1**)$_2$].

4-5- XYZ Coordinates

[Pd(1)$_2$].log

SCF (M06L) = -2610.58136399
E(SCF)+ZPE(0 K) = -2609.302627
H(298 K) = -2609.228276
G(298 K) = -2609.409952
Lowest Frequency = 16.2727 cm^{-1}

Pd	0.004121000	-0.065181000	-1.394658000
Al	2.149727000	-0.207636000	-0.377820000
N	3.392991000	1.323921000	-0.175072000
N	2.874528000	-1.038878000	1.281248000
C	4.061002000	1.678612000	0.926832000
C	4.151536000	0.851651000	2.056292000
H	4.740979000	1.232170000	2.882836000
C	3.679530000	-0.459255000	2.183656000
C	4.792716000	2.987883000	0.969405000
H	5.453947000	3.099514000	0.104165000
H	5.381933000	3.085446000	1.882304000
H	4.086010000	3.823445000	0.922345000
C	4.129424000	-1.239795000	3.384487000
H	3.279127000	-1.531263000	4.010193000
H	4.825910000	-0.663393000	3.994510000
H	4.614776000	-2.173467000	3.082097000
C	3.539710000	2.130970000	-1.358216000
C	4.422756000	1.671187000	-2.361682000
C	4.592887000	2.456188000	-3.503242000
H	5.271887000	2.124468000	-4.283238000
C	3.897502000	3.648458000	-3.668757000
H	4.045565000	4.245121000	-4.564733000
C	2.994894000	4.055850000	-2.698736000
H	2.419203000	4.967730000	-2.844623000
C	2.791258000	3.309386000	-1.534654000
C	5.158760000	0.351791000	-2.209161000
H	4.446602000	-0.355556000	-1.750987000
C	5.584399000	-0.259540000	-3.537691000
H	4.743288000	-0.352264000	-4.231201000
H	6.000797000	-1.258591000	-3.374491000
H	6.366230000	0.330657000	-4.028495000
C	6.363530000	0.456174000	-1.272924000
H	7.083227000	1.192897000	-1.648878000
H	6.881185000	-0.507461000	-1.204355000
H	6.081095000	0.746993000	-0.257440000
C	1.726113000	3.753166000	-0.554874000
H	1.803535000	3.120611000	0.335693000
C	0.336866000	3.531722000	-1.154116000
H	-0.443812000	3.806097000	-0.435849000
H	0.182270000	2.477753000	-1.429139000
H	0.199770000	4.144884000	-2.053769000
C	1.882787000	5.207976000	-0.116128000
H	2.874848000	5.423025000	0.296497000
H	1.136299000	5.452436000	0.647737000
H	1.724019000	5.897788000	-0.952691000
C	2.572920000	-2.435317000	1.436406000
C	1.543478000	-2.860400000	2.301077000
C	1.259720000	-4.226025000	2.375544000
H	0.460705000	-4.561438000	3.034165000
C	1.964893000	-5.157648000	1.624612000
H	1.721606000	-6.214309000	1.694932000
C	2.978242000	-4.725069000	0.781615000
H	3.533582000	-5.449048000	0.188414000
C	3.301346000	-3.370017000	0.670752000
C	0.721312000	-1.897229000	3.135358000
H	1.205834000	-0.914633000	3.096321000
C	-0.681573000	-1.740471000	2.550060000
H	-1.172416000	-2.715285000	2.436074000
H	-0.636043000	-1.268728000	1.558420000
H	-1.318940000	-1.127223000	3.197971000
C	0.639939000	-2.321460000	4.601216000
H	0.060597000	-3.242944000	4.723620000
H	0.141775000	-1.548435000	5.195593000
H	1.628055000	-2.499467000	5.038758000
C	4.421323000	-2.964334000	-0.268418000
H	4.576407000	-1.881513000	-0.170778000
C	4.044881000	-3.256653000	-1.720975000
H	3.843524000	-4.324154000	-1.868299000
H	4.857528000	-2.974770000	-2.399279000
H	3.145701000	-2.704309000	-2.019604000
C	5.740182000	-3.642642000	0.098771000
H	6.038914000	-3.422177000	1.128545000
H	6.544436000	-3.306771000	-0.564238000
H	5.672223000	-4.731884000	0.001667000
Al	-2.129338000	0.166050000	-0.362975000
N	-3.394498000	-1.339659000	-0.085919000
N	-2.845512000	1.090910000	1.246682000
C	-4.169324000	-1.572692000	0.975232000
C	-4.216871000	-0.710423000	2.080798000
H	-4.850212000	-1.021068000	2.904291000
C	-3.658801000	0.568780000	2.178743000
C	-5.096726000	-2.753248000	0.987068000
H	-5.994458000	-2.531022000	0.397918000
H	-5.418223000	-2.990611000	2.002830000
H	-4.642482000	-3.636668000	0.531854000
C	-4.032424000	1.385234000	3.382943000
H	-3.161276000	1.557516000	4.023560000
H	-4.798617000	0.884857000	3.976534000
H	-4.398732000	2.375419000	3.093724000
C	-3.565838000	-2.157081000	-1.256114000
C	-4.649562000	-1.893305000	-2.117751000
C	-4.791674000	-2.674488000	-3.266191000
H	-5.621053000	-2.476156000	-3.942289000
C	-3.886098000	-3.683639000	-3.564846000
H	-4.013920000	-4.284961000	-4.460836000
C	-2.806544000	-3.909236000	-2.720959000
H	-2.088201000	-4.688523000	-2.963703000
C	-2.615223000	-3.151015000	-1.562958000
C	-5.606315000	-0.738460000	-1.886366000
H	-5.445965000	-0.346706000	-0.873363000
C	-5.295395000	0.391060000	-2.869980000
H	-5.450950000	0.058851000	-3.902684000
H	-4.252642000	0.721081000	-2.787256000
H	-5.946148000	1.255977000	-2.695631000
C	-7.072698000	-1.149158000	-1.994273000
H	-7.727379000	-0.308879000	-1.740450000
H	-7.318960000	-1.980989000	-1.326182000
H	-7.329850000	-1.462801000	-3.011846000
C	-1.441971000	-3.442959000	-0.650255000
H	-1.262458000	-2.545268000	-0.046613000
C	-1.771709000	-4.595635000	0.298537000
H	-2.627046000	-4.365641000	0.943753000
H	-0.915321000	-4.819069000	0.943869000
H	-2.015556000	-5.506438000	-0.261928000
C	-0.144736000	-3.714394000	-1.403942000
H	0.686874000	-3.793995000	-0.693876000
H	0.088338000	-2.891370000	-2.090026000
H	-0.179187000	-4.651361000	-1.973375000
C	-2.495010000	2.480869000	1.355528000
C	-1.481668000	2.913973000	2.232665000
C	-1.198577000	4.281107000	2.301102000
H	-0.412717000	4.622993000	2.972421000
C	-1.891283000	5.202214000	1.528653000
H	-1.660347000	6.261677000	1.600916000
C	-2.865345000	4.757932000	0.642880000
H	-3.387623000	5.478224000	0.019542000
C	-3.175827000	3.401339000	0.527034000
C	-0.653443000	1.954076000	3.062249000
H	-1.118920000	0.964006000	3.001224000
C	0.753758000	1.838219000	2.478729000
H	1.251131000	2.817145000	2.466925000
H	0.720616000	1.462476000	1.446131000
H	1.380819000	1.164981000	3.073854000
C	-0.575238000	2.354932000	4.534523000
H	-0.061318000	1.581112000	5.114665000
H	-1.562701000	2.510962000	4.981065000
H	-0.010173000	3.283915000	4.668100000
C	-4.235647000	2.939202000	-0.455557000
H	-3.939717000	1.938611000	-0.809125000

C	-4.325994000	3.823691000	-1.692228000
H	-4.737030000	4.813172000	-1.463822000
H	-4.986487000	3.368355000	-2.436059000
H	-3.342837000	3.962556000	-2.153722000
C	-5.600807000	2.786757000	0.215200000
H	-5.937163000	3.740957000	0.637527000
H	-5.578235000	2.049348000	1.024215000
H	-6.355295000	2.457402000	-0.508714000

[Pd(1)PCy$_3$].log

SCF (M06L) = -2416.41103039
E(SCF)+ZPE(0 K)= -2415.286339
H(298 K) = -2415.227133
G(298 K) = -2415.380701
Lowest Frequency = 13.8384 cm^{-1}

Pd	0.561963000	-0.353021000	-0.963757000
P	2.767456000	-0.368323000	-0.256873000
C	3.474321000	1.356343000	-0.206068000
H	4.523674000	1.306295000	0.127453000
C	2.721021000	2.275312000	0.758733000
H	1.652571000	2.282945000	0.483561000
H	2.774571000	1.889817000	1.785731000
C	3.280328000	3.693220000	0.719817000
H	2.728640000	4.330492000	1.420827000
H	4.324616000	3.680490000	1.068308000
C	3.228457000	4.270676000	-0.688159000
H	3.639534000	5.287247000	-0.706880000
H	2.175026000	4.351869000	-0.996461000
C	3.970648000	3.371396000	-1.669018000
H	5.042376000	3.366440000	-1.417021000
H	3.896752000	3.766812000	-2.689219000
C	3.436371000	1.944595000	-1.622316000
H	3.995480000	1.307284000	-2.320498000
H	2.388439000	1.925191000	-1.964780000
C	4.091712000	-1.291363000	-1.216362000
H	3.823974000	-1.060730000	-2.261352000
C	5.556902000	-0.882991000	-1.030298000
H	5.869729000	-1.076574000	0.005196000
H	5.687502000	0.192912000	-1.194877000
C	6.463599000	-1.654709000	-1.987232000
H	6.213493000	-1.368325000	-3.019826000
H	7.509493000	-1.363883000	-1.833535000
C	6.296181000	-3.161318000	-1.834287000
H	6.632577000	-3.460677000	-0.830149000
H	6.938216000	-3.694251000	-2.545067000
C	4.838712000	-3.569441000	-2.011933000
H	4.719205000	-4.649707000	-1.869233000
H	4.524373000	-3.356908000	-3.044243000
C	3.937151000	-2.805741000	-1.048689000
H	2.886071000	-3.095759000	-1.177096000
H	4.212434000	-3.079504000	-0.018736000
C	2.859765000	-0.896761000	1.527876000
H	2.260315000	-0.099207000	1.997409000
C	2.095232000	-2.193588000	1.816404000
H	1.124011000	-2.165885000	1.302559000
H	2.640546000	-3.053020000	1.402779000
C	1.925263000	-2.388271000	3.319250000
H	1.392402000	-3.324210000	3.526241000
H	1.290921000	-1.578947000	3.713812000
C	3.270060000	-2.369426000	4.038579000
H	3.132369000	-2.478843000	5.120627000
H	3.861495000	-3.238881000	3.714352000
C	4.050762000	-1.097066000	3.726401000
H	5.026167000	-1.109709000	4.226941000
H	3.505747000	-0.229627000	4.131026000
C	4.222366000	-0.906495000	2.220890000
H	4.830083000	-1.731830000	1.818965000
H	4.777698000	0.017587000	2.012477000
Al	-1.747191000	0.127060000	-0.651298000
N	-2.581626000	1.783699000	0.000072000
N	-3.421354000	-0.853921000	-0.362030000
C	-3.893392000	2.031739000	0.115719000
C	-4.867401000	1.034926000	-0.028410000
H	-5.900132000	1.349103000	0.070926000
C	-4.642270000	-0.339786000	-0.174603000
C	-4.359839000	3.423199000	0.430684000
H	-3.893330000	3.798182000	1.347584000
H	-5.443922000	3.462919000	0.543976000
H	-4.065253000	4.118374000	-0.363350000
C	-5.822481000	-1.259999000	-0.081617000
H	-5.830798000	-1.976959000	-0.908467000
H	-6.761532000	-0.705030000	-0.076931000
H	-5.772006000	-1.858084000	0.835306000
C	-1.662287000	2.830699000	0.351269000
C	-1.086374000	2.806152000	1.639200000
C	-0.254465000	3.865204000	2.008451000
H	0.194517000	3.871431000	2.998465000
C	0.031751000	4.897603000	1.122673000
H	0.678045000	5.716273000	1.428917000
C	-0.473779000	4.856733000	-0.169141000
H	-0.201182000	5.635567000	-0.878375000
C	-1.318810000	3.824021000	-0.582805000
C	-1.348151000	1.653085000	2.590257000
H	-1.465008000	0.750494000	1.971068000
C	-0.178615000	1.385394000	3.529781000
H	0.761625000	1.283419000	2.975703000
H	-0.344268000	0.457192000	4.085997000
H	-0.050632000	2.182840000	4.270334000
C	-2.646457000	1.831031000	3.377566000
H	-2.617278000	2.747595000	3.978619000
H	-2.803263000	0.988885000	4.061767000
H	-3.520678000	1.885944000	2.721223000
C	-1.759715000	3.748916000	-2.029788000
H	-2.572373000	3.014643000	-2.101184000
C	-0.594687000	3.236544000	-2.881509000
H	-0.893126000	3.117062000	-3.928512000
H	-0.227717000	2.268779000	-2.513131000
H	0.244182000	3.943650000	-2.849443000
C	-2.285273000	5.076892000	-2.567439000
H	-3.097175000	5.479532000	-1.952126000
H	-2.664636000	4.956036000	-3.586662000
H	-1.497944000	5.837312000	-2.607023000
C	-3.258118000	-2.281258000	-0.339055000
C	-3.163285000	-2.996288000	-1.546739000
C	-2.943142000	-4.374792000	-1.482966000
H	-2.868147000	-4.938866000	-2.410639000
C	-2.810558000	-5.029576000	-0.266602000
H	-2.638556000	-6.102001000	-0.238982000
C	-2.887753000	-4.304199000	0.916049000
H	-2.762761000	-4.813354000	1.869398000
C	-3.107936000	-2.925909000	0.905192000
C	-3.272326000	-2.328025000	-2.902390000
H	-3.470279000	-1.260834000	-2.740379000
C	-1.956936000	-2.445349000	-3.672173000
H	-1.713843000	-3.494900000	-3.874392000
H	-1.126463000	-2.008500000	-3.104585000
H	-2.023308000	-1.928642000	-4.635390000
C	-4.433010000	-2.895501000	-3.718279000
H	-4.282226000	-3.958525000	-3.937388000
H	-4.524940000	-2.375073000	-4.676832000
H	-5.389031000	-2.802915000	-3.192533000
C	-3.099084000	-2.154034000	2.211062000
H	-3.488192000	-1.145369000	2.020408000
C	-1.661590000	-2.005716000	2.712833000
H	-1.217920000	-2.988361000	2.912229000
H	-1.625784000	-1.427545000	3.643957000
H	-1.022159000	-1.506219000	1.971665000
C	-3.980902000	-2.789337000	3.282581000
H	-5.009408000	-2.932808000	2.935625000
H	-4.012569000	-2.158497000	4.176878000
H	-3.600628000	-3.768488000	3.593122000

1-3-difluorobenzene_M06L.log

SCF (M06L) = -430.668681868
E(SCF)+ZPE(0 K) = -430.584165
H(298 K) = -430.577311

G(298 K) = -430.614281
Lowest Frequency = 234.1176 cm^{-1}

C	1.212737	1.082175	0.000001
C	1.185339	0.306072	0.000030
C	0.000001	1.027923	0.000018
C	1.185342	0.306064	0.000004
C	1.212738	1.082176	0.000004
C	0.000003	1.765829	0.000018
H	2.164573	1.600357	0.000017
H	0.000012	2.110708	0.000018
H	2.164566	1.600373	0.000015
H	0.000000	2.851409	0.000039
F	2.345417	0.982343	0.000002
F	2.345418	0.982341	0.000011

Fluorobenzene_M06L.log

SCF (M06L) = -331.444587957
E(SCF)+ZPE(0 K)= -331.352010
H(298 K)= -331.345926
G(298 K)= -331.380828
Lowest Frequency = 240.8324 cm^{-1}

C	0.930458	0.000184	0.000008
C	0.259413	1.214944	0.000010
C	1.132607	1.205275	0.000022
C	1.831361	0.000125	0.000015
C	1.132526	1.205372	0.000003
C	0.259650	1.214730	0.000015
H	2.916900	0.000331	0.000024
F	2.276350	0.000066	0.000019
H	0.827459	2.138919	0.000029
H	1.671343	2.148525	0.000008
H	1.671861	2.148195	0.000036
H	0.827320	2.139121	0.000015

1,2,3-Trifluorobenzene _M06L.log

SCF (M06L) = 529.879809653
E(SCF)+ZPE(0 K) = 529.803363
H(298 K) = 529.795584
G(298 K) = 529.834746
Lowest Frequency = 157.2409 cm^{-1}

C	1.198338	0.014774	0.000014
C	-0.000250	0.720685	0.000004
C	-1.198414	0.013388	0.000015
C	-1.210491	1.373458	0.000009
C	0.001185	2.059484	0.000009
C	1.212015	1.372338	0.000021
F	-0.001491	2.055485	0.000009
H	-2.163459	1.889706	0.000018
F	-2.341934	0.707838	0.000031
F	2.341409	0.709977	0.000026
H	2.165689	1.887233	0.000035
H	0.001619	3.144164	0.000014

Int-1_AlPdAl_ M06L.log

SCF (M06L) = -3041.28411458
E(SCF)+ZPE(0 K) = -3039.919656
H(298 K) = -3039.838174
G(298 K) = -3040.004255
Lowest Frequency = 24.4723 cm^{-1}

Pd	0.024282000	-0.142823000	1.080739000
Al	-2.053148000	-0.158213000	-0.067793000
N	-3.222638000	1.430546000	-0.306198000
N	-2.782432000	-0.955652000	-1.744697000
C	-3.901400000	1.788185000	-1.402785000
C	-4.077909000	0.929439000	-2.495189000
H	-4.706450000	1.297048000	-3.298679000
C	-3.661215000	-0.404562000	-2.592519000
C	-4.566850000	3.132088000	-1.465685000
H	-5.224003000	3.289771000	-0.604098000
H	-5.149933000	3.245040000	-2.380811000
H	-3.819572000	3.932301000	-1.429674000
C	-4.312105000	-1.240924000	-3.659141000
H	-3.627971000	-1.957632000	-4.118560000
H	-4.752522000	-0.612502000	-4.435473000
H	-5.122380000	-1.830949000	-3.212851000
C	-3.343636000	2.256221000	0.864579000
C	-4.258265000	1.843886000	1.859756000
C	-4.439078000	2.666755000	2.973234000
H	-5.150362000	2.375433000	3.741743000
C	-3.712528000	3.842467000	3.127226000
H	-3.869347000	4.469860000	4.000833000
C	-2.768980000	4.194624000	2.172959000
H	-2.169280000	5.092540000	2.311243000
C	-2.561701000	3.414179000	1.031724000
C	-5.021675000	0.537488000	1.728079000
H	-4.331762000	-0.188041000	1.262913000
C	-5.432114000	-0.052573000	3.071370000
H	-4.581736000	-0.124393000	3.756233000
H	-5.839407000	-1.058838000	2.933613000
H	-6.212658000	0.541038000	3.560483000
C	-6.241308000	0.658125000	0.813555000
H	-6.941914000	1.409053000	1.197699000
H	-6.776245000	-0.297408000	0.759816000
H	-5.970677000	0.939027000	-0.208183000
C	-1.467354000	3.800480000	0.059520000
H	-1.564594000	3.162993000	-0.826055000
C	-0.096406000	3.519790000	0.675309000
H	0.703735000	3.768600000	-0.031325000
H	0.011355000	2.459711000	0.944347000
H	0.056268000	4.119506000	1.582456000
C	-1.553372000	5.255328000	-0.397189000
H	-2.532099000	5.509267000	-0.819527000
H	-0.791771000	5.454529000	-1.159294000
H	-1.367817000	5.948629000	0.431014000
C	-2.487135000	-2.351017000	-1.912331000
C	-1.338743000	-2.730001000	-2.638826000
C	-1.114240000	-4.093125000	-2.845254000
H	-0.250895000	-4.406270000	-3.426932000
C	-1.966693000	-5.056393000	-2.317297000
H	-1.767238000	-6.111156000	-2.486207000
C	-3.059259000	-4.664810000	-1.556097000
H	-3.708602000	-5.419306000	-1.117112000
C	-3.342405000	-3.313262000	-1.340866000
C	-0.406223000	-1.692464000	-3.236814000
H	-0.443126000	-0.808990000	-2.581990000
C	1.049621000	-2.140636000	-3.305499000
H	1.196620000	-2.984344000	-3.989795000
H	1.440794000	-2.431679000	-2.326258000
H	1.676656000	-1.322793000	-3.678887000
C	-0.872618000	-1.269974000	-4.631202000
H	-0.952143000	-2.139645000	-5.294684000
H	-0.154366000	-0.575380000	-5.081019000
H	-1.846191000	-0.771539000	-4.614479000
C	-4.523234000	-2.925707000	-0.470281000
H	-4.707814000	-1.850168000	-0.600055000
C	-4.193270000	-3.161384000	1.005846000
H	-3.965142000	-4.218073000	1.189172000
H	-5.041521000	-2.884697000	1.642722000
H	-3.321746000	-2.574085000	1.323851000
C	-5.804419000	-3.660940000	-0.856789000
H	-6.042543000	-3.542659000	-1.919065000
H	-6.653257000	-3.284647000	-0.277022000
H	-5.730429000	-4.735361000	-0.656642000
Al	2.023724000	0.025332000	-0.175294000
N	3.277564000	-1.519688000	-0.259345000
N	2.915408000	0.877678000	-1.716824000
C	4.168766000	-1.789930000	-1.212453000
C	4.321845000	-0.978538000	-2.350693000
H	5.018853000	-1.333810000	-3.101837000
C	3.788782000	0.298021000	-2.557994000
C	5.104925000	-2.954471000	-1.068002000

H	4.637069000	-3.797297000	-0.553818000
H	5.973767000	-2.662542000	-0.466376000
H	5.477211000	-3.281881000	-2.040867000
C	4.249950000	1.051276000	-3.772057000
H	3.407653000	1.253186000	-4.443298000
H	5.005269000	0.491431000	-4.325116000
H	4.662653000	2.028341000	-3.499842000
C	3.304154000	-2.249451000	0.978797000
C	4.280396000	-1.932692000	1.944668000
C	4.248948000	-2.603536000	3.170671000
H	4.997858000	-2.362585000	3.922935000
C	3.270251000	-3.546240000	3.449752000
H	3.257507000	-4.055322000	4.409551000
C	2.292209000	-3.819531000	2.501689000
H	1.507450000	-4.533830000	2.733103000
C	2.278710000	-3.177922000	1.261614000
C	5.300699000	-0.827640000	1.750206000
H	5.235639000	-0.462291000	0.716483000
C	4.969978000	0.343805000	2.676500000
H	5.024212000	0.033987000	3.726566000
H	3.958856000	0.730946000	2.500921000
H	5.682253000	1.165062000	2.537433000
C	6.733226000	-1.300154000	1.990698000
H	7.447148000	-0.499873000	1.769455000
H	6.994636000	-2.165239000	1.372839000
H	6.886442000	-1.591271000	3.035730000
C	1.217935000	-3.522526000	0.236861000
H	1.027556000	-2.614743000	-0.353752000
C	1.726973000	-4.615579000	-0.703585000
H	2.605506000	-4.291326000	-1.272752000
H	0.951104000	-4.898964000	-1.422029000
H	2.007603000	-5.512858000	-0.138781000
C	-0.122964000	-3.908000000	0.848550000
H	-0.875316000	-3.997441000	0.058048000
H	-0.467876000	-3.144202000	1.552841000
H	-0.083664000	-4.872338000	1.370052000
C	2.641610000	2.281741000	-1.869115000
C	1.670832000	2.749357000	-2.774855000
C	1.464541000	4.128614000	-2.879470000
H	0.709721000	4.497299000	-3.571513000
C	2.193465000	5.029164000	-2.117806000
H	2.026675000	6.097847000	-2.223243000
C	3.114463000	4.552377000	-1.193576000
H	3.654340000	5.256973000	-0.567773000
C	3.339070000	3.183719000	-1.032105000
C	0.790601000	1.825968000	-3.592583000
H	1.172985000	0.801156000	-3.487122000
C	-0.633875000	1.849097000	-3.035950000
H	-1.052813000	2.863463000	-3.076912000
H	-0.645677000	1.524391000	-1.986292000
H	-1.301318000	1.193903000	-3.605470000
C	0.787908000	2.190092000	-5.076703000
H	0.187858000	1.477563000	-5.651729000
H	1.796157000	2.204353000	-5.503673000
H	0.351801000	3.180771000	-5.243520000
C	4.300080000	2.691954000	0.034592000
H	3.913170000	1.725887000	0.397901000
C	4.354125000	3.617748000	1.244015000
H	4.834553000	4.574474000	1.011728000
H	4.934766000	3.158425000	2.048448000
H	3.350771000	3.823869000	1.630962000
C	5.698695000	2.432930000	-0.524695000
H	6.124893000	3.348681000	-0.951302000
H	5.692103000	1.667258000	-1.307432000
H	6.374736000	2.088331000	0.266793000
H	1.651684000	-0.714089000	3.346864000
C	0.682095000	-0.271963000	3.553162000
C	0.493605000	1.114735000	3.581930000
C	-0.414358000	-1.053976000	3.922567000
C	-0.709961000	1.701302000	3.949256000
C	-1.627302000	-0.509952000	4.329597000
C	-1.761599000	0.873599000	4.343896000
H	-0.809180000	2.781148000	3.935457000
H	-2.437634000	-1.172255000	4.614356000
H	-2.704751000	1.322314000	4.640681000
F	-0.263844000	-2.392093000	3.947938000
F	1.544119000	1.908578000	3.292821000

Int-1'_AlPdPCy$_3$_ M06L.log

SCF (M06L) = -2847.10200505
E(SCF)+ZPE(0 K) = -2845.891723
H(298 K) = -2845.825147
G(298 K) = -2845.993598
Lowest Frequency = 12.4671 cm^{-1}

Pd	-0.595890	0.031162	-0.755848
P	-2.196072	0.326114	0.900299
C	-2.161034	-1.187496	1.991111
H	-2.899941	-1.077948	2.801889
C	-0.788765	-1.428942	2.629494
H	-0.028189	-1.442379	1.829704
H	-0.521492	-0.598018	3.297145
C	-0.753381	-2.748471	3.396108
H	0.245172	-2.908483	3.823004
H	-1.450235	-2.696808	4.246906
C	-1.133849	-3.921343	2.501933
H	-1.097706	-4.863131	3.063181
H	-0.388833	-4.009451	1.697275
C	-2.506572	-3.705793	1.881376
H	-3.273736	-3.698359	2.671272
H	-2.762495	-4.531372	1.206043
C	-2.544179	-2.389205	1.119140
H	-3.530809	-2.240826	0.659152
H	-1.824715	-2.431586	0.285393
C	-4.033475	0.457363	0.511735
H	-4.143980	-0.259150	-0.321316
C	-5.037151	0.036121	1.591913
H	-4.948947	0.691121	2.468920
H	-4.821517	-0.978835	1.944491
C	-6.468177	0.096204	1.064154
H	-6.574915	-0.632952	0.245649
H	-7.172775	-0.210498	1.846258
C	-6.808841	1.485961	0.542674
H	-6.758712	2.205218	1.374361
H	-7.838849	1.519002	0.168820
C	-5.828998	1.902839	-0.546214
H	-6.057262	2.911153	-0.911433
H	-5.944182	1.225542	-1.403726
C	-4.386884	1.838069	-0.053004
H	-3.687689	2.103162	-0.858804
H	-4.253186	2.597215	0.732008
C	-1.746757	1.721851	2.055613
H	-0.806783	1.348769	2.497218
C	-1.372022	2.999458	1.290684
H	-0.629851	2.752473	0.517366
H	-2.250876	3.388220	0.757826
C	-0.842990	4.078254	2.230202
H	-0.625092	4.992881	1.665834
H	0.117463	3.744945	2.650185
C	-1.811156	4.361664	3.372495
H	-1.402439	5.124214	4.045852
H	-2.744078	4.779090	2.963842
C	-2.134250	3.085651	4.140529
H	-2.833422	3.290467	4.959921
H	-1.214145	2.700866	4.606319
C	-2.705403	2.019879	3.209561
H	-3.662333	2.383438	2.807130
H	-2.934318	1.102128	3.767417
Al	1.756867	-0.131912	-0.926745
N	3.178768	-1.413884	-0.457075
N	3.111223	1.283267	-1.173665
C	4.433584	-1.350873	-0.929859
C	4.972043	-0.192986	-1.507082
H	5.991093	-0.261940	-1.870264
C	4.386368	1.082047	-1.514426
C	5.325648	-2.553310	-0.820874
H	5.446598	-2.863190	0.222360
H	6.311115	-2.355912	-1.244509
H	4.883374	-3.410660	-1.339718
C	5.245811	2.252141	-1.888294

H	4.735305	2.903018	-2.604957
H	6.198892	1.928896	-2.309178
H	5.453229	2.873477	-1.009962
C	2.817047	-2.568268	0.319045
C	2.949149	-2.499536	1.723924
C	2.694209	-3.654969	2.466423
H	2.802885	-3.629960	3.547028
C	2.287997	-4.833846	1.852014
H	2.095305	-5.720817	2.449719
C	2.080350	-4.855881	0.481006
H	1.704244	-5.759726	0.006078
C	2.327732	-3.728770	-0.307796
C	3.332376	-1.202375	2.416128
H	2.849530	-0.389279	1.850631
C	2.820238	-1.128421	3.850311
H	1.747263	-1.332207	3.912244
H	2.997291	-0.132923	4.266754
H	3.337497	-1.838969	4.504487
C	4.838932	-0.934121	2.396493
H	5.386524	-1.756505	2.872217
H	5.069694	-0.018899	2.954434
H	5.231823	-0.803677	1.384534
C	1.978800	-3.767754	-1.781242
H	2.370838	-2.856367	-2.251404
C	0.456199	-3.749699	-1.929056
H	0.157079	-3.723051	-2.983068
H	0.025959	-2.871208	-1.430057
H	0.007308	-4.641832	-1.475499
C	2.583881	-4.966496	-2.507142
H	3.673069	-5.009201	-2.399745
H	2.354377	-4.924793	-3.576246
H	2.181282	-5.912213	-2.128286
C	2.633060	2.624159	-0.992773
C	1.890555	3.253823	-2.009394
C	1.384278	4.533589	-1.765199
H	0.809834	5.028071	-2.546147
C	1.592901	5.175412	-0.552522
H	1.187075	6.168917	-0.381211
C	2.315307	4.533986	0.445654
H	2.462846	5.025767	1.405989
C	2.843750	3.256841	0.249362
C	1.611040	2.594711	-3.343825
H	2.080980	1.602874	-3.337595
C	0.110934	2.391627	-3.539682
H	-0.415840	3.351629	-3.599391
H	-0.317967	1.820396	-2.704769
H	-0.094110	1.840874	-4.463324
C	2.212720	3.390327	-4.501467
H	1.766238	4.388871	-4.570340
H	2.031079	2.884859	-5.455230
H	3.294412	3.523353	-4.392716
C	3.578468	2.580206	1.390208
H	3.961124	1.615091	1.033969
C	2.620784	2.298736	2.547306
H	2.231109	3.233731	2.966320
H	3.132557	1.765290	3.355591
H	1.760464	1.696041	2.226617
C	4.769157	3.403362	1.878538
H	5.477365	3.621952	1.072889
H	5.313047	2.871934	2.666443
H	4.445556	4.363377	2.295940
H	-1.496276	-1.439118	-2.547383
C	-2.586328	-1.436282	-2.605830
C	-3.350456	-2.523769	-2.200580
C	-3.279290	-0.337198	-3.098722
C	-4.739058	-2.528547	-2.249090
C	-4.663077	-0.298681	-3.192665
C	-5.387827	-1.405287	-2.755414
H	-5.282854	-3.403190	-1.910318
H	-5.147651	0.585141	-3.592009
H	-6.472536	-1.389857	-2.807877
F	-2.720798	-3.621487	-1.742231
F	-2.576392	0.737049	-3.497621

Int-1_1,2,3-TriFB_AlPdAl _M06L.log

SCF (M06L) = 3140.49908072
E(SCF)+ZPE(0 K) = -3139.142413
H(298 K) = -3139.059981
G(298 K) = -3139.257126
Lowest Frequency = 19.4972cm-1

Pd	-0.019036	-0.386776	-0.932324
Al	1.904751	0.093406	0.383059
N	3.021317	1.710868	0.119734
N	2.455089	0.012031	2.294528
C	3.564456	2.490509	1.060916
C	3.627100	2.112655	2.408620
H	4.156074	2.786860	3.072989
C	3.225571	0.887894	2.955689
C	4.197461	3.795896	0.677237
H	4.937061	3.660073	-0.118403
H	4.679943	4.271282	1.532494
H	3.442997	4.483161	0.279015
C	3.767070	0.542822	4.315252
H	3.040880	0.027993	4.948170
H	4.127959	1.434526	4.830976
H	4.616895	-0.142005	4.203133
C	3.278758	2.025580	-1.259464
C	4.311183	1.307286	-1.906387
C	4.605054	1.627209	-3.232155
H	5.392075	1.086277	-3.748776
C	3.885471	2.599881	-3.916888
H	4.130074	2.829898	-4.950474
C	2.838161	3.252345	-3.283377
H	2.249862	3.988742	-3.828269
C	2.509502	2.977626	-1.952307
C	5.081747	0.216982	-1.180014
H	4.357935	-0.310131	-0.534007
C	5.677489	-0.830886	-2.112219
H	4.931381	-1.261771	-2.783820
H	6.115614	-1.644399	-1.525496
H	6.484807	-0.417259	-2.727109
C	6.174315	0.783396	-0.271486
H	6.899230	1.363431	-0.854858
H	6.721170	-0.028760	0.221833
H	5.775704	1.432162	0.513256
C	1.308232	3.660033	-1.332646
H	1.293755	3.411193	-0.265752
C	0.017327	3.115321	-1.945173
H	-0.860439	3.581087	-1.481812
H	-0.064009	2.028716	-1.798312
H	-0.019328	3.321867	-3.023513
C	1.350185	5.181398	-1.462986
H	2.272545	5.615021	-1.061080
H	0.503566	5.628864	-0.930491
H	1.275036	5.495794	-2.510179
C	2.156419	-1.227913	2.954923
C	0.944547	-1.351721	3.666465
C	0.715141	-2.537467	4.368192
H	-0.199106	-2.643366	4.946806
C	1.627329	-3.586305	4.336140
H	1.424286	-4.501089	4.886419
C	2.785420	-3.468150	3.580125
H	3.482341	-4.301790	3.527876
C	3.073600	-2.294062	2.878771
C	-0.056701	-0.212107	3.724073
H	0.039849	0.351188	2.783664
C	-1.506660	-0.673639	3.824540
H	-1.715889	-1.195764	4.765359
H	-1.788736	-1.338032	3.002526
H	-2.178711	0.191613	3.791039
C	0.252928	0.739418	4.881196
H	0.267257	0.198851	5.835358
H	-0.515447	1.517375	4.953285
H	1.218330	1.240436	4.765688
C	4.322488	-2.221036	2.020519
H	4.489846	-1.170207	1.746060
C	4.115267	-3.009635	0.724393
H	3.905106	-4.063861	0.940546

H	5.009774	-2.966585	0.093114
H	3.270959	-2.617548	0.142232
C	5.571809	-2.707696	2.750372
H	5.722599	-2.186689	3.701669
H	6.461824	-2.547895	2.133480
H	5.521884	-3.779911	2.968806
Al	-2.156526	0.095458	-0.024824
N	-3.369126	-1.384629	0.531365
N	-3.223963	1.399804	1.004264
C	-4.353175	-1.334110	1.429490
C	-4.649201	-0.168505	2.157299
H	-5.414840	-0.260998	2.919753
C	-4.166606	1.122973	1.920738
C	-5.239092	-2.525758	1.649392
H	-4.690266	-3.467091	1.568570
H	-6.022649	-2.554469	0.882912
H	-5.733405	-2.474631	2.621454
C	-4.761414	2.242322	2.725133
H	-4.000225	2.696677	3.369599
H	-5.578589	1.889793	3.355858
H	-5.132610	3.044476	2.079174
C	-3.236504	-2.536639	-0.318390
C	-4.100215	-2.684378	-1.422357
C	-3.913851	-3.776127	-2.275109
H	-4.572985	-3.893491	-3.133248
C	-2.895857	-4.693469	-2.057374
H	-2.763181	-5.535406	-2.731169
C	-2.033137	-4.516569	-0.983071
H	-1.218768	-5.218322	-0.830118
C	-2.172302	-3.439057	-0.105361
C	-5.159514	-1.658550	-1.776239
H	-5.236944	-0.931805	-0.956413
C	-4.733130	-0.895522	-3.031291
H	-4.635256	-1.576555	-3.884458
H	-3.762748	-0.402425	-2.889714
H	-5.472902	-0.132204	-3.297556
C	-6.539230	-2.281242	-1.979041
H	-7.291503	-1.505687	-2.156801
H	-6.859338	-2.868934	-1.112654
H	-6.552529	-2.950229	-2.846428
C	-1.229936	-3.293405	1.070867
H	-1.144403	-2.219862	1.291876
C	-1.807244	-3.998579	2.298762
H	-2.757976	-3.556150	2.617099
H	-1.112348	-3.936886	3.142041
H	-1.987330	-5.059409	2.086860
C	0.186533	-3.771137	0.778324
H	0.842717	-3.511090	1.615033
H	0.584881	-3.286762	-0.119430
H	0.246086	-4.858108	0.644571
C	-2.995453	2.771715	0.637813
C	-2.124282	3.596889	1.373666
C	-1.944507	4.919159	0.954879
H	-1.265656	5.560577	1.513836
C	-2.605760	5.423468	-0.154326
H	-2.460375	6.457134	-0.456562
C	-3.433818	4.588106	-0.893746
H	-3.924078	4.975709	-1.782011
C	-3.628136	3.253521	-0.532300
C	-1.330048	3.108034	2.568033
H	-1.678243	2.097803	2.824392
C	0.151678	3.009656	2.203235
H	0.543644	3.987140	1.891911
H	0.302153	2.308987	1.370869
H	0.754269	2.668968	3.051716
C	-1.515975	4.008105	3.789433
H	-0.982842	3.604297	4.656126
H	-2.569317	4.124903	4.064662
H	-1.116138	5.011622	3.608821
C	-4.496089	2.351054	-1.390620
H	-4.072907	1.335674	-1.324919
C	-4.469454	2.740143	-2.863844
H	-4.971114	3.696094	-3.048550
H	-4.989200	1.989294	-3.465222
H	-3.442489	2.818954	-3.236869
C	-5.932225	2.269765	-0.873173
H	-6.401328	3.260646	-0.863060
H	-5.979845	1.863475	0.142138
H	-6.538479	1.619941	-1.515146
H	-1.191677	-2.250745	-2.653166
C	-0.231788	-1.896855	-3.022348
C	-0.064651	-0.593146	-3.517702
C	0.853219	-2.768107	-3.062460
C	1.161281	-0.178232	-4.047893
C	2.073412	-2.373957	-3.597897
C	2.207809	-1.085030	-4.102088
H	1.311328	0.828682	-4.422866
F	0.741962	-4.025349	-2.608503
H	-0.913084	0.084673	-3.520683
F	3.112844	-3.218046	-3.620070
F	3.388417	-0.748459	-4.646050

Int-1_FB_AlPdAl _M06L.log

SCF (M06L) = 2942.05840275
E(SCF)+ZPE(0 K) = -2940.686552
H(298 K) = 2940.605639
G(298 K) = -2940.799938
Lowest Frequency = 17.2096 cm^{-1}

Pd	0.040261	-0.037629	1.146062
Al	-1.977184	-0.194130	-0.114412
N	-3.225795	1.316516	-0.429101
N	-2.630277	-1.082452	-1.775406
C	-3.864483	1.621228	-1.564742
C	-3.958539	0.728324	-2.640392
H	-4.566257	1.048002	-3.479611
C	-3.497230	-0.593318	-2.673052
C	-4.584429	2.931866	-1.690969
H	-5.304265	3.066878	-0.876859
H	-5.110315	3.006434	-2.643929
H	-3.879874	3.766960	-1.615524
C	-4.084413	-1.491511	-3.726205
H	-3.358390	-2.190683	-4.146978
H	-4.533681	-0.908398	-4.532187
H	-4.877259	-2.103759	-3.278652
C	-3.444116	2.161057	0.715003
C	-4.390955	1.731228	1.672064
C	-4.660883	2.571641	2.754245
H	-5.392517	2.266336	3.497632
C	-3.999511	3.784825	2.908576
H	-4.223628	4.422509	3.759371
C	-3.028774	4.158860	1.992137
H	-2.473447	5.082794	2.140673
C	-2.724306	3.357536	0.889071
C	-5.083709	0.385337	1.538493
H	-4.322278	-0.326632	1.172806
C	-5.593703	-0.162361	2.865777
H	-4.811716	-0.166816	3.630484
H	-5.947292	-1.190394	2.738187
H	-6.440375	0.418358	3.249189
C	-6.225017	0.400745	0.519893
H	-6.990195	1.131328	0.808148
H	-6.707925	-0.582581	0.472645
H	-5.886455	0.645943	-0.490158
C	-1.592127	3.760684	-0.032143
H	-1.614732	3.095185	-0.901865
C	-0.245911	3.555745	0.664879
H	0.581125	3.790403	-0.015790
H	-0.121209	2.514264	0.995402
H	-0.159544	4.204559	1.545543
C	-1.708383	5.197069	-0.537819
H	-2.668233	5.397178	-1.027141
H	-0.909816	5.407428	-1.258027
H	-1.603691	5.920135	0.278912
C	-2.283999	-2.471120	-1.885896
C	-1.110588	-2.833429	-2.580044
C	-0.841619	-4.193901	-2.746125
H	0.042609	-4.496025	-3.301800
C	-1.675272	-5.168398	-2.208191
H	-1.442397	-6.220715	-2.347310

C	-2.789647	-4.790498	-1.471986
H	-3.421226	-5.552697	-1.020489
C	-3.115802	-3.442836	-1.296233
C	-0.192384	-1.782023	-3.176569
H	-0.266568	-0.889490	-2.536861
C	1.275044	-2.195939	-3.204531
H	1.458909	-3.037402	-3.882672
H	1.646497	-2.475662	-2.214331
H	1.892836	-1.364352	-3.562732
C	-0.634220	-1.390822	-4.587904
H	-0.674619	-2.271192	-5.240779
H	0.077828	-0.684294	-5.029198
H	-1.619734	-0.916707	-4.603181
C	-4.308824	-3.066333	-0.437725
H	-4.523797	-2.000506	-0.596443
C	-3.967693	-3.251685	1.043657
H	-3.715365	-4.297740	1.255662
H	-4.818046	-2.974610	1.677650
H	-3.108646	-2.635593	1.339914
C	-5.569353	-3.847023	-0.800697
H	-5.817364	-3.757308	-1.863431
H	-6.425451	-3.483530	-0.223174
H	-5.463190	-4.914495	-0.578712
Al	2.101602	0.101231	-0.011411
N	3.383354	-1.428845	-0.057185
N	3.005987	0.935594	-1.564273
C	4.298257	-1.696564	-0.990180
C	4.464992	-0.898650	-2.134300
H	5.186255	-1.251292	-2.863333
C	3.908343	0.362310	-2.376209
C	5.246838	-2.846410	-0.810604
H	4.765270	-3.706760	-0.339379
H	6.070983	-2.551668	-0.150147
H	5.682706	-3.150136	-1.764292
C	4.377962	1.100664	-3.596340
H	3.553053	1.234586	-4.305088
H	5.180872	0.563030	-4.102524
H	4.729084	2.106262	-3.343306
C	3.401572	-2.158569	1.180448
C	4.353013	-1.822002	2.164617
C	4.324735	-2.505005	3.383191
H	5.052986	-2.247572	4.149898
C	3.376521	-3.486140	3.636214
H	3.370322	-4.009105	4.588614
C	2.422650	-3.781199	2.670149
H	1.666454	-4.534686	2.875517
C	2.404079	-3.122713	1.437714
C	5.340881	-0.684408	1.990251
H	5.298348	-0.335635	0.949790
C	4.933765	0.486396	2.886181
H	4.957724	0.193208	3.941938
H	3.915257	0.827309	2.660978
H	5.617196	1.333738	2.759946
C	6.780669	-1.099996	2.284235
H	7.470832	-0.276578	2.073089
H	7.093277	-1.962557	1.686861
H	6.912546	-1.370501	3.337592
C	1.368892	-3.493113	0.396524
H	1.211764	-2.610753	-0.239441
C	1.887569	-4.636945	-0.475862
H	2.790555	-4.353423	-1.028231
H	1.131333	-4.940479	-1.206435
H	2.134080	-5.511667	0.138026
C	0.004346	-3.824959	0.986611
H	-0.720378	-3.972217	0.179186
H	-0.359312	-2.998090	1.608047
H	0.014096	-4.744696	1.584640
C	2.692761	2.326436	-1.752347
C	1.730753	2.745506	-2.690702
C	1.476573	4.114712	-2.819290
H	0.728272	4.446223	-3.536844
C	2.148914	5.051332	-2.049248
H	1.942764	6.111327	-2.171271
C	3.066400	4.621896	-1.098522
H	3.565938	5.354981	-0.471855
C	3.340772	3.265171	-0.915858
C	0.913823	1.781218	-3.527236
H	1.320814	0.770168	-3.385698
C	-0.535019	1.770999	-3.038277
H	-0.982282	2.770709	-3.118900
H	-0.585881	1.466081	-1.984149
H	-1.154890	1.085062	-3.625143
C	0.966551	2.120246	-5.016767
H	0.417071	1.378152	-5.604955
H	1.991570	2.163016	-5.399438
H	0.505311	3.092658	-5.219924
C	4.312660	2.822745	0.162897
H	3.962880	1.845709	0.533863
C	4.326864	3.762293	1.362507
H	4.765819	4.736293	1.120762
H	4.927369	3.336707	2.171312
H	3.315414	3.932024	1.747106
C	5.723326	2.612616	-0.386958
H	6.115791	3.539253	-0.822154
H	5.749569	1.838780	-1.160967
H	6.408391	2.302482	0.410905
H	1.112494	1.219695	3.416161
C	0.229753	0.622658	3.621793
C	-0.967083	1.246523	3.967203
C	0.269039	-0.780468	3.613936
C	-2.102740	0.533755	4.324715
C	-0.869769	-1.516286	3.960449
C	-2.043018	-0.859288	4.323509
H	-0.829632	-2.602294	3.950980
F	-1.002682	2.593189	3.993483
H	-3.006668	1.073934	4.588751
H	-2.926921	-1.433008	4.591511
H	1.204232	-1.285427	3.378782

Int-2_AlPdAl _M06L.log

SCF (M06L) = -3041.37625001
E(SCF)+ZPE(0 K) = -3040.012552
H(298 K) = -3039.930524
G(298 K) = -3040.129238
Lowest Frequency = 13.9835 cm^{-1}

Pd	-0.467156000	-0.188433000	-0.443546000
Al	2.448543000	0.337359000	0.543809000
N	3.737484000	1.699154000	0.160646000
N	3.682349000	-0.819017000	1.444700000
C	4.701381000	1.946600000	1.062699000
C	5.064952000	1.041083000	2.069339000
H	5.829186000	1.376205000	2.760981000
C	4.655664000	-0.297104000	2.195798000
C	5.460682000	3.235983000	0.976562000
H	4.772750000	4.086351000	0.927902000
H	6.059437000	3.279784000	0.061122000
H	6.127615000	3.361667000	1.829899000
C	5.361767000	-1.164286000	3.193283000
H	6.211951000	-0.649488000	3.641842000
H	5.709799000	-2.093026000	2.730430000
H	4.673223000	-1.462010000	3.992209000
C	3.632082000	2.551803000	-0.988051000
C	4.499533000	2.332968000	-2.078409000
C	4.347748000	3.130746000	-3.214274000
H	5.006131000	2.967315000	-4.065619000
C	3.366963000	4.112616000	-3.280040000
C	2.519578000	4.310868000	-2.197950000
H	1.745845000	5.073944000	-2.250094000
C	2.634313000	3.545244000	-1.034418000
C	3.391976000	-2.221249000	1.499939000
C	2.542097000	-2.722407000	2.502685000
C	2.215929000	-4.081553000	2.467738000
H	1.553705000	-4.485033000	3.231564000

C	2.695593000	-4.910647000	1.463992000
C	3.504166000	-4.387661000	0.460300000
H	3.841550000	-5.035854000	-0.343080000
C	3.861109000	-3.038283000	0.449195000
F	1.501903000	0.972385000	1.780712000
C	1.127500000	-1.904816000	-0.880418000
C	1.646368000	-0.576627000	-1.010937000
C	0.810613000	-2.652906000	-2.019320000
C	1.675885000	-0.054631000	-2.337160000
C	0.920085000	-2.148925000	-3.300415000
C	1.338095000	-0.820745000	-3.447517000
H	2.009260000	0.970276000	-2.497543000
H	0.674204000	-2.774270000	-4.152048000
H	1.407952000	-0.390271000	-4.443216000
F	0.417247000	-3.937178000	-1.849167000
H	1.119033000	-2.424799000	0.075538000
H	2.419486000	-5.961554000	1.447050000
H	3.261296000	4.719621000	-4.175047000
C	5.552132000	1.239406000	-2.080182000
C	6.958256000	1.795310000	-2.302425000
C	5.234112000	0.185312000	-3.140791000
H	5.536614000	0.740604000	-1.102799000
H	7.228766000	2.555454000	-1.562664000
H	7.702709000	0.994300000	-2.244258000
H	7.051372000	2.256587000	-3.292102000
H	4.252000000	-0.271074000	-2.978620000
H	5.232256000	0.626478000	-4.144367000
H	5.988084000	-0.610125000	-3.134189000
C	1.659282000	3.758472000	0.105706000
C	0.287679000	3.196597000	-0.267192000
C	1.537522000	5.223802000	0.516184000
H	2.017296000	3.197579000	0.976267000
H	0.336071000	2.141213000	-0.575856000
H	-0.406841000	3.248891000	0.577828000
H	-0.151812000	3.757904000	-1.101675000
H	2.506014000	5.663457000	0.776801000
H	1.100248000	5.834378000	-0.282551000
H	0.877321000	5.315575000	1.385253000
C	4.680088000	-2.448335000	-0.684769000
C	4.580474000	-3.255798000	-1.972872000
C	6.145005000	-2.245625000	-0.293924000
H	4.259356000	-1.452901000	-0.893380000
H	3.538379000	-3.435086000	-2.256474000
H	5.062456000	-2.717110000	-2.793871000
H	5.082426000	-4.226457000	-1.887825000
H	6.257846000	-1.546258000	0.540003000
H	6.609453000	-3.195791000	-0.004977000
H	6.715961000	-1.840603000	-1.137832000
C	1.898983000	-1.824579000	3.541400000
C	2.081436000	-2.354104000	4.961884000
C	0.412952000	-1.638531000	3.225653000
H	2.364255000	-0.834197000	3.481983000
H	3.135578000	-2.514132000	5.213376000
H	1.664780000	-1.652209000	5.690793000
H	1.566185000	-3.310809000	5.103355000
H	0.245503000	-1.276399000	2.201399000
H	-0.128894000	-2.585734000	3.341789000
H	-0.038294000	-0.906229000	3.902868000
Al	-2.723457000	0.033226000	0.201795000
N	-4.151894000	-1.305815000	0.161326000
C	-5.364208000	-1.219635000	0.721439000
C	-5.782960000	-0.098895000	1.451647000
C	-5.133397000	1.137915000	1.537644000
N	-3.924436000	1.359613000	1.000164000
C	-5.852952000	2.256301000	2.233870000
H	-5.925015000	3.139518000	1.590935000

H	-5.305502000	2.576220000	3.127391000
H	-6.857015000	1.954236000	2.534382000
H	-6.761009000	-0.163154000	1.914967000
C	-6.354555000	-2.330812000	0.530799000
H	-5.883639000	-3.313508000	0.620178000
H	-6.782116000	-2.285626000	-0.477626000
H	-7.175039000	-2.253782000	1.246201000
C	-3.884232000	-2.401863000	-0.731538000
C	-4.398454000	-2.348136000	-2.041217000
C	-4.113320000	-3.403478000	-2.910286000
C	-3.327962000	-4.471625000	-2.503096000
C	-2.793611000	-4.486956000	-1.220533000
C	-1.233862000	-4.408700000	1.185358000
C	-3.047300000	-3.457319000	-0.311709000
C	-2.425865000	-3.466878000	1.073559000
H	-2.144248000	-5.306091000	-0.929009000
H	-3.111831000	-5.284765000	-3.190578000
H	-4.498758000	-3.372019000	-3.927379000
H	-2.044096000	-2.446871000	1.252550000
H	-0.489578000	-4.224146000	0.405961000
H	-0.743109000	-4.280880000	2.154606000
H	-1.539714000	-5.459906000	1.119902000
C	-3.442839000	-3.784911000	2.170460000
H	-4.246583000	-3.046188000	2.224843000
H	-3.897988000	-4.769367000	2.007184000
H	-2.952955000	-3.806446000	3.150074000
C	-4.220238000	-0.318241000	-3.466425000
H	-4.714929000	0.596976000	-3.812456000
H	-3.294548000	-0.038430000	-2.947457000
H	-3.931272000	-0.897427000	-4.350831000
C	-5.148367000	-1.138186000	-2.566791000
H	-5.432664000	-0.501758000	-1.718433000
C	-6.429294000	-1.503718000	-3.311951000
H	-6.974494000	-0.600590000	-3.605196000
H	-6.217565000	-2.064045000	-4.228974000
H	-7.100723000	-2.118223000	-2.703016000
C	-3.425251000	2.707532000	0.973113000
C	-2.822794000	3.270850000	2.112860000
C	-2.377820000	4.593528000	2.039728000
C	-2.505042000	5.332083000	0.871076000
C	-3.054015000	4.742574000	-0.260918000
C	-3.511902000	3.423228000	-0.239831000
H	-3.117298000	5.313327000	-1.183329000
C	-4.096718000	2.784299000	-1.486144000
C	-5.623313000	2.866275000	-1.504455000
H	-6.071457000	2.350662000	-0.649011000
H	-6.025980000	2.404420000	-2.413512000
H	-5.958917000	3.909734000	-1.483076000
H	-3.834807000	1.714968000	-1.458226000
C	-3.505521000	3.337931000	-2.775911000
H	-2.412065000	3.291705000	-2.766090000
H	-3.857365000	2.755499000	-3.632678000
H	-3.802715000	4.377365000	-2.953526000
H	-2.152527000	6.359606000	0.833769000
H	-1.907082000	5.042558000	2.911608000
C	-1.105154000	1.965027000	3.331391000
C	-2.554438000	2.453368000	3.359590000
H	-3.202971000	1.568207000	3.335486000
H	-0.885689000	1.361270000	2.442823000
H	-0.873412000	1.363578000	4.217462000
H	-0.409185000	2.813170000	3.314400000
H	-2.165160000	4.049168000	4.797660000
C	-2.854335000	3.210883000	4.649702000
H	-2.742169000	2.550969000	5.515232000
H	-3.871816000	3.617166000	4.664805000

Int-2'_ AlPdPCy$_3$ _M06L.log

SCF (M06L) = -2847.20513757
E(SCF)+ZPE(0 K) = -2845.993470
H(298 K) = -2845.927670
G(298 K) = -2846.092481
Lowest Frequency = 9.4237 cm^{-1}

Pd	1.011027	-0.022694	-0.790899
Al	-1.618309	-0.059821	0.610329
N	-2.751368	-1.569021	0.916854
N	-2.900321	1.261500	1.136834
C	-3.604561	-1.510407	1.956706
C	-3.967297	-0.315373	2.594123
H	-4.614271	-0.415924	3.458166
C	-3.723645	0.997952	2.152874
C	-4.248372	-2.771853	2.451661
H	-5.094476	-3.055061	1.817076
H	-4.627029	-2.639183	3.466254
H	-3.548147	-3.611837	2.431253
C	-4.433091	2.123992	2.841362
H	-5.189912	1.758263	3.536031
H	-4.905104	2.794970	2.117261
H	-3.715551	2.735357	3.400962
C	-2.692103	-2.772388	0.141540
C	-3.784123	-3.116997	-0.681711
C	-3.701243	-4.286024	-1.442664
H	-4.536060	-4.556343	-2.086268
C	-2.569218	-5.085430	-1.411747
C	-1.483248	-4.708806	-0.630182
H	-0.589598	-5.324484	-0.634768
C	-1.513463	-3.553198	0.153306
C	-2.790735	2.585062	0.598679
C	-1.928897	3.522422	1.196136
C	-1.798861	4.776145	0.590550
H	-1.131159	5.510458	1.036488
C	-2.486057	5.087660	-0.573655
C	-3.304058	4.134257	-1.170002
H	-3.811464	4.373510	-2.100144
C	-3.463927	2.866649	-0.608727
F	-0.529591	-0.014083	1.904573
C	-0.601991	1.489656	-1.694772
C	-1.112922	0.218934	-1.283055
C	-0.387311	1.760550	-3.049655
C	-1.288214	-0.742951	-2.319221
C	-0.643192	0.834896	-4.043791
C	-1.087575	-0.435513	-3.661359
H	-1.624240	-1.746614	-2.063653
H	-0.475563	1.098147	-5.082882
H	-1.268095	-1.189453	-4.423132
F	0.091099	2.976795	-3.390335
P	3.149731	-0.019103	0.003032
C	3.120640	0.265311	1.845742
C	2.554610	-0.966815	2.558141
C	4.364773	0.809083	2.553784
H	2.344516	1.042270	1.930061
C	2.272391	-0.672823	4.026270
H	3.268700	-1.803762	2.483328
H	1.632995	-1.276541	2.048868
C	4.063336	1.090892	4.024718
H	5.195076	0.092247	2.486469
H	4.711568	1.728495	2.065612
C	3.514658	-0.142344	4.732625
H	1.896702	-1.572046	4.529372
H	1.464922	0.071486	4.083054
H	4.962746	1.461172	4.530624
H	3.319950	1.899986	4.086052
H	3.296717	0.084066	5.782762
H	4.288242	-0.925493	4.740606
C	4.158864	1.408918	-0.658421
C	5.685237	1.297338	-0.598579
C	3.718115	1.783336	-2.079922
H	3.851863	2.241164	0.001387
C	6.349893	2.593377	-1.055964
H	6.006977	0.480888	-1.262603
H	6.026191	1.027542	0.406810
C	4.389928	3.066965	-2.553374
H	3.973327	0.958034	-2.763055
H	2.625050	1.874312	-2.115919
C	5.908458	2.974787	-2.463423
H	7.440832	2.497479	-1.004214
H	6.077656	3.399357	-0.357534
H	4.075236	3.302543	-3.576320
H	4.042378	3.901379	-1.925996
H	6.373479	3.918547	-2.770872
H	6.266239	2.210345	-3.169299
C	4.079955	-1.614878	-0.262355
C	4.209478	-1.913469	-1.760644
C	5.395754	-1.900397	0.468423
H	3.345535	-2.336793	0.130289
C	4.671095	-3.346796	-1.999318
H	4.935739	-1.218119	-2.210621
H	3.248908	-1.722411	-2.258626
C	5.831509	-3.344840	0.229340
H	6.185080	-1.223524	0.118245
H	5.285873	-1.720209	1.543625
C	5.969147	-3.645440	-1.258262
H	4.785161	-3.536822	-3.072797
H	3.888281	-4.034369	-1.644739
H	6.771535	-3.549472	0.754910
H	5.079075	-4.019911	0.666420
H	6.272480	-4.687003	-1.414802
H	6.774646	-3.023276	-1.676576
H	-0.495923	2.317913	-0.996152
H	-2.369500	6.066264	-1.030874
H	-2.521394	-5.991105	-2.009903
C	-4.989827	-2.214715	-0.874230
C	-6.319988	-2.944723	-0.697598
C	-4.927307	-1.582708	-2.266314
H	-4.948654	-1.401535	-0.138698
H	-6.396224	-3.455907	0.267238
H	-7.154966	-2.240325	-0.769511
H	-6.467866	-3.700846	-1.476375
H	-3.995377	-1.028073	-2.418473
H	-4.980398	-2.354712	-3.042778
H	-5.766566	-0.896158	-2.424495
C	-0.309816	-3.146076	0.983364
C	0.977724	-3.846684	0.569566
C	-0.550358	-3.323055	2.482428
H	-0.126072	-2.070745	0.808840
H	1.202013	-3.690050	-0.491975
H	1.813997	-3.446907	1.152259
H	0.942613	-4.925443	0.761035
H	-1.382025	-2.713347	2.842636
H	-0.759104	-4.373032	2.720708
H	0.337735	-3.019558	3.046674
C	-4.315855	1.809465	-1.284648
C	-4.378595	1.977086	-2.797636
C	-5.721515	1.753606	-0.684250
H	-3.837897	0.839425	-1.084500
H	-3.376448	2.043737	-3.233833

H	-4.886169	1.121679	-3.253022
H	-4.938410	2.872443	-3.090217
H	-5.702942	1.495507	0.379430
H	-6.230718	2.718894	-0.787915
H	-6.333068	0.999094	-1.192605
C	-1.075510	3.185117	2.402343
C	-1.191076	4.231029	3.508595
C	0.383383	3.013064	1.973143
H	-1.403719	2.219830	2.804399
H	-2.229896	4.393607	3.815577
H	-0.623328	3.922225	4.391659
H	-0.792138	5.200692	3.190797
H	0.492725	2.233185	1.207484
H	0.785445	3.949020	1.565776
H	1.004350	2.720149	2.827668

Int-2_1,2,3-TriFB_AlPdAl _M06L.log

SCF (M06L) = -3140.59533963
E(SCF)+ZPE(0 K) = -3139.238015
H(298 K) = -3139.156216
G(298 K) = -3139.351137
Lowest Frequency = 15.7892 cm^{-1}

Pd	-0.182968	0.265928	-0.028697
Al	3.283036	0.192959	-0.733024
N	3.595893	-1.572360	-0.057462
N	3.925889	1.119928	0.816448
C	4.710635	-1.765007	0.665212
C	5.438884	-0.709709	1.240819
H	6.359438	-0.985149	1.742622
C	5.006539	0.609479	1.430500
C	5.190717	-3.158578	0.961151
H	4.579761	-3.617875	1.744724
H	6.224568	-3.140272	1.309629
H	5.114005	-3.815511	0.093012
C	5.771642	1.443852	2.414240
H	5.795625	2.497239	2.123832
H	6.792417	1.071925	2.518017
H	5.304087	1.398538	3.403265
C	2.824962	-2.743136	-0.373029
C	1.896466	-3.202064	0.580429
C	1.284633	-4.437250	0.351107
H	0.565248	-4.817128	1.071142
C	1.559301	-5.169750	-0.796745
C	2.406642	-4.649825	-1.767389
H	2.570874	-5.203631	-2.687828
C	3.049483	-3.421742	-1.586597
C	3.386695	2.357245	1.301378
C	3.525695	3.524974	0.517794
C	2.982613	4.714315	1.006802
H	3.080580	5.623432	0.421284
C	2.303974	4.754871	2.219833
C	2.148027	3.592341	2.958993
H	1.588611	3.616126	3.892627
C	2.681688	2.376231	2.521813
C	1.527544	0.815043	-1.385048
C	1.064706	0.236780	-2.583669
C	0.892172	2.057523	-1.053617
C	0.159936	0.845259	-3.436761
C	-0.015624	2.689811	-1.924061
H	1.226580	2.613980	-0.181202
C	-0.369513	2.095040	-3.125756
H	-0.453070	3.644267	-1.642169
H	-1.075686	2.556444	-3.809867
F	4.438636	0.370744	-1.943755
H	1.080448	-6.134060	-0.952583
H	1.884320	5.691084	2.578571
C	4.219367	3.482096	-0.830995
C	5.739024	3.382802	-0.697339
C	3.842080	4.642574	-1.742551
H	3.891811	2.569616	-1.345359
H	6.046859	2.468905	-0.183516
H	6.206963	3.367344	-1.686118
H	6.139626	4.241978	-0.146038
H	2.756758	4.748943	-1.838064
H	4.246197	5.595888	-1.382708
H	4.252362	4.476984	-2.742303
C	2.423370	1.131870	3.351476
C	0.935989	0.780665	3.338130
C	2.894786	1.288629	4.797827
H	2.967672	0.291538	2.902095
H	0.549008	0.619595	2.317709
H	0.745195	-0.129245	3.919084
H	0.342089	1.586357	3.785631
H	3.941880	1.597978	4.874022
H	2.298421	2.040039	5.327474
H	2.783556	0.345083	5.342023
C	3.941305	-2.843376	-2.672474
C	3.492510	-3.263549	-4.069069
C	5.423860	-3.170191	-2.482539
H	3.861634	-1.753523	-2.619854
H	2.417629	-3.121114	-4.211054
H	4.014804	-2.667655	-4.822931
H	3.724935	-4.315028	-4.275205
H	5.852562	-2.657265	-1.617643
H	5.586083	-4.248798	-2.363182
H	5.993688	-2.843897	-3.358206
C	1.520920	-2.364762	1.792945
C	2.458940	-2.536658	2.991002
C	0.087040	-2.624619	2.238096
H	1.575141	-1.308089	1.476010
H	3.452723	-2.111525	2.825109
H	2.038928	-2.034559	3.870250
H	2.575344	-3.596995	3.248093
H	-0.609826	-2.591510	1.394432
H	-0.020268	-3.597261	2.734745
H	-0.224746	-1.856623	2.951036
Al	-2.529123	0.067500	0.349420
N	-3.929026	1.382223	-0.109988
N	-3.872982	-1.367068	0.488907
C	-5.202066	-1.260347	0.350345
C	-5.849867	-0.026408	0.210837
C	-5.253672	1.226765	0.025636
H	-6.933944	-0.047340	0.224202
C	-6.058385	-2.493923	0.367229
H	-5.654911	-3.255369	-0.307178
H	-7.083240	-2.264328	0.072792
H	-6.082128	-2.954009	1.360813
H	-5.921492	3.082560	0.838740
H	-7.204452	2.138922	0.056404
C	-6.154694	2.428526	-0.009568
H	-6.011662	3.035477	-0.906520
C	-3.325468	-2.670936	0.746814
C	-3.444536	-3.215693	2.044455
C	-2.915137	-4.485034	2.278249
C	-2.273161	-5.194993	1.268671
C	-2.127509	-4.625922	0.012399
C	-2.635947	-3.354586	-0.272360
H	-1.595665	-5.164620	-0.769407
H	-1.871073	-6.185017	1.469846
H	-2.998619	-4.922832	3.269684
C	-4.058358	-2.414956	3.179069
H	-4.811645	-1.738788	2.756719
C	-2.992575	-1.536356	3.837864
H	-2.489249	-0.888303	3.107419
H	-3.431059	-0.900939	4.614786
H	-2.218129	-2.156719	4.303829
H	-5.486941	-3.959620	3.769988
C	-4.760496	-3.276741	4.222645
H	-5.291915	-2.646402	4.941713
H	-4.051079	-3.882653	4.796258
H	-2.735419	-1.717545	-1.614724
C	-2.438786	-2.769940	-1.654902
C	-3.341048	-3.451470	-2.681855
H	-3.121758	-4.523233	-2.751930
H	-4.401316	-3.346120	-2.424615
C	-0.976369	-2.791909	-2.088200
H	-0.850514	-2.249485	-3.031796

H	-0.338460	-2.303368	-1.338523
H	-0.599253	-3.810343	-2.236290
H	-3.194118	-3.016659	-3.675960
C	-3.470514	2.649910	-0.608199
C	-2.982378	3.628504	0.277680
C	-2.614033	4.874625	-0.238199
C	-2.721011	5.153481	-1.592942
C	-3.155725	4.157636	-2.458475
C	-3.519312	2.888143	-1.999620
C	-3.938243	1.875773	-3.059978
H	-3.202209	4.353120	-3.529099
H	-2.442918	6.130844	-1.977352
H	-2.240831	5.636533	0.443382
C	-2.829278	3.385984	1.765354
H	-3.207411	2.379618	1.991493
C	-3.640896	4.381615	2.592708
H	-4.703309	4.368667	2.327734
H	-3.558048	4.156693	3.660691
H	-3.281653	5.406421	2.446722
H	-1.232503	3.249008	3.233557
C	-1.353339	3.426427	2.158686
H	-0.780510	2.660846	1.618765
H	-0.909040	4.403071	1.931901
H	-3.465762	2.249439	-3.980045
H	-5.845002	2.879465	-3.455474
C	-5.444858	1.867952	-3.330073
H	-5.661891	1.305182	-4.243792
H	-5.993005	1.378753	-2.517525
H	-3.410453	-0.076739	-3.832988
C	-3.434613	0.446221	-2.870328
H	-4.094993	-0.129119	-2.210236
H	-2.416868	0.418609	-2.458370
F	1.595461	-0.948752	-2.974650
F	-0.213155	0.214771	-4.569348

Int-2_FB_AlPdAl _M06L.log

SCF (M06L) = -2942.14171088
E(SCF)+ZPE(0 K) = -2940.767318
H(298 K) = -2940.687527
G(298 K) = -2940.878244
Lowest Frequency = 17.6925 cm^{-1}

Pd	0.065501	-0.217398	-0.379744
Al	-3.289890	-0.393433	-0.696614
N	-3.720669	1.286871	0.104507
N	-3.563784	-1.444931	0.883320
C	-4.783495	1.294225	0.925539
C	-5.288209	0.123243	1.512139
H	-6.197251	0.232716	2.093439
C	-4.644363	-1.122134	1.610445
C	-5.442341	2.594975	1.287662
H	-4.800333	3.190835	1.943429
H	-6.386689	2.420198	1.805188
H	-5.625103	3.211519	0.403779
C	-5.204955	-2.083928	2.616895
H	-4.870274	-3.108965	2.446444
H	-6.297011	-2.053482	2.584169
H	-4.911023	-1.798361	3.631773
C	-3.111564	2.545125	-0.206782
C	-2.192787	3.089827	0.712348
C	-1.718428	4.382475	0.469667
H	-1.009011	4.828672	1.161623
C	-2.106926	5.088814	-0.661894
C	-2.926106	4.486950	-1.609201
H	-3.168186	5.020431	-2.524405
C	-3.434848	3.199932	-1.411784
C	-2.761573	-2.572860	1.251497
C	-2.710176	-3.690946	0.386877
C	-1.881102	-4.758688	0.734294
H	-1.822897	-5.622637	0.078959
C	-1.129893	-4.738860	1.903446
C	-1.194959	-3.637230	2.741438
H	-0.594951	-3.613137	3.649515
C	-1.996017	-2.533856	2.435238
C	-1.628745	-0.531160	-1.736271
C	-1.346941	0.501768	-2.680099
C	-0.965775	-1.771655	-1.989709
C	-0.629822	0.275985	-3.850648
H	-1.749928	1.498442	-2.505809
C	-0.249896	-2.002269	-3.173668
H	-1.105839	-2.610295	-1.308845
C	-0.118747	-0.996127	-4.121659
H	0.193948	-2.979570	-3.346095
H	0.413138	-1.185793	-5.051866
F	-4.623873	-0.728302	-1.676308
H	-1.729691	6.094802	-0.830375
H	-0.492442	-5.582541	2.156639
C	-3.557851	-3.746867	-0.871073
C	-5.031872	-3.988062	-0.543994
C	-3.070352	-4.766863	-1.892452
H	-3.506702	-2.769392	-1.367823
H	-5.446591	-3.194351	0.082034
H	-5.626334	-4.018175	-1.461932
H	-5.162071	-4.943127	-0.021357
H	-2.004606	-4.648369	-2.116255
H	-3.230349	-5.795802	-1.550130
H	-3.623331	-4.647895	-2.828079
C	-1.960031	-1.342097	3.373534
C	-0.565945	-0.716947	3.395458
C	-2.365303	-1.721403	4.799219
H	-2.659299	-0.580482	3.005734
H	-0.221409	-0.434596	2.389047
H	-0.551912	0.175147	4.032326
H	0.166884	-1.419884	3.808922
H	-3.320359	-2.252980	4.842952
H	-1.613719	-2.374426	5.257237
H	-2.444124	-0.828277	5.428240
C	-4.264547	2.514253	-2.485122
C	-3.909095	2.997074	-3.887910
C	-5.771418	2.618342	-2.252281
H	-4.028937	1.442790	-2.451082
H	-2.826253	2.997127	-4.056945
H	-4.367130	2.343808	-4.635766
H	-4.275330	4.012410	-4.078126
H	-6.085453	2.062435	-1.365573
H	-6.085540	3.663384	-2.141126
H	-6.314873	2.193165	-3.101632
C	-1.670367	2.275624	1.886324
C	-2.547423	2.342190	3.140211
C	-0.243788	2.665962	2.248986
H	-1.643833	1.224594	1.548952
H	-3.501656	1.820643	3.027101
H	-2.027483	1.876176	3.985098
H	-2.753917	3.382668	3.420917
H	0.405110	2.682466	1.368015
H	-0.194815	3.650414	2.729986
H	0.172751	1.941159	2.952567
Al	2.387685	0.065937	0.121296
N	3.843847	-1.171325	-0.393177
N	3.670309	1.559408	0.195442
C	4.994313	1.528532	-0.026840
C	5.695154	0.346917	-0.290478
C	5.158658	-0.941011	-0.420781
H	6.771587	0.435457	-0.386146
C	5.793040	2.799633	0.043692
H	5.298534	3.611144	-0.498175
H	6.794278	2.655013	-0.364715
H	5.895305	3.144723	1.078574
H	6.192894	-2.607206	0.408580
H	7.118777	-1.741162	-0.824234
C	6.119434	-2.086889	-0.554474
H	5.786838	-2.829644	-1.282498
C	3.089518	2.812969	0.586937
C	3.259471	3.260954	1.914155
C	2.704871	4.491266	2.269450
C	1.989083	5.248256	1.348910
C	1.786795	4.765698	0.062966
C	2.317935	3.537459	-0.342382
H	1.189322	5.341928	-0.638830

H	1.567620	6.206380	1.643146
H	2.826817	4.858003	3.285529
C	3.959055	2.397315	2.949393
H	4.701952	1.776135	2.433909
C	2.958874	1.440878	3.604642
H	2.409234	0.847743	2.860526
H	3.463756	0.747762	4.286690
H	2.212629	1.999992	4.181433
H	5.385775	3.932649	3.567464
C	4.701183	3.202027	4.010715
H	5.286101	2.537483	4.653467
H	4.012485	3.748989	4.663413
H	2.054240	1.914776	-1.680243
C	2.073425	3.012443	-1.743570
C	3.207190	3.395524	-2.692942
H	3.318799	4.484725	-2.754716
H	4.166667	2.977277	-2.369597
C	0.714716	3.417613	-2.299328
H	0.525922	2.890384	-3.240519
H	-0.088991	3.153724	-1.601858
H	0.646452	4.492202	-2.506963
H	3.009770	3.021309	-3.703078
C	3.355105	-2.477661	-0.727839
C	3.125003	-3.416622	0.294320
C	2.536837	-4.638048	-0.039790
C	2.208454	-4.936524	-1.354824
C	2.471867	-4.006681	-2.352422
C	3.028622	-2.754925	-2.070253
C	3.283321	-1.827323	-3.253488
H	2.226232	-4.240683	-3.387400
H	1.751607	-5.890596	-1.603733
H	2.335910	-5.362465	0.747544
C	3.478161	-3.131761	1.740124
H	3.986839	-2.159284	1.782306
C	4.431075	-4.181680	2.309116
H	5.341874	-4.280508	1.709358
H	4.725254	-3.927188	3.332410
H	3.957004	-5.168955	2.343033
H	2.446013	-2.715750	3.612921
C	2.213863	-3.033394	2.589365
H	1.499314	-2.324532	2.155391
H	1.712099	-4.006307	2.645628
H	2.616820	-2.210861	-4.040158
H	5.009249	-3.006684	-3.911911
C	4.713043	-1.960378	-3.784132
H	4.808514	-1.460822	-4.753900
H	5.431104	-1.482486	-3.108425
H	2.831877	0.117400	-4.075110
C	2.929012	-0.349567	-3.087673
H	3.705049	0.207504	-2.548566
H	1.968374	-0.215352	-2.572900
H	-0.481000	1.087641	-4.560458

Int-3_AlPdAl _M06L.log

SCF (M06L) = -3041.27753876
E(SCF)+ZPE(0 K) = -3039.916047
H(298 K) = -3039.835605
G(298 K) = -3040.02618
Lowest Frequency = 19.7929 cm^{-1}

Pd	- 0.005080000	0.624897000	0.967767000
Al	-1.693772000	-0.201880000	-0.417780000
N	-2.794423000	1.122439000	-1.331568000
N	-2.501333000	-1.675729000	-1.409585000
C	-3.457958000	0.946302000	-2.476766000
C	-3.630906000	-0.319424000	-3.057584000
H	-4.213365000	-0.354173000	-3.971208000
C	-3.293185000	-1.551037000	-2.491741000
C	-4.110754000	2.115666000	-3.149621000
H	-4.757753000	2.654853000	-2.450582000
H	-4.700586000	1.800235000	-4.011232000
H	-3.358871000	2.836864000	-3.486178000
C	-3.929281000	-2.769003000	-3.099034000
H	-3.268250000	-3.638236000	-3.106251000
H	-4.268133000	-2.562188000	-4.115577000
H	-4.808662000	-3.057493000	-2.510261000
C	-2.858099000	2.409194000	-0.680312000
C	-3.731452000	2.547388000	0.422002000
C	-3.770823000	3.781300000	1.071549000
H	-4.415368000	3.910539000	1.935418000
C	-2.967324000	4.839022000	0.661568000
H	-3.000478000	5.783120000	1.198403000
C	-2.100026000	4.673482000	-0.405922000
H	-1.443369000	5.489016000	-0.701759000
C	-2.024385000	3.460446000	-1.095216000
C	-4.579381000	1.383462000	0.907877000
H	-3.921674000	0.496389000	0.923737000
C	-5.104894000	1.565972000	2.326100000
H	-4.310515000	1.824939000	3.029887000
H	-5.576451000	0.641244000	2.672644000
H	-5.870235000	2.349822000	2.369062000
C	-5.753305000	1.069689000	-0.023808000
H	-6.410540000	1.941925000	-0.121285000
H	-6.355460000	0.250693000	0.387824000
H	-5.440215000	0.769111000	-1.027095000
C	-1.001624000	3.300273000	-2.200383000
H	-1.194268000	2.348438000	-2.710993000
C	0.405717000	3.230703000	-1.611284000
H	1.145472000	3.063178000	-2.399573000
H	0.486829000	2.426923000	-0.867662000
H	0.656783000	4.164427000	-1.095694000
C	-1.076117000	4.409756000	-3.247931000
H	-2.077383000	4.517998000	-3.679772000
H	-0.374519000	4.207282000	-4.065211000
H	-0.801483000	5.380546000	-2.821126000
C	-2.368271000	-2.994609000	-0.849523000
C	-1.226505000	-3.764260000	-1.146400000
C	-1.163058000	-5.066450000	-0.642504000
H	-0.298957000	-5.683908000	-0.875849000
C	-2.184348000	-5.588774000	0.141504000
H	-2.114445000	-6.604677000	0.520383000
C	-3.285463000	-4.801774000	0.452324000
H	-4.069902000	-5.201687000	1.090574000
C	-3.398317000	-3.495303000	-0.028495000
C	-0.131787000	-3.242394000	-2.056659000
H	-0.158241000	-2.144318000	-2.009757000
C	1.267037000	-3.674258000	-1.632952000
H	1.409806000	-4.758750000	-1.699613000
H	1.495816000	-3.374145000	-0.604966000
H	2.018324000	-3.218031000	-2.287828000
C	-0.384905000	-3.653874000	-3.507637000
H	-0.460163000	-4.744046000	-3.596949000
H	0.439547000	-3.325066000	-4.149945000
H	-1.307738000	-3.221789000	-3.906752000
C	-4.571133000	-2.635573000	0.401532000
H	-4.627599000	-1.763562000	-0.263915000
C	-4.330697000	-2.115628000	1.820631000
H	-4.243553000	-2.948529000	2.528462000
H	-5.160191000	-1.479532000	2.148975000
H	-3.408251000	-1.528079000	1.898062000
C	-5.909278000	-3.364318000	0.314782000
H	-6.081818000	-3.794904000	-0.677295000
H	-6.732571000	-2.676534000	0.530921000
H	-5.975901000	-4.180445000	1.042119000
Al	1.875362000	-0.355482000	-0.061272000
N	2.946403000	-1.594115000	0.986555000
N	3.071171000	-0.343069000	-1.563217000
C	3.949007000	-2.328948000	0.512183000
C	4.366730000	-2.252325000	-0.830963000
H	5.134603000	-2.956487000	-1.132301000
C	4.019450000	-1.280597000	-1.771372000
C	4.727690000	-3.239093000	1.414030000
H	4.178249000	-3.493360000	2.322210000
H	5.662374000	-2.754852000	1.717822000
H	5.002157000	-4.155183000	0.884985000
C	4.755515000	-1.288405000	-3.078898000
H	4.060562000	-1.463914000	-3.908234000
H	5.520782000	-2.064914000	-3.096833000

H	5.228082000	-0.321818000	-3.280049000
C	2.706812000	-1.485528000	2.402695000
C	3.571341000	-0.703845000	3.192716000
C	3.243123000	-0.511511000	4.537366000
H	3.899382000	0.096827000	5.156544000
C	2.086136000	-1.046909000	5.081311000
H	1.837459000	-0.866636000	6.123234000
C	1.237567000	-1.803896000	4.283655000
H	0.322925000	-2.206362000	4.707843000
C	1.527146000	-2.041289000	2.939246000
C	4.796756000	0.001244000	2.644872000
H	4.917430000	-0.262750000	1.585434000
C	4.613159000	1.516813000	2.724797000
H	4.525741000	1.843229000	3.766863000
H	3.706150000	1.839771000	2.203111000
H	5.475299000	2.035465000	2.289910000
C	6.071899000	-0.411167000	3.380343000
H	6.952522000	0.046802000	2.917402000
H	6.217402000	-1.496028000	3.388258000
H	6.046013000	-0.082366000	4.425122000
C	0.627045000	-2.926676000	2.103310000
H	0.588856000	-2.500931000	1.088804000
C	1.210733000	-4.336595000	2.003637000
H	2.194523000	-4.341463000	1.521083000
H	0.550278000	-4.987377000	1.420503000
H	1.326655000	-4.778722000	3.000380000
C	-0.810953000	-2.963791000	2.596183000
H	-1.435820000	-3.493034000	1.871037000
H	-1.206889000	-1.951177000	2.724673000
H	-0.907790000	-3.496337000	3.550163000
C	3.019191000	0.770973000	-2.476820000
C	2.269834000	0.702592000	-3.666279000
C	2.313846000	1.789962000	-4.543805000
H	1.731812000	1.751765000	-5.462296000
C	3.084483000	2.909784000	-4.266682000
H	3.120687000	3.737287000	-4.969866000
C	3.782411000	2.979983000	-3.068236000
H	4.356618000	3.872672000	-2.836145000
C	3.745426000	1.934625000	-2.142291000
C	1.380809000	-0.474499000	-4.011725000
H	1.597447000	-1.286389000	-3.303623000
C	-0.088327000	-0.084919000	-3.838184000
H	-0.355639000	0.747676000	-4.501566000
H	-0.285905000	0.242966000	-2.808594000
H	-0.756576000	-0.922660000	-4.067206000
C	1.628350000	-0.994975000	-5.426807000
H	1.008295000	-1.873033000	-5.633359000
H	2.674531000	-1.275273000	-5.587482000
H	1.375000000	-0.242563000	-6.180911000
C	4.483122000	2.072282000	-0.822912000
H	4.025024000	1.376254000	-0.107155000
C	4.336387000	3.470967000	-0.231620000
H	4.815549000	4.235779000	-0.851789000
H	4.808073000	3.519084000	0.753185000
H	3.282197000	3.739767000	-0.109509000
C	5.953103000	1.674319000	-0.957500000
H	6.468354000	2.307578000	-1.689325000
H	6.065795000	0.632573000	-1.277664000
H	6.473391000	1.781251000	0.001253000
H	1.814681000	1.068253000	0.993635000
C	-0.835888000	1.870843000	2.448962000
C	-0.484601000	3.213461000	2.548973000
C	-1.703334000	1.470682000	3.455389000
C	-0.929694000	4.097517000	3.526038000
C	-2.192239000	2.280040000	4.477058000
C	-1.796943000	3.615427000	4.502957000
H	-0.600383000	5.132412000	3.512679000
H	-2.864381000	1.867075000	5.223884000
H	-2.160984000	4.276300000	5.284674000
F	-2.146074000	0.169916000	3.462406000
F	0.370150000	3.730533000	1.614913000

Int-3'_AlPdPCy$_3$ _M06L.log

SCF (M06L) = -2847.09923891
E(SCF)+ZPE(0 K) = -2845.892288
H(298 K) = -2845.826356
G(298 K) = -2845.991348
Lowest Frequency = 17.1200 cm^{-1}

Pd	-0.703730	0.023118	-0.817660
P	-1.999487	-0.791578	0.917161
C	-1.481615	-2.505386	1.409429
H	-2.205963	-2.866977	2.157637
C	-0.088516	-2.587872	2.035716
H	0.646983	-2.175635	1.324915
H	-0.029948	-1.962635	2.938143
C	0.274538	-4.033094	2.368721
H	1.286749	-4.079287	2.790726
H	-0.404602	-4.408807	3.149142
C	0.180099	-4.928218	1.139319
H	0.439812	-5.962419	1.396046
H	0.924236	-4.594684	0.402477
C	-1.201509	-4.851557	0.506760
H	-1.952214	-5.261693	1.200047
H	-1.247169	-5.468574	-0.398268
C	-1.557295	-3.409952	0.171612
H	-2.547585	-3.358505	-0.295671
H	-0.855378	-3.015871	-0.579614
C	-3.785461	-1.062095	0.432660
H	-3.662033	-1.407880	-0.606425
C	-4.615961	-2.126401	1.161213
H	-4.830873	-1.803753	2.189357
H	-4.075727	-3.076135	1.242910
C	-5.929431	-2.357435	0.414186
H	-5.697363	-2.772267	-0.578185
H	-6.529490	-3.114671	0.932560
C	-6.723420	-1.067376	0.243120
H	-7.048129	-0.713128	1.233351
H	-7.638390	-1.255967	-0.330187
C	-5.881882	0.016119	-0.420387
H	-6.445941	0.952956	-0.498498
H	-5.631134	-0.279332	-1.450001
C	-4.584281	0.242600	0.346470
H	-3.991968	1.031377	-0.123627
H	-4.823454	0.591312	1.363478
C	-1.960904	0.203809	2.485917
H	-0.942320	0.025561	2.870599
C	-2.064380	1.706871	2.187301
H	-1.269467	1.992673	1.482890
H	-3.009318	1.920917	1.672094
C	-1.992636	2.546644	3.458146
H	-2.137437	3.605016	3.210471
H	-0.983181	2.468945	3.886748
C	-3.001222	2.089699	4.504818
H	-2.917325	2.698517	5.412391
H	-4.021541	2.243074	4.122051
C	-2.808506	0.613191	4.827538
H	-3.524382	0.282255	5.588760
H	-1.806131	0.463489	5.257205
C	-2.949836	-0.235595	3.568351
H	-3.975502	-0.127736	3.189991
H	-2.817489	-1.300646	3.800429
Al	1.622879	0.382120	-0.439572
N	3.284416	-0.564120	-0.703599
N	2.484053	2.118522	-0.512714
C	4.415925	-0.018958	-1.189796

C	4.595637	1.358067	-1.351190
H	5.556411	1.675868	-1.739043
C	3.719661	2.375039	-0.941876
C	5.572806	-0.910620	-1.533004
H	5.873083	-1.515956	-0.671247
H	6.431018	-0.329009	-1.870874
H	5.298995	-1.620351	-2.321037
C	4.211140	3.788874	-0.968891
H	3.638595	4.384380	-1.687190
H	5.266349	3.842277	-1.238118
H	4.066676	4.266636	0.005368
C	3.323260	-1.960985	-0.355362
C	3.565836	-2.304845	0.992050
C	3.718735	-3.657927	1.305056
H	3.918162	-3.950064	2.332244
C	3.612883	-4.639203	0.326421
H	3.739578	-5.685549	0.591119
C	3.296842	-4.283647	-0.977709
H	3.162033	-5.057918	-1.729170
C	3.134396	-2.944658	-1.342857
C	3.649399	-1.239105	2.071958
H	2.886777	-0.478666	1.828060
C	3.315679	-1.769899	3.460887
H	2.357780	-2.298240	3.478504
H	3.257992	-0.944750	4.176875
H	4.086460	-2.454983	3.830828
C	5.004802	-0.530071	2.103824
H	5.812232	-1.248625	2.287249
H	5.029706	0.208671	2.913199
H	5.227041	-0.000529	1.173531
C	2.689764	-2.585302	-2.745881
H	2.922795	-1.526308	-2.917073
C	1.170814	-2.742192	-2.849886
H	0.806301	-2.455600	-3.840982
H	0.644195	-2.115243	-2.119716
H	0.875462	-3.783515	-2.667195
C	3.395901	-3.396295	-3.828271
H	4.486693	-3.344952	-3.739845
H	3.120580	-3.027257	-4.820397
H	3.113415	-4.453868	-3.793128
C	1.645616	3.168319	-0.002414
C	0.837664	3.922733	-0.875609
C	-0.103050	4.786911	-0.307995
H	-0.754977	5.356636	-0.966972
C	-0.220599	4.929449	1.068295
H	-0.964561	5.603153	1.484980
C	0.625856	4.218620	1.910703
H	0.548708	4.345587	2.989506
C	1.565354	3.323459	1.396506
C	0.986517	3.874838	-2.384892
H	1.806876	3.185659	-2.624170
C	-0.265277	3.360563	-3.090420
H	-1.129763	3.998701	-2.879959
H	-0.520923	2.347478	-2.773069
H	-0.110988	3.354156	-4.174583
C	1.351318	5.259281	-2.928997
H	0.518437	5.959348	-2.800167
H	1.569569	5.208057	-4.000287
H	2.219205	5.699194	-2.425924
C	2.483715	2.572208	2.343541
H	3.156340	1.944300	1.744002
C	1.707300	1.647264	3.277616
H	1.054807	2.218791	3.947253
H	2.390811	1.064868	3.905577
H	1.076611	0.945856	2.714983
C	3.354871	3.535595	3.148944
H	3.945253	4.190977	2.501103
H	4.048420	2.987778	3.795605
H	2.743363	4.177377	3.792822
H	0.477154	0.476941	-1.962037
C	-2.281821	0.221412	-2.214311
C	-2.662482	-0.768435	-3.109434
C	-3.007052	1.395833	-2.361059
C	-3.656725	-0.641490	-4.074252
C	-4.009799	1.615441	-3.298435
C	-4.329387	0.575037	-4.168445
H	-3.886175	-1.474095	-4.732125
H	-4.523774	2.571265	-3.333615
H	-5.107462	0.709573	-4.914144
F	-2.031423	-1.980939	-3.032304
F	-2.744587	2.425245	-1.489398

Int-3_1,2,3-TriFB_AlPdAl _M06L.log

SCF (M06L) = -3140.48294905
E(SCF)+ZPE(0 K) = -3139.130038
H(298 K) = -3139.048355
G(298 K) = -3139.242545
Lowest Frequency = 20.1374 cm^{-1}

Pd	-0.205750	0.979658	-0.447871
Al	-1.407161	-0.880762	0.234477
N	-2.445232	-1.823482	-1.118602
N	-1.909540	-2.122583	1.641243
C	-2.906363	-3.075527	-1.011412
C	-2.878607	-3.792650	0.193260
H	-3.304303	-4.789131	0.167608
C	-2.526931	-3.304371	1.453550
C	-3.543502	-3.744006	-2.192752
H	-4.376373	-3.146986	-2.578395
H	-3.909742	-4.738019	-1.933610
H	-2.830292	-3.835334	-3.018401
C	-2.945196	-4.129420	2.637169
H	-2.204714	-4.131603	3.439871
H	-3.158092	-5.156543	2.336693
H	-3.863245	-3.713296	3.069818
C	-2.780235	-1.107691	-2.329673
C	-3.897681	-0.248751	-2.299145
C	-4.313899	0.330570	-3.498625
H	-5.170073	0.998700	-3.497038
C	-3.630022	0.101641	-4.686211
H	-3.972494	0.562675	-5.608747
C	-2.479832	-0.672964	-4.676307
H	-1.906187	-0.803901	-5.591691
C	-2.028728	-1.283550	-3.502567
C	-4.604047	0.075354	-0.996875
H	-3.822034	0.121035	-0.220450
C	-5.267263	1.446316	-1.020147
H	-4.581879	2.211673	-1.395286
H	-5.579242	1.735914	-0.011845
H	-6.166452	1.452860	-1.646786
C	-5.609189	-1.000118	-0.582762
H	-6.385671	-1.118293	-1.347927
H	-6.109317	-0.719183	0.351941
H	-5.141727	-1.977535	-0.424469
C	-0.706145	-2.016580	-3.509690
H	-0.597922	-2.537857	-2.551113
C	0.424076	-0.992464	-3.610512
H	1.399069	-1.485855	-3.572910
H	0.370463	-0.257382	-2.795159
H	0.360118	-0.441140	-4.557163

C	-0.584412	-3.055654	-4.620966
H	-1.386638	-3.801370	-4.589965
H	0.371479	-3.584788	-4.539342
H	-0.611347	-2.590072	-5.612334
C	-1.719853	-1.709170	3.007966
C	-0.478876	-1.936871	3.632173
C	-0.362114	-1.628359	4.990973
H	0.580814	-1.816769	5.498986
C	-1.430725	-1.102723	5.705915
H	-1.320003	-0.877788	6.763046
C	-2.634965	-0.848072	5.061123
H	-3.459505	-0.409306	5.617191
C	-2.802530	-1.136020	3.705070
C	0.676483	-2.584789	2.893501
H	0.517898	-2.416813	1.819019
C	2.031208	-1.980162	3.241222
H	2.295312	-2.117019	4.295893
H	2.065150	-0.906416	3.029605
H	2.822274	-2.459845	2.653425
C	0.702232	-4.095253	3.130593
H	0.792149	-4.321921	4.199476
H	1.560196	-4.549764	2.622119
H	-0.201260	-4.589597	2.760350
C	-4.096866	-0.776527	3.001356
H	-4.182501	-1.395602	2.097864
C	-4.049266	0.686490	2.551799
H	-3.949690	1.354299	3.415338
H	-4.965690	0.960222	2.017297
H	-3.198995	0.892687	1.888702
C	-5.337774	-1.036663	3.849646
H	-5.362715	-2.058964	4.241558
H	-6.243296	-0.881186	3.255499
H	-5.398715	-0.354325	4.704004
Al	1.944016	0.138713	0.135710
N	2.984934	1.165651	1.423701
N	3.355115	-1.131061	-0.203907
C	4.129265	0.777229	1.978598
C	4.734503	-0.455931	1.668359
H	5.616249	-0.714160	2.244458
C	4.422545	-1.302906	0.605369
C	4.871854	1.673769	2.923979
H	4.232384	2.442682	3.360572
H	5.684951	2.181022	2.392873
H	5.332614	1.086130	3.722038
C	5.345476	-2.459287	0.353857
H	4.812404	-3.408680	0.477319
H	6.190542	-2.445335	1.042775
H	5.727026	-2.455859	-0.671961
C	2.521811	2.520908	1.574450
C	3.134849	3.547086	0.830832
C	2.592867	4.832148	0.914019
H	3.050195	5.634285	0.337973
C	1.475518	5.094346	1.692005
H	1.058791	6.096712	1.729724
C	0.882115	4.067345	2.414809
H	-0.003325	4.274650	3.006930
C	1.385426	2.766490	2.371101
C	4.317527	3.319205	-0.090805
H	4.627217	2.267402	-0.019636
C	3.928057	3.587573	-1.544344
H	3.625261	4.631655	-1.679885
H	3.089301	2.957558	-1.857864
H	4.778115	3.403766	-2.211674
C	5.514401	4.188733	0.296136
H	6.386536	3.946548	-0.320680
H	5.796592	4.067562	1.346651
H	5.293104	5.250774	0.143240
C	0.763179	1.665244	3.205625
H	0.785190	0.741326	2.605988
C	1.577814	1.423512	4.477389
H	2.601215	1.099520	4.260585
H	1.107684	0.648269	5.092556
H	1.637887	2.339508	5.077241
C	-0.697690	1.913759	3.545212
H	-1.117789	1.029523	4.031141
H	-1.279631	2.127992	2.645391
H	-0.821122	2.751357	4.242350
C	3.327958	-1.886116	-1.430169
C	2.774567	-3.179709	-1.477523
C	2.818786	-3.876405	-2.689377
H	2.384096	-4.872858	-2.741602
C	3.419702	-3.330629	-3.814312
H	3.463094	-3.897378	-4.740424
C	3.943713	-2.045208	-3.756306
H	4.385307	-1.608848	-4.647692
C	3.882764	-1.288257	-2.584099
C	2.134246	-3.849632	-0.277386
H	2.328253	-3.224177	0.605031
C	0.619065	-3.946514	-0.452035
H	0.356432	-4.520632	-1.350257
H	0.174448	-2.949249	-0.561792
H	0.147537	-4.436889	0.406678
C	2.716955	-5.239345	-0.018391
H	2.295301	-5.673667	0.893894
H	3.806199	-5.221383	0.088355
H	2.484064	-5.928114	-0.837343
C	4.397623	0.140378	-2.576475
H	3.845584	0.687916	-1.799876
C	4.126189	0.856022	-3.895920
H	4.719566	0.445200	-4.719799
H	4.386961	1.914208	-3.813404
H	3.069176	0.792822	-4.173069
C	5.880781	0.214796	-2.211698
H	6.490332	-0.356291	-2.922031
H	6.078740	-0.175120	-1.207834
H	6.229883	1.253623	-2.232827
H	1.579496	1.357177	-1.078906
C	-1.541164	2.389461	-1.259335
C	-1.822934	2.473948	-2.634848
C	-2.142552	3.370485	-0.482127
C	-2.631523	3.466754	-3.193442
C	-2.939886	4.389506	-0.993367
C	-3.181371	4.430025	-2.361834
H	-2.840162	3.508369	-4.259055
F	-1.977986	3.383682	0.872293
H	-1.406741	1.723562	-3.307288
F	-3.483627	5.321479	-0.187721
F	-3.958806	5.416944	-2.852645

Int-3_FB_AlPdAl _M06L.log

SCF (M06L) = -2942.04152775
E(SCF)+ZPE(0 K) = -2940.672991
H(298 K) = -2940.593013
G(298 K) = -2940.783480
Lowest Frequency = 17.4183 cm^{-1}

Pd	-0.132409	-0.220586	1.266748
Al	-1.581321	0.115604	-0.501377
N	-2.667476	1.734418	-0.489687
N	-2.389437	-0.545581	-2.137887

C	-3.332231	2.226262	-1.541280
C	-3.502463	1.509291	-2.735500
H	-4.079252	1.999937	-3.511077
C	-3.170085	0.173374	-2.966532
C	-3.993465	3.569304	-1.451338
H	-4.667718	3.616061	-0.590169
H	-4.557494	3.791917	-2.357899
H	-3.250885	4.358981	-1.297029
C	-3.796915	-0.482732	-4.163444
H	-3.127515	-1.185732	-4.663962
H	-4.138887	0.265854	-4.879955
H	-4.672141	-1.064762	-3.849619
C	-2.785228	2.457685	0.758301
C	-3.711201	1.988097	1.711910
C	-3.905710	2.749455	2.866119
H	-4.605979	2.401367	3.619586
C	-3.203049	3.928250	3.080191
H	-3.373371	4.505804	3.984801
C	-2.251990	4.338088	2.158810
H	-1.658075	5.228490	2.353675
C	-2.013980	3.607293	0.993528
C	-4.465634	0.688493	1.505651
H	-3.755186	-0.017247	1.043850
C	-4.918039	0.055375	2.814732
H	-4.102342	0.005277	3.540813
H	-5.273190	-0.965224	2.639455
H	-5.750399	0.610504	3.263592
C	-5.655547	0.844568	0.556523
H	-6.370930	1.575482	0.952286
H	-6.187518	-0.108687	0.448446
H	-5.361901	1.171407	-0.446174
C	-0.864381	3.997604	0.092728
H	-0.952865	3.433555	-0.843863
C	0.444056	3.581342	0.764558
H	1.296726	3.798248	0.115993
H	0.444609	2.512439	1.015357
H	0.581184	4.129367	1.704731
C	-0.832097	5.480881	-0.264246
H	-1.765293	5.823517	-0.725555
H	-0.014541	5.681864	-0.965576
H	-0.657685	6.104792	0.619134
C	-2.252293	-1.947623	-2.432467
C	-1.107426	-2.403062	-3.113780
C	-1.045083	-3.755688	-3.461862
H	-0.178000	-4.124403	-4.004888
C	-2.073796	-4.631931	-3.139818
H	-2.007169	-5.677999	-3.425813
C	-3.179523	-4.169805	-2.436869
H	-3.969898	-4.863850	-2.161785
C	-3.288959	-2.828582	-2.063462
C	-0.008186	-1.451330	-3.544260
H	-0.059092	-0.569834	-2.889669
C	1.390477	-2.036429	-3.394603
H	1.552682	-2.907368	-4.039689
H	1.596588	-2.344100	-2.364223
H	2.143995	-1.291649	-3.674775
C	-0.224128	-0.976544	-4.981722
H	-0.249501	-1.825790	-5.674658
H	0.593219	-0.317113	-5.295251
H	-1.160159	-0.422063	-5.100177
C	-4.464355	-2.367957	-1.222986
H	-4.553797	-1.277527	-1.318277
C	-4.194296	-2.675356	0.252356
H	-4.073284	-3.754032	0.407965
H	-5.023816	-2.332949	0.880997
H	-3.278517	-2.189797	0.613779
C	-5.792963	-2.974476	-1.664363
H	-5.980432	-2.822793	-2.732650
H	-6.620511	-2.522216	-1.109520
H	-5.833433	-4.052099	-1.473212
Al	1.825607	-0.175687	-0.108654
N	2.909068	-1.800849	0.033995
N	3.056701	0.695225	-1.315903
C	3.933350	-2.120794	-0.750079
C	4.368207	-1.284477	-1.796259
H	5.151532	-1.681962	-2.432318
C	4.016328	0.047801	-2.011006
C	4.725316	-3.373298	-0.516825
H	4.185508	-4.103690	0.088059
H	5.657279	-3.132297	0.006391
H	5.006510	-3.826937	-1.470788
C	4.769943	0.793310	-3.073804
H	4.091841	1.109738	-3.874395
H	5.552681	0.171913	-3.509979
H	5.224239	1.708103	-2.680717
C	2.650256	-2.546197	1.237523
C	3.465751	-2.342285	2.366636
C	3.127669	-3.003186	3.550437
H	3.744444	-2.849096	4.433676
C	2.013280	-3.824754	3.624904
H	1.757777	-4.315807	4.559504
C	1.215058	-4.005577	2.502662
H	0.335739	-4.639093	2.570353
C	1.510766	-3.373338	1.294070
C	4.659769	-1.407423	2.374768
H	4.787875	-0.987103	1.367908
C	4.426924	-0.241196	3.334994
H	4.311266	-0.600200	4.363588
H	3.521708	0.317278	3.075925
H	5.282058	0.444552	3.322006
C	5.949878	-2.139075	2.747241
H	6.814086	-1.471659	2.660974
H	6.131573	-3.014066	2.115340
H	5.914444	-2.492529	3.783757
C	0.666905	-3.624342	0.061509
H	0.634529	-2.686899	-0.514285
C	1.302838	-4.698876	-0.821074
H	2.293792	-4.401266	-1.180793
H	0.676678	-4.897035	-1.698033
H	1.417651	-5.638798	-0.268059
C	-0.778454	-3.967259	0.381395
H	-1.360337	-4.023214	-0.542305
H	-1.222445	-3.194083	1.018298
H	-0.879175	-4.935952	0.885137
C	3.010816	2.129666	-1.434896
C	2.273100	2.750969	-2.460152
C	2.296131	4.146823	-2.542194
H	1.719357	4.639624	-3.322602
C	3.049615	4.908781	-1.661206
H	3.070062	5.991244	-1.753921
C	3.757783	4.281245	-0.643788
H	4.320824	4.883277	0.063702
C	3.731447	2.893358	-0.489422
C	1.450443	1.975782	-3.470721
H	1.677534	0.907713	-3.346529
C	-0.043858	2.165372	-3.212678
H	-0.329239	3.222912	-3.283830
H	-0.312640	1.823650	-2.204876
H	-0.649877	1.607223	-3.935004
C	1.783924	2.373076	-4.908952
H	1.229624	1.753715	-5.621571
H	2.850820	2.272338	-5.132352

H	1.508921	3.414096	-5.108856
C	4.455397	2.242919	0.676389
H	3.927091	1.308765	0.913211
C	4.411738	3.103327	1.934832
H	5.005571	4.018095	1.832504
H	4.822468	2.551486	2.784298
H	3.385754	3.388840	2.186827
C	5.895418	1.869618	0.322036
H	6.469212	2.754328	0.021274
H	5.944105	1.142006	-0.494819
H	6.400334	1.423402	1.186636
H	1.767025	0.324970	1.546238
C	-1.189677	-0.434322	3.062000
C	-1.281088	0.655729	3.924466
C	-1.772246	-1.613728	3.558073
C	-1.888815	0.638794	5.173174
C	-2.374256	-1.691388	4.817540
C	-2.440008	-0.559625	5.627116
H	-2.803399	-2.631058	5.160031
F	-0.722514	1.840896	3.532734
H	-1.918104	1.545742	5.772328
H	-2.916961	-0.601939	6.603108
H	-1.749158	-2.516095	2.944192

Int-4_AlPdAl _M06L.log

SCF (M06L) = -3041.26833238

E(SCF)+ZPE(0 K) = -3039.828642

H(298 K) = -3039.827698

G(298 K) = -3040.024149

Lowest Frequency = 16.5919 cm^{-1}

Pd	0.108678000	-0.102763000	-0.237573000
Al	2.361827000	0.339170000	0.225014000
N	3.269308000	1.957492000	-0.278456000
N	3.750523000	-0.239576000	1.416305000
C	4.374026000	2.454861000	0.287262000
C	5.072042000	1.775471000	1.297075000
H	5.952069000	2.271667000	1.689744000
C	4.820745000	0.487169000	1.780845000
C	4.919860000	3.769175000	-0.182795000
H	5.030786000	3.776113000	-1.271888000
H	5.884835000	3.985759000	0.277055000
H	4.229474000	4.585344000	0.056309000
C	5.827037000	-0.113463000	2.716003000
H	5.346257000	-0.525107000	3.608633000
H	6.574605000	0.620797000	3.017485000
H	6.340523000	-0.955424000	2.238708000
C	2.672884000	2.646716000	-1.395190000
C	2.858876000	2.108331000	-2.685292000
C	2.296248000	2.789748000	-3.765910000
H	2.418947000	2.390502000	-4.768432000
C	1.556521000	3.950435000	-3.578014000
H	1.125016000	4.463359000	-4.433373000
C	1.331125000	4.425516000	-2.294541000
H	0.700120000	5.299074000	-2.146183000
C	1.868745000	3.779017000	-1.179615000
C	3.623483000	0.814820000	-2.894544000
H	3.355399000	0.147088000	-2.055842000
C	3.209990000	0.079578000	-4.162253000
H	2.122173000	-0.019445000	-4.229265000
H	3.640327000	-0.926768000	-4.172806000
H	3.566809000	0.590747000	-5.064019000
C	5.137286000	1.020054000	-2.846317000
H	5.461052000	1.708899000	-3.635409000
H	5.662032000	0.069821000	-3.000503000
H	5.471716000	1.428271000	-1.886792000
C	1.470046000	4.225760000	0.211657000
H	2.146580000	3.754217000	0.936495000
C	0.053655000	3.727918000	0.509943000
H	-0.256338000	3.999404000	1.525055000
H	-0.029129000	2.637450000	0.403236000
H	-0.666553000	4.172735000	-0.187418000
C	1.563765000	5.736507000	0.407629000
H	2.557321000	6.127191000	0.160727000
H	1.345335000	6.003167000	1.446358000
H	0.839611000	6.269489000	-0.217756000
C	3.700173000	-1.617964000	1.832707000
C	3.012221000	-1.984221000	3.003443000
C	3.065310000	-3.323008000	3.404501000
H	2.552577000	-3.618096000	4.318305000
C	3.730895000	-4.279125000	2.650590000
H	3.754959000	-5.314519000	2.978973000
C	4.339520000	-3.910617000	1.457328000
H	4.825060000	-4.666301000	0.843839000
C	4.341294000	-2.582550000	1.027404000
C	2.187794000	-1.007166000	3.819803000
H	2.218742000	-0.029858000	3.320826000
C	0.725425000	-1.456802000	3.860063000
H	0.626960000	-2.456178000	4.301386000
H	0.297083000	-1.483270000	2.852099000
H	0.123041000	-0.769216000	4.462851000
C	2.741109000	-0.835559000	5.233464000
H	2.718993000	-1.781898000	5.786001000
H	2.144853000	-0.111648000	5.798672000
H	3.777584000	-0.482219000	5.230765000
C	4.962909000	-2.227704000	-0.310882000
H	5.069055000	-1.135860000	-0.365712000
C	4.029420000	-2.659756000	-1.445000000
H	3.889581000	-3.747063000	-1.438760000
H	4.444395000	-2.380609000	-2.420291000
H	3.033812000	-2.207670000	-1.357838000
C	6.354433000	-2.826126000	-0.497999000
H	7.033116000	-2.551972000	0.316162000
H	6.796819000	-2.477148000	-1.436355000
H	6.322667000	-3.919900000	-0.545880000
Al	-2.185895000	-0.286760000	0.534318000
N	-3.296647000	-1.890620000	0.475239000
N	-3.536919000	0.697618000	1.550415000
C	-4.402971000	-2.128956000	1.188956000
C	-4.983869000	-1.168743000	2.028325000
H	-5.866833000	-1.479287000	2.575095000
C	-4.622036000	0.177016000	2.147502000
C	-5.097410000	-3.452105000	1.061982000
H	-5.570329000	-3.543646000	0.077740000
H	-5.869052000	-3.568805000	1.824185000
H	-4.387978000	-4.281871000	1.132405000
C	-5.524823000	1.064414000	2.953125000
H	-5.005658000	1.465672000	3.829342000
H	-6.408209000	0.522724000	3.293115000
H	-5.844125000	1.932170000	2.366386000
C	-2.894299000	-2.879440000	-0.493489000
C	-3.535988000	-2.892218000	-1.749426000
C	-3.152506000	-3.859328000	-2.678516000
H	-3.624722000	-3.870746000	-3.658098000
C	-2.168398000	-4.793420000	-2.378509000
H	-1.885936000	-5.543071000	-3.112629000
C	-1.529143000	-4.745017000	-1.149817000
H	-0.733713000	-5.453238000	-0.927029000
C	-1.860279000	-3.782056000	-0.192558000
C	-4.538986000	-1.824166000	-2.141782000
H	-4.927847000	-1.356849000	-1.227049000
C	-3.817197000	-0.740353000	-2.944678000
H	-3.412318000	-1.148956000	-3.877814000
H	-2.960443000	-0.332569000	-2.394948000
H	-4.491721000	0.086026000	-3.199600000
C	-5.736020000	-2.367039000	-2.915676000
H	-6.469125000	-1.572454000	-3.089151000
H	-6.239781000	-3.178596000	-2.380098000
H	-5.445008000	-2.752278000	-3.898603000
C	-1.087653000	-3.750412000	1.110706000
H	-1.437378000	-2.888823000	1.696619000
C	-1.339927000	-5.005925000	1.944351000
H	-2.402102000	-5.141968000	2.175539000
H	-0.793060000	-4.956143000	2.892585000
H	-1.000853000	-5.904366000	1.415754000

C	0.406130000	-3.556295000	0.856638000
H	0.962848000	-3.570496000	1.798522000
H	0.599679000	-2.598293000	0.354353000
H	0.815634000	-4.348898000	0.219154000
C	-3.355807000	2.123837000	1.614769000
C	-2.833037000	2.732015000	2.770668000
C	-2.765104000	4.127530000	2.810011000
H	-2.367304000	4.611532000	3.699842000
C	-3.175768000	4.899673000	1.733115000
H	-3.122185000	5.983723000	1.785788000
C	-3.615952000	4.279320000	0.570039000
H	-3.888274000	4.884709000	-0.290021000
C	-3.700255000	2.888664000	0.478678000
C	-2.267189000	1.932337000	3.928489000
H	-2.561211000	0.882010000	3.801211000
C	-0.737820000	1.996937000	3.886152000
H	-0.398015000	3.037424000	3.957157000
H	-0.341993000	1.575636000	2.954432000
H	-0.297559000	1.447694000	4.726343000
C	-2.778360000	2.406419000	5.287423000
H	-2.414884000	1.750938000	6.084778000
H	-3.871966000	2.424450000	5.340993000
H	-2.426346000	3.417877000	5.517536000
C	-4.144640000	2.221300000	-0.809984000
H	-3.534482000	1.309450000	-0.924976000
C	-3.876221000	3.066615000	-2.048663000
H	-4.562973000	3.918384000	-2.116831000
H	-4.018366000	2.462174000	-2.950296000
H	-2.850547000	3.443010000	-2.065857000
C	-5.610214000	1.786889000	-0.762150000
H	-6.264496000	2.652268000	-0.602853000
H	-5.806396000	1.064127000	0.035902000
H	-5.904312000	1.318098000	-1.708345000
H	0.724928000	0.284655000	1.309127000
C	-0.141206000	-0.385211000	-2.278394000
C	-0.569262000	0.658254000	-3.094383000
C	0.069684000	-1.566603000	-2.981757000
C	-0.775459000	0.582754000	-4.467033000
C	-0.133028000	-1.738217000	-4.347854000
C	-0.557101000	-0.642104000	-5.097494000
H	-1.107925000	1.460973000	-5.013717000
H	0.048443000	-2.707747000	-4.802594000
H	-0.714026000	-0.740953000	-6.167807000
F	0.515712000	-2.655867000	-2.288570000
F	-0.841141000	1.859701000	-2.497990000

Int-4'_AlPdPCy$_3$ _M06L.log

SCF (M06L) = -2847.10394193
E(SCF)+ZPE(0 K) = -2845.897563
H(298 K) = -2845.831658
G(298 K) = -2845.996280
Lowest Frequency = 11.6147 cm^{-1}

Pd	-0.453382	-0.271395	0.036609
P	-2.801087	-0.386141	0.421974
C	-3.320388	-1.370894	1.909204
H	-4.422009	-1.374791	1.965091
C	-2.779213	-0.787648	3.217020
H	-1.681578	-0.723511	3.144083
H	-3.136951	0.239498	3.363826
C	-3.177776	-1.647329	4.413390
H	-2.769225	-1.219528	5.336568
H	-4.272484	-1.623772	4.522881
C	-2.723349	-3.092393	4.247004
H	-3.054884	-3.699797	5.097208
H	-1.624652	-3.126461	4.252200
C	-3.228545	-3.682009	2.936184
H	-4.325903	-3.762318	2.972124
H	-2.849904	-4.702103	2.801901
C	-2.831134	-2.816109	1.746503
H	-3.208560	-3.257009	0.815758
H	-1.735348	-2.796908	1.642737
C	-3.736100	-1.219365	-0.961043
H	-3.063934	-2.065774	-1.187256
C	-5.127451	-1.803059	-0.692535
H	-5.846706	-0.990787	-0.517905
H	-5.137802	-2.421713	0.212492
C	-5.582941	-2.637759	-1.888847
H	-4.906822	-3.499586	-1.992272
H	-6.581774	-3.050801	-1.704358
C	-5.567595	-1.831429	-3.183264
H	-6.326555	-1.037052	-3.117791
H	-5.858495	-2.463834	-4.030044
C	-4.204305	-1.193725	-3.426776
H	-4.221154	-0.578175	-4.333830
H	-3.450111	-1.975529	-3.600225
C	-3.771800	-0.358573	-2.227759
H	-2.797795	0.104641	-2.407100
H	-4.490117	0.464012	-2.085053
C	-3.533619	1.300514	0.711748
H	-3.178905	1.545680	1.727475
C	-2.913638	2.352273	-0.220587
H	-1.816224	2.305335	-0.151650
H	-3.155677	2.116526	-1.265649
C	-3.419349	3.752474	0.109092
H	-2.995838	4.477736	-0.595694
H	-3.049907	4.042677	1.104987
C	-4.941920	3.820979	0.109854
H	-5.283380	4.831239	0.363726
H	-5.314396	3.614440	-0.904825
C	-5.531797	2.797667	1.072820
H	-6.627081	2.844438	1.068156
H	-5.215409	3.040939	2.098449
C	-5.061307	1.388844	0.724522
H	-5.448256	1.124721	-0.270037
H	-5.484068	0.655549	1.424108
Al	1.734155	0.361751	0.487457
N	3.342839	-0.539883	0.963932
N	2.478785	2.112729	0.663028
C	4.428792	0.033945	1.501856
C	4.534886	1.416227	1.698281
H	5.445233	1.767496	2.170162
C	3.646373	2.405046	1.247950
C	5.589866	-0.832145	1.881388
H	5.996703	-1.340995	1.001177
H	5.273072	-1.626007	2.566194
H	6.385742	-0.253135	2.350533
C	4.048714	3.838646	1.407472
H	3.402956	4.340600	2.135114
H	3.930916	4.383722	0.465873
H	5.081472	3.927551	1.745921
C	3.392329	-1.923363	0.559931
C	2.941866	-2.928232	1.433501
C	2.944175	-4.247880	0.977429
H	2.589944	-5.035830	1.638508
C	3.368015	-4.564860	-0.306204
H	3.353756	-5.596741	-0.645618
C	3.795835	-3.556592	-1.159247
H	4.110663	-3.801660	-2.171830
C	3.817829	-2.221643	-0.749101
C	2.399791	-2.605591	2.809787
H	2.647687	-1.560360	3.035418
C	0.875012	-2.728018	2.813800
H	0.416851	-2.108799	2.030882
H	0.462159	-2.414676	3.779781
H	0.565975	-3.765505	2.633344
C	3.018473	-3.474003	3.902215
H	2.758029	-4.529830	3.771963
H	2.653057	-3.171712	4.888490
H	4.111365	-3.405900	3.911814
C	4.272769	-1.153235	-1.725799
H	4.251020	-0.181586	-1.213377
C	3.323277	-1.066577	-2.920173
H	3.634883	-0.269107	-3.604846
H	2.291232	-0.869420	-2.608460
H	3.311745	-2.003791	-3.487685
C	5.709019	-1.389014	-2.190911
H	6.411509	-1.436594	-1.352209
H	6.037274	-0.585074	-2.857858
H	5.797007	-2.329880	-2.745324

C	1.650064	3.153097	0.112017
C	1.639819	3.317514	-1.292979
C	0.791130	4.287666	-1.828807
H	0.761198	4.433138	-2.903822
C	-0.031777	5.057801	-1.015015
H	-0.686803	5.804271	-1.456395
C	-0.033381	4.853999	0.356321
H	-0.705732	5.432174	0.987879
C	0.796059	3.896990	0.948107
C	2.507961	2.456029	-2.197761
H	2.387549	1.411182	-1.857537
C	2.083234	2.488392	-3.660975
H	2.295056	3.462886	-4.116582
H	1.022221	2.267786	-3.784419
H	2.648195	1.741311	-4.226787
C	3.996204	2.801377	-2.099077
H	4.167158	3.850597	-2.367290
H	4.572740	2.185902	-2.798964
H	4.413438	2.634288	-1.103008
C	0.694294	3.667733	2.444301
H	1.453858	2.930265	2.733652
C	-0.675234	3.089323	2.808253
H	-1.468078	3.817498	2.597566
H	-0.725157	2.846424	3.875379
H	-0.888639	2.178189	2.239601
C	0.940180	4.949087	3.241836
H	1.894655	5.425567	2.995676
H	0.934246	4.743111	4.316682
H	0.154597	5.688037	3.049506
C	-0.329795	-1.056106	-1.875968
C	-0.050870	-2.403524	-2.079931
C	-0.407843	-0.332674	-3.058232
C	0.143056	-3.005186	-3.317941
C	-0.227954	-0.848219	-4.336415
C	0.055062	-2.207953	-4.458520
H	0.365019	-4.066710	-3.376505
H	-0.310400	-0.199502	-5.203580
H	0.203917	-2.645809	-5.441196
H	0.130141	0.355623	1.554569
F	-0.696184	1.008800	-2.964077
F	0.051622	-3.202853	-0.974733

Int-5_AlPdAl _M06L.log

SCF (M06L) = -3041.27671329
E(SCF)+ZPE(0 K) = -3039.918493
H(298 K) = -3039.836665
G(298 K) = -3040.034017
Lowest Frequency = 17.8397 cm^{-1}

Pd	0.046856000	0.100819000	0.737444000
Al	-2.258497000	0.782042000	0.336747000
N	-4.072955000	0.019873000	0.188823000
N	-2.917815000	2.521781000	-0.315983000
C	-5.225359000	0.688316000	0.063908000
C	-5.273995000	2.077294000	-0.115947000
H	-6.260919000	2.523822000	-0.163563000
C	-4.191530000	2.929436000	-0.374173000
C	-6.526042000	-0.059886000	0.098406000
H	-7.369619000	0.598244000	-0.113816000
H	-6.684727000	-0.520803000	1.079685000
H	-6.525871000	-0.882085000	-0.624510000
C	-4.503259000	4.339393000	-0.783954000
H	-5.535360000	4.600149000	-0.544101000
H	-4.365273000	4.467169000	-1.863583000
H	-3.827023000	5.052957000	-0.304442000
C	-4.089926000	-1.414798000	0.203288000
C	-3.702084000	-2.093982000	-0.970722000
C	-3.719156000	-3.491069000	-0.961602000
H	-3.429772000	-4.031296000	-1.859881000
C	-4.084630000	-4.198903000	0.176280000
C	-4.407271000	-3.513463000	1.340102000
H	-4.642973000	-4.069624000	2.245032000
C	-4.404789000	-2.117609000	1.382285000
C	-1.897651000	3.381406000	-0.847265000
C	-0.995836000	4.031156000	0.017223000
C	-0.024960000	4.865101000	-0.546483000
H	0.669638000	5.380378000	0.113532000
C	0.073231000	5.040704000	-1.918830000
C	-0.786807000	4.346255000	-2.760912000
H	-0.675695000	4.437849000	-3.839966000
C	-1.770174000	3.499179000	-2.248733000
C	0.598856000	-0.265556000	2.765430000
C	1.700761000	-0.999069000	3.169676000
C	-0.135125000	0.154442000	3.879056000
C	2.076457000	-1.379379000	4.446075000
C	0.141708000	-0.166364000	5.204318000
C	1.256094000	-0.951826000	5.487756000
H	2.972747000	-1.967034000	4.615697000
H	-0.508384000	0.201450000	5.992441000
H	1.492705000	-1.217796000	6.513348000
H	0.627854000	-0.025758000	-0.893391000
F	-1.216353000	0.962227000	3.686700000
F	2.637056000	-1.412763000	2.162240000
C	-1.042891000	3.877068000	1.523954000
H	-1.739422000	3.064530000	1.766839000
C	-2.579017000	2.642703000	-3.205750000
H	-3.408381000	2.181543000	-2.654229000
C	-3.306451000	-1.341983000	-2.227764000
H	-2.970641000	-0.338905000	-1.927506000
C	-4.623351000	-1.404978000	2.701291000
H	-4.793156000	-0.341570000	2.491950000
Al	2.122600000	-0.721702000	0.092695000
N	2.578034000	-2.431139000	-0.622286000
N	3.849864000	0.065032000	-0.093362000
C	4.968485000	-0.660286000	-0.199171000
C	4.950009000	-2.054912000	-0.362229000
C	3.846727000	-2.871664000	-0.648631000
C	4.129277000	-4.286903000	-1.058296000
C	2.365023000	-2.597147000	-3.534277000
H	3.187590000	-2.148727000	-2.962268000
H	4.076031000	-4.392779000	-2.147598000
H	5.128835000	-4.589655000	-0.742681000
H	3.390346000	-4.978915000	-0.644476000
H	6.559643000	0.279176000	0.876911000
C	6.302317000	0.019900000	-0.155675000
H	7.089930000	-0.625398000	-0.546645000
H	6.290889000	0.957662000	-0.718167000
H	5.918385000	-2.541844000	-0.387953000
C	1.550881000	-3.259847000	-1.198366000
C	1.508299000	-3.427867000	-2.597261000
C	0.553771000	-4.296592000	-3.130981000
C	-0.357078000	-4.952951000	-2.313771000
C	-0.356406000	-4.708629000	-0.946411000
C	0.585131000	-3.857636000	-0.363095000
H	0.507594000	-4.435115000	-4.209187000
C	0.577769000	-3.591031000	1.125350000
H	-1.107762000	-5.175188000	-0.314585000
H	0.904957000	-2.553965000	1.258773000
C	3.885695000	1.504139000	-0.090591000
C	3.486102000	2.166032000	-1.272434000
C	3.124845000	1.393197000	-2.526729000
C	4.207687000	2.223546000	1.075561000
C	4.170892000	3.620290000	1.018103000
C	3.807928000	4.287948000	-0.141163000
C	3.461677000	3.560927000	-1.273268000
H	3.155763000	4.086080000	-2.173442000
H	2.702950000	0.426562000	-2.217819000
C	4.527868000	1.564946000	2.402839000
H	4.540086000	0.477100000	2.258104000
H	4.413599000	4.187478000	1.914622000
H	-4.091129000	-5.286129000	0.163220000
H	0.834589000	5.697350000	-2.333557000
H	-1.093150000	-5.625785000	-2.747466000
H	3.778349000	5.374594000	-0.161551000
C	-3.347896000	-1.510644000	3.540457000
H	-3.157094000	-2.555742000	3.813607000
H	-2.475689000	-1.147303000	2.986322000
H	-3.427293000	-0.926437000	4.463462000

C	-5.830759000	-1.928171000	3.473538000
H	-5.687518000	-2.966460000	3.791918000
H	-5.993792000	-1.335874000	4.379084000
H	-6.750052000	-1.894048000	2.878519000
C	-4.508625000	-1.141660000	-3.150196000
H	-4.223252000	-0.585328000	-4.050719000
H	-4.925190000	-2.104249000	-3.469089000
H	-5.307928000	-0.579712000	-2.654214000
C	-2.134619000	-1.987717000	-2.954875000
H	-1.266783000	-2.087006000	-2.292151000
H	-2.377270000	-2.983496000	-3.341828000
H	-1.836461000	-1.374503000	-3.811240000
C	0.318917000	3.474321000	2.084721000
H	0.247603000	3.276113000	3.159016000
H	0.683227000	2.556434000	1.601036000
H	1.072501000	4.257675000	1.937153000
C	-1.564429000	5.149522000	2.190146000
H	-2.568501000	5.411336000	1.838916000
H	-1.610577000	5.029316000	3.277217000
H	-0.908411000	6.001948000	1.977121000
C	-1.690198000	1.511336000	-3.729306000
H	-1.240824000	0.937681000	-2.908026000
H	-2.260251000	0.821743000	-4.362921000
H	-0.867498000	1.915903000	-4.331313000
C	-3.180291000	3.430867000	-4.365788000
H	-3.807068000	2.781930000	-4.986076000
H	-3.800114000	4.265583000	-4.021984000
H	-2.404420000	3.847632000	-5.017248000
C	1.513717000	-1.449630000	-4.085608000
H	0.686651000	-1.840863000	-4.690107000
H	1.073321000	-0.851321000	-3.278260000
H	2.110200000	-0.786195000	-4.722681000
C	2.982767000	-3.400836000	-4.674576000
H	3.560171000	-4.258151000	-4.312776000
H	2.219765000	-3.787262000	-5.358749000
H	3.654273000	-2.770540000	-5.266346000
C	1.572033000	-4.483745000	1.863771000
H	2.591108000	-4.358402000	1.482345000
H	1.587820000	-4.240061000	2.931364000
H	1.301547000	-5.541792000	1.762290000
C	-0.807245000	-3.646529000	1.753282000
H	-1.223397000	-4.661334000	1.782397000
H	-0.753990000	-3.282344000	2.785396000
H	-1.511279000	-3.002114000	1.213006000
C	3.438097000	1.889199000	3.427161000
H	2.444103000	1.617476000	3.061615000
H	3.609101000	1.351529000	4.365658000
H	3.429399000	2.962543000	3.651917000
C	5.889936000	1.999347000	2.945952000
H	5.888938000	3.066167000	3.195896000
H	6.132517000	1.453412000	3.862983000
H	6.703206000	1.839397000	2.230781000
C	4.370210000	1.081821000	-3.357245000
H	4.867438000	2.003602000	-3.680641000
H	5.098182000	0.491063000	-2.790083000
H	4.108603000	0.509542000	-4.255066000
C	2.050835000	2.082728000	-3.357008000
H	1.167564000	2.305259000	-2.748711000
H	2.402457000	3.022338000	-3.798913000
H	1.739914000	1.437580000	-4.184656000

Int-5'_AlPdPCy$_3$ _M06L.log

SCF (M06L) = 2847.10693426
E(SCF)+ZPE(0 K) = 2845.901519
H(298 K) = 2845.835476
G(298 K) = 2846.000697
Lowest Frequency = 13.7612 cm^{-1}

Pd	0.586485	-0.291779	-0.521448
P	2.795749	-0.245927	0.397426
C	3.472167	1.488105	0.368172
H	4.495734	1.488710	0.782065
C	2.600996	2.420538	1.221712
H	1.567790	2.389774	0.832242
H	2.543047	2.073266	2.261190
C	3.120728	3.852386	1.182723
H	2.491048	4.495514	1.808753
H	4.130281	3.882885	1.621189
C	3.171123	4.375966	-0.245646
H	3.558262	5.401666	-0.272235
H	2.143025	4.417990	-0.636549
C	4.009127	3.461839	-1.131692
H	5.058657	3.506670	-0.803424
H	3.997104	3.814967	-2.169808
C	3.522389	2.017830	-1.070341
H	4.153168	1.379270	-1.700002
H	2.507697	1.945698	-1.493693
C	4.146598	-1.267870	-0.372754
H	4.368512	-0.703376	-1.291927
C	5.457682	-1.442072	0.393491
H	5.269546	-2.043793	1.295356
H	5.845429	-0.474814	0.739379
C	6.493884	-2.161656	-0.466549
H	6.731896	-1.535266	-1.338911
H	7.431185	-2.286687	0.088681
C	5.969426	-3.511485	-0.944172
H	5.827132	-4.166911	-0.071648
H	6.710307	-4.007260	-1.582215
C	4.640144	-3.363647	-1.677045
H	4.257683	-4.345427	-1.979849
H	4.796239	-2.793544	-2.604895
C	3.609831	-2.631844	-0.824591
H	2.672011	-2.493195	-1.375194
H	3.368804	-3.240411	0.060307
C	2.734598	-0.614652	2.226385
H	1.874717	0.010710	2.519700
C	2.305938	-2.056940	2.506700
H	1.468067	-2.331014	1.850355
H	3.136086	-2.740021	2.265993
C	1.919724	-2.226323	3.972622
H	1.629310	-3.263896	4.176716
H	1.028725	-1.611108	4.172710
C	3.048669	-1.791538	4.901337
H	2.741970	-1.881694	5.949968
H	3.902595	-2.473279	4.771285
C	3.504008	-0.367873	4.597079
H	4.334267	-0.081155	5.253313
H	2.681297	0.331154	4.815468
C	3.904704	-0.217233	3.130966
H	4.773113	-0.857143	2.931115
H	4.229092	0.811434	2.929399
Al	-1.687763	0.060195	-0.291303
N	-2.576948	1.684935	0.166862
N	-3.228655	-1.054957	-0.197517
C	-3.896621	1.858006	0.013888
C	-4.769044	0.800686	-0.284667
H	-5.812544	1.065171	-0.411291
C	-4.478121	-0.570897	-0.281375
C	-4.484792	3.224543	0.191770
H	-4.178684	3.664300	1.146209
H	-5.573749	3.198711	0.144480
H	-4.117847	3.903140	-0.585748
C	-5.636604	-1.522127	-0.315077
H	-5.843275	-1.907612	0.689836
H	-5.425494	-2.392808	-0.942059
H	-6.538814	-1.024470	-0.673559
C	-1.773552	2.772188	0.665241
C	-1.421076	2.764443	2.031716

C	-0.671710	3.834841	2.524911
H	-0.398168	3.853264	3.576388
C	-0.262437	4.871372	1.694934
C	-0.569204	4.830876	0.342727
H	-0.211876	5.620182	-0.315828
C	-1.321144	3.785333	-0.200312
C	-3.049085	-2.463208	0.047554
C	-2.570775	-3.291622	-0.988112
C	-2.438722	-4.657081	-0.729367
H	-2.077967	-5.312084	-1.517188
C	-2.745770	-5.187951	0.517677
C	-3.158086	-4.347079	1.541715
H	-3.351127	-4.756508	2.530915
C	-3.309201	-2.973958	1.333320
C	0.402876	-0.101824	-2.629952
C	1.430399	-0.305999	-3.553741
C	-0.767919	0.240258	-3.283886
C	1.318360	-0.197599	-4.936339
C	-1.001771	0.372849	-4.641319
C	0.084754	0.144080	-5.483813
H	2.186252	-0.379061	-5.562565
H	-1.981185	0.648069	-5.018563
H	-0.031130	0.233932	-6.559420
H	-0.301545	-0.332113	0.976849
F	-1.929800	0.517231	-2.483162
F	2.666572	-0.640712	-3.086201
H	-2.639319	-6.254280	0.696874
H	0.313875	5.699154	2.099651
C	-2.195520	-2.706213	-2.332509
C	-3.417120	-2.417916	-3.203167
C	-1.167933	-3.530020	-3.095673
H	-1.708997	-1.746976	-2.121956
H	-4.097389	-1.701561	-2.732525
H	-3.107462	-1.991865	-4.163343
H	-3.979341	-3.336645	-3.409198
H	-0.294518	-3.753830	-2.475052
H	-1.581659	-4.476915	-3.461844
H	-0.819554	-2.967000	-3.967123
C	-3.626271	-2.075472	2.513506
C	-2.368323	-1.917762	3.371588
C	-4.792250	-2.578861	3.359791
H	-3.895487	-1.079317	2.138682
H	-1.529729	-1.517511	2.788745
H	-2.552192	-1.245947	4.218050
H	-2.052635	-2.886237	3.776882
H	-5.696420	-2.742876	2.764202
H	-4.553531	-3.525477	3.856146
H	-5.034987	-1.856397	4.145675
C	-1.541829	3.740983	-1.698154
C	-0.216152	3.414037	-2.389881
C	-2.125325	5.039246	-2.251881
H	-2.243639	2.928934	-1.920174
H	0.244514	2.514911	-1.963656
H	-0.356375	3.241548	-3.462472
H	0.492871	4.243218	-2.271182
H	-3.063659	5.321254	-1.762216
H	-1.430300	5.876471	-2.125438
H	-2.322426	4.944083	-3.323997
C	-1.851825	1.640632	2.955561
C	-3.224748	1.912035	3.571029
C	-0.814094	1.338289	4.030396
H	-1.948062	0.731203	2.347413
H	-4.001985	2.004764	2.804744
H	-3.520191	1.095298	4.240090
H	-3.218658	2.838417	4.157060
H	0.173820	1.168574	3.587069
H	-0.723583	2.149291	4.761100
H	-1.091576	0.437738	4.586547

Int-6_AlPdAl _M06L.log

SCF (M06L) = -3041.28303438
E(SCF)+ZPE(0 K) = -3039.922237
H(298 K) = -3039.840842
G(298 K) = -3040.037543
Lowest Frequency = 12.9692 cm^{-1}

Pd	-0.340850000	0.450527000	1.050150000
Al	2.140024000	0.475855000	0.370094000
N	3.249900000	1.989419000	-0.170376000
N	3.544925000	-0.335397000	1.459289000
C	4.221559000	2.466079000	0.612317000
C	4.712556000	1.768648000	1.724786000
H	5.478691000	2.270770000	2.303913000
C	4.477469000	0.427453000	2.049593000
C	4.857482000	3.787815000	0.295009000
H	5.117630000	3.861676000	-0.764769000
H	5.754244000	3.947133000	0.894848000
H	4.160266000	4.608068000	0.495701000
C	5.397259000	-0.185610000	3.064121000
H	4.867583000	-0.854162000	3.746389000
H	5.912620000	0.585604000	3.638034000
H	6.158108000	-0.796893000	2.565480000
C	2.917809000	2.710702000	-1.364634000
C	3.385825000	2.205645000	-2.593806000
C	3.019153000	2.876811000	-3.761849000
H	3.368626000	2.505440000	-4.721643000
C	2.207496000	4.004389000	-3.717548000
H	1.929617000	4.510782000	-4.638122000
C	1.744832000	4.475508000	-2.496429000
H	1.091549000	5.345543000	-2.465988000
C	2.083879000	3.841747000	-1.298888000
C	4.277968000	0.978966000	-2.644448000
H	4.003555000	0.342081000	-1.793561000
C	4.078816000	0.147539000	-3.905678000
H	3.022836000	-0.101319000	-4.056009000
H	4.639486000	-0.789749000	-3.833250000
H	4.437742000	0.665427000	-4.802296000
C	5.749848000	1.350956000	-2.460307000
H	6.084458000	2.038619000	-3.246003000
H	6.384600000	0.458210000	-2.504539000
H	5.929444000	1.832705000	-1.493457000
C	1.491065000	4.344335000	0.004030000
H	1.978706000	3.816883000	0.833557000
C	-0.002827000	4.029231000	0.089192000
H	-0.414115000	4.358528000	1.049552000
H	-0.208067000	2.954222000	-0.001580000
H	-0.554487000	4.549670000	-0.704581000
C	1.714168000	5.844656000	0.195641000
H	2.767838000	6.129489000	0.103277000
H	1.363259000	6.163017000	1.182235000
H	1.157801000	6.426892000	-0.547452000
C	3.566780000	-1.743891000	1.741966000
C	2.681985000	-2.281339000	2.697404000
C	2.665279000	-3.668851000	2.868439000
H	1.985180000	-4.099138000	3.601884000
C	3.503059000	-4.498977000	2.136887000
H	3.465490000	-5.575805000	2.278364000
C	4.410665000	-3.942766000	1.244264000
H	5.085991000	-4.590468000	0.688159000
C	4.469605000	-2.564519000	1.035815000
C	1.839357000	-1.415669000	3.619262000
H	1.922714000	-0.372782000	3.288578000
C	0.357684000	-1.779253000	3.596626000
H	0.192483000	-2.825567000	3.884056000
H	-0.081412000	-1.611993000	2.604334000
H	-0.193087000	-1.152372000	4.305850000
C	2.363134000	-1.506535000	5.055309000
H	2.263951000	-2.527399000	5.442751000

H	1.792579000	-0.845194000	5.715444000
H	3.418504000	-1.228953000	5.138448000
C	5.491332000	-2.009801000	0.057381000
H	5.490017000	-0.914795000	0.134062000
C	5.154224000	-2.364226000	-1.391232000
H	5.038299000	-3.447360000	-1.512635000
H	5.956521000	-2.036677000	-2.062502000
H	4.224051000	-1.897687000	-1.720687000
C	6.902877000	-2.496664000	0.389470000
H	7.173457000	-2.309880000	1.433877000
H	7.641204000	-1.996085000	-0.245518000
H	7.004784000	-3.573950000	0.217918000
Al	-2.513778000	-0.418379000	0.437620000
N	-3.664149000	-1.858798000	-0.231445000
N	-4.095277000	0.609308000	1.012385000
C	-4.953025000	-2.061702000	0.095319000
C	-5.713689000	-1.146170000	0.828261000
H	-6.734094000	-1.436158000	1.051814000
C	-5.337811000	0.155811000	1.191230000
C	-5.644246000	-3.305613000	-0.381162000
H	-5.837607000	-3.254556000	-1.458562000
H	-6.599168000	-3.443372000	0.127823000
H	-5.020613000	-4.191477000	-0.228084000
C	-6.384485000	1.049541000	1.788006000
H	-6.150842000	1.271375000	2.835098000
H	-7.372306000	0.588739000	1.746709000
H	-6.418194000	2.015351000	1.274362000
C	-3.085835000	-2.743369000	-1.208180000
C	-3.401605000	-2.542472000	-2.569155000
C	-2.848632000	-3.405688000	-3.515319000
H	-3.079028000	-3.255205000	-4.568284000
C	-2.003275000	-4.441460000	-3.136868000
H	-1.583378000	-5.106703000	-3.887037000
C	-1.684685000	-4.609052000	-1.797809000
H	-1.009789000	-5.409151000	-1.499528000
C	-2.208926000	-3.768353000	-0.810876000
C	-4.261546000	-1.381673000	-3.034270000
H	-4.770418000	-0.948810000	-2.163629000
C	-3.368393000	-0.293440000	-3.628665000
H	-2.833942000	-0.670096000	-4.509363000
H	-2.608890000	0.040872000	-2.911753000
H	-3.957595000	0.577241000	-3.938884000
C	-5.338529000	-1.796540000	-4.033064000
H	-5.985060000	-0.946371000	-4.273548000
H	-5.972685000	-2.600584000	-3.645407000
H	-4.905214000	-2.148273000	-4.975517000
C	-1.822895000	-4.011473000	0.633860000
H	-2.307786000	-3.245680000	1.254379000
C	-2.314968000	-5.376100000	1.115397000
H	-3.399545000	-5.483743000	1.007651000
H	-2.065643000	-5.527570000	2.170409000
H	-1.847014000	-6.188158000	0.547362000
C	-0.314938000	-3.873670000	0.823226000
H	-0.029162000	-4.095388000	1.856137000
H	0.022714000	-2.856527000	0.594601000
H	0.241883000	-4.559546000	0.174306000
C	-3.807609000	1.992018000	1.273401000
C	-3.573040000	2.440425000	2.585788000
C	-3.299177000	3.796905000	2.779882000
H	-3.112877000	4.158726000	3.788837000
C	-3.251926000	4.681868000	1.712293000
H	-3.044940000	5.734825000	1.883963000
C	-3.435466000	4.211466000	0.417657000
H	-3.357115000	4.901943000	-0.417939000
C	-3.694113000	2.863058000	0.168818000
C	-3.535073000	1.494520000	3.769155000
H	-3.948804000	0.529583000	3.449510000
C	-2.085888000	1.255393000	4.197047000
H	-1.625145000	2.190504000	4.536469000
H	-1.477257000	0.875392000	3.361188000
H	-2.035068000	0.535407000	5.021682000
C	-4.366805000	1.992784000	4.949328000
H	-4.386241000	1.246345000	5.749372000
H	-5.402475000	2.211404000	4.666977000
H	-3.947397000	2.910120000	5.376406000
C	-3.869275000	2.357075000	-1.251099000
H	-3.546422000	1.304828000	-1.265945000
C	-2.995726000	3.089662000	-2.262148000
H	-3.285172000	4.140135000	-2.376088000
H	-3.092141000	2.626005000	-3.249046000
H	-1.939342000	3.052859000	-1.977740000
C	-5.339417000	2.368443000	-1.670494000
H	-5.748356000	3.384925000	-1.635738000
H	-5.952767000	1.737878000	-1.017837000
H	-5.457748000	1.995389000	-2.694673000
H	1.148072000	1.246219000	1.571177000
C	1.477720000	-0.470404000	-1.288193000
C	0.702734000	0.214998000	-2.229202000
C	1.573909000	-1.830596000	-1.580417000
C	0.158797000	-0.330514000	-3.382242000
C	1.036897000	-2.462144000	-2.694805000
C	0.331068000	-1.692808000	-3.612737000
H	-0.404255000	0.305292000	-4.057487000
H	1.165549000	-3.532463000	-2.817550000
H	-0.102580000	-2.157310000	-4.493983000
F	2.225380000	-2.631093000	-0.696349000
F	0.415781000	1.521261000	-1.994630000

Int-6'_AlPdPCy$_3$ _M06L.log

SCF (M06L) = -2847.12597620
E(SCF)+ZPE(0 K) = -2845.917569
H(298 K) = -2845.852000
G(298 K) = -2846.015320
Lowest Frequency = 17.8638 cm^{-1}

Pd	1.020768	-0.501839	-1.185697
P	3.040567	-0.404825	-0.048531
C	4.431033	-0.702623	-1.248070
H	5.394874	-0.661680	-0.714804
C	4.446542	0.351001	-2.359706
H	3.453225	0.361189	-2.838558
H	4.595118	1.355669	-1.944468
C	5.521469	0.057458	-3.401152
H	5.497398	0.818628	-4.189472
H	6.511958	0.135743	-2.928399
C	5.359129	-1.336252	-3.994571
H	6.154852	-1.541990	-4.719535
H	4.412095	-1.381691	-4.551728
C	5.341925	-2.395055	-2.899495
H	6.323164	-2.419952	-2.401458
H	5.189369	-3.392381	-3.327410
C	4.263416	-2.098221	-1.864135
H	4.258541	-2.871645	-1.085751
H	3.267992	-2.142359	-2.338371
C	3.357837	-1.681376	1.279677
H	2.884174	-2.582850	0.855015
C	4.812906	-2.040053	1.604512
H	5.321101	-1.171510	2.045016
H	5.372436	-2.294578	0.696986
C	4.868219	-3.209747	2.584865
H	4.443326	-4.100100	2.097582
H	5.910218	-3.456331	2.819788
C	4.085134	-2.914836	3.858652
H	4.571388	-2.085631	4.394327
H	4.114163	-3.776478	4.535418
C	2.646144	-2.524425	3.542087
H	2.098615	-2.274204	4.458589
H	2.121162	-3.382344	3.095427
C	2.596555	-1.354118	2.567782
H	1.561181	-1.075178	2.340715
H	3.055059	-0.475873	3.046716
C	3.411903	1.278013	0.659844

H	3.542766	1.884301	-0.251213
C	2.197709	1.871620	1.384426
H	1.308397	1.781065	0.746446
H	1.976010	1.289512	2.290025
C	2.438771	3.326204	1.766083
H	1.567990	3.716993	2.305770
H	2.520514	3.927771	0.847121
C	3.713963	3.484251	2.586179
H	3.888106	4.537666	2.834035
H	3.594597	2.957572	3.545159
C	4.916573	2.904536	1.849227
H	5.827857	3.009489	2.449609
H	5.086218	3.479227	0.926127
C	4.689348	1.438843	1.486233
H	4.606584	0.853579	2.413394
H	5.555944	1.038018	0.943441
Al	-1.349910	0.026899	-0.549143
N	-2.978004	-0.928233	-1.083772
N	-2.039205	1.791242	-1.058501
C	-3.718607	-0.491164	-2.104273
C	-3.580649	0.795002	-2.645584
H	-4.213382	1.020604	-3.495801
C	-2.885524	1.881426	-2.099610
C	-4.776166	-1.375102	-2.698605
H	-5.385860	-1.846914	-1.922961
H	-4.319564	-2.192726	-3.266745
H	-5.425742	-0.814287	-3.371467
C	-3.166872	3.221222	-2.717435
H	-2.305566	3.564336	-3.297714
H	-3.355055	3.985728	-1.959005
H	-4.026079	3.168214	-3.386700
C	-3.302379	-2.197801	-0.501947
C	-2.869937	-3.390829	-1.110712
C	-3.168449	-4.596049	-0.471446
H	-2.833617	-5.528524	-0.921486
C	-3.859780	-4.624821	0.732675
H	-4.075285	-5.574682	1.214686
C	-4.272047	-3.435598	1.321238
H	-4.811284	-3.459466	2.265380
C	-4.003193	-2.204631	0.719610
C	-2.045262	-3.409066	-2.384985
H	-2.056207	-2.401750	-2.820757
C	-0.582270	-3.752287	-2.094801
H	-0.106165	-2.998918	-1.456884
H	-0.010913	-3.804667	-3.028308
H	-0.502357	-4.724163	-1.592925
C	-2.613822	-4.381443	-3.418625
H	-2.516581	-5.420646	-3.085639
H	-2.072744	-4.295806	-4.365969
H	-3.676413	-4.205710	-3.618209
C	-4.490540	-0.913155	1.345783
H	-3.847549	-0.108002	0.970156
C	-4.386278	-0.902714	2.866101
H	-4.605441	0.098845	3.252147
H	-3.382832	-1.180957	3.202568
H	-5.102717	-1.588323	3.332905
C	-5.917253	-0.593962	0.896562
H	-5.979890	-0.443589	-0.186526
H	-6.282592	0.320205	1.377570
H	-6.602539	-1.407338	1.162475
C	-1.545495	3.028104	-0.508560
C	-2.195769	3.552831	0.630737
C	-1.723636	4.748071	1.172282
H	-2.206494	5.161473	2.053074
C	-0.635119	5.410914	0.616175
H	-0.273496	6.334366	1.060692
C	-0.015648	4.887960	-0.507214
H	0.831406	5.410183	-0.948668
C	-0.461740	3.702948	-1.103761
C	-3.369026	2.824039	1.262780
H	-3.137799	1.751072	1.210544
C	-3.577779	3.169732	2.732901
H	-3.950215	4.192618	2.861672
H	-2.655892	3.067868	3.310394
H	-4.329524	2.503967	3.169074
C	-4.675985	3.047820	0.498243
H	-4.894214	4.118139	0.402252
H	-5.513465	2.588791	1.034884
H	-4.659599	2.612257	-0.504429
C	0.221076	3.246767	-2.381550
H	-0.320097	2.375657	-2.775039
C	1.661241	2.808474	-2.130356
H	2.239145	3.606575	-1.644942
H	2.159494	2.561314	-3.074668
H	1.692410	1.911634	-1.498446
C	0.217610	4.352432	-3.441943
H	-0.770902	4.794477	-3.599639
H	0.573157	3.964360	-4.401510
H	0.888696	5.170030	-3.156110
C	-1.054518	-0.420298	1.411296
C	-0.918863	-1.752532	1.817239
C	-0.886584	0.464278	2.475862
C	-0.778368	-2.193622	3.126432
C	-0.715524	0.112266	3.809783
C	-0.688381	-1.239580	4.135895
H	-0.715479	-3.257579	3.328400
H	-0.607572	0.889805	4.558537
H	-0.570341	-1.549457	5.170001
H	-0.467227	-0.438337	-2.036416
F	-0.855661	1.794838	2.205076
F	-0.877541	-2.709639	0.858602

Int-7_AlPdAl _M06L.log

SCF (M06L) = -3041.33029951
E(SCF)+ZPE(0 K) = -3039.968488
H(298 K) = -3039.887629
G(298 K) = -3040.079614
Lowest Frequency = 18.5541 cm^{-1}

Pd	-0.377260000	0.063655000	0.873190000
Al	-2.750988000	0.449512000	0.164209000
N	-3.736761000	-0.890775000	-0.803300000
N	-3.022324000	1.838637000	-1.147168000
C	-4.806406000	-0.542582000	-1.526648000
C	-5.041648000	0.777570000	-1.939478000
H	-5.959887000	0.949741000	-2.489272000
C	-4.172189000	1.876387000	-1.834321000
C	-5.792525000	-1.597709000	-1.931713000
H	-5.299118000	-2.441626000	-2.422846000
H	-6.558639000	-1.198250000	-2.597157000
H	-6.283053000	-2.010812000	-1.042313000
C	-4.559079000	3.134612000	-2.554255000
H	-4.789577000	3.930528000	-1.837236000
H	-5.432470000	2.977579000	-3.188106000
H	-3.734636000	3.509398000	-3.168641000
C	-3.392194000	-2.274419000	-0.668119000
C	-2.490048000	-2.825536000	-1.600013000
C	-2.137740000	-4.169950000	-1.463917000
H	-1.439937000	-4.612370000	-2.171740000
C	-2.659960000	-4.947867000	-0.437599000
C	-3.530801000	-4.380933000	0.484487000
H	-3.919072000	-4.984857000	1.302243000
C	-3.905714000	-3.037591000	0.395728000

C	-2.151505000	2.981343000	-1.178761000
C	-2.419625000	4.093303000	-0.356398000
C	-1.559610000	5.192605000	-0.428427000
H	-1.753703000	6.056003000	0.204814000
C	-0.455258000	5.188223000	-1.267706000
C	-0.179519000	4.065800000	-2.039224000
H	0.708372000	4.055496000	-2.665351000
C	-1.012793000	2.945384000	-2.012102000
C	0.261063000	0.328729000	2.790218000
C	0.961015000	0.516488000	3.953932000
C	-1.076572000	0.494060000	2.765403000
C	0.261158000	0.856215000	5.113689000
C	-1.846805000	0.832399000	3.867402000
C	-1.128008000	1.007925000	5.061261000
H	0.805016000	1.001435000	6.042528000
H	-2.923507000	0.968424000	3.820551000
H	-1.658449000	1.275430000	5.972786000
H	-1.128577000	-0.187467000	-0.575126000
F	-3.832403000	0.854469000	1.399100000
F	2.320305000	0.391438000	4.007390000
C	-3.547067000	4.095040000	0.656210000
H	-4.212553000	3.251681000	0.440588000
C	-0.703718000	1.729906000	-2.863719000
H	-1.001369000	0.852049000	-2.278826000
C	-1.937573000	-1.999688000	-2.744517000
H	-2.100567000	-0.943232000	-2.498257000
C	-4.784352000	-2.417326000	1.461689000
H	-5.130357000	-1.443709000	1.095190000
Al	1.957731000	-0.369578000	0.345994000
N	2.939814000	-2.024042000	0.583603000
N	3.548659000	0.724580000	0.503725000
C	4.656858000	0.330833000	1.153145000
C	4.882030000	-0.993456000	1.550249000
C	4.136484000	-2.120277000	1.179292000
C	4.744927000	-3.471504000	1.418407000
H	5.802635000	-1.180916000	2.090806000
H	5.656604000	-3.392113000	2.011844000
H	4.048910000	-4.148297000	1.919862000
H	4.993782000	-3.950757000	0.465383000
C	5.745205000	1.325720000	1.420635000
H	6.425649000	0.961129000	2.191222000
H	5.337302000	2.293562000	1.723491000
C	3.540276000	2.024969000	-0.121036000
C	4.210150000	2.193031000	-1.352244000
C	4.957919000	1.072040000	-2.051372000
H	4.961708000	0.189821000	-1.398315000
C	4.153930000	3.442897000	-1.973885000
H	4.658175000	3.578676000	-2.928934000
C	3.466774000	4.504411000	-1.400829000
C	2.814953000	4.321492000	-0.189717000
H	2.269849000	5.147413000	0.261752000
C	2.829866000	3.089777000	0.469522000
C	2.117815000	2.952166000	1.796424000
H	6.332135000	1.508384000	0.513935000
H	2.088244000	1.890530000	2.050618000
C	3.349418000	-2.580093000	-2.200590000
H	3.039975000	-1.554848000	-1.929338000
H	2.227859000	-4.968373000	-2.856841000
C	2.051032000	-4.715107000	-1.815703000
C	2.575158000	-3.530874000	-1.297074000
C	2.335163000	-3.225469000	0.065722000
C	1.521385000	-4.047830000	0.869518000
C	1.023125000	-3.780970000	2.288141000
H	1.014409000	-4.771163000	2.769830000
C	1.023268000	-5.222495000	0.288735000
C	1.290789000	-5.567869000	-1.024410000
H	0.397138000	-5.869106000	0.901486000
H	0.206816000	6.050389000	-1.308158000
H	-2.374873000	-5.993836000	-0.349498000
H	0.891204000	-6.489518000	-1.440069000
H	3.434658000	5.469795000	-1.899884000
C	-4.377193000	5.375740000	0.620897000
H	-4.773154000	5.588414000	-0.378233000
H	-5.225101000	5.300241000	1.308400000
H	-3.790471000	6.248050000	0.929640000
C	-2.969461000	3.865683000	2.053639000
H	-3.767412000	3.763430000	2.796360000
H	-2.365023000	2.954151000	2.093137000
H	-2.329007000	4.706081000	2.349562000
C	0.782258000	1.576643000	-3.159179000
H	1.382445000	1.651713000	-2.243589000
H	0.975781000	0.597193000	-3.612418000
H	1.154629000	2.333262000	-3.860984000
C	-1.516679000	1.707377000	-4.159024000
H	-1.225213000	0.852384000	-4.780068000
H	-2.590913000	1.616994000	-3.967271000
H	-1.350795000	2.617082000	-4.748553000
C	-3.956945000	-2.155759000	2.721147000
H	-3.606516000	-3.100357000	3.155673000
H	-3.075693000	-1.540586000	2.507854000
H	-4.548260000	-1.628748000	3.476987000
C	-6.011399000	-3.265205000	1.783808000
H	-5.735678000	-4.222379000	2.240387000
H	-6.659973000	-2.745656000	2.495666000
H	-6.602919000	-3.488697000	0.889403000
C	-2.686092000	-2.283740000	-4.047601000
H	-2.268106000	-1.698179000	-4.873533000
H	-2.613760000	-3.343403000	-4.319682000
H	-3.748581000	-2.031532000	-3.971241000
C	-0.438704000	-2.199822000	-2.928905000
H	0.107080000	-1.999666000	-1.998470000
H	-0.196270000	-3.221922000	-3.244528000
H	-0.058392000	-1.521243000	-3.702122000
C	3.027855000	-2.766075000	-3.680004000
H	3.466362000	-1.950676000	-4.262458000
H	3.451845000	-3.698605000	-4.068970000
H	1.952564000	-2.779378000	-3.873093000
C	4.867383000	-2.659271000	-2.011829000
H	5.197893000	-2.300022000	-1.034629000
H	5.216969000	-3.691112000	-2.135465000
H	5.375719000	-2.049561000	-2.767420000
C	4.251143000	0.684759000	-3.349976000
H	4.181524000	1.542476000	-4.028837000
H	4.800001000	-0.106055000	-3.873651000
H	3.231481000	0.331667000	-3.168187000
C	6.410956000	1.442872000	-2.348188000
H	6.951248000	1.769462000	-1.454357000
H	6.948265000	0.587434000	-2.770784000
H	6.471660000	2.258115000	-3.077499000
C	2.879403000	3.657287000	2.918370000
H	2.355773000	3.527539000	3.871188000
H	3.891337000	3.257364000	3.043252000
H	2.966391000	4.733251000	2.725647000
C	0.676276000	3.447296000	1.729839000
H	0.117407000	2.955679000	0.922426000
H	0.157244000	3.229532000	2.669657000
H	0.621194000	4.529405000	1.560568000
C	1.807565000	-2.858350000	3.212684000
H	1.348176000	-2.886609000	4.206145000
H	2.855316000	-3.145268000	3.334235000
H	1.774776000	-1.817808000	2.890638000
C	-0.435895000	-3.314553000	2.208610000
H	-1.076973000	-4.058967000	1.727039000
H	-0.832551000	-3.103784000	3.208189000
H	-0.520102000	-2.388085000	1.623229000

Int-7'_AlPdPCy$_3$ _M06L.log

SCF (M06L) = -2847.16180980
E(SCF)+ZPE(0 K) = -2845.953805
H(298 K) = -2845.888297
G(298 K) = -2846.049945
Lowest Frequency = 19.0978 cm^{-1}

Pd	0.617569	-0.127658	-0.863132
P	2.391491	0.370393	0.609313
C	2.774596	2.175273	0.399858
H	3.676234	2.402637	0.992793

C	1.612245	3.024509	0.933075
H	0.703528	2.784384	0.355552
H	1.379248	2.770470	1.975474
C	1.919109	4.511943	0.812175
H	1.079858	5.096445	1.205132
H	2.791461	4.752967	1.439382
C	2.213405	4.890790	-0.633056
H	2.451918	5.957501	-0.713648
H	1.305248	4.727336	-1.232745
C	3.348672	4.043563	-1.196024
H	4.278492	4.283022	-0.658335
H	3.530567	4.289873	-2.248018
C	3.056328	2.552454	-1.058957
H	3.887992	1.964826	-1.466349
H	2.176731	2.291886	-1.666405
C	3.918499	-0.509867	0.030094
H	4.041079	-0.067726	-0.970708
C	5.213801	-0.274191	0.808338
H	5.139233	-0.747087	1.799692
H	5.377137	0.797251	0.985020
C	6.399526	-0.877095	0.058737
H	6.514544	-0.353091	-0.901590
H	7.327099	-0.708651	0.618009
C	6.188468	-2.364055	-0.202093
H	6.162182	-2.895871	0.761169
H	7.036788	-2.778601	-0.758318
C	4.883089	-2.613892	-0.949435
H	4.725614	-3.688077	-1.098864
H	4.941491	-2.164025	-1.949538
C	3.692925	-2.008986	-0.211479
H	2.767241	-2.156280	-0.782764
H	3.556301	-2.529679	0.746594
C	2.212461	0.178856	2.454112
H	1.191765	0.554024	2.640427
C	2.225649	-1.293966	2.878779
H	1.514318	-1.874058	2.273083
H	3.220577	-1.718355	2.679201
C	1.923143	-1.441170	4.366567
H	1.934305	-2.500272	4.649758
H	0.904304	-1.077934	4.564386
C	2.901112	-0.637872	5.213103
H	2.670196	-0.747534	6.278588
H	3.917840	-1.034786	5.073584
C	2.873260	0.829109	4.805907
H	3.583091	1.414112	5.401714
H	1.876015	1.242938	5.019085
C	3.176703	1.001526	3.320120
H	4.209829	0.684769	3.125648
H	3.127456	2.064013	3.056327
Al	-1.910002	-0.199084	-0.929876
N	-3.006298	1.098250	-0.022495
N	-2.784502	-1.735772	-0.172857
C	-4.320806	0.834632	0.015863
C	-4.834417	-0.468044	-0.087210
H	-5.914982	-0.556658	-0.072736
C	-4.116645	-1.676313	-0.041735
C	-5.291220	1.961931	0.206053
H	-5.045492	2.553889	1.093261
H	-6.315454	1.598634	0.295979
H	-5.236629	2.651464	-0.644425
C	-4.901058	-2.927659	0.221607
H	-4.971423	-3.120889	1.297488
H	-4.433289	-3.804880	-0.232189
H	-5.920803	-2.824640	-0.154643
C	-2.543230	2.414053	0.308661
C	-2.122825	2.670203	1.631823
C	-1.698202	3.962202	1.950058
H	-1.375839	4.184875	2.963793
C	-1.660290	4.966056	0.988329
C	-2.023585	4.681094	-0.319688
H	-1.960420	5.457632	-1.079080
C	-2.472369	3.408472	-0.685346
C	-2.077322	-2.924391	0.191853
C	-1.410666	-3.666952	-0.809145
C	-0.682593	-4.793061	-0.421263
H	-0.165208	-5.375439	-1.177556
C	-0.606767	-5.184315	0.910519
C	-1.251641	-4.434385	1.882150
H	-1.172081	-4.723011	2.928649
C	-1.987229	-3.293625	1.548610
C	1.651829	-0.302499	-2.602331
C	2.582872	-0.375891	-3.608340
C	0.342297	-0.383545	-2.887785
C	2.135197	-0.551534	-4.919928
C	-0.191470	-0.543131	-4.157739
C	0.764712	-0.631336	-5.181493
H	2.861847	-0.615938	-5.724516
H	-1.257361	-0.595195	-4.355686
H	0.436052	-0.760739	-6.210552
H	-0.557894	-0.048886	0.339022
F	-2.582831	-0.103441	-2.480098
F	3.923366	-0.275060	-3.382316
H	-0.038182	-6.067714	1.187892
H	-1.327854	5.965323	1.257974
C	-1.508132	-3.270178	-2.268499
C	-2.869723	-3.627141	-2.863766
C	-0.378256	-3.820140	-3.127813
H	-1.425760	-2.177916	-2.325577
H	-3.685915	-3.120704	-2.341121
H	-2.917444	-3.316893	-3.912624
H	-3.048753	-4.708134	-2.820411
H	0.604064	-3.565541	-2.714378
H	-0.435473	-4.909346	-3.237128
H	-0.431242	-3.387539	-4.130696
C	-2.561679	-2.452598	2.672418
C	-1.416182	-1.801278	3.445199
C	-3.437141	-3.257852	3.631328
H	-3.172547	-1.648328	2.243935
H	-0.782873	-1.192302	2.788971
H	-1.793034	-1.162328	4.252623
H	-0.777684	-2.567481	3.900091
H	-4.233912	-3.802121	3.115206
H	-2.846778	-3.998259	4.182297
H	-3.903500	-2.600062	4.372183
C	-2.798658	3.113457	-2.134516
C	-1.500134	2.943513	-2.925989
C	-3.684712	4.179663	-2.772518
H	-3.331524	2.157759	-2.183584
H	-0.845637	2.184916	-2.479080
H	-1.703212	2.628857	-3.954290
H	-0.939883	3.886403	-2.958033
H	-4.606710	4.342483	-2.203945
H	-3.173116	5.145623	-2.848131
H	-3.964280	3.884517	-3.788206
C	-2.108089	1.565447	2.672306
C	-3.490342	1.296053	3.272581
C	-1.110894	1.827277	3.796616
H	-1.797785	0.650200	2.146862
H	-4.203221	0.923793	2.532200
H	-3.423280	0.541071	4.064995
H	-3.905946	2.206945	3.719765
H	-0.128993	2.127015	3.414268

H	-1.456729	2.619369	4.470753
H	-0.974930	0.926733	4.404851

Int-8_AlPdAl _M06L.log

SCF (M06L) = -3041.35485858
E(SCF)+ZPE(0 K) = -3039.993648
H(298 K) = -3039.913475
G(298 K) = -3040.103641
Lowest Frequency = 15.1731 cm^{-1}

Pd	0.049354000	-0.072851000	-0.222827000
Al	-3.430222000	-0.225989000	0.468906000
N	-3.297702000	-1.681587000	-0.797945000
N	-3.558186000	1.136188000	-0.903072000
C	-4.250769000	-1.650174000	-1.745988000
C	-4.853154000	-0.456523000	-2.172218000
H	-5.656387000	-0.556070000	-2.893064000
C	-4.470541000	0.854794000	-1.849470000
C	-4.728597000	-2.930969000	-2.366316000
H	-3.906155000	-3.544859000	-2.740720000
H	-5.427976000	-2.740880000	-3.181403000
H	-5.235752000	-3.533943000	-1.604018000
C	-5.128692000	1.975656000	-2.597743000
H	-5.731023000	2.578832000	-1.908167000
H	-5.776666000	1.598666000	-3.389656000
H	-4.393050000	2.657205000	-3.033972000
C	-2.560465000	-2.904314000	-0.645225000
C	-1.493055000	-3.165040000	-1.535372000
C	-0.867940000	-4.412174000	-1.471179000
H	-0.049264000	-4.631918000	-2.151545000
C	-1.258614000	-5.369510000	-0.541366000
C	-2.258364000	-5.067986000	0.370688000
H	-2.542350000	-5.799576000	1.124936000
C	-2.922828000	-3.837419000	0.342497000
C	-3.120180000	2.499382000	-0.780395000
C	-3.826217000	3.388435000	0.051870000
C	-3.387999000	4.713675000	0.135544000
H	-3.921354000	5.404393000	0.785418000
C	-2.294136000	5.154839000	-0.592402000
C	-1.600649000	4.262890000	-1.402313000
H	-0.726242000	4.609365000	-1.944619000
C	-1.985472000	2.924160000	-1.508471000
C	-0.772022000	-0.177638000	1.657126000
C	0.038316000	-0.222343000	2.780924000
C	-2.163507000	-0.156518000	1.941967000
C	-0.352635000	-0.242104000	4.103793000
C	-2.610443000	-0.166832000	3.278855000
C	-1.726455000	-0.208699000	4.353957000
H	0.382881000	-0.279977000	4.902653000
H	-3.682302000	-0.157613000	3.480096000
H	-2.088559000	-0.222220000	5.378672000
H	1.645739000	0.108892000	-1.223057000
F	-5.016344000	-0.327610000	1.053088000
F	1.466981000	-0.233945000	2.598846000
C	-5.012128000	2.945746000	0.884153000
H	-5.321607000	1.948903000	0.552219000
C	-1.178811000	1.964496000	-2.364548000
H	-1.130498000	1.012613000	-1.798412000
C	-1.008575000	-2.130564000	-2.537716000
H	-1.206553000	-1.138460000	-2.093681000
C	-3.990498000	-3.562223000	1.381457000
H	-4.409964000	-2.564303000	1.209110000
Al	2.230870000	0.066679000	0.453246000
N	3.608652000	-1.211506000	0.731876000
N	3.206680000	1.626857000	0.923952000
C	4.335336000	1.562356000	1.644321000
C	4.969987000	0.352674000	1.969358000
C	4.706777000	-0.923670000	1.450654000
C	5.739316000	-1.987488000	1.676665000
H	5.859650000	0.438894000	2.582529000
H	6.531703000	-1.632348000	2.336057000
H	5.297219000	-2.890658000	2.101732000
H	6.189115000	-2.293500000	0.726564000
C	5.015976000	2.829498000	2.066412000
H	5.700057000	2.645786000	2.896181000
H	4.296670000	3.602148000	2.349216000
C	2.771237000	2.904853000	0.414382000
C	3.449050000	3.474403000	-0.680287000
C	4.570429000	2.762861000	-1.412212000
H	4.890283000	1.896422000	-0.818202000
C	3.002330000	4.706434000	-1.167471000
H	3.509668000	5.150482000	-2.021511000
C	1.916626000	5.355136000	-0.597072000
C	1.241202000	4.764210000	0.463764000
H	0.367984000	5.252820000	0.886513000
C	1.642982000	3.533237000	0.984356000
C	0.883585000	2.882940000	2.117548000
H	5.602603000	3.238341000	1.235698000
H	0.907819000	1.811583000	1.911118000
C	4.502567000	-1.244562000	-2.007874000
H	3.907221000	-0.406978000	-1.618334000
H	4.435539000	-3.763367000	-2.987498000
C	4.063519000	-3.726507000	-1.967936000
C	4.041242000	-2.504915000	-1.290782000
C	3.559398000	-2.479647000	0.039172000
C	3.046489000	-3.642861000	0.655615000
C	2.303001000	-3.725664000	1.984357000
H	2.287733000	-4.798682000	2.224719000
C	3.091176000	-4.833479000	-0.080525000
C	3.604963000	-4.889383000	-1.366320000
H	2.691535000	-5.734396000	0.381722000
H	-1.967656000	6.189998000	-0.521614000
H	-0.760574000	-6.335459000	-0.511715000
H	3.631516000	-5.832459000	-1.905356000
H	1.582023000	6.311857000	-0.990996000
C	-6.211525000	3.879087000	0.737559000
H	-6.506064000	4.011020000	-0.309258000
H	-7.073588000	3.480251000	1.280621000
H	-6.005038000	4.874936000	1.145438000
C	-4.601153000	2.823620000	2.351993000
H	-5.423836000	2.429047000	2.957089000
H	-3.743491000	2.154344000	2.475470000
H	-4.314204000	3.801519000	2.758587000
C	0.254868000	2.431862000	-2.594393000
H	0.756439000	2.690589000	-1.654711000
H	0.831880000	1.630946000	-3.067286000
H	0.298016000	3.304729000	-3.258119000
C	-1.826400000	1.636615000	-3.711207000
H	-1.146405000	1.019219000	-4.309046000
H	-2.761704000	1.080095000	-3.612218000
H	-2.028189000	2.551556000	-4.281600000
C	-3.380660000	-3.580731000	2.783458000
H	-3.022232000	-4.584256000	3.042845000
H	-2.535657000	-2.890505000	2.860722000
H	-4.123010000	-3.288112000	3.533381000
C	-5.146198000	-4.557693000	1.290237000
H	-4.806335000	-5.581398000	1.485952000
H	-5.918826000	-4.320252000	2.027887000
H	-5.617327000	-4.552856000	0.301931000
C	-1.749869000	-2.180724000	-3.877290000
H	-1.256189000	-1.530425000	-4.607210000
H	-1.742252000	-3.198852000	-4.285561000
H	-2.788532000	-1.849509000	-3.803352000
C	0.493965000	-2.237637000	-2.796434000
H	1.065270000	-2.333031000	-1.865814000
H	0.735692000	-3.097000000	-3.434861000
H	0.846017000	-1.336433000	-3.310881000
C	4.238962000	-1.305604000	-3.509710000
H	4.406957000	-0.324770000	-3.962607000
H	4.909871000	-2.007658000	-4.016579000
H	3.209634000	-1.606017000	-3.726733000
C	5.972821000	-0.908754000	-1.749174000
H	6.164400000	-0.640824000	-0.706075000
H	6.622301000	-1.752854000	-2.009851000
H	6.281106000	-0.053873000	-2.362486000
C	4.047248000	2.235770000	-2.749102000
H	3.681218000	3.059545000	-3.373181000
H	4.840037000	1.722530000	-3.305040000

H	3.214299000	1.537360000	-2.605102000
C	5.793533000	3.650351000	-1.632640000
H	6.164598000	4.086103000	-0.699241000
H	6.609311000	3.075443000	-2.082689000
H	5.571102000	4.480567000	-2.311537000
C	1.559719000	3.097795000	3.469924000
H	0.987274000	2.599647000	4.259693000
H	2.573957000	2.685706000	3.492849000
H	1.621196000	4.163843000	3.720281000
C	-0.591028000	3.257248000	2.170246000
H	-1.076122000	3.098688000	1.200194000
H	-1.103179000	2.622841000	2.902160000
H	-0.754056000	4.300949000	2.466857000
C	2.893824000	-3.042717000	3.215013000
H	2.238374000	-3.238268000	4.070286000
H	3.879850000	-3.439914000	3.475135000
H	2.965610000	-1.959855000	3.118629000
C	0.850080000	-3.308070000	1.751312000
H	0.362012000	-3.960126000	1.021607000
H	0.272208000	-3.341115000	2.682168000
H	0.778364000	-2.289930000	1.356469000

Int-8'_AlPdPCy$_3$ _M06L.log

SCF (M06L) = -2847.14408844
E(SCF)+ZPE(0 K) = -2845.935440
H(298 K) = -2845.870468
G(298 K) = -2846.029614
Lowest Frequency = 24.5882 cm^{-1}

Pd	-1.208691	1.069929	-0.318204
P	-2.424986	-0.769104	0.033696
C	-1.814374	-2.371474	-0.695711
H	-2.380696	-3.163869	-0.182591
C	-0.328517	-2.546073	-0.361095
H	0.225096	-1.698004	-0.788498
H	-0.176556	-2.491926	0.725695
C	0.243973	-3.845856	-0.911122
H	1.318154	-3.888962	-0.699339
H	-0.210595	-4.702515	-0.388372
C	-0.020521	-3.967531	-2.406152
H	0.396812	-4.902458	-2.798048
H	0.494153	-3.148574	-2.932503
C	-1.511913	-3.873459	-2.695709
H	-2.032190	-4.711657	-2.206669
H	-1.708217	-3.971199	-3.769431
C	-2.083441	-2.551531	-2.192211
H	-3.160650	-2.512863	-2.395580
H	-1.623643	-1.734095	-2.755127
C	-4.096094	-0.552841	-0.730565
H	-3.848294	-0.623646	-1.799689
C	-5.109794	-1.656756	-0.413173
H	-5.393983	-1.606532	0.648283
H	-4.666778	-2.649846	-0.568034
C	-6.359237	-1.492673	-1.274550
H	-6.084028	-1.639877	-2.329517
H	-7.089762	-2.273461	-1.033769
C	-6.971925	-0.107474	-1.102494
H	-7.326983	0.000968	-0.066542
H	-7.854683	0.001521	-1.742536
C	-5.954111	0.988618	-1.399269
H	-6.399924	1.978339	-1.249588
H	-5.662929	0.937781	-2.458946
C	-4.702855	0.840538	-0.540412
H	-3.955177	1.600671	-0.799575
H	-4.956437	1.004817	0.515780
C	-2.601459	-1.183557	1.857699
H	-1.592615	-0.972099	2.246644
C	-3.564583	-0.234159	2.577406
H	-3.302174	0.803883	2.340944
H	-4.586021	-0.397529	2.203161
C	-3.557162	-0.469165	4.082539
H	-4.234341	0.237465	4.575712
H	-2.550136	-0.260630	4.476226
C	-3.937962	-1.906531	4.407054
H	-3.936987	-2.079205	5.489164
H	-4.966697	-2.093116	4.063890
C	-2.987531	-2.871472	3.714422
H	-3.269779	-3.911412	3.914686
H	-1.981166	-2.740622	4.137031
C	-2.921655	-2.645857	2.203898
H	-3.879237	-2.937793	1.749344
H	-2.162243	-3.316335	1.784439
Al	2.400683	0.749054	-0.952702
N	3.238514	-0.337109	0.416916
N	2.022096	2.209564	0.290791
C	4.229261	0.340388	1.028199
C	4.219120	1.735525	1.160161
H	5.107203	2.185329	1.590750
C	3.133850	2.597220	0.944577
C	5.398263	-0.404090	1.602026
H	5.083029	-1.189758	2.294327
H	6.080285	0.269998	2.121289
H	5.947230	-0.907732	0.798177
C	3.293081	3.996185	1.469620
H	3.516013	3.997302	2.539812
H	2.420486	4.625615	1.292314
H	4.154162	4.453169	0.970452
C	3.210883	-1.758521	0.608128
C	2.542260	-2.273344	1.742106
C	2.615977	-3.646884	1.985016
H	2.113514	-4.062684	2.853792
C	3.320424	-4.495050	1.137605
C	3.929825	-3.978581	0.004729
H	4.453809	-4.643508	-0.679011
C	3.882928	-2.611066	-0.286083
C	0.811209	2.945257	0.473916
C	0.170572	3.526717	-0.654633
C	-1.015976	4.248399	-0.448074
H	-1.523806	4.674009	-1.308407
C	-1.492900	4.509094	0.830569
C	-0.828851	3.980581	1.925684
H	-1.221283	4.152244	2.925647
C	0.281975	3.148706	1.777025
C	-0.322960	0.510555	-2.134801
C	-1.094892	0.487276	-3.289668
C	1.059828	0.255472	-2.297799
C	-0.625970	0.207634	-4.563793
C	1.566176	-0.000274	-3.591388
C	0.735825	-0.050239	-4.708447
H	-1.306550	0.205901	-5.411030
H	2.637096	-0.138840	-3.732755
H	1.143701	-0.265724	-5.693141
H	-1.985648	1.507125	1.044566
F	3.750710	1.287555	-1.814084
F	-2.442947	0.762639	-3.180554
H	-2.386261	5.111515	0.966516
H	3.372505	-5.558945	1.351870
C	0.864362	3.596325	-2.001446
C	1.954692	4.669514	-1.960757
C	-0.084465	3.834455	-3.168341
H	1.366419	2.644954	-2.195947
H	2.716053	4.444252	-1.208296
H	2.461253	4.732843	-2.928474
H	1.529645	5.654571	-1.734343
H	-0.946454	3.161523	-3.133716
H	-0.454720	4.866230	-3.187510
H	0.437253	3.659050	-4.113275
C	0.809445	2.453523	3.018856
C	-0.230670	1.448836	3.518926
C	1.152297	3.429528	4.144977
H	1.715063	1.890493	2.761832
H	-0.467868	0.710400	2.746496
H	0.122791	0.930573	4.418363
H	-1.168982	1.956760	3.773100
H	1.811056	4.238022	3.817825
H	0.247830	3.894006	4.552840
H	1.643209	2.903821	4.970709
C	4.514242	-2.113469	-1.569985
C	3.706667	-2.632316	-2.760754
C	5.979370	-2.524424	-1.700675

H	4.474447	-1.016865	-1.583963
H	2.661003	-2.314474	-2.699806
H	4.115018	-2.261394	-3.706613
H	3.721141	-3.728767	-2.794234
H	6.581721	-2.187554	-0.850755
H	6.087077	-3.613027	-1.765765
H	6.416833	-2.098828	-2.608558
C	1.767061	-1.367589	2.685164
C	2.666137	-0.684378	3.722486
C	0.656695	-2.103434	3.430545
H	1.306873	-0.577945	2.068008
H	3.347528	0.048167	3.284219
H	2.056511	-0.154036	4.462717
H	3.263672	-1.428241	4.263151
H	0.028820	-2.706317	2.765125
H	1.060536	-2.774291	4.197560
H	0.011555	-1.384645	3.946988

Int-9_AlPdAl _M06L.log

SCF (M06L) = -3041.33211018
E(SCF)+ZPE(0 K) = -3039.971195
H(298 K) = -3039.889881
G(298 K) = -3040.086082
Lowest Frequency = 11.3647 cm^{-1}

Pd	-0.517196000	-0.495850000	-0.842168000
Al	2.614626000	0.054222000	-0.021471000
N	3.576039000	1.705641000	-0.117299000
N	3.432696000	-0.543402000	1.615645000
C	4.101873000	2.300264000	0.960695000
C	4.270704000	1.643434000	2.186962000
H	4.717425000	2.227758000	2.983059000
C	4.011555000	0.297496000	2.482022000
C	4.540928000	3.731186000	0.873462000
H	3.664821000	4.387834000	0.830164000
H	5.112732000	3.924267000	-0.038315000
H	5.138711000	4.016428000	1.739953000
C	4.419306000	-0.197911000	3.837938000
H	5.112421000	0.494645000	4.317056000
H	4.880708000	-1.187659000	3.782053000
H	3.543002000	-0.301153000	4.486633000
C	3.547722000	2.411543000	-1.368177000
C	4.548271000	2.109758000	-2.314565000
C	4.516016000	2.777982000	-3.539056000
H	5.276416000	2.562937000	-4.284971000
C	3.518325000	3.704238000	-3.824079000
C	2.526897000	3.965237000	-2.888852000
H	1.734044000	4.672753000	-3.123816000
C	2.514959000	3.324896000	-1.646253000
C	3.245158000	-1.919124000	1.982132000
C	2.188221000	-2.293602000	2.832824000
C	2.024607000	-3.652903000	3.116890000
H	1.203613000	-3.961639000	3.762213000
C	2.873224000	-4.609398000	2.578220000
C	3.906046000	-4.220730000	1.732176000
H	4.559101000	-4.975330000	1.302906000
C	4.110936000	-2.877500000	1.414714000
F	1.045365000	0.532423000	0.490139000
C	1.044073000	-1.440643000	-1.873944000
C	2.399137000	-1.130519000	-1.561888000
C	0.843014000	-2.351045000	-2.912365000
C	3.432964000	-1.707463000	-2.326808000
C	1.856558000	-2.927013000	-3.664757000
C	3.174447000	-2.589447000	-3.369733000
H	4.474256000	-1.474639000	-2.111241000
H	1.606743000	-3.620841000	-4.462999000
H	3.991712000	-3.018433000	-3.946372000
F	-0.423168000	-2.730538000	-3.245108000
H	-1.496077000	-1.258873000	-1.745154000
H	2.725507000	-5.660884000	2.808336000
H	3.508260000	4.212143000	-4.784562000
C	5.634757000	1.099688000	-1.996290000
C	6.780634000	1.724948000	-1.198244000
C	6.175576000	0.389183000	-3.231117000
H	5.181451000	0.334657000	-1.348764000
H	6.456170000	2.072711000	-0.213169000
H	7.581884000	0.995584000	-1.038540000
H	7.210507000	2.578982000	-1.734641000
H	5.369546000	-0.012677000	-3.852112000
H	6.785225000	1.055837000	-3.851371000
H	6.819257000	-0.445128000	-2.931275000
C	1.384914000	3.594840000	-0.672422000
C	0.065655000	3.027963000	-1.197731000
C	1.222946000	5.082812000	-0.364232000
H	1.608496000	3.073373000	0.265805000
H	0.148745000	1.968682000	-1.470686000
H	-0.719964000	3.112452000	-0.437110000
H	-0.267018000	3.578382000	-2.087083000
H	2.156568000	5.551047000	-0.033928000
H	0.877883000	5.635864000	-1.245802000
H	0.471421000	5.226238000	0.419262000
C	5.243189000	-2.445072000	0.503663000
C	5.672425000	-3.522729000	-0.482641000
C	6.448205000	-1.928197000	1.291098000
H	4.867060000	-1.599565000	-0.087373000
H	4.818397000	-3.927299000	-1.033449000
H	6.372296000	-3.103362000	-1.213518000
H	6.188386000	-4.351521000	0.015498000
H	6.206743000	-1.036771000	1.877948000
H	6.828741000	-2.693498000	1.977630000
H	7.262144000	-1.658615000	0.608919000
C	1.199873000	-1.293857000	3.402576000
C	1.060127000	-1.410569000	4.919997000
C	-0.164827000	-1.467865000	2.740650000
H	1.550436000	-0.282750000	3.161747000
H	2.020643000	-1.340606000	5.440850000
H	0.407425000	-0.620859000	5.307779000
H	0.609592000	-2.368025000	5.204153000
H	-0.095297000	-1.385658000	1.649062000
H	-0.588846000	-2.451303000	2.978961000
H	-0.872675000	-0.705483000	3.091970000
Al	-2.659226000	0.048583000	0.157497000
N	-4.066194000	-1.300516000	0.320037000
C	-5.101109000	-1.266558000	1.168012000
C	-5.351873000	-0.182134000	2.023150000
C	-4.735407000	1.072413000	1.993171000
N	-3.685229000	1.342092000	1.201042000
C	-5.303505000	2.155425000	2.861587000
H	-5.557264000	3.040886000	2.269490000
H	-4.568136000	2.486914000	3.602503000
H	-6.195762000	1.814853000	3.388171000
H	-6.187818000	-0.291553000	2.704692000
C	-6.091084000	-2.393960000	1.182540000
H	-5.602043000	-3.366801000	1.086393000
H	-6.772217000	-2.307687000	0.327898000
H	-6.693056000	-2.375332000	2.092513000
C	-4.019061000	-2.361614000	-0.654268000
C	-4.849419000	-2.279382000	-1.788222000
C	-4.782240000	-3.304785000	-2.733659000
C	-3.913376000	-4.374469000	-2.570845000
C	-3.073553000	-4.417539000	-1.466085000
C	-0.806742000	-4.099887000	0.335505000
C	-3.097007000	-3.414482000	-0.495618000
C	-2.148268000	-3.465030000	0.683169000
H	-2.365140000	-5.234441000	-1.363691000
H	-3.872300000	-5.162546000	-3.317403000
H	-5.409888000	-3.249624000	-3.620633000
H	-1.937960000	-2.423868000	0.963040000
H	-0.389712000	-3.659403000	-0.575603000
H	-0.086548000	-3.934902000	1.145130000
H	-0.889275000	-5.184337000	0.196059000
C	-2.786685000	-4.147670000	1.892474000
H	-3.666745000	-3.605634000	2.255478000
H	-3.099555000	-5.169455000	1.646359000
H	-2.073834000	-4.212861000	2.722967000
C	-5.049022000	-0.206650000	-3.141300000
H	-5.625455000	0.706341000	-3.331138000
H	-4.029565000	0.079318000	-2.855117000

H	-4.972315000	-0.756743000	-4.085597000
C	-5.718433000	-1.068110000	-2.068527000
H	-5.788666000	-0.462669000	-1.155034000
C	-7.140816000	-1.431828000	-2.485460000
H	-7.750540000	-0.529740000	-2.601536000
H	-7.158361000	-1.958394000	-3.445577000
H	-7.633378000	-2.077897000	-1.751210000
C	-3.240173000	2.703355000	1.078292000
C	-2.387326000	3.273572000	2.041204000
C	-1.990249000	4.602481000	1.868225000
C	-2.410063000	5.343976000	0.772975000
C	-3.211141000	4.750383000	-0.194659000
C	-3.629143000	3.423779000	-0.072672000
H	-3.503298000	5.323369000	-1.069837000
C	-4.471882000	2.775626000	-1.156091000
C	-5.959954000	2.777485000	-0.804198000
H	-6.167157000	2.222432000	0.115958000
H	-6.547756000	2.314970000	-1.605564000
H	-6.326195000	3.802067000	-0.670816000
H	-4.162299000	1.719925000	-1.222481000
C	-4.241966000	3.382854000	-2.533831000
H	-3.177996000	3.411395000	-2.787760000
H	-4.753614000	2.790778000	-3.297842000
H	-4.636420000	4.402449000	-2.603643000
H	-2.091286000	6.376570000	0.658000000
H	-1.327103000	5.055130000	2.602910000
C	-0.354636000	2.139483000	2.909246000
C	-1.819091000	2.476425000	3.197926000
H	-2.371668000	1.529895000	3.266483000
H	-0.239712000	1.566694000	1.983505000
H	0.083118000	1.555741000	3.728057000
H	0.239330000	3.056923000	2.803926000
H	-1.344402000	4.105191000	4.569195000
C	-1.953540000	3.195370000	4.538213000
H	-1.610084000	2.551642000	5.354090000
H	-2.986588000	3.488906000	4.753360000

Int-9'_AlPdPCy_3 _M06L.log

SCF (M06L) = -2847.16682371
E(SCF)+ZPE(0 K) = -2845.959105
H(298 K) = -2845.893254
G(298 K) = -2846.057699
Lowest Frequency = 16.7658 cm^{-1}

Pd	1.241897	-0.009592	-1.303473
Al	-1.718336	0.058596	0.047479
N	-2.708758	-1.235968	1.029987
N	-2.359562	1.577707	1.033538
C	-2.914596	-1.055917	2.345154
C	-2.859247	0.204944	2.950753
H	-3.059389	0.232931	4.015856
C	-2.716705	1.452496	2.316453
C	-3.222368	-2.242969	3.207535
H	-4.033738	-2.848518	2.794519
H	-3.481610	-1.944306	4.223872
H	-2.343997	-2.898644	3.250681
C	-3.000120	2.676259	3.134677
H	-3.549760	2.423887	4.042145
H	-3.570869	3.412081	2.561152
H	-2.067795	3.168515	3.429888
C	-3.112375	-2.476019	0.426375
C	-4.432010	-2.573572	-0.062990
C	-4.836016	-3.784769	-0.626781
H	-5.850145	-3.883866	-1.005163
C	-3.959857	-4.859631	-0.727245
C	-2.652015	-4.726367	-0.284390
H	-1.956467	-5.555882	-0.394892
C	-2.201268	-3.536901	0.294756
C	-2.284374	2.879253	0.428618
C	-1.191069	3.726989	0.686779
C	-1.155204	4.966800	0.040945
H	-0.313400	5.631067	0.225358
C	-2.156999	5.355800	-0.835057
C	-3.216400	4.494885	-1.097118
H	-3.988491	4.793401	-1.800392
C	-3.297836	3.245471	-0.482673
F	-0.092663	-0.053296	0.600264
C	-0.430766	-0.006704	-2.515287
C	-1.708419	-0.055222	-1.891281
C	-0.424007	-0.070565	-3.908895
C	-2.868041	-0.202716	-2.676226
C	-1.565284	-0.193581	-4.690838
C	-2.805984	-0.269581	-4.063459
H	-3.844900	-0.270981	-2.194630
H	-1.467748	-0.239989	-5.771960
H	-3.711073	-0.381500	-4.656666
F	0.753804	-0.023513	-4.585934
P	3.206808	-0.107721	0.011807
C	2.715699	-0.117402	1.807102
C	2.084081	-1.460588	2.187024
C	3.695848	0.350466	2.884545
H	1.887898	0.608131	1.802892
C	1.359829	-1.352144	3.523641
H	2.864321	-2.234740	2.257625
H	1.393452	-1.775638	1.397796
C	2.970900	0.457142	4.225750
H	4.536840	-0.352330	2.974858
H	4.132239	1.322860	2.621339
C	2.302667	-0.857569	4.616155
H	0.921314	-2.319325	3.801438
H	0.518409	-0.650630	3.411401
H	3.663719	0.784633	5.009511
H	2.201708	1.241423	4.144555
H	1.769663	-0.746430	5.567372
H	3.080944	-1.616368	4.787260
C	4.339566	1.361641	-0.159047
C	5.805495	1.184147	0.247865
C	4.248433	1.987476	-1.556355
H	3.890209	2.086685	0.540668
C	6.547112	2.517304	0.202274
H	6.288197	0.483845	-0.449981
H	5.888945	0.734826	1.243919
C	5.001844	3.311131	-1.612868
H	4.666758	1.286922	-2.295363
H	3.197469	2.120253	-1.837591
C	6.454240	3.155426	-1.178189
H	7.593965	2.377397	0.495780
H	6.105577	3.195850	0.948226
H	4.943113	3.736133	-2.621022
H	4.501697	4.030557	-0.946033
H	6.968765	4.123019	-1.190407
H	6.980647	2.516992	-1.903004
C	4.150944	-1.683199	-0.279918
C	4.629686	-1.778017	-1.733446
C	5.268369	-2.099678	0.683026
H	3.343213	-2.428148	-0.168025
C	5.126463	-3.183901	-2.052271
H	5.447067	-1.056788	-1.892414
H	3.819775	-1.487531	-2.413551
C	5.735804	-3.518238	0.365701
H	6.120683	-1.416082	0.590116
H	4.932981	-2.036378	1.724486
C	6.209955	-3.632504	-1.078483
H	5.490387	-3.231751	-3.084658
H	4.275381	-3.878536	-1.993426
H	6.530330	-3.818666	1.058582
H	4.900516	-4.215711	0.531310
H	6.526952	-4.658013	-1.299564
H	7.099827	-2.999353	-1.212703
H	2.034704	0.035231	-2.610502
H	-2.107273	6.323559	-1.326038
H	-4.293626	-5.793566	-1.170733
C	-5.388352	-1.396834	0.011676
C	-6.186085	-1.380563	1.317589
C	-6.340148	-1.330442	-1.179323
H	-4.776569	-0.483572	-0.000789
H	-5.546637	-1.235407	2.192900
H	-6.918678	-0.566223	1.312331
H	-6.734032	-2.320712	1.450777
H	-5.809396	-1.408973	-2.133546

H	-7.089911	-2.128646	-1.148368
H	-6.888606	-0.382218	-1.171850
C	-0.747391	-3.401955	0.695533
C	0.130790	-3.358294	-0.556697
C	-0.286174	-4.513161	1.635024
H	-0.628016	-2.445839	1.221269
H	-0.156909	-2.546853	-1.235001
H	1.185626	-3.206573	-0.294641
H	0.058554	-4.299891	-1.113848
H	-0.898327	-4.571114	2.541600
H	-0.329029	-5.494069	1.148687
H	0.753277	-4.348426	1.940050
C	-4.449979	2.304491	-0.769133
C	-5.075676	2.507763	-2.142248
C	-5.513737	2.358476	0.328237
H	-4.034036	1.288969	-0.762883
H	-4.319057	2.496316	-2.932208
H	-5.788010	1.702783	-2.351927
H	-5.629524	3.451097	-2.207141
H	-5.118577	2.047969	1.301375
H	-5.917833	3.371831	0.433986
H	-6.349477	1.690912	0.087683
C	-0.033123	3.342840	1.588476
C	0.236126	4.400424	2.660369
C	1.236308	3.115737	0.767612
H	-0.273287	2.394653	2.088193
H	-0.658786	4.656855	3.236292
H	1.001986	4.053271	3.361386
H	0.605993	5.329817	2.213864
H	1.122069	2.298553	0.041172
H	1.511037	4.019531	0.210671
H	2.074534	2.869690	1.431317

Int-S2_AlPdAl _M06L.log

SCF (M06L) = -3041.37161759
E(SCF)+ZPE(0 K) = -3040.006161
H(298 K) = -3039.925239
G(298 K) = -3040.118444
Lowest Frequency = 17.3238 cm^{-1}

Pd	-0.080206000	0.219843000	-0.199541000
Al	3.306061000	0.054941000	-0.836828000
N	3.438689000	-1.765348000	-0.278813000
N	3.964056000	0.819370000	0.792682000
C	4.557597000	-2.093133000	0.388856000
C	5.356122000	-1.137887000	1.038427000
H	6.270743000	-1.507467000	1.488026000
C	5.009305000	0.186582000	1.350465000
C	4.965728000	-3.533842000	0.507940000
H	4.292610000	-4.080310000	1.175515000
H	5.978660000	-3.617338000	0.904252000
H	4.912619000	-4.044545000	-0.457462000
C	5.834239000	0.876314000	2.396236000
H	5.912234000	1.950839000	2.211739000
H	6.834438000	0.441615000	2.439899000
H	5.382129000	0.754930000	3.386312000
C	2.556454000	-2.837813000	-0.634534000
C	1.694597000	-3.349368000	0.355992000
C	0.996451000	-4.524901000	0.066524000
H	0.328691000	-4.945841000	0.813852000
C	1.110688000	-5.139344000	-1.174482000
C	1.863187000	-4.542143000	-2.178074000
H	1.885131000	-4.986694000	-3.169583000
C	2.590414000	-3.373008000	-1.936580000
C	3.516308000	2.051084000	1.372985000
C	3.718033000	3.259052000	0.667853000
C	3.296443000	4.449539000	1.263090000
H	3.448492000	5.387998000	0.738118000
C	2.687569000	4.456647000	2.512646000
C	2.466634000	3.258812000	3.174574000
H	1.956827000	3.259718000	4.136245000
C	2.865824000	2.038219000	2.623309000
C	1.593479000	0.678352000	-1.556204000
C	0.908611000	-0.122155000	-2.520801000
C	1.169628000	2.040879000	-1.489548000
C	0.032098000	0.456286000	-3.434183000
H	1.129465000	-1.181785000	-2.629411000
C	0.276213000	2.593164000	-2.410415000
H	1.610178000	2.701346000	-0.743748000
C	-0.274636000	1.808259000	-3.422810000
H	0.001163000	3.641854000	-2.335843000
H	-0.959468000	2.217475000	-4.159858000
F	4.552071000	0.239283000	-1.957744000
F	-0.547470000	-0.338861000	-4.368286000
H	0.565990000	-6.058503000	-1.377898000
H	2.368840000	5.394946000	2.959056000
C	4.389466000	3.267954000	-0.692866000
C	5.902761000	3.083169000	-0.581566000
C	4.065030000	4.503718000	-1.522547000
H	4.012459000	2.410325000	-1.264500000
H	6.165787000	2.134181000	-0.107940000
H	6.360907000	3.083849000	-1.574957000
H	6.351070000	3.896439000	0.001637000
H	2.986184000	4.678733000	-1.590249000
H	4.529244000	5.407624000	-1.111879000
H	4.448130000	4.378913000	-2.538895000
C	2.524074000	0.760638000	3.366573000
C	1.016715000	0.510727000	3.318040000
C	2.994054000	0.784888000	4.821290000
H	3.014834000	-0.082530000	2.863637000
H	0.636604000	0.416407000	2.287402000
H	0.759715000	-0.407659000	3.858821000
H	0.469430000	1.335662000	3.791287000
H	4.055279000	1.034760000	4.920269000
H	2.434253000	1.521844000	5.407763000
H	2.833076000	-0.190059000	5.292511000
C	3.374145000	-2.695948000	-3.047478000
C	2.726608000	-2.885908000	-4.415840000
C	4.842799000	-3.116179000	-3.082778000
H	3.374598000	-1.617076000	-2.846271000
H	1.660102000	-2.633457000	-4.404316000
H	3.215295000	-2.242177000	-5.152593000
H	2.821756000	-3.916394000	-4.776082000
H	5.377506000	-2.796747000	-2.184485000
H	4.940189000	-4.204498000	-3.178481000
H	5.350049000	-2.655720000	-3.936031000
C	1.461205000	-2.605434000	1.661528000
C	2.438749000	-2.954575000	2.786811000
C	0.034971000	-2.799559000	2.159704000
H	1.583934000	-1.533432000	1.430501000
H	3.458284000	-2.606832000	2.598672000
H	2.108678000	-2.487296000	3.722208000
H	2.469922000	-4.037824000	2.959330000
H	-0.696776000	-2.613456000	1.366645000
H	-0.129514000	-3.810214000	2.552185000
H	-0.174366000	-2.099946000	2.972602000
Al	-2.397852000	0.204314000	0.342266000
N	-3.676636000	1.653499000	-0.068400000
N	-3.881032000	-1.058454000	0.647753000
C	-5.196674000	-0.801666000	0.614741000
C	-5.710534000	0.492383000	0.466869000
C	-4.993722000	1.660004000	0.175165000
H	-6.785090000	0.598228000	0.566882000
C	-6.182650000	-1.924864000	0.765233000
H	-5.924751000	-2.760020000	0.106712000
H	-7.195454000	-1.590576000	0.536497000
H	-6.178247000	-2.330921000	1.782422000
H	-5.398948000	3.597157000	0.969487000
H	-6.823958000	2.790550000	0.287832000
C	-5.753861000	2.956013000	0.154112000
H	-5.595644000	3.522401000	-0.766184000
C	-3.462660000	-2.405611000	0.919644000
C	-3.529238000	-2.889197000	2.244795000
C	-3.124338000	-4.201673000	2.488373000
C	-2.649796000	-5.011511000	1.461477000
C	-2.548716000	-4.504488000	0.174298000
C	-2.940851000	-3.195155000	-0.122369000
H	-2.148214000	-5.126852000	-0.623468000

H	-2.341647000	-6.032651000	1.672202000
H	-3.170379000	-4.593999000	3.501157000
C	-3.952379000	-1.985532000	3.389385000
H	-4.650363000	-1.236062000	2.997214000
C	-2.742102000	-1.222076000	3.931862000
H	-2.211763000	-0.679498000	3.137590000
H	-3.042543000	-0.499953000	4.698734000
H	-2.022541000	-1.914951000	4.383630000
H	-5.506924000	-3.322856000	4.144861000
C	-4.668428000	-2.723741000	4.515000000
H	-5.059351000	-2.012552000	5.248663000
H	-3.994585000	-3.397577000	5.054769000
H	-2.908448000	-1.587535000	-1.503845000
C	-2.815513000	-2.677645000	-1.539989000
C	-3.950889000	-3.197474000	-2.420108000
H	-3.944543000	-4.292878000	-2.464210000
H	-4.931939000	-2.886020000	-2.043292000
C	-1.450247000	-2.971900000	-2.151089000
H	-1.340695000	-2.442299000	-3.103551000
H	-0.643783000	-2.635956000	-1.485991000
H	-1.303099000	-4.040313000	-2.345220000
H	-3.853056000	-2.822148000	-3.444029000
C	-3.113372000	2.837864000	-0.654966000
C	-2.431409000	3.772360000	0.146415000
C	-1.966971000	4.949482000	-0.447887000
C	-2.166202000	5.202288000	-1.797207000
C	-2.790267000	4.241827000	-2.583252000
C	-3.256619000	3.038854000	-2.045744000
C	-3.884272000	2.053213000	-3.025109000
H	-2.908698000	4.410377000	-3.652951000
H	-1.813126000	6.129024000	-2.241348000
H	-1.447658000	5.680596000	0.168718000
C	-2.188949000	3.562207000	1.627377000
H	-2.627606000	2.596733000	1.915270000
C	-2.862833000	4.646283000	2.468128000
H	-3.939625000	4.706870000	2.278058000
H	-2.720470000	4.454342000	3.536379000
H	-2.438984000	5.633867000	2.253853000
H	-0.515997000	3.356879000	2.998838000
C	-0.692927000	3.499412000	1.926651000
H	-0.211496000	2.670555000	1.389532000
H	-0.187032000	4.426770000	1.633100000
H	-3.449122000	2.336220000	-3.994432000
H	-5.683494000	3.271508000	-3.306790000
C	-5.398239000	2.222545000	-3.174961000
H	-5.756240000	1.665123000	-4.046753000
H	-5.932304000	1.827128000	-2.304078000
H	-3.656234000	0.028075000	-3.755764000
C	-3.542013000	0.579534000	-2.816023000
H	-4.209350000	0.108822000	-2.083972000
H	-2.505510000	0.443424000	-2.482366000

TS-1_ AlPdAl _M06L.log

SCF (M06L) = -3041.25111915

E(SCF)+ZPE(0 K) = -3039.887444

H(298 K) = -3039.806845

G(298 K) = -3039.999451

Lowest Frequency = -36.5771 cm^{-1}

Pd	0.065202000	-0.231710000	-1.000312000
Al	-2.170213000	-0.044380000	-0.083897000
N	-3.339005000	1.553851000	-0.302491000
N	-3.643290000	-1.107568000	0.659323000
C	-4.631951000	1.638883000	0.027712000
C	-5.402071000	0.521884000	0.386326000
H	-6.461626000	0.699035000	0.533802000
C	-4.938066000	-0.747329000	0.734390000
C	-5.310271000	2.977208000	0.117645000
H	-5.036317000	3.456176000	1.065310000
H	-6.396059000	2.866816000	0.103626000
H	-5.006969000	3.664410000	-0.673781000
C	-5.948441000	-1.708380000	1.295202000
H	-5.788579000	-2.724539000	0.924602000
H	-6.963514000	-1.390921000	1.050892000
H	-5.866547000	-1.763537000	2.386821000
C	-2.646123000	2.795387000	-0.544308000
C	-2.056620000	3.468272000	0.541784000
C	-1.431699000	4.696593000	0.305454000
H	-0.961048000	5.218147000	1.137287000
C	-1.399352000	5.250203000	-0.964244000
C	-1.955538000	4.556162000	-2.032841000
H	-1.895156000	4.981063000	-3.030669000
C	-2.574999000	3.316243000	-1.856396000
C	-3.276746000	-2.329032000	1.328110000
C	-3.005254000	-3.492229000	0.578967000
C	-2.710519000	-4.667190000	1.277993000
H	-2.506734000	-5.578936000	0.724472000
C	-2.666033000	-4.693025000	2.666474000
C	-2.888466000	-3.526614000	3.385329000
H	-2.819631000	-3.542626000	4.470264000
C	-3.193328000	-2.328239000	2.736479000
C	-0.951111000	-0.700416000	-2.804025000
C	-0.297776000	0.469766000	-3.298479000
C	-0.454691000	-1.971977000	-3.206327000
C	0.748506000	0.317945000	-4.206311000
H	-0.684785000	1.457741000	-3.080512000
C	0.602545000	-2.054387000	-4.098158000
H	-0.932035000	-2.868761000	-2.827841000
C	1.204764000	-0.913397000	-4.644391000
H	0.965612000	-3.036712000	-4.389629000
H	2.023554000	-0.973259000	-5.352176000
F	-2.385837000	-0.641040000	-2.647014000
F	1.338299000	1.441714000	-4.680564000
H	-0.918143000	6.210980000	-1.130547000
H	-2.437421000	-5.619542000	3.186536000
C	-3.027887000	-3.467640000	-0.937933000
C	-4.438662000	-3.356899000	-1.517752000
C	-2.314705000	-4.659410000	-1.563445000
H	-2.483184000	-2.560964000	-1.241399000
H	-4.942477000	-2.434293000	-1.221818000
H	-4.394756000	-3.362267000	-2.611720000
H	-5.060158000	-4.204580000	-1.204115000
H	-1.288251000	-4.758830000	-1.195493000
H	-2.843287000	-5.599620000	-1.367215000
H	-2.274239000	-4.544289000	-2.651079000
C	-3.366314000	-1.052325000	3.541510000
C	-2.007641000	-0.383368000	3.758203000
C	-4.074528000	-1.269313000	4.875192000
H	-3.980501000	-0.353755000	2.959543000
H	-1.488846000	-0.188786000	2.808901000
H	-2.119515000	0.571121000	4.286040000
H	-1.352438000	-1.026287000	4.358139000
H	-5.022815000	-1.803509000	4.754656000
H	-3.460279000	-1.843780000	5.576707000
H	-4.287295000	-0.308576000	5.353639000
C	-3.141683000	2.563382000	-3.049368000
C	-2.492615000	2.969774000	-4.371820000
C	-4.660916000	2.698058000	-3.190391000
H	-2.932538000	1.500625000	-2.881396000
H	-1.399797000	3.013625000	-4.317780000
H	-2.756395000	2.249580000	-5.151761000
H	-2.844597000	3.952375000	-4.707458000
H	-5.205727000	2.192751000	-2.390041000
H	-4.965800000	3.751705000	-3.202635000
H	-4.989335000	2.249761000	-4.133566000
C	-2.048199000	2.911214000	1.952131000
C	-2.698199000	3.868474000	2.950761000
C	-0.619363000	2.586927000	2.381903000
H	-2.628566000	1.977368000	1.966893000
H	-3.719729000	4.139525000	2.664164000
H	-2.737587000	3.417473000	3.947982000
H	-2.126032000	4.798985000	3.036994000
H	-0.160642000	1.835363000	1.724371000
H	0.009810000	3.481888000	2.351286000
H	-0.601559000	2.204080000	3.408097000
Al	2.259350000	-0.004683000	0.117450000
N	3.514375000	-1.540527000	0.168886000
N	3.618806000	1.144686000	0.945015000

C	4.920999000	0.866299000	1.136003000
C	5.450974000	-0.420854000	1.010355000
C	4.755481000	-1.586175000	0.649172000
H	6.496609000	-0.540613000	1.272395000
C	5.860973000	1.964642000	1.546051000
H	5.704307000	2.859105000	0.935568000
H	6.899852000	1.645159000	1.451346000
H	5.693866000	2.273266000	2.583775000
H	5.101042000	-3.326222000	1.820239000
H	6.523536000	-2.788517000	0.922847000
C	5.439508000	-2.904828000	0.865591000
H	5.195279000	-3.638306000	0.094939000
C	3.141366000	2.428921000	1.371295000
C	2.997223000	2.663676000	2.759659000
C	2.572478000	3.924496000	3.175063000
C	2.256115000	4.917583000	2.250946000
C	2.338739000	4.646749000	0.893934000
C	2.782018000	3.404155000	0.424480000
H	2.063150000	5.412793000	0.170871000
H	1.925032000	5.894445000	2.594482000
H	2.469668000	4.131251000	4.236993000
C	3.235516000	1.546767000	3.762716000
H	4.066515000	0.928389000	3.401534000
C	2.009656000	0.632804000	3.847229000
H	1.683424000	0.277879000	2.859808000
H	2.215010000	-0.246107000	4.469008000
H	1.160338000	1.166811000	4.287988000
H	4.468557000	2.733849000	5.119819000
C	3.619942000	2.042648000	5.151513000
H	3.894598000	1.200301000	5.793160000
H	2.789229000	2.559526000	5.644192000
H	3.186989000	2.135819000	-1.242627000
C	2.868859000	3.172926000	-1.071254000
C	3.916168000	4.079662000	-1.715756000
H	3.657995000	5.136834000	-1.583656000
H	4.910818000	3.927557000	-1.283482000
C	1.505238000	3.350087000	-1.736500000
H	1.551510000	3.066597000	-2.792781000
H	0.741788000	2.724831000	-1.253737000
H	1.167812000	4.391082000	-1.681408000
H	3.983420000	3.888367000	-2.791306000
C	2.894118000	-2.730453000	-0.333705000
C	2.039604000	-3.470115000	0.506626000
C	1.361997000	-4.566676000	-0.031879000
C	1.547430000	-4.941444000	-1.355795000
C	2.397168000	-4.199084000	-2.165336000
C	3.065908000	-3.065936000	-1.692137000
C	3.948008000	-2.317602000	-2.685752000
H	2.538277000	-4.484644000	-3.206629000
H	1.022866000	-5.803555000	-1.760162000
H	0.681826000	-5.132570000	0.604066000
C	1.850842000	-3.126199000	1.971261000
H	2.513934000	-2.283487000	2.211970000
C	2.251374000	-4.297005000	2.869515000
H	3.267889000	-4.648143000	2.662330000
H	2.198822000	-4.010342000	3.925090000
H	1.576715000	-5.149441000	2.730052000
H	0.292807000	-2.460427000	3.330198000
C	0.415913000	-2.695147000	2.266177000
H	0.128749000	-1.814486000	1.676607000
H	-0.292794000	-3.494346000	2.022740000
H	3.561265000	-2.628081000	-3.667825000
H	5.498957000	-3.863700000	-2.621191000
C	5.407957000	-2.772562000	-2.623553000
H	5.964807000	-2.388654000	-3.484780000
H	5.904939000	-2.387655000	-1.726334000
H	4.263076000	-0.399550000	-3.624216000
C	3.879112000	-0.790281000	-2.675313000
H	4.493882000	-0.353795000	-1.879635000
H	2.848779000	-0.429683000	-2.565300000

TS-1’_AlPdPCy$_3$ _M06L.log

SCF (M06L) = -2847.08307615
E(SCF)+ZPE(0 K) = -2845.874483
H(298 K) = -2845.808772
G(298 K) = -2845.972447
Lowest Frequency = -108.8960 cm^{-1}

Pd	-0.405888	-0.479345	-0.566668
Al	1.698701	0.566223	-0.091295
N	3.481368	0.051458	0.566039
N	2.222749	2.415398	-0.370829
C	4.545779	0.849828	0.667938
C	4.538228	2.189632	0.241723
H	5.474852	2.725931	0.352270
C	3.444775	2.941600	-0.192780
C	5.826628	0.359547	1.279755
H	6.239606	1.118616	1.948728
H	6.576110	0.180338	0.501922
H	5.694173	-0.570186	1.835395
C	3.650116	4.415783	-0.390069
H	3.097957	4.787947	-1.257107
H	4.709024	4.653012	-0.506443
H	3.276503	4.973269	0.476747
C	3.514688	-1.316453	0.999541
C	2.710699	-1.680284	2.105182
C	2.611858	-3.032187	2.440329
H	1.999806	-3.320476	3.291938
C	3.292071	-4.006751	1.722204
C	4.098878	-3.631098	0.657784
H	4.636923	-4.392098	0.096365
C	4.223896	-2.294147	0.267151
C	1.092339	3.290896	-0.511972
C	0.418772	3.363790	-1.749475
C	-0.736372	4.144903	-1.822144
H	-1.269995	4.220856	-2.765114
C	-1.234568	4.806819	-0.705815
C	-0.573291	4.702619	0.509674
H	-0.978886	5.199544	1.389047
C	0.599385	3.951986	0.631319
C	0.883172	-1.349982	-1.932685
C	1.091082	-2.694988	-1.507153
C	-0.102478	-1.097361	-2.938649
C	0.261634	-3.678381	-2.005135
H	1.841962	-2.935959	-0.762768
C	-0.893275	-2.147861	-3.418997
H	-0.171200	-0.108562	-3.380576
C	-0.733953	-3.451568	-2.959551
H	-1.644522	-1.930754	-4.175233
H	-1.342225	-4.275080	-3.314996
F	2.167723	-0.549806	-2.072092
F	0.412631	-4.937553	-1.532615
C	1.246780	3.799094	1.996825
H	2.246507	3.368109	1.867260
C	0.444499	2.820072	2.854450
C	1.416077	5.133162	2.719659
H	1.963618	5.862986	2.113831
H	0.449343	5.581338	2.972965
H	1.962133	4.996531	3.658177
H	-0.544495	3.231607	3.084979
H	0.952405	2.616378	3.804705
H	0.287368	1.865849	2.333071
C	0.932752	2.603523	-2.957881
H	1.179647	1.587658	-2.612426
C	2.217504	3.212057	-3.522180
C	-0.097669	2.463553	-4.071545
H	-1.044584	2.048965	-3.709056
H	-0.316605	3.426189	-4.548429
H	0.284297	1.799645	-4.853201
H	2.059111	4.257617	-3.812403
H	2.540745	2.664630	-4.413242
H	3.040125	3.178437	-2.804460
C	5.111502	-1.980789	-0.924352
H	5.095146	-0.898181	-1.098565
C	4.612384	-2.657184	-2.201644
C	6.556388	-2.411176	-0.656204
H	6.949448	-2.013700	0.283879
H	6.630484	-3.502949	-0.600440
H	7.215183	-2.084138	-1.467234

H	4.596748	-3.747048	-2.087415
H	5.279550	-2.425860	-3.038478
H	3.606147	-2.330747	-2.467525
C	2.047564	-0.653969	3.007703
H	2.074387	0.321633	2.502876
C	2.849528	-0.505157	4.301778
C	0.582095	-0.966097	3.291690
H	0.014139	-0.998804	2.352320
H	0.455294	-1.922968	3.810521
H	0.143060	-0.189455	3.929362
H	2.863955	-1.445277	4.864942
H	2.410805	0.263353	4.947246
H	3.889751	-0.226038	4.101996
H	3.199312	-5.053797	1.995847
H	-2.147168	5.392405	-0.784526
P	-2.653724	-0.543939	0.174934
C	-3.795804	0.273496	-1.053040
H	-3.821477	-0.470447	-1.870465
H	-5.734627	-0.318324	-0.244353
C	-5.242615	0.573057	-0.648888
H	-5.244823	1.318787	0.159789
C	-6.034119	1.128176	-1.831231
H	-6.093077	0.357109	-2.614284
H	-7.066759	1.339405	-1.529870
C	-5.375863	2.377951	-2.403948
H	-5.944241	2.753836	-3.262409
H	-5.399489	3.174287	-1.644757
H	-3.901640	1.387625	-3.625426
C	-3.926854	2.106627	-2.791552
H	-3.447741	3.022290	-3.161970
C	-3.134810	1.537069	-1.622069
H	-2.099131	1.310189	-1.912707
H	-3.051469	2.296292	-0.829848
H	-2.577291	-2.623620	-0.900374
C	-3.065358	-2.357208	0.055168
H	-1.263014	-2.831826	1.157494
C	-2.319865	-3.138118	1.145501
H	-2.731455	-2.879735	2.134829
C	-2.434316	-4.642765	0.927597
H	-1.901955	-4.909031	0.003493
H	-1.929371	-5.180683	1.738194
H	-3.958330	-6.157898	0.643166
C	-3.890698	-5.077925	0.816927
H	-4.132929	-4.556227	-1.254994
C	-4.605050	-4.313183	-0.290901
H	-5.651770	-4.629161	-0.370376
H	-4.400908	-4.886341	1.773424
C	-4.524358	-2.804143	-0.070155
H	-5.090986	-2.538219	0.833126
H	-5.009140	-2.281261	-0.904161
H	-1.938906	1.832006	1.536225
C	-2.900206	1.547929	1.987465
H	-3.678544	2.049790	1.392475
C	-3.087228	0.028190	1.894204
H	-2.272319	-0.422921	2.486577
H	-4.493986	-1.500748	2.524077
C	-4.402836	-0.408751	2.545313
H	-2.156108	1.614445	4.004427
C	-2.997200	2.031480	3.429079
H	-2.887576	3.122963	3.470174
H	-5.145447	2.086054	3.549134
C	-4.309235	1.597937	4.072189
H	-4.359393	1.934171	5.114148
C	-4.482152	0.086380	3.988187
H	-3.687355	-0.398863	4.574890
H	-5.431341	-0.221151	4.442134
H	-5.257149	-0.015046	1.979001

TS-3_ 1,2,3-TriFB_AlPdAl _M06L.log

SCF (M06L) = -3140.46478082
E(SCF)+ZPE(0 K) = -3139.108711
H(298 K) = -3139.027435
G(298 K) = -3139.220874

Lowest Frequency = -35.2998 cm^{-1}

Pd	-0.044675	0.165161	-0.929147
Al	2.169910	0.355001	0.019086
N	3.702204	-0.891983	0.128636
N	3.203439	1.818837	0.826038
C	4.890868	-0.638179	0.684783
C	5.313465	0.658255	1.010792
H	6.343965	0.760922	1.332137
C	4.523667	1.803217	1.102950
C	5.812587	-1.763331	1.058745
H	5.466065	-2.208161	1.999494
H	6.829522	-1.400204	1.217680
H	5.826438	-2.567205	0.321554
C	5.198744	3.043956	1.619684
H	4.859663	3.938624	1.090611
H	6.282477	2.958696	1.524099
H	4.966892	3.207493	2.678041
C	3.378514	-2.273757	-0.128242
C	2.726294	-3.014342	0.873602
C	2.397890	-4.346809	0.606110
H	1.872806	-4.925008	1.365105
C	2.728534	-4.932002	-0.605771
C	3.376453	-4.183692	-1.583044
H	3.612134	-4.649236	-2.535034
C	3.706558	-2.841869	-1.379521
C	2.493776	2.984907	1.287401
C	2.096558	3.981633	0.375090
C	1.533158	5.154438	0.889977
H	1.232571	5.943889	0.207073
C	1.352910	5.331097	2.255882
C	1.692997	4.310633	3.135294
H	1.514376	4.438823	4.199603
C	2.257237	3.120329	2.673075
C	0.942111	0.474793	-2.762297
C	0.676094	-0.791139	-3.361744
C	0.089512	1.545545	-3.177301
C	-0.328305	-0.957093	-4.299139
C	-0.889139	1.353011	-4.150541
H	0.274002	2.537579	-2.780224
C	-1.103755	0.110335	-4.741810
H	-1.496465	2.201568	-4.452190
H	-1.865896	-0.056127	-5.494279
F	2.351724	0.787868	-2.622512
H	2.472012	-5.970516	-0.799403
H	0.922433	6.255104	2.633311
C	2.287190	3.793298	-1.117405
C	3.743335	3.946955	-1.558424
C	1.399543	4.707909	-1.951827
H	1.991114	2.757874	-1.341074
H	4.399956	3.205615	-1.096711
H	3.822794	3.817976	-2.642586
H	4.125290	4.945072	-1.311072
H	0.346938	4.634509	-1.656609
H	1.703923	5.758066	-1.870310
H	1.474289	4.437530	-3.009746
C	2.571095	1.987523	3.635985
C	1.418323	0.980994	3.666165
C	2.896628	2.459050	5.048854
H	3.454352	1.451979	3.264037
H	1.157733	0.613526	2.663242
H	1.671210	0.114462	4.288846
H	0.513960	1.441278	4.081150
H	3.678893	3.225209	5.057854
H	2.018162	2.877345	5.552104
H	3.240527	1.618696	5.658987
C	4.357724	-2.024919	-2.487572
C	4.216334	-2.665854	-3.866166
C	5.845185	-1.736762	-2.255585
H	3.832196	-1.061879	-2.511261
H	3.190849	-2.963186	-4.088087
H	4.534330	-1.955560	-4.634922
H	4.858918	-3.549327	-3.964141
H	6.030849	-1.043010	-1.433722
H	6.401456	-2.661260	-2.057792

H	6.277130	-1.284707	-3.154284
C	2.367093	-2.431885	2.227844
C	2.995287	-3.232775	3.369060
C	0.853313	-2.361836	2.408841
H	2.765010	-1.408231	2.288262
H	4.079994	-3.335921	3.259220
H	2.797240	-2.751913	4.332976
H	2.576057	-4.244005	3.418285
H	0.381764	-1.716898	1.654926
H	0.406509	-3.356439	2.321899
H	0.600112	-1.972384	3.401165
Al	-2.256070	-0.253465	0.146127
N	-3.794362	0.976559	-0.028495
N	-3.419794	-1.609267	0.972388
C	-4.760010	-1.577517	1.084170
C	-5.519575	-0.427740	0.845105
C	-5.049072	0.812960	0.393332
H	-6.580477	-0.496719	1.058957
C	-5.500176	-2.810529	1.520335
H	-5.155360	-3.687854	0.964712
H	-6.574395	-2.693216	1.370835
H	-5.325930	-3.035518	2.578004
H	-5.731663	2.629611	1.267929
H	-7.025833	1.657326	0.553885
C	-5.991687	1.980501	0.422470
H	-5.919402	2.598670	-0.474880
C	-2.750110	-2.774834	1.480655
C	-2.644915	-2.929405	2.883334
C	-2.065297	-4.097286	3.376430
C	-1.569960	-5.073601	2.515160
C	-1.617634	-4.870745	1.144654
C	-2.202987	-3.721786	0.598363
H	-1.197352	-5.615918	0.471266
H	-1.124549	-5.979324	2.918752
H	-1.989518	-4.244792	4.450325
C	-3.106977	-1.823400	3.817818
H	-4.013820	-1.372605	3.395294
C	-2.056427	-0.712060	3.894381
H	-1.740806	-0.366479	2.900390
H	-2.436914	0.154172	4.447450
H	-1.154890	-1.067947	4.405844
H	-4.186107	-3.131791	5.194038
C	-3.462112	-2.310706	5.217094
H	-3.894900	-1.495861	5.804767
H	-2.579272	-2.661972	5.762223
H	-2.713558	-2.597047	-1.138691
C	-2.221647	-3.551450	-0.906903
C	-3.031104	-4.650399	-1.592527
H	-2.588142	-5.637888	-1.419471
H	-4.064154	-4.684458	-1.230311
C	-0.801234	-3.481113	-1.464092
H	-0.816857	-3.284811	-2.540161
H	-0.219680	-2.680266	-0.986404
H	-0.261782	-4.422220	-1.304774
H	-3.058270	-4.488221	-2.674716
C	-3.419194	2.201904	-0.669695
C	-2.831368	3.228384	0.092741
C	-2.365137	4.365704	-0.570391
C	-2.516749	4.503108	-1.944109
C	-3.119349	3.486123	-2.673101
C	-3.556799	2.303137	-2.068543
C	-4.185399	1.248650	-2.973971
H	-3.241727	3.593041	-3.749962
H	-2.160580	5.398332	-2.447350
H	-1.880311	5.153808	0.003935
C	-2.688922	3.121142	1.598002
H	-3.296739	2.270459	1.936221
C	-3.208984	4.370023	2.308410
H	-4.231993	4.620187	2.008029
H	-3.198155	4.227332	3.393644
H	-2.580367	5.241788	2.093654
H	-1.136026	2.791517	3.082852
C	-1.238360	2.852982	1.992801
H	-0.852120	1.925328	1.550691
H	-0.591896	3.663205	1.642880
H	-3.830962	1.516258	-3.980334
H	-6.054514	2.378047	-3.148690
C	-5.712416	1.347802	-3.005890
H	-6.117040	0.741341	-3.822885
H	-6.153922	0.966623	-2.078518
H	-4.013485	-0.794950	-3.649025
C	-3.766298	-0.206384	-2.758540
H	-4.296702	-0.668991	-1.917230
H	-2.684787	-0.299763	-2.595325
F	1.449000	-1.847214	-3.038225
F	-0.532072	-2.194661	-4.799318

TS-3 _FB_AlPdAl _M06L.log

SCF (M06L) = -2942.02314822
E(SCF)+ZPE(0 K) = -2940.652368
H(298 K) = -2940.572308
G(298 K) = -2940.765653
Lowest Frequency = -43.0295 cm^{-1}

Pd	0.046383	-0.154368	-1.023910
Al	-2.175991	-0.061285	-0.049049
N	-3.433418	1.478955	-0.222332
N	-3.559382	-1.177485	0.793842
C	-4.687792	1.531390	0.233821
C	-5.387778	0.393059	0.663373
H	-6.435726	0.537613	0.902085
C	-4.857939	-0.862712	0.961690
C	-5.391761	2.849919	0.392725
H	-5.091828	3.300390	1.346761
H	-6.474681	2.714263	0.420874
H	-5.138752	3.569423	-0.387139
C	-5.796341	-1.865166	1.574130
H	-5.629350	-2.869265	1.174728
H	-6.835307	-1.578264	1.403324
H	-5.640605	-1.936371	2.656575
C	-2.799782	2.724094	-0.574730
C	-2.095048	3.436079	0.413702
C	-1.481748	4.640935	0.057070
H	-0.919943	5.190616	0.810820
C	-1.574120	5.136143	-1.233852
C	-2.260956	4.412991	-2.202907
H	-2.311205	4.799179	-3.216801
C	-2.877917	3.194623	-1.905790
C	-3.109698	-2.391479	1.423357
C	-2.851943	-3.538533	0.645266
C	-2.483041	-4.712470	1.310151
H	-2.289268	-5.612112	0.733245
C	-2.351983	-4.750853	2.692817
C	-2.560235	-3.597815	3.437982
H	-2.423872	-3.623649	4.516140
C	-2.936166	-2.401257	2.824086
C	-1.048333	-0.595069	-2.801030
C	-0.534582	0.622524	-3.330637
C	-0.471859	-1.820211	-3.226820
C	0.484574	0.589183	-4.286184
H	-0.995500	1.563033	-3.043275
C	0.541990	-1.814219	-4.176920
H	-0.851694	-2.753862	-2.823327
C	1.005863	-0.620834	-4.738886
H	0.967130	-2.764578	-4.491607
H	1.788733	-0.636780	-5.491609
F	-2.472322	-0.652650	-2.529697
H	-1.097188	6.077988	-1.494049
H	-2.065951	-5.675660	3.187062
C	-2.964273	-3.495716	-0.866937
C	-4.409050	-3.425568	-1.362840
C	-2.244358	-4.648298	-1.554287
H	-2.470422	-2.567201	-1.186967
H	-4.925889	-2.526756	-1.019317
H	-4.428587	-3.409740	-2.457415
H	-4.981898	-4.300121	-1.030759
H	-1.199301	-4.722173	-1.236542
H	-2.731901	-5.610595	-1.358033

H	-2.257689	-4.503555	-2.639204
C	-3.094972	-1.135482	3.649147
C	-1.766601	-0.378860	3.717341
C	-3.635048	-1.389938	5.052732
H	-3.812605	-0.478281	3.141916
H	-1.361329	-0.164633	2.718209
H	-1.884870	0.573833	4.247422
H	-1.011783	-0.969865	4.249707
H	-4.555036	-1.983650	5.038201
H	-2.910406	-1.920169	5.679976
H	-3.853089	-0.441954	5.553586
C	-3.595049	2.408440	-2.992963
C	-3.129956	2.774511	-4.400847
C	-5.120116	2.544108	-2.937982
H	-3.358077	1.351478	-2.825537
H	-2.040313	2.789397	-4.497235
H	-3.515301	2.045762	-5.119602
H	-3.504982	3.758392	-4.706876
H	-5.559021	2.055696	-2.065842
H	-5.424106	3.597976	-2.930926
H	-5.567470	2.079429	-3.822555
C	-1.968281	2.954086	1.846171
C	-2.537404	3.971566	2.835506
C	-0.512432	2.646308	2.186645
H	-2.547618	2.026018	1.958009
H	-3.574437	4.239974	2.608125
H	-2.506734	3.576385	3.856449
H	-1.952499	4.898296	2.827806
H	-0.102990	1.860084	1.537531
H	0.112622	3.536556	2.066762
H	-0.421334	2.317793	3.227998
Al	2.298706	0.054209	-0.029640
N	3.571017	-1.470931	-0.034956
N	3.680130	1.208375	0.768476
C	4.986954	0.933542	0.928674
C	5.519289	-0.350476	0.780606
C	4.823330	-1.514314	0.416468
H	6.570721	-0.467999	1.019255
C	5.932247	2.032241	1.325603
H	5.766154	2.926692	0.717446
H	6.970044	1.714044	1.215921
H	5.779330	2.340984	2.365475
H	5.165624	-3.302874	1.515860
H	6.601092	-2.706122	0.674856
C	5.520122	-2.832531	0.590219
H	5.300930	-3.533024	-0.218161
C	3.210461	2.491708	1.205945
C	3.086799	2.723780	2.596685
C	2.666118	3.982867	3.021755
C	2.337299	4.979266	2.105882
C	2.403583	4.712949	0.747004
C	2.839678	3.471465	0.268151
H	2.118370	5.481510	0.030172
H	2.009409	5.954392	2.457032
H	2.578244	4.185797	4.085757
C	3.345540	1.606626	3.594075
H	4.167241	0.987422	3.213451
C	2.120632	0.694609	3.705789
H	1.771497	0.341836	2.725599
H	2.339145	-0.184980	4.321923
H	1.282304	1.229230	4.166593
H	4.610831	2.792841	4.922232
C	3.762493	2.102428	4.973526
H	4.050962	1.259882	5.608830
H	2.943903	2.620152	5.485244
H	3.244123	2.216355	-1.408533
C	2.907379	3.246271	-1.229551
C	3.923491	4.177987	-1.888325
H	3.640933	5.228889	-1.756917
H	4.926392	4.052488	-1.467032
C	1.528299	3.395291	-1.868850
H	1.571101	3.153682	-2.937018
H	0.797481	2.719864	-1.404132
H	1.151369	4.419748	-1.772053
H	3.984595	3.985953	-2.964327
C	2.952394	-2.660118	-0.542115
C	2.151473	-3.440419	0.313757
C	1.468388	-4.532576	-0.226416
C	1.598012	-4.864586	-1.568280
C	2.399032	-4.085261	-2.392468
C	3.070836	-2.955355	-1.915652
C	3.906593	-2.171413	-2.921567
H	2.498977	-4.339526	-3.446584
H	1.068687	-5.723072	-1.974104
H	0.827408	-5.129057	0.421894
C	2.016579	-3.136200	1.793040
H	2.720390	-2.329427	2.041235
C	2.384719	-4.349005	2.647978
H	3.373725	-4.747616	2.397587
H	2.382512	-4.086482	3.710929
H	1.660495	-5.161020	2.514596
H	0.513836	-2.463905	3.210527
C	0.607689	-2.655596	2.134938
H	0.343253	-1.740651	1.588585
H	-0.135844	-3.414608	1.869399
H	3.500577	-2.475055	-3.897972
H	5.491718	-3.684073	-2.922377
C	5.377106	-2.595305	-2.904268
H	5.903647	-2.185386	-3.772594
H	5.887688	-2.213709	-2.013199
H	4.179611	-0.236109	-3.831699
C	3.807317	-0.646299	-2.886070
H	4.421735	-0.211331	-2.089020
H	2.771629	-0.305064	-2.766492
H	0.855490	1.531057	-4.685824

TS-2_ AlPdAl _M06L.log

SCF (M06L) = -3041.21720661
E(SCF)+ZPE(0 K) = -3039.854629
H(298 K) = -3039.773804
G(298 K) = -3039.967228
Lowest Frequency = -329.4915 cm^{-1}

Pd	-0.485069000	0.209760000	1.200721000
Al	1.889109000	0.576542000	0.267684000
N	2.629359000	2.433578000	0.497894000
N	3.425753000	-0.145902000	1.308747000
C	3.545483000	2.824861000	1.391812000
C	4.229812000	1.928851000	2.224129000
H	4.917455000	2.366568000	2.938677000
C	4.240601000	0.534612000	2.127390000
C	3.910781000	4.276454000	1.507731000
H	4.143020000	4.704771000	0.527483000
H	4.767770000	4.416491000	2.167693000
H	3.073801000	4.858234000	1.904894000
C	5.269303000	-0.202439000	2.935737000
H	5.720087000	0.448489000	3.686144000
H	6.068871000	-0.584047000	2.291132000
H	4.839247000	-1.078037000	3.430397000
C	2.105015000	3.419873000	-0.404945000
C	2.606212000	3.447454000	-1.725956000
C	2.125877000	4.424576000	-2.600499000
H	2.511372000	4.466737000	-3.615400000
C	1.148138000	5.329516000	-2.203113000
C	0.631265000	5.255857000	-0.917938000
H	-0.154363000	5.944591000	-0.611561000
C	1.094304000	4.311404000	0.003998000
C	3.737880000	-1.526760000	1.076693000
C	3.046015000	-2.541009000	1.765183000
C	3.431066000	-3.866843000	1.547021000
H	2.916113000	-4.658673000	2.087373000
C	4.436637000	-4.192639000	0.647361000
C	5.064425000	-3.184084000	-0.073025000
H	5.831534000	-3.437254000	-0.803098000
C	4.737158000	-1.841444000	0.130465000
C	-1.716570000	0.440933000	2.963287000
C	-2.890538000	1.213088000	2.851335000
C	-1.665819000	-0.636423000	3.871288000

C	-4.004800000	0.784628000	3.547781000
H	-2.962935000	2.057232000	2.174903000
C	-2.820496000	-1.029239000	4.531482000
H	-0.722533000	-1.146411000	4.035002000
C	-4.019859000	-0.327958000	4.383596000
H	-2.781729000	-1.888721000	5.196932000
H	-4.928219000	-0.607356000	4.903672000
H	0.781138000	6.078607000	-2.899906000
H	4.715954000	-5.231957000	0.492614000
C	1.904708000	-2.251993000	2.719928000
C	2.291017000	-2.538211000	4.169978000
C	0.665470000	-3.047754000	2.318803000
H	1.649576000	-1.184812000	2.640631000
H	3.141583000	-1.932620000	4.499074000
H	1.454747000	-2.324931000	4.843801000
H	2.563167000	-3.592079000	4.303235000
H	0.380334000	-2.832035000	1.283583000
H	0.830144000	-4.128183000	2.405255000
H	-0.186806000	-2.790843000	2.955315000
C	5.442845000	-0.778048000	-0.692081000
C	5.028121000	-0.881371000	-2.159771000
C	6.962966000	-0.856545000	-0.564904000
H	5.126281000	0.208507000	-0.330012000
H	3.949730000	-0.719230000	-2.280700000
H	5.553527000	-0.137713000	-2.770210000
H	5.262262000	-1.872369000	-2.567075000
H	7.292787000	-0.796898000	0.477011000
H	7.352705000	-1.793839000	-0.977306000
H	7.439254000	-0.036910000	-1.112891000
C	0.455043000	4.260806000	1.377175000
C	-0.988395000	3.776757000	1.264729000
C	0.477664000	5.617165000	2.083555000
H	0.998831000	3.535252000	1.994012000
H	-1.052198000	2.781003000	0.802866000
H	-1.450390000	3.710701000	2.253816000
H	-1.580972000	4.470708000	0.655255000
H	1.479947000	6.053849000	2.141277000
H	-0.158929000	6.343540000	1.565681000
H	0.093739000	5.520937000	3.103357000
C	3.645109000	2.444972000	-2.192971000
C	5.060304000	2.857397000	-1.787508000
C	3.569131000	2.165865000	-3.689013000
H	3.426086000	1.492318000	-1.680887000
H	5.175067000	2.914165000	-0.700356000
H	5.797539000	2.136207000	-2.159393000
H	5.314782000	3.838350000	-2.205931000
H	2.546361000	1.928770000	-4.001008000
H	3.913334000	3.018178000	-4.285262000
H	4.207649000	1.316235000	-3.950311000
Al	-1.625105000	-0.701764000	-0.681662000
N	-2.945956000	0.132649000	-1.860992000
N	-1.372832000	-2.183600000	-1.913251000
C	-1.898082000	-2.330460000	-3.137080000
C	-2.794175000	-1.412694000	-3.693868000
C	-3.362953000	-0.302777000	-3.053753000
H	-5.465188000	-0.019122000	-3.261202000
H	-4.543947000	1.432860000	-3.594858000
C	-4.538888000	0.348304000	-3.719686000
H	-4.575536000	0.103623000	-4.782543000
H	-3.171771000	-1.645871000	-4.682922000
C	-1.543562000	-3.541019000	-3.950480000
H	-1.756169000	-4.462340000	-3.398384000
H	-0.470543000	-3.563407000	-4.169100000
H	-2.091053000	-3.558297000	-4.893567000
C	-2.069510000	3.030517000	-2.155605000
H	-1.762214000	3.967786000	-1.667381000
H	-3.432032000	4.026179000	-3.553885000
C	-2.528136000	3.408441000	-3.566126000
H	-1.739907000	3.968627000	-4.081319000
H	-2.734122000	2.516852000	-4.169284000
C	-0.809018000	2.177237000	-2.234478000
H	-0.484930000	1.825962000	-1.246804000
H	-3.616118000	4.527332000	-0.656734000
C	-3.947965000	3.491881000	-0.593738000
C	-3.226936000	2.523852000	-1.304028000
C	-3.675779000	1.191755000	-1.216621000
C	-4.805144000	0.842023000	-0.445935000
C	-5.472959000	1.847012000	0.254192000
C	-5.049604000	3.167255000	0.185198000
H	-5.572252000	3.938782000	0.743399000
H	-6.322699000	1.584567000	0.879196000
C	-5.301784000	-0.589034000	-0.336452000
H	-4.750426000	-1.208025000	-1.057244000
C	-5.036992000	-1.154144000	1.058265000
H	-3.973295000	-1.113595000	1.325769000
H	-5.577772000	-0.581753000	1.819527000
H	-5.370400000	-2.196370000	1.123756000
H	-7.407626000	-0.183870000	0.056697000
C	-6.786363000	-0.705555000	-0.679159000
H	-7.099041000	-1.754956000	-0.681768000
C	2.391242000	-3.311641000	-3.586749000
H	2.951729000	-4.169268000	-3.206208000
H	3.099797000	-2.657947000	-4.106183000
H	1.685769000	-3.687798000	-4.337730000
C	1.666864000	-2.532163000	-2.483401000
C	0.756466000	-3.446703000	-1.659962000
C	-0.610522000	-3.272505000	-1.369374000
H	2.438153000	-2.216339000	-1.758492000
C	-1.296635000	-4.143280000	-0.486808000
C	-0.621758000	-5.254629000	0.013888000
H	-1.139187000	-5.939109000	0.680508000
C	0.715830000	-5.469176000	-0.292319000
C	1.389449000	-4.561133000	-1.090736000
H	2.452940000	-4.698474000	-1.265707000
H	1.244379000	-6.323983000	0.122892000
C	1.112016000	-1.246431000	-3.088670000
H	0.657342000	-0.588520000	-2.343876000
H	1.946242000	-0.687786000	-3.531222000
H	0.388312000	-1.423864000	-3.891751000
H	-2.285574000	-3.905880000	2.062067000
H	-2.881468000	-2.781004000	-0.079785000
C	-3.020909000	-4.316686000	1.362666000
H	-4.008606000	-3.965239000	1.675374000
H	-3.020906000	-5.407188000	1.466387000
C	-2.732545000	-3.873706000	-0.067297000
C	-3.746199000	-4.469021000	-1.043711000
H	-3.648009000	-4.044790000	-2.048208000
H	-4.770669000	-4.276761000	-0.704979000
H	-3.619481000	-5.555134000	-1.123104000
H	-7.019779000	-0.280975000	-1.661278000
H	-0.929322000	1.306506000	-2.889200000
H	0.009390000	2.778380000	-2.644829000
F	-0.350344000	1.325941000	3.106540000
F	-5.164305000	1.469569000	3.378403000

TS-2'_AlPdPCy$_3$ _M06L.log

SCF (M06L) = -2847.06337815
E(SCF)+ZPE(0 K) = -2845.855981
H(298 K) = -2845.789874
G(298 K) = -2845.955367
Lowest Frequency = -309.2826 cm^{-1}

Pd	0.934528	-0.007376	-1.058735
P	2.019717	-0.306913	0.941333
C	2.085093	1.255639	1.948614
H	2.701193	1.051436	2.839843
C	0.721881	1.749085	2.439636
H	0.076769	1.951969	1.569407
H	0.212518	0.973143	3.027475
C	0.877644	3.018379	3.272313
H	-0.105843	3.365302	3.613166
H	1.458315	2.785571	4.178189
C	1.585080	4.116116	2.486530
H	1.708564	5.013597	3.104451
H	0.948123	4.407608	1.638313
C	2.929183	3.635584	1.953550

H	3.610421	3.437342	2.795346
H	3.406618	4.414873	1.348593
C	2.764312	2.362729	1.132771
H	3.730645	2.030613	0.730528
H	2.133310	2.567149	0.253812
C	3.829818	-0.764138	0.812937
H	4.152516	-0.180137	-0.066715
C	4.747901	-0.383432	1.980949
H	4.445840	-0.927525	2.888031
H	4.662157	0.682547	2.217850
C	6.203659	-0.708726	1.655226
H	6.519764	-0.088518	0.802899
H	6.847743	-0.431158	2.497910
C	6.383080	-2.177906	1.297272
H	6.137471	-2.796325	2.174245
H	7.430352	-2.390439	1.053880
C	5.473688	-2.560770	0.137198
H	5.581649	-3.623881	-0.108351
H	5.774097	-1.997419	-0.756509
C	4.016312	-2.241543	0.450478
H	3.369506	-2.504251	-0.397713
H	3.696172	-2.863696	1.300091
C	1.149568	-1.510388	2.058617
H	0.190547	-0.987967	2.228562
C	0.783728	-2.822696	1.353277
H	0.268140	-2.593418	0.408782
H	1.692433	-3.375768	1.084690
C	-0.090960	-3.701838	2.240566
H	-0.293232	-4.654067	1.737324
H	-1.069272	-3.216998	2.367248
C	0.536050	-3.935274	3.610084
H	-0.127249	-4.541570	4.238058
H	1.463357	-4.516124	3.491681
C	0.865586	-2.615464	4.296801
H	1.320955	-2.789387	5.278664
H	-0.066924	-2.057845	4.480034
C	1.785556	-1.764800	3.426088
H	2.742297	-2.292329	3.293657
H	2.022460	-0.817245	3.927573
Al	-1.581482	0.099439	-0.466254
N	-2.848774	1.613776	-0.610111
N	-2.962309	-1.119293	-1.199346
C	-3.976731	1.664020	-1.335568
C	-4.533020	0.536293	-1.949175
H	-5.448096	0.693147	-2.508896
C	-4.112480	-0.791653	-1.799838
C	-4.713632	2.964303	-1.475080
H	-4.940794	3.394896	-0.494197
H	-5.644913	2.834401	-2.027639
H	-4.101277	3.707027	-1.997673
C	-5.038400	-1.867086	-2.289825
H	-4.495286	-2.702588	-2.739049
H	-5.752453	-1.468487	-3.012524
H	-5.610966	-2.283910	-1.452886
C	-2.480142	2.780166	0.143220
C	-2.773400	2.796102	1.524324
C	-2.456861	3.944443	2.252865
H	-2.684049	3.982093	3.314896
C	-1.838018	5.032287	1.648328
C	-1.500339	4.972656	0.303510
H	-0.976037	5.807266	-0.156961
C	-1.807184	3.851531	-0.472242
C	-2.705718	-2.503456	-0.920517
C	-1.742318	-3.191080	-1.686512
C	-1.504603	-4.534903	-1.386858
H	-0.769944	-5.085327	-1.967957

C	-2.184989	-5.176708	-0.358990
C	-3.097313	-4.469867	0.413312
H	-3.596883	-4.963872	1.244403
C	-3.368782	-3.124291	0.157305
C	2.603628	0.231200	-2.391969
C	3.238339	1.485151	-2.258246
C	3.372867	-0.897972	-2.754591
C	4.618840	1.533532	-2.324667
H	2.674403	2.384844	-2.040292
C	4.752538	-0.782289	-2.841096
H	2.882712	-1.849867	-2.927673
C	5.414864	0.428403	-2.611871
H	5.334606	-1.666332	-3.092478
H	6.491519	0.527734	-2.676658
F	1.230667	0.321241	-3.243752
F	5.219376	2.724306	-2.096384
H	-1.591480	5.913427	2.234918
H	-1.986866	-6.223792	-0.146112
C	-1.002419	-2.497418	-2.812390
C	-1.827881	-2.462941	-4.097666
C	0.380318	-3.077746	-3.073349
H	-0.842863	-1.452245	-2.501320
H	-2.770066	-1.922957	-3.964090
H	-1.271458	-1.961818	-4.895773
H	-2.066689	-3.477755	-4.437966
H	0.973984	-3.118611	-2.151306
H	0.335630	-4.087092	-3.498813
H	0.912664	-2.443250	-3.787429
C	-4.295126	-2.356004	1.082943
C	-3.578712	-2.043910	2.398400
C	-5.606974	-3.086912	1.356492
H	-4.541662	-1.395185	0.613150
H	-2.641252	-1.496630	2.228819
H	-4.211215	-1.438884	3.058396
H	-3.324892	-2.967170	2.932880
H	-6.127435	-3.359665	0.432778
H	-5.444848	-4.010321	1.922761
H	-6.280346	-2.459434	1.949295
C	-1.340900	3.784127	-1.912155
C	0.153676	3.462235	-1.948123
C	-1.638870	5.058203	-2.697940
H	-1.864909	2.955079	-2.403975
H	0.368563	2.521864	-1.418033
H	0.513888	3.352668	-2.976014
H	0.735130	4.257201	-1.462663
H	-2.697965	5.334517	-2.653237
H	-1.063775	5.910409	-2.320518
H	-1.368156	4.930668	-3.750045
C	-3.404045	1.593775	2.202454
C	-4.917652	1.536266	1.991897
C	-3.066154	1.496333	3.684797
H	-2.977766	0.697727	1.720443
H	-5.185019	1.454982	0.934095
H	-5.348309	0.669190	2.506498
H	-5.399822	2.434496	2.394991
H	-1.989128	1.585335	3.861954
H	-3.567733	2.273661	4.271822
H	-3.395284	0.532391	4.085082

TS-3_CH_AlPdAl _M06L.log

SCF (M06L) = -3041.24993981
E(SCF)+ZPE(0 K)= -3039.89118
H(298 K) = -3039.809948
G(298 K) = -3040.004255

Lowest Frequency = -712.0310 cm^{-1}

Pd	-0.022240000	-0.164670000	1.204672000
Al	-1.808074000	-0.007482000	-0.431208000
N	-3.051987000	1.512471000	-0.477897000
N	-2.485412000	-0.681714000	-2.158197000
C	-3.769613000	1.939526000	-1.522820000
C	-3.857578000	1.219517000	-2.722878000
H	-4.496103000	1.642439000	-3.490187000
C	-3.354374000	-0.060583000	-2.974044000
C	-4.577011000	3.199994000	-1.414832000
H	-5.239802000	3.163916000	-0.543999000
H	-5.176788000	3.362936000	-2.311266000
H	-3.929152000	4.070302000	-1.266021000
C	-3.899722000	-0.774489000	-4.179281000
H	-3.147711000	-1.376212000	-4.694720000
H	-4.342172000	-0.067116000	-4.882779000
H	-4.688561000	-1.470802000	-3.869368000
C	-3.202562000	2.213543000	0.773609000
C	-4.001234000	1.620047000	1.772543000
C	-4.202481000	2.328274000	2.959222000
H	-4.806514000	1.886456000	3.746460000
C	-3.623651000	3.574767000	3.160706000
H	-3.795357000	4.109344000	4.091305000
C	-2.791812000	4.113602000	2.190205000
H	-2.292974000	5.062506000	2.373562000
C	-2.551771000	3.441124000	0.991073000
C	-4.618647000	0.248450000	1.572844000
H	-3.876861000	-0.359699000	1.026661000
C	-4.885072000	-0.479188000	2.883625000
H	-4.002569000	-0.479672000	3.530375000
H	-5.161902000	-1.520184000	2.689314000
H	-5.715569000	-0.027397000	3.438322000
C	-5.883317000	0.300001000	0.715118000
H	-6.647268000	0.927978000	1.188639000
H	-6.308762000	-0.703500000	0.593700000
H	-5.692480000	0.699937000	-0.286096000
C	-1.521382000	3.971429000	0.018648000
H	-1.681767000	3.481101000	-0.948991000
C	-0.125874000	3.578440000	0.507169000
H	0.640247000	3.897678000	-0.205864000
H	-0.038096000	2.492233000	0.652754000
H	0.088424000	4.046541000	1.475336000
C	-1.605137000	5.476499000	-0.214080000
H	-2.604189000	5.794992000	-0.532999000
H	-0.889860000	5.778115000	-0.987436000
H	-1.353773000	6.042449000	0.689417000
C	-2.172880000	-2.050680000	-2.469314000
C	-0.982725000	-2.353858000	-3.160053000
C	-0.766948000	-3.680018000	-3.545032000
H	0.130201000	-3.930603000	-4.105681000
C	-1.677077000	-4.681887000	-3.230896000
H	-1.489160000	-5.705470000	-3.543164000
C	-2.812523000	-4.373752000	-2.492985000
H	-3.503441000	-5.165530000	-2.212896000
C	-3.080033000	-3.062116000	-2.093975000
C	0.001614000	-1.269404000	-3.556217000
H	-0.104338000	-0.448379000	-2.831616000
C	1.458062000	-1.719387000	-3.511621000
H	1.672440000	-2.523061000	-4.224997000
H	1.750522000	-2.073408000	-2.517693000
H	2.119614000	-0.885408000	-3.772515000
C	-0.328383000	-0.716737000	-4.943247000
H	-0.327059000	-1.518175000	-5.691577000
H	0.417237000	0.024678000	-5.250389000
H	-1.309300000	-0.232587000	-4.973851000
C	-4.285386000	-2.770075000	-1.220325000
H	-4.482570000	-1.689886000	-1.250377000
C	-3.971803000	-3.141011000	0.232216000
H	-3.746076000	-4.210461000	0.318499000
H	-4.826776000	-2.923271000	0.882317000
H	-3.102403000	-2.594528000	0.621066000
C	-5.553018000	-3.477344000	-1.691467000
H	-5.771269000	-3.274130000	-2.745169000
H	-6.414231000	-3.150667000	-1.100394000
H	-5.480137000	-4.563985000	-1.575251000
Al	1.962374000	-0.037225000	-0.142970000
N	3.245370000	-1.536393000	-0.140140000
N	3.064623000	0.988894000	-1.401122000
C	4.263638000	-1.718649000	-0.980827000
C	4.566993000	-0.804538000	-2.004897000
H	5.367782000	-1.088515000	-2.678709000
C	4.064009000	0.489969000	-2.154903000
C	5.181617000	-2.894225000	-0.819332000
H	4.662471000	-3.772722000	-0.429996000
H	5.973708000	-2.651367000	-0.101642000
H	5.665279000	-3.143786000	-1.765988000
C	4.704749000	1.360999000	-3.196446000
H	3.972017000	1.665887000	-3.951982000
H	5.522884000	0.841785000	-3.696954000
H	5.090455000	2.287211000	-2.757735000
C	3.105275000	-2.391842000	1.009519000
C	3.945886000	-2.199003000	2.121678000
C	3.730611000	-2.982372000	3.258332000
H	4.369005000	-2.836584000	4.127652000
C	2.703366000	-3.912465000	3.306659000
H	2.542005000	-4.502607000	4.204407000
C	1.868608000	-4.073177000	2.208204000
H	1.045630000	-4.779256000	2.261227000
C	2.046009000	-3.321871000	1.045983000
C	5.005663000	-1.115927000	2.179085000
H	5.092428000	-0.647754000	1.188927000
C	4.577773000	-0.030773000	3.167928000
H	4.493789000	-0.442016000	4.179967000
H	3.599706000	0.390529000	2.908579000
H	5.313342000	0.781262000	3.200627000
C	6.381393000	-1.660080000	2.560308000
H	7.136734000	-0.868231000	2.517651000
H	6.705833000	-2.472035000	1.901468000
H	6.382710000	-2.054393000	3.582327000
C	1.152374000	-3.542188000	-0.156884000
H	1.001068000	-2.564481000	-0.639462000
C	1.831535000	-4.469695000	-1.165403000
H	2.761894000	-4.044232000	-1.557569000
H	1.170357000	-4.663766000	-2.016583000
H	2.074435000	-5.432812000	-0.700695000
C	-0.237380000	-4.048502000	0.198024000
H	-0.865138000	-4.055237000	-0.697098000
H	-0.707642000	-3.404433000	0.945430000
H	-0.219266000	-5.074749000	0.584903000
C	2.854040000	2.414064000	-1.442222000
C	2.075396000	3.016145000	-2.448369000
C	1.985031000	4.411056000	-2.477441000
H	1.383116000	4.885545000	-3.249609000
C	2.632126000	5.196479000	-1.535037000
H	2.558253000	6.279566000	-1.580177000
C	3.340639000	4.586096000	-0.508390000
H	3.808450000	5.200348000	0.255564000
C	3.450806000	3.196002000	-0.428098000
C	1.258612000	2.215143000	-3.441276000
H	1.582447000	1.166882000	-3.387394000
C	-0.215557000	2.269171000	-3.031819000
H	-0.585467000	3.302356000	-3.049977000
H	-0.347961000	1.893572000	-2.007580000
H	-0.847064000	1.676240000	-3.702984000
C	1.434140000	2.699359000	-4.879051000
H	0.865685000	2.073476000	-5.574062000
H	2.482533000	2.686492000	-5.194938000
H	1.069220000	3.723880000	-5.005952000
C	4.202761000	2.561028000	0.727109000
H	3.753552000	1.572343000	0.911126000
C	4.056501000	3.352336000	2.021578000
H	4.574390000	4.316326000	1.976954000
H	4.490930000	2.797290000	2.856924000
H	3.003685000	3.540113000	2.255056000
C	5.674125000	2.324883000	0.385512000
H	6.176011000	3.270624000	0.149410000
H	5.793412000	1.656513000	-0.473924000
H	6.200864000	1.869617000	1.232336000
H	0.739871000	-0.231186000	2.643760000
C	-0.807898000	-0.311255000	3.156595000

C	-1.083033000	0.825267000	3.920234000
C	-1.239752000	-1.496701000	3.752253000
C	-1.753096000	0.822969000	5.135709000
C	-1.903854000	-1.576832000	4.968981000
C	-2.164424000	-0.398264000	5.666022000
H	-1.937419000	1.763094000	5.646339000
H	-2.214872000	-2.545921000	5.346161000
H	-2.684739000	-0.432210000	6.618380000
F	-1.016403000	-2.665910000	3.092216000
F	-0.656838000	2.022278000	3.440554000

TS-3'_AlPdPCy$_3$ _M06L.log

SCF (M06L) = -2847.07654735
E(SCF)+ZPE(0 K) = -2845.870577
H(298 K) = -2845.804855
G(298 K) = -2845.968666
Lowest Frequency = -837.1260 cm^{-1}

Pd	-0.724729	0.080571	-0.803117
P	-2.085925	-0.822873	0.909237
C	-1.638651	-2.582161	1.305983
H	-2.392211	-2.994484	1.997596
C	-0.264565	-2.716610	1.968151
H	0.488976	-2.242582	1.316646
H	-0.238378	-2.168505	2.920650
C	0.101124	-4.181671	2.190677
H	1.100339	-4.256875	2.639076
H	-0.598387	-4.626734	2.914626
C	0.052451	-4.969126	0.887536
H	0.317459	-6.019231	1.061368
H	0.812218	-4.565920	0.203150
C	-1.312446	-4.850492	0.226114
H	-2.077405	-5.320288	0.863692
H	-1.328940	-5.392474	-0.726857
C	-1.671594	-3.388576	-0.000554
H	-2.650882	-3.308177	-0.487591
H	-0.952869	-2.930916	-0.699745
C	-3.843381	-1.008620	0.292189
H	-3.659226	-1.316812	-0.751481
C	-4.764096	-2.068239	0.908454
H	-5.037794	-1.785066	1.933758
H	-4.262801	-3.040339	0.981893
C	-6.031946	-2.209499	0.067510
H	-5.752117	-2.582718	-0.929348
H	-6.694955	-2.965317	0.505065
C	-6.760609	-0.878777	-0.083477
H	-7.126820	-0.557828	0.903613
H	-7.647978	-0.997936	-0.715878
C	-5.837464	0.195760	-0.646022
H	-6.357834	1.158634	-0.713953
H	-5.541678	-0.067051	-1.672415
C	-4.577625	0.333079	0.201557
H	-3.916933	1.104898	-0.207634
H	-4.858833	0.665296	1.213204
C	-2.139754	0.054773	2.549758
H	-1.166369	-0.197305	3.004558
C	-2.149045	1.577478	2.344483
H	-1.293039	1.866362	1.716715
H	-3.043891	1.870403	1.779292
C	-2.134664	2.332989	3.669208
H	-2.204939	3.411633	3.482827
H	-1.169028	2.170188	4.169853
C	-3.245881	1.869048	4.603032
H	-3.205061	2.415435	5.552360
H	-4.222751	2.102249	4.153020
C	-3.154857	0.366753	4.841735
H	-3.947688	0.030883	5.520219
H	-2.201172	0.135974	5.340155
C	-3.233187	-0.393656	3.521931
H	-4.217826	-0.204277	3.072627
H	-3.175221	-1.477067	3.691206
Al	1.643293	0.372844	-0.413123
N	3.335527	-0.588321	-0.636219
N	2.593847	2.100131	-0.389337
C	4.505309	-0.060189	-1.040755
C	4.734256	1.316807	-1.125438
H	5.726145	1.621970	-1.438409
C	3.864998	2.337991	-0.716061
C	5.653606	-0.969661	-1.369480
H	5.890992	-1.626422	-0.525931
H	6.544936	-0.399437	-1.633548
H	5.401883	-1.630574	-2.205916
C	4.407746	3.731031	-0.617126
H	3.905655	4.398822	-1.324224
H	5.479610	3.759283	-0.816111
H	4.219909	4.146537	0.378192
C	3.319551	-2.001307	-0.358361
C	3.487857	-2.420244	0.979628
C	3.591174	-3.790964	1.230190
H	3.734549	-4.138609	2.249394
C	3.505052	-4.720035	0.200254
H	3.591475	-5.781367	0.417327
C	3.258635	-4.291130	-1.096494
H	3.137184	-5.023235	-1.891277
C	3.150028	-2.931610	-1.400301
C	3.548236	-1.413847	2.116539
H	2.812176	-0.626250	1.880489
C	3.145566	-2.007023	3.461263
H	2.174051	-2.508039	3.414343
H	3.081698	-1.218872	4.217440
H	3.883085	-2.731379	3.824270
C	4.915252	-0.738571	2.243016
H	5.698587	-1.483929	2.424374
H	4.919204	-0.042421	3.089703
H	5.189712	-0.167923	1.351987
C	2.780144	-2.494930	-2.802958
H	3.043620	-1.434769	-2.910274
C	1.264599	-2.612397	-2.982504
H	0.951989	-2.270482	-3.974293
H	0.720132	-2.011274	-2.243275
H	0.940772	-3.654732	-2.866146
C	3.519684	-3.267608	-3.891042
H	4.605997	-3.242947	-3.751268
H	3.297186	-2.846146	-4.875656
H	3.216690	-4.319688	-3.920036
C	1.767687	3.146025	0.146624
C	1.063923	4.014661	-0.709884
C	0.110537	4.863914	-0.142018
H	-0.462287	5.520016	-0.794481
C	-0.121031	4.882936	1.227287
H	-0.875081	5.545006	1.644415
C	0.627276	4.063190	2.062594
H	0.465294	4.093731	3.139186
C	1.576186	3.180397	1.543136
C	1.325987	4.087736	-2.202329
H	2.150488	3.402788	-2.437491
C	0.120340	3.653492	-3.030493
H	-0.746620	4.291171	-2.828992
H	-0.170594	2.625894	-2.802828
H	0.346486	3.722489	-4.099727
C	1.750703	5.501274	-2.608283

H	0.924541	6.209327	-2.480524
H	2.043080	5.530215	-3.662717
H	2.589370	5.877231	-2.012665
C	2.390572	2.318615	2.490909
H	3.070920	1.697063	1.892994
C	1.509411	1.380354	3.311385
H	0.840000	1.944759	3.969724
H	2.119322	0.727988	3.946122
H	0.887397	0.747154	2.664663
C	3.254159	3.181662	3.410865
H	3.921068	3.840042	2.845401
H	3.872199	2.557902	4.065390
H	2.633370	3.817419	4.052106
H	-0.531327	0.596329	-2.330561
C	-2.091862	0.438873	-2.375441
C	-2.525008	-0.543552	-3.265789
C	-2.822387	1.625629	-2.452197
C	-3.584330	-0.397214	-4.151385
C	-3.885758	1.846654	-3.315488
C	-4.262934	0.819601	-4.178840
H	-3.859711	-1.221615	-4.801152
H	-4.408924	2.797327	-3.292303
H	-5.091790	0.963711	-4.864938
F	-1.876840	-1.739653	-3.250542
F	-2.500522	2.624208	-1.582399

TS-3_ 1,2,3-TriFB_AlPdAl _M06L.log

SCF (M06L) = -3140.45757698
E(SCF)+ZPE(0 K) = -3139.106370
H(298 K) = -3139.024277
G(298 K) = -3139.220715
Lowest Frequency = -751.6146 cm^{-1}

Pd	-0.109220	0.973941	-0.494960
Al	-1.668588	-0.700516	0.323186
N	-2.801716	-1.696164	-0.945522
N	-2.150402	-1.928907	1.789787
C	-3.357806	-2.899302	-0.762738
C	-3.335499	-3.562811	0.472147
H	-3.846976	-4.517861	0.512642
C	-2.874969	-3.055858	1.691310
C	-4.090739	-3.566551	-1.889792
H	-4.859014	-2.906035	-2.304795
H	-4.560577	-4.494562	-1.561504
H	-3.409514	-3.795405	-2.716447
C	-3.299998	-3.789448	2.932215
H	-2.521192	-3.818249	3.697586
H	-3.612583	-4.807754	2.694806
H	-4.156893	-3.277582	3.387095
C	-3.095901	-1.005305	-2.176379
C	-4.084690	-0.000862	-2.148055
C	-4.445509	0.602762	-3.354073
H	-5.207583	1.377130	-3.354144
C	-3.827389	0.253759	-4.548037
H	-4.125277	0.734871	-5.475988
C	-2.800193	-0.679601	-4.543053
H	-2.279843	-0.916018	-5.468777
C	-2.410498	-1.319391	-3.363734
C	-4.740599	0.422421	-0.847630
H	-3.991680	0.292512	-0.048541
C	-5.121052	1.897068	-0.837042
H	-4.268580	2.525017	-1.115431
H	-5.452803	2.197791	0.161378
H	-5.944996	2.116519	-1.524763
C	-5.932849	-0.465092	-0.491991
H	-6.702653	-0.414121	-1.270915
H	-6.391196	-0.141722	0.450046
H	-5.642857	-1.515165	-0.375231
C	-1.213536	-2.244067	-3.369525
H	-1.221406	-2.813252	-2.432738
C	0.065593	-1.406131	-3.389034
H	0.953178	-2.044322	-3.340021
H	0.100016	-0.702203	-2.544208
H	0.123236	-0.815578	-4.312481
C	-1.214687	-3.247731	-4.518753
H	-2.134879	-3.841669	-4.555077
H	-0.369684	-3.937685	-4.417084
H	-1.107139	-2.752845	-5.490144
C	-1.861818	-1.453431	3.116045
C	-0.604000	-1.721752	3.692109
C	-0.390498	-1.327169	5.015573
H	0.562524	-1.550331	5.488806
C	-1.370668	-0.657606	5.738032
H	-1.181488	-0.359899	6.765688
C	-2.581797	-0.349370	5.132964
H	-3.333790	0.207187	5.687335
C	-2.850543	-0.735083	3.817575
C	0.465359	-2.487599	2.936116
H	0.297406	-2.312930	1.862964
C	1.884777	-2.025938	3.247982
H	2.162548	-2.197651	4.294002
H	2.028263	-0.960883	3.038792
H	2.605667	-2.582285	2.637911
C	0.341987	-3.990415	3.190783
H	0.410617	-4.209927	4.262849
H	1.149800	-4.534363	2.688930
H	-0.606811	-4.396433	2.826804
C	-4.153341	-0.316701	3.162692
H	-4.302832	-0.928565	2.262457
C	-4.060414	1.145006	2.716221
H	-3.881949	1.802174	3.575311
H	-4.991311	1.464858	2.234915
H	-3.238122	1.308206	2.006547
C	-5.367880	-0.525547	4.063179
H	-5.427743	-1.550826	4.443145
H	-6.291380	-0.315216	3.515051
H	-5.351836	0.144468	4.929338
Al	2.031618	0.018241	0.038132
N	3.244968	0.948278	1.281542
N	3.316990	-1.439987	-0.227891
C	4.345657	0.449849	1.846052
C	4.805834	-0.851568	1.580310
H	5.666846	-1.181390	2.151038
C	4.377160	-1.704121	0.560692
C	5.186407	1.300239	2.751898
H	4.586593	2.011470	3.324004
H	5.893940	1.890503	2.158123
H	5.771302	0.680861	3.434892
C	5.166973	-2.960023	0.332705
H	4.537797	-3.844961	0.479940
H	6.017016	-3.021988	1.013120
H	5.534419	-3.017490	-0.697203
C	2.944044	2.348775	1.432250
C	3.666564	3.295032	0.681190
C	3.311834	4.641342	0.795455
H	3.856485	5.381580	0.212568
C	2.266747	5.042913	1.614021
H	1.998682	6.093312	1.683367
C	1.549601	4.092715	2.328811
H	0.711210	4.406762	2.943247

C	1.861874	2.735185	2.248559
C	4.743913	2.909762	-0.314150
H	4.960783	1.838082	-0.207788
C	4.233865	3.139238	-1.737401
H	4.014600	4.199926	-1.902461
H	3.308318	2.584275	-1.929902
H	4.985108	2.837045	-2.476280
C	6.048941	3.672799	-0.094076
H	6.827105	3.313742	-0.775848
H	6.423726	3.570578	0.929495
H	5.920694	4.743774	-0.284809
C	1.091175	1.721405	3.068749
H	1.047119	0.787304	2.488170
C	1.824992	1.433053	4.379138
H	2.816070	0.997465	4.209061
H	1.254661	0.731258	4.996406
H	1.960934	2.356032	4.955402
C	-0.355917	2.115779	3.327852
H	-0.884337	1.281805	3.799359
H	-0.867031	2.363625	2.393414
H	-0.438452	2.973280	4.006586
C	3.162267	-2.233419	-1.420241
C	2.509922	-3.480329	-1.395757
C	2.452664	-4.226436	-2.577105
H	1.945486	-5.189078	-2.569420
C	3.020899	-3.761369	-3.753614
H	2.977589	-4.361951	-4.658079
C	3.612058	-2.504630	-3.776452
H	4.017470	-2.123328	-4.709126
C	3.675077	-1.710731	-2.629069
C	1.806250	-4.016525	-0.165630
H	2.089536	-3.391582	0.692320
C	0.292389	-3.893251	-0.351442
H	-0.043318	-4.479215	-1.216807
H	0.006783	-2.848149	-0.532873
H	-0.255516	-4.252398	0.526766
C	2.188934	-5.462456	0.145419
H	1.703261	-5.805462	1.064376
H	3.269606	-5.588148	0.268559
H	1.872359	-6.141804	-0.652859
C	4.279235	-0.320092	-2.699771
H	3.766470	0.300172	-1.947670
C	4.045316	0.348674	-4.049424
H	4.619364	-0.128566	-4.850903
H	4.358669	1.395242	-4.014097
H	2.986919	0.324108	-4.327738
C	5.764925	-0.322074	-2.338701
H	6.331406	-0.963017	-3.024510
H	5.940190	-0.680957	-1.319178
H	6.181302	0.689698	-2.407093
H	0.459217	2.240552	-1.338811
C	-1.156787	2.564168	-1.418052
C	-1.643439	2.484821	-2.735528
C	-1.548236	3.690759	-0.697882
C	-2.486425	3.449637	-3.285327
C	-2.380245	4.674569	-1.217686
C	-2.847896	4.548594	-2.521644
H	-2.869167	3.361039	-4.297677
F	-1.137669	3.862954	0.581137
H	-1.369441	1.626842	-3.347230
F	-2.740120	5.733424	-0.472856
F	-3.650998	5.510543	-3.015825

TS-3_ FB_AlPdAl _M06L.log

SCF (M06L) = -2942.01857184

E(SCF)+ZPE(0 K) = -2940.651609
H(298 K) = -2940.571377
G(298 K) = -2940.763163
Lowest Frequency = -796.9908 cm^{-1}

Pd	-0.011597	-0.260523	1.233432
Al	-1.817147	-0.001146	-0.373470
N	-3.033633	1.544817	-0.352943
N	-2.517633	-0.586796	-2.124723
C	-3.742283	2.030797	-1.377825
C	-3.847377	1.364392	-2.607610
H	-4.477201	1.833224	-3.355252
C	-3.375674	0.083708	-2.911540
C	-4.522454	3.302743	-1.215769
H	-5.189650	3.242760	-0.349625
H	-5.114833	3.518924	-2.105864
H	-3.855934	4.150699	-1.025812
C	-3.941269	-0.571720	-4.140227
H	-3.207178	-1.178760	-4.674920
H	-4.360476	0.172124	-4.820073
H	-4.752577	-1.252457	-3.854624
C	-3.181177	2.185560	0.930854
C	-3.997116	1.555717	1.892799
C	-4.201060	2.209211	3.109944
H	-4.821761	1.739691	3.867855
C	-3.609994	3.437718	3.375108
H	-3.785694	3.931130	4.327433
C	-2.760655	4.010678	2.439548
H	-2.253069	4.944119	2.672021
C	-2.515729	3.392013	1.212899
C	-4.640466	0.209009	1.618061
H	-3.916116	-0.378439	1.027972
C	-4.911671	-0.591775	2.884556
H	-4.021275	-0.661720	3.516575
H	-5.225872	-1.608496	2.628901
H	-5.719984	-0.150706	3.479199
C	-5.909762	0.338282	0.775257
H	-6.656583	0.953353	1.291163
H	-6.357940	-0.646908	0.598017
H	-5.716762	0.793012	-0.202043
C	-1.466988	3.950831	0.276318
H	-1.625529	3.508732	-0.714697
C	-0.083340	3.510639	0.759218
H	0.696903	3.848737	0.069366
H	-0.016368	2.417507	0.853877
H	0.128949	3.929350	1.750469
C	-1.524138	5.466192	0.113857
H	-2.515027	5.815662	-0.198123
H	-0.797197	5.791891	-0.638552
H	-1.272136	5.985097	1.045001
C	-2.232945	-1.950039	-2.480062
C	-1.042022	-2.255238	-3.169594
C	-0.836718	-3.577026	-3.573436
H	0.063428	-3.828593	-4.128691
C	-1.758056	-4.575171	-3.279531
H	-1.576815	-5.596125	-3.604311
C	-2.896669	-4.266129	-2.547653
H	-3.598115	-5.054638	-2.284441
C	-3.154180	-2.957719	-2.130883
C	-0.041557	-1.175344	-3.534924
H	-0.120800	-0.383880	-2.774317
C	1.405773	-1.655477	-3.532075
H	1.600607	-2.404016	-4.308405
H	1.695657	-2.091793	-2.570548
H	2.082189	-0.816064	-3.730310

C	-0.378176	-0.558033	-4.892791
H	-0.408885	-1.328303	-5.672590
H	0.382180	0.175668	-5.181661
H	-1.346114	-0.047689	-4.887869
C	-4.361229	-2.672902	-1.257680
H	-4.532171	-1.588009	-1.242162
C	-4.070124	-3.113658	0.179604
H	-3.865301	-4.190129	0.220328
H	-4.926690	-2.908507	0.831319
H	-3.195480	-2.598363	0.598182
C	-5.639516	-3.329808	-1.771452
H	-5.844110	-3.072103	-2.815873
H	-6.499030	-3.013551	-1.172302
H	-5.589748	-4.422106	-1.707372
Al	1.956440	-0.033685	-0.123671
N	3.213306	-1.558294	-0.172221
N	3.076722	1.008933	-1.351436
C	4.215771	-1.736586	-1.033608
C	4.530055	-0.796021	-2.029978
H	5.314121	-1.077336	-2.724281
C	4.054791	0.514202	-2.133283
C	5.101887	-2.942423	-0.926237
H	4.556297	-3.824210	-0.582456
H	5.895638	-2.758896	-0.192963
H	5.583837	-3.159071	-1.881704
C	4.696367	1.404244	-3.157627
H	3.957129	1.743470	-3.892026
H	5.498330	0.887232	-3.685866
H	5.103240	2.310618	-2.697222
C	3.078930	-2.439902	0.957636
C	3.950615	-2.294841	2.053718
C	3.750767	-3.105624	3.173926
H	4.413073	-2.993841	4.030175
C	2.712627	-4.023227	3.222344
H	2.567633	-4.639752	4.104974
C	1.847240	-4.136714	2.141864
H	1.023904	-4.844040	2.188906
C	2.002106	-3.349539	0.999630
C	5.028732	-1.230244	2.115912
H	5.104566	-0.740360	1.135637
C	4.634472	-0.163027	3.137856
H	4.566167	-0.596354	4.141814
H	3.656548	0.275817	2.908912
H	5.380594	0.639137	3.173147
C	6.403006	-1.802676	2.458676
H	7.168288	-1.020297	2.420536
H	6.704407	-2.602652	1.774648
H	6.418105	-2.221231	3.470866
C	1.067803	-3.525795	-0.179045
H	0.973353	-2.550445	-0.678776
C	1.659954	-4.519102	-1.179433
H	2.613064	-4.166682	-1.589925
H	0.974918	-4.680219	-2.018042
H	1.842150	-5.488501	-0.700390
C	-0.346634	-3.922520	0.219223
H	-0.995483	-3.911175	-0.661707
H	-0.756081	-3.212105	0.946873
H	-0.397840	-4.932217	0.644101
C	2.878937	2.436061	-1.348329
C	2.076039	3.070601	-2.314500
C	1.980144	4.465328	-2.290905
H	1.357924	4.964269	-3.031006
C	2.647533	5.219178	-1.337330
H	2.568444	6.302833	-1.341433
C	3.384687	4.575642	-0.351730
H	3.868814	5.164568	0.421828
C	3.499450	3.184078	-0.322619
C	1.241334	2.304814	-3.320283
H	1.556568	1.252444	-3.300189
C	-0.228415	2.360284	-2.895838
H	-0.590783	3.396272	-2.885458
H	-0.354563	1.960698	-1.880169
H	-0.870841	1.788823	-3.575060
C	1.408472	2.829679	-4.744968
H	0.821993	2.234653	-5.452022
H	2.452859	2.810033	-5.073525
H	1.059161	3.863453	-4.835594
C	4.273455	2.509112	0.794658
H	3.811774	1.523884	0.966056
C	4.178914	3.271040	2.111364
H	4.720737	4.222280	2.076774
H	4.619183	2.684768	2.921995
H	3.137686	3.480807	2.375876
C	5.731156	2.257354	0.408187
H	6.241395	3.200117	0.178132
H	5.817735	1.604918	-0.467008
H	6.273059	1.777086	1.231325
H	0.792357	-0.411563	2.637423
C	-0.792111	-0.668825	3.145972
C	-1.205539	0.401078	3.941416
C	-1.086301	-1.944308	3.656373
C	-1.905541	0.267147	5.130448
C	-1.787071	-2.120502	4.850277
C	-2.203195	-1.015833	5.591067
H	-2.005817	-3.125605	5.203839
F	-0.903678	1.660694	3.525662
H	-2.208382	1.158088	5.674178
H	-2.748277	-1.145330	6.522013
H	-0.753261	-2.822224	3.103963

TS-4_ AlPdAl _M06L.log

SCF (M06L) = -3041.25927713
E(SCF)+ZPE(0 K) = -3039.899717
H(298 K) = -3039.819398
G(298 K) = -3040.010588
Lowest Frequency = -75.1113 cm^{-1}

Pd	-0.054265000	-0.043215000	0.435650000
Al	2.151903000	0.706664000	0.672729000
N	3.019156000	2.259600000	-0.005738000
N	3.716911000	0.023730000	1.548951000
C	4.045489000	2.836326000	0.636980000
C	4.779563000	2.182057000	1.635317000
H	5.580056000	2.749420000	2.095508000
C	4.696645000	0.825167000	1.982971000
C	4.459844000	4.223302000	0.251566000
H	4.684708000	4.280403000	-0.818140000
H	5.334368000	4.548187000	0.816016000
H	3.641918000	4.930048000	0.427722000
C	5.782821000	0.255044000	2.841896000
H	5.380479000	-0.425946000	3.596936000
H	6.353974000	1.045100000	3.330953000
H	6.477338000	-0.334981000	2.232941000
C	2.464692000	2.909956000	-1.163803000
C	2.921275000	2.501730000	-2.435734000
C	2.382578000	3.142189000	-3.552994000
H	2.711139000	2.848166000	-4.545542000
C	1.414978000	4.132535000	-3.420439000
H	1.008104000	4.613827000	-4.305717000
C	0.952883000	4.486448000	-2.161895000
H	0.169837000	5.235946000	-2.062545000
C	1.462760000	3.881708000	-1.009749000
C	3.930650000	1.373584000	-2.583134000

H	3.608371000	0.577878000	-1.890302000
C	3.958936000	0.766805000	-3.980741000
H	2.959858000	0.506931000	-4.336222000
H	4.558046000	-0.149259000	-3.975522000
H	4.419711000	1.450770000	-4.703616000
C	5.357375000	1.775323000	-2.197053000
H	5.694819000	2.626303000	-2.800904000
H	6.045877000	0.944528000	-2.390752000
H	5.466096000	2.040917000	-1.143318000
C	0.877656000	4.232692000	0.343548000
H	1.487199000	3.750512000	1.118723000
C	-0.542211000	3.677927000	0.459001000
H	-0.946348000	3.822540000	1.467576000
H	-0.581873000	2.602946000	0.234668000
H	-1.212400000	4.191791000	-0.240569000
C	0.881965000	5.736794000	0.610193000
H	1.882161000	6.175891000	0.525704000
H	0.505114000	5.949938000	1.615311000
H	0.234438000	6.267496000	-0.096618000
C	3.765373000	-1.387420000	1.818982000
C	2.959783000	-1.916823000	2.846664000
C	2.919636000	-3.304944000	2.997107000
H	2.299661000	-3.730316000	3.784184000
C	3.653977000	-4.143008000	2.169027000
H	3.596718000	-5.220873000	2.294717000
C	4.475237000	-3.597206000	1.191428000
H	5.063409000	-4.253412000	0.552842000
C	4.554067000	-2.216992000	0.998454000
C	2.224419000	-1.039423000	3.843504000
H	2.302660000	0.002595000	3.511058000
C	0.738955000	-1.367825000	3.947751000
H	0.577187000	-2.397948000	4.287207000
H	0.228377000	-1.236527000	2.985864000
H	0.257221000	-0.702157000	4.671531000
C	2.885577000	-1.142755000	5.220187000
H	2.816528000	-2.164264000	5.611829000
H	2.393201000	-0.478841000	5.938008000
H	3.947528000	-0.876519000	5.191852000
C	5.470621000	-1.672913000	-0.082392000
H	5.496125000	-0.578423000	-0.000492000
C	4.958739000	-2.012244000	-1.481297000
H	4.837348000	-3.094929000	-1.602954000
H	5.664723000	-1.666411000	-2.245137000
H	3.987612000	-1.555768000	-1.685330000
C	6.901515000	-2.184588000	0.087839000
H	7.292489000	-2.002045000	1.094365000
H	7.571637000	-1.699018000	-0.629110000
H	6.962143000	-3.263791000	-0.090306000
Al	-2.442611000	-0.506974000	0.546361000
N	-3.433835000	-2.081816000	-0.083808000
N	-4.136680000	0.395135000	0.993979000
C	-4.708406000	-2.395459000	0.169265000
C	-5.583412000	-1.521430000	0.827190000
H	-6.586135000	-1.888511000	1.014285000
C	-5.338973000	-0.179744000	1.145378000
C	-5.251261000	-3.710831000	-0.302510000
H	-5.315673000	-3.730167000	-1.396017000
H	-6.245893000	-3.901060000	0.103119000
H	-4.583859000	-4.533108000	-0.026246000
C	-6.494794000	0.635819000	1.647899000
H	-6.310768000	0.990934000	2.667659000
H	-7.418933000	0.056475000	1.644567000
H	-6.636204000	1.533067000	1.036272000
C	-2.674647000	-2.922890000	-0.969534000
C	-2.799981000	-2.721835000	-2.359267000
C	-1.999188000	-3.481383000	-3.213041000
H	-2.071032000	-3.320789000	-4.287337000
C	-1.102598000	-4.417818000	-2.715582000
H	-0.480330000	-4.992267000	-3.396603000
C	-0.992902000	-4.602371000	-1.344957000
H	-0.281219000	-5.326193000	-0.952259000
C	-1.765544000	-3.860439000	-0.447734000
C	-3.742719000	-1.691111000	-2.953193000
H	-4.341015000	-1.255069000	-2.142450000
C	-2.955318000	-0.557233000	-3.606953000
H	-2.330251000	-0.936602000	-4.423420000

H	-2.285594000	-0.061592000	-2.896904000
H	-3.631890000	0.196719000	-4.026851000
C	-4.711588000	-2.310557000	-3.959086000
H	-5.423995000	-1.561269000	-4.320516000
H	-5.285246000	-3.137515000	-3.527681000
H	-4.180886000	-2.704353000	-4.833010000
C	-1.601745000	-4.097256000	1.039408000
H	-2.274713000	-3.410484000	1.569413000
C	-1.998390000	-5.523611000	1.420391000
H	-3.027051000	-5.759567000	1.127318000
H	-1.914162000	-5.675873000	2.501367000
H	-1.344612000	-6.256240000	0.933553000
C	-0.176360000	-3.795466000	1.494245000
H	-0.079733000	-3.935631000	2.576681000
H	0.112734000	-2.764510000	1.252163000
H	0.550131000	-4.456082000	1.007684000
C	-4.037570000	1.815040000	1.193159000
C	-3.930839000	2.348811000	2.490417000
C	-3.890837000	3.738332000	2.632467000
H	-3.809580000	4.166581000	3.629185000
C	-3.944714000	4.575725000	1.526993000
H	-3.923475000	5.654396000	1.657236000
C	-3.987502000	4.029581000	0.249818000
H	-3.979922000	4.688699000	-0.614162000
C	-4.011459000	2.647387000	0.053935000
C	-3.780477000	1.462812000	3.710638000
H	-4.077863000	0.444043000	3.431809000
C	-2.309342000	1.414484000	4.131126000
H	-1.960848000	2.413614000	4.419586000
H	-1.665065000	1.063466000	3.315866000
H	-2.170425000	0.749409000	4.990601000
C	-4.661003000	1.900045000	4.878408000
H	-4.593278000	1.181118000	5.700605000
H	-5.715152000	1.986870000	4.593317000
H	-4.350743000	2.871839000	5.277282000
C	-4.003421000	2.058669000	-1.345164000
H	-3.399765000	1.137136000	-1.302114000
C	-3.336123000	2.962532000	-2.374385000
H	-3.939517000	3.851838000	-2.590105000
H	-3.206199000	2.423375000	-3.317553000
H	-2.345091000	3.288901000	-2.044728000
C	-5.405455000	1.658823000	-1.807399000
H	-6.073113000	2.528625000	-1.817300000
H	-5.853131000	0.896925000	-1.161568000
H	-5.376198000	1.250598000	-2.824339000
H	0.843849000	0.961655000	1.817609000
C	0.794899000	-0.561628000	-1.432121000
C	0.387289000	0.140735000	-2.567461000
C	1.418995000	-1.767763000	-1.743880000
C	0.526381000	-0.296978000	-3.878260000
C	1.614596000	-2.268505000	-3.026444000
C	1.149936000	-1.522854000	-4.105680000
H	0.146650000	0.315556000	-4.691483000
H	2.105929000	-3.228052000	-3.159039000
H	1.279792000	-1.889918000	-5.119857000
F	1.879786000	-2.536882000	-0.713080000
F	-0.255286000	1.331017000	-2.387746000

TS-4'_AlPdPCy$_3$ _M06L.log

SCF (M06L) = -2847.09690659
E(SCF)+ZPE(0 K) = -2845.891951
H(298 K) = -2845.826583
G(298 K) = -2845.989929
Lowest Frequency = -91.7674 cm-1

Pd	0.664193	-0.316488	-0.321269
P	3.003264	-0.425383	-0.174331
C	3.905513	-1.251513	-1.580516
H	4.990839	-1.235244	-1.386069
C	3.636782	-0.531690	-2.905669
H	2.545161	-0.453703	-3.041574
H	4.015829	0.497491	-2.870451
C	4.254137	-1.265964	-4.091381
H	4.027475	-0.731847	-5.021563

H	5.350037	-1.259264	-3.992146
C	3.769135	-2.707701	-4.167478
H	4.233497	-3.228488	-5.012876
H	2.685467	-2.712245	-4.356420
C	4.050596	-3.441281	-2.862747
H	5.138962	-3.521792	-2.719830
H	3.669205	-4.468385	-2.906015
C	3.438914	-2.710027	-1.673572
H	3.661751	-3.249660	-0.744686
H	2.341610	-2.709031	-1.766455
C	3.585183	-1.404679	1.308523
H	2.938574	-2.297838	1.242389
C	5.034870	-1.898083	1.360260
H	5.716907	-1.043182	1.459359
H	5.315181	-2.407503	0.430516
C	5.232937	-2.839510	2.546902
H	4.612975	-3.735909	2.394657
H	6.272039	-3.187402	2.585942
C	4.839410	-2.175810	3.862166
H	5.525706	-1.338155	4.058638
H	4.963100	-2.875662	4.696795
C	3.411213	-1.645723	3.808381
H	3.149600	-1.134070	4.742123
H	2.708296	-2.486467	3.713198
C	3.214694	-0.709006	2.622102
H	2.179941	-0.349742	2.580480
H	3.853979	0.178098	2.754277
C	3.758375	1.277564	-0.111427
H	3.672232	1.607470	-1.160849
C	2.906037	2.255291	0.708330
H	1.856153	2.193103	0.390716
H	2.913271	1.958390	1.766620
C	3.428541	3.681443	0.576816
H	2.824589	4.360782	1.189622
H	3.299200	4.013112	-0.465762
C	4.903454	3.780082	0.949456
H	5.267674	4.806358	0.823396
H	5.022432	3.539395	2.016577
C	5.743101	2.809781	0.126537
H	6.799433	2.870567	0.413820
H	5.693911	3.097510	-0.934528
C	5.234387	1.378640	0.277486
H	5.352908	1.070495	1.326803
H	5.844143	0.687403	-0.319439
Al	-1.564373	0.165289	-0.805799
N	-3.186829	-0.792206	-1.066482
N	-2.331438	1.899331	-1.046194
C	-4.194072	-0.296696	-1.801649
C	-4.240018	1.034687	-2.234350
H	-5.075680	1.305208	-2.868933
C	-3.413448	2.087749	-1.812614
C	-5.345510	-1.188891	-2.148991
H	-5.932109	-1.424489	-1.254128
H	-4.996512	-2.148087	-2.543482
H	-6.005955	-0.716292	-2.876482
C	-3.788681	3.479396	-2.221091
H	-2.998199	3.923735	-2.834370
H	-3.895614	4.129785	-1.347581
H	-4.720127	3.489570	-2.787675
C	-3.326842	-2.101421	-0.484826
C	-2.692223	-3.201475	-1.087888
C	-2.811675	-4.448484	-0.469828
H	-2.321054	-5.308334	-0.921572
C	-3.525805	-4.603092	0.709764
H	-3.599094	-5.580196	1.179244
C	-4.146518	-3.503279	1.289346
H	-4.701580	-3.623237	2.217627
C	-4.066872	-2.237064	0.707051
C	-1.888990	-3.080091	-2.367874
H	-1.958227	-2.043642	-2.722110
C	-0.410796	-3.378555	-2.121737
H	0.014076	-2.697430	-1.373546
H	0.163993	-3.263455	-3.047973
H	-0.268150	-4.403949	-1.759476
C	-2.449763	-3.981998	-3.466883
H	-2.372656	-5.039994	-3.192503
H	-1.893423	-3.845516	-4.399436
H	-3.505327	-3.773935	-3.671368
C	-4.742666	-1.059488	1.385950
H	-4.699032	-0.195649	0.709967
C	-3.998663	-0.674925	2.664034
H	-4.466245	0.194969	3.140474
H	-2.949288	-0.434785	2.466558
H	-4.009384	-1.498023	3.387666
C	-6.214852	-1.332612	1.688308
H	-6.774091	-1.634682	0.796423
H	-6.694711	-0.437683	2.097890
H	-6.330911	-2.130492	2.429831
C	-1.640791	3.046439	-0.516873
C	-1.977915	3.477547	0.784385
C	-1.322684	4.601507	1.288050
H	-1.563079	4.955841	2.285860
C	-0.339885	5.254277	0.552460
H	0.165372	6.121180	0.969948
C	0.012960	4.780670	-0.701827
H	0.806476	5.271666	-1.262553
C	-0.627888	3.672701	-1.265416
C	-2.967995	2.701544	1.638970
H	-2.746765	1.632509	1.479816
C	-2.809848	2.975119	3.130640
H	-3.181742	3.971437	3.397153
H	-1.767462	2.902312	3.445836
H	-3.391509	2.249092	3.707381
C	-4.430346	2.927111	1.245615
H	-4.687810	3.991589	1.300903
H	-5.092491	2.394196	1.938145
H	-4.667378	2.571716	0.240016
C	-0.186693	3.188668	-2.634077
H	-0.826702	2.346575	-2.928184
C	1.252402	2.673976	-2.592632
H	1.947337	3.474479	-2.307938
H	1.557820	2.298756	-3.575660
H	1.360699	1.851349	-1.874462
C	-0.314621	4.282693	-3.694950
H	-1.326452	4.696736	-3.755602
H	-0.052676	3.893600	-4.683728
H	0.362485	5.117888	-3.483949
C	-0.332937	-0.531092	1.543098
C	-0.546844	-1.824160	2.025433
C	-0.362707	0.427880	2.554410
C	-0.774633	-2.163945	3.352490
C	-0.582920	0.174629	3.903893
C	-0.796706	-1.143409	4.300623
H	-0.921252	-3.204658	3.624629
H	-0.571035	0.991976	4.618410
H	-0.972629	-1.374973	5.346809
H	-0.270285	-0.086696	-1.987795
F	-0.107676	1.724380	2.208542
F	-0.480083	-2.852197	1.133570

TS-5_ AlPdAl _M06L.log

SCF (M06L) = -3041.2648155
E(SCF)+ZPE(0 K) = -3039.908259
H(298 K) = -3039.826536
G(298 K) = -3040.023297
Lowest Frequency = -165.0723 cm-1

Pd	0.064658000	0.076599000	0.739749000
Al	-2.230680000	0.720956000	0.272371000
N	-4.053612000	-0.020741000	0.155277000
N	-2.895835000	2.503339000	-0.236461000
C	-5.204450000	0.661303000	0.111045000
C	-5.248008000	2.058296000	0.013388000
H	-6.232669000	2.512133000	0.023648000
C	-4.168672000	2.918952000	-0.229507000
C	-6.509245000	-0.079352000	0.149059000
H	-7.352349000	0.591363000	-0.021521000
H	-6.650134000	-0.570685000	1.118072000

H	-6.530925000	-0.876862000	-0.600499000
C	-4.486394000	4.350889000	-0.548546000
H	-5.503780000	4.604313000	-0.246022000
H	-4.400389000	4.533925000	-1.625683000
H	-3.781915000	5.034406000	-0.066188000
C	-4.077519000	-1.454636000	0.099728000
C	-3.715765000	-2.075147000	-1.115278000
C	-3.711382000	-3.470566000	-1.168157000
H	-3.434765000	-3.966632000	-2.094767000
C	-4.036353000	-4.234232000	-0.053721000
C	-4.346298000	-3.606895000	1.145102000
H	-4.555869000	-4.206061000	2.028955000
C	-4.362152000	-2.214044000	1.250208000
C	-1.891093000	3.392022000	-0.751468000
C	-0.959012000	3.990316000	0.118296000
C	-0.016179000	4.867267000	-0.427511000
H	0.701044000	5.345521000	0.235825000
C	0.025871000	5.131528000	-1.788742000
C	-0.859036000	4.482152000	-2.640689000
H	-0.788336000	4.641643000	-3.715035000
C	-1.816327000	3.595405000	-2.146780000
C	0.355429000	-0.230320000	2.826446000
C	1.338832000	-0.979817000	3.277577000
C	-0.433890000	0.149946000	3.920600000
C	1.770341000	-1.499480000	4.459187000
C	-0.186542000	-0.274477000	5.226000000
C	0.903942000	-1.104851000	5.492228000
H	2.650728000	-2.111772000	4.603444000
H	-0.844563000	0.052720000	6.024667000
H	1.099128000	-1.438669000	6.507424000
H	0.994816000	-0.125869000	-0.690429000
F	-1.494590000	0.976731000	3.737743000
F	2.793878000	-1.376762000	2.008928000
C	-0.954145000	3.735723000	1.611763000
H	-1.594786000	2.868918000	1.814046000
C	-2.652910000	2.788548000	-3.123136000
H	-3.466098000	2.297795000	-2.573300000
C	-3.368853000	-1.253324000	-2.342666000
H	-2.946308000	-0.299294000	-1.992981000
C	-4.567311000	-1.568687000	2.605362000
H	-4.729636000	-0.494421000	2.453819000
Al	2.306621000	-0.839444000	0.364105000
N	2.669603000	-2.443066000	-0.638495000
N	3.929578000	0.065771000	-0.113701000
C	5.049067000	-0.627327000	-0.336547000
C	5.046595000	-2.018384000	-0.542052000
C	3.940757000	-2.847984000	-0.787505000
C	4.225626000	-4.232504000	-1.293465000
C	2.352766000	-2.410539000	-3.556782000
H	3.170334000	-1.957989000	-2.981292000
H	4.140495000	-4.278095000	-2.384530000
H	5.239613000	-4.537836000	-1.029303000
H	3.510633000	-4.957906000	-0.894593000
H	6.659664000	0.410176000	0.618577000
C	6.364790000	0.088977000	-0.386783000
H	7.153672000	-0.549218000	-0.786517000
H	6.297504000	0.998803000	-0.990602000
H	6.020215000	-2.482386000	-0.654874000
C	1.624129000	-3.221081000	-1.235835000
C	1.537847000	-3.305363000	-2.641432000
C	0.563362000	-4.135207000	-3.200767000
C	-0.334336000	-4.830757000	-2.402579000
C	-0.299412000	-4.661237000	-1.024319000
C	0.664914000	-3.854288000	-0.417216000
H	0.487111000	-4.205037000	-4.284272000
C	0.686939000	-3.674203000	1.084760000
H	-1.042019000	-5.154989000	-0.403278000
H	1.009127000	-2.644410000	1.280668000
C	3.940241000	1.499604000	-0.054789000
C	3.464881000	2.212964000	-1.175293000
C	3.030274000	1.494961000	-2.437474000
C	4.322524000	2.166215000	1.125042000
C	4.268097000	3.562765000	1.143095000
C	3.833733000	4.282000000	0.039514000
C	3.428648000	3.606603000	-1.105166000
H	3.065602000	4.171459000	-1.959620000
H	2.708440000	0.486108000	-2.148227000
C	4.706748000	1.432068000	2.393647000
H	4.768645000	0.360318000	2.170401000
H	4.555411000	4.087940000	2.052179000
H	-4.024180000	-5.319680000	-0.115169000
H	0.765346000	5.820934000	-2.189335000
H	-1.087284000	-5.471424000	-2.855633000
H	3.794506000	5.368127000	0.074140000
C	-3.288847000	-1.725889000	3.430176000
H	-3.106847000	-2.783324000	3.657787000
H	-2.417830000	-1.352607000	2.880437000
H	-3.354864000	-1.179179000	4.376725000
C	-5.772533000	-2.124922000	3.358191000
H	-5.632552000	-3.179490000	3.619302000
H	-5.925964000	-1.580645000	4.295052000
H	-6.695979000	-2.055290000	2.773090000
C	-4.620799000	-0.905708000	-3.147712000
H	-4.365620000	-0.300365000	-4.025462000
H	-5.125163000	-1.812543000	-3.501294000
H	-5.339438000	-0.333621000	-2.550654000
C	-2.298937000	-1.899635000	-3.211034000
H	-1.397508000	-2.118131000	-2.628959000
H	-2.638502000	-2.835424000	-3.669653000
H	-2.018830000	-1.227009000	-4.027975000
C	0.441631000	3.379131000	2.118504000
H	0.400510000	3.083421000	3.172251000
H	0.863662000	2.537041000	1.551559000
H	1.139258000	4.221656000	2.038470000
C	-1.533930000	4.925223000	2.375689000
H	-2.561659000	5.145193000	2.066551000
H	-1.544197000	4.727779000	3.452588000
H	-0.937591000	5.829921000	2.207254000
C	-1.780226000	1.686600000	-3.730496000
H	-1.314034000	1.065014000	-2.954756000
H	-2.367096000	1.035184000	-4.388650000
H	-0.969030000	2.122375000	-4.325861000
C	-3.287645000	3.634259000	-4.223172000
H	-3.936158000	3.018075000	-4.854404000
H	-3.893166000	4.451971000	-3.818464000
H	-2.531771000	4.081262000	-4.877956000
C	1.449936000	-1.272569000	-4.041800000
H	0.625322000	-1.667670000	-4.647525000
H	1.007862000	-0.726978000	-3.199636000
H	2.008697000	-0.561217000	-4.660912000
C	2.969061000	-3.142371000	-4.745560000
H	3.586880000	-3.992512000	-4.438083000
H	2.201605000	-3.527299000	-5.425914000
H	3.601323000	-2.463031000	-5.326411000
C	1.708968000	-4.594286000	1.747465000
H	2.716628000	-4.423880000	1.354796000
H	1.742513000	-4.419964000	2.828237000
H	1.452122000	-5.647694000	1.582883000
C	-0.684947000	-3.786097000	1.735656000
H	-1.082658000	-4.808405000	1.715399000
H	-0.620098000	-3.482474000	2.786736000
H	-1.411639000	-3.127732000	1.244312000
C	3.612240000	1.620490000	3.445617000
H	2.630681000	1.328082000	3.060320000
H	3.815736000	1.022106000	4.340199000
H	3.546826000	2.672202000	3.750226000
C	6.054898000	1.889917000	2.947592000
H	6.019685000	2.938660000	3.263181000
H	6.333592000	1.297938000	3.825025000
H	6.861402000	1.802286000	2.211952000
C	4.202666000	1.330684000	-3.405216000
H	4.606485000	2.304489000	-3.706383000
H	5.019918000	0.754658000	-2.957149000
H	3.889083000	0.803696000	-4.314086000
C	1.838938000	2.165527000	-3.108549000
H	1.000822000	2.268978000	-2.410160000
H	2.080537000	3.163139000	-3.493646000
H	1.498565000	1.569017000	-3.960672000

TS-5'_AlPdPCy$_3$ _M06L.log

SCF (M06L) = -2847.09478814
E(SCF)+ZPE(0 K) = -2845.890624
H(298 K) = -2845.824801
G(298 K) = -2845.988537
Lowest Frequency = -151.5457cm-1

Pd	0.602657	-0.244687	-0.572055
P	2.766646	0.011006	0.402949
C	3.292482	1.792398	0.268750
H	4.306318	1.910896	0.689319
C	2.329093	2.696687	1.051311
H	1.305719	2.535765	0.668628
H	2.301016	2.422061	2.113510
C	2.703190	4.166945	0.908550
H	2.003578	4.785549	1.482429
H	3.699266	4.331709	1.347852
C	2.719364	4.588062	-0.554119
H	2.999083	5.643661	-0.652944
H	1.699245	4.494561	-0.957424
C	3.663507	3.703738	-1.359833
H	4.695767	3.873780	-1.018201
H	3.642071	3.980526	-2.420519
C	3.315559	2.227379	-1.202512
H	4.013407	1.609761	-1.780994
H	2.317535	2.034516	-1.628925
C	4.212812	-0.942938	-0.277233
H	4.409166	-0.418991	-1.225914
C	5.519343	-0.964887	0.516166
H	5.363739	-1.525368	1.450132
H	5.821886	0.049514	0.807489
C	6.625002	-1.648469	-0.284815
H	6.827389	-1.058721	-1.191093
H	7.559257	-1.662691	0.288973
C	6.219676	-3.063097	-0.684363
H	6.115210	-3.673640	0.225185
H	7.009128	-3.534727	-1.280969
C	4.896095	-3.069223	-1.442091
H	4.598466	-4.095819	-1.685674
H	5.022648	-2.547943	-2.402384
C	3.795849	-2.371263	-0.650693
H	2.857426	-2.346599	-1.217949
H	3.588374	-2.941376	0.267435
C	2.691421	-0.248279	2.249723
H	1.777598	0.319728	2.493049
C	2.383148	-1.702450	2.616607
H	1.575531	-2.087666	1.977501
H	3.269013	-2.326517	2.417906
C	2.006204	-1.816766	4.090594
H	1.801439	-2.861424	4.353828
H	1.067050	-1.267163	4.256557
C	3.089255	-1.235524	4.992648
H	2.787074	-1.294906	6.044663
H	4.001147	-1.844366	4.899327
C	3.414529	0.203983	4.608649
H	4.210955	0.603284	5.247636
H	2.529839	0.835717	4.784904
C	3.811362	0.304395	3.137509
H	4.734549	-0.266361	2.979249
H	4.045856	1.343936	2.876644
Al	-1.815129	0.003654	-0.600638
N	-2.792199	1.494475	0.124368
N	-3.120446	-1.326329	-0.117516
C	-4.130213	1.493108	0.089934
C	-4.882653	0.321879	-0.097615
H	-5.958814	0.448866	-0.131853
C	-4.420329	-1.004263	-0.068473
C	-4.872723	2.781076	0.286828
H	-4.557272	3.283305	1.206892
H	-5.950667	2.620341	0.322667
H	-4.649416	3.477338	-0.529486
C	-5.447888	-2.087071	0.082286
H	-5.516334	-2.410213	1.126934
H	-5.186256	-2.974541	-0.500909
H	-6.434620	-1.731039	-0.218434
C	-2.093805	2.688383	0.511094
C	-1.638959	2.800560	1.841841
C	-0.984120	3.975402	2.218531
H	-0.631105	4.084676	3.240579
C	-0.760663	5.001068	1.307390
C	-1.161123	4.847404	-0.011901
H	-0.946495	5.630647	-0.736371
C	-1.826919	3.694420	-0.436608
C	-2.718098	-2.662926	0.214480
C	-2.203301	-3.505057	-0.794690
C	-1.834310	-4.805725	-0.448579
H	-1.443982	-5.467531	-1.216303
C	-1.945640	-5.264325	0.858631
C	-2.398360	-4.405540	1.849178
H	-2.439354	-4.746695	2.881710
C	-2.781761	-3.094743	1.553557
C	0.619148	-0.284145	-2.700111
C	1.651309	-0.521891	-3.618978
C	-0.495709	-0.146351	-3.380131
C	1.444536	-0.599877	-4.996879
C	-0.930915	-0.177709	-4.667949
C	0.160868	-0.429975	-5.517558
H	2.289394	-0.789424	-5.650531
H	-1.947773	-0.029616	-5.005342
H	-0.003954	-0.486039	-6.589803
H	-0.506621	-0.231308	0.719081
F	-2.179459	0.272105	-2.333942
F	2.921360	-0.688170	-3.178510
H	-1.656781	-6.282009	1.106497
H	-0.252977	5.909248	1.622166
C	-2.046422	-3.001865	-2.214003
C	-3.376773	-2.956624	-2.963101
C	-0.993421	-3.752413	-3.017852
H	-1.689067	-1.968464	-2.142391
H	-4.096033	-2.293705	-2.473655
H	-3.229724	-2.582313	-3.981910
H	-3.822659	-3.955970	-3.033732
H	-0.039330	-3.801462	-2.482549
H	-1.304165	-4.776219	-3.256285
H	-0.816729	-3.238059	-3.968086
C	-3.129211	-2.161037	2.696884
C	-1.849927	-1.820482	3.464174
C	-4.177462	-2.736360	3.646432
H	-3.525988	-1.224828	2.283486
H	-1.100291	-1.361571	2.808501
H	-2.058558	-1.129374	4.289238
H	-1.403574	-2.725560	3.893231
H	-5.093025	-3.035225	3.125712
H	-3.801181	-3.620155	4.173098
H	-4.451415	-1.999528	4.408483
C	-2.150493	3.522675	-1.905719
C	-0.856459	3.226720	-2.666914
C	-2.866800	4.729838	-2.504864
H	-2.802005	2.649100	-2.017760
H	-0.311831	2.387958	-2.216008
H	-1.058757	2.971433	-3.712664
H	-0.191160	4.099401	-2.652499

H	-3.785627	4.977147	-1.962247
H	-2.233010	5.623417	-2.494812
H	-3.135515	4.538112	-3.548226
C	-1.847786	1.676188	2.837665
C	-3.216715	1.755886	3.514508
C	-0.734604	1.607181	3.877059
H	-1.821408	0.737602	2.267886
H	-4.035557	1.661105	2.793953
H	-3.333369	0.949594	4.248463
H	-3.340841	2.707945	4.044097
H	0.252819	1.605509	3.401377
H	-0.768338	2.449492	4.577266
H	-0.821371	0.693869	4.473649

TS-6 _AlPdAl _M06L.log

SCF (M06L) = -3041.3190939
E(SCF)+ZPE(0 K) = -3039.957993
H(298 K) = -3039.877937
G(298 K) = -3040.067847
Lowest Frequency = -56.1964 cm^{-1}

Pd	-0.255438000	-0.109331000	0.533280000
Al	-2.907413000	0.399055000	0.307476000
N	-3.480158000	-1.053559000	-0.833012000
N	-2.947022000	1.757223000	-1.079875000
C	-4.490918000	-0.763247000	-1.666997000
C	-4.763733000	0.537870000	-2.109664000
H	-5.635218000	0.649781000	-2.744855000
C	-3.997342000	1.697404000	-1.913793000
C	-5.412680000	-1.855170000	-2.129555000
H	-4.886739000	-2.780507000	-2.372361000
H	-6.004099000	-1.543237000	-2.991842000
H	-6.104694000	-2.094048000	-1.312552000
C	-4.405539000	2.923878000	-2.677354000
H	-4.813594000	3.672409000	-1.988209000
H	-5.167205000	2.690612000	-3.422235000
H	-3.555092000	3.398391000	-3.174133000
C	-3.056499000	-2.417305000	-0.722951000
C	-2.183323000	-2.929610000	-1.708392000
C	-1.792062000	-4.265979000	-1.610099000
H	-1.113205000	-4.675965000	-2.354550000
C	-2.250466000	-5.079977000	-0.580346000
C	-3.104108000	-4.557047000	0.380075000
H	-3.460173000	-5.190328000	1.191240000
C	-3.517666000	-3.221993000	0.333919000
C	-2.150461000	2.952849000	-1.085913000
C	-2.533958000	4.060428000	-0.305132000
C	-1.744794000	5.213360000	-0.359400000
H	-2.026505000	6.072677000	0.246270000
C	-0.612666000	5.274062000	-1.158636000
C	-0.244329000	4.169251000	-1.917274000
H	0.653520000	4.215081000	-2.528657000
C	-0.998888000	2.994409000	-1.900544000
C	-0.432648000	0.341569000	2.483412000
C	0.012972000	0.500148000	3.773354000
C	-1.785198000	0.401202000	2.176316000
C	-0.873611000	0.695970000	4.824185000
C	-2.709974000	0.588368000	3.226814000
C	-2.236696000	0.733143000	4.533110000
H	-0.503918000	0.809889000	5.839491000
H	-3.775731000	0.641727000	3.036068000
H	-2.948674000	0.882931000	5.340918000
H	-0.407362000	-0.543607000	-1.015117000
F	-4.415289000	0.742479000	1.021613000
F	1.354923000	0.470020000	4.047500000
C	-3.740513000	4.027492000	0.611427000
H	-4.319888000	3.123565000	0.391967000
C	-0.599348000	1.807056000	-2.751605000
H	-0.934853000	0.912392000	-2.216290000
C	-1.700240000	-2.079551000	-2.869415000
H	-1.803582000	-1.030401000	-2.567580000
C	-4.455011000	-2.701134000	1.405082000
H	-4.693783000	-1.654049000	1.181071000
Al	2.048447000	-0.295618000	0.487742000
N	3.094784000	-1.880701000	0.709159000
N	3.529287000	0.899011000	0.717764000
C	4.644646000	0.555928000	1.386210000
C	4.937814000	-0.760864000	1.763444000
C	4.268605000	-1.921636000	1.353428000
C	4.921652000	-3.245541000	1.619498000
H	5.852275000	-0.904815000	2.327151000
H	5.850305000	-3.121874000	2.177598000
H	4.257902000	-3.913718000	2.175551000
H	5.143536000	-3.760060000	0.678787000
C	5.668697000	1.605013000	1.696297000
H	6.324527000	1.278396000	2.504527000
H	5.204542000	2.557280000	1.964395000
C	3.455758000	2.212969000	0.126779000
C	4.194049000	2.469627000	-1.048696000
C	5.047943000	1.420811000	-1.739408000
H	5.119624000	0.537352000	-1.092052000
C	4.090219000	3.734436000	-1.631741000
H	4.643801000	3.940540000	-2.545762000
C	3.291275000	4.721979000	-1.071957000
C	2.567637000	4.447636000	0.079786000
H	1.931442000	5.216104000	0.511278000
C	2.621722000	3.195552000	0.697696000
C	1.828860000	2.954650000	1.964964000
H	6.294315000	1.800197000	0.818355000
H	1.762884000	1.875475000	2.128380000
C	3.605374000	-2.402228000	-2.054187000
H	3.241739000	-1.394779000	-1.785347000
H	2.598360000	-4.837176000	-2.753915000
C	2.388394000	-4.596590000	-1.715850000
C	2.846743000	-3.392917000	-1.181769000
C	2.556665000	-3.105093000	0.174232000
C	1.756489000	-3.956848000	0.956901000
C	1.212739000	-3.712048000	2.363448000
H	1.324128000	-4.679076000	2.879174000
C	1.332539000	-5.154820000	0.364147000
C	1.650196000	-5.484544000	-0.941848000
H	0.721113000	-5.831939000	0.958256000
H	-0.009572000	6.179122000	-1.187379000
H	-1.934994000	-6.119648000	-0.526684000
H	1.302491000	-6.422272000	-1.367881000
H	3.223902000	5.701657000	-1.538611000
C	-4.654390000	5.236213000	0.419384000
H	-4.967036000	5.360439000	-0.622952000
H	-5.556429000	5.134349000	1.030483000
H	-4.162496000	6.167191000	0.723018000
C	-3.288616000	3.931861000	2.068673000
H	-4.147344000	3.822698000	2.739553000
H	-2.630111000	3.073553000	2.231017000
H	-2.737119000	4.833119000	2.364838000
C	0.909844000	1.679593000	-2.910200000
H	1.420657000	1.771708000	-1.943521000
H	1.157367000	0.697074000	-3.330874000
H	1.333591000	2.436671000	-3.582510000
C	-1.281922000	1.808104000	-4.120398000
H	-0.905236000	0.980848000	-4.733285000
H	-2.366061000	1.679627000	-4.040880000
H	-1.088862000	2.739968000	-4.666337000
C	-3.790951000	-2.746359000	2.780749000
H	-3.557733000	-3.778416000	3.070925000
H	-2.857440000	-2.176077000	2.797169000
H	-4.451339000	-2.325492000	3.546460000
C	-5.768370000	-3.483093000	1.426721000
H	-5.602756000	-4.529833000	1.707254000
H	-6.462694000	-3.054502000	2.156215000
H	-6.263046000	-3.483666000	0.450092000
C	-2.540667000	-2.288462000	-4.131359000
H	-2.123178000	-1.715112000	-4.966450000
H	-2.552883000	-3.344580000	-4.427160000
H	-3.577003000	-1.965080000	-4.003393000
C	-0.228523000	-2.322930000	-3.186004000
H	0.388912000	-2.243658000	-2.284922000

H	-0.061504000	-3.312873000	-3.629774000
H	0.126449000	-1.582058000	-3.912534000
C	3.340256000	-2.586413000	-3.544497000
H	3.759560000	-1.744350000	-4.103462000
H	3.819811000	-3.493955000	-3.928070000
H	2.273574000	-2.646496000	-3.772225000
C	5.118319000	-2.416251000	-1.815780000
H	5.400540000	-2.070096000	-0.818928000
H	5.519185000	-3.427430000	-1.953180000
H	5.622836000	-1.763895000	-2.537687000
C	4.390116000	0.981280000	-3.047590000
H	4.270689000	1.832203000	-3.728008000
H	4.999624000	0.228948000	-3.560893000
H	3.394229000	0.559891000	-2.880265000
C	6.469106000	1.910808000	-2.014791000
H	6.964234000	2.287829000	-1.114392000
H	7.082260000	1.099794000	-2.421108000
H	6.476553000	2.722198000	-2.750477000
C	2.538178000	3.545247000	3.183275000
H	1.963006000	3.341608000	4.092009000
H	3.535998000	3.115747000	3.324536000
H	2.652000000	4.631787000	3.089182000
C	0.398280000	3.472356000	1.861807000
H	-0.121179000	3.040532000	0.997015000
H	-0.167181000	3.196096000	2.758051000
H	0.354123000	4.563825000	1.768673000
C	1.845910000	-2.660022000	3.266141000
H	1.381636000	-2.727457000	4.255437000
H	2.922851000	-2.778669000	3.408520000
H	1.654425000	-1.643534000	2.915456000
C	-0.291037000	-3.430192000	2.260740000
H	-0.834113000	-4.249376000	1.782086000
H	-0.719469000	-3.263535000	3.255502000
H	-0.474750000	-2.525337000	1.663142000

TS-6'_AlPdPCy$_3$ _M06L.log

SCF (M06L) = -2847.13771534
E(SCF)+ZPE(0 K) = -2845.929572
H(298 K) = -2845.865180
G(298 K) = -2846.024410
Lowest Frequency = -42.2447 cm^{-1}

Pd	0.754829	-0.773529	-0.331969
P	2.431148	0.627404	0.075002
C	2.209097	2.411967	-0.383673
H	3.167664	2.890911	-0.116938
C	1.099568	3.075998	0.438119
H	0.134299	2.619781	0.163783
H	1.229351	2.889644	1.510792
C	1.061738	4.576896	0.171387
H	0.274032	5.041276	0.774070
H	2.014443	5.021302	0.500078
C	0.850318	4.865759	-1.308442
H	0.856957	5.945574	-1.496644
H	-0.147964	4.505469	-1.598229
C	1.905359	4.166820	-2.158681
H	2.890764	4.611505	-1.951772
H	1.711480	4.330189	-3.224727
C	1.954283	2.668708	-1.870374
H	2.711803	2.185283	-2.498064
H	0.993708	2.214325	-2.144610
C	3.920786	0.084075	-0.885798
H	3.678911	0.425595	-1.900419
C	5.251713	0.722634	-0.478063
H	5.527814	0.392746	0.533798
H	5.166336	1.816535	-0.437153
C	6.357871	0.312508	-1.447301
H	6.125017	0.710579	-2.445578
H	7.308424	0.767652	-1.146122
C	6.485956	-1.204085	-1.527009
H	6.800351	-1.589051	-0.545090
H	7.274788	-1.484610	-2.234050
C	5.160014	-1.846734	-1.917484
H	5.254171	-2.938236	-1.945516
H	4.889626	-1.533097	-2.935731
C	4.038772	-1.443181	-0.966865
H	3.078550	-1.862930	-1.294387
H	4.234162	-1.857220	0.033086
C	2.872590	0.713400	1.888738
H	1.876706	0.819191	2.346466
C	3.451636	-0.601830	2.420531
H	2.846757	-1.446628	2.067140
H	4.468052	-0.743220	2.022865
C	3.511270	-0.582280	3.944092
H	3.920733	-1.527570	4.318954
H	2.485386	-0.512538	4.337557
C	4.328620	0.599383	4.450924
H	4.339651	0.623162	5.546460
H	5.374952	0.474888	4.134276
C	3.792663	1.912136	3.894016
H	4.405219	2.755631	4.232310
H	2.780510	2.086777	4.289595
C	3.727481	1.895449	2.367225
H	4.744875	1.814739	1.962793
H	3.328566	2.851133	2.009734
Al	-2.516730	-0.483141	-0.984225
N	-3.121500	0.607063	0.475200
N	-2.365827	-2.067710	0.149477
C	-4.209279	0.076032	1.066679
C	-4.438504	-1.306142	1.103077
H	-5.386748	-1.630392	1.518365
C	-3.510923	-2.318469	0.805636
C	-5.224794	0.979842	1.700259
H	-4.773037	1.659997	2.426681
H	-6.011275	0.409161	2.195619
H	-5.681692	1.612729	0.930856
C	-3.877387	-3.711629	1.223992
H	-4.110772	-3.766010	2.290623
H	-3.094755	-4.436965	0.995456
H	-4.784221	-4.006344	0.684953
C	-2.779102	1.961112	0.777268
C	-2.127178	2.235798	2.004001
C	-1.909681	3.572354	2.344284
H	-1.424525	3.810715	3.286849
C	-2.277287	4.607467	1.490702
C	-2.826855	4.312390	0.253093
H	-3.075638	5.118314	-0.434901
C	-3.086500	2.991886	-0.129318
C	-1.241585	-2.943679	0.219901
C	-0.678688	-3.407427	-1.001805
C	0.517158	-4.132015	-0.955650
H	0.969275	-4.459910	-1.886963
C	1.099706	-4.486007	0.254741
C	0.488175	-4.111665	1.441069
H	0.938672	-4.397892	2.389326
C	-0.659801	-3.316109	1.461964
C	0.227855	-0.235818	-2.274087
C	0.946298	-0.096180	-3.453070
C	-1.166209	-0.020970	-2.349671
C	0.399042	0.260015	-4.675561
C	-1.752068	0.309510	-3.593768
C	-0.976598	0.475103	-4.737533
H	1.035296	0.351865	-5.551778
H	-2.831524	0.436913	-3.665741

H	-1.437552	0.755643	-5.681821
H	0.971363	-1.130944	1.230189
F	-3.938653	-0.769097	-1.851963
F	2.301626	-0.331046	-3.440990
H	2.018759	-5.065438	0.269660
H	-2.103808	5.640444	1.781136
C	-1.460061	-3.317279	-2.298660
C	-2.577580	-4.363096	-2.288714
C	-0.604636	-3.443461	-3.550216
H	-1.955151	-2.343879	-2.362494
H	-3.265749	-4.206612	-1.452389
H	-3.162666	-4.305572	-3.211495
H	-2.167200	-5.376360	-2.207755
H	0.243538	-2.752400	-3.526555
H	-0.220068	-4.461472	-3.681667
H	-1.202830	-3.205995	-4.434258
C	-1.184504	-2.893320	2.824942
C	-0.138559	-2.107888	3.618733
C	-1.610221	-4.108263	3.655915
H	-2.053203	-2.236306	2.689297
H	0.203944	-1.227075	3.068979
H	-0.546311	-1.793257	4.586570
H	0.743667	-2.726210	3.823342
H	-2.298964	-4.770434	3.126493
H	-0.734827	-4.707131	3.931307
H	-2.090537	-3.790184	4.587248
C	-3.686451	2.736633	-1.497775
C	-2.794743	3.314123	-2.596203
C	-5.101210	3.304559	-1.605435
H	-3.766158	1.654184	-1.660064
H	-1.777528	2.913150	-2.539557
H	-3.189848	3.067092	-3.586766
H	-2.737198	4.406774	-2.526032
H	-5.771197	2.880286	-0.851121
H	-5.100267	4.392422	-1.470587
H	-5.530411	3.091313	-2.589084
C	-1.612255	1.122071	2.906387
C	-2.660754	0.551422	3.868222
C	-0.411784	1.568567	3.739729
H	-1.283355	0.306925	2.239766
H	-3.445563	-0.018344	3.367452
H	-2.180573	-0.126354	4.582943
H	-3.133883	1.353831	4.447419
H	0.335020	2.099190	3.142135
H	-0.713499	2.235198	4.556135
H	0.072921	0.701917	4.200986

TS-7 _AlPdAl _M06L.log

SCF (M06L) = -3041.31492869
E(SCF)+ZPE(0 K) = -3039.956113
H(298 K) = -3039.874455
G(298 K) = -3040.072072
Lowest Frequency = -735.3878 cm^{-1}

Pd	-0.595648000	-0.498319000	-0.317609000
Al	2.525410000	0.002326000	0.330401000
N	3.936531000	1.281531000	0.465903000
N	3.392820000	-1.309562000	1.428099000
C	4.626365000	1.390788000	1.616065000
C	4.745651000	0.340200000	2.530653000
H	5.351696000	0.532338000	3.409036000
C	4.250356000	-0.969550000	2.391522000
C	5.288527000	2.695746000	1.948740000
H	4.517134000	3.451733000	2.138758000
H	5.891400000	3.078915000	1.121900000
H	5.915457000	2.611473000	2.837406000
C	4.717369000	-1.995381000	3.380430000
H	5.682654000	-1.717229000	3.806548000
H	4.794944000	-2.986213000	2.925623000
H	4.002337000	-2.082107000	4.206080000
C	4.132555000	2.276884000	-0.547310000
C	5.217374000	2.113885000	-1.436269000
C	5.405921000	3.085317000	-2.420929000
H	6.234812000	2.984688000	-3.117222000
C	4.539100000	4.166560000	-2.540220000
C	3.457086000	4.286292000	-1.678987000
H	2.769141000	5.121909000	-1.788997000
C	3.231965000	3.350236000	-0.665752000
C	2.903044000	-2.651338000	1.299514000
C	1.904916000	-3.127343000	2.170047000
C	1.382036000	-4.401015000	1.927015000
H	0.604003000	-4.784044000	2.584801000
C	1.821531000	-5.170311000	0.859238000
C	2.806683000	-4.679372000	0.008819000
H	3.141631000	-5.284426000	-0.828993000
C	3.363934000	-3.416126000	0.206922000
F	1.246710000	0.585969000	1.279999000
C	0.742005000	-1.043325000	-1.784974000
C	2.027650000	-0.516888000	-1.501389000
C	0.467606000	-1.447352000	-3.091070000
C	2.926652000	-0.356301000	-2.573334000
C	1.369222000	-1.312830000	-4.137242000
C	2.613007000	-0.746312000	-3.871691000
H	3.906110000	0.077222000	-2.384535000
H	1.087352000	-1.642427000	-5.133021000
H	3.334681000	-0.621710000	-4.676114000
F	-0.740196000	-1.992042000	-3.385043000
H	-0.619922000	-1.875023000	-1.054620000
H	1.393377000	-6.153726000	0.684459000
H	4.701466000	4.908322000	-3.317505000
C	6.130458000	0.898653000	-1.354996000
C	7.341280000	1.116291000	-0.444908000
C	6.605626000	0.428146000	-2.728187000
H	5.540010000	0.084250000	-0.909199000
H	7.058604000	1.255981000	0.601257000
H	8.008437000	0.248466000	-0.486014000
H	7.917207000	1.993909000	-0.761520000
H	5.779668000	0.320481000	-3.438303000
H	7.331287000	1.121958000	-3.166875000
H	7.105435000	-0.542057000	-2.642639000
C	2.026453000	3.477942000	0.244613000
C	0.747307000	3.046109000	-0.478457000
C	1.866998000	4.883712000	0.816544000
H	2.162615000	2.791519000	1.087602000
H	0.852414000	2.065986000	-0.960767000
H	-0.093530000	2.981076000	0.223448000
H	0.481893000	3.768982000	-1.260491000
H	2.772581000	5.223575000	1.330380000
H	1.636702000	5.616909000	0.034599000
H	1.036542000	4.908437000	1.529885000
C	4.451425000	-2.879027000	-0.703581000
C	4.414310000	-3.468836000	-2.106319000
C	5.836830000	-3.040224000	-0.075843000
H	4.276079000	-1.801432000	-0.814653000
H	3.413398000	-3.396914000	-2.542755000
H	5.100648000	-2.921033000	-2.761103000
H	4.723170000	-4.520593000	-2.119905000
H	5.925895000	-2.487930000	0.865734000
H	6.058417000	-4.093993000	0.129295000
H	6.612915000	-2.661405000	-0.750783000
C	1.354980000	-2.306604000	3.321091000
C	1.427949000	-3.070674000	4.643497000
C	-0.081757000	-1.869039000	3.045509000
H	1.953812000	-1.393420000	3.416669000
H	2.433829000	-3.448811000	4.855331000
H	1.122407000	-2.430553000	5.477276000
H	0.753913000	-3.934914000	4.637188000
H	-0.168106000	-1.310293000	2.105619000
H	-0.743002000	-2.739362000	2.976105000
H	-0.453271000	-1.227546000	3.852686000
Al	-2.799602000	0.119040000	0.488913000

N	-4.433735000	-0.923213000	0.179974000
C	-5.686557000	-0.561056000	0.477432000
C	-5.996187000	0.666401000	1.082122000
C	-5.143605000	1.766737000	1.223625000
N	-3.843699000	1.721696000	0.891974000
C	-5.729496000	3.048196000	1.739358000
H	-5.581557000	3.866200000	1.026615000
H	-5.225670000	3.357550000	2.661911000
H	-6.796341000	2.944945000	1.941399000
H	-7.032059000	0.818947000	1.363527000
C	-6.831939000	-1.458847000	0.112516000
H	-6.593837000	-2.513203000	0.274554000
H	-7.063174000	-1.353940000	-0.953980000
H	-7.730456000	-1.196790000	0.673991000
C	-4.224265000	-2.105617000	-0.613547000
C	-4.432621000	-2.038903000	-2.003853000
C	-4.176305000	-3.178939000	-2.768496000
C	-3.720696000	-4.348153000	-2.180067000
C	-3.491911000	-4.387227000	-0.809996000
C	-2.488406000	-4.443936000	1.877172000
C	-3.724398000	-3.273370000	-0.000977000
C	-3.433119000	-3.314347000	1.487777000
H	-3.106972000	-5.299247000	-0.364972000
H	-3.523348000	-5.225834000	-2.789080000
H	-4.317166000	-3.135703000	-3.846376000
H	-2.922687000	-2.368242000	1.737116000
H	-1.562925000	-4.415488000	1.292249000
H	-2.224176000	-4.366388000	2.937088000
H	-2.952084000	-5.427460000	1.739209000
C	-4.708856000	-3.389226000	2.327947000
H	-5.348558000	-2.513418000	2.192785000
H	-5.294209000	-4.278842000	2.066624000
H	-4.465094000	-3.454928000	3.393501000
C	-3.624892000	-0.190433000	-3.462528000
H	-3.883848000	0.755379000	-3.952163000
H	-2.770580000	-0.020017000	-2.794934000
H	-3.286466000	-0.888741000	-4.235271000
C	-4.829653000	-0.756347000	-2.709004000
H	-5.127978000	-0.015240000	-1.955020000
C	-6.011005000	-0.946367000	-3.658094000
H	-6.319607000	0.013101000	-4.085848000
H	-5.751519000	-1.603779000	-4.494818000
H	-6.879969000	-1.386284000	-3.157208000
C	-3.116142000	2.961066000	0.839834000
C	-2.522539000	3.494833000	1.997791000
C	-1.881657000	4.733087000	1.901709000
C	-1.817812000	5.418718000	0.696624000
C	-2.347008000	4.843010000	-0.451587000
C	-2.981533000	3.599565000	-0.411696000
H	-2.244956000	5.358650000	-1.402442000
C	-3.491770000	2.949522000	-1.684999000
C	-4.970218000	3.246492000	-1.936735000
H	-5.609113000	2.868911000	-1.132000000
H	-5.304984000	2.776107000	-2.868580000
H	-5.142587000	4.325516000	-2.027362000
H	-3.401706000	1.860686000	-1.549900000
C	-2.651187000	3.309234000	-2.904298000
H	-1.590442000	3.099542000	-2.734709000
H	-2.971724000	2.724567000	-3.771069000
H	-2.752016000	4.364956000	-3.178635000
H	-1.323988000	6.385538000	0.644328000
H	-1.418797000	5.159451000	2.789558000
C	-1.087518000	2.125929000	3.490597000
C	-2.482227000	2.730327000	3.304487000
H	-3.200007000	1.902483000	3.236779000
H	-0.809965000	1.470138000	2.657834000
H	-1.031647000	1.546061000	4.418389000
H	-0.329989000	2.918010000	3.544948000
H	-2.144300000	4.384796000	4.686807000
C	-2.870885000	3.584860000	4.507934000
H	-2.906302000	2.975880000	5.416396000
H	-3.851207000	4.056797000	4.380931000

TS-7'_AlPdPCy3 _M06L.log

SCF (M06L) = -2847.15020374
E(SCF)+ZPE(0 K) = -2845.945104
H(298 K) = -2845.879028
G(298 K) = -2846.043862
Lowest Frequency = -535.8709 cm^{-1}

Pd	1.286863	-0.148772	-1.376142
Al	-1.635740	0.080049	0.193318
N	-2.781687	-1.174324	1.069747
N	-2.427741	1.633623	0.992744
C	-3.070682	-0.945664	2.362228
C	-3.062611	0.338870	2.920600
H	-3.323984	0.405987	3.970659
C	-2.875084	1.561982	2.250509
C	-3.395380	-2.102364	3.260724
H	-4.094025	-2.804658	2.800067
H	-3.801145	-1.769022	4.216863
H	-2.474084	-2.665316	3.457231
C	-3.205572	2.821151	2.993680
H	-3.777868	2.609435	3.897491
H	-3.771637	3.513773	2.363917
H	-2.290130	3.347648	3.284462
C	-3.143250	-2.431162	0.483363
C	-4.462879	-2.583172	0.004920
C	-4.824604	-3.811217	-0.551081
H	-5.838280	-3.950997	-0.918540
C	-3.907464	-4.850272	-0.661130
C	-2.601160	-4.663342	-0.232578
H	-1.874974	-5.465726	-0.347445
C	-2.192338	-3.457492	0.343920
C	-2.322282	2.900684	0.328355
C	-1.235189	3.755132	0.589750
C	-1.163158	4.957785	-0.120248
H	-0.326297	5.628230	0.064543
C	-2.123941	5.302036	-1.059441
C	-3.175917	4.431865	-1.323057
H	-3.913886	4.694632	-2.075949
C	-3.291206	3.217294	-0.646875
F	-0.178222	-0.043662	1.045291
C	-0.406444	-0.047038	-2.494750
C	-1.611748	-0.132828	-1.755501
C	-0.461957	-0.227355	-3.875086
C	-2.788323	-0.484276	-2.444450
C	-1.631975	-0.539341	-4.554052
C	-2.808933	-0.676651	-3.823167
H	-3.717657	-0.610005	-1.888648
H	-1.604353	-0.679449	-5.630717
H	-3.736778	-0.929486	-4.331835
F	0.672608	-0.124942	-4.608340
P	3.242913	-0.135100	-0.001089
C	2.759443	-0.063327	1.797157
C	2.179474	-1.403562	2.259357
C	3.731112	0.499296	2.836684
H	1.903925	0.625095	1.758365
C	1.473061	-1.251155	3.601600
H	2.983063	-2.151959	2.350344
H	1.477169	-1.774662	1.505559
C	3.016927	0.652570	4.179055
H	4.600500	-0.163796	2.957487
H	4.125970	1.472289	2.516778
C	2.411578	-0.665422	4.650935
H	1.075626	-2.218480	3.935418
H	0.606810	-0.588194	3.461648
H	3.704347	1.053973	4.932818
H	2.212810	1.396709	4.063583
H	1.888556	-0.525653	5.604036
H	3.224182	-1.380960	4.848523
C	4.329372	1.362136	-0.240455
C	5.791060	1.255228	0.204611
C	4.260231	1.912581	-1.669782
H	3.836792	2.107504	0.408053
C	6.484319	2.612095	0.123173
H	6.312765	0.549619	-0.459358
H	5.872655	0.844815	1.216802
C	4.962133	3.262472	-1.767734

H	4.733873	1.195326	-2.357762
H	3.214668	1.987419	-1.989332
C	6.406074	3.188111	-1.285158
H	7.527343	2.524353	0.448951
H	5.997632	3.303053	0.828468
H	4.917427	3.637911	-2.796227
H	4.413057	3.990189	-1.150439
H	6.879637	4.175739	-1.324933
H	6.980382	2.543155	-1.966628
C	4.250733	-1.689168	-0.203044
C	4.714256	-1.842265	-1.656831
C	5.393369	-2.012931	0.765209
H	3.476213	-2.460591	-0.038141
C	5.267552	-3.239874	-1.912496
H	5.497071	-1.095718	-1.864926
H	3.884842	-1.617186	-2.340776
C	5.914985	-3.425632	0.512675
H	6.217121	-1.302498	0.628777
H	5.064265	-1.909545	1.805534
C	6.378959	-3.591522	-0.930002
H	5.623494	-3.324343	-2.945405
H	4.448931	-3.967498	-1.808300
H	6.728515	-3.659242	1.209201
H	5.111421	-4.147986	0.722284
H	6.738203	-4.612111	-1.104312
H	7.238818	-2.928087	-1.106595
H	1.041048	1.097501	-2.294289
H	-2.047023	6.241899	-1.598908
H	-4.208227	-5.797726	-1.099575
C	-5.460278	-1.437945	0.060612
C	-6.320953	-1.466588	1.325258
C	-6.357927	-1.385549	-1.173835
H	-4.878004	-0.506030	0.089563
H	-5.730007	-1.320529	2.233347
H	-7.074148	-0.671823	1.296272
H	-6.849260	-2.422884	1.416564
H	-5.781996	-1.430595	-2.104090
H	-7.077858	-2.211108	-1.190679
H	-6.940324	-0.457956	-1.180023
C	-0.742361	-3.273791	0.745660
C	0.143255	-3.215051	-0.503015
C	-0.252975	-4.362111	1.697699
H	-0.650068	-2.311497	1.263981
H	-0.213514	-2.467337	-1.220596
H	1.182229	-2.966589	-0.242089
H	0.158936	-4.180662	-1.022376
H	-0.856882	-4.411359	2.609761
H	-0.285419	-5.351925	1.228674
H	0.786181	-4.175631	1.991225
C	-4.428894	2.258920	-0.936554
C	-4.964278	2.366271	-2.358201
C	-5.560467	2.396664	0.083279
H	-4.021591	1.245892	-0.827013
H	-4.157710	2.312209	-3.095229
H	-5.657686	1.543185	-2.561545
H	-5.516231	3.298852	-2.521655
H	-5.232162	2.146827	1.097837
H	-5.955942	3.418885	0.096991
H	-6.388841	1.723302	-0.165992
C	-0.122793	3.408208	1.562311
C	0.118667	4.523325	2.580035
C	1.172810	3.102418	0.809158
H	-0.401402	2.496758	2.107605
H	-0.796105	4.820238	3.103542
H	0.850378	4.207085	3.330539
H	0.518624	5.422172	2.098101
H	1.070419	2.225858	0.155398
H	1.477138	3.952424	0.185932
H	1.986131	2.904434	1.518897

[Pd(1)$_2$]_B3PW91.log

SCF (B3PW91) = -2609.94111879
E(SCF)+ZPE(0 K) = -2608.662842
H(298 K) = -2608.585690
G(298 K) = -2608.786861
Lowest Frequency = 4.9502 cm^{-1}

Pd	0.000068	-0.001386	0.003624
Al	-2.402377	-0.048825	0.006111
N	-3.836877	1.230674	0.525063
N	-3.762382	-1.410767	-0.503156
C	-5.160715	1.039294	0.475175
C	-5.746235	-0.154004	0.033174
H	-6.828639	-0.189266	0.045144
C	-5.094225	-1.305989	-0.425671
C	-6.093472	2.141792	0.910569
H	-5.916027	2.410419	1.956105
H	-7.136213	1.841181	0.796818
H	-5.921061	3.049735	0.325314
C	-5.962655	-2.464186	-0.849365
H	-5.805512	-2.700128	-1.906132
H	-7.019094	-2.239971	-0.693408
H	-5.705350	-3.368118	-0.289864
C	-3.346731	2.498481	1.003872
C	-3.086441	2.667164	2.381527
C	-2.585894	3.897965	2.816317
H	-2.378881	4.043010	3.873323
C	-2.341841	4.934773	1.923847
H	-1.956047	5.885216	2.283000
C	-2.587023	4.746823	0.569324
H	-2.380941	5.554023	-0.128572
C	-3.087659	3.535747	0.081873
C	-3.300753	1.551163	3.393849
H	-3.812050	0.729255	2.881786
C	-1.953510	1.012146	3.898364
H	-1.329927	0.668291	3.066040
H	-2.107788	0.173219	4.586678
H	-1.398021	1.789568	4.435060
C	-4.189950	1.982089	4.568690
H	-3.714687	2.763593	5.171356
H	-4.380874	1.130872	5.231206
H	-5.157103	2.368730	4.230610
C	-3.307578	3.370540	-1.414746
H	-3.800670	2.405816	-1.574723
C	-1.965148	3.332744	-2.160313
H	-2.126069	3.161420	-3.231020
H	-1.320097	2.536348	-1.773985
H	-1.425624	4.279532	-2.048668
C	-4.226325	4.454556	-1.996105
H	-5.193033	4.491225	-1.482833
H	-4.416166	4.261006	-3.057490
H	-3.775023	5.449922	-1.921754
C	-3.200723	-2.639873	-1.003892
C	-2.971343	-2.784454	-2.389788
C	-2.400932	-3.975880	-2.847272
H	-2.216207	-4.101830	-3.910816
C	-2.059203	-4.997153	-1.968922
H	-1.620385	-5.917382	-2.345467
C	-2.273933	-4.831497	-0.606452
H	-1.990623	-5.624850	0.080157
C	-2.840547	-3.659362	-0.096298
C	-3.287046	-1.678317	-3.386277
H	-3.851529	-0.903150	-2.857442
C	-1.991816	-1.032938	-3.902575
H	-1.387255	-1.760490	-4.455897
H	-1.383189	-0.653125	-3.074735
H	-2.218430	-0.200206	-4.578288
C	-4.158268	-2.162634	-4.553548
H	-3.633027	-2.895728	-5.175208
H	-4.426557	-1.320424	-5.200571
H	-5.086196	-2.629130	-4.206332
C	-3.021971	-3.515558	1.407532
H	-3.560701	-2.579647	1.590228
C	-1.661106	-3.411094	2.112060
H	-1.076605	-4.328047	1.979987
H	-1.798632	-3.252359	3.187984
H	-1.070334	-2.580215	1.710823
C	-3.863890	-4.651011	2.006955

H	-4.844403	-4.732268	1.526004
H	-4.027227	-4.478034	3.076294
H	-3.364852	-5.620916	1.905551
Al	2.402458	0.048617	0.004936
N	3.837955	-1.230323	0.522554
N	3.761411	1.411052	-0.505689
C	5.161669	-1.038629	0.470968
C	5.746300	0.154776	0.028063
H	6.828711	0.190307	0.038570
C	5.093392	1.306561	-0.429987
C	6.095264	-2.140815	0.905360
H	5.919842	-2.408614	1.951455
H	7.137786	-1.840280	0.789404
H	5.921718	-3.049210	0.321154
C	5.960995	2.464878	-0.855056
H	5.802748	2.700208	-1.911802
H	7.017664	2.241099	-0.700038
H	5.703927	3.369013	-0.295788
C	3.348656	-2.498193	1.002037
C	3.090687	-2.667084	2.380107
C	2.590866	-3.897938	2.815564
H	2.385622	-4.043137	3.872893
C	2.345284	-4.934613	1.923352
H	1.960083	-5.885102	2.283019
C	2.588148	-4.746453	0.568445
H	2.380865	-5.553539	-0.129231
C	3.087949	-3.535298	0.080318
C	3.306532	-1.551143	3.392168
H	3.817366	-0.729341	2.879472
C	1.959998	-1.011861	3.898309
H	1.405062	-1.789176	4.435731
H	1.335409	-0.668007	3.066745
H	2.115238	-0.172906	4.586372
C	4.197044	-1.982213	4.565953
H	4.389082	-1.130966	5.228110
H	5.163641	-2.369241	4.226727
H	3.722271	-2.763457	5.169340
C	3.305320	-3.369923	-1.416652
H	3.797510	-2.404866	-1.577414
C	4.223916	-4.453324	-1.999411
H	5.191525	-4.489145	-1.487782
H	4.411797	-4.259817	-3.061152
H	3.773503	-5.449021	-1.924088
C	1.961696	-3.333049	-2.160105
H	2.120843	-3.161594	-3.231056
H	1.316718	-2.537068	-1.772769
H	1.422999	-4.280216	-2.047677
C	3.198860	2.640061	-1.005675
C	2.967327	2.784527	-2.391215
C	2.396188	3.975913	-2.847904
H	2.209831	4.101778	-3.911172
C	2.055778	4.997233	-1.969103
H	1.616357	5.917426	-2.345035
C	2.272598	4.831677	-0.606948
H	1.990300	5.625063	0.080039
C	2.840062	3.659609	-0.097597
C	3.281486	1.678324	-3.388109
H	3.846732	0.903165	-2.860072
C	1.985440	1.032973	-3.902391
H	1.380096	1.760506	-4.454883
H	1.378046	0.653284	-3.073579
H	2.210988	0.200156	-4.578357
C	4.150972	2.162541	-4.556712
H	4.418230	1.320288	-5.204105
H	5.079451	2.628997	-4.210915
H	3.624834	2.895643	-5.177603
C	3.023791	3.515864	1.405961
H	3.563068	2.580114	1.587847
C	1.663992	3.410974	2.112473
H	1.079054	4.327778	1.981327
H	1.803116	3.252164	3.188180
H	1.072853	2.580011	1.711990
C	3.866255	4.651558	2.004154
H	3.366806	5.621324	1.903478
H	4.846045	4.733080	1.521777
H	4.031201	4.478628	3.073254

[Pd(1)$_2$]_WB97X.log

SCF (wB97x) = -2610.19144565
E(SCF)+ZPE(0 K) = -2608.896051
H(298 K) = -2608.821704
G(298 K) = -2609.007427
Lowest Frequency = 10.0653 cm^{-1}

Pd	0.016186	0.042387	-0.966687
Al	2.270742	0.024142	-0.160596
N	3.625302	1.471023	-0.204617
N	3.331959	-0.934451	1.226850
C	4.545152	1.737066	0.719805
C	4.836920	0.865243	1.779718
H	5.607978	1.183104	2.470970
C	4.328266	-0.426095	1.957663
C	5.343490	3.017657	0.646101
H	5.808188	3.137668	-0.336140
H	6.117901	3.038513	1.414348
H	4.682199	3.877545	0.790292
C	4.987328	-1.277196	3.017397
H	4.252086	-1.653688	3.733579
H	5.749350	-0.709991	3.553394
H	5.457191	-2.153994	2.559816
C	3.552433	2.331715	-1.359695
C	4.251407	1.940641	-2.521832
C	4.233102	2.796600	-3.622393
H	4.773285	2.523778	-4.524235
C	3.518330	3.987731	-3.594637
H	3.516954	4.642106	-4.462429
C	2.781644	4.320400	-2.469307
H	2.187830	5.231644	-2.467911
C	2.774320	3.501223	-1.337584
C	4.997125	0.610884	-2.592752
H	4.446525	-0.101713	-1.965353
C	5.025351	0.018645	-4.004284
H	4.022650	-0.022397	-4.440536
H	5.425341	-1.000360	-3.970594
H	5.671288	0.593660	-4.677355
C	6.422257	0.705589	-2.032418
H	7.001883	1.460317	-2.577210
H	6.937289	-0.256485	-2.138762
H	6.430857	0.967016	-0.970152
C	1.876850	3.874007	-0.165881
H	2.068638	3.167939	0.650725
C	0.403107	3.729107	-0.569632
H	-0.254359	3.979336	0.270134
H	0.177436	2.701099	-0.882551
H	0.161257	4.404053	-1.399858
C	2.147041	5.287465	0.364465
H	3.194008	5.433845	0.652366
H	1.522437	5.481971	1.243500
H	1.901750	6.048773	-0.384471
C	3.003237	-2.326691	1.415718
C	2.054750	-2.712068	2.381473
C	1.763518	-4.070447	2.523953
H	1.034060	-4.381613	3.269011
C	2.380561	-5.028510	1.731884
H	2.138110	-6.080620	1.856784
C	3.301665	-4.632198	0.772398
H	3.775822	-5.382234	0.143328
C	3.630347	-3.287118	0.597641
C	1.324251	-1.707058	3.260318
H	1.749734	-0.716908	3.064452
C	-0.163463	-1.653496	2.891149
H	-0.633344	-2.637584	3.012037
H	-0.289284	-1.333158	1.849692
H	-0.695288	-0.946905	3.539907
C	1.495412	-2.004155	4.755366
H	1.018193	-2.950420	5.033552
H	1.027981	-1.213068	5.352224
H	2.549378	-2.067342	5.046488
C	4.637301	-2.904693	-0.479532
H	4.859605	-1.836929	-0.374641

C	4.041947	-3.124212	-1.876356
H	3.799877	-4.181419	-2.035564
H	4.755224	-2.821740	-2.652043
H	3.119972	-2.547419	-2.012741
C	5.964231	-3.657053	-0.326662
H	6.397698	-3.511799	0.668773
H	6.688721	-3.299911	-1.066678
H	5.839074	-4.734314	-0.482032
Al	-2.251934	0.000747	-0.202449
N	-3.534737	-1.512071	-0.092965
N	-3.304167	0.999644	1.167970
C	-4.404890	-1.752057	0.885766
C	-4.675757	-0.836955	1.912361
H	-5.395727	-1.147373	2.659946
C	-4.225748	0.487373	1.987474
C	-5.201794	-3.035095	0.887306
H	-5.937435	-3.023910	0.076273
H	-5.730300	-3.161527	1.833472
H	-4.555563	-3.899256	0.713899
C	-4.852792	1.351802	3.058048
H	-4.115389	1.607662	3.824492
H	-5.683212	0.830658	3.536428
H	-5.215755	2.294265	2.639000
C	-3.523126	-2.412172	-1.220692
C	-4.449485	-2.193800	-2.260378
C	-4.441673	-3.059258	-3.353813
H	-5.148939	-2.900928	-4.165085
C	-3.543268	-4.114976	-3.426388
H	-3.553555	-4.783778	-4.283037
C	-2.623480	-4.302130	-2.405769
H	-1.909086	-5.119582	-2.472733
C	-2.584790	-3.456952	-1.294267
C	-5.427444	-1.024450	-2.249951
H	-5.373820	-0.536750	-1.270335
C	-5.027727	0.014176	-3.306204
H	-5.074470	-0.415357	-4.313402
H	-4.004315	0.372490	-3.146529
H	-5.706164	0.875586	-3.276397
C	-6.879402	-1.470987	-2.456799
H	-7.555793	-0.613723	-2.368373
H	-7.183658	-2.218642	-1.716467
H	-7.029701	-1.907565	-3.450278
C	-1.522716	-3.692904	-0.229952
H	-1.618283	-2.905847	0.525392
C	-1.705952	-5.040974	0.478362
H	-2.685189	-5.123087	0.963484
H	-0.934086	-5.166502	1.246689
H	-1.612203	-5.875877	-0.225867
C	-0.115810	-3.585224	-0.829903
H	0.640956	-3.708457	-0.048136
H	0.035474	-2.600956	-1.290056
H	0.053637	-4.360411	-1.587440
C	-3.025620	2.411189	1.267614
C	-2.170482	2.905906	2.269275
C	-1.979282	4.286407	2.359198
H	-1.326511	4.683678	3.133333
C	-2.594862	5.157242	1.473028
H	-2.437813	6.229093	1.560666
C	-3.392290	4.650718	0.455363
H	-3.845436	5.336828	-0.254080
C	-3.615428	3.279453	0.324970
C	-1.404171	1.992472	3.215437
H	-1.816992	0.982476	3.120231
C	0.071287	1.927456	2.796939
H	0.526600	2.925591	2.830908
H	0.172020	1.536209	1.776898
H	0.635145	1.277226	3.477473
C	-1.521286	2.413163	4.685423
H	-1.054571	1.659653	5.329353
H	-2.562812	2.532272	5.002852
H	-1.009043	3.363050	4.874044
C	-4.491913	2.750488	-0.805277
H	-4.105851	1.762679	-1.085232
C	-4.427694	3.613571	-2.067490
H	-4.933753	4.576354	-1.933077
H	-4.927880	3.098723	-2.894093
H	-3.392022	3.806311	-2.364389
C	-5.946618	2.558698	-0.357855
H	-6.373477	3.508278	-0.013709
H	-6.031385	1.830678	0.455238
H	-6.558692	2.196071	-1.192386

1-3-difluorobenzene_B3PW91.log

SCF (B3PW91) = -430.555393051
E(SCF)+ZPE(0 K) = -430.470731
H(298 K) = -430.463877
G(298 K) = -430.500849
Lowest Frequency = 234.2166 cm^{-1}

C	1.214034	1.082732	0.000015
C	1.186499	-0.306746	0.000012
C	0.000000	-1.029132	-0.000006
C	-1.186501	-0.306742	-0.000003
C	-1.214034	1.082733	0.000015
C	0.000002	1.767894	0.000016
H	2.166188	1.601227	0.000013
H	-0.000007	-2.112525	-0.000023
H	-2.166184	1.601236	0.000017
H	0.000001	2.853808	0.000021
F	-2.346385	-0.982677	-0.000014
F	2.346385	-0.982676	-0.000022

1-3-difluorobenzene_WB97X.log

SCF (wB97x) = -430.612457592
E(SCF)+ZPE(0 K) = -430.526797
H(298 K) = -430.520018
G(298 K) = -430.556868
Lowest Frequency = 237.3030 cm^{-1}

C	-1.211779	-1.080878	0.000011
C	-1.183238	0.305863	0.000001
C	0.000000	1.028184	-0.000009
C	1.183238	0.305862	-0.000003
C	1.211779	-1.080879	0.000012
C	-0.000001	-1.765168	0.000017
H	-2.164807	-1.598110	0.000014
H	0.000002	2.111710	-0.000021
H	2.164807	-1.598112	0.000017
H	0.000000	-2.851280	0.000026
F	2.343408	0.980994	-0.000009
F	-2.343408	0.980994	-0.000013

Int-1_AlPdAl_B3PW91.log

SCF (B3PW91) = -3040.49238449
E(SCF)+ZPE(0 K) = -3039.128565
H(298 K) = -3039.043932
G(298 K) = -3039.258295
Lowest Frequency = 10.5057 cm^{-1}

Pd	-0.001003	-0.001137	0.073089
Al	2.330666	-0.236858	-0.456911
N	3.543436	-1.808486	-0.213873
N	3.651423	0.633714	-1.685448
C	4.563169	-2.144180	-1.009795
C	5.042399	-1.311044	-2.031170
H	5.861473	-1.703997	-2.620808
C	4.675994	0.015122	-2.291994
C	5.283658	-3.452989	-0.802880
H	5.857084	-3.432292	0.129056
H	5.971758	-3.653416	-1.625961
H	4.577564	-4.281838	-0.714354
C	5.516180	0.761937	-3.299255
H	4.901655	1.132525	-4.123880
H	6.299882	0.120412	-3.704958

H	5.981850	1.640617	-2.843707
C	3.249471	-2.655107	0.916545
C	3.930994	-2.432591	2.135351
C	3.619705	-3.241622	3.231897
H	4.134107	-3.077123	4.175159
C	2.664361	-4.245707	3.141194
H	2.436533	-4.864798	4.004719
C	1.997843	-4.446868	1.940226
H	1.243635	-5.226233	1.872310
C	2.267288	-3.664015	0.812470
C	4.976470	-1.339634	2.309390
H	5.133154	-0.863633	1.336029
C	4.485262	-0.258636	3.282783
H	3.559226	0.208079	2.936716
H	5.241590	0.525812	3.398002
H	4.292209	-0.680906	4.274980
C	6.330629	-1.898039	2.772122
H	6.263510	-2.337247	3.773329
H	7.078022	-1.098158	2.814612
H	6.707394	-2.673835	2.098020
C	1.486899	-3.935051	-0.464236
H	1.837344	-3.233226	-1.228990
C	-0.010464	-3.678268	-0.254446
H	-0.568014	-3.876315	-1.175036
H	-0.190315	-2.631811	0.030242
H	-0.419741	-4.325453	0.529389
C	1.726069	-5.357763	-0.993383
H	2.786977	-5.561908	-1.173096
H	1.191815	-5.503787	-1.938407
H	1.359914	-6.113110	-0.289290
C	3.490344	2.046028	-1.934623
C	2.716405	2.498603	-3.026263
C	2.637320	3.875297	-3.262432
H	2.051603	4.233385	-4.105026
C	3.283681	4.791990	-2.443622
H	3.213738	5.856644	-2.649723
C	4.005320	4.335598	-1.348898
H	4.493525	5.052662	-0.694107
C	4.119441	2.970216	-1.068662
C	1.933236	1.562590	-3.934416
H	2.176744	0.533120	-3.650459
C	0.425709	1.761633	-3.714801
H	0.137470	2.803142	-3.896869
H	0.145271	1.495958	-2.689034
H	-0.150506	1.133521	-4.402130
C	2.289551	1.738223	-5.418099
H	1.998211	2.726863	-5.789175
H	1.761006	0.994313	-6.024023
H	3.361763	1.620144	-5.605313
C	4.912412	2.545667	0.159232
H	4.865265	1.453980	0.228616
C	4.292123	3.119967	1.441136
H	4.305931	4.215386	1.435102
H	4.854426	2.788408	2.320122
H	3.254291	2.797755	1.561846
C	6.394194	2.935436	0.051271
H	6.867453	2.499339	-0.834151
H	6.946034	2.587179	0.931224
H	6.518356	4.022393	-0.007677
Al	-2.332791	0.236093	-0.455237
N	-3.544690	1.807837	-0.210015
N	-3.654913	-0.633510	-1.682707
C	-4.565131	2.144316	-1.004749
C	-5.046326	1.311454	-2.025384
H	-5.866219	1.704709	-2.613665
C	-4.680542	-0.014724	-2.287171
C	-5.282971	3.454540	-0.797799
H	-4.575046	4.283453	-0.724710
H	-5.843741	3.441494	0.141830
H	-5.981279	3.649779	-1.613464
C	-5.523168	-0.761595	-3.292315
H	-4.909958	-1.138506	-4.114998
H	-6.303885	-0.118245	-3.700879
H	-5.993140	-1.636245	-2.833333
C	-3.248679	2.654199	0.920113
C	-3.926542	2.429883	2.140659
C	-3.613618	3.238795	3.236848
H	-4.125464	3.073051	4.181292
C	-2.659865	4.244197	3.144327
H	-2.430586	4.863019	4.007659
C	-1.996641	4.446872	1.941789
H	-1.243428	5.227068	1.872509
C	-2.268101	3.664507	0.814194
C	-4.970498	1.335802	2.316995
H	-5.125061	0.856312	1.345018
C	-4.479601	0.258744	3.294889
H	-4.289678	0.684187	4.286332
H	-3.552084	-0.207587	2.952405
H	-5.234957	-0.526535	3.410765
C	-6.326058	1.894117	2.775852
H	-7.072025	1.093113	2.822237
H	-6.703982	2.665460	2.097329
H	-6.260121	2.338937	3.774667
C	-1.490675	3.936945	-0.464026
H	-1.844123	3.237154	-1.229275
C	-1.728659	5.361106	-0.989859
H	-2.789770	5.568324	-1.164828
H	-1.197900	5.507409	-1.936806
H	-1.357735	6.114441	-0.286118
C	0.006872	3.677691	-0.258377
H	0.562580	3.877047	-1.179800
H	0.186297	2.630525	0.023944
H	0.418624	4.322793	0.525883
C	-3.493995	-2.045655	-1.932830
C	-2.719670	-2.497358	-3.024527
C	-2.640346	-3.873891	-3.261595
H	-2.054463	-4.231324	-4.104357
C	-3.286598	-4.791221	-2.443421
H	-3.216302	-5.855737	-2.650112
C	-4.008615	-4.335656	-1.348590
H	-4.496590	-5.053221	-0.694166
C	-4.123321	-2.970461	-1.067741
C	-1.936699	-1.560559	-3.932017
H	-2.178639	-0.531367	-3.645729
C	-0.429087	-1.761646	-3.714958
H	-0.142029	-2.803050	-3.899436
H	-0.147042	-1.498136	-2.689062
H	0.146780	-1.132734	-4.401838
C	-2.295658	-1.733009	-5.415425
H	-1.766154	-0.989525	-6.021044
H	-3.367842	-1.611769	-5.600751
H	-2.007491	-2.721868	-5.788403
C	-4.916034	-2.546823	0.160590
H	-4.874541	-1.454736	0.227051
C	-4.289202	-3.114708	1.442202
H	-4.295713	-4.210213	1.437668
H	-4.851684	-2.785463	2.321948
H	-3.253193	-2.785523	1.559987
C	-6.395975	-2.944640	0.057480
H	-6.515041	-4.032414	0.003619
H	-6.873348	-2.514828	-0.828822
H	-6.947609	-2.595336	0.937136
H	0.002243	-0.000104	2.411838
C	0.003724	0.000503	3.508488
C	-0.721050	-0.930936	4.237271
C	0.731037	0.932921	4.233441
C	-0.737692	-0.954863	5.627161
C	0.752769	0.958725	5.623196
C	0.008815	0.002350	6.312739
H	-1.322203	-1.706714	6.145936
H	1.339079	1.711325	6.138826
H	0.010782	0.003046	7.398829
F	1.448573	1.852508	3.559450
F	-1.441430	-1.851279	3.567344

Int-1_AlPdAl_WB97X.log

SCF (wB97x) = -3040.82844742
E(SCF)+ZPE(0 K) = -3039.446401
H(298 K) = -3039.364523
G(298 K) = -3039.565559

Lowest Frequency = 11.9048 cm^{-1}

Pd	-0.012772	0.097823	0.966595
Al	2.075014	0.085704	-0.150913
N	3.346042	-1.441029	-0.385604
N	2.880170	1.012932	-1.738663
C	4.100629	-1.699916	-1.452502
C	4.250162	-0.809632	-2.523545
H	4.920516	-1.119630	-3.316610
C	3.774745	0.508680	-2.589382
C	4.917764	-2.969639	-1.506422
H	5.756504	-2.907430	-0.804448
H	5.317510	-3.131257	-2.508816
H	4.321313	-3.835659	-1.209894
C	4.408028	1.384187	-3.649963
H	3.709062	2.096377	-4.089211
H	4.847576	0.771594	-4.439535
H	5.213194	1.968234	-3.189222
C	3.488748	-2.295310	0.768855
C	4.535864	-2.024345	1.674310
C	4.663234	-2.822579	2.810987
H	5.466619	-2.622664	3.516833
C	3.784041	-3.869810	3.055214
H	3.901803	-4.488344	3.941548
C	2.757679	-4.122161	2.157546
H	2.069794	-4.943480	2.347314
C	2.584582	-3.346321	1.008121
C	5.512541	-0.873010	1.465029
H	5.388879	-0.498896	0.443227
C	5.188646	0.283651	2.418479
H	4.158941	0.633512	2.280809
H	5.868481	1.127480	2.248799
H	5.297583	-0.033161	3.463229
C	6.975183	-1.306538	1.615449
H	7.206439	-1.607323	2.643118
H	7.643424	-0.477246	1.360186
H	7.216566	-2.150756	0.960533
C	1.440676	-3.682993	0.063237
H	1.437587	-2.937734	-0.736440
C	0.082377	-3.605333	0.768589
H	-0.722750	-3.828006	0.059394
H	-0.093359	-2.600709	1.170258
H	0.015112	-4.331649	1.588761
C	1.627003	-5.060703	-0.586329
H	2.569870	-5.133770	-1.139872
H	0.805997	-5.255533	-1.285976
H	1.620831	-5.858475	0.165736
C	2.526867	2.402577	-1.900689
C	1.440206	2.754672	-2.729397
C	1.199726	4.109933	-2.961317
H	0.386850	4.401480	-3.621372
C	1.973444	5.096237	-2.362515
H	1.769571	6.145352	-2.560022
C	2.985737	4.733032	-1.487990
H	3.563567	5.506105	-0.986331
C	3.276504	3.390291	-1.235211
C	0.551487	1.705210	-3.389866
H	0.621919	0.796909	-2.782490
C	-0.925614	2.115217	-3.421588
H	-1.105018	2.964693	-4.090535
H	-1.292181	2.384539	-2.427014
H	-1.538989	1.286199	-3.792452
C	1.022039	1.369726	-4.811884
H	1.073227	2.275896	-5.427584
H	0.319368	0.679096	-5.292649
H	2.008557	0.896684	-4.821611
C	4.361495	3.056394	-0.219384
H	4.495168	1.968442	-0.204489
C	3.920785	3.494271	1.184726
H	3.756973	4.577456	1.221836
H	4.689237	3.245681	1.925190
H	2.986480	3.003477	1.479613
C	5.715006	3.678625	-0.581633
H	6.043553	3.382923	-1.583760
H	6.481900	3.361417	0.133509
H	5.673527	4.773215	-0.555650
Al	-2.092473	-0.021299	-0.160731
N	-3.417096	1.485199	-0.200354
N	-3.069435	-0.951659	-1.621338
C	-4.307008	1.735701	-1.150778
C	-4.511703	0.880957	-2.250379
H	-5.249784	1.204355	-2.975244
C	-4.010117	-0.414596	-2.408514
C	-5.229719	2.928925	-1.053512
H	-4.776620	3.760152	-0.511610
H	-6.139606	2.639873	-0.515668
H	-5.525520	3.260598	-2.051440
C	-4.611190	-1.240914	-3.522248
H	-3.841211	-1.563773	-4.227754
H	-5.369279	-0.672811	-4.062820
H	-5.068987	-2.150184	-3.120404
C	-3.427797	2.269645	1.010766
C	-4.371980	1.967050	2.010860
C	-4.337237	2.689577	3.205551
H	-5.059604	2.462214	3.986456
C	-3.390897	3.679859	3.417301
H	-3.375317	4.232001	4.353331
C	-2.452273	3.952627	2.431063
H	-1.699198	4.713598	2.610980
C	-2.442800	3.257275	1.220945
C	-5.393927	0.846301	1.871193
H	-5.337029	0.446172	0.852946
C	-5.062674	-0.297283	2.838598
H	-5.147618	0.038197	3.878718
H	-4.042003	-0.669198	2.694315
H	-5.760402	-1.131748	2.699603
C	-6.830129	1.335298	2.095935
H	-7.542437	0.525281	1.905573
H	-7.083678	2.173903	1.438947
H	-6.981274	1.669637	3.128234
C	-1.394742	3.586646	0.167371
H	-1.164910	2.653867	-0.359743
C	-1.928592	4.606621	-0.846972
H	-2.780593	4.218504	-1.415214
H	-1.142969	4.873833	-1.562341
H	-2.249516	5.523230	-0.337081
C	-0.071724	4.075258	0.761754
H	0.690137	4.104259	-0.023124
H	0.279055	3.407601	1.554212
H	-0.156680	5.090028	1.170939
C	-2.773449	-2.356485	-1.781143
C	-1.824214	-2.796184	-2.726105
C	-1.597761	-4.167799	-2.859865
H	-0.869934	-4.515646	-3.589867
C	-2.281046	-5.094595	-2.086841
H	-2.099210	-6.158359	-2.216019
C	-3.185583	-4.647937	-1.135657
H	-3.706658	-5.371349	-0.512907
C	-3.434927	-3.286535	-0.952017
C	-1.009266	-1.849325	-3.597416
H	-1.363194	-0.829425	-3.412668
C	0.472667	-1.910133	-3.202778
H	0.862312	-2.931421	-3.291998
H	0.601471	-1.586914	-2.164429
H	1.081143	-1.264959	-3.846303
C	-1.169444	-2.149266	-5.094018
H	-0.634053	-1.402734	-5.690973
H	-2.217530	-2.144060	-5.410422
H	-0.753750	-3.130002	-5.350149
C	-4.406808	-2.876582	0.147509
H	-4.414737	-1.782829	0.210456
C	-3.949520	-3.416965	1.508753
H	-3.893400	-4.511015	1.506061
H	-4.655430	-3.125941	2.293103
H	-2.962717	-3.025769	1.779134
C	-5.838902	-3.322048	-0.172035
H	-5.910212	-4.413859	-0.237182
H	-6.186202	-2.906538	-1.124434
H	-6.527501	-2.988355	0.612450
H	-1.388544	0.987679	3.296894
C	-0.469464	0.455074	3.522487

C	-0.437533	-0.931563	3.667126
C	0.720218	1.127885	3.783836
C	0.705581	-1.625068	4.034381
C	1.881578	0.485008	4.189889
C	1.863901	-0.899431	4.304534
H	0.679753	-2.707245	4.104523
H	2.776055	1.068078	4.382111
H	2.769989	-1.430030	4.582647
F	0.727430	2.472022	3.685877
F	-1.581179	-1.621759	3.482008

Int-2_AlPdAl_B3PW91.log

SCF (B3PW91) = -3040.58034943
E(SCF)+ZPE(0 K) = -3039.214964
H(298 K) = -3039.131338
G(298 K) = -3039.339947
Lowest Frequency = 8.1681 cm^{-1}

Pd	-0.521945	-0.219943	0.032820
Al	2.856055	0.188483	-0.775414
N	4.715021	-0.212894	-0.333346
N	3.091198	2.093185	-0.983412
C	5.685630	0.535887	-0.866586
C	5.460049	1.812075	-1.407083
H	6.330210	2.305348	-1.822908
C	4.278740	2.564524	-1.400842
C	7.105073	0.027962	-0.939984
H	7.333040	-0.694911	-0.156826
H	7.818176	0.853554	-0.891964
H	7.245591	-0.473997	-1.904188
C	4.385642	3.980682	-1.909250
H	3.888241	4.069924	-2.879781
H	5.430248	4.271325	-2.028838
H	3.890700	4.688357	-1.240997
C	5.064824	-1.411458	0.389445
C	5.309813	-1.311000	1.780752
C	5.645579	-2.473890	2.478981
H	5.838792	-2.416519	3.545775
C	5.730180	-3.704432	1.837479
C	5.478519	-3.786359	0.475488
H	5.544276	-4.749573	-0.023730
C	5.144377	-2.653700	-0.276100
C	1.977388	3.011701	-0.973912
C	1.227882	3.221100	-2.152985
C	0.206940	4.176108	-2.123820
H	-0.375085	4.352362	-3.024184
C	-0.077615	4.899287	-0.973252
C	0.646870	4.656977	0.186874
H	0.407684	5.210287	1.090366
C	1.675589	3.711054	0.215778
C	1.627646	-0.647511	0.528228
C	1.648913	-0.380058	1.928094
C	1.031819	-1.890347	0.137762
C	1.219410	-1.329782	2.834894
H	2.053382	0.549573	2.315004
C	0.617414	-2.844633	1.094182
H	1.055661	-2.190220	-0.907215
C	0.730055	-2.579073	2.449520
H	0.212589	-3.794421	0.757520
H	0.435726	-3.298272	3.207094
F	2.624927	-0.441375	-2.323899
F	1.296728	-1.053556	4.160597
H	5.989512	-4.596689	2.401069
H	-0.872584	5.639485	-0.974285
C	1.464189	2.431095	-3.432163
C	1.683929	3.328628	-4.658538
C	0.296259	1.466742	-3.685119
H	2.357398	1.815835	-3.296930
H	2.503748	4.040389	-4.515954
H	1.922906	2.714814	-5.533542
H	0.786639	3.908143	-4.902528
H	0.109740	0.830590	-2.814449
H	-0.625949	2.017482	-3.902828
H	0.516680	0.817671	-4.539033
C	2.441984	3.481701	1.510467
C	1.503310	3.308621	2.712334
C	3.454402	4.604278	1.786556
H	3.010356	2.552718	1.390367
H	0.730465	2.561740	2.511941
H	2.070789	2.991797	3.594347
H	1.002882	4.247705	2.972585
H	4.209402	4.678946	0.998774
H	2.950037	5.574359	1.862134
H	3.978122	4.425123	2.732222
C	4.905190	-2.812495	-1.771158
C	3.729764	-3.757492	-2.057749
C	6.164541	-3.310340	-2.497736
H	4.635514	-1.835354	-2.180629
H	2.824615	-3.430644	-1.542309
H	3.518311	-3.783549	-3.131848
H	3.951038	-4.781259	-1.734590
H	7.030364	-2.668107	-2.311606
H	6.434663	-4.323014	-2.177451
H	5.991829	-3.341653	-3.579063
C	5.256567	0.023069	2.515666
C	6.637411	0.698801	2.563494
C	4.699547	-0.085967	3.940445
H	4.590312	0.678765	1.943512
H	7.026029	0.921630	1.567482
H	6.580608	1.643332	3.116062
H	7.364158	0.055413	3.072622
H	3.743436	-0.611476	3.976281
H	5.397085	-0.601064	4.610454
H	4.549658	0.917022	4.354737
Al	-2.886260	-0.371946	-0.009740
N	-4.025553	-1.981847	-0.356295
N	-4.486116	0.706693	0.461463
C	-5.766007	0.306087	0.454871
C	-6.172057	-0.962338	0.027204
C	-5.360252	-2.025326	-0.396656
H	-7.240491	-1.139328	0.004440
C	-6.849766	1.256504	0.899420
H	-6.620857	1.671572	1.884819
H	-7.816768	0.752799	0.941930
H	-6.929373	2.107181	0.215891
H	-6.049561	-3.188395	-2.048015
H	-7.118180	-3.250357	-0.640720
C	-6.072316	-3.235054	-0.953196
H	-5.596252	-4.172159	-0.663766
C	-4.208430	2.077458	0.811255
C	-4.303094	3.073442	-0.187500
C	-3.995560	4.391645	0.158891
C	-3.605425	4.727512	1.450294
C	-3.516618	3.737550	2.419719
C	-3.814239	2.402328	2.126482
H	-3.211495	4.002206	3.428763
H	-3.373795	5.759563	1.700024
H	-4.062217	5.168866	-0.597532
C	-4.697544	2.748013	-1.621739
H	-5.114692	1.735997	-1.634643
C	-3.461837	2.743212	-2.533013
H	-2.698589	2.050476	-2.164723
H	-3.734870	2.451843	-3.553888
H	-3.008225	3.739347	-2.573939
H	-6.656869	3.731800	-1.527200
C	-5.774870	3.690780	-2.174956
H	-6.099063	3.352370	-3.164925
H	-5.402110	4.714223	-2.289896
H	-4.041211	0.403923	2.815442
C	-3.713846	1.362051	3.231757
C	-4.640392	1.687620	4.412751
H	-4.346740	2.618604	4.909873
H	-5.683255	1.796788	4.097129
C	-2.264903	1.187393	3.707147
H	-2.205691	0.412035	4.478867
H	-1.612470	0.895064	2.877725
H	-1.874636	2.116376	4.137869
H	-4.597068	0.888449	5.160595
C	-3.292328	-3.181551	-0.670708
C	-2.851762	-3.403154	-1.994294

C	-2.127121	-4.565980	-2.275413
C	-1.843634	-5.494914	-1.284498
C	-2.274910	-5.256066	0.014822
C	-2.993680	-4.104484	0.360055
C	-3.406987	-3.994292	1.830350
H	-2.045760	-5.978695	0.795186
H	-1.283164	-6.395837	-1.518800
H	-1.780806	-4.742000	-3.290302
C	-3.125036	-2.423778	-3.126244
H	-3.753187	-1.618705	-2.729064
C	-3.891382	-3.077091	-4.286176
H	-4.822398	-3.546787	-3.952766
H	-4.142294	-2.327911	-5.044889
H	-3.291353	-3.850857	-4.777270
H	-2.032569	-1.041588	-4.403045
C	-1.821597	-1.787639	-3.628846
H	-1.287094	-1.295236	-2.809985
H	-1.155882	-2.541322	-4.064238
H	-2.736500	-4.694548	2.345699
H	-5.003821	-5.483032	1.629206
C	-4.833226	-4.505229	2.091435
H	-5.001576	-4.608088	3.169405
H	-5.585664	-3.807498	1.711844
H	-3.234861	-2.767889	3.598786
C	-3.194103	-2.638240	2.511065
H	-3.979443	-1.924648	2.244594
H	-2.220205	-2.205996	2.260334

Int-2_AlPdAl_WB97X.log

SCF (wB97x) = -3040.92231128
E(SCF)+ZPE(0 K) = -3039.539885
H(298 K) = -3039.458249
G(298 K) = -3039.657676
Lowest Frequency = 15.8046 cm^{-1}

Pd	-0.245901	0.279135	-0.083367
Al	3.369136	0.169701	-0.613129
N	3.802418	-1.619823	-0.089263
N	4.014827	1.002901	0.985342
C	4.892646	-1.829411	0.649592
C	5.553980	-0.811684	1.355158
H	6.466364	-1.094504	1.866449
C	5.104712	0.501984	1.567739
C	5.434351	-3.229594	0.783593
H	4.748008	-3.843653	1.375806
H	6.407378	-3.224437	1.276352
H	5.526221	-3.706114	-0.196099
C	5.941127	1.366843	2.479064
H	6.666789	1.909734	1.861600
H	6.495080	0.758870	3.196630
H	5.345408	2.108895	3.012089
C	3.085064	-2.734234	-0.655093
C	2.119421	-3.393518	0.130366
C	1.336874	-4.378341	-0.473225
H	0.563480	-4.873568	0.110438
C	1.518838	-4.723488	-1.805871
C	2.507026	-4.096938	-2.551521
H	2.661711	-4.386539	-3.588545
C	3.310674	-3.097819	-1.997025
C	3.476696	2.271329	1.402787
C	3.869985	3.454585	0.752574
C	3.258169	4.650376	1.137517
H	3.544199	5.575715	0.642227
C	2.298891	4.677457	2.138995
C	1.934421	3.499493	2.779742
H	1.184476	3.531733	3.565443
C	2.504600	2.277169	2.424072
C	1.581062	0.623546	-1.326933
C	0.970470	-0.225323	-2.296779
C	1.125534	1.975298	-1.326426
C	0.107654	0.297193	-3.252441
H	1.231398	-1.279261	-2.371737
C	0.250013	2.469734	-2.301460
H	1.534709	2.680048	-0.604494
C	-0.236709	1.638720	-3.304166
H	-0.054737	3.513000	-2.273183
H	-0.906215	1.999980	-4.079035
F	4.525283	0.569869	-1.779311
F	-0.421711	-0.542753	-4.173143
H	0.897014	-5.490670	-2.260985
H	1.835690	5.618077	2.426368
C	4.933454	3.480073	-0.337955
C	6.115771	4.371633	0.063408
C	4.352005	3.933138	-1.682673
H	5.306994	2.462342	-0.482775
H	6.537924	4.076181	1.030060
H	6.909136	4.311674	-0.689024
H	5.814163	5.422348	0.143755
H	3.535753	3.281299	-2.007513
H	3.967785	4.958637	-1.623094
H	5.126461	3.906721	-2.455970
C	2.113221	1.001734	3.162522
C	0.615324	0.913548	3.458613
C	2.916919	0.854920	4.462702
H	2.365491	0.153539	2.514834
H	0.023415	0.963830	2.535641
H	0.394827	-0.042298	3.946146
H	0.280105	1.706871	4.137456
H	3.994584	0.804680	4.280450
H	2.728235	1.703831	5.130665
H	2.624723	-0.060318	4.989288
C	4.413428	-2.476409	-2.847390
C	3.856895	-1.763357	-4.085382
C	5.443560	-3.535991	-3.261793
H	4.929315	-1.718944	-2.251368
H	3.167844	-0.959294	-3.812105
H	4.675031	-1.316157	-4.659061
H	3.324520	-2.460430	-4.743364
H	5.870625	-4.052541	-2.395139
H	4.994581	-4.296519	-3.910954
H	6.265388	-3.068966	-3.814610
C	1.915693	-3.083375	1.607746
C	2.042810	-4.348410	2.468079
C	0.566930	-2.407378	1.862301
H	2.699929	-2.385866	1.922275
H	2.975717	-4.889792	2.274743
H	2.014883	-4.086298	3.531155
H	1.213259	-5.039277	2.279173
H	0.468392	-1.461445	1.307721
H	-0.264328	-3.050305	1.552162
H	0.443378	-2.189770	2.930304
Al	-2.592068	0.137823	0.243466
N	-4.018427	1.435989	-0.248768
N	-3.950550	-1.237910	0.674737
C	-5.278250	-1.090679	0.676506
C	-5.920938	0.123444	0.408140
C	-5.321608	1.323887	-0.002913
H	-6.998317	0.135514	0.522450
C	-6.159770	-2.272236	1.005690
H	-5.910162	-3.125868	0.368502
H	-7.212666	-2.021423	0.870563
H	-6.004502	-2.595624	2.039939
H	-6.086754	3.162697	0.753252
H	-7.276839	2.222708	-0.162347
C	-6.229112	2.526554	-0.128086
H	-6.000374	3.134398	-1.004093
C	-3.409514	-2.526731	1.038611
C	-3.248561	-2.847026	2.402207
C	-2.697239	-4.084056	2.734938
C	-2.311916	-4.988655	1.753005
C	-2.469668	-4.655916	0.415651
C	-3.015105	-3.427527	0.032972
H	-2.165124	-5.361888	-0.354604
H	-1.888171	-5.950260	2.032415
H	-2.562146	-4.343944	3.781990
C	-3.619688	-1.868668	3.509695
H	-4.264016	-1.094890	3.079280
C	-2.361556	-1.171140	4.041742
H	-1.820469	-0.663921	3.235662
H	-2.619825	-0.430337	4.807300

H	-1.678217	-1.901724	4.492317
H	-5.280547	-3.068386	4.284629
C	-4.400493	-2.528596	4.651329
H	-4.740255	-1.768779	5.362806
H	-3.782205	-3.240824	5.208357
H	-3.622795	-2.126560	-1.535471
C	-3.162662	-3.114136	-1.448345
C	-4.093595	-4.110149	-2.149974
H	-3.685272	-5.126823	-2.122081
H	-5.085316	-4.136693	-1.684565
C	-1.797309	-3.052387	-2.143193
H	-1.902682	-2.694267	-3.173158
H	-1.118548	-2.371808	-1.617255
H	-1.324227	-4.041108	-2.175264
H	-4.220773	-3.832727	-3.201883
C	-3.537621	2.645191	-0.872271
C	-3.029583	3.689200	-0.076697
C	-2.559870	4.845002	-0.703743
C	-2.588245	4.975074	-2.083154
C	-3.074714	3.928289	-2.853968
C	-3.547356	2.745056	-2.280274
C	-4.071850	1.691659	-3.262191
H	-3.085652	4.022402	-3.938483
H	-2.225521	5.882847	-2.558303
H	-2.166234	5.656216	-0.095373
C	-2.964855	3.611756	1.442338
H	-3.386583	2.649104	1.752571
C	-3.797464	4.716739	2.104984
H	-4.840330	4.699944	1.770698
H	-3.787086	4.601219	3.194111
H	-3.394176	5.709066	1.873860
H	-1.468425	3.518169	3.017125
C	-1.511866	3.661560	1.930508
H	-0.912676	2.878830	1.453303
H	-1.049488	4.629500	1.701693
H	-3.605911	1.962430	-4.218826
H	-5.899082	2.831667	-3.662251
C	-5.588820	1.797528	-3.478259
H	-5.886707	1.194366	-4.342924
H	-6.142629	1.417835	-2.612864
H	-3.850942	-0.353723	-3.912872
C	-3.682287	0.233311	-3.002766
H	-4.298820	-0.208380	-2.213829
H	-2.625078	0.130750	-2.735822

Int-3_AlPdAl_B3PW91.log

SCF (B3PW91) = -3040.48209327
E(SCF)+ZPE(0 K) = -3039.120783
H(298 K) = -3039.037474
G(298 K) = -3039.242452
Lowest Frequency = 14.7583 cm^{-1}

Pd	0.099795	-0.041027	-0.784564
Al	-1.916917	-0.174327	0.495698
N	-3.124939	-1.748573	0.505223
N	-3.047025	0.797654	1.823850
C	-3.995590	-2.036319	1.480950
C	-4.329816	-1.138518	2.502652
H	-5.042055	-1.496704	3.235142
C	-3.968379	0.207873	2.606380
C	-4.698455	-3.370725	1.498904
H	-5.284215	-3.520282	0.588257
H	-5.361061	-3.448756	2.362124
H	-3.972103	-4.187060	1.534157
C	-4.689792	1.007291	3.664755
H	-4.000899	1.318051	4.454616
H	-5.487318	0.415386	4.115906
H	-5.118791	1.922267	3.249172
C	-3.064831	-2.681536	-0.600487
C	-3.946801	-2.501743	-1.689453
C	-3.921043	-3.439238	-2.725941
H	-4.593512	-3.312732	-3.570152
C	-3.052091	-4.521964	-2.700326
H	-3.046836	-5.238857	-3.516916
C	-2.181099	-4.674100	-1.630684
H	-1.487333	-5.510214	-1.621406
C	-2.166915	-3.767588	-0.566779
C	-4.922321	-1.337266	-1.784166
H	-4.769525	-0.699364	-0.907450
C	-4.649869	-0.487764	-3.032740
H	-3.612451	-0.146053	-3.065565
H	-5.307835	0.388310	-3.049031
H	-4.838496	-1.055955	-3.949818
C	-6.385207	-1.805824	-1.758896
H	-6.610025	-2.451245	-2.615084
H	-7.062445	-0.945852	-1.806897
H	-6.623273	-2.366591	-0.849417
C	-1.165588	-3.970336	0.557011
H	-1.383366	-3.235752	1.340472
C	0.256576	-3.701205	0.051932
H	0.979226	-3.818412	0.863849
H	0.340045	-2.684307	-0.349192
H	0.524825	-4.399640	-0.748253
C	-1.256737	-5.364905	1.193446
H	-2.262227	-5.590412	1.564943
H	-0.561384	-5.437789	2.036410
H	-0.986419	-6.151187	0.480411
C	-2.906533	2.228740	1.974310
C	-2.111191	2.765806	3.012286
C	-2.087914	4.153724	3.182845
H	-1.486617	4.578503	3.982193
C	-2.813640	4.999290	2.353339
H	-2.786993	6.074452	2.508402
C	-3.562826	4.459890	1.316652
H	-4.122539	5.121893	0.661968
C	-3.625814	3.078722	1.102558
C	-1.262018	1.905606	3.936957
H	-1.529822	0.858205	3.763676
C	0.226995	2.064391	3.595283
H	0.541712	3.108856	3.699496
H	0.426642	1.744111	2.568078
H	0.842892	1.461277	4.271606
C	-1.490830	2.213212	5.424859
H	-1.123866	3.210016	5.691843
H	-0.948099	1.492635	6.046141
H	-2.547657	2.168750	5.706154
C	-4.511462	2.553761	-0.019282
H	-4.311384	1.482914	-0.131187
C	-4.204575	3.219694	-1.366468
H	-4.411390	4.295334	-1.344334
H	-4.836551	2.785696	-2.148242
H	-3.162821	3.073619	-1.658773
C	-6.000300	2.704400	0.331905
H	-6.262099	2.168813	1.249582
H	-6.625176	2.307964	-0.475661
H	-6.265808	3.758281	0.472478
Al	2.173829	0.240605	0.313354
N	3.365624	1.746742	-0.118258
N	3.528570	-0.683633	1.398465
C	4.473318	2.051210	0.563800
C	5.006073	1.226894	1.567998
H	5.890545	1.600357	2.068390
C	4.623042	-0.076981	1.894194
C	5.230799	3.317028	0.252595
H	4.614498	4.194437	0.468218
H	5.485207	3.372095	-0.808958
H	6.146722	3.377981	0.842034
C	5.540039	-0.831737	2.824217
H	4.991921	-1.266655	3.662667
H	6.321760	-0.176522	3.210882
H	6.014799	-1.667039	2.300298
C	3.035969	2.578153	-1.256769
C	3.500989	2.200429	-2.536755
C	3.235142	3.049418	-3.615018
H	3.594230	2.776942	-4.603419
C	2.515519	4.225748	-3.451419
H	2.318666	4.869848	-4.304072
C	2.032555	4.561167	-2.194462
H	1.443447	5.465771	-2.072186
C	2.277767	3.753271	-1.079893

C	4.278327	0.917793	-2.793177
H	4.241632	0.317798	-1.877851
C	3.636154	0.085800	-3.911720
H	3.695856	0.594943	-4.879619
H	2.583028	-0.114461	-3.697586
H	4.156079	-0.872279	-4.017057
C	5.756543	1.198864	-3.103494
H	6.297373	0.262243	-3.279351
H	6.255845	1.722927	-2.282268
H	5.859056	1.815683	-4.003329
C	1.665534	4.143126	0.254081
H	2.061890	3.468154	1.021115
C	2.007048	5.578914	0.677609
H	3.086657	5.761452	0.706733
H	1.604815	5.782603	1.675827
H	1.568475	6.315094	-0.004452
C	0.145950	3.944113	0.196276
H	-0.314993	4.191382	1.156146
H	-0.104877	2.908652	-0.054730
H	-0.300790	4.587667	-0.568949
C	3.364389	-2.084826	1.711097
C	2.675267	-2.479248	2.879607
C	2.602678	-3.843693	3.180652
H	2.083164	-4.159286	4.081648
C	3.180157	-4.800656	2.355731
H	3.116751	-5.854750	2.611602
C	3.826517	-4.399661	1.193790
H	4.261045	-5.149320	0.538053
C	3.931684	-3.048940	0.847028
C	2.001249	-1.489744	3.818194
H	2.211425	-0.480634	3.448984
C	0.478042	-1.681111	3.798655
H	0.200666	-2.691820	4.117660
H	0.083312	-1.516073	2.791630
H	-0.006457	-0.973193	4.479621
C	2.529971	-1.584650	5.257523
H	2.062700	-0.816721	5.883572
H	3.613809	-1.443639	5.312751
H	2.300841	-2.556763	5.707279
C	4.643752	-2.679686	-0.446240
H	4.686961	-1.587634	-0.505965
C	3.856594	-3.182049	-1.665776
H	3.759144	-4.273200	-1.650814
H	4.372141	-2.908128	-2.592163
H	2.850896	-2.754507	-1.698964
C	6.089374	-3.196928	-0.483067
H	6.127062	-4.291612	-0.476145
H	6.674840	-2.840202	0.370475
H	6.588977	-2.858058	-1.396834
H	1.654670	-0.232737	-1.428633
C	-0.712415	-0.065628	-2.713795
C	-0.680202	-1.164494	-3.566675
C	-1.137853	1.095722	-3.349338
C	-1.052851	-1.155342	-4.908333
C	-1.526156	1.196658	-4.682535
C	-1.483391	0.046190	-5.468543
H	-0.994285	-2.069546	-5.490994
H	-1.843966	2.154042	-5.084008
H	-1.778376	0.087341	-6.513344
F	-1.178284	2.253784	-2.621368
F	-0.233181	-2.351729	-3.072334

Int-3_AlPdAl_WB97X.log

SCF (wB97x) = -3040.82745914
E(SCF)+ZPE(0 K) = -3039.447842
H(298 K) = -3039.367516
G(298 K) = -3039.560042
Lowest Frequency = 21.3799 cm^{-1}

Pd	-0.049535	-0.281324	1.050670
Al	1.704483	0.092318	-0.460581
N	3.011505	-1.317233	-0.828393
N	2.492367	1.280302	-1.811857
C	3.724385	-1.436333	-1.945250
C	3.803551	-0.421692	-2.913207
H	4.429849	-0.623194	-3.773821
C	3.336325	0.888329	-2.776416
C	4.541471	-2.680827	-2.192653
H	5.206740	-2.880378	-1.348235
H	5.135602	-2.580658	-3.102063
H	3.885750	-3.550367	-2.292794
C	3.918069	1.898437	-3.740019
H	3.216546	2.688514	-4.008340
H	4.269017	1.398538	-4.644868
H	4.781259	2.381149	-3.267486
C	3.182068	-2.320825	0.202794
C	4.057011	-2.033956	1.273790
C	4.222193	-3.005503	2.258973
H	4.876890	-2.810936	3.101800
C	3.533602	-4.211210	2.207111
H	3.667068	-4.945818	2.996756
C	2.656453	-4.458750	1.166083
H	2.093804	-5.388883	1.147687
C	2.462984	-3.524184	0.146406
C	4.781693	-0.693042	1.385365
H	4.050940	0.087850	1.130291
C	5.267660	-0.385006	2.803947
H	4.469969	-0.508393	3.541052
H	5.622580	0.649657	2.854027
H	6.109437	-1.028377	3.086967
C	5.966842	-0.567395	0.416285
H	6.690942	-1.371812	0.593192
H	6.484272	0.386293	0.578447
H	5.666860	-0.600178	-0.634314
C	1.440809	-3.824277	-0.939596
H	1.535611	-3.062684	-1.721649
C	0.019978	-3.731900	-0.372770
H	-0.715472	-3.910093	-1.163337
H	-0.163488	-2.747183	0.072234
H	-0.131539	-4.476137	0.417165
C	1.656324	-5.190490	-1.602635
H	2.671694	-5.310560	-1.997167
H	0.950104	-5.316168	-2.431200
H	1.479254	-6.009272	-0.896854
C	2.233213	2.697817	-1.708399
C	1.098462	3.252070	-2.333510
C	0.929044	4.636833	-2.282126
H	0.071768	5.087940	-2.775153
C	1.832771	5.451547	-1.612485
H	1.680283	6.527307	-1.585001
C	2.921657	4.884136	-0.967842
H	3.615086	5.522211	-0.425347
C	3.144593	3.506268	-1.002749
C	0.093701	2.397015	-3.097146
H	0.171545	1.376211	-2.707913
C	-1.353916	2.856070	-2.891602
H	-1.551826	3.818018	-3.377832
H	-1.601425	2.960927	-1.830763
H	-2.048175	2.130171	-3.330371
C	0.410744	2.356570	-4.598366
H	0.444495	3.370398	-5.014621
H	-0.365135	1.799934	-5.136610
H	1.370232	1.873461	-4.806275
C	4.344116	2.932355	-0.259178
H	4.458427	1.881394	-0.547240
C	4.109402	2.979362	1.256893
H	3.952893	4.011634	1.591839
H	4.983091	2.584959	1.788753
H	3.236567	2.394432	1.566379
C	5.652872	3.645440	-0.620491
H	5.821153	3.671237	-1.702647
H	6.500860	3.129415	-0.157532
H	5.663487	4.679328	-0.258353
Al	-1.981132	0.180583	-0.141148
N	-3.206378	1.617494	0.317146
N	-3.089373	-0.528754	-1.556527
C	-4.172663	2.055877	-0.481761
C	-4.523205	1.414796	-1.684783
H	-5.319893	1.876933	-2.255555
C	-4.102876	0.153126	-2.113428

C	-5.023556	3.237301	-0.084291
H	-4.480754	3.942421	0.546280
H	-5.892223	2.887729	0.483768
H	-5.392886	3.748245	-0.976192
C	-4.878775	-0.466088	-3.251328
H	-4.223760	-0.711669	-4.090448
H	-5.661778	0.209624	-3.596711
H	-5.338519	-1.405670	-2.929183
C	-3.131448	2.138021	1.666932
C	-4.048554	1.664892	2.625931
C	-3.967542	2.166998	3.924808
H	-4.664342	1.807310	4.678358
C	-3.005989	3.102353	4.275456
H	-2.956764	3.481330	5.292729
C	-2.095858	3.538438	3.324528
H	-1.332383	4.257096	3.607580
C	-2.130908	3.063645	2.012225
C	-5.086419	0.588999	2.329862
H	-5.081444	0.379875	1.254000
C	-4.717904	-0.711021	3.056693
H	-4.749497	-0.569551	4.142865
H	-3.707286	-1.040161	2.793913
H	-5.427112	-1.508143	2.801870
C	-6.508276	1.023809	2.707100
H	-7.231575	0.259946	2.402080
H	-6.791044	1.969101	2.231837
H	-6.610144	1.157920	3.789416
C	-1.123476	3.590516	1.001829
H	-1.036056	2.851254	0.198645
C	-1.610833	4.910824	0.390407
H	-2.551988	4.787721	-0.157841
H	-0.864639	5.305972	-0.306751
H	-1.774696	5.659395	1.174801
C	0.280422	3.751106	1.590546
H	0.986447	3.985136	0.787125
H	0.612997	2.834230	2.087423
H	0.329116	4.576117	2.311562
C	-2.876303	-1.887703	-2.008795
C	-2.101177	-2.148255	-3.156517
C	-2.010283	-3.464090	-3.614237
H	-1.420796	-3.677602	-4.503087
C	-2.658268	-4.502802	-2.961369
H	-2.588233	-5.518106	-3.342482
C	-3.370655	-4.238275	-1.801674
H	-3.848846	-5.056698	-1.268856
C	-3.478451	-2.941819	-1.294144
C	-1.327797	-1.068882	-3.903150
H	-1.577373	-0.100231	-3.455983
C	0.181249	-1.291067	-3.731050
H	0.489586	-2.248643	-4.167067
H	0.448577	-1.310392	-2.669076
H	0.756883	-0.498433	-4.222350
C	-1.681881	-1.011106	-5.395036
H	-1.130614	-0.201662	-5.885725
H	-2.749693	-0.838742	-5.563379
H	-1.413733	-1.942679	-5.904876
C	-4.230000	-2.737633	0.014921
H	-4.165007	-1.679798	0.291016
C	-3.580146	-3.549264	1.143694
H	-3.590896	-4.621786	0.919760
H	-4.125681	-3.401703	2.081110
H	-2.540851	-3.244500	1.307716
C	-5.718695	-3.075271	-0.129903
H	-5.865176	-4.130649	-0.386392
H	-6.195355	-2.471460	-0.910444
H	-6.246105	-2.885566	0.811657
H	-1.713221	-0.688169	1.472419
C	0.922712	-0.864113	2.831584
C	0.767645	-2.126129	3.391887
C	1.680881	-0.028186	3.635249
C	1.296294	-2.551560	4.604649
C	2.237067	-0.354954	4.867704
C	2.041610	-1.644785	5.352184
H	1.116232	-3.566175	4.947354
H	2.804047	0.387535	5.421899
H	2.462121	-1.938913	6.310224
F	1.927109	1.253746	3.199206
F	0.026383	-3.050908	2.709426

Int-4_AlPdAl_B3PW91.log

SCF (B3PW91) = -3040.49415792
E(SCF)+ZPE(0 K) = -3039.133177
H(298 K) = -3039.049508
G(298 K) = -3039.256197
Lowest Frequency = 12.1589 cm^{-1}

Pd	-0.000006	-0.000057	-0.087329
Al	2.348482	0.224281	0.364728
N	3.568786	1.751285	0.085084
N	3.740163	-0.806486	1.336741
C	4.749825	1.941433	0.688187
C	5.354637	0.982864	1.508174
H	6.309374	1.257429	1.939098
C	4.921289	-0.324258	1.755061
C	5.506777	3.226548	0.468303
H	5.767505	3.348864	-0.586787
H	6.422563	3.245001	1.061094
H	4.891824	4.090011	0.735939
C	5.864396	-1.198945	2.544855
H	5.944623	-2.199465	2.115367
H	5.498111	-1.323655	3.568265
H	6.857125	-0.748580	2.592484
C	3.145667	2.750025	-0.870564
C	3.541429	2.614931	-2.219269
C	3.118722	3.585532	-3.132119
H	3.406359	3.492022	-4.175876
C	2.335352	4.660507	-2.732140
H	2.015281	5.402958	-3.458224
C	1.958227	4.778324	-1.401000
H	1.337314	5.615212	-1.093279
C	2.351147	3.834795	-0.447604
C	4.399666	1.458594	-2.713267
H	4.700830	0.867017	-1.842184
C	3.600728	0.537791	-3.645692
H	2.695251	0.161318	-3.162130
H	4.212214	-0.317215	-3.955712
H	3.285574	1.067983	-4.550324
C	5.685628	1.941228	-3.400473
H	5.466511	2.502822	-4.314919
H	6.308349	1.085520	-3.683585
H	6.282108	2.589349	-2.749925
C	1.894075	3.996908	0.992652
H	2.417377	3.247813	1.597422
C	0.390458	3.722696	1.114230
H	0.066909	3.810235	2.155974
H	0.137868	2.716975	0.758829
H	-0.188969	4.439730	0.523679
C	2.243872	5.376111	1.570435
H	3.312488	5.601872	1.487157
H	1.972405	5.420420	2.630777
H	1.697581	6.177372	1.061300
C	3.480867	-2.207907	1.571085
C	2.994658	-2.644099	2.823941
C	2.808218	-4.016047	3.019981
H	2.437013	-4.367887	3.978853
C	3.088585	-4.936384	2.018321
H	2.943945	-5.998678	2.195738
C	3.547737	-4.490015	0.785868
H	3.761372	-5.211705	0.002773
C	3.747248	-3.129234	0.531639
C	2.655220	-1.689448	3.960553
H	2.994942	-0.688473	3.673882
C	1.137056	-1.614811	4.177244
H	0.738315	-2.591850	4.473866
H	0.621521	-1.295390	3.267753
H	0.902490	-0.902531	4.975933
C	3.350816	-2.064542	5.278539
H	2.962922	-3.005949	5.682387
H	3.172133	-1.290630	6.032703

H	4.433146	-2.181626	5.165132
C	4.298013	-2.702945	-0.823331
H	4.155364	-1.620224	-0.917320
C	3.563293	-3.358917	-1.998415
H	3.718320	-4.443335	-2.021631
H	3.948246	-2.960237	-2.942968
H	2.489766	-3.162743	-1.963338
C	5.808734	-2.972078	-0.916489
H	6.370961	-2.436342	-0.145820
H	6.194230	-2.655376	-1.891767
H	6.021313	-4.041304	-0.803553
Al	-2.348436	-0.224306	0.364910
N	-3.568759	-1.751284	0.085393
N	-3.739984	0.806590	1.336922
C	-4.749744	-1.941370	0.688628
C	-5.354452	-0.982746	1.508623
H	-6.309140	-1.257277	1.939675
C	-4.921065	0.324387	1.755393
C	-5.506752	-3.226472	0.468871
H	-5.767892	-3.348638	-0.586136
H	-6.422315	-3.245016	1.062004
H	-4.891682	-4.089962	0.736140
C	-5.864084	1.199093	2.545277
H	-5.497807	1.323588	3.568716
H	-6.856870	0.748843	2.592819
H	-5.944182	2.199683	2.115934
C	-3.145795	-2.750053	-0.870286
C	-3.541878	-2.615053	-2.218906
C	-3.119351	-3.585692	-3.131798
H	-3.407238	-3.492259	-4.175492
C	-2.335847	-4.660615	-2.731937
H	-2.015921	-5.403098	-3.458052
C	-1.958398	-4.778334	-1.400881
H	-1.337379	-5.615179	-1.093257
C	-2.351128	-3.834764	-0.447447
C	-4.400237	-1.458748	-2.712764
H	-4.701344	-0.867224	-1.841626
C	-3.601436	-0.537865	-3.645230
H	-3.286352	-1.068013	-4.549912
H	-2.695919	-0.161370	-3.161758
H	-4.212992	0.317125	-3.955154
C	-5.686244	-1.941401	-3.399872
H	-6.309057	-1.085706	-3.682817
H	-6.282602	-2.589641	-2.749331
H	-5.467197	-2.502873	-4.314410
C	-1.893721	-3.996800	0.992711
H	-2.416797	-3.247601	1.597547
C	-2.243541	-5.375921	1.570684
H	-3.312214	-5.601535	1.487741
H	-1.971764	-5.420204	2.630949
H	-1.697515	-6.177287	1.061429
C	-0.390046	-3.722752	1.113907
H	-0.066253	-3.810244	2.155578
H	-0.137439	-2.717079	0.758378
H	0.189161	-4.439897	0.523273
C	-3.480600	2.208002	1.571201
C	-2.994291	2.644199	2.824011
C	-2.807729	4.016141	3.019982
H	-2.436436	4.367994	3.978815
C	-3.088074	4.936455	2.018296
H	-2.943343	5.998745	2.195660
C	-3.547315	4.490073	0.785879
H	-3.760907	5.211751	0.002764
C	-3.746940	3.129298	0.531713
C	-2.654873	1.689559	3.960644
H	-2.994665	0.688598	3.674007
C	-1.136707	1.614815	4.177288
H	-0.737881	2.591822	4.473899
H	-0.621228	1.295347	3.267782
H	-0.902169	0.902518	4.975971
C	-3.350409	2.064735	5.278638
H	-3.171722	1.290852	6.032831
H	-4.432739	2.181847	5.165267
H	-2.962469	3.006149	5.682422
C	-4.297769	2.702945	-0.823211
H	-4.154723	1.620286	-0.917333
C	-3.563506	3.359281	-1.998377
H	-3.719042	4.443626	-2.021571
H	-3.948406	2.960430	-2.942877
H	-2.489891	3.163572	-1.963441
C	-5.808608	2.971512	-0.916091
H	-6.021574	4.040638	-0.802934
H	-6.370509	2.435424	-0.145432
H	-6.194133	2.654833	-1.891365
H	0.000017	-0.000032	1.527169
C	-0.000181	-0.000080	-2.171643
C	-0.155086	1.146303	-2.946648
C	0.154618	-1.146388	-2.946775
C	-0.163267	1.193570	-4.337825
C	0.162596	-1.193520	-4.337959
C	-0.000387	0.000056	-5.040397
H	-0.291222	2.144499	-4.845664
H	0.290486	-2.144401	-4.845905
H	-0.000463	0.000105	-6.126777
F	0.314802	-2.338185	-2.299252
F	-0.315153	2.338042	-2.298984

Int-4_AlPdAl_WB97X.log

SCF (wB97x) = -3040.83123425
E(SCF)+ZPE(0 K) = -3039.453407
H(298 K) = -3039.371842
G(298 K) = -3039.570731
Lowest Frequency = 10.7366 cm^{-1}

Pd	-0.041632	0.028657	0.060525
Al	2.274845	0.344014	0.537806
N	3.468509	1.836359	0.174790
N	3.686004	-0.663089	1.431256
C	4.645284	2.065518	0.758924
C	5.273022	1.140626	1.602665
H	6.224530	1.437176	2.026906
C	4.859865	-0.175217	1.844181
C	5.377063	3.354324	0.478390
H	5.685889	3.396488	-0.570657
H	6.262070	3.444067	1.109814
H	4.722145	4.213705	0.647804
C	5.818634	-1.060306	2.604419
H	6.787181	-0.571085	2.716061
H	5.956417	-2.018242	2.096340
H	5.423313	-1.280276	3.600428
C	3.028989	2.745726	-0.860244
C	3.463477	2.519860	-2.180326
C	3.007591	3.378560	-3.180530
H	3.323206	3.213077	-4.208321
C	2.153460	4.432327	-2.888014
H	1.806648	5.089470	-3.681201
C	1.734906	4.636649	-1.582046
H	1.052101	5.453458	-1.359347
C	2.154559	3.799451	-0.548049
C	4.395619	1.372963	-2.555465
H	4.712491	0.869496	-1.634680
C	3.672628	0.336645	-3.424541
H	2.780286	-0.059975	-2.929015
H	4.344343	-0.498645	-3.658558
H	3.341816	0.776764	-4.371805
C	5.664838	1.871836	-3.257601
H	5.431729	2.336235	-4.222056
H	6.345085	1.034974	-3.450305
H	6.202592	2.611894	-2.654777
C	1.634181	4.039956	0.860854
H	2.129522	3.328696	1.532009
C	0.125685	3.777140	0.936613
H	-0.236624	3.910131	1.962169
H	-0.117719	2.756330	0.617617
H	-0.429538	4.471872	0.294889
C	1.968363	5.450488	1.361948
H	3.044599	5.654225	1.326528
H	1.634385	5.574110	2.397713
H	1.466641	6.216340	0.760065
C	3.425764	-2.071617	1.615050

C	2.979000	-2.556294	2.857104
C	2.777274	-3.931059	2.993981
H	2.434379	-4.326506	3.947393
C	2.996926	-4.798186	1.934860
H	2.838897	-5.866027	2.062355
C	3.395218	-4.296936	0.702174
H	3.534400	-4.981126	-0.129242
C	3.606515	-2.931294	0.510847
C	2.672471	-1.640875	4.036037
H	3.049028	-0.639876	3.798523
C	1.156570	-1.522568	4.245702
H	0.727479	-2.497206	4.509091
H	0.654413	-1.160801	3.342654
H	0.939028	-0.827290	5.065244
C	3.348900	-2.101989	5.333569
H	2.925935	-3.047734	5.689580
H	3.195315	-1.359451	6.123580
H	4.427124	-2.251215	5.212024
C	4.037788	-2.402376	-0.854999
H	3.571544	-1.417570	-0.991411
C	3.551612	-3.264863	-2.021983
H	4.066638	-4.232026	-2.057818
H	3.762478	-2.750402	-2.965479
H	2.472816	-3.430829	-1.966837
C	5.557122	-2.202482	-0.937125
H	5.920337	-1.469733	-0.209773
H	5.838182	-1.844499	-1.934504
H	6.079858	-3.149908	-0.759333
Al	-2.394047	-0.248848	0.366896
N	-3.556064	-1.769687	-0.004115
N	-3.841662	0.754939	1.206061
C	-4.797142	-1.958065	0.444916
C	-5.496626	-0.998199	1.186771
H	-6.502148	-1.259911	1.492590
C	-5.069175	0.299776	1.483026
C	-5.522477	-3.239073	0.115305
H	-5.701055	-3.305857	-0.963001
H	-6.480545	-3.288134	0.634544
H	-4.917513	-4.108347	0.387784
C	-6.087027	1.221330	2.111149
H	-5.688665	1.712236	3.001388
H	-6.992629	0.673804	2.375149
H	-6.351562	2.018325	1.408834
C	-2.994530	-2.765861	-0.890227
C	-3.244940	-2.660114	-2.271294
C	-2.656418	-3.596848	-3.120477
H	-2.820461	-3.522090	-4.192632
C	-1.861371	-4.619198	-2.620730
H	-1.411167	-5.339751	-3.298633
C	-1.637970	-4.713963	-1.255989
H	-1.004696	-5.509456	-0.869779
C	-2.188656	-3.789946	-0.367532
C	-4.112741	-1.552285	-2.856759
H	-4.679724	-1.094229	-2.037891
C	-3.244540	-0.456403	-3.486999
H	-2.635566	-0.858168	-4.305176
H	-2.547033	-0.025318	-2.761336
H	-3.871432	0.349235	-3.888049
C	-5.136485	-2.083860	-3.866375
H	-5.810527	-1.278996	-4.178142
H	-5.743592	-2.891932	-3.442951
H	-4.651654	-2.469552	-4.769518
C	-1.883532	-3.912587	1.117841
H	-2.461045	-3.146645	1.648142
C	-2.311752	-5.274870	1.677706
H	-3.374457	-5.474852	1.499916
H	-2.136593	-5.313428	2.758241
H	-1.740173	-6.090771	1.221573
C	-0.398352	-3.652006	1.394041
H	-0.192572	-3.713732	2.468993
H	-0.092140	-2.659745	1.040750
H	0.233439	-4.393370	0.891439
C	-3.572050	2.147571	1.485222
C	-3.074606	2.526836	2.746530
C	-2.892201	3.886790	3.001870
H	-2.524410	4.198992	3.977036
C	-3.159275	4.845118	2.034213
H	-3.008461	5.899039	2.252906
C	-3.600838	4.449130	0.780195
H	-3.787185	5.199869	0.015396
C	-3.816240	3.102772	0.480260
C	-2.719340	1.519473	3.833377
H	-2.875660	0.513983	3.427970
C	-1.240098	1.634496	4.223766
H	-1.027957	2.603935	4.690311
H	-0.594085	1.519731	3.348146
H	-0.978825	0.853153	4.946809
C	-3.607824	1.670177	5.075225
H	-3.328981	0.930452	5.833259
H	-4.668937	1.528031	4.846389
H	-3.493751	2.664042	5.522846
C	-4.298591	2.728120	-0.916334
H	-4.446192	1.642428	-0.950424
C	-3.249268	3.088505	-1.975339
H	-3.086219	4.172372	-2.009309
H	-3.588377	2.772632	-2.968641
H	-2.283185	2.613137	-1.780763
C	-5.646270	3.380573	-1.250079
H	-5.564428	4.472999	-1.271784
H	-6.421284	3.120351	-0.520981
H	-5.990161	3.055244	-2.237617
H	-0.168677	0.134787	1.665380
C	0.133135	-0.138068	-2.014936
C	0.069058	0.929999	-2.900824
C	0.317078	-1.349768	-2.668260
C	0.165381	0.849607	-4.284877
C	0.423809	-1.530608	-4.042253
C	0.345303	-0.405974	-4.860590
H	0.104730	1.753949	-4.883249
H	0.560756	-2.529514	-4.446355
H	0.426627	-0.507112	-5.939571
F	0.405977	-2.478860	-1.899610
F	-0.111101	2.180367	-2.377334

Int-5_AlPdAl_B3PW91.log

SCF (B3PW91) = -3040.49750542
E(SCF)+ZPE(0 K) = -3039.136743
H(298 K) =-3039.053025
G(298 K) = -3039.261039
Lowest Frequency = 10.2878 cm^{-1}

Pd	0.024413	-0.050313	0.390491
Al	2.353611	-0.535186	0.123328
N	4.053374	0.493590	0.164935
N	3.281551	-2.194074	-0.421733
C	5.283825	-0.026022	0.069055
C	5.531076	-1.398909	-0.069605
H	6.572082	-1.698129	-0.060749
C	4.603212	-2.408160	-0.349052
C	6.495067	0.874124	0.035826
H	7.397249	0.314642	0.290869
H	6.398865	1.732445	0.700943
H	6.623164	1.271362	-0.977496
C	5.158431	-3.782359	-0.629135
H	6.211356	-3.838716	-0.347691
H	5.072080	-4.025845	-1.692526
H	4.599900	-4.549929	-0.088522
C	3.937034	1.929938	0.256158
C	3.827251	2.693753	-0.926226
C	3.758687	4.086012	-0.814721
H	3.672074	4.685791	-1.716752
C	3.801706	4.714293	0.422653
C	3.881878	3.946973	1.578116
H	3.897991	4.440806	2.545534
C	3.934487	2.550303	1.526393
C	2.461298	-3.257504	-0.951099
C	1.790314	-4.141395	-0.079937
C	1.013777	-5.162072	-0.639322
H	0.494558	-5.851203	0.021095
C	0.889002	-5.308497	-2.013997

C	1.540288	-4.418289	-2.859138
H	1.430502	-4.525164	-3.935135
C	2.332652	-3.383994	-2.353891
C	-0.258124	0.045765	2.485533
C	-1.372504	0.608869	3.095306
C	0.634973	-0.419314	3.456231
C	-1.632107	0.759908	4.449745
C	0.466653	-0.344659	4.836141
C	-0.680954	0.263792	5.338389
H	-2.547450	1.232972	4.789503
H	1.230983	-0.746140	5.494114
H	-0.834901	0.347388	6.409988
H	0.119331	-0.074424	-1.208431
F	1.807806	-1.005661	3.059874
F	-2.413631	1.095430	2.274626
C	1.870506	-4.028303	1.433820
H	2.498997	-3.166247	1.676334
C	2.995996	-2.422927	-3.331355
H	3.663731	-1.768397	-2.762229
C	3.756087	2.065417	-2.310185
H	3.914471	0.987136	-2.199808
C	3.992672	1.763622	2.829042
H	3.783334	0.715485	2.595901
Al	-2.262041	0.515852	0.041418
N	-3.123893	2.195136	-0.515126
N	-3.964718	-0.499477	0.067175
C	-5.175878	0.065207	0.005971
C	-5.380699	1.451150	-0.089443
C	-4.438196	2.437746	-0.398476
C	-4.966463	3.826709	-0.656138
C	-2.832459	2.259778	-3.432337
H	-3.435563	1.585955	-2.815797
H	-4.778293	4.132446	-1.689271
H	-6.039578	3.877090	-0.465822
H	-4.458386	4.556412	-0.020066
H	-6.338639	-1.696656	0.543221
C	-6.420890	-0.786765	-0.049942
H	-7.291811	-0.215057	0.276809
H	-6.596912	-1.097686	-1.086164
H	-6.412069	1.779089	-0.045685
C	-2.300973	3.228105	-1.101896
C	-2.165768	3.269425	-2.508791
C	-1.377020	4.274765	-3.075607
C	-0.730611	5.217455	-2.286382
C	-0.863284	5.157670	-0.905830
C	-1.643232	4.174820	-0.287285
H	-1.267899	4.316309	-4.156291
C	-1.733496	4.161862	1.229667
H	-0.349328	5.888460	-0.287453
H	-2.444538	3.383314	1.517367
C	-3.891329	-1.943586	0.084033
C	-3.733698	-2.641309	-1.133576
C	-3.562085	-1.943245	-2.474090
C	-3.986016	-2.642537	1.310389
C	-3.980094	-4.040886	1.277000
C	-3.858209	-4.740743	0.083778
C	-3.721512	-4.039491	-1.106040
H	-3.604439	-4.585264	-2.038459
H	-3.599026	-0.862452	-2.299490
C	-4.095017	-1.950851	2.663275
H	-3.946844	-0.879103	2.505699
H	-4.069372	-4.590255	2.210178
H	3.762873	5.798449	0.487988
H	0.282092	-6.110176	-2.426311
H	-0.124638	5.993945	-2.745781
H	-3.859556	-5.827518	0.083292
C	2.917636	2.227471	3.819999
H	3.109541	3.243168	4.183714
H	1.924054	2.206699	3.366897
H	2.902143	1.566959	4.692263
C	5.378683	1.833418	3.489652
H	5.653655	2.870252	3.715446
H	5.377270	1.276061	4.432823
H	6.163698	1.409468	2.857420
C	4.848266	2.594751	-3.250979
H	4.814121	2.064519	-4.208899
H	4.714509	3.661007	-3.463359
H	5.850754	2.466864	-2.829946
C	2.363691	2.270494	-2.924894
H	1.582059	1.843345	-2.289877
H	2.143426	3.335645	-3.052777
H	2.305643	1.794948	-3.910161
C	0.483925	-3.762446	2.033221
H	0.558774	-3.620965	3.116158
H	0.042316	-2.859039	1.601081
H	-0.198622	-4.599332	1.848958
C	2.512386	-5.268198	2.074201
H	3.519950	-5.457727	1.689461
H	2.588533	-5.136661	3.158981
H	1.914523	-6.168189	1.891842
C	1.945002	-1.532180	-4.010379
H	1.358262	-0.978603	-3.271554
H	2.425985	-0.812689	-4.682698
H	1.249678	-2.133402	-4.606710
C	3.849866	-3.144506	-4.384026
H	4.368954	-2.415193	-5.015458
H	4.606040	-3.791513	-3.927825
H	3.237338	-3.768679	-5.043506
C	-1.781861	1.407913	-4.158438
H	-1.143871	2.027731	-4.798142
H	-1.137964	0.886116	-3.444700
H	-2.267505	0.662434	-4.797943
C	-3.778863	2.928315	-4.440382
H	-4.552307	3.522692	-3.943634
H	-3.236961	3.593856	-5.121122
H	-4.280423	2.170354	-5.052095
C	-2.242840	5.493852	1.800015
H	-3.211539	5.782857	1.378602
H	-2.359368	5.417833	2.886498
H	-1.541888	6.312513	1.603450
C	-0.375059	3.796777	1.844801
H	0.381269	4.553479	1.608948
H	-0.455214	3.726402	2.935051
H	-0.016565	2.833734	1.467515
C	-2.998583	-2.434782	3.623501
H	-2.003668	-2.298288	3.193908
H	-3.038672	-1.868247	4.558680
H	-3.122678	-3.494421	3.873093
C	-5.474972	-2.148044	3.311459
H	-5.683224	-3.210650	3.480602
H	-5.510434	-1.645200	4.284127
H	-6.286749	-1.743074	2.700754
C	-4.692845	-2.288650	-3.454238
H	-4.691733	-3.354891	-3.705964
H	-5.678500	-2.048796	-3.042754
H	-4.572460	-1.729977	-4.388858
C	-2.191063	-2.267326	-3.084012
H	-1.382080	-1.964304	-2.413390
H	-2.086337	-3.340249	-3.277392
H	-2.063077	-1.743368	-4.037080

Int-5_AlPdAl_WB97X.log

SCF (wB97x) = -3040.83704547
E(SCF)+ZPE(0 K) = -3039.459937
H(298 K) = -3039.378283
G(298 K) = -3039.576839
Lowest Frequency = 17.6721 cm^{-1}

Pd	-0.017383	0.059786	0.565934
Al	-2.311406	0.640069	0.274241
N	-4.014637	-0.315305	0.190065
N	-3.132101	2.297653	-0.361304
C	-5.222980	0.237827	0.083424
C	-5.422102	1.616841	-0.076328
H	-6.449570	1.960407	-0.091651
C	-4.436179	2.576233	-0.346649
C	-6.450593	-0.638593	0.127073
H	-7.355035	-0.052865	-0.042069
H	-6.528869	-1.139368	1.096617
H	-6.386642	-1.426627	-0.629157

C	-4.907029	3.968474	-0.690935
H	-5.948360	4.107025	-0.395614
H	-4.828485	4.139716	-1.769701
H	-4.285702	4.723414	-0.202110
C	-3.897061	-1.752593	0.262641
C	-3.593959	-2.469542	-0.910746
C	-3.471832	-3.857970	-0.827551
H	-3.242419	-4.425681	-1.726478
C	-3.625658	-4.522808	0.380116
C	-3.882613	-3.796680	1.534714
H	-3.970195	-4.316625	2.486095
C	-4.013167	-2.407847	1.503476
C	-2.217629	3.267351	-0.915989
C	-1.401027	4.041080	-0.072551
C	-0.509729	4.943226	-0.660289
H	0.129533	5.547040	-0.020430
C	-0.417076	5.075299	-2.036615
C	-1.211180	4.284460	-2.856561
H	-1.117154	4.370282	-3.936804
C	-2.112383	3.366571	-2.319449
C	0.463461	-0.042613	2.639581
C	1.588332	-0.675673	3.139587
C	-0.300043	0.468006	3.692366
C	1.967371	-0.870018	4.457578
C	-0.014752	0.347607	5.047506
C	1.131506	-0.340100	5.433990
H	2.882942	-1.399250	4.700965
H	-0.690598	0.782966	5.777307
H	1.374802	-0.455536	6.486196
H	-0.112885	0.032619	-1.037248
F	-1.448271	1.151480	3.410564
F	2.535820	-1.198186	2.211572
C	-1.446600	3.944195	1.445343
H	-2.098707	3.109520	1.720976
C	-2.896379	2.462047	-3.263626
H	-3.674159	1.947692	-2.689299
C	-3.391720	-1.792785	-2.260598
H	-3.382555	-0.708356	-2.101676
C	-4.202040	-1.655892	2.813621
H	-4.366163	-0.598115	2.581115
Al	2.169865	-0.629141	0.077935
N	2.906654	-2.289829	-0.568336
N	3.842475	0.353807	-0.060734
C	5.030774	-0.217326	-0.224108
C	5.206734	-1.611676	-0.325692
C	4.215005	-2.569481	-0.559326
C	4.670272	-3.972920	-0.876370
C	2.598312	-2.372808	-3.469113
H	3.348120	-1.825820	-2.887596
H	4.499882	-4.200592	-1.933504
H	5.732729	-4.094342	-0.660423
H	4.096518	-4.703042	-0.298472
H	6.177408	1.622226	0.014875
C	6.279513	0.616716	-0.390959
H	7.131431	0.117316	0.076881
H	6.499138	0.707429	-1.460936
H	6.232326	-1.960426	-0.366764
C	1.991437	-3.257865	-1.130247
C	1.852853	-3.315806	-2.532790
C	0.958269	-4.235408	-3.078813
C	0.200294	-5.069458	-2.267873
C	0.327286	-4.982666	-0.890707
C	1.219300	-4.086026	-0.296221
H	0.841170	-4.288157	-4.159007
C	1.305736	-4.043197	1.221320
H	-0.281869	-5.620772	-0.254286
H	2.051516	-3.294823	1.498544
C	3.723807	1.795115	-0.029938
C	3.382682	2.478705	-1.212060
C	3.108901	1.767949	-2.530719
C	3.891891	2.486774	1.186703
C	3.793689	3.879309	1.171777
C	3.502293	4.572273	0.005725
C	3.284605	3.870569	-1.170070
H	3.028225	4.412286	-2.077482
H	3.087575	0.688217	-2.342422
C	4.172397	1.785419	2.511012
H	3.990922	0.715345	2.372201
H	3.939646	4.430379	2.097470
H	-3.530584	-5.605031	0.424782
H	0.282070	5.785261	-2.472178
H	-0.495344	-5.778431	-2.710613
H	3.431895	5.657143	0.017291
C	-2.922053	-1.752267	3.654600
H	-2.751100	-2.785777	3.979696
H	-2.049297	-1.431484	3.079320
H	-2.993574	-1.121609	4.547566
C	-5.411629	-2.153191	3.613863
H	-5.278596	-3.192620	3.933727
H	-5.541921	-1.547021	4.516483
H	-6.341097	-2.102896	3.036153
C	-4.550362	-2.099926	-3.217402
H	-4.403190	-1.588213	-4.175277
H	-4.619152	-3.175302	-3.418450
H	-5.512290	-1.774569	-2.805833
C	-2.045134	-2.178279	-2.883855
H	-1.216368	-1.955859	-2.202464
H	-2.003597	-3.245844	-3.126219
H	-1.884766	-1.621413	-3.814429
C	-0.061469	3.638194	2.026115
H	-0.134457	3.479187	3.107466
H	0.351954	2.727396	1.577661
H	0.644560	4.458690	1.849979
C	-2.037350	5.217707	2.063236
H	-3.050946	5.414950	1.695934
H	-2.085768	5.125713	3.153487
H	-1.421254	6.093189	1.826951
C	-1.971675	1.385114	-3.847898
H	-1.476408	0.810861	-3.056491
H	-2.538794	0.690317	-4.479733
H	-1.187754	1.841458	-4.464495
C	-3.596739	3.237682	-4.385118
H	-4.218156	2.561234	-4.981438
H	-4.241037	4.031517	-3.991638
H	-2.876072	3.703673	-5.065831
C	1.626195	-1.340564	-4.055724
H	0.863056	-1.832108	-4.671168
H	1.108724	-0.787160	-3.263654
H	2.161538	-0.623229	-4.689908
C	3.341143	-3.114187	-4.586558
H	4.032603	-3.864386	-4.187744
H	2.647708	-3.628002	-5.261179
H	3.920830	-2.407052	-5.189467
C	1.766319	-5.387784	1.797858
H	2.736058	-5.694822	1.389781
H	1.864454	-5.319647	2.886563
H	1.046502	-6.184684	1.578662
C	-0.034019	-3.612710	1.834340
H	-0.818766	-4.354698	1.642693
H	0.063800	-3.492985	2.920020
H	-0.367977	-2.653858	1.420187
C	3.228222	2.272419	3.618449
H	2.179342	2.172400	3.323479
H	3.377186	1.678089	4.525684
H	3.416721	3.320985	3.875567
C	5.631271	1.966576	2.952875
H	5.870116	3.029323	3.078437
H	5.801362	1.469870	3.914206
H	6.339046	1.547824	2.231018
C	4.225455	2.030837	-3.548511
H	4.285023	3.096344	-3.798917
H	5.204058	1.725929	-3.161799
H	4.038570	1.478195	-4.476373
C	1.740074	2.161241	-3.098069
H	0.943289	1.955710	-2.374984
H	1.699472	3.226608	-3.350537
H	1.531347	1.596270	-4.013562

Int-6_AlPdAl_B3PW91.log

SCF (B3PW91) = -3040.50029697

E(SCF)+ZPE(0 K) = -3039.138896
H(298 K) = -3039.054991
G(298 K) = -3039.263870
Lowest Frequency = 8.8154 cm^{-1}

Pd	-0.464844	0.217731	0.608980
Al	2.170823	0.332809	0.233530
N	2.986872	2.049336	-0.351662
N	3.525983	-0.031309	1.629287
C	3.578372	2.803454	0.570705
C	3.841726	2.358123	1.882737
H	4.206083	3.119198	2.562966
C	3.918641	1.042182	2.343856
C	4.042957	4.209546	0.271939
H	3.992948	4.453791	-0.788225
H	5.070031	4.340231	0.623597
H	3.420404	4.924873	0.819864
C	4.511265	0.849909	3.719818
H	5.389060	0.200008	3.698241
H	3.781692	0.366543	4.376209
H	4.793544	1.808628	4.157253
C	2.753647	2.589761	-1.666370
C	3.565241	2.165131	-2.740738
C	3.301311	2.673349	-4.015980
H	3.921751	2.360921	-4.850782
C	2.264552	3.571650	-4.234947
H	2.072616	3.952609	-5.234576
C	1.479444	3.984080	-3.166612
H	0.670233	4.688670	-3.339420
C	1.702040	3.512365	-1.869015
C	4.749604	1.235003	-2.532458
H	4.584526	0.702768	-1.590350
C	4.890528	0.178777	-3.633815
H	3.961264	-0.380733	-3.769851
H	5.679813	-0.532517	-3.370582
H	5.167198	0.623280	-4.596434
C	6.047612	2.045652	-2.389894
H	6.249574	2.618122	-3.302555
H	6.902583	1.383600	-2.212654
H	5.992235	2.754843	-1.558431
C	0.803957	4.016966	-0.747556
H	1.179687	3.623831	0.201926
C	-0.630668	3.498945	-0.900480
H	-1.262239	3.894451	-0.097950
H	-0.666075	2.403899	-0.834398
H	-1.062364	3.807803	-1.859702
C	0.805487	5.551787	-0.659158
H	1.817118	5.963815	-0.593573
H	0.246390	5.878028	0.224592
H	0.323649	6.004090	-1.533070
C	4.031277	-1.305683	2.081534
C	3.282703	-2.106138	2.968005
C	3.831438	-3.318726	3.401710
H	3.256859	-3.946114	4.077984
C	5.094223	-3.728706	2.998800
H	5.506036	-4.669099	3.355560
C	5.827200	-2.925264	2.133853
H	6.816404	-3.245664	1.817269
C	5.316520	-1.716582	1.654651
C	1.904718	-1.708746	3.470702
H	1.701433	-0.684696	3.138471
C	0.828169	-2.607852	2.852179
H	0.977859	-3.655748	3.138813
H	0.848442	-2.545587	1.761421
H	-0.169158	-2.297774	3.178852
C	1.814283	-1.737222	5.003660
H	1.903772	-2.756443	5.395602
H	0.847053	-1.344533	5.332514
H	2.598626	-1.136251	5.475333
C	6.161991	-0.893822	0.692515
H	5.531661	-0.083676	0.313707
C	6.628569	-1.725507	-0.510509
H	7.335545	-2.507563	-0.211885
H	7.142709	-1.086784	-1.237027
H	5.783481	-2.207988	-1.005666
C	7.370588	-0.254108	1.393294
H	7.067129	0.421031	2.198532
H	7.964860	0.327017	0.679220
H	8.025085	-1.018881	1.826896
Al	-2.773070	-0.425015	0.310049
N	-3.924624	-1.734005	-0.651015
N	-4.354901	0.307178	1.278647
C	-5.196724	-2.041059	-0.356172
C	-5.931061	-1.400096	0.648803
H	-6.933389	-1.772666	0.820830
C	-5.560480	-0.264094	1.379542
C	-5.908969	-3.123444	-1.129392
H	-5.855034	-2.936743	-2.205302
H	-6.956809	-3.187831	-0.832458
H	-5.438406	-4.096400	-0.960311
C	-6.612512	0.343619	2.273075
H	-6.192883	0.642972	3.235746
H	-7.431564	-0.358596	2.438908
H	-7.026779	1.248042	1.815724
C	-3.318601	-2.416473	-1.769249
C	-3.316056	-1.792778	-3.036174
C	-2.756111	-2.483958	-4.115860
H	-2.760683	-2.021802	-5.099624
C	-2.195382	-3.743732	-3.954925
H	-1.766607	-4.264866	-4.806619
C	-2.177411	-4.330568	-2.695673
H	-1.719755	-5.307651	-2.569085
C	-2.734608	-3.689588	-1.585864
C	-3.903051	-0.409285	-3.275615
H	-4.221163	-0.008505	-2.307071
C	-2.854177	0.552188	-3.851524
H	-2.537375	0.243602	-4.854125
H	-1.964748	0.598573	-3.217582
H	-3.269270	1.562045	-3.937278
C	-5.145670	-0.464237	-4.176790
H	-5.563920	0.538902	-4.314928
H	-5.930016	-1.098446	-3.751540
H	-4.899648	-0.860239	-5.168317
C	-2.654287	-4.359250	-0.222493
H	-3.314379	-3.813525	0.460253
C	-3.116760	-5.822661	-0.239573
H	-4.120399	-5.935937	-0.663111
H	-3.134205	-6.222748	0.779754
H	-2.439984	-6.455919	-0.822979
C	-1.227539	-4.247762	0.332104
H	-1.167701	-4.677027	1.338286
H	-0.909240	-3.202200	0.382774
H	-0.517187	-4.782394	-0.308216
C	-4.145426	1.580616	1.921202
C	-3.509869	1.641743	3.179474
C	-3.264683	2.900577	3.738393
H	-2.775091	2.962327	4.706630
C	-3.631275	4.068310	3.082883
H	-3.426664	5.035911	3.533167
C	-4.269238	3.991408	1.850414
H	-4.561220	4.906788	1.342573
C	-4.544550	2.759548	1.250157
C	-3.109755	0.399681	3.960365
H	-3.359835	-0.474186	3.348581
C	-1.599894	0.359656	4.221773
H	-1.275937	1.206840	4.836930
H	-1.041563	0.384931	3.276709
H	-1.337651	-0.559254	4.757627
C	-3.892401	0.283082	5.277854
H	-3.624535	-0.642807	5.798582
H	-4.975222	0.274603	5.116146
H	-3.665960	1.117789	5.950412
C	-5.248735	2.735058	-0.100355
H	-5.527002	1.699056	-0.318381
C	-4.310890	3.200549	-1.222291
H	-3.994084	4.237133	-1.063968
H	-4.815740	3.148485	-2.193622
H	-3.408042	2.585042	-1.268643
C	-6.540279	3.565950	-0.103475
H	-6.334537	4.635572	0.010766
H	-7.218437	3.274405	0.705079

H	-7.071179	3.436930	-1.052852
H	0.919496	0.946628	1.264604
C	2.060678	-0.929011	-1.343372
C	1.274831	-0.605561	-2.452048
C	2.643708	-2.190116	-1.446676
C	1.099057	-1.405457	-3.574279
C	2.514616	-3.052630	-2.529585
C	1.733155	-2.644735	-3.606208
H	0.469509	-1.060247	-4.386093
H	3.017118	-4.013761	-2.511994
H	1.612548	-3.294600	-4.467825
F	3.402557	-2.634021	-0.417096
F	0.627678	0.583082	-2.452628

Int-7_AlPdAl_B3PW91.log

SCF (B3PW91) = -3040.54210556
E(SCF)+ZPE(0 K)= -3039.180918
H(298 K) = -3039.097166
G(298 K) = -3039.304766
Lowest Frequency = 8.8858 cm^{-1}

Pd	0.139112	0.010608	-0.633539
Al	-2.153735	-0.368646	-0.019344
N	-3.733352	0.771957	-0.253323
N	-3.173051	-1.976735	0.404358
C	-4.990628	0.308990	-0.302115
C	-5.321087	-1.048733	-0.193949
H	-6.369150	-1.290604	-0.319931
C	-4.493743	-2.106583	0.192919
C	-6.152214	1.265245	-0.403253
H	-7.046296	0.747039	-0.754285
H	-5.942802	2.112279	-1.055431
H	-6.369945	1.676974	0.588677
C	-5.166354	-3.432558	0.441033
H	-6.169556	-3.440108	0.011899
H	-5.251561	-3.623833	1.515504
H	-4.588569	-4.258285	0.022088
C	-3.546512	2.208389	-0.292294
C	-3.495239	2.924099	0.924171
C	-3.344993	4.313222	0.875598
H	-3.300153	4.873584	1.805415
C	-3.254910	4.987790	-0.333928
C	-3.301337	4.269661	-1.521204
H	-3.227591	4.798622	-2.467286
C	-3.440403	2.877689	-1.533708
C	-2.490447	-3.086711	1.033876
C	-1.813007	-4.046687	0.251487
C	-1.192244	-5.115249	0.908426
H	-0.670749	-5.863192	0.317980
C	-1.228554	-5.239266	2.290351
C	-1.888742	-4.278975	3.047226
H	-1.908329	-4.372546	4.129672
C	-2.530486	-3.194035	2.443799
C	-0.835278	-0.518680	-2.360099
C	0.446897	-0.185204	-2.648566
C	-1.675837	-0.923446	-3.367391
C	0.980917	-0.183899	-3.927819
C	-1.206443	-0.975033	-4.681223
C	0.112911	-0.597252	-4.951617
H	2.002030	0.117929	-4.131554
H	-1.873497	-1.296546	-5.475440
H	0.466874	-0.627994	-5.979810
H	0.948476	0.422472	0.705475
F	-2.974538	-1.273235	-3.123237
F	3.219688	1.054541	-2.083202
C	-1.742823	-3.982608	-1.265972
H	-2.229134	-3.058930	-1.589333
C	-3.225642	-2.168685	3.329718
H	-3.795869	-1.492665	2.684459
C	-3.585957	2.253236	2.286706
H	-3.744485	1.180819	2.127885
C	-3.490040	2.168410	-2.880288
H	-3.506832	1.090949	-2.698544
Al	2.542227	0.620384	-0.576837
N	3.233908	2.089814	0.513275
N	3.850006	-0.712151	0.039397
C	5.068215	-0.295425	0.398903
C	5.413902	1.056972	0.576101
C	4.559739	2.158276	0.702172
C	5.187967	3.466378	1.112969
C	2.523774	2.247172	3.357380
H	3.198777	1.546375	2.857464
H	4.822848	3.796061	2.089036
H	6.274577	3.379959	1.158330
H	4.923630	4.252507	0.399624
H	6.039905	-2.241543	0.220878
C	6.161788	-1.285120	0.727757
H	7.137694	-0.862875	0.476734
H	6.160204	-1.480350	1.805888
H	6.463524	1.249475	0.762682
C	2.426246	3.191702	0.959938
C	2.062808	3.268305	2.325417
C	1.254111	4.329449	2.742086
C	0.810576	5.295684	1.847034
C	1.185007	5.214357	0.512536
C	1.999220	4.177701	0.043574
H	0.968472	4.398824	3.788800
C	2.407496	4.163937	-1.421844
H	0.846196	5.975464	-0.185325
H	3.106029	3.336927	-1.574925
C	3.611427	-2.131760	-0.030561
C	3.201295	-2.825178	1.128172
C	2.930013	-2.125897	2.451048
C	3.819235	-2.814359	-1.253590
C	3.654142	-4.203339	-1.266591
C	3.273732	-4.903028	-0.127472
C	3.036601	-4.212182	1.052764
H	2.722427	-4.756665	1.939286
H	3.137524	-1.059948	2.314045
C	4.247845	-2.101993	-2.531619
H	3.992931	-1.042786	-2.431924
H	3.827994	-4.747118	-2.190338
H	-3.146579	6.068816	-0.351818
H	-0.743109	-6.080687	2.777580
H	0.182348	6.113287	2.190724
H	3.157495	-5.983196	-0.163429
C	-2.245943	2.480586	-3.723879
H	-2.202816	3.540513	-3.997849
H	-1.327475	2.228223	-3.190588
H	-2.264173	1.899530	-4.650884
C	-4.754907	2.527861	-3.677918
H	-4.794952	3.601178	-3.895094
H	-4.757517	1.995990	-4.635360
H	-5.675492	2.260980	-3.151005
C	-4.771757	2.771933	3.113386
H	-4.844309	2.225525	4.060034
H	-4.657047	3.833901	3.355594
H	-5.722864	2.656582	2.584037
C	-2.271232	2.420911	3.059398
H	-1.426239	2.024647	2.490204
H	-2.064254	3.477041	3.261171
H	-2.318416	1.898795	4.020898
C	-0.289127	-3.943250	-1.755813
H	-0.261733	-3.792967	-2.839845
H	0.267812	-3.126862	-1.289751
H	0.235457	-4.878551	-1.533738
C	-2.490864	-5.152124	-1.925672
H	-3.548177	-5.182747	-1.644205
H	-2.441377	-5.061935	-3.015963
H	-2.046151	-6.115422	-1.651717
C	-2.194453	-1.322200	4.089818
H	-1.516043	-0.807963	3.402712
H	-2.693890	-0.569293	4.709492
H	-1.584767	-1.948668	4.749903
C	-4.219846	-2.808402	4.309655
H	-4.762232	-2.031263	4.858793
H	-4.957101	-3.434504	3.797123
H	-3.713123	-3.436014	5.050312
C	1.343549	1.434607	3.905613
H	0.626488	2.080105	4.424856

H	0.814879	0.918185	3.100106
H	1.695363	0.685072	4.623576
C	3.298488	2.897530	4.513867
H	4.154216	3.481911	4.161924
H	2.659938	3.569041	5.098327
H	3.677005	2.128860	5.196726
C	3.120100	5.460904	-1.832976
H	3.986643	5.671892	-1.197449
H	3.473332	5.383983	-2.866888
H	2.452516	6.328241	-1.777223
C	1.198687	3.902152	-2.329543
H	0.447623	4.694639	-2.233943
H	1.511572	3.852388	-3.377859
H	0.719020	2.951837	-2.079088
C	3.515109	-2.635922	-3.769514
H	2.431689	-2.635058	-3.627910
H	3.741944	-2.002390	-4.633450
H	3.829190	-3.653714	-4.028462
C	5.766148	-2.191933	-2.757721
H	6.094685	-3.236704	-2.813778
H	6.035532	-1.703917	-3.700835
H	6.333028	-1.701610	-1.962837
C	3.837864	-2.644341	3.576537
H	3.629824	-3.695637	3.804858
H	4.898524	-2.569108	3.318318
H	3.672225	-2.070612	4.495098
C	1.454987	-2.263581	2.849079
H	0.800446	-1.839512	2.082536
H	1.174734	-3.312829	2.987466
H	1.263751	-1.739850	3.791779

Int-7_AlPdAl_WB97X.log

SCF (wB97x) = -3040.88964047
E(SCF)+ZPE(0 K) = -3039.510914
H(298 K) = -3039.429609
G(298 K) = -3039.627292
Lowest Frequency = 12.9209 cm^{-1}

Pd	-0.130740	-0.008569	0.639193
Al	2.130199	-0.328876	0.009663
N	3.621394	0.869570	0.248886
N	3.197563	-1.841042	-0.490015
C	4.902731	0.502419	0.221749
C	5.318098	-0.827537	0.048773
H	6.383844	-1.009818	0.116794
C	4.526311	-1.913439	-0.333680
C	5.991690	1.543512	0.298574
H	6.936274	1.091060	0.604962
H	5.740549	2.362192	0.972353
H	6.130981	1.979301	-0.697749
C	5.239708	-3.204002	-0.651359
H	6.263960	-3.179195	-0.276997
H	5.269216	-3.362880	-1.734760
H	4.715191	-4.059397	-0.219111
C	3.293314	2.275454	0.370540
C	3.079521	3.025441	-0.801038
C	2.754321	4.376678	-0.674790
H	2.576606	4.966887	-1.570898
C	2.648561	4.975931	0.570920
C	2.849164	4.218138	1.715565
H	2.753569	4.689508	2.690688
C	3.158735	2.858443	1.646692
C	2.522471	-2.945896	-1.137790
C	1.824068	-3.899365	-0.377205
C	1.176906	-4.937106	-1.054689
H	0.628316	-5.680135	-0.480625
C	1.220006	-5.034549	-2.436207
C	1.905706	-4.076967	-3.173360
H	1.922955	-4.149431	-4.257949
C	2.561093	-3.019025	-2.546104
C	0.802857	-0.585321	2.354112
C	-0.487330	-0.289604	2.637319
C	1.645591	-1.014301	3.344242
C	-1.024458	-0.357980	3.913332
C	1.171701	-1.140161	4.647288
C	-0.157865	-0.803289	4.921118
H	-2.053549	-0.082908	4.124537
H	1.839861	-1.481834	5.432472
H	-0.519854	-0.891250	5.943299
H	-0.933008	0.448947	-0.694697
F	2.956213	-1.314000	3.082751
F	-3.205464	0.982307	2.175949
C	1.761428	-3.861330	1.142097
H	2.225589	-2.932482	1.481901
C	3.252538	-1.957357	-3.394102
H	3.934521	-1.389652	-2.751845
C	3.166467	2.426025	-2.198835
H	3.428620	1.364694	-2.107547
C	3.347098	2.084407	2.946864
H	3.418773	1.019068	2.708433
Al	-2.508814	0.574764	0.670795
N	-3.228521	1.995426	-0.428392
N	-3.728824	-0.790474	0.019911
C	-4.960692	-0.436174	-0.332281
C	-5.374167	0.904679	-0.475845
C	-4.552304	2.029357	-0.608246
C	-5.210674	3.319160	-1.034511
C	-2.679806	2.124104	-3.306339
H	-3.370808	1.455380	-2.782804
H	-4.962010	3.560083	-2.072222
H	-6.295729	3.245934	-0.946671
H	-4.852671	4.149783	-0.418998
H	-5.789672	-2.456534	-0.261079
C	-6.003997	-1.474583	-0.681895
H	-6.986346	-1.140264	-0.338932
H	-6.056788	-1.575392	-1.771151
H	-6.432574	1.058318	-0.652361
C	-2.430595	3.081718	-0.927488
C	-2.158947	3.154236	-2.310363
C	-1.358303	4.195280	-2.779442
C	-0.818582	5.136816	-1.913046
C	-1.085073	5.047055	-0.555592
C	-1.896434	4.033547	-0.039622
H	-1.142310	4.261916	-3.843992
C	-2.170684	3.999174	1.456744
H	-0.659355	5.781253	0.125206
H	-2.863407	3.179862	1.667178
C	-3.373530	-2.187149	0.039313
C	-2.868580	-2.788561	-1.126931
C	-2.595642	-2.003470	-2.402393
C	-3.526748	-2.922086	1.234194
C	-3.259598	-4.291400	1.199413
C	-2.817217	-4.913445	0.038730
C	-2.602757	-4.160189	-1.104699
H	-2.219701	-4.642120	-2.001585
H	-2.738166	-0.940109	-2.179904
C	-3.982032	-2.269344	2.536184
H	-3.700052	-1.212081	2.503591
H	-3.390156	-4.882107	2.101851
H	2.402044	6.031722	0.650911
H	0.714227	-5.853001	-2.942574
H	-0.192460	5.938997	-2.297202
H	-2.620235	-5.982995	0.034837
C	2.153087	2.268898	3.893255
H	2.071712	3.305788	4.239165
H	1.210172	1.992912	3.413323
H	2.275576	1.634261	4.777104
C	4.641063	2.492136	3.666610
H	4.632837	3.560720	3.911306
H	4.741600	1.935316	4.604186
H	5.534519	2.292918	3.066877
C	4.264803	3.091732	-3.036882
H	4.341944	2.609356	-4.017450
H	4.046295	4.152116	-3.204745
H	5.243647	3.029084	-2.549210
C	1.810326	2.513943	-2.908103
H	1.022336	2.034009	-2.317661
H	1.511838	3.556786	-3.060715
H	1.855638	2.029180	-3.890230

C	0.313664	-3.878234	1.648107
H	0.290381	-3.686294	2.727234
H	-0.294410	-3.110932	1.157631
H	-0.163264	-4.848453	1.467986
C	2.558238	-5.017171	1.761513
H	3.613271	-4.992530	1.467516
H	2.515211	-4.961328	2.854350
H	2.148443	-5.987436	1.457599
C	2.219769	-0.969081	-3.952029
H	1.634222	-0.499633	-3.152363
H	2.713644	-0.176706	-4.527128
H	1.513271	-1.482558	-4.614723
C	4.095926	-2.553187	-4.526433
H	4.661174	-1.762348	-5.030348
H	4.809205	-3.296060	-4.153070
H	3.474020	-3.040488	-5.284826
C	-1.530166	1.263346	-3.844124
H	-0.809146	1.876061	-4.399147
H	-0.995463	0.766157	-3.027956
H	-1.913602	0.494580	-4.526489
C	-3.445502	2.768591	-4.468869
H	-4.266272	3.404126	-4.120299
H	-2.787536	3.389662	-5.086903
H	-3.870485	1.994868	-5.117368
C	-2.835176	5.293808	1.939694
H	-3.765818	5.494578	1.397268
H	-3.075211	5.220073	3.005592
H	-2.177931	6.160898	1.805558
C	-0.881722	3.718682	2.238836
H	-0.152608	4.529030	2.113865
H	-1.096461	3.609688	3.307851
H	-0.406310	2.793193	1.894253
C	-3.302582	-2.887358	3.763388
H	-2.216288	-2.937914	3.639408
H	-3.511613	-2.274874	4.647033
H	-3.676951	-3.896095	3.974535
C	-5.506545	-2.327516	2.705855
H	-5.866650	-3.362408	2.652457
H	-5.792693	-1.919051	3.680981
H	-6.027330	-1.743764	1.942170
C	-3.565533	-2.389507	-3.526030
H	-3.441177	-3.442079	-3.806564
H	-4.610134	-2.246882	-3.232147
H	-3.379147	-1.781650	-4.418966
C	-1.146184	-2.192749	-2.861685
H	-0.443771	-1.899640	-2.073731
H	-0.939658	-3.235019	-3.127316
H	-0.944473	-1.579697	-3.747219

TS-1_AlPdAl _B3PW91.log

SCF (B3PW91) = -3040.46500947
E(SCF)+ZPE(0 K) = -3039.100545
H(298 K) = -3039.017854
G(298 K) = -3039.221397
Lowest Frequency = -28.3296 cm^{-1}

Pd	-0.001074	-0.053421	-0.611521
Al	2.286991	0.061878	0.280572
N	3.738728	-1.352855	0.234067
N	3.589643	1.433771	0.971751
C	4.946446	-1.252811	0.793805
C	5.486262	-0.042886	1.251443
H	6.500460	-0.092184	1.630037
C	4.869269	1.207737	1.318277
C	5.801437	-2.475591	1.036809
H	5.632332	-2.815006	2.065936
H	6.862370	-2.231384	0.946182
H	5.562241	-3.305620	0.374070
C	5.718177	2.325908	1.881619
H	5.550112	3.270005	1.360841
H	6.776689	2.065885	1.820070
H	5.474079	2.498505	2.934767
C	3.363926	-2.615130	-0.351904
C	2.567320	-3.516045	0.386740
C	2.165524	-4.708215	-0.225159
H	1.542633	-5.403875	0.330686
C	2.558922	-5.023584	-1.518836
C	3.373657	-4.143075	-2.221675
H	3.691413	-4.400882	-3.228000
C	3.788832	-2.927006	-1.668427
C	3.079629	2.748524	1.272206
C	2.978401	3.716587	0.247567
C	2.565887	5.007262	0.597218
H	2.498680	5.770119	-0.171986
C	2.239602	5.334903	1.907618
C	2.302239	4.360463	2.895534
H	2.025311	4.615803	3.914397
C	2.721195	3.058986	2.605054
C	1.075017	-0.021615	-2.411374
C	0.711331	-1.339649	-2.814073
C	0.420401	1.086308	-3.022538
C	-0.247783	-1.488228	-3.807159
H	1.237466	-2.204078	-2.427783
C	-0.523841	0.869396	-4.021203
H	0.722051	2.091117	-2.754666
C	-0.859430	-0.417911	-4.448782
H	-0.996157	1.729290	-4.486307
H	-1.577829	-0.597266	-5.240276
F	2.484691	0.183850	-2.155727
F	-0.571773	-2.742212	-4.192721
H	2.240306	-5.956122	-1.976678
H	1.926324	6.345574	2.155865
C	3.333202	3.396893	-1.198683
C	4.835624	3.558711	-1.479810
C	2.529097	4.223245	-2.209336
H	3.083235	2.345512	-1.371529
H	5.440677	2.868023	-0.887569
H	5.046754	3.357364	-2.535818
H	5.166757	4.580432	-1.259562
H	1.458118	4.210464	-1.984590
H	2.855599	5.269357	-2.237774
H	2.673250	3.817297	-3.215829
C	2.754366	2.019313	3.718414
C	1.349434	1.451918	3.966015
C	3.355576	2.549262	5.027305
H	3.383033	1.187294	3.386005
H	0.925938	1.027601	3.050244
H	1.379641	0.666145	4.729376
H	0.671188	2.237853	4.316702
H	4.336578	3.011507	4.875173
H	2.709322	3.295641	5.501636
H	3.476221	1.728975	5.742828
C	4.709461	-2.025103	-2.485288
C	4.211265	-1.803805	-3.920520
C	6.142715	-2.582312	-2.533799
H	4.740867	-1.049140	-1.993537
H	3.181995	-1.443050	-3.941813
H	4.842080	-1.060137	-4.419524
H	4.265654	-2.722655	-4.514963
H	6.587568	-2.685598	-1.541435
H	6.160422	-3.569175	-3.010443
H	6.787231	-1.918324	-3.120646
C	2.177096	-3.264154	1.834516
C	2.774240	-4.332159	2.764864
C	0.659182	-3.175886	2.011167
H	2.600833	-2.298276	2.131541
H	3.860719	-4.409785	2.655623
H	2.553134	-4.092192	3.810779
H	2.351074	-5.321340	2.557902
H	0.239067	-2.371813	1.397338
H	0.166046	-4.112272	1.732498
H	0.408858	-2.973165	3.057690
Al	-2.386823	0.015194	0.126488
N	-3.760652	1.446344	-0.264350
N	-3.797462	-1.091380	1.045759
C	-5.098166	-0.789966	1.189358
C	-5.661130	0.429225	0.799714
C	-5.025979	1.500581	0.157881
H	-6.708831	0.568037	1.036960

C	-6.037408	-1.782763	1.832478
H	-5.936792	-2.770986	1.376615
H	-7.072739	-1.451129	1.738356
H	-5.809346	-1.907797	2.895346
H	-5.547225	3.483040	0.759280
H	-6.905925	2.555711	0.110691
C	-5.840665	2.760944	-0.011371
H	-5.674377	3.241794	-0.975809
C	-3.366279	-2.344189	1.615644
C	-3.082240	-2.408129	3.000262
C	-2.705617	-3.637988	3.545487
C	-2.611743	-4.779841	2.757312
C	-2.877618	-4.697617	1.397340
C	-3.253746	-3.489201	0.799369
H	-2.796570	-5.589081	0.780774
H	-2.326613	-5.729061	3.203167
H	-2.485005	-3.704179	4.607234
C	-3.151925	-1.179016	3.896604
H	-3.721275	-0.408811	3.366716
C	-1.745285	-0.615040	4.142706
H	-1.237933	-0.386858	3.200137
H	-1.794745	0.302688	4.739532
H	-1.127628	-1.338650	4.686754
H	-4.862116	-1.877668	5.081446
C	-3.869141	-1.439812	5.227833
H	-3.992815	-0.500642	5.777596
H	-3.300822	-2.117866	5.873513
H	-3.820001	-2.440235	-0.961610
C	-3.534505	-3.462996	-0.694904
C	-4.705172	-4.379697	-1.079516
H	-4.482765	-5.429100	-0.856577
H	-5.625064	-4.115650	-0.547541
C	-2.277209	-3.816320	-1.499396
H	-2.465701	-3.725654	-2.573011
H	-1.449307	-3.147976	-1.246503
H	-1.955293	-4.845425	-1.303076
H	-4.906251	-4.306840	-2.153765
C	-3.266477	2.546120	-1.049121
C	-2.630364	3.627550	-0.399752
C	-2.115320	4.668909	-1.176890
C	-2.240028	4.661703	-2.560058
C	-2.883351	3.598525	-3.180169
C	-3.403805	2.517704	-2.455734
C	-4.124991	1.453524	-3.289542
H	-2.989459	3.596241	-4.263275
H	-1.840496	5.480421	-3.152549
H	-1.615003	5.499753	-0.686872
C	-2.496651	3.696454	1.113669
H	-3.091077	2.880382	1.538334
C	-3.048657	5.010825	1.685760
H	-4.078629	5.199911	1.365440
H	-3.033324	4.981841	2.780714
H	-2.445013	5.870664	1.375789
H	-0.966926	3.497678	2.643725
C	-1.042675	3.485389	1.550947
H	-0.650651	2.529096	1.187899
H	-0.394187	4.277937	1.164804
H	-3.635185	1.508900	-4.270668
H	-5.707916	2.854823	-3.873072
C	-5.599920	1.821902	-3.526728
H	-6.035799	1.162465	-4.285744
H	-6.193170	1.703627	-2.614677
H	-4.337643	-0.653757	-3.679047
C	-4.010809	-0.010040	-2.854339
H	-4.647533	-0.236574	-1.994771
H	-2.974825	-0.279921	-2.620803

TS-1_AlPdAl _WB97X.log

SCF (wB97x) = -3040.80099918
E(SCF)+ZPE(0 K) = -3039.418657
H(298 K) = -3039.338446
G(298 K) = -3039.532883
Lowest Frequency = -57.9263 cm^{-1}

Pd	0.052220	-0.132647	-0.877551
Al	-2.230781	-0.185919	-0.056374
N	-3.573306	1.293886	-0.140115
N	-3.544966	-1.434859	0.710001
C	-4.843615	1.217067	0.241661
C	-5.472711	0.013272	0.603582
H	-6.531856	0.075523	0.825403
C	-4.853900	-1.205721	0.882445
C	-5.683015	2.464348	0.413959
H	-5.569860	2.811133	1.447852
H	-6.739572	2.237580	0.255059
H	-5.384179	3.279756	-0.243392
C	-5.734158	-2.263716	1.511866
H	-5.467266	-3.270811	1.187884
H	-6.782384	-2.072067	1.274845
H	-5.622537	-2.235717	2.601485
C	-3.029804	2.600816	-0.436262
C	-2.434156	3.340178	0.600792
C	-1.886391	4.590060	0.300973
H	-1.406082	5.163592	1.090969
C	-1.942247	5.104730	-0.983250
C	-2.537322	4.364068	-1.997565
H	-2.566343	4.774943	-3.001989
C	-3.084100	3.102094	-1.757734
C	-3.012384	-2.638164	1.304802
C	-2.746735	-3.764801	0.502874
C	-2.300015	-4.929499	1.131837
H	-2.104633	-5.816682	0.536708
C	-2.097070	-4.977967	2.504208
C	-2.312969	-3.842925	3.271869
H	-2.122203	-3.876844	4.341729
C	-2.769272	-2.658319	2.692944
C	-0.998114	-0.501750	-2.612775
C	-0.530849	0.748907	-3.115946
C	-0.410364	-1.693965	-3.137073
C	0.416375	0.750688	-4.135982
H	-0.983872	1.683433	-2.805356
C	0.539652	-1.619424	-4.138870
H	-0.747407	-2.654942	-2.765398
C	0.958230	-0.395626	-4.681796
H	0.965713	-2.543909	-4.522012
H	1.688280	-0.333075	-5.481057
F	-2.448879	-0.623320	-2.381539
F	0.804578	1.955580	-4.618600
H	-1.517472	6.081463	-1.201436
H	-1.753476	-5.896957	2.972450
C	-2.961899	-3.742180	-1.006054
C	-4.422045	-4.029452	-1.381573
C	-2.035772	-4.708023	-1.751948
H	-2.730843	-2.731844	-1.358632
H	-5.098599	-3.253683	-1.012407
H	-4.532057	-4.068839	-2.470578
H	-4.746676	-4.994322	-0.972991
H	-0.988873	-4.577668	-1.453410
H	-2.314038	-5.754673	-1.581379
H	-2.110982	-4.530981	-2.830315
C	-2.951799	-1.423510	3.569299
C	-1.593574	-0.766714	3.852990
C	-3.686963	-1.725185	4.880219
H	-3.557890	-0.695583	3.019229
H	-1.078723	-0.492486	2.924854
H	-1.722947	0.140218	4.456167
H	-0.940910	-1.452544	4.407127
H	-4.636908	-2.242386	4.706368
H	-3.085815	-2.351208	5.548521
H	-3.900218	-0.793403	5.414379
C	-3.719809	2.318049	-2.907215
C	-3.185933	2.725279	-4.287322
C	-5.252380	2.433579	-2.932387
H	-3.471048	1.264249	-2.754956
H	-2.093610	2.762871	-4.329647
H	-3.521258	1.997193	-5.032566
H	-3.572325	3.704264	-4.595598
H	-5.726642	1.921506	-2.092606
H	-5.566423	3.484500	-2.921019
H	-5.642428	1.978341	-3.849039

C	-2.367384	2.842566	2.039123
C	-3.086603	3.799890	2.998952
C	-0.916663	2.629399	2.481865
H	-2.881716	1.875891	2.096486
H	-4.121544	3.986495	2.692899
H	-3.099353	3.383814	4.012279
H	-2.577251	4.768866	3.046374
H	-0.404457	1.911267	1.831402
H	-0.354109	3.568087	2.453989
H	-0.884399	2.251488	3.510806
Al	2.299634	0.097955	0.092042
N	3.717624	-1.293184	-0.043221
N	3.576681	1.349718	0.908487
C	4.902548	1.192737	1.027527
C	5.566320	-0.008794	0.771059
C	4.981968	-1.214778	0.346679
H	6.630479	-0.029795	0.975288
C	5.750250	2.346553	1.512476
H	5.498866	3.264753	0.974243
H	6.810283	2.127941	1.375269
H	5.569826	2.543257	2.574383
H	5.682498	-2.945583	1.364189
H	6.909147	-2.182629	0.343348
C	5.851655	-2.448855	0.401035
H	5.613448	-3.167501	-0.383358
C	3.007331	2.569839	1.426307
C	2.828623	2.689534	2.822764
C	2.326832	3.887558	3.325348
C	1.984769	4.935606	2.476404
C	2.117369	4.781586	1.106158
C	2.625948	3.601232	0.553246
H	1.826800	5.593375	0.442738
H	1.601171	5.866045	2.887862
H	2.193924	4.006820	4.397191
C	3.132087	1.526339	3.762722
H	3.904355	0.903227	3.299492
C	1.886681	0.645277	3.940857
H	1.492516	0.294814	2.979311
H	2.116663	-0.232215	4.556773
H	1.085595	1.208383	4.434344
H	4.527121	2.646568	5.023127
C	3.672580	1.968765	5.125564
H	3.998133	1.095118	5.699511
H	2.908549	2.478941	5.722288
H	3.110329	2.485683	-1.201246
C	2.744743	3.489735	-0.960080
C	3.763304	4.489200	-1.520926
H	3.459563	5.522109	-1.315241
H	4.758138	4.340765	-1.085859
C	1.376439	3.655688	-1.632693
H	1.441609	3.425861	-2.701449
H	0.638456	2.978881	-1.187678
H	1.001787	4.680795	-1.526383
H	3.848254	4.376310	-2.606857
C	3.212730	-2.524522	-0.592109
C	2.551059	-3.430452	0.256925
C	1.976429	-4.573199	-0.301704
C	2.069061	-4.825634	-1.662448
C	2.736184	-3.924699	-2.481225
C	3.309435	-2.753298	-1.979298
C	4.030595	-1.863812	-2.998695
H	2.809534	-4.124524	-3.548958
H	1.621067	-5.720989	-2.086245
H	1.447270	-5.272831	0.342200
C	2.448210	-3.209374	1.760076
H	2.993388	-2.291144	2.007684
C	3.107944	-4.355413	2.538082
H	4.149080	-4.507814	2.232809
H	3.093275	-4.145351	3.613040
H	2.575588	-5.299822	2.378696
H	0.942786	-2.804730	3.274493
C	0.992497	-3.021099	2.199985
H	0.514233	-2.198740	1.655651
H	0.402923	-3.925822	2.015900
H	3.570715	-2.132869	-3.958707
H	5.669207	-3.301896	-3.211664
C	5.520078	-2.221474	-3.111739
H	5.958896	-1.733731	-3.989120
H	6.080052	-1.876916	-2.235647
H	4.180263	0.122768	-3.815081
C	3.862464	-0.344976	-2.876249
H	4.482974	0.077643	-2.078592
H	2.815458	-0.068926	-2.709715

TS-3_AlPdAl_Dipp_B3PW91.log

SCF (B3PW91) = -3040.46573231
E(SCF)+ZPE(0 K) = -3039.106375
H(298 K) = -3039.022644
G(298 K) = -3039.231255
Lowest Frequency = -800.9277 cm^{-1}

Pd	-0.054920	-0.064620	-0.723143
Al	2.050286	0.202865	0.475219
N	3.328819	1.730232	0.353523
N	3.221679	-0.729038	1.813630
C	4.284685	2.023322	1.242297
C	4.648919	1.164876	2.287738
H	5.431635	1.522163	2.945207
C	4.223362	-0.151631	2.497200
C	5.061732	3.310434	1.119512
H	5.664837	3.313229	0.206679
H	5.724912	3.447281	1.975213
H	4.388704	4.168374	1.046695
C	4.972861	-0.933452	3.549593
H	4.315182	-1.201025	4.380693
H	5.807928	-0.351126	3.941641
H	5.356356	-1.873536	3.144321
C	3.207455	2.600085	-0.793759
C	4.000278	2.340819	-1.934196
C	3.890056	3.200322	-3.030892
H	4.490549	3.008795	-3.916246
C	3.025804	4.287461	-3.012980
H	2.955255	4.945044	-3.875259
C	2.244052	4.520548	-1.890003
H	1.554841	5.360612	-1.883527
C	2.313734	3.690174	-0.767203
C	4.947682	1.153649	-2.026037
H	4.955518	0.652561	-1.052595
C	4.444945	0.143615	-3.066983
H	3.427437	-0.188429	-2.842830
H	5.100006	-0.734190	-3.100016
H	4.428739	0.584855	-4.069123
C	6.391629	1.576452	-2.332705
H	6.473023	2.047980	-3.317977
H	7.051740	0.702090	-2.332965
H	6.777059	2.286701	-1.593922
C	1.404716	3.976009	0.415510
H	1.664840	3.276006	1.217287
C	-0.057997	3.721061	0.032838
H	-0.716330	3.903776	0.886540
H	-0.199317	2.685310	-0.298002
H	-0.369922	4.382156	-0.783162
C	1.583611	5.397285	0.968750
H	2.619331	5.601775	1.260702
H	0.950682	5.539774	1.851183
H	1.292793	6.155345	0.233410
C	3.005718	-2.136139	2.055915
C	2.232529	-2.563782	3.159170
C	2.137580	-3.935484	3.415209
H	1.553972	-4.275441	4.266665
C	2.768341	-4.870686	2.604612
H	2.689405	-5.931338	2.827258
C	3.483991	-4.438954	1.496166
H	3.961044	-5.171081	0.850504
C	3.612809	-3.078988	1.194388
C	1.467252	-1.602647	4.057194
H	1.766339	-0.582696	3.793600
C	-0.041104	-1.728292	3.792711
H	-0.385396	-2.751511	3.980496

H	-0.276938	-1.469911	2.755611
H	-0.605996	-1.060860	4.452435
C	1.758604	-1.809709	5.551129
H	1.384969	-2.776364	5.905656
H	1.261234	-1.033806	6.143098
H	2.828385	-1.769064	5.779142
C	4.429921	-2.676779	-0.025477
H	4.294318	-1.600842	-0.179303
C	3.949355	-3.386686	-1.298132
H	4.094250	-4.470884	-1.235784
H	4.520563	-3.033149	-2.162689
H	2.891508	-3.194697	-1.492386
C	5.930555	-2.920358	0.197824
H	6.311960	-2.360605	1.057435
H	6.504040	-2.611575	-0.682984
H	6.135396	-3.982525	0.373307
Al	-2.194372	-0.172971	0.361338
N	-3.493068	-1.653936	0.058373
N	-3.532666	0.854507	1.431477
C	-4.590975	-1.883153	0.786846
C	-5.084226	-0.975482	1.734981
H	-5.972421	-1.284370	2.271740
C	-4.647942	0.332283	1.970218
C	-5.374285	-3.158134	0.595686
H	-4.755762	-4.025892	0.842694
H	-5.678114	-3.283801	-0.446647
H	-6.262999	-3.169233	1.228535
C	-5.525549	1.181673	2.857055
H	-4.960398	1.601479	3.692464
H	-6.358577	0.597632	3.250860
H	-5.928409	2.033001	2.300164
C	-3.206299	-2.577063	-1.015742
C	-3.719388	-2.307361	-2.304879
C	-3.477849	-3.234041	-3.323588
H	-3.874340	-3.044242	-4.317400
C	-2.736878	-4.385821	-3.093123
H	-2.559186	-5.092634	-3.899071
C	-2.209399	-4.617342	-1.830174
H	-1.605928	-5.504252	-1.657919
C	-2.428060	-3.727960	-0.773821
C	-4.522706	-1.056087	-2.630403
H	-4.498305	-0.406455	-1.749225
C	-3.901543	-0.275908	-3.797449
H	-3.941325	-0.847431	-4.730893
H	-2.854284	-0.031593	-3.600398
H	-4.448083	0.658519	-3.964229
C	-5.995501	-1.379896	-2.924126
H	-6.554079	-0.463447	-3.144880
H	-6.483755	-1.871521	-2.077070
H	-6.087874	-2.042538	-3.791841
C	-1.776877	-4.009364	0.569517
H	-2.157652	-3.276774	1.290214
C	-2.106277	-5.407037	1.113371
H	-3.184976	-5.582902	1.186175
H	-1.676702	-5.532604	2.113041
H	-1.687048	-6.195507	0.479138
C	-0.259245	-3.813193	0.462679
H	0.223310	-3.985460	1.428775
H	-0.016641	-2.797714	0.129459
H	0.173424	-4.512766	-0.260407
C	-3.313966	2.265887	1.645892
C	-2.628569	2.718526	2.795376
C	-2.520515	4.097271	3.007271
H	-2.004973	4.456840	3.893949
C	-3.053482	5.014079	2.109986
H	-2.964186	6.080561	2.297897
C	-3.683040	4.555149	0.960559
H	-4.077803	5.271435	0.244885
C	-3.823096	3.187739	0.702614
C	-1.976340	1.776095	3.795805
H	-2.232073	0.750727	3.508766
C	-0.447705	1.911144	3.725728
H	-0.132859	2.937305	3.945476
H	-0.082320	1.643649	2.729354
H	0.032196	1.252658	4.457425
C	-2.466826	1.998151	5.234244
H	-2.021224	1.254980	5.904354
H	-3.554675	1.913406	5.320237
H	-2.182751	2.987142	5.609720
C	-4.511733	2.755413	-0.583750
H	-4.549676	1.661488	-0.591702
C	-3.707008	3.202242	-1.813303
H	-3.630940	4.294180	-1.861846
H	-4.195278	2.864131	-2.733279
H	-2.691745	2.795990	-1.799056
C	-5.958903	3.262703	-0.666615
H	-6.000709	4.357096	-0.691552
H	-6.558844	2.927190	0.185354
H	-6.439057	2.894600	-1.579658
H	-0.998406	-0.241692	-2.000321
C	0.394134	-0.212313	-2.793155
C	0.447250	0.889369	-3.648313
C	0.784941	-1.406795	-3.398433
C	0.875274	0.848114	-4.969975
C	1.225006	-1.524531	-4.711418
C	1.268930	-0.377958	-5.503984
H	0.891941	1.761068	-5.556467
H	1.522024	-2.496317	-5.092930
H	1.605152	-0.440992	-6.534743
F	0.744141	-2.542894	-2.655205
F	0.042637	2.088882	-3.163089

TS-3_AlPdAl _WB97X.log

SCF (wB97x) = -3040.80071758
E(SCF)+ZPE(0 K) = -3039.423187
H(298 K) = -3039.342287
G(298 K) = -3039.538868
Lowest Frequency = -906.4199 cm^{-1}

Pd	-0.008152	0.081862	1.110840
Al	-1.842983	-0.089507	-0.457753
N	-3.269015	1.248364	-0.678378
N	-2.538392	-1.147473	-1.984573
C	-4.015132	1.437286	-1.764541
C	-4.048121	0.530478	-2.835015
H	-4.718956	0.770729	-3.651166
C	-3.456709	-0.738480	-2.866019
C	-4.913970	2.646744	-1.861138
H	-5.587816	2.699331	-1.001345
H	-5.504595	2.620991	-2.778066
H	-4.315790	3.562782	-1.851295
C	-3.982338	-1.688535	-3.919380
H	-3.212510	-2.349307	-4.319412
H	-4.446324	-1.132421	-4.736232
H	-4.750757	-2.326554	-3.467465
C	-3.490283	2.106985	0.463951
C	-4.331208	1.635254	1.495114
C	-4.575058	2.476424	2.579579
H	-5.215667	2.138214	3.387903
C	-3.985240	3.731482	2.664933
H	-4.183868	4.367907	3.523511
C	-3.117943	4.152601	1.671143
H	-2.623451	5.116551	1.763625
C	-2.850613	3.353258	0.557894
C	-4.941059	0.236260	1.453116
H	-4.184363	-0.430501	1.017343
C	-5.260338	-0.319948	2.842894
H	-4.401124	-0.240462	3.516125
H	-5.535615	-1.377071	2.762354
H	-6.111632	0.197606	3.300589
C	-6.190607	0.161172	0.565059
H	-6.957116	0.859475	0.921974
H	-6.617126	-0.848860	0.596724
H	-5.975156	0.393344	-0.481871
C	-1.831315	3.827514	-0.466173
H	-1.882413	3.157272	-1.330768
C	-0.414526	3.732798	0.115224
H	0.325062	4.041127	-0.630974
H	-0.182224	2.711586	0.440556
H	-0.313886	4.385683	0.990071

C	-2.104146	5.248599	-0.973058
H	-3.117462	5.361010	-1.375275
H	-1.391328	5.502745	-1.765854
H	-1.981083	5.990196	-0.176148
C	-2.113214	-2.524657	-2.064789
C	-0.944862	-2.857814	-2.780031
C	-0.611047	-4.207763	-2.903379
H	0.275553	-4.490522	-3.464351
C	-1.387431	-5.200770	-2.319068
H	-1.110233	-6.245813	-2.431524
C	-2.501302	-4.850629	-1.570861
H	-3.086146	-5.627870	-1.084799
C	-2.881095	-3.514988	-1.424167
C	-0.073156	-1.792797	-3.437080
H	-0.185070	-0.876326	-2.846511
C	1.415661	-2.155499	-3.439796
H	1.635441	-2.983941	-4.122640
H	1.768985	-2.437847	-2.443043
H	2.012612	-1.300734	-3.777024
C	-0.527334	-1.490669	-4.871833
H	-0.540355	-2.407019	-5.474074
H	0.163495	-0.786551	-5.350061
H	-1.526825	-1.047358	-4.905088
C	-4.076390	-3.175716	-0.542654
H	-4.327259	-2.119686	-0.694220
C	-3.713300	-3.365016	0.937712
H	-3.448333	-4.409789	1.138598
H	-4.565767	-3.106618	1.576707
H	-2.860832	-2.746441	1.241568
C	-5.324679	-3.991220	-0.898920
H	-5.581335	-3.901818	-1.960122
H	-6.181412	-3.642535	-0.312220
H	-5.189963	-5.055745	-0.677981
Al	2.062583	0.001586	-0.077811
N	3.459075	-1.386579	0.043581
N	3.094654	0.996716	-1.450003
C	4.398539	-1.630304	-0.867064
C	4.644129	-0.784959	-1.959618
H	5.427409	-1.094503	-2.640807
C	4.108069	0.492032	-2.164378
C	5.299336	-2.832216	-0.716738
H	4.716258	-3.733000	-0.509152
H	5.976918	-2.694309	0.131758
H	5.895419	-2.984929	-1.617691
C	4.754349	1.322007	-3.250217
H	4.045335	1.521211	-4.058000
H	5.620664	0.805447	-3.665161
H	5.071496	2.293783	-2.861305
C	3.478468	-2.166489	1.261796
C	4.319455	-1.747984	2.311919
C	4.342577	-2.498572	3.487186
H	4.986209	-2.185533	4.306410
C	3.552324	-3.629636	3.633542
H	3.582542	-4.202854	4.556324
C	2.712959	-4.014242	2.599543
H	2.079100	-4.889468	2.721327
C	2.652786	-3.294990	1.404234
C	5.175649	-0.489997	2.232684
H	5.076273	-0.064045	1.227970
C	4.675816	0.557733	3.235594
H	4.751900	0.183769	4.262657
H	3.626349	0.815456	3.053377
H	5.278328	1.471408	3.169421
C	6.664106	-0.782546	2.457720
H	7.253992	0.132760	2.335464
H	7.045529	-1.525242	1.748817
H	6.849531	-1.162759	3.468313
C	1.687783	-3.755659	0.323815
H	1.761255	-3.055141	-0.513549
C	2.040737	-5.151457	-0.204635
H	3.059177	-5.196549	-0.607069
H	1.346496	-5.434978	-1.003439
H	1.963400	-5.905297	0.587221
C	0.241747	-3.712860	0.827466
H	-0.444779	-4.004194	0.026281
H	-0.026045	-2.707128	1.167746
H	0.093037	-4.403058	1.666527
C	2.774491	2.391726	-1.658922
C	1.970554	2.801198	-2.739546
C	1.792316	4.169545	-2.958064
H	1.181575	4.498291	-3.795923
C	2.371701	5.114654	-2.126374
H	2.231407	6.174916	-2.319206
C	3.103289	4.695890	-1.023644
H	3.520057	5.439172	-0.350603
C	3.304412	3.340762	-0.758524
C	1.235130	1.825049	-3.648006
H	1.578644	0.812515	-3.409168
C	-0.270787	1.892165	-3.355649
H	-0.661653	2.896188	-3.560539
H	-0.463287	1.668963	-2.300701
H	-0.830206	1.181441	-3.974672
C	1.491492	2.080827	-5.138809
H	1.002467	1.311053	-5.745668
H	2.557681	2.077434	-5.386921
H	1.084261	3.047645	-5.453720
C	4.096401	2.925829	0.476312
H	3.736508	1.937270	0.785305
C	3.861030	3.860776	1.666055
H	4.321048	4.843681	1.514617
H	4.305303	3.432672	2.569524
H	2.791234	4.002646	1.849866
C	5.593659	2.792160	0.171447
H	6.000639	3.743595	-0.191104
H	5.788206	2.026282	-0.587063
H	6.146461	2.511346	1.075781
H	0.641832	0.238196	2.568068
C	-0.837229	0.266579	3.047579
C	-1.110882	1.487509	3.660098
C	-1.363855	-0.824084	3.734907
C	-1.848188	1.646323	4.824747
C	-2.103129	-0.745376	4.906098
C	-2.347829	0.511286	5.455027
H	-2.016236	2.644366	5.216912
H	-2.477122	-1.655613	5.364623
H	-2.922636	0.603360	6.372203
F	-1.158124	-2.065042	3.218871
F	-0.609802	2.610474	3.086499

TS-4_AlPdAl_Dipp_B3PW91.log

SCF (B3PW91) = -3040.47958975
E(SCF)+ZPE(0 K) = -3039.119541
H(298 K) = -3039.036754
G(298 K) = -3039.241161
Lowest Frequency = -122.2360 cm^{-1}

Pd	-0.106559	0.084556	0.210678
Al	2.182757	0.355342	0.598949
N	3.415779	1.832076	0.330936
N	3.490053	-0.807961	1.486076
C	4.483083	2.011785	1.125456
C	4.968149	1.037308	2.001146
H	5.825675	1.319501	2.599325
C	4.562497	-0.301370	2.108607
C	5.251046	3.307003	1.062825
H	5.828023	3.369583	0.135295
H	5.944075	3.381340	1.902348
H	4.578268	4.167125	1.071559
C	5.408981	-1.164873	3.012672
H	5.432497	-2.206776	2.693879
H	4.996551	-1.141526	4.026574
H	6.428652	-0.778033	3.060597
C	3.172603	2.831762	-0.682674
C	3.886171	2.756036	-1.900388
C	3.613197	3.712432	-2.882172
H	4.140484	3.663835	-3.831235
C	2.687900	4.726791	-2.665803
H	2.492177	5.461096	-3.442731
C	2.023057	4.803661	-1.449525

H	1.307938	5.604052	-1.279420
C	2.246839	3.865382	-0.437173
C	4.947294	1.695510	-2.171294
H	5.134996	1.158891	-1.235787
C	4.472723	0.661294	-3.200393
H	3.565735	0.149896	-2.870120
H	5.251993	-0.090169	-3.372028
H	4.249086	1.136426	-4.161462
C	6.280288	2.314001	-2.620585
H	6.195418	2.782516	-3.606901
H	7.050664	1.538755	-2.694192
H	6.638453	3.079764	-1.924778
C	1.500820	4.002871	0.881090
H	1.850731	3.211381	1.552937
C	-0.007035	3.806249	0.692936
H	-0.528304	3.898385	1.650294
H	-0.229608	2.813216	0.286716
H	-0.422978	4.556448	0.011806
C	1.790089	5.348833	1.563721
H	2.861009	5.510553	1.726757
H	1.292372	5.390912	2.538510
H	1.417021	6.189256	0.968080
C	3.220288	-2.217218	1.658376
C	2.542537	-2.669563	2.811417
C	2.327378	-4.045625	2.949480
H	1.803739	-4.412443	3.828097
C	2.774220	-4.947515	1.994673
H	2.603013	-6.012599	2.126441
C	3.441231	-4.483277	0.866446
H	3.781594	-5.195165	0.121716
C	3.673038	-3.119945	0.667830
C	2.059308	-1.742383	3.919548
H	2.396261	-0.726872	3.686222
C	0.528095	-1.701441	4.001043
H	0.121332	-2.691693	4.234450
H	0.088188	-1.353676	3.062741
H	0.215042	-1.015889	4.795856
C	2.634995	-2.134815	5.290822
H	2.221858	-3.088714	5.636466
H	2.375400	-1.378504	6.039373
H	3.723981	-2.240380	5.277787
C	4.424383	-2.645615	-0.570935
H	4.008682	-1.669460	-0.849261
C	4.250616	-3.573930	-1.777848
H	4.807321	-4.510147	-1.654557
H	4.639818	-3.085333	-2.676596
H	3.198902	-3.811619	-1.945545
C	5.924416	-2.446840	-0.296511
H	6.114927	-1.668294	0.445542
H	6.441489	-2.156351	-1.217764
H	6.378571	-3.377803	0.061991
Al	-2.515470	-0.226951	0.226947
N	-3.721505	-1.700659	-0.402191
N	-4.102592	0.833832	0.843406
C	-5.033315	-1.825339	-0.166508
C	-5.790789	-0.856069	0.501665
H	-6.839904	-1.083946	0.643256
C	-5.371457	0.409735	0.925935
C	-5.775147	-3.042017	-0.662078
H	-5.777299	-3.071653	-1.756047
H	-6.808811	-3.037281	-0.312468
H	-5.289801	-3.962848	-0.329444
C	-6.439364	1.323448	1.476034
H	-6.180274	1.676777	2.476992
H	-7.403001	0.813546	1.519003
H	-6.543239	2.216493	0.852898
C	-3.095737	-2.713871	-1.217156
C	-3.078544	-2.551169	-2.620186
C	-2.441112	-3.525008	-3.393968
H	-2.410110	-3.407651	-4.473797
C	-1.844270	-4.635408	-2.809730
H	-1.353651	-5.381663	-3.428973
C	-1.878645	-4.785706	-1.429713
H	-1.409007	-5.653531	-0.974304
C	-2.500093	-3.838814	-0.609626
C	-3.711162	-1.351155	-3.310020
H	-4.283851	-0.796309	-2.559531
C	-2.629878	-0.409533	-3.856145
H	-2.023492	-0.907850	-4.619666
H	-1.948845	-0.086033	-3.063831
H	-3.083171	0.478663	-4.310938
C	-4.690655	-1.758383	-4.419891
H	-5.185354	-0.871775	-4.831370
H	-5.467618	-2.437527	-4.053328
H	-4.178962	-2.258976	-5.248881
C	-2.504017	-4.052903	0.895512
H	-3.102759	-3.253787	1.345876
C	-3.145390	-5.390514	1.293938
H	-4.165132	-5.492070	0.908122
H	-3.189622	-5.479072	2.385016
H	-2.565632	-6.241311	0.919766
C	-1.084183	-3.945529	1.463519
H	-1.099530	-4.058768	2.552774
H	-0.633067	-2.978591	1.221670
H	-0.433070	-4.725250	1.054938
C	-3.841852	2.196679	1.236702
C	-3.527617	2.495573	2.581196
C	-3.322720	3.833582	2.933345
H	-3.086563	4.078491	3.965695
C	-3.415493	4.852895	1.993827
H	-3.258775	5.886528	2.290550
C	-3.697846	4.540148	0.670217
H	-3.754814	5.336821	-0.067025
C	-3.911624	3.219221	0.263812
C	-3.395488	1.424219	3.654118
H	-3.648257	0.461137	3.198140
C	-1.947439	1.331093	4.155571
H	-1.634563	2.268124	4.630915
H	-1.258809	1.116441	3.333005
H	-1.855670	0.533264	4.901095
C	-4.355526	1.648744	4.832138
H	-4.269587	0.826933	5.551322
H	-5.401149	1.704084	4.512794
H	-4.124916	2.576795	5.366626
C	-4.208017	2.939451	-1.203420
H	-4.388170	1.864725	-1.309583
C	-3.006296	3.296910	-2.089998
H	-2.777694	4.367139	-2.032649
H	-3.222066	3.060692	-3.137838
H	-2.108674	2.746866	-1.795767
C	-5.470389	3.666881	-1.689844
H	-5.348574	4.754768	-1.647331
H	-6.350043	3.409547	-1.091224
H	-5.684532	3.400983	-2.730731
H	0.778331	0.590410	1.709710
C	0.913724	-0.212097	-1.649102
C	0.813710	0.760431	-2.648241
C	1.178823	-1.480779	-2.164740
C	0.959274	0.534981	-4.012480
C	1.340450	-1.791332	-3.511407
C	1.231955	-0.761871	-4.444269
H	0.852109	1.360877	-4.708177
H	1.529313	-2.816530	-3.811621
H	1.353857	-0.970490	-5.503287
F	1.268260	-2.517212	-1.287448
F	0.509045	2.029839	-2.277829

TS-4_AlPdAl_WB97X.log

SCF (wB97x) = -3040.82165888
E(SCF)+ZPE(0 K) = -3039.444500
H(298 K) = -3039.363956
G(298 K) = -3039.559232
Lowest Frequency = -112.8864 cm^{-1}

Pd	-0.131540	0.102176	0.355471
Al	2.113611	0.500539	0.710979
N	3.270319	1.985218	0.347859
N	3.469708	-0.562352	1.582053
C	4.330575	2.256972	1.113897
C	4.862257	1.354991	2.041626

H	5.709479	1.700321	2.620963
C	4.516452	0.002190	2.186784
C	5.041063	3.577150	0.953821
H	5.564533	3.612465	-0.006603
H	5.769610	3.720927	1.752736
H	4.328326	4.405802	0.957188
C	5.403900	-0.823309	3.086752
H	6.332058	-0.291983	3.301077
H	5.636849	-1.792791	2.641702
H	4.889729	-1.017180	4.032712
C	2.972251	2.879911	-0.746398
C	3.660822	2.704093	-1.962538
C	3.324313	3.533535	-3.032021
H	3.832832	3.406549	-3.985286
C	2.356691	4.520606	-2.898790
H	2.109605	5.158230	-3.743683
C	1.711360	4.694581	-1.683869
H	0.955351	5.470161	-1.583791
C	1.997974	3.878871	-0.588481
C	4.761142	1.661903	-2.144471
H	4.971016	1.207254	-1.169646
C	4.326815	0.534090	-3.087378
H	3.437922	0.016674	-2.716722
H	5.134893	-0.199910	-3.197910
H	4.087345	0.923221	-4.083530
C	6.066273	2.295815	-2.644068
H	5.954671	2.692664	-3.659068
H	6.864810	1.546453	-2.669768
H	6.396330	3.120375	-2.002964
C	1.250043	4.098751	0.719717
H	1.635507	3.388366	1.459393
C	-0.249008	3.822399	0.559958
H	-0.768026	3.944941	1.517190
H	-0.430386	2.799111	0.211641
H	-0.704082	4.514319	-0.159831
C	1.483986	5.511670	1.270108
H	2.548892	5.727312	1.413179
H	0.983022	5.628066	2.236933
H	1.080227	6.273784	0.593977
C	3.278514	-1.986252	1.739443
C	2.627573	-2.489766	2.879445
C	2.494513	-3.875591	2.998397
H	1.991436	-4.287453	3.870427
C	2.990295	-4.731594	2.028216
H	2.883016	-5.807147	2.143563
C	3.612589	-4.212642	0.899120
H	3.977042	-4.892593	0.135208
C	3.758542	-2.837640	0.723168
C	2.065209	-1.599985	3.982819
H	2.353385	-0.564377	3.770577
C	0.532481	-1.646864	4.013334
H	0.178961	-2.659683	4.242360
H	0.101550	-1.333898	3.057492
H	0.152263	-0.973456	4.790265
C	2.618004	-1.977295	5.364973
H	2.253671	-2.961213	5.680659
H	2.287001	-1.250751	6.114358
H	3.712105	-2.014312	5.387027
C	4.413245	-2.280459	-0.538605
H	3.861249	-1.373010	-0.814825
C	4.325241	-3.234681	-1.732518
H	5.011824	-4.082739	-1.623364
H	4.604649	-2.702870	-2.648284
H	3.309536	-3.616924	-1.854998
C	5.878764	-1.878006	-0.318224
H	5.988961	-1.058652	0.397439
H	6.321740	-1.545842	-1.264582
H	6.463795	-2.732311	0.043200
Al	-2.512045	-0.353398	0.307144
N	-3.559098	-1.844950	-0.455142
N	-4.173952	0.564539	0.844254
C	-4.858446	-2.089736	-0.290819
C	-5.725334	-1.214268	0.376902
H	-6.759975	-1.524256	0.460720
C	-5.411040	0.064180	0.853075
C	-5.463742	-3.339699	-0.882615
H	-5.462373	-3.278130	-1.976175
H	-6.491703	-3.473195	-0.542287
H	-4.875872	-4.222187	-0.616056
C	-6.550914	0.899160	1.385275
H	-6.373243	1.170539	2.430030
H	-7.496067	0.359229	1.314959
H	-6.633644	1.837271	0.828483
C	-2.797479	-2.719911	-1.314977
C	-2.801618	-2.473969	-2.702429
C	-2.014036	-3.281013	-3.521385
H	-1.992384	-3.095387	-4.592523
C	-1.244890	-4.308718	-2.991240
H	-0.635236	-4.927015	-3.645476
C	-1.252993	-4.537376	-1.624468
H	-0.642303	-5.336274	-1.210125
C	-2.022043	-3.752380	-0.762626
C	-3.602696	-1.334733	-3.320441
H	-4.323821	-0.977809	-2.576670
C	-2.679054	-0.161218	-3.668585
H	-1.921474	-0.465631	-4.399948
H	-2.140112	0.202554	-2.786186
H	-3.253475	0.671135	-4.093069
C	-4.405268	-1.778238	-4.548739
H	-5.044944	-0.960286	-4.896838
H	-5.045131	-2.639452	-4.326274
H	-3.750996	-2.056573	-5.381937
C	-1.989013	-4.046482	0.729478
H	-2.647031	-3.327816	1.230917
C	-2.523704	-5.451320	1.036565
H	-3.543071	-5.592200	0.660228
H	-2.535726	-5.627317	2.117679
H	-1.892618	-6.222980	0.581312
C	-0.578806	-3.864651	1.300470
H	-0.582301	-4.039495	2.382604
H	-0.201727	-2.853221	1.112664
H	0.131007	-4.569469	0.852327
C	-3.983439	1.930893	1.266596
C	-3.739572	2.219987	2.622488
C	-3.549597	3.552119	2.992985
H	-3.360620	3.791165	4.037263
C	-3.594617	4.574429	2.055339
H	-3.449938	5.606494	2.364200
C	-3.810064	4.269726	0.719082
H	-3.826780	5.070853	-0.016820
C	-3.996567	2.952200	0.297310
C	-3.650769	1.135837	3.688156
H	-3.912530	0.180009	3.221307
C	-2.215994	1.015720	4.219138
H	-1.911647	1.939518	4.726039
H	-1.506735	0.824252	3.406908
H	-2.145739	0.196216	4.944449
C	-4.632893	1.373735	4.842142
H	-4.590211	0.541865	5.553189
H	-5.666889	1.465873	4.492275
H	-4.388021	2.289061	5.392350
C	-4.205975	2.677401	-1.187462
H	-4.311561	1.595065	-1.324580
C	-2.998131	3.133070	-2.016163
H	-2.835211	4.212800	-1.916828
H	-3.165247	2.920561	-3.078135
H	-2.077372	2.625104	-1.711075
C	-5.493896	3.331812	-1.703597
H	-5.445736	4.423312	-1.617750
H	-6.373987	2.993051	-1.146245
H	-5.649466	3.087689	-2.760022
H	0.730890	0.756006	1.842636
C	0.888590	-0.246215	-1.513946
C	0.726941	0.676968	-2.545498
C	1.204634	-1.515108	-1.988076
C	0.879419	0.416657	-3.900483
C	1.371201	-1.866652	-3.322834
C	1.209993	-0.878504	-4.288632
H	0.729475	1.214764	-4.621087
H	1.603444	-2.893974	-3.585783
H	1.336458	-1.118260	-5.340938
F	1.356788	-2.517908	-1.076320

F	0.347676	1.941867	-2.217214

TS-5_AlPdAl_Dipp_B3PW91.log

SCF (B3PW91) = -3040.47449158
E(SCF)+ZPE(0 K) = -3039.116178
H(298 K) = -3039.032497
G(298 K) = -3039.240918
Lowest Frequency = -227.7864 cm^{-1}

Pd	-0.033365	-0.045453	0.530329
Al	2.330095	-0.381094	0.055223
N	3.965524	0.780770	0.157949
N	3.440945	-1.974265	-0.403850
C	5.236800	0.371785	0.073810
C	5.606785	-0.975345	-0.048456
H	6.670566	-1.179554	-0.032101
C	4.775270	-2.067838	-0.315381
C	6.366738	1.373801	0.032266
H	7.309340	0.902488	0.318286
H	6.186463	2.240526	0.668484
H	6.479060	1.747762	-0.991826
C	5.455996	-3.390571	-0.569153
H	6.503644	-3.351114	-0.265606
H	5.415502	-3.647966	-1.632360
H	4.956990	-4.201433	-0.033778
C	3.727257	2.200618	0.250120
C	3.598821	2.965154	-0.929489
C	3.409726	4.345706	-0.811002
H	3.306531	4.945894	-1.710998
C	3.351972	4.961576	0.431623
C	3.450056	4.191322	1.584236
H	3.383859	4.675922	2.553638
C	3.622045	2.804789	1.524422
C	2.725484	-3.117806	-0.913549
C	2.116938	-4.032695	-0.028277
C	1.432849	-5.126441	-0.570439
H	0.964039	-5.840625	0.101104
C	1.337574	-5.315265	-1.942283
C	1.928191	-4.397273	-2.802544
H	1.843423	-4.540450	-3.876475
C	2.629336	-3.291565	-2.314258
C	-0.150800	-0.056689	2.621102
C	-1.259952	0.407319	3.148463
C	0.727787	-0.436313	3.641648
C	-1.757770	0.627362	4.402449
C	0.411499	-0.309582	4.994568
C	-0.822982	0.229168	5.371968
H	-2.729874	1.039691	4.636396
H	1.132350	-0.624109	5.742452
H	-1.066516	0.339968	6.425251
H	-1.021675	0.164547	-0.827458
F	1.948692	-0.948757	3.344217
F	-2.807876	0.912094	2.001373
C	2.177720	-3.882376	1.483425
H	2.717751	-2.958066	1.709334
C	3.232725	-2.303260	-3.303439
H	3.886213	-1.624032	-2.746971
C	3.624156	2.343447	-2.317826
H	3.902469	1.289492	-2.210149
C	3.700243	2.000177	2.815251
H	3.441130	0.964996	2.572665
Al	-2.437918	0.489646	0.298889
N	-3.293887	2.012760	-0.568032
N	-3.885836	-0.784551	-0.048122
C	-5.135996	-0.350639	-0.235886
C	-5.479909	1.007524	-0.364737
C	-4.631086	2.096201	-0.603389
C	-5.287424	3.404548	-0.966476
C	-2.778647	2.109027	-3.457335
H	-3.372955	1.387024	-2.889206
H	-5.070428	3.677722	-2.003073
H	-6.369759	3.344331	-0.843116
H	-4.901149	4.216296	-0.344468
H	-6.106739	-2.272670	0.102780
C	-6.275543	-1.326385	-0.409561
H	-7.209740	-0.882702	-0.057903
H	-6.400779	-1.550550	-1.474917
H	-6.541294	1.215915	-0.425370
C	-2.528606	3.113762	-1.096320
C	-2.285746	3.174209	-2.488214
C	-1.549516	4.250389	-2.991857
C	-1.057121	5.244698	-2.155681
C	-1.293796	5.166728	-0.790048
C	-2.027344	4.113248	-0.233746
H	-1.358224	4.307120	-4.060429
C	-2.246634	4.088753	1.270963
H	-0.902502	5.940175	-0.134585
H	-2.871267	3.224069	1.509111
C	-3.647605	-2.208057	-0.023555
C	-3.329214	-2.878370	-1.224310
C	-3.138999	-2.153991	-2.547599
C	-3.749161	-2.916434	1.197127
C	-3.591040	-4.306042	1.169440
C	-3.314174	-4.983674	-0.011014
C	-3.170184	-4.267371	-1.191231
H	-2.930822	-4.793902	-2.111252
H	-3.274866	-1.083107	-2.365503
C	-4.034898	-2.238188	2.531166
H	-3.928320	-1.159165	2.390599
H	-3.686707	-4.866273	2.095412
H	3.218611	6.037750	0.503692
H	0.802396	-6.172972	-2.341003
H	-0.489988	6.075507	-2.567164
H	-3.200644	-6.064546	-0.008113
C	2.692201	2.482283	3.865265
H	2.952994	3.469386	4.263390
H	1.680729	2.533478	3.454921
H	2.677980	1.788413	4.711123
C	5.114445	1.998216	3.417702
H	5.451502	3.020328	3.626680
H	5.122965	1.443022	4.362206
H	5.847335	1.530401	2.755618
C	4.660203	2.999736	-3.241410
H	4.705648	2.468738	-4.198451
H	4.403922	4.042056	-3.459819
H	5.663489	2.992958	-2.803243
C	2.225319	2.391880	-2.949892
H	1.490655	1.877929	-2.322553
H	1.886517	3.425840	-3.074911
H	2.230943	1.916257	-3.936866
C	0.770985	-3.745762	2.079212
H	0.831093	-3.587584	3.160832
H	0.244899	-2.893781	1.637647
H	0.171067	-4.645387	1.904184
C	2.937317	-5.041130	2.147026
H	3.959999	-5.135432	1.767560
H	2.996287	-4.884727	3.229625
H	2.432515	-5.998913	1.977971
C	2.131792	-1.452111	-3.952796
H	1.546340	-0.918906	-3.197397
H	2.565363	-0.715069	-4.638187
H	1.441096	-2.079856	-4.526521
C	4.092034	-2.984590	-4.377672
H	4.580496	-2.231031	-5.004957
H	4.872662	-3.614841	-3.939330
H	3.490987	-3.617090	-5.039745
C	-1.598611	1.348836	-4.078538
H	-0.958053	2.020671	-4.660614
H	-0.983828	0.879950	-3.305614
H	-1.960204	0.566699	-4.755383
C	-3.679517	2.690162	-4.557275
H	-4.539116	3.227802	-4.144847
H	-3.131508	3.388346	-5.199428
H	-4.062881	1.888043	-5.197496
C	-2.972376	5.345275	1.773936
H	-3.935632	5.492405	1.274388
H	-3.162784	5.266131	2.849827
H	-2.375789	6.249905	1.611723
C	-0.913249	3.904498	2.009528
H	-0.240598	4.751677	1.835523

H	-1.082515	3.823219	3.088778
H	-0.400577	2.996796	1.677153
C	-3.022747	-2.661838	3.605267
H	-1.995158	-2.491657	3.274798
H	-3.181854	-2.082641	4.520250
H	-3.126097	-3.720871	3.866836
C	-5.463815	-2.513479	3.026177
H	-5.633394	-3.587748	3.163650
H	-5.631387	-2.024080	3.992043
H	-6.222428	-2.142028	2.331966
C	-4.173935	-2.587743	-3.596116
H	-4.063612	-3.647921	-3.850047
H	-5.199979	-2.439054	-3.245418
H	-4.046785	-2.012992	-4.520113
C	-1.712801	-2.355174	-3.077392
H	-0.973188	-1.998990	-2.355467
H	-1.506259	-3.411702	-3.278687
H	-1.572407	-1.804747	-4.013555

TS-5_AlPdAl_WB97X.log

SCF (wB97x) = -3040.81254804
E(SCF)+ZPE(0 K) = -3039.437379
H(298 K) = -3039.355939
G(298 K) = -3039.553916
Lowest Frequency = -233.7298 cm^{-1}

Pd	-0.065636	-0.063466	0.705236
Al	2.271256	-0.458573	0.179689
N	3.905342	0.652884	0.188790
N	3.304527	-2.033178	-0.405884
C	5.164071	0.223592	0.108586
C	5.505112	-1.127790	-0.048229
H	6.562050	-1.366069	-0.037030
C	4.629020	-2.180099	-0.343778
C	6.298541	1.217289	0.173071
H	7.260602	0.721472	0.037563
H	6.301123	1.739976	1.133742
H	6.178143	1.982408	-0.600067
C	5.250882	-3.518520	-0.665069
H	6.290092	-3.549491	-0.333478
H	5.227861	-3.698806	-1.745181
H	4.694378	-4.333104	-0.194441
C	3.653296	2.070755	0.287619
C	3.309478	2.785815	-0.875317
C	3.035795	4.150344	-0.761983
H	2.765552	4.713411	-1.652657
C	3.091018	4.795923	0.464159
C	3.407002	4.072081	1.605581
H	3.425825	4.574790	2.570125
C	3.683972	2.706037	1.544407
C	2.521454	-3.096799	-0.986463
C	1.758190	-3.949823	-0.169970
C	0.997916	-4.948730	-0.785209
H	0.403137	-5.616509	-0.166372
C	0.982497	-5.100729	-2.162465
C	1.722035	-4.233977	-2.957157
H	1.688886	-4.341114	-4.038775
C	2.492476	-3.219178	-2.392264
C	-0.292247	-0.233762	2.812708
C	-1.376209	0.324193	3.262043
C	0.458911	-0.717803	3.885019
C	-1.968340	0.583696	4.466240
C	0.045725	-0.574301	5.206359
C	-1.158776	0.081060	5.491929
H	-2.912574	1.087251	4.618705
H	0.666977	-0.967857	6.004537
H	-1.475874	0.199806	6.524318
H	-0.870478	0.292963	-0.729914
F	1.639527	-1.342670	3.666055
F	-2.865188	0.985499	2.017157
C	1.735876	-3.843079	1.347847
H	2.266174	-2.931455	1.639390
C	3.221377	-2.239575	-3.305464
H	3.962246	-1.694169	-2.711830
C	3.215939	2.131834	-2.247556
H	3.404880	1.058588	-2.129388
C	3.944745	1.949827	2.839898
H	4.221822	0.921009	2.586251
Al	-2.372554	0.593659	0.345120
N	-3.121748	2.109579	-0.573534
N	-3.769947	-0.652820	-0.144644
C	-4.994250	-0.223910	-0.424787
C	-5.329490	1.143101	-0.534524
C	-4.448413	2.220533	-0.690745
C	-5.048532	3.551293	-1.075218
C	-2.710793	2.265698	-3.465551
H	-3.445219	1.655782	-2.929564
H	-4.871291	3.762489	-2.134483
H	-6.125366	3.553585	-0.899084
H	-4.584806	4.361394	-0.505235
H	-5.927807	-2.191256	-0.313443
C	-6.108442	-1.199197	-0.726473
H	-7.057914	-0.812814	-0.347865
H	-6.205537	-1.299644	-1.813342
H	-6.383044	1.362868	-0.665150
C	-2.278505	3.157038	-1.090060
C	-2.094136	3.256664	-2.485795
C	-1.265591	4.262573	-2.980977
C	-0.610949	5.141597	-2.128091
C	-0.777304	5.013663	-0.758435
C	-1.609488	4.031607	-0.214065
H	-1.115648	4.348535	-4.054987
C	-1.748657	3.952312	1.299114
H	-0.250126	5.689440	-0.088435
H	-2.432136	3.135661	1.543354
C	-3.475472	-2.065532	-0.118862
C	-2.990514	-2.689833	-1.282174
C	-2.715808	-1.927334	-2.570137
C	-3.628759	-2.785871	1.083859
C	-3.376083	-4.158573	1.065483
C	-2.945225	-4.799507	-0.087830
C	-2.737861	-4.062554	-1.243722
H	-2.365501	-4.559253	-2.136819
H	-2.852199	-0.859126	-2.367813
C	-4.057525	-2.125495	2.389871
H	-3.909065	-1.046338	2.288902
H	-3.509197	-4.735043	1.977423
H	2.877626	5.859896	0.532755
H	0.387631	-5.888004	-2.619486
H	0.034080	5.918319	-2.532314
H	-2.755119	-5.870131	-0.079443
C	2.659668	1.890137	3.675536
H	2.372725	2.891916	4.017760
H	1.830848	1.486970	3.086888
H	2.797741	1.255339	4.558069
C	5.089812	2.556513	3.659693
H	4.844361	3.568753	3.999624
H	5.279859	1.948529	4.550491
H	6.022347	2.618317	3.088397
C	4.285709	2.676017	-3.202165
H	4.226741	2.169439	-4.172085
H	4.150353	3.749411	-3.377326
H	5.295737	2.527782	-2.804183
C	1.812224	2.295964	-2.842021
H	1.047419	1.894246	-2.167285
H	1.573172	3.350071	-3.020713
H	1.739356	1.769531	-3.801033
C	0.304484	-3.719009	1.881340
H	0.323483	-3.550101	2.963665
H	-0.213404	-2.873227	1.413845
H	-0.282921	-4.624882	1.691312
C	2.462565	-5.028518	1.995586
H	3.505501	-5.094701	1.665852
H	2.460287	-4.927266	3.086149
H	1.972514	-5.976531	1.744341
C	2.232218	-1.205708	-3.860127
H	1.700797	-0.686448	-3.053927
H	2.753854	-0.456454	-4.468356
H	1.477615	-1.692049	-4.489857
C	3.976492	-2.930765	-4.445893

H	4.567230	-2.197634	-5.005077
H	4.658254	-3.702845	-4.072657
H	3.293030	-3.406953	-5.157175
C	-1.628249	1.316565	-3.995030
H	-0.872547	1.869210	-4.566470
H	-1.117937	0.804390	-3.172084
H	-2.067475	0.560065	-4.657162
C	-3.440311	2.950985	-4.626854
H	-4.201044	3.655997	-4.274534
H	-2.747135	3.506310	-5.268149
H	-3.937215	2.203397	-5.254306
C	-2.352359	5.239339	1.874147
H	-3.331253	5.456374	1.432055
H	-2.484216	5.146416	2.957572
H	-1.704578	6.104379	1.690671
C	-0.399252	3.633082	1.957633
H	0.323879	4.444390	1.810184
H	-0.527241	3.483233	3.036613
H	0.039633	2.718359	1.539826
C	-3.198969	-2.592253	3.572903
H	-2.130340	-2.476098	3.363589
H	-3.434412	-1.997353	4.461795
H	-3.386877	-3.642474	3.823908
C	-5.541715	-2.374352	2.690472
H	-5.750562	-3.448968	2.756588
H	-5.818848	-1.918332	3.647224
H	-6.193940	-1.952008	1.920094
C	-3.698932	-2.323520	-3.678333
H	-3.588384	-3.381839	-3.942061
H	-4.739000	-2.165214	-3.373441
H	-3.516532	-1.732946	-4.583510
C	-1.266583	-2.130336	-3.026393
H	-0.564332	-1.826592	-2.241998
H	-1.062953	-3.178591	-3.271361
H	-1.062452	-1.534326	-3.923076
